# Supplementary material for: Carbene-catalyzed atroposelective synthesis of axially chiral styrenes
Source: Nat Commun. 2022 Jan 10;13:84. doi: 10.1038/s41467-021-27771-x (PMC8748895; doi:10.1038/s41467-021-27771-x)
Supplement: Supplementary file 1 — Supplementary information [file 41467_2021_27771_MOESM1_ESM.pdf]

Supplementary Information

Carbene-Catalyzed Atroposelective Synthesis of Axially Chiral Styrenes

Jia-Lei Yan, Rakesh Maiti, Shi-Chao Ren, Weiyi Tian*, Tingting Li, Jun Xu, Bivas Mondal, Zhichao Jin & Yonggui Robin Chi*

Table of Contents

1. Supplementary Figures S2
2. Supplementary Methods S123
    2.1 General Information S123
    2.2 Preparation of the NHC Pre-catalysts S124
    2.3 Preparation of the Sulfinic Acids S125
    2.4 Preparation of the Ynals S125
3. Supplementary Results of Condition Optimization S133
4. Proposed Reaction Mechanism S135
5. Supplementary Data of the Products S137
    5.1 Characterization of the Axially Chiral Styrenes S137
    5.2 Further Transformations of the Products S153
6. DFT Calculations on the Rotation Barrier S161
7. Experimental Racemization Studies S164
8. Supplementary References S168

1. Supplementary Figures

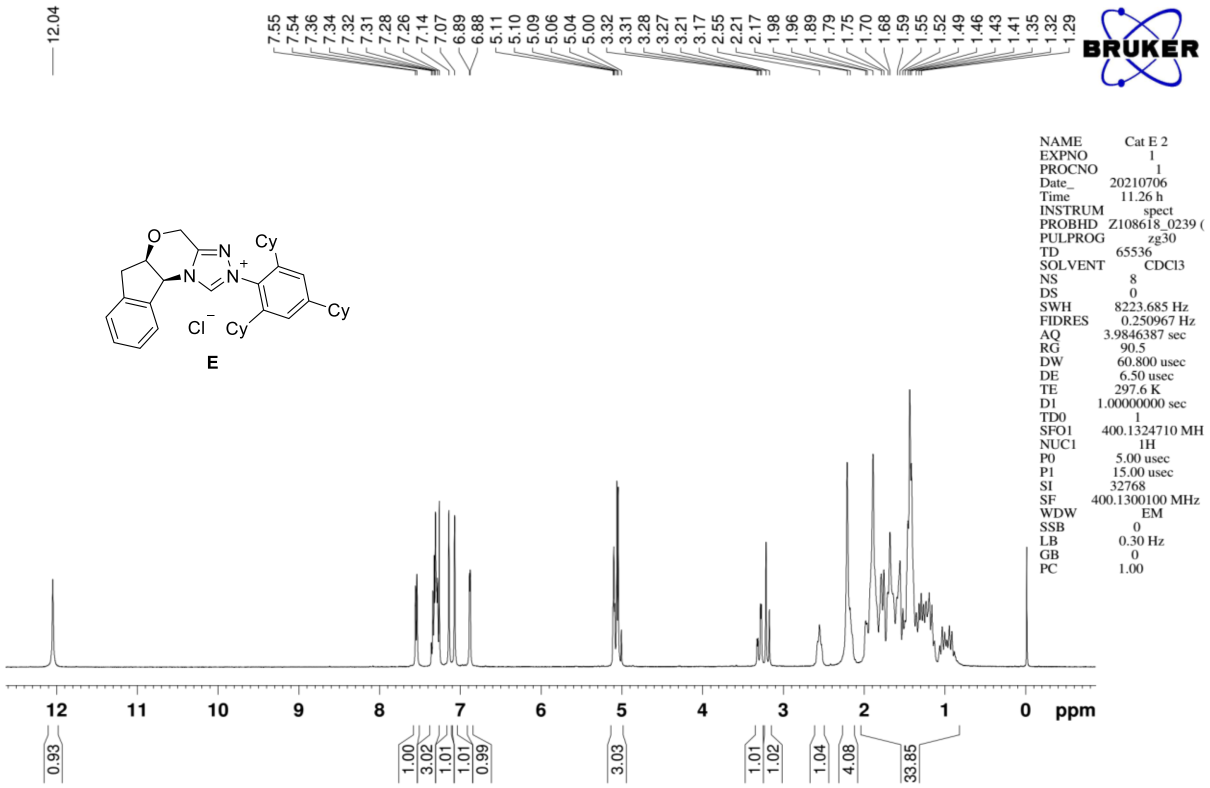

Supplementary Figure 1. ^{1}H NMR ($CDCl_3$, 400MHz) spectra of NHC pre-catalyst **E**

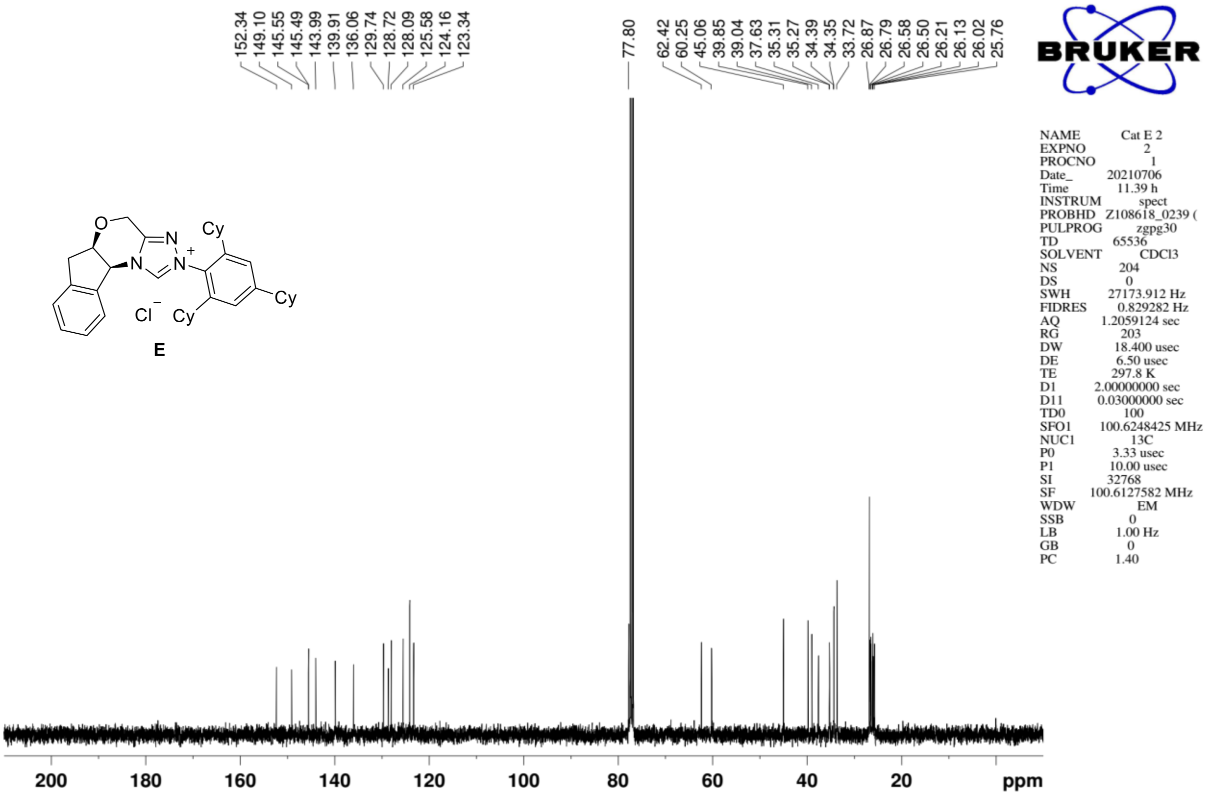

Supplementary Figure 2. ^{13}C NMR ($CDCl_3$, 101MHz) spectra of NHC pre-catalyst **E**

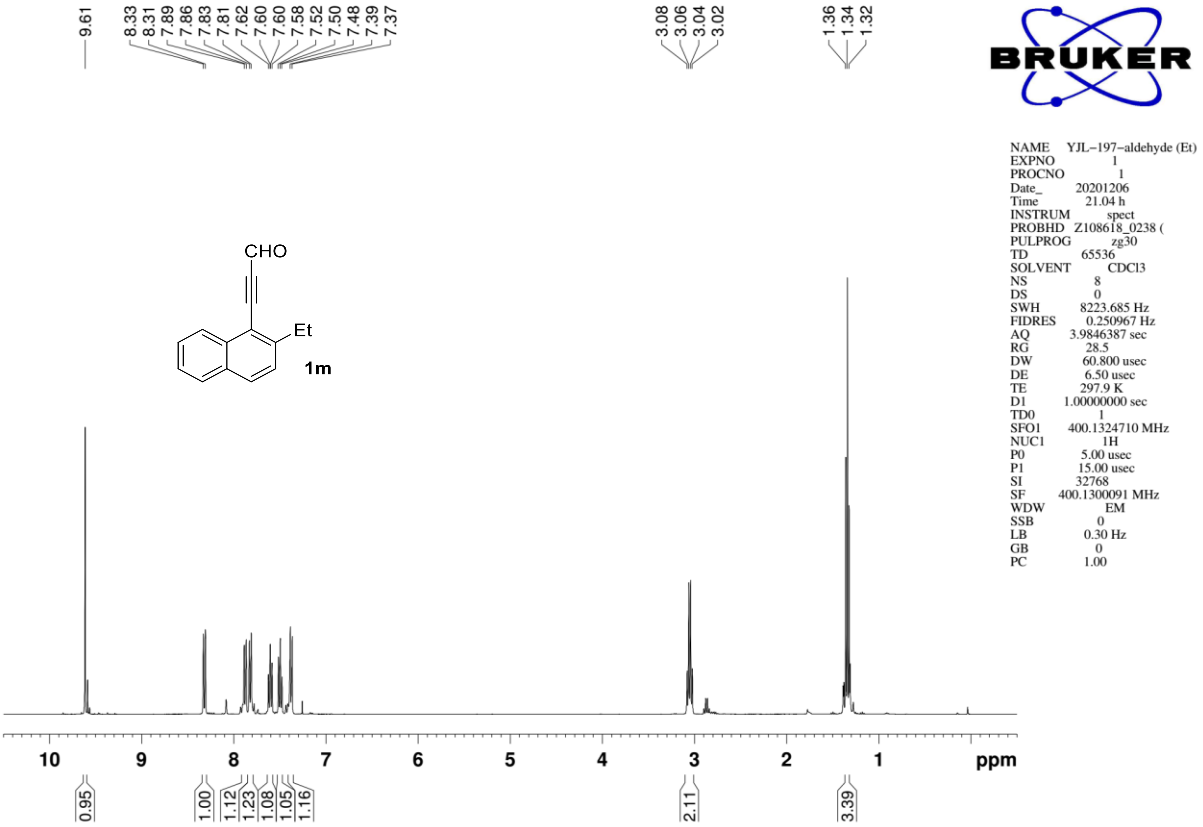

Supplementary Figure 3. ^{1}H NMR ($CDCl_3$, 400MHz) spectra of substrate **1m**

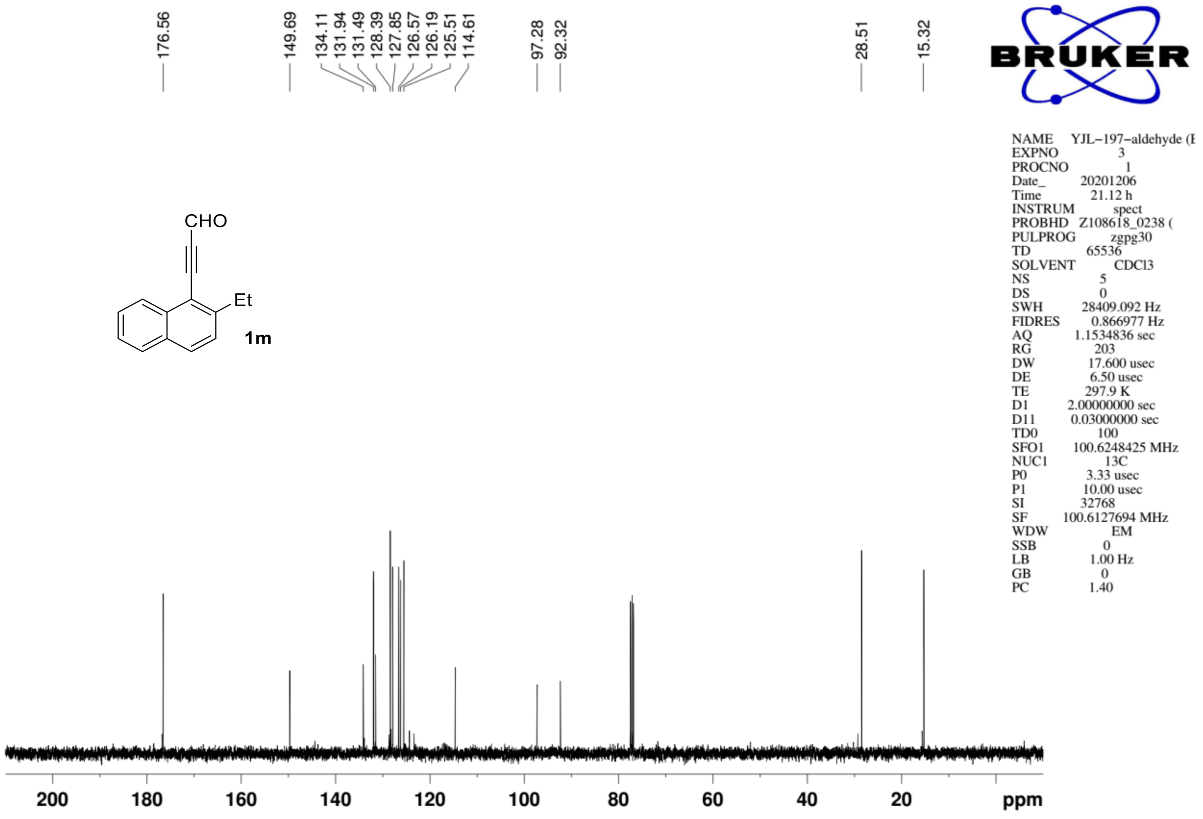

Supplementary Figure 4. ^{13}C NMR ($CDCl_3$, 101MHz) spectra of spectra of substrate **1m**

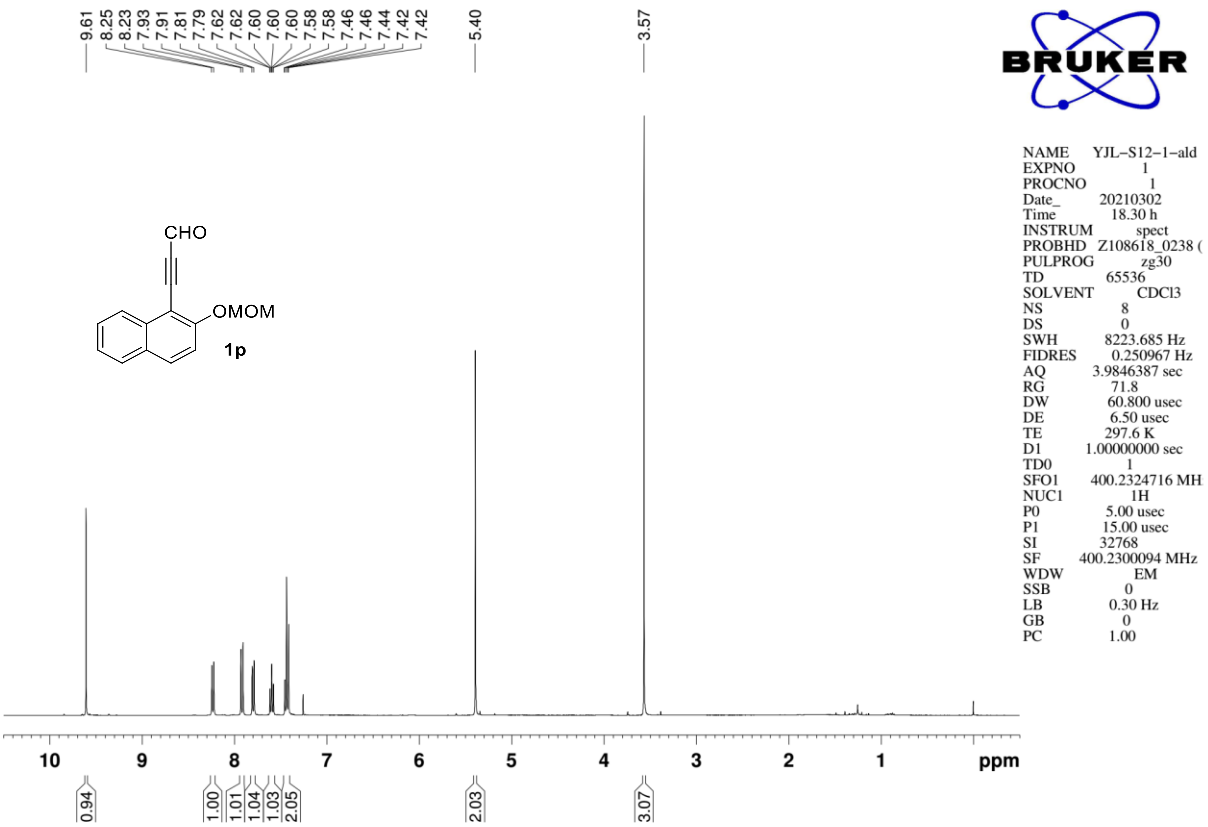

Supplementary Figure 5. ^{1}H NMR ($CDCl_3$, 400MHz) spectra of substrate **1p**

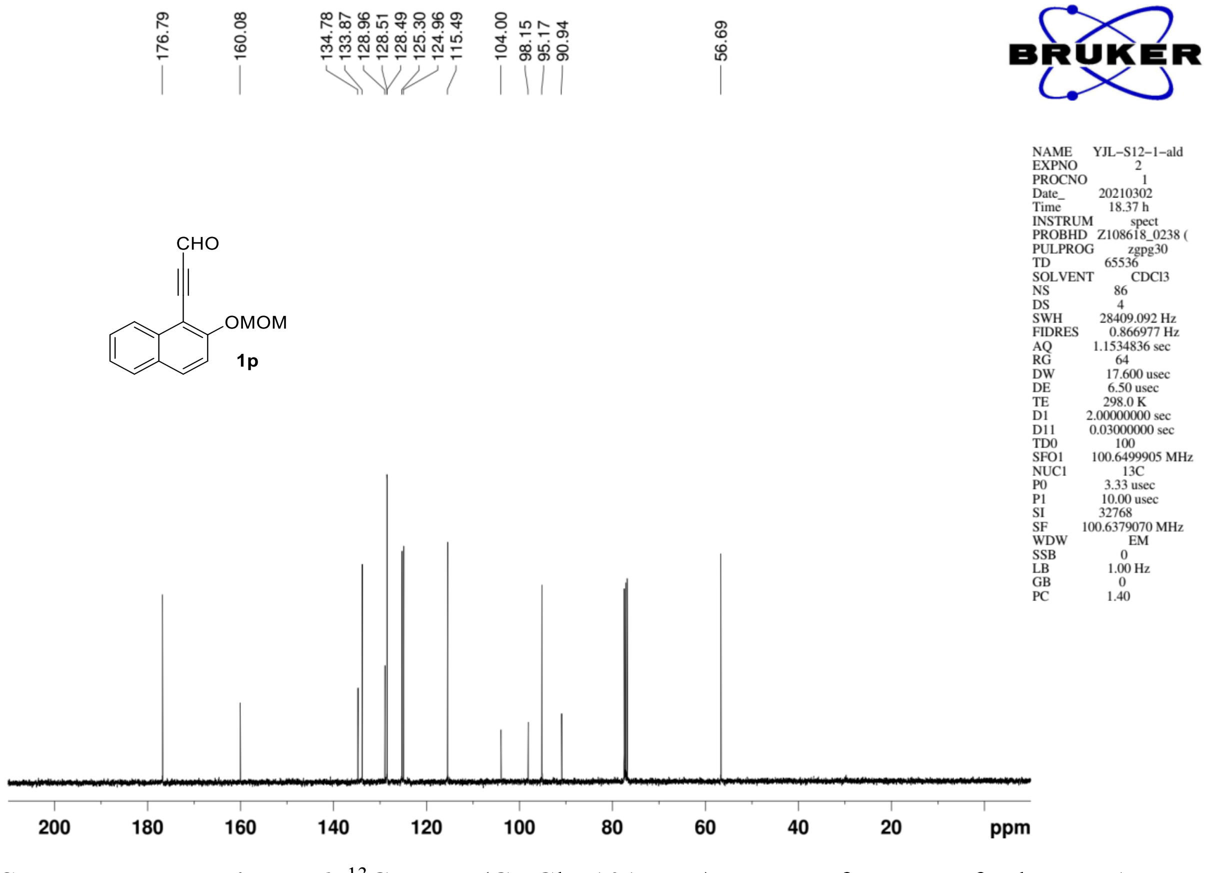

Supplementary Figure 6. ^{13}C NMR ($CDCl_3$, 101MHz) spectra of spectra of substrate **1p**

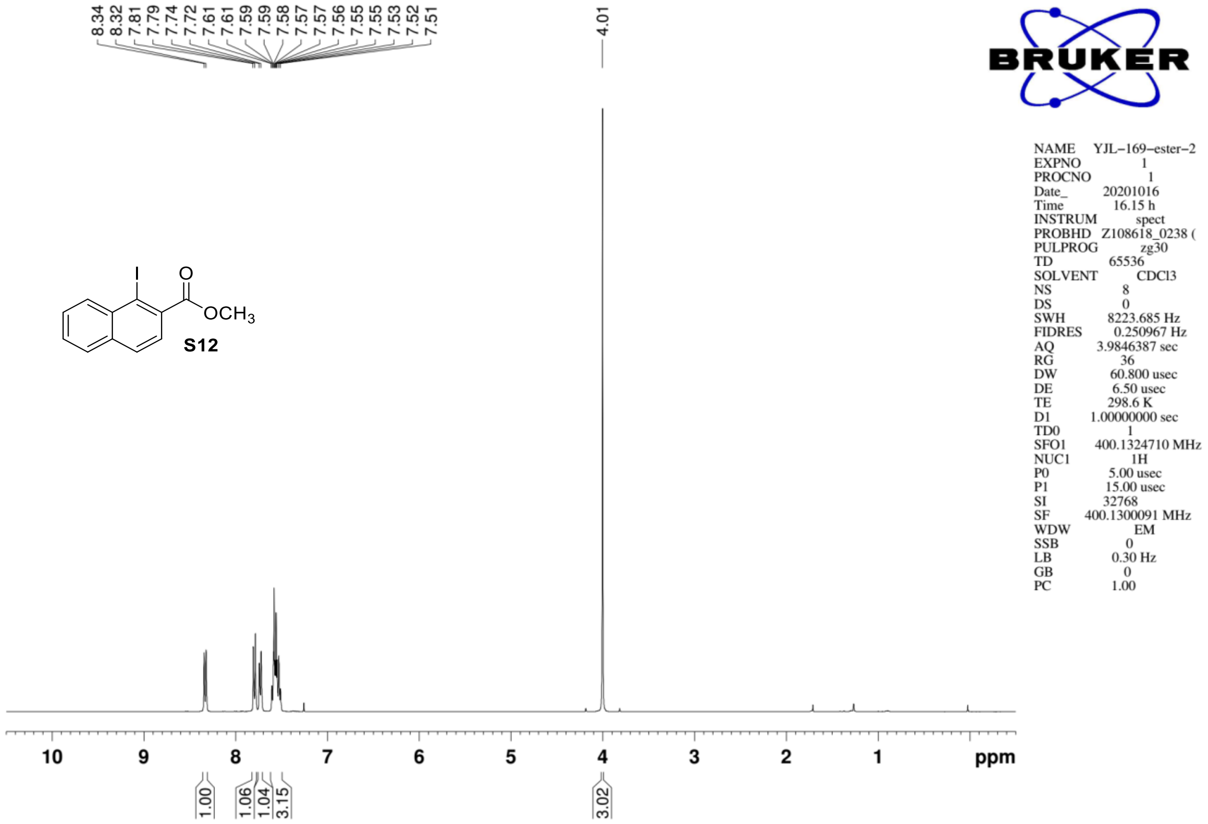

Supplementary Figure 7. ^{1}H NMR ($CDCl_3$, 400MHz) spectra of **S12**

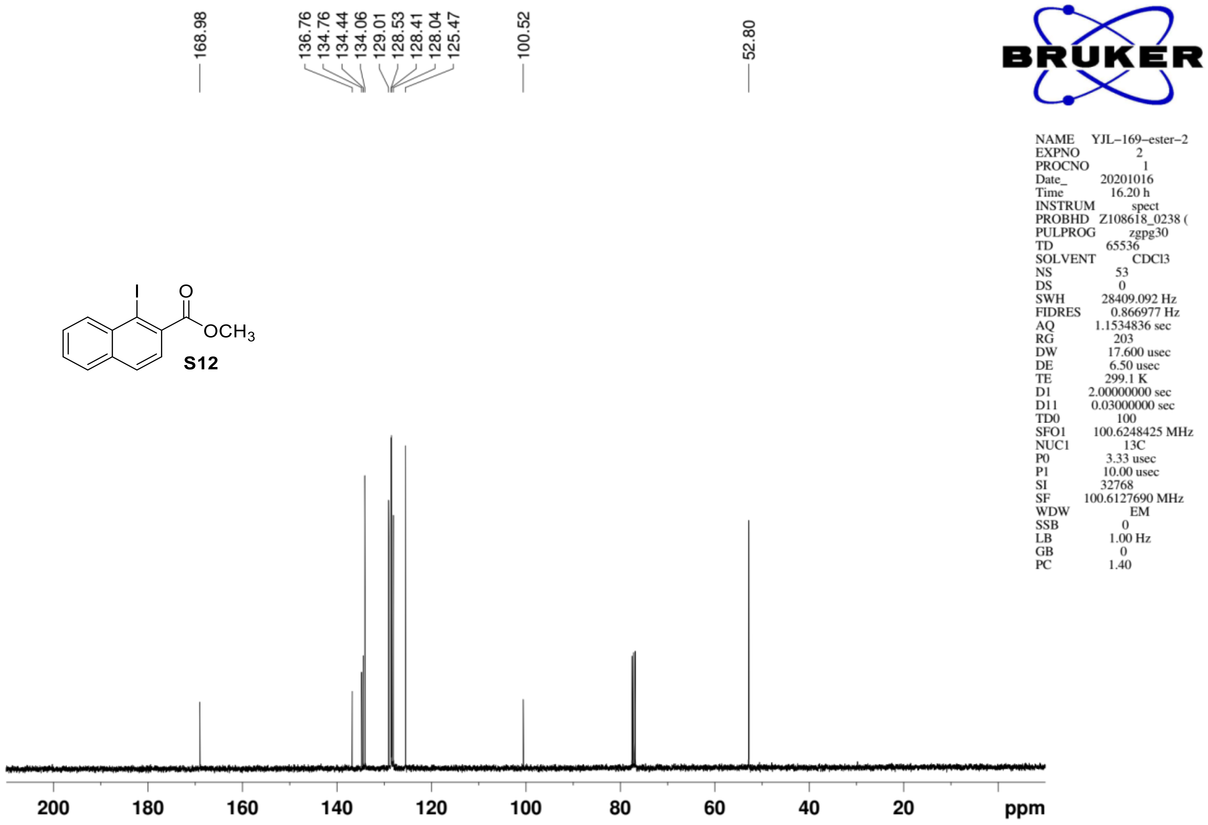

Supplementary Figure 8. ^{13}C NMR ($CDCl_3$, 101MHz) spectra of spectra of **S12**

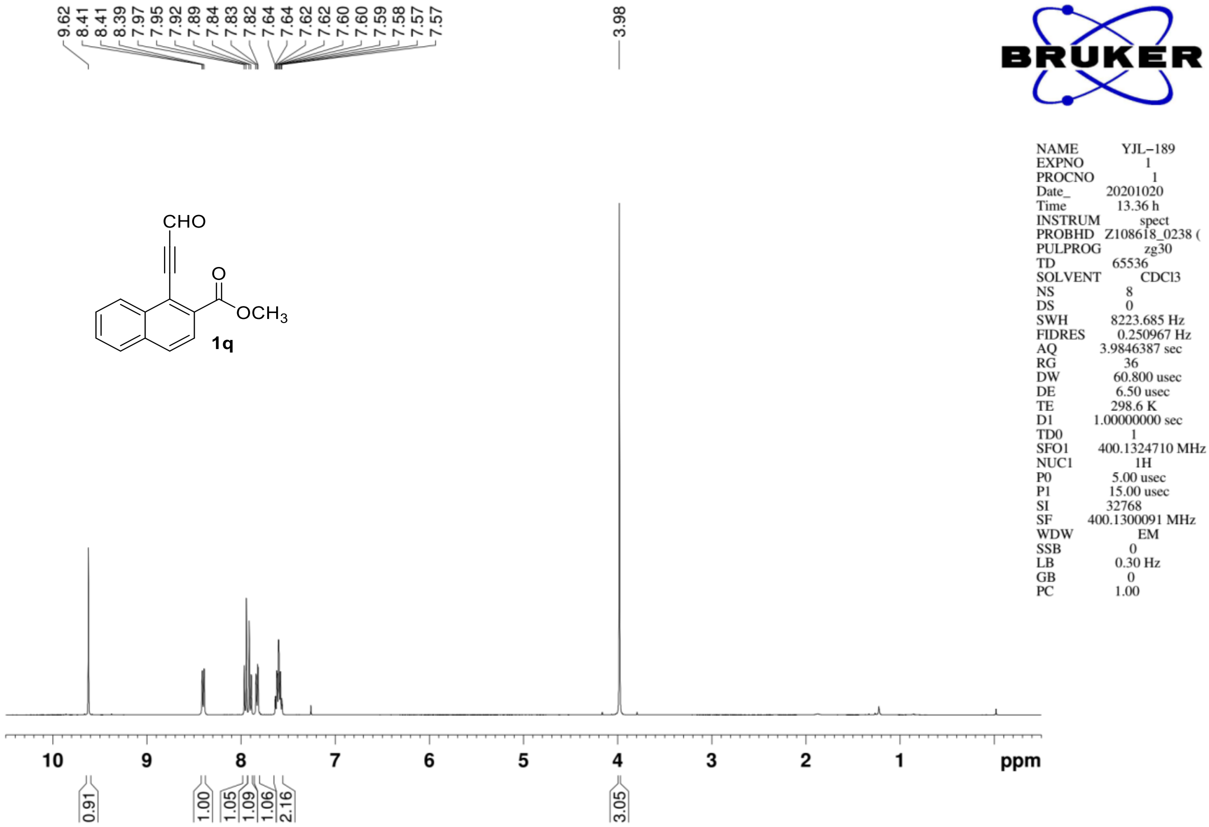

Supplementary Figure 9. ^{1}H NMR ($CDCl_3$, 400MHz) spectra of substrate **1q**

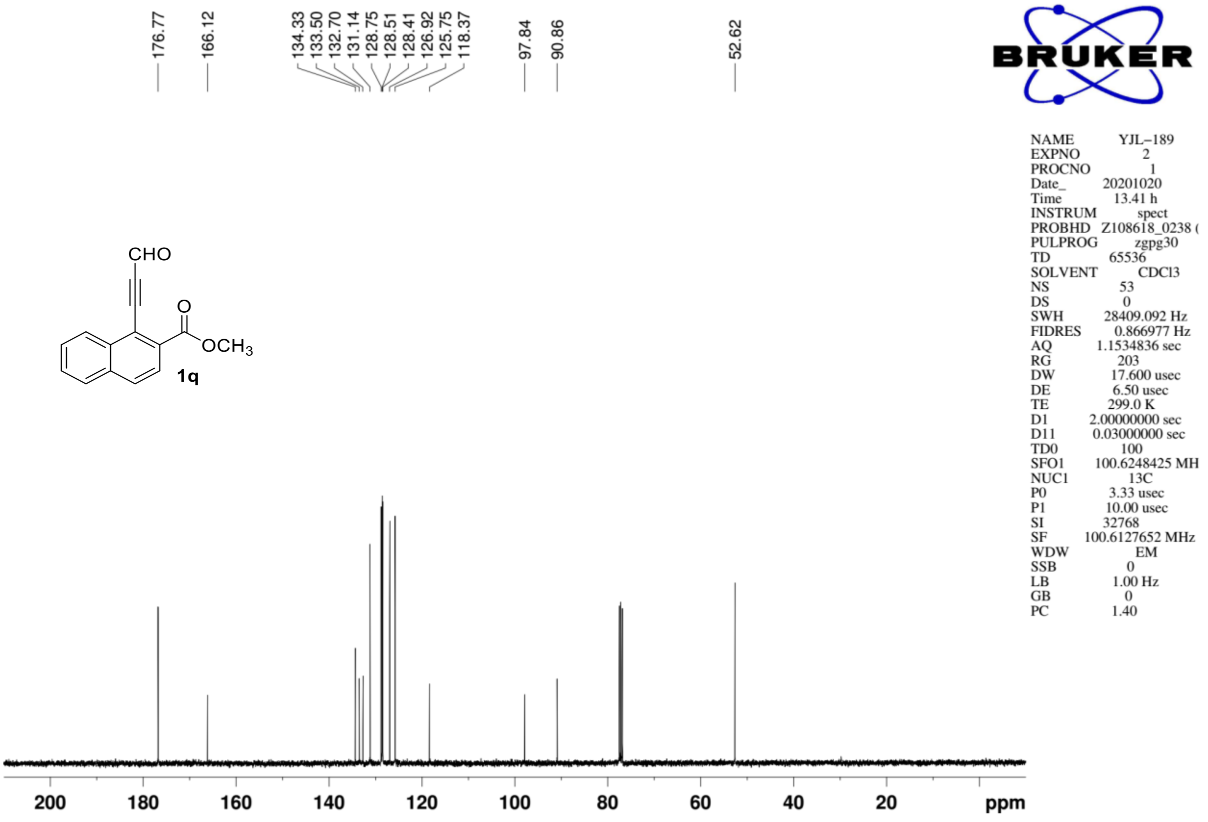

Supplementary Figure 10. ^{13}C NMR ($CDCl_3$, 101MHz) spectra of spectra of substrate **1q**

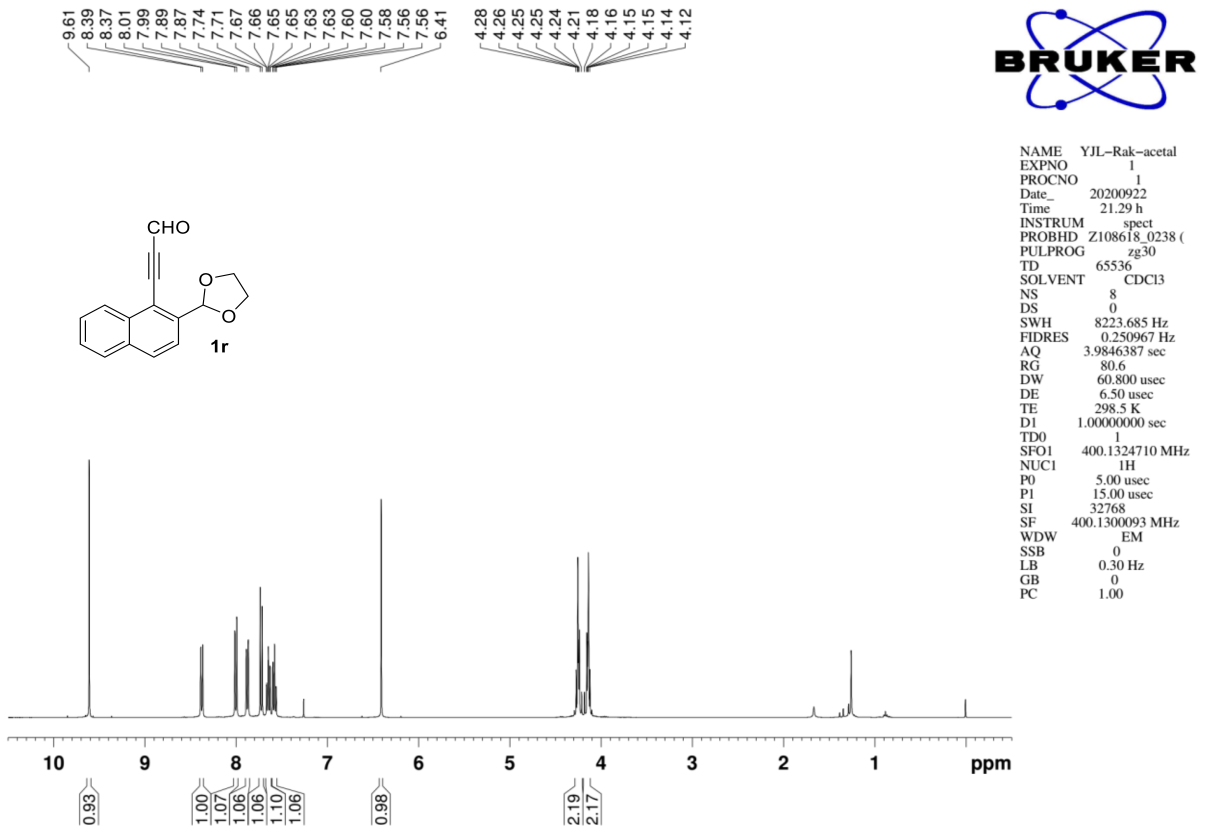

Supplementary Figure 11. ^{1}H NMR ($CDCl_3$, 400MHz) spectra of substrate **1r**

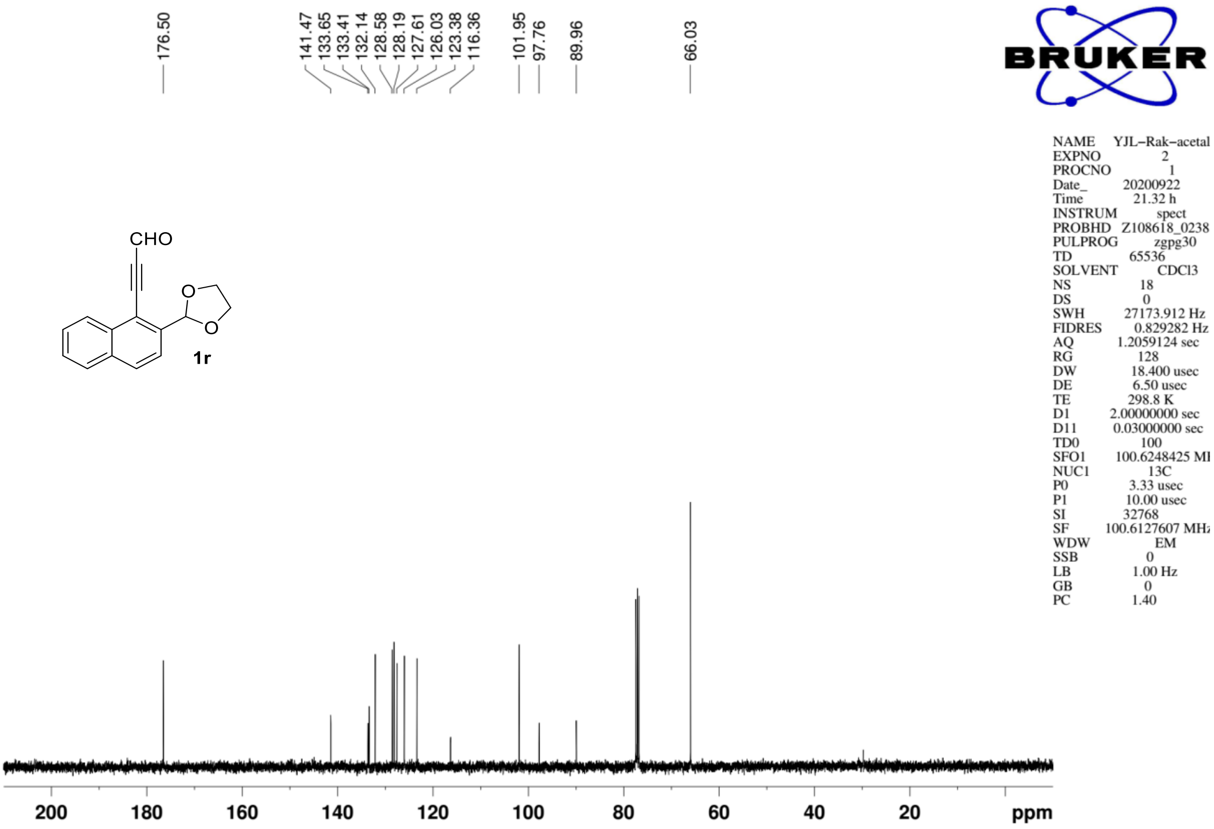

Supplementary Figure 12. ^{13}C NMR ($CDCl_3$, 101MHz) spectra of spectra of substrate **1r**

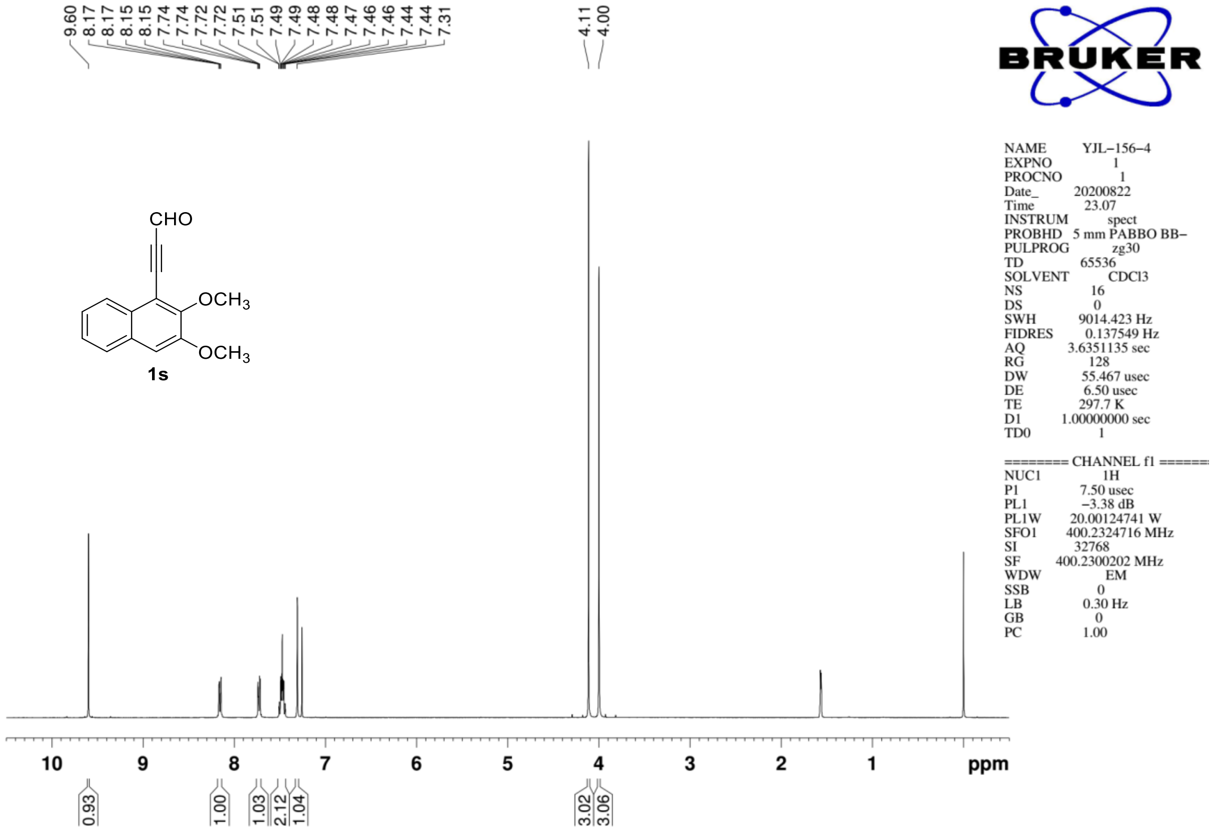

Supplementary Figure 13. ^{1}H NMR ($CDCl_3$, 400MHz) spectra of substrate **1s**

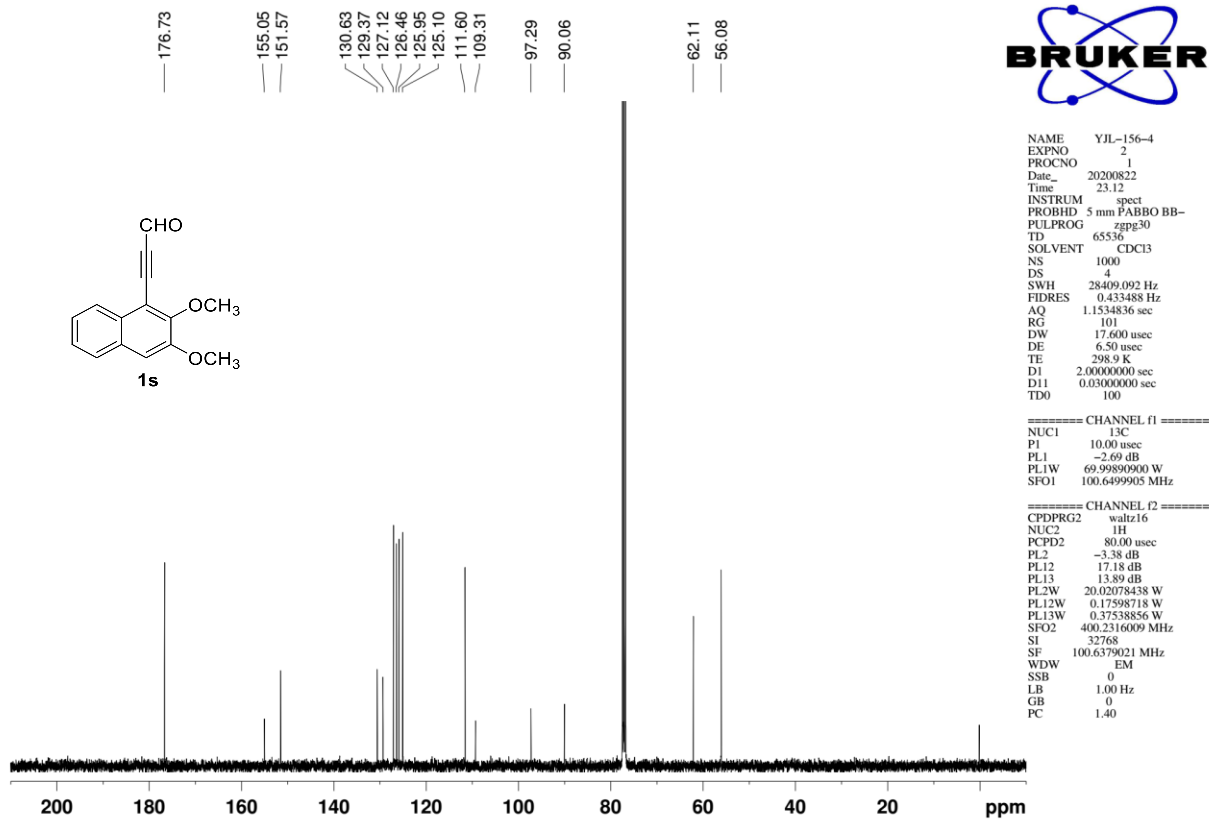

Supplementary Figure 14. ^{13}C NMR ($CDCl_3$, 101MHz) spectra of spectra of substrate **1s**

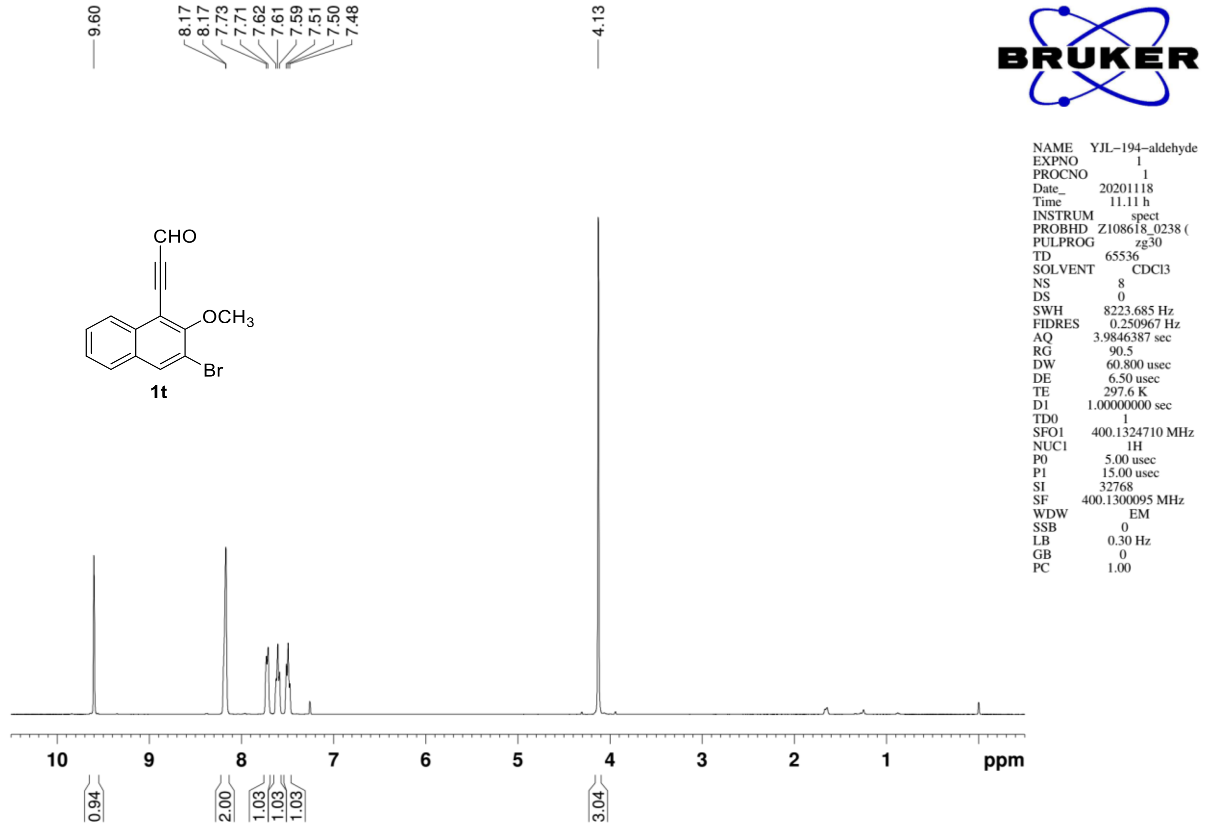

Supplementary Figure 15. ^{1}H NMR ($CDCl_3$, 400MHz) spectra of substrate **1t**

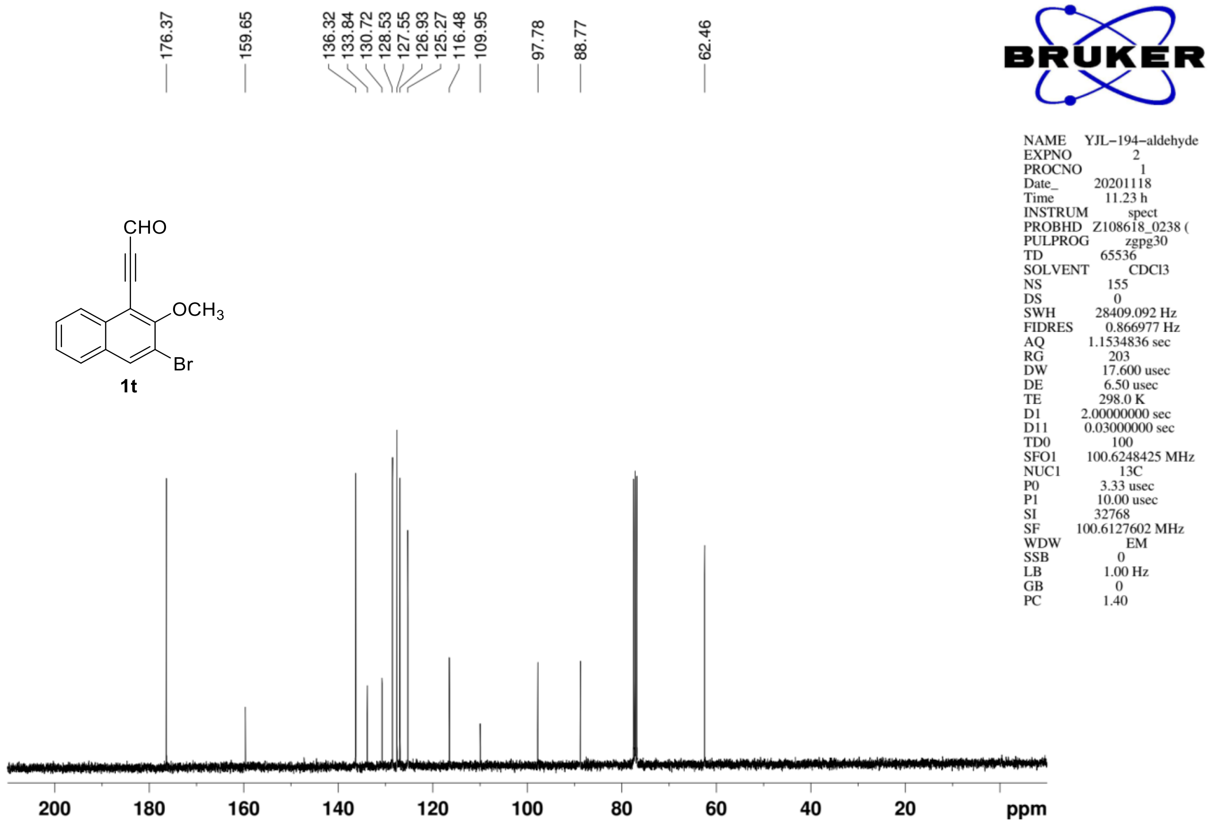

Supplementary Figure 16. ^{13}C NMR ($CDCl_3$, 101MHz) spectra of spectra of substrate **1t**

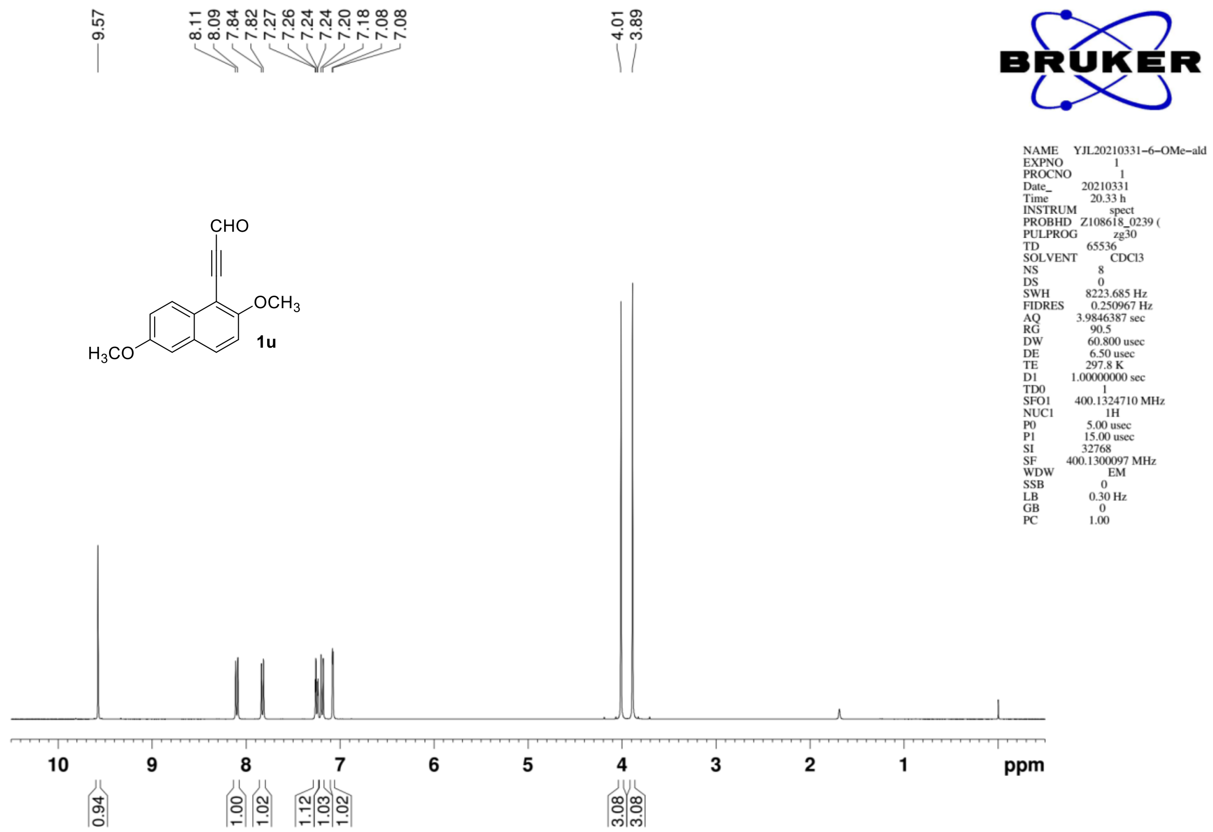

Supplementary Figure 17. ^{1}H NMR ($CDCl_3$, 400MHz) spectra of substrate **1u**

^{13}C NMR ($CDCl_3$, 101MHz)

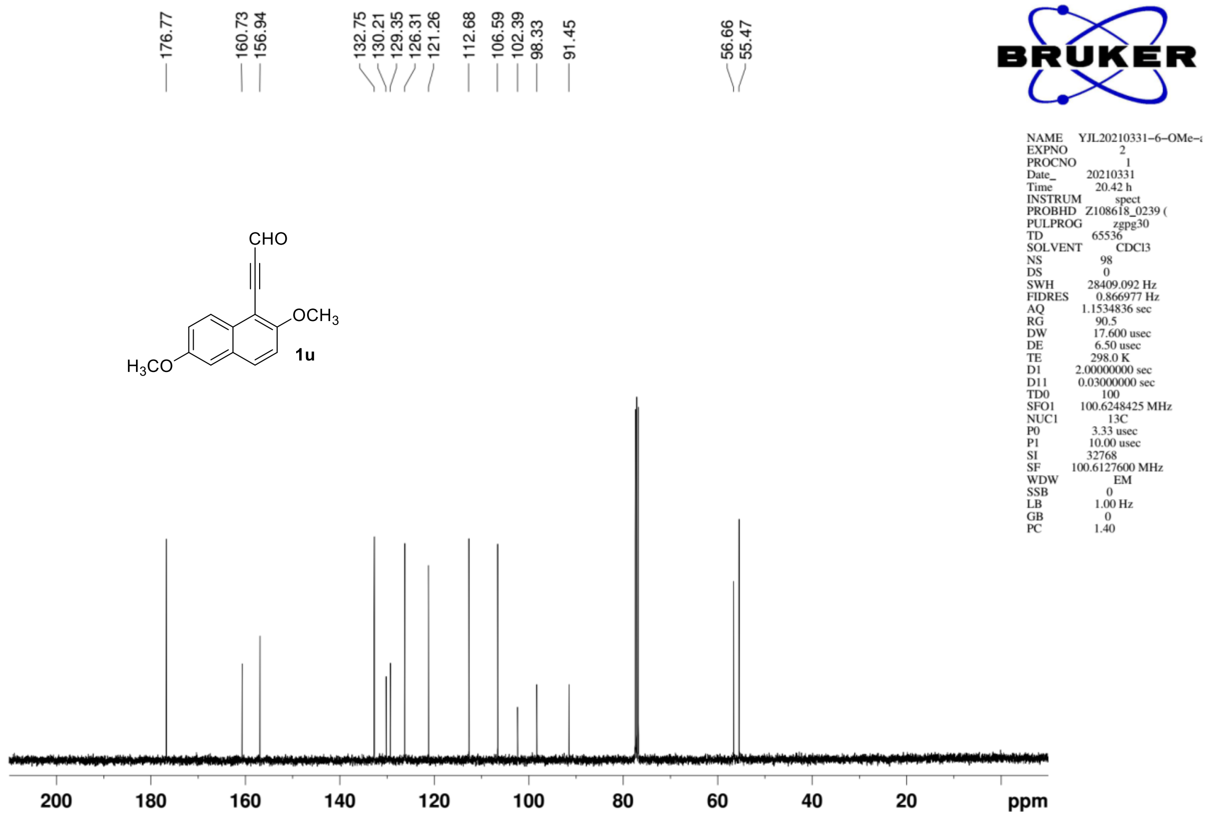

Supplementary Figure 18. ^{13}C NMR ($CDCl_3$, 101MHz) spectra of spectra of substrate **1u**

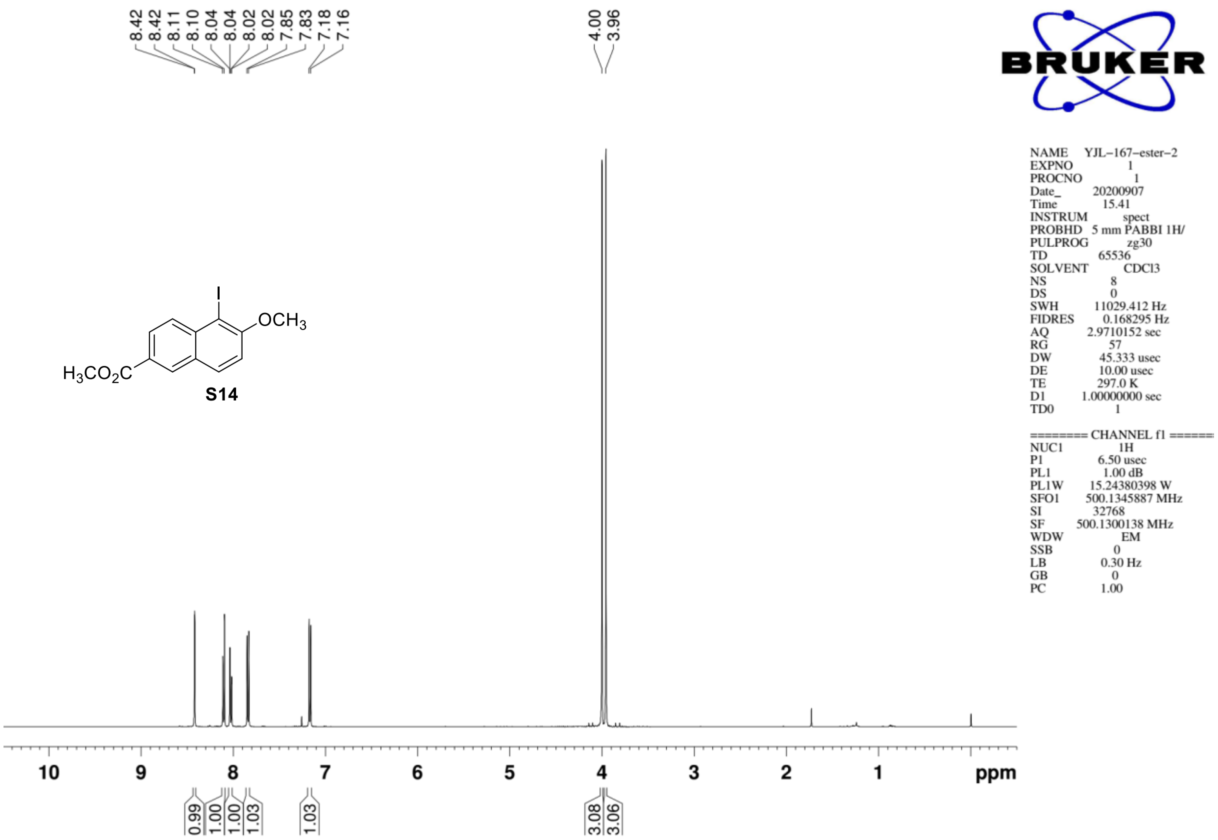

Supplementary Figure 19. ^{1}H NMR ($CDCl_3$, 500MHz) spectra of **S14**

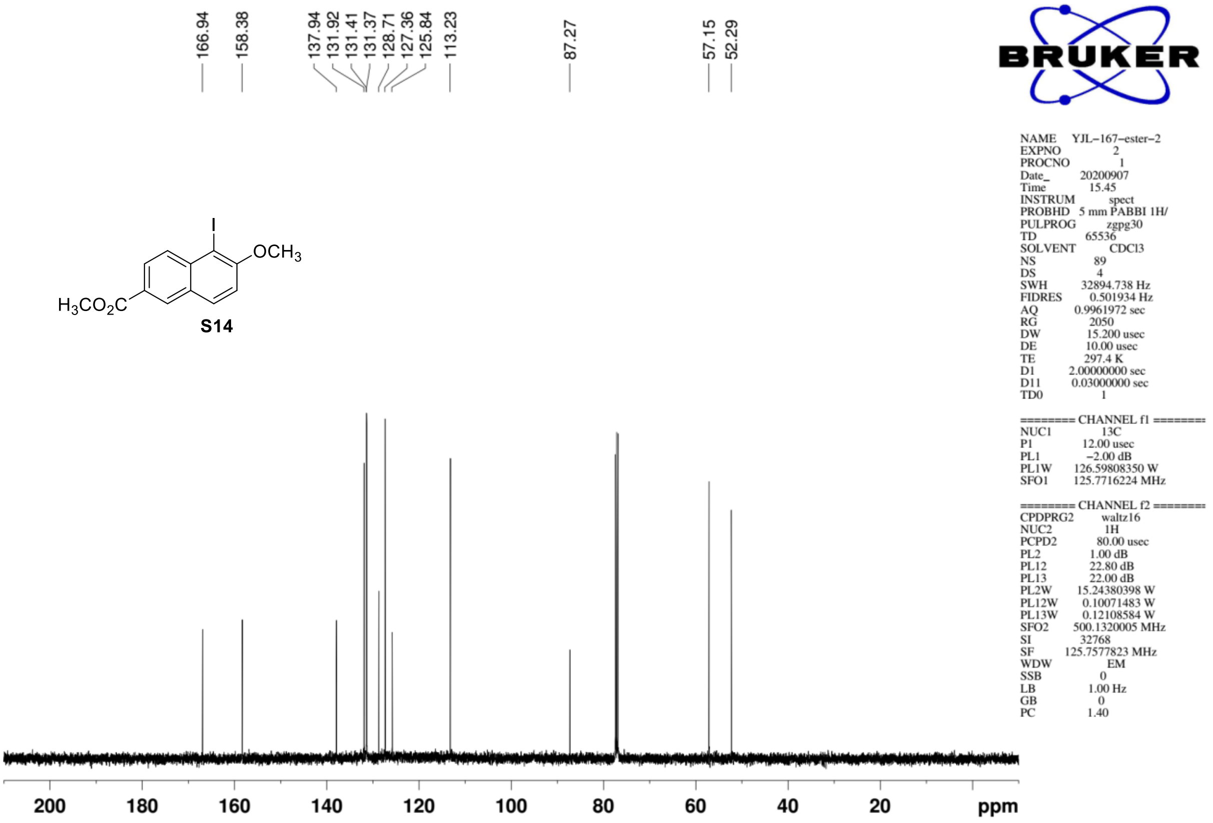

Supplementary Figure 20. ^{13}C NMR ($CDCl_3$, 126MHz) spectra of spectra of **S14**

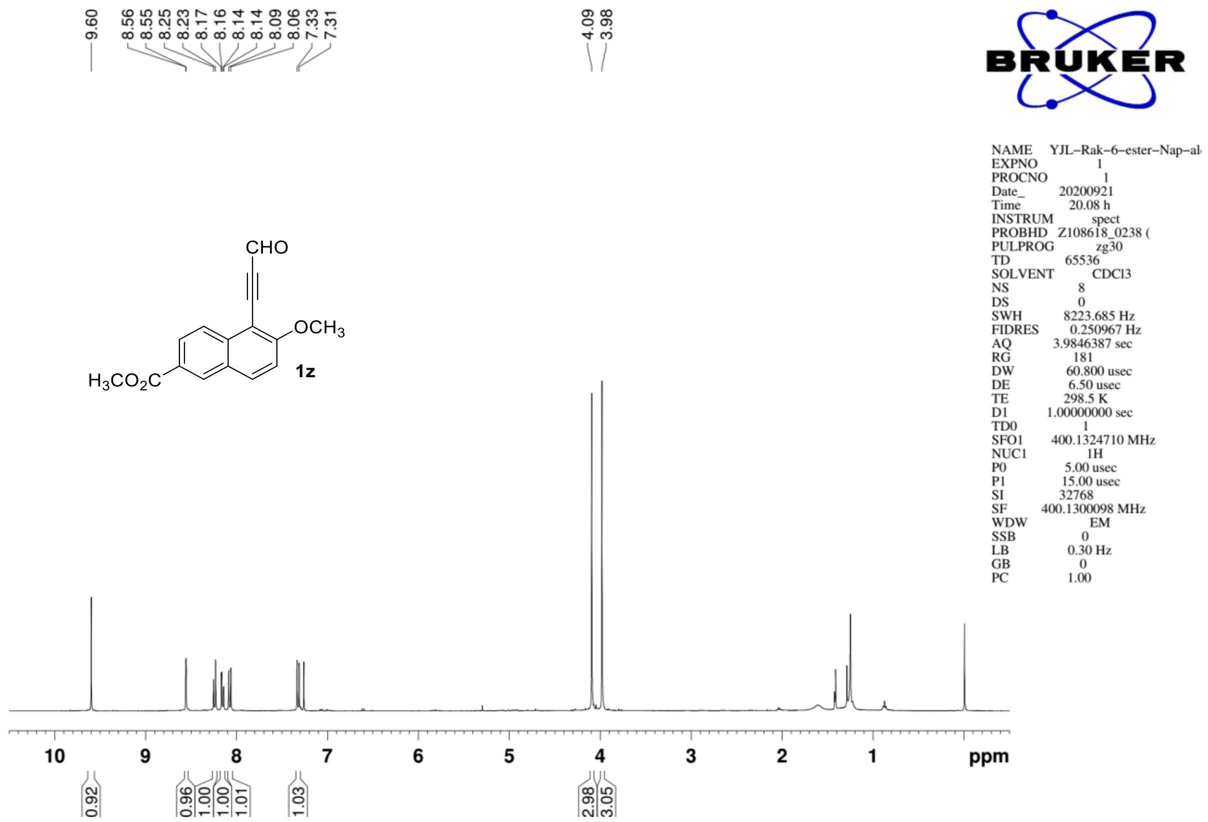

Supplementary Figure 21. ^{1}H NMR ($CDCl_3$, 400MHz) spectra of substrate **1z**

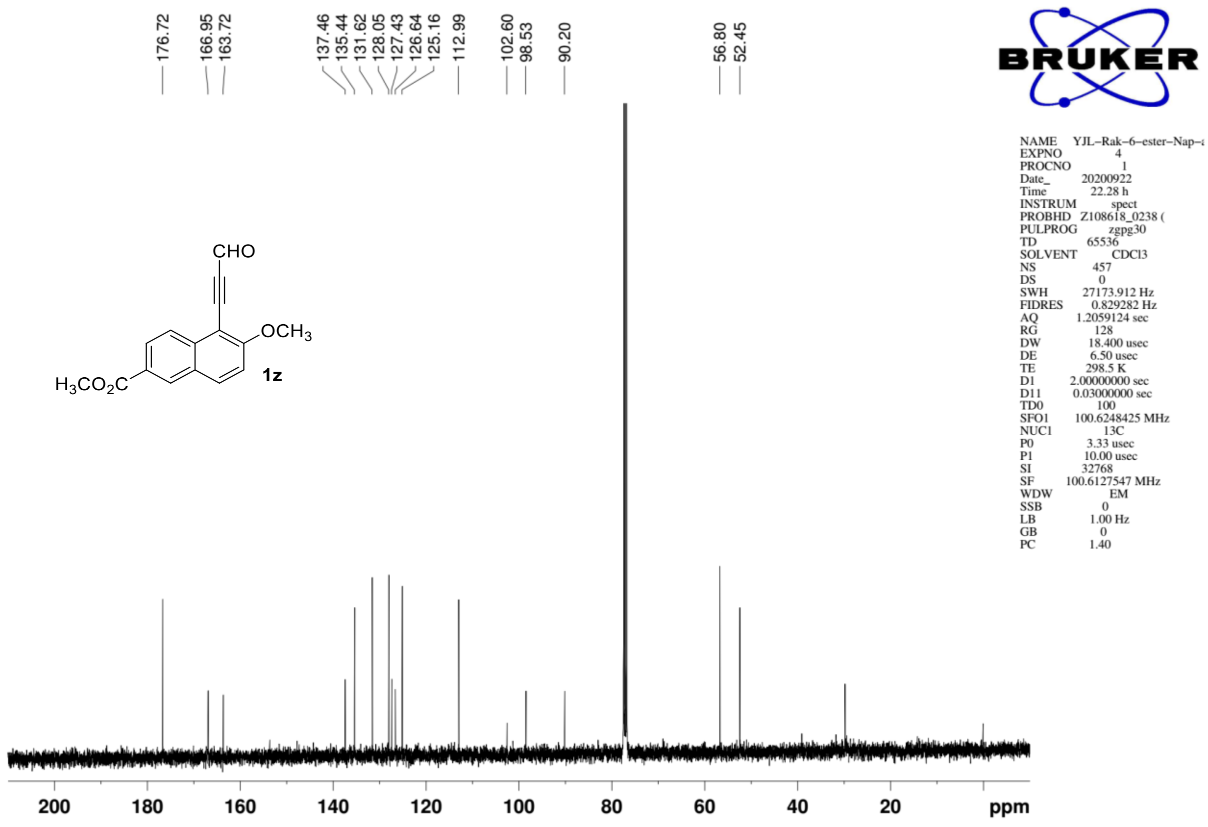

Supplementary Figure 22. ^{13}C NMR ($CDCl_3$, 101MHz) spectra of spectra of substrate **1z**

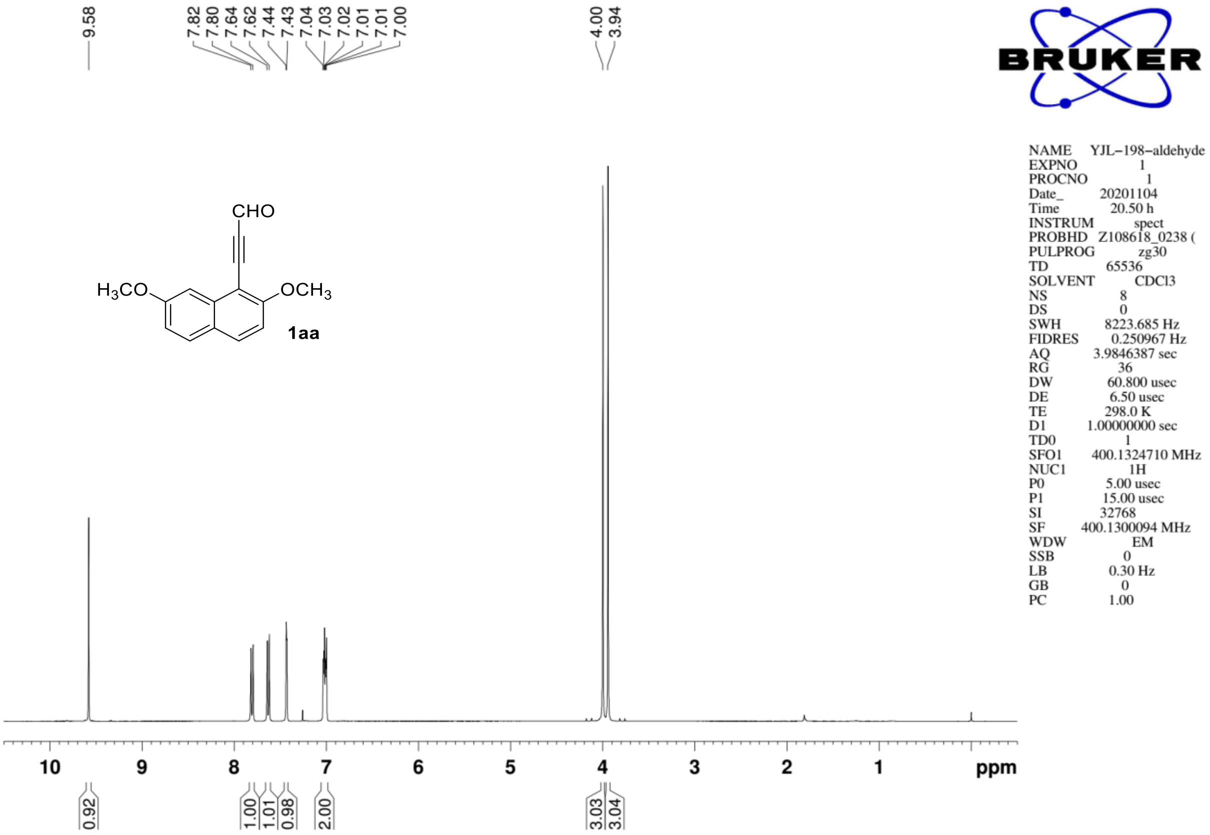

Supplementary Figure 23. ^{1}H NMR ($CDCl_3$, 400MHz) spectra of substrate **1aa**

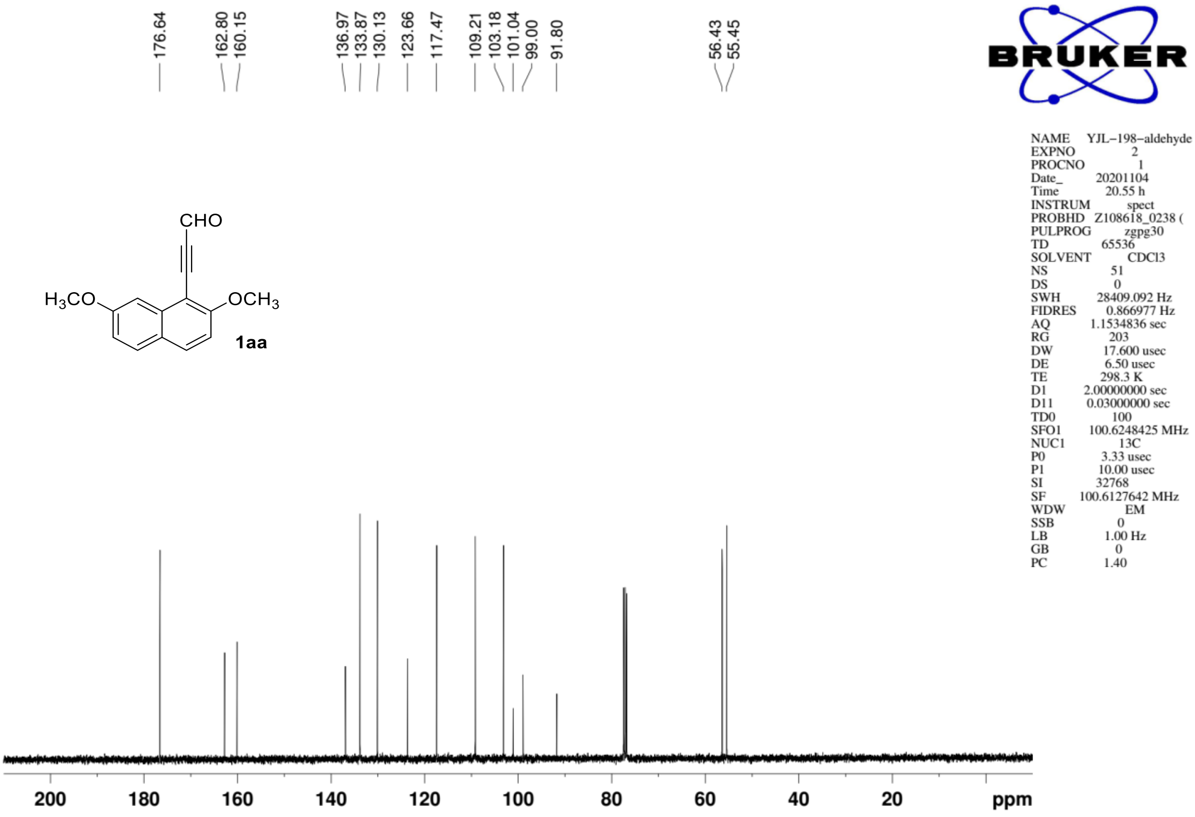

Supplementary Figure 24. ^{13}C NMR ($CDCl_3$, 101MHz) spectra of spectra of substrate **1aa**

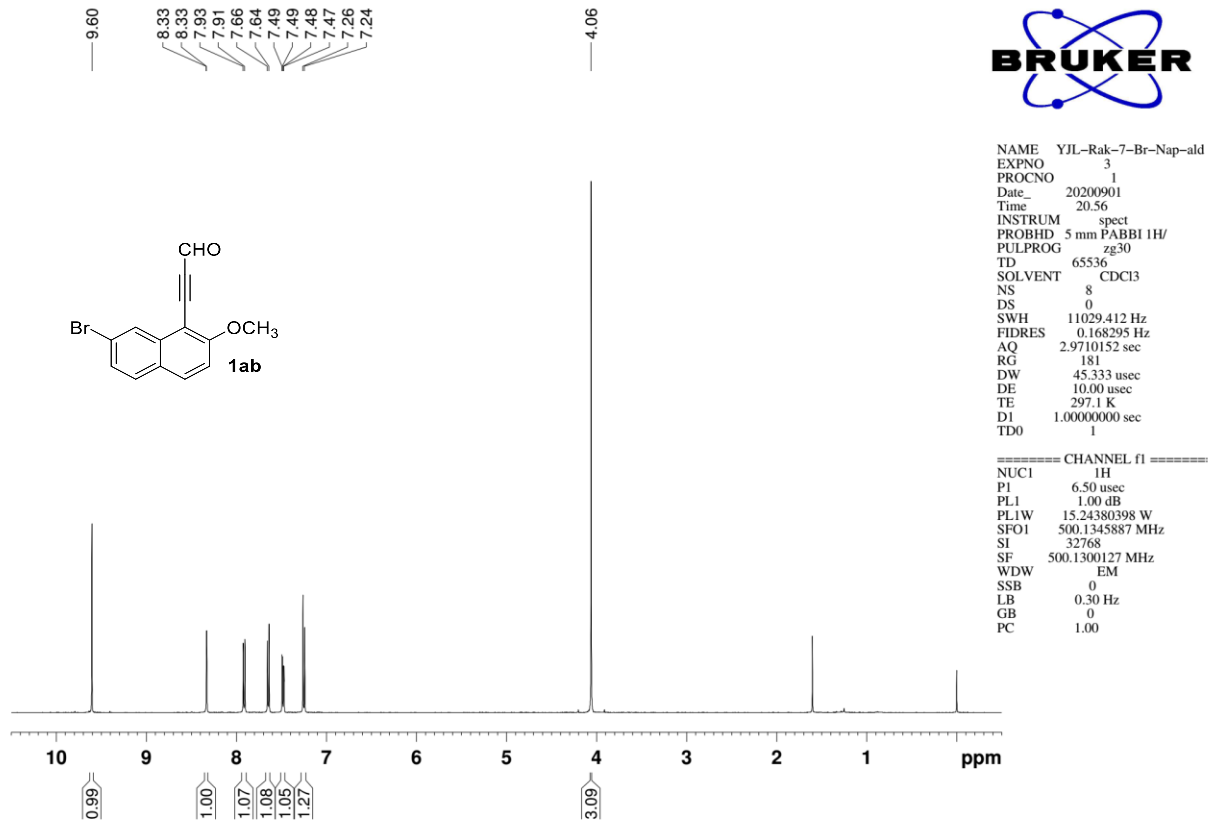

Supplementary Figure 25. ^{1}H NMR ($CDCl_3$, 500MHz) spectra of substrate **1ab**

^{13}C NMR ($CDCl_3$, 126MHz)

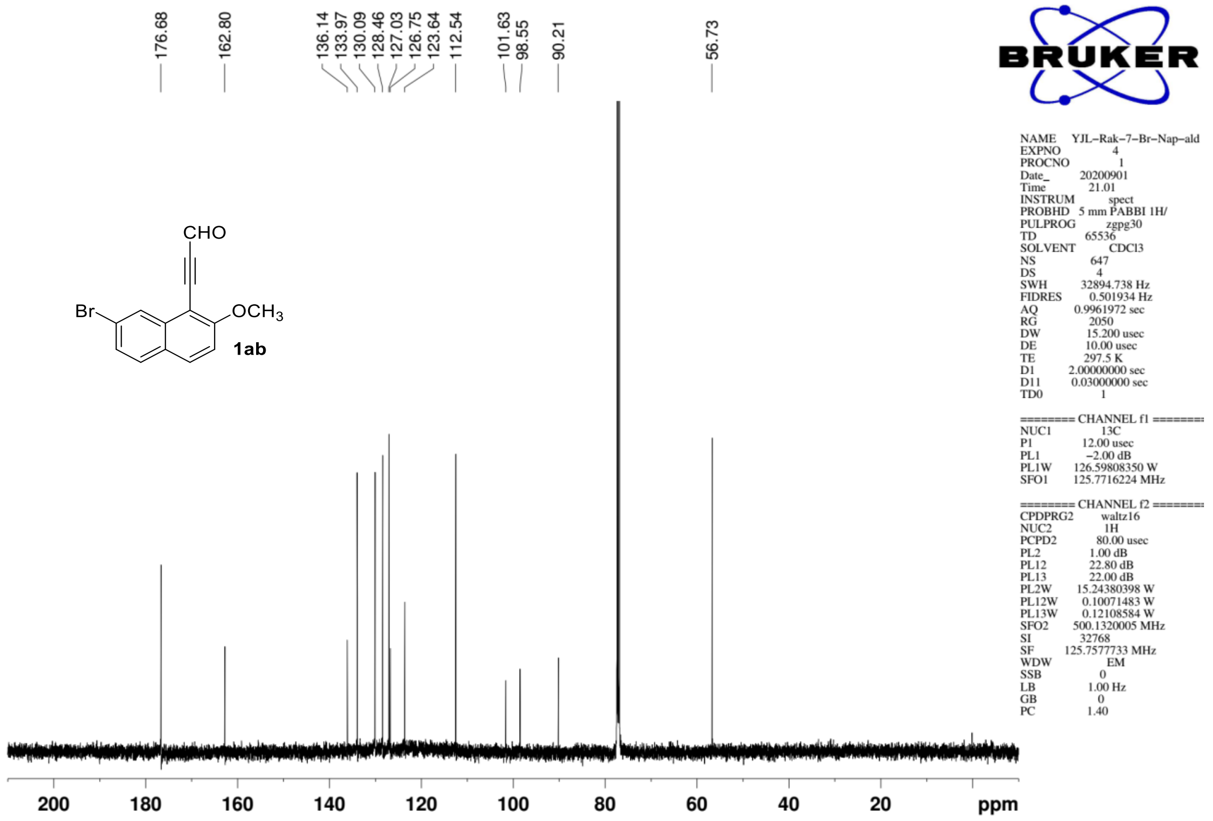

Supplementary Figure 26. ^{13}C NMR ($CDCl_3$, 126MHz) spectra of spectra of substrate **1ab**

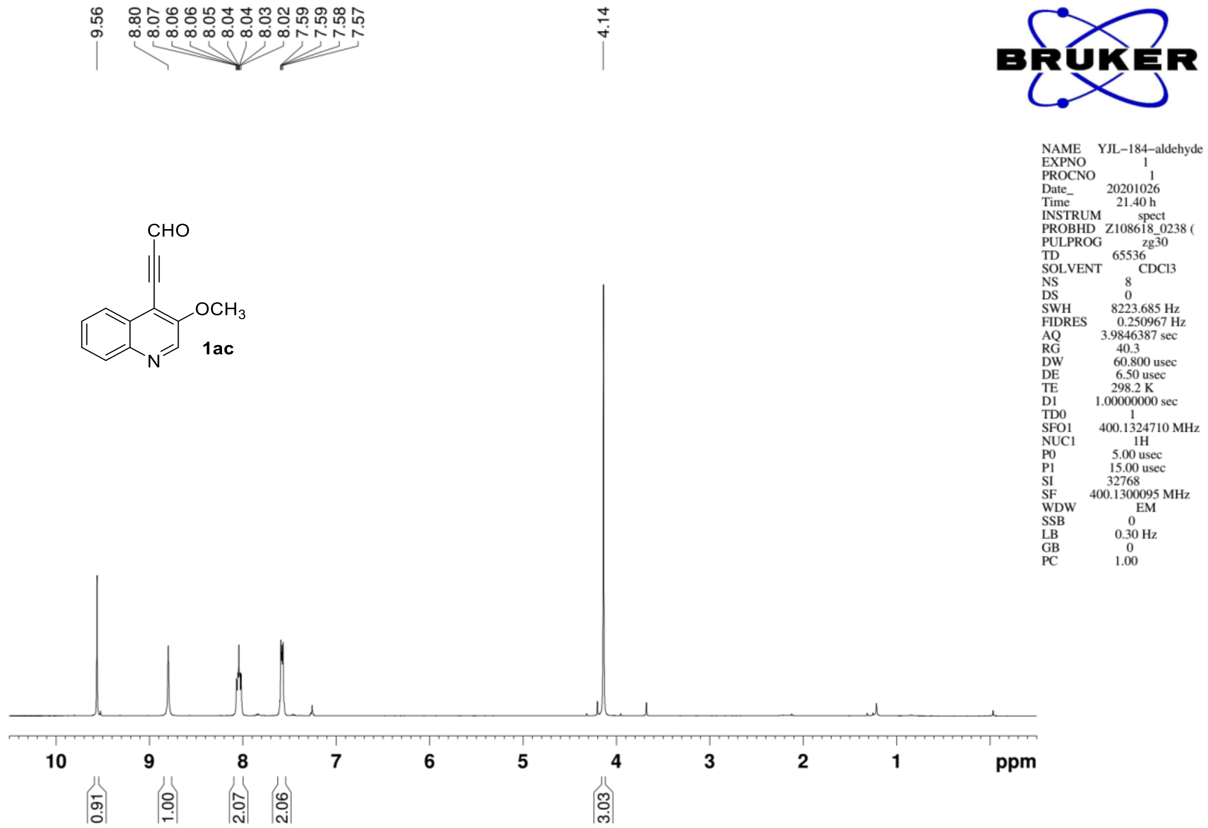

Supplementary Figure 27. ^{1}H NMR ($CDCl_3$, 400MHz) spectra of substrate **1ac**

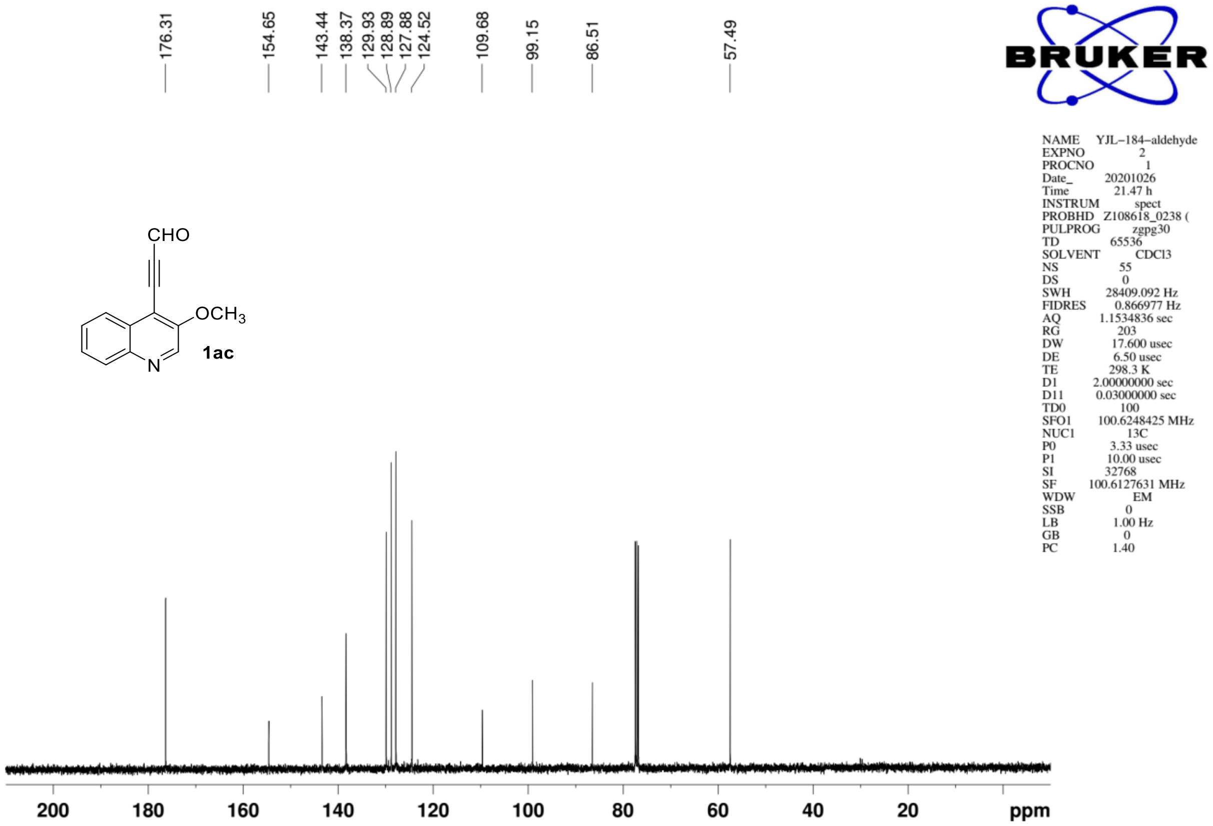

Supplementary Figure 28. ^{13}C NMR ($CDCl_3$, 101MHz) spectra of spectra of substrate **1ac**

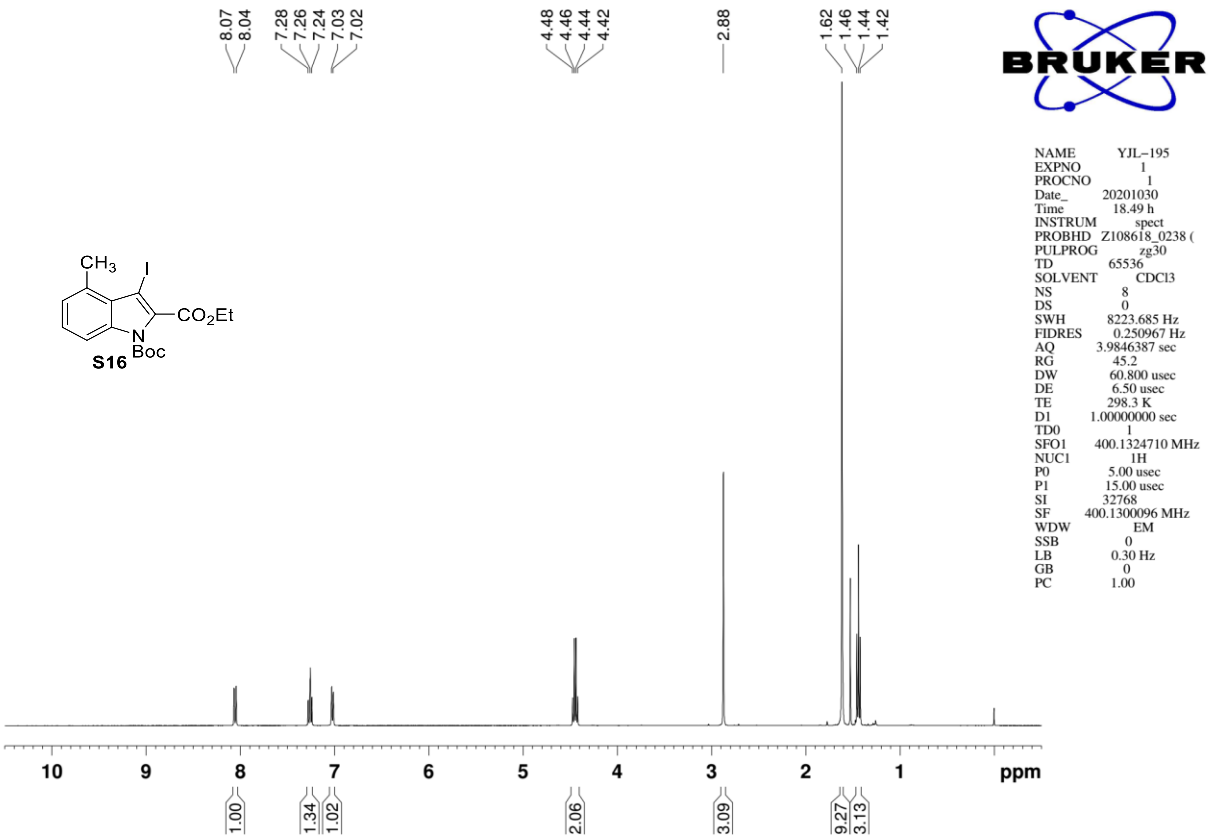

Supplementary Figure 29. ^{1}H NMR ($CDCl_3$, 400MHz) spectra of **S16**

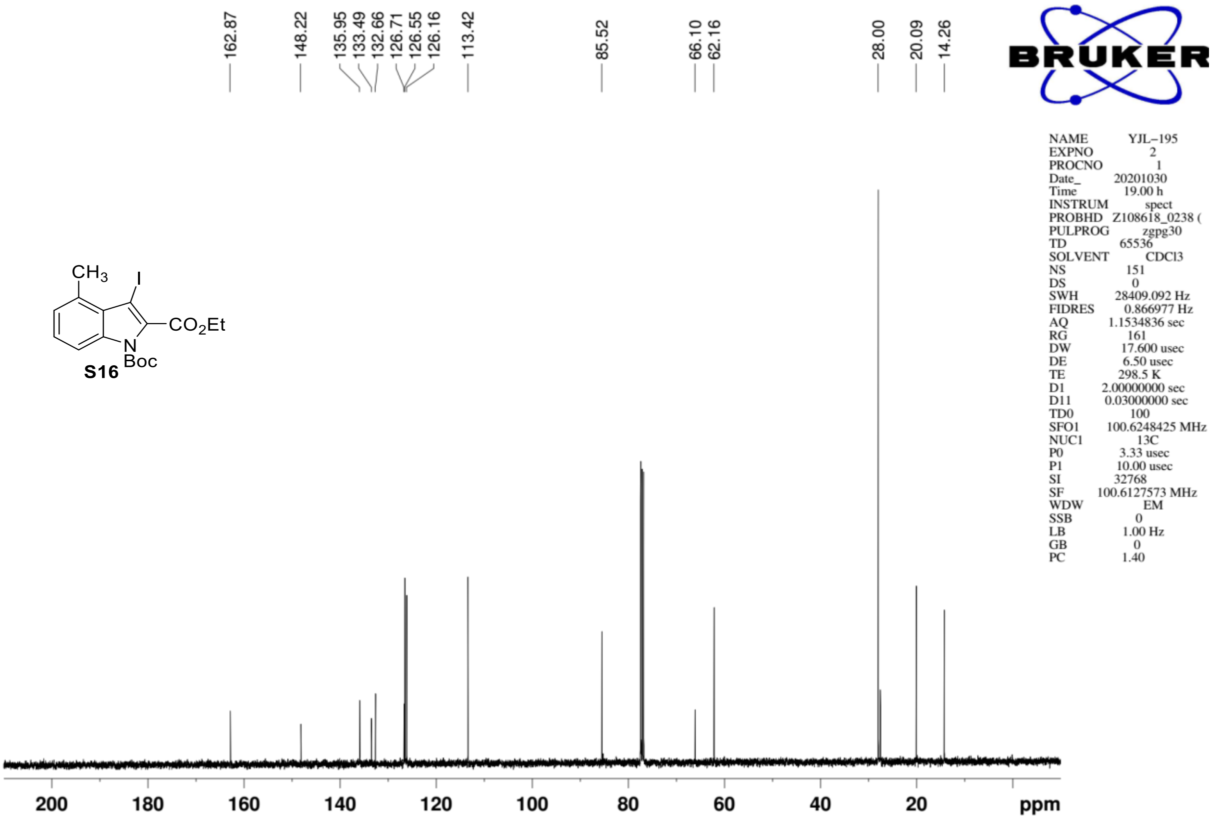

Supplementary Figure 30. ^{13}C NMR ($CDCl_3$, 101MHz) spectra of spectra of **S16**

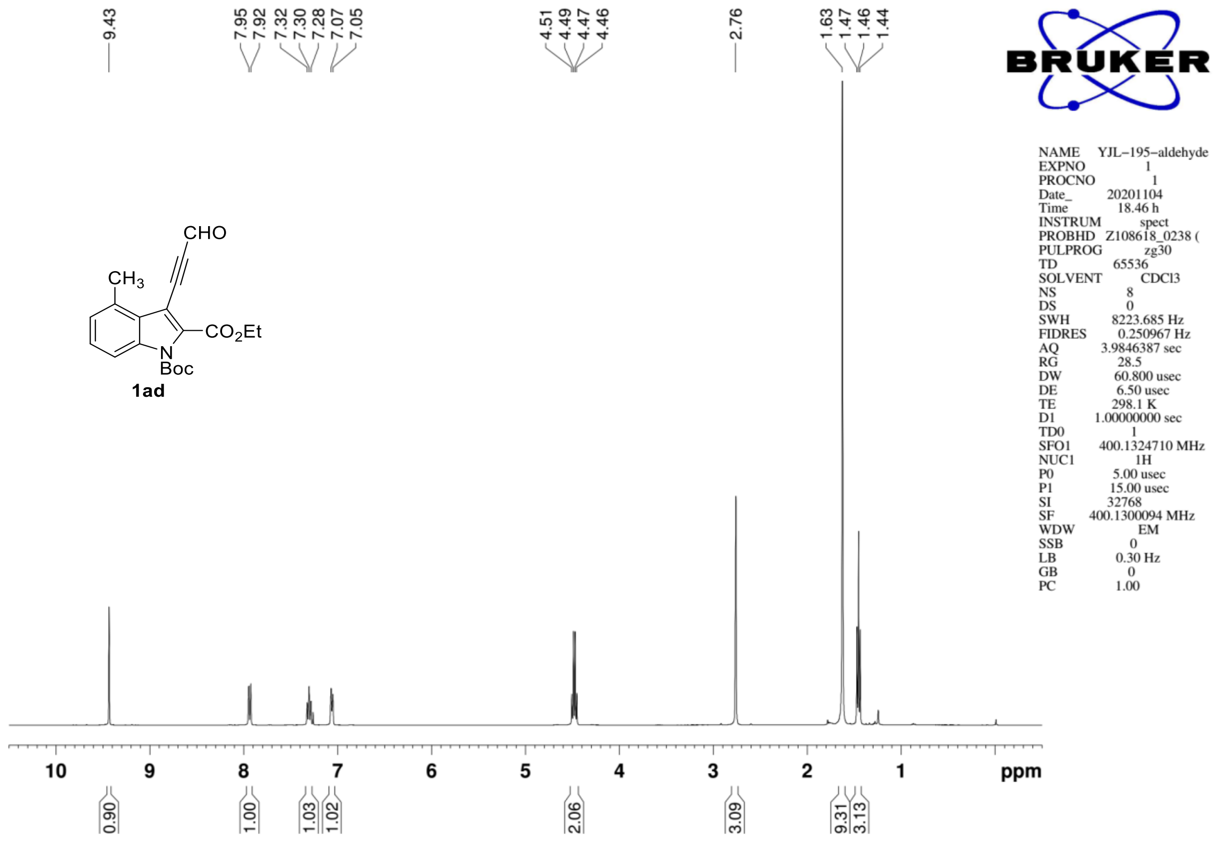

Supplementary Figure 31. ^{1}H NMR ($CDCl_3$, 400MHz) spectra of substrate **1ad**

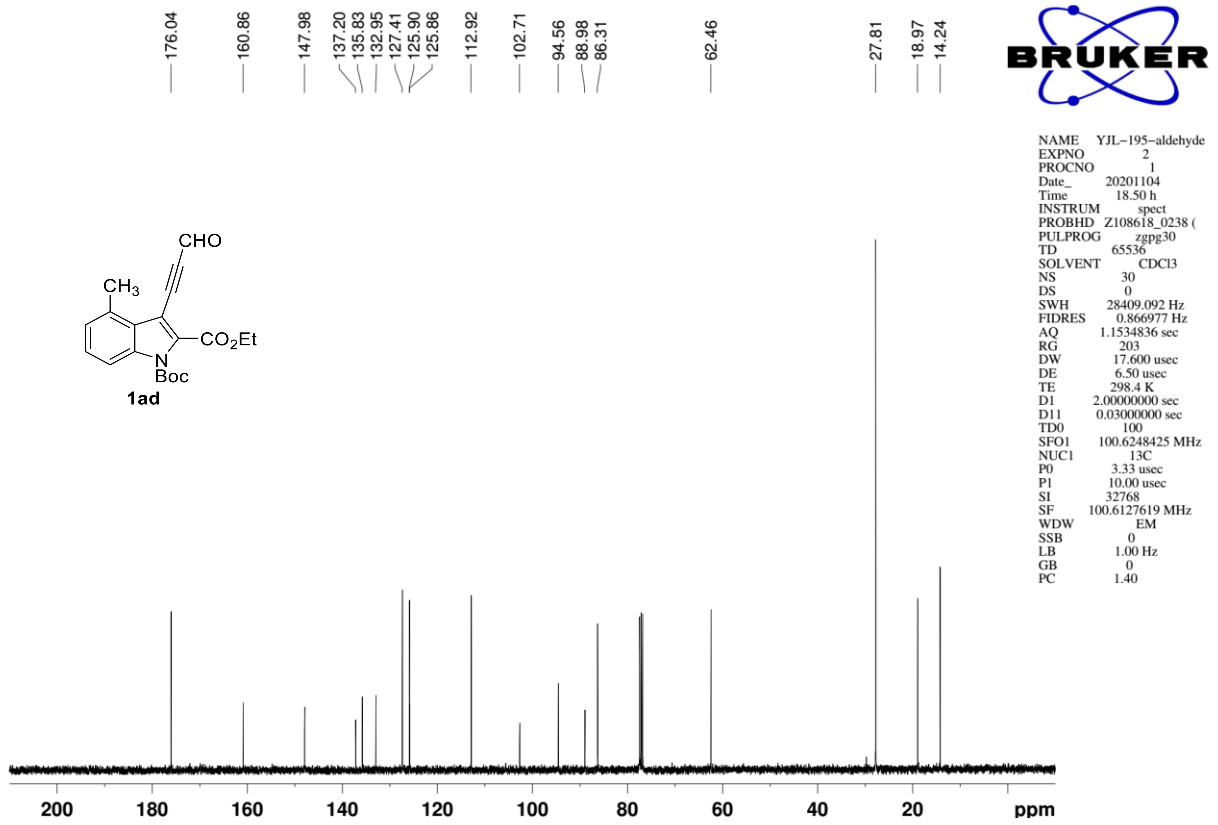

Supplementary Figure 32. ^{13}C NMR ($CDCl_3$, 101MHz) spectra of spectra of substrate **1ad**

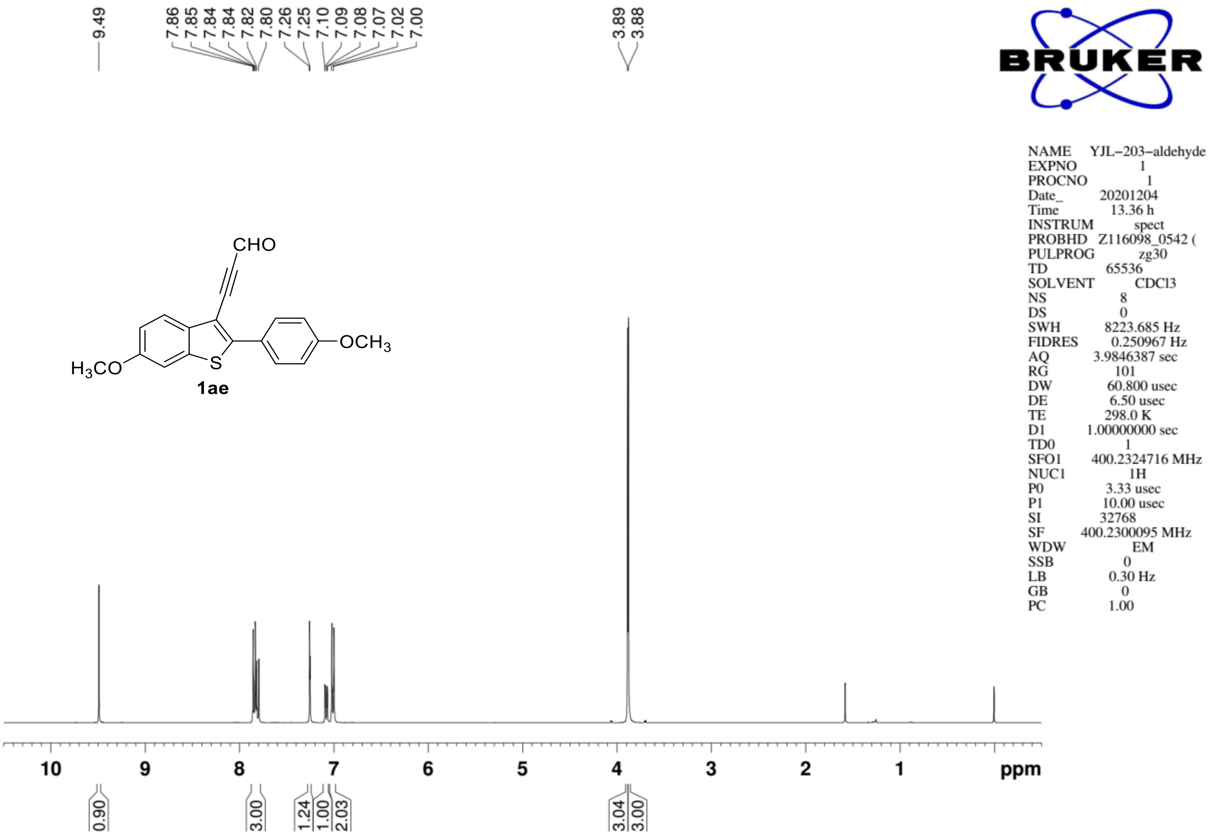

Supplementary Figure 33. ^{1}H NMR ($CDCl_3$, 400MHz) spectra of substrate **1ae**

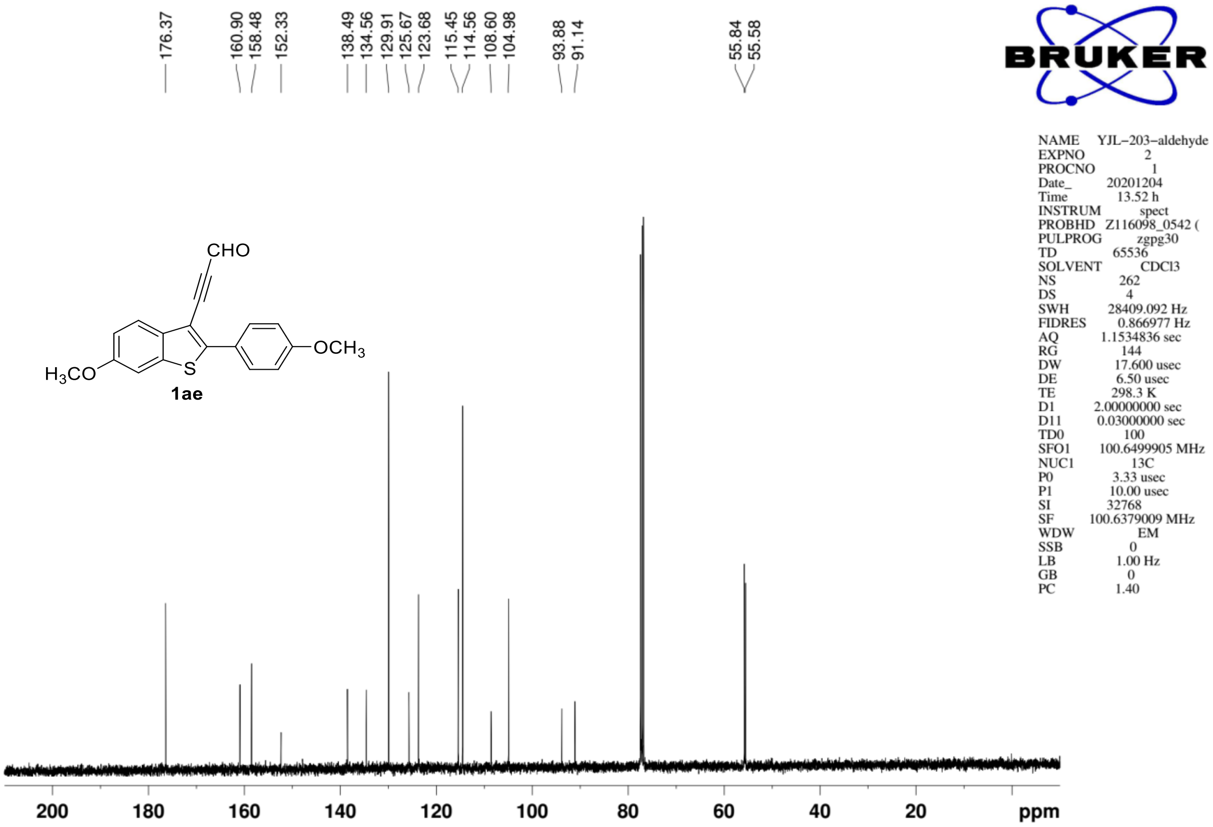

Supplementary Figure 34. ^{13}C NMR ($CDCl_3$, 101MHz) spectra of spectra of substrate **1ae**

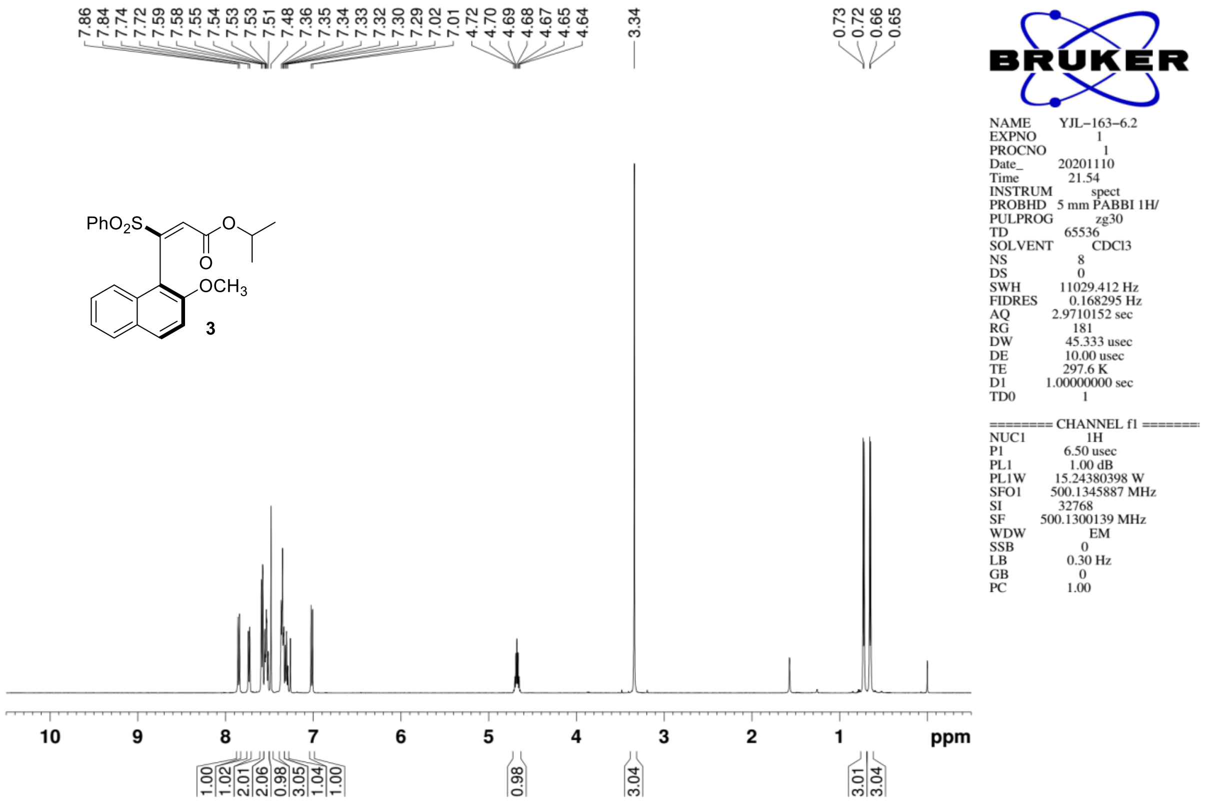

Supplementary Figure 35. ^{1}H NMR ($CDCl_3$, 500MHz) spectra of **3**

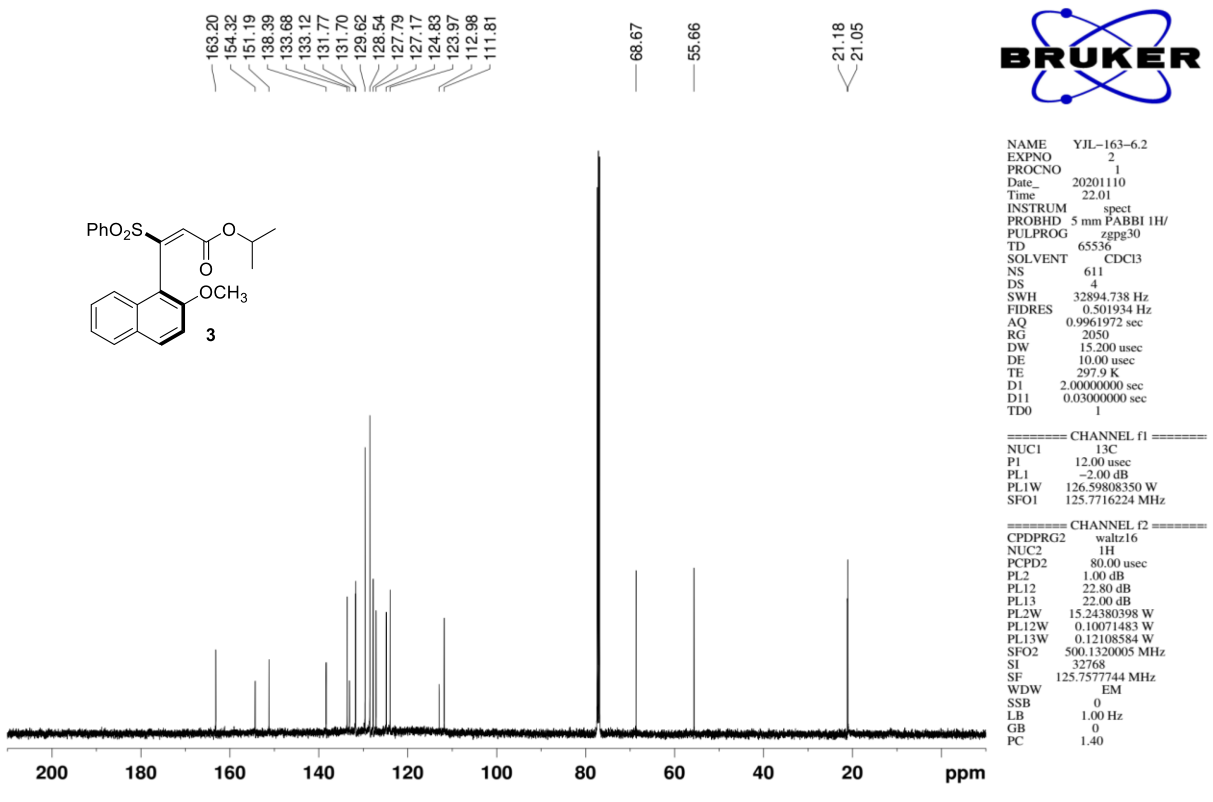

Supplementary Figure 36. ^{13}C NMR ($CDCl_3$, 126MHz) spectra of spectra of **3**

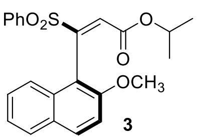

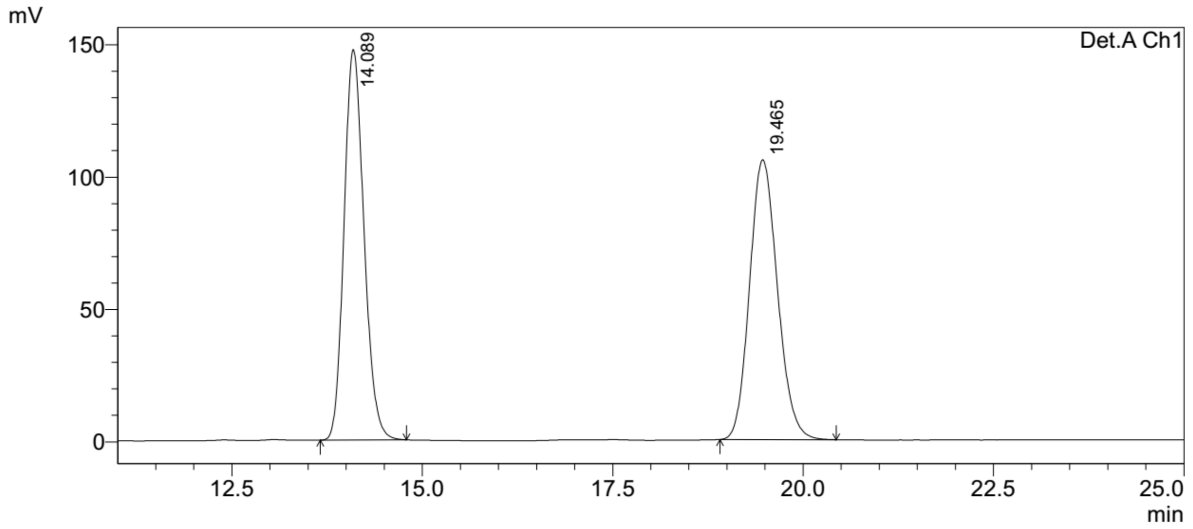

Detector A Ch1 254nm

Peak#	Ret. Time	Area	Height	Area %	Height %
1	14.089	2689432	147594	49.919	58.249
2	19.465	2698172	105791	50.081	41.751
Total		5387604	253385	100.000	100.000

Axially chiral styrene **3** (prepared by NHC catalytic reaction)

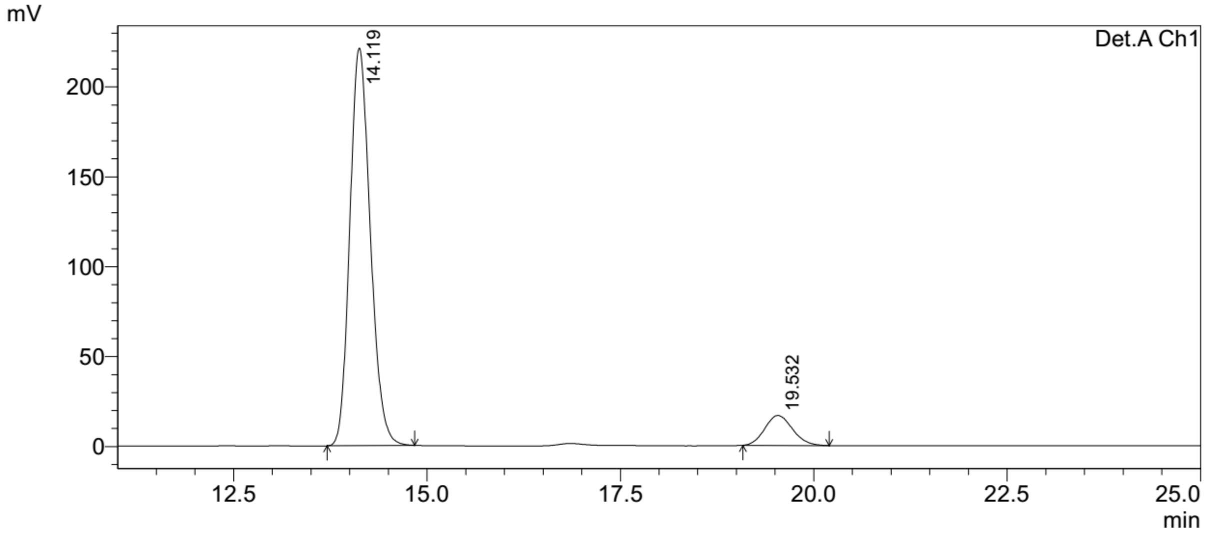

Detector A Ch1 254nm

Peak#	Ret. Time	Area	Height	Area %	Height %
1	14.119	4081531	221160	90.768	93.004
2	19.532	415116	16635	9.232	6.996
Total		4496647	237795	100.000	100.000

Supplementary Figure 37. HPLC spectra of **3** (prepared by NHC catalytic reaction)

Axially chiral styrene **3** (prepared by transesterification)

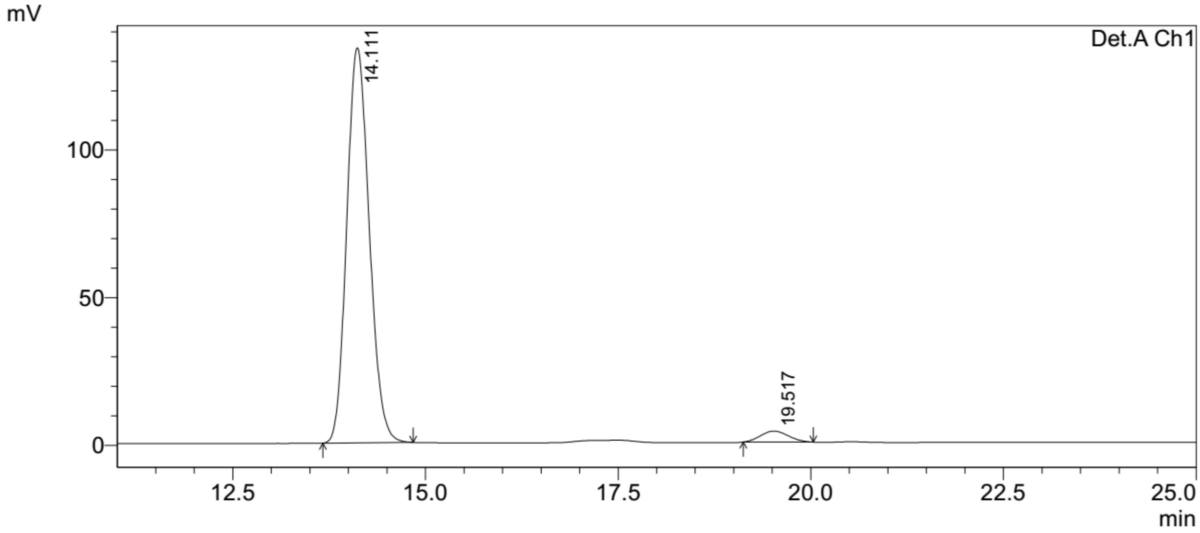

Detector A Ch1 254nm

Peak#	Ret. Time	Area	Height	Area %	Height %
1	14.111	2715857	133719	96.702	97.303
2	19.517	92635	3707	3.298	2.697
Total		2808492	137425	100.000	100.000

Supplementary Figure 38. HPLC spectra of **3** (prepared by transesterification)

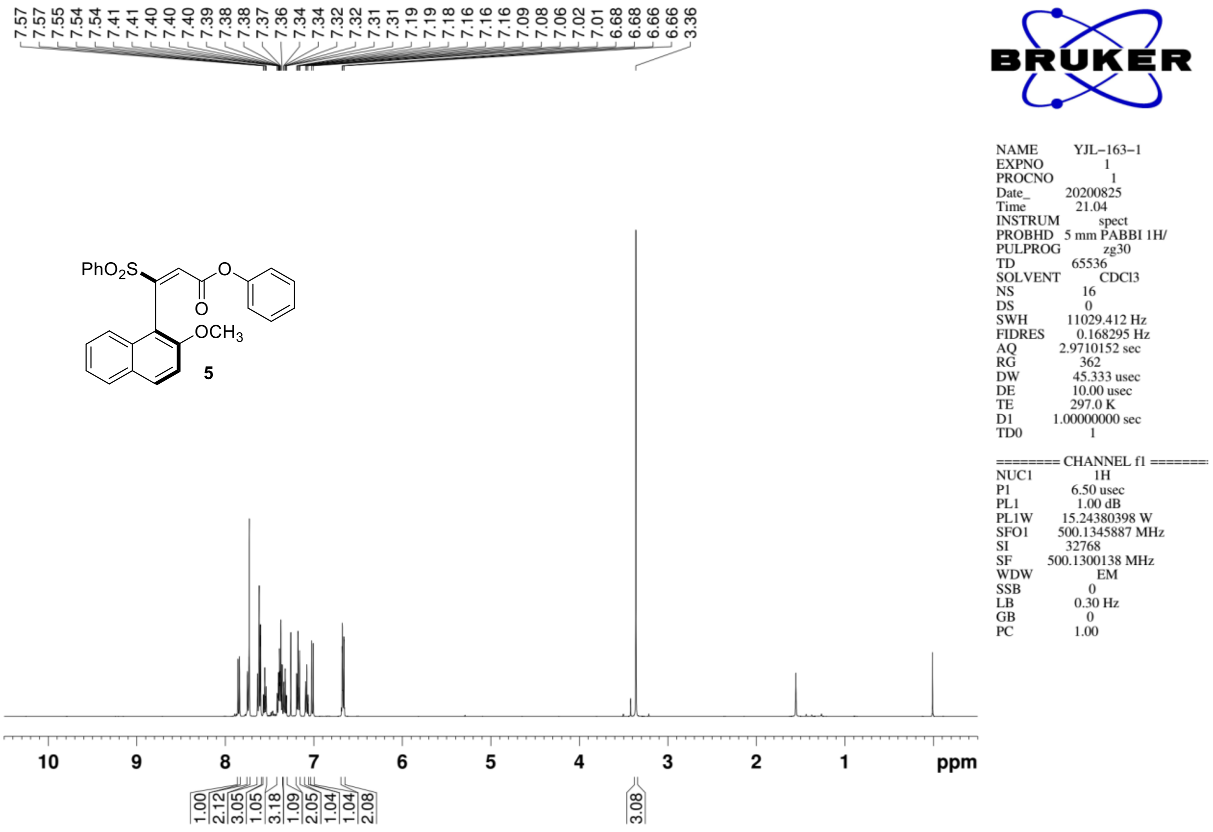

Supplementary Figure 39. ^{1}H NMR ($CDCl_3$, 500MHz) spectra of **5**

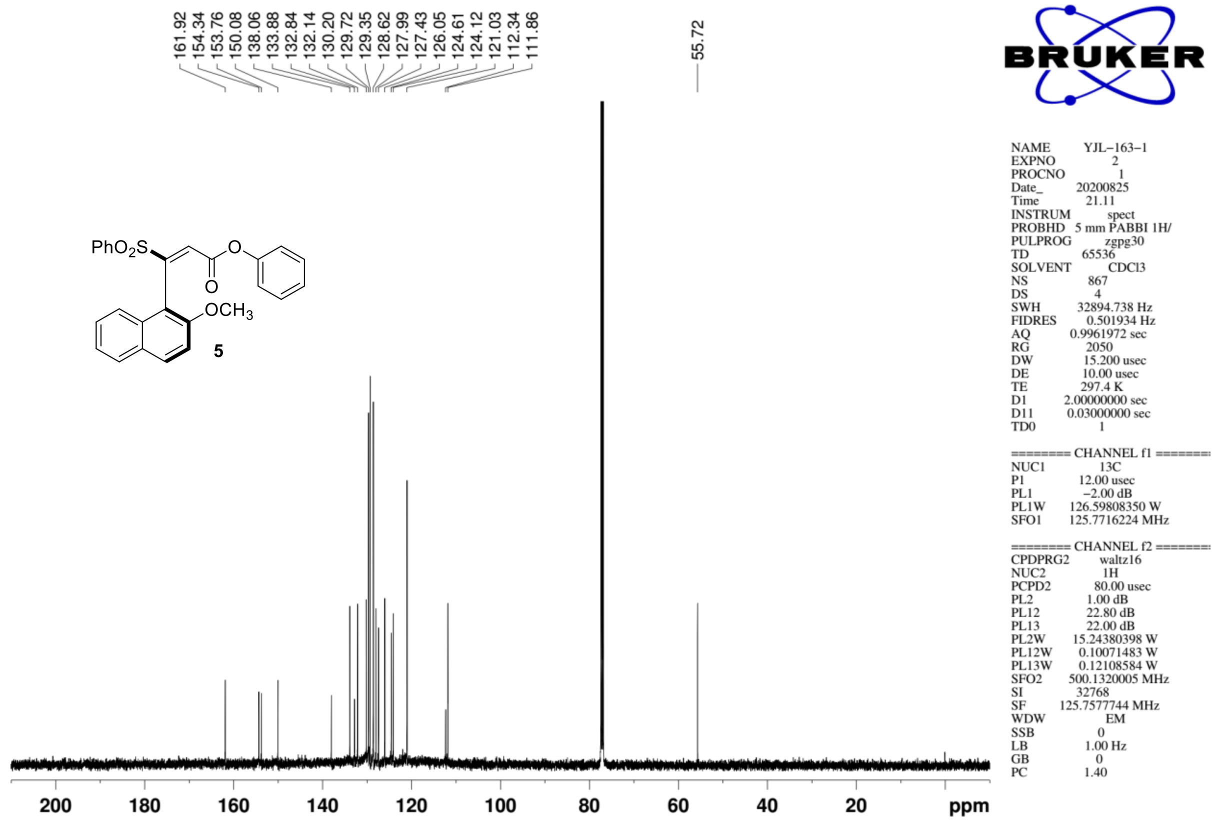

Supplementary Figure 40. ^{13}C NMR ($CDCl_3$, 126MHz) spectra of spectra of **5**

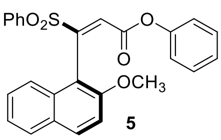

5

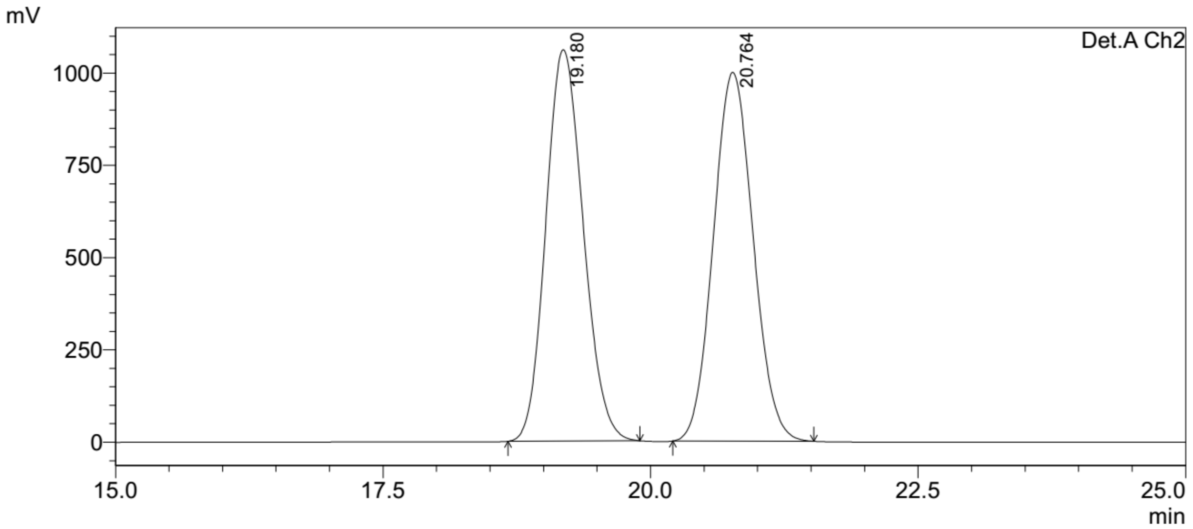

Detector A Ch2 220nm

Peak#	Ret. Time	Area	Height	Area %	Height %
1	19.180	25791628	1060143	49.979	51.482
2	20.764	25812883	999118	50.021	48.518
Total		51604511	2059261	100.000	100.000

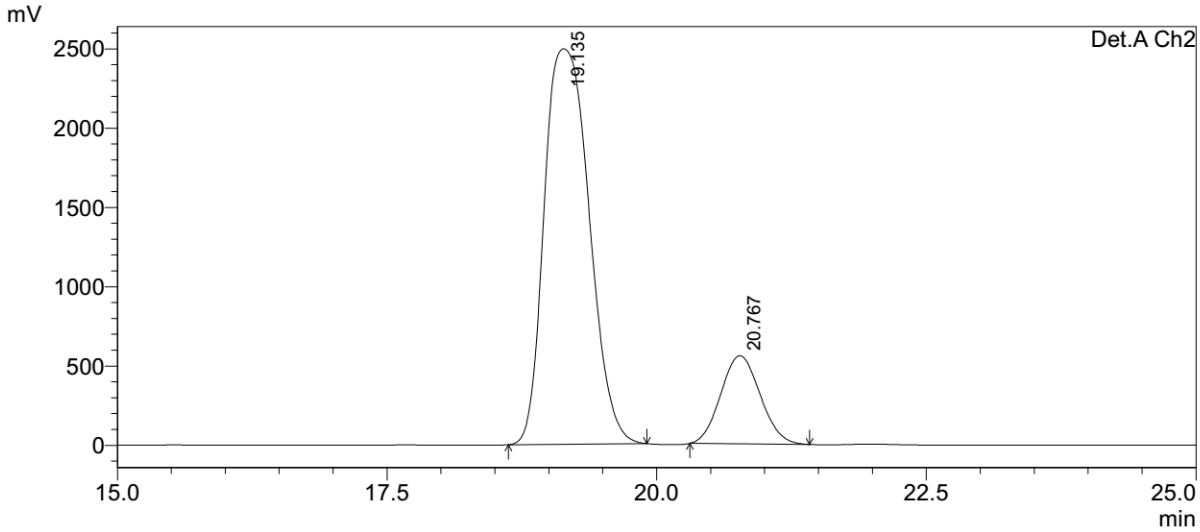

Detector A Ch2 220nm

Peak#	Ret. Time	Area	Height	Area %	Height %
1	19.135	72261084	2493968	83.645	81.774
2	20.767	14128824	555846	16.355	18.226
Total		86389908	3049814	100.000	100.000

Supplementary Figure 41. HPLC spectra of **5**

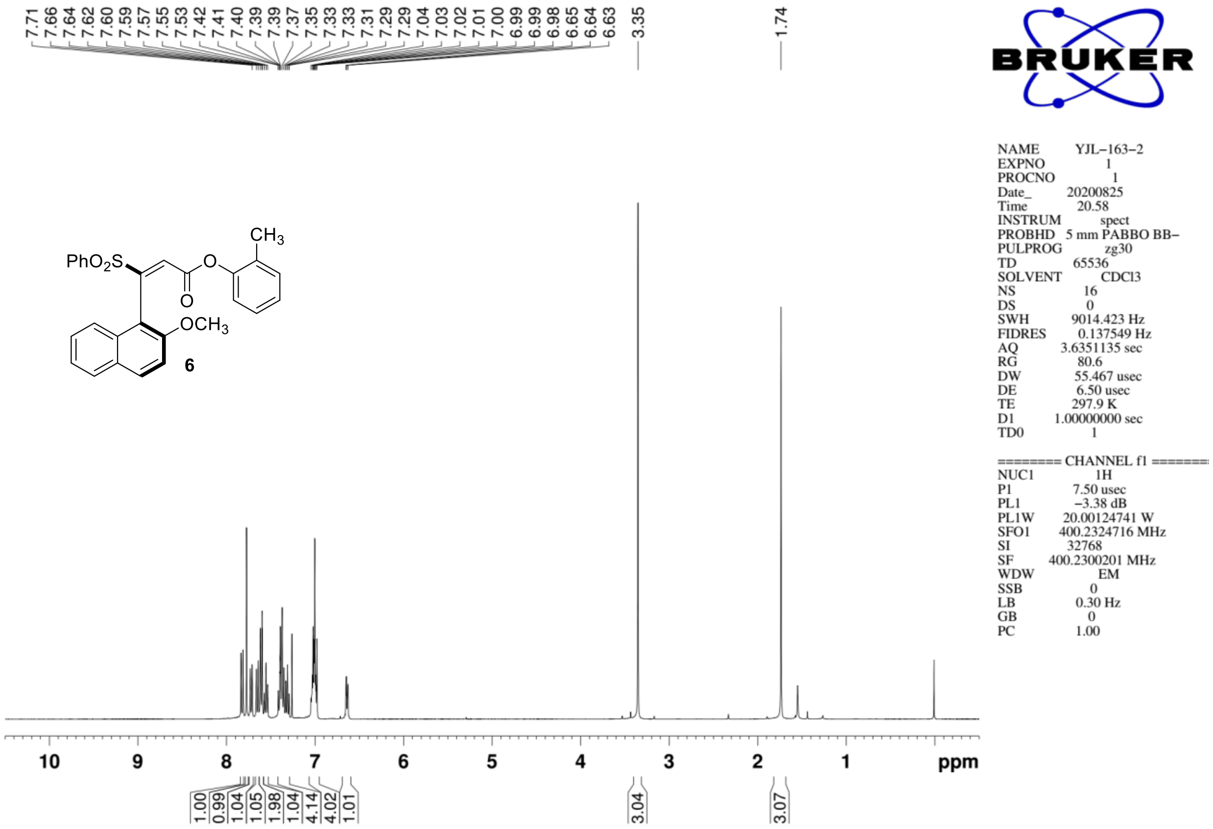

Supplementary Figure 42. ^{1}H NMR ($CDCl_3$, 400MHz) spectra of **6**

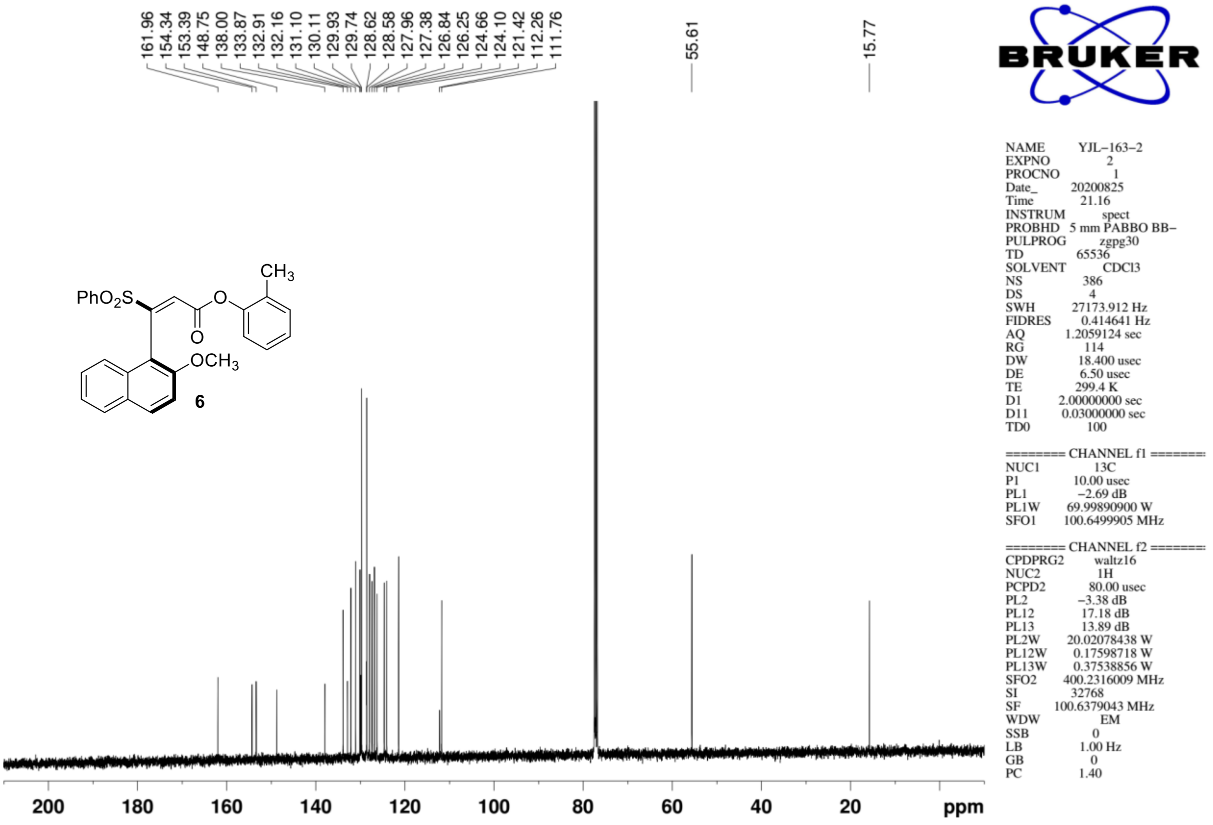

Supplementary Figure 43. ^{13}C NMR ($CDCl_3$, 101MHz) spectra of spectra of **6**

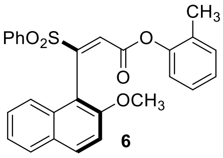

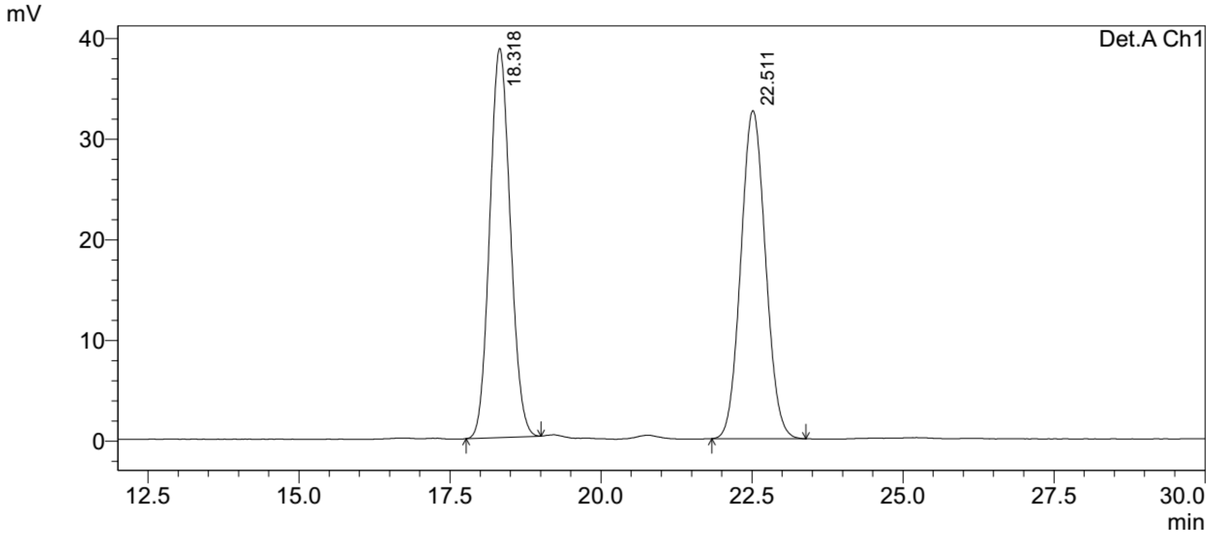

Detector A Ch1 254nm

Peak#	Ret. Time	Area	Height	Area %	Height %
1	18.318	918189	38673	49.755	54.258
2	22.511	927249	32603	50.245	45.742
Total		1845438	71276	100.000	100.000

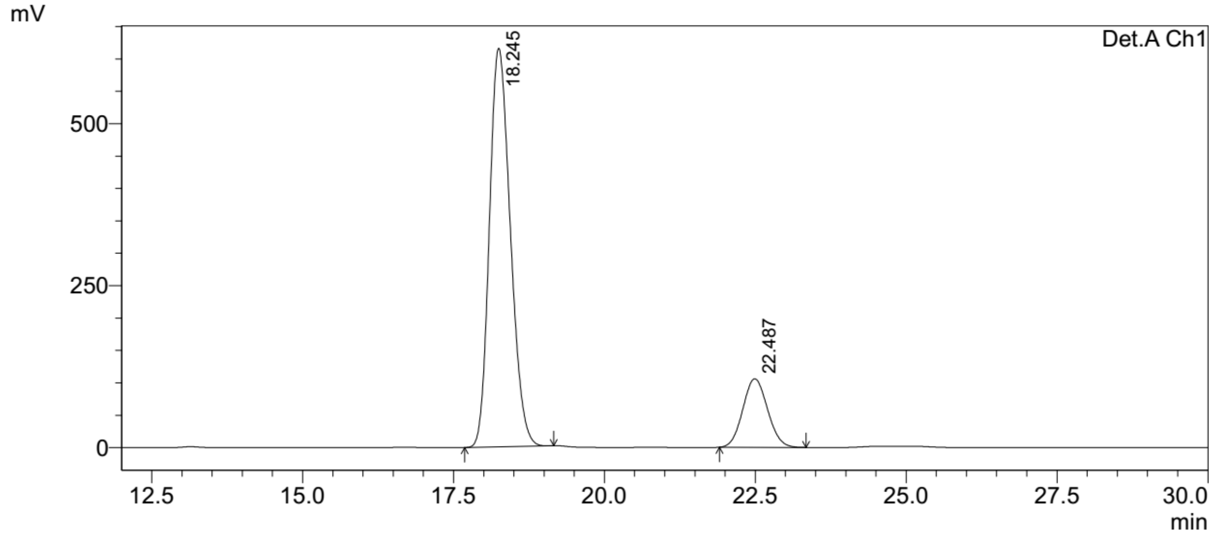

Detector A Ch1 254nm

Peak#	Ret. Time	Area	Height	Area %	Height %
1	18.245	14835275	614995	83.086	85.318
2	22.487	3019960	105835	16.914	14.682
Total		17855235	720830	100.000	100.000

Supplementary Figure 44. HPLC spectra of **6**

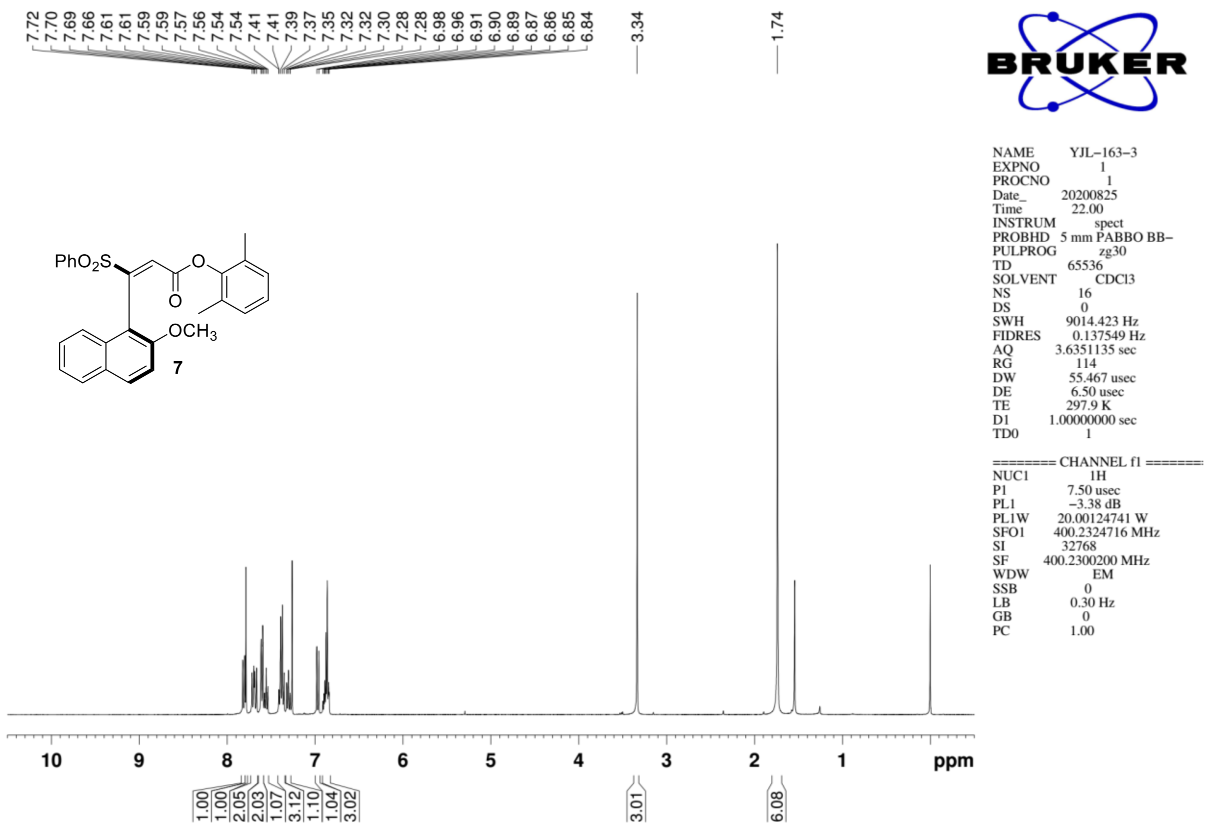

Supplementary Figure 45. ^{1}H NMR ($CDCl_3$, 400MHz) spectra of **7**

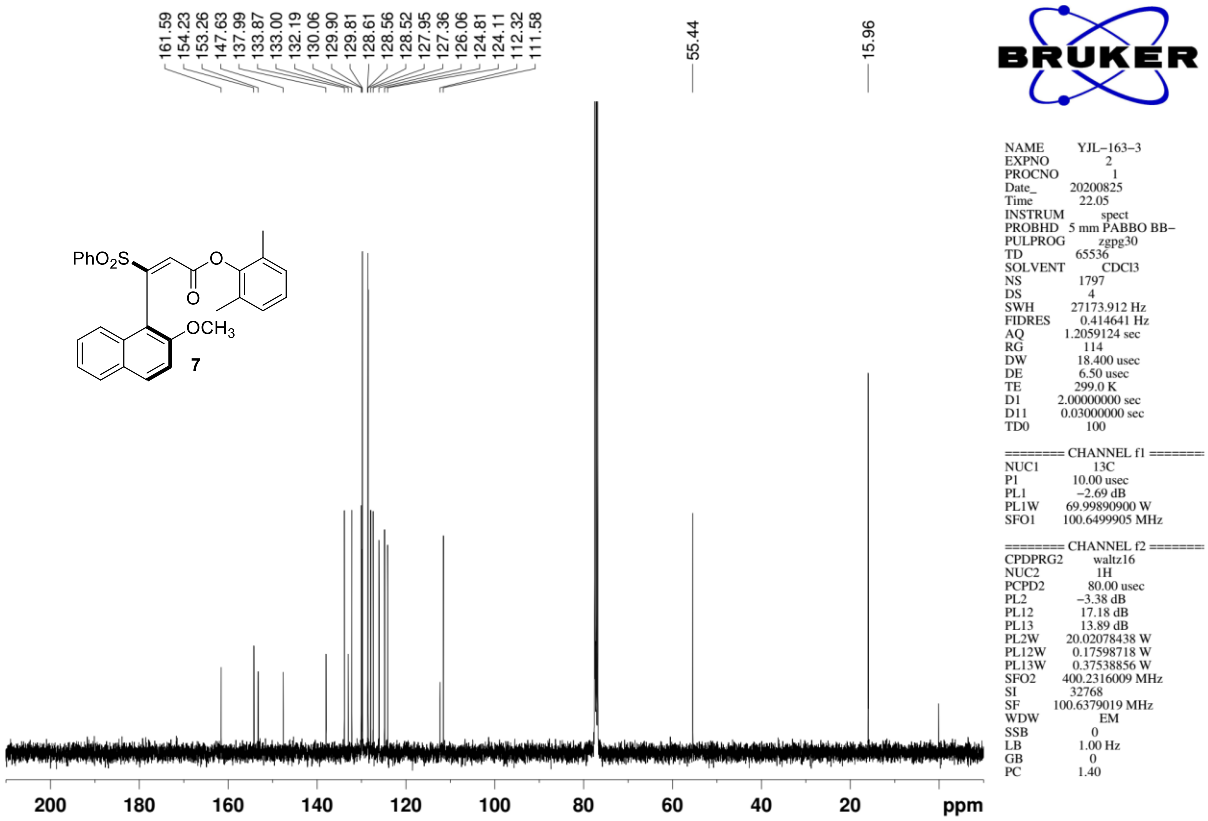

Supplementary Figure 46. ^{13}C NMR ($CDCl_3$, 101MHz) spectra of spectra of **7**

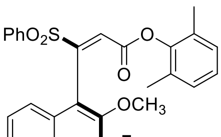

7

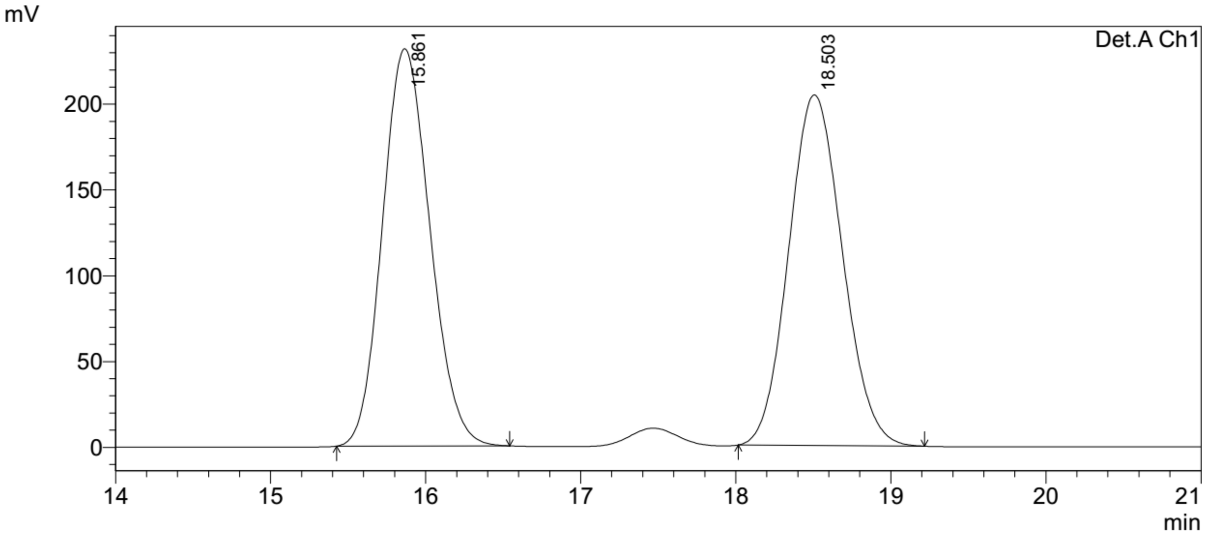

Detector A Ch1 254nm

Peak#	Ret. Time	Area	Height	Area %	Height %
1	15.861	4930148	231548	50.019	53.110
2	18.503	4926375	204429	49.981	46.890
Total		9856523	435976	100.000	100.000

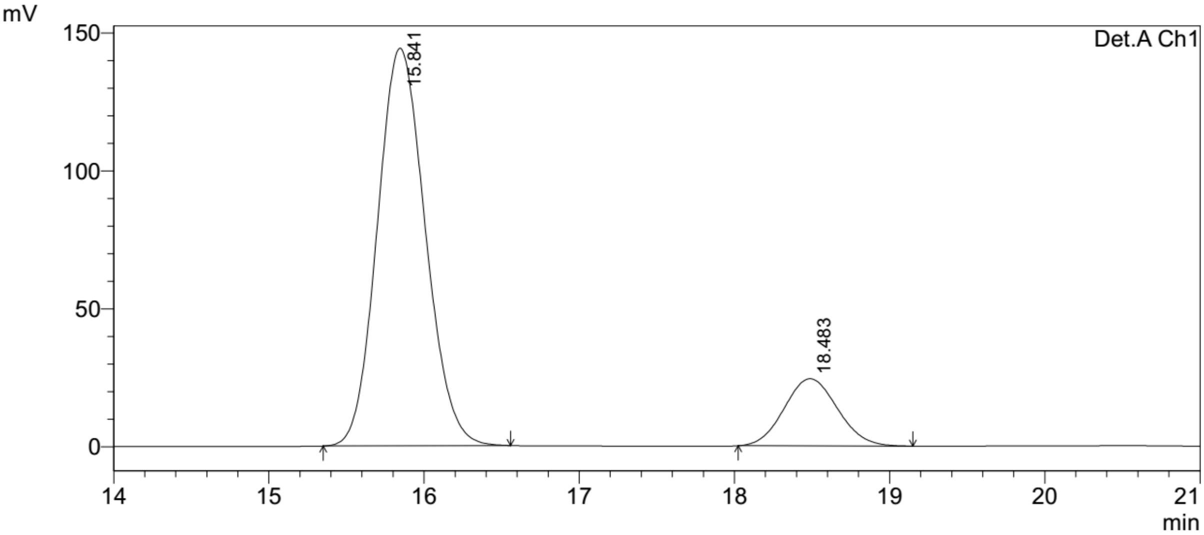

Detector A Ch1 254nm

Peak#	Ret. Time	Area	Height	Area %	Height %
1	15.841	3094618	144105	84.155	85.530
2	18.483	582665	24380	15.845	14.470
Total		3677283	168485	100.000	100.000

Supplementary Figure 47. HPLC spectra of **7**

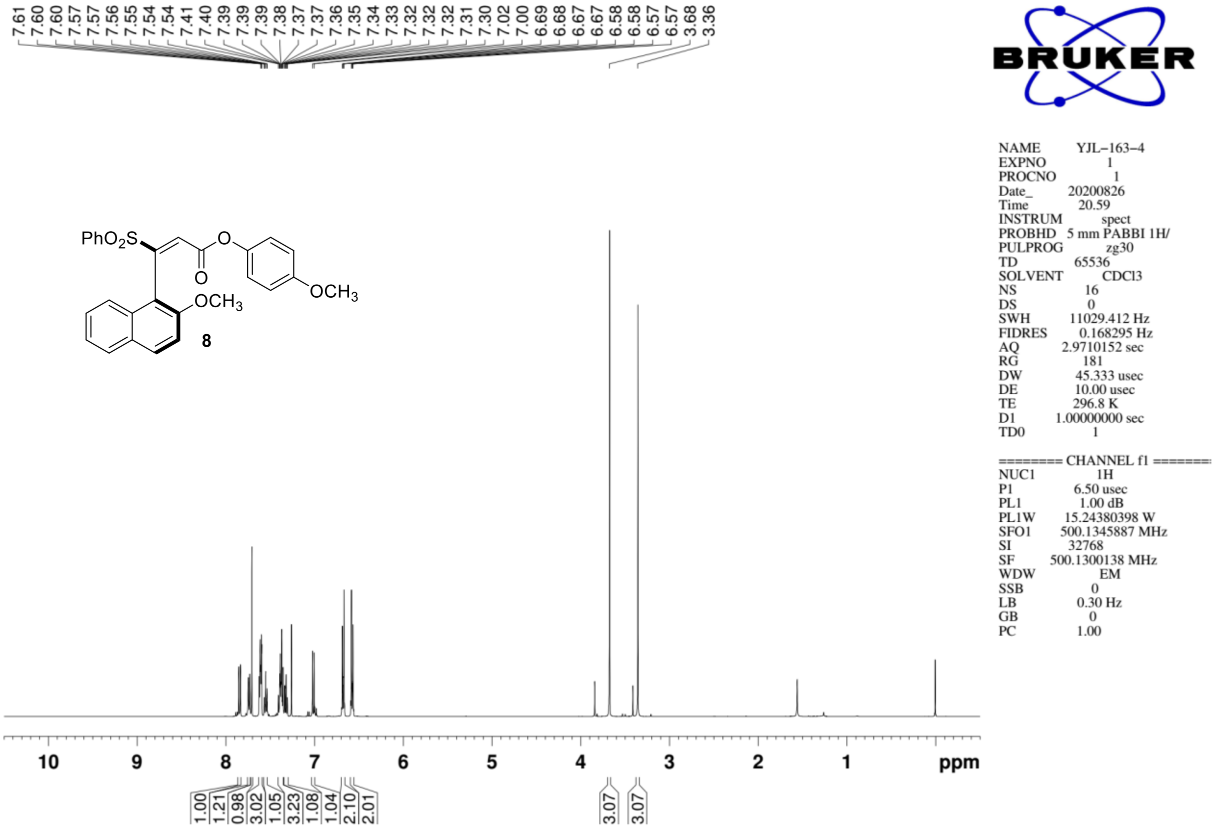

Supplementary Figure 48. ^{1}H NMR ($CDCl_3$, 500MHz) spectra of **8**

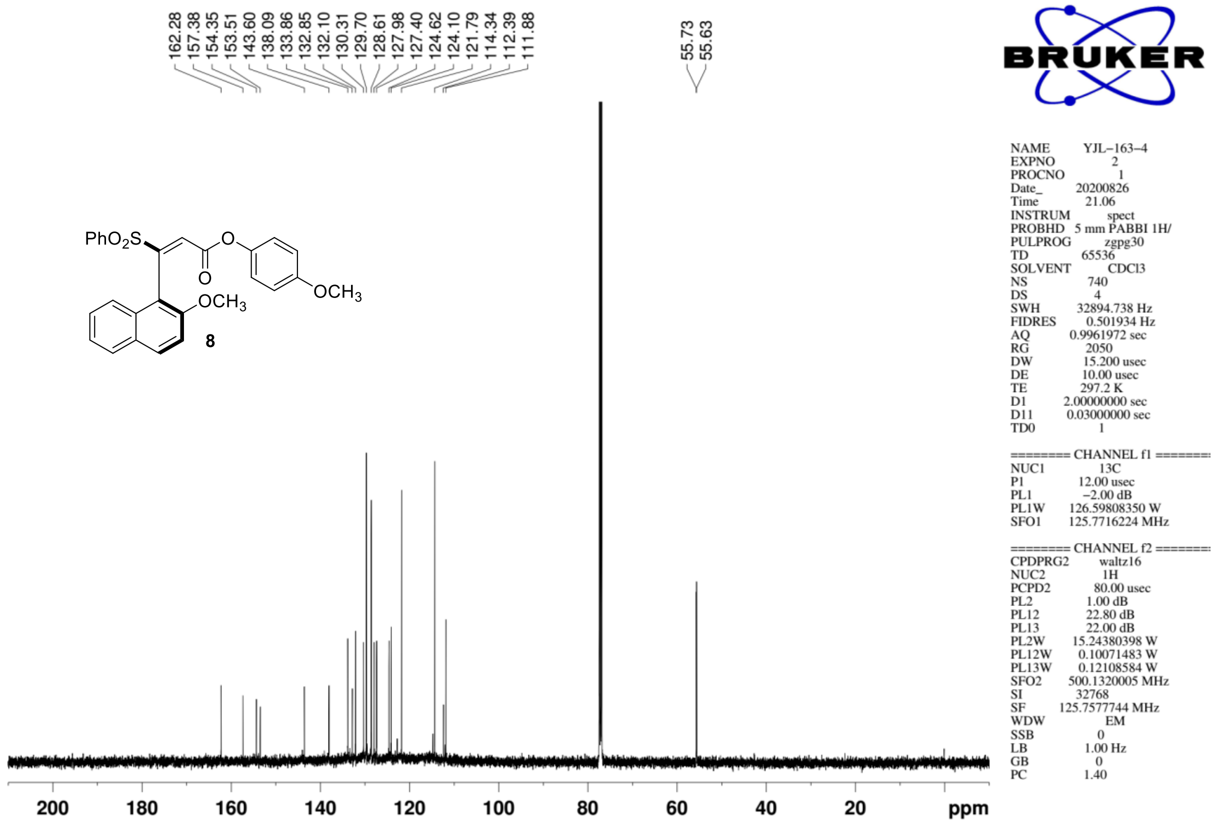

Supplementary Figure 49. ^{13}C NMR ($CDCl_3$, 126MHz) spectra of spectra of **8**

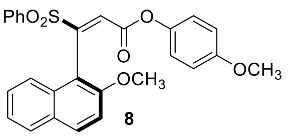

8

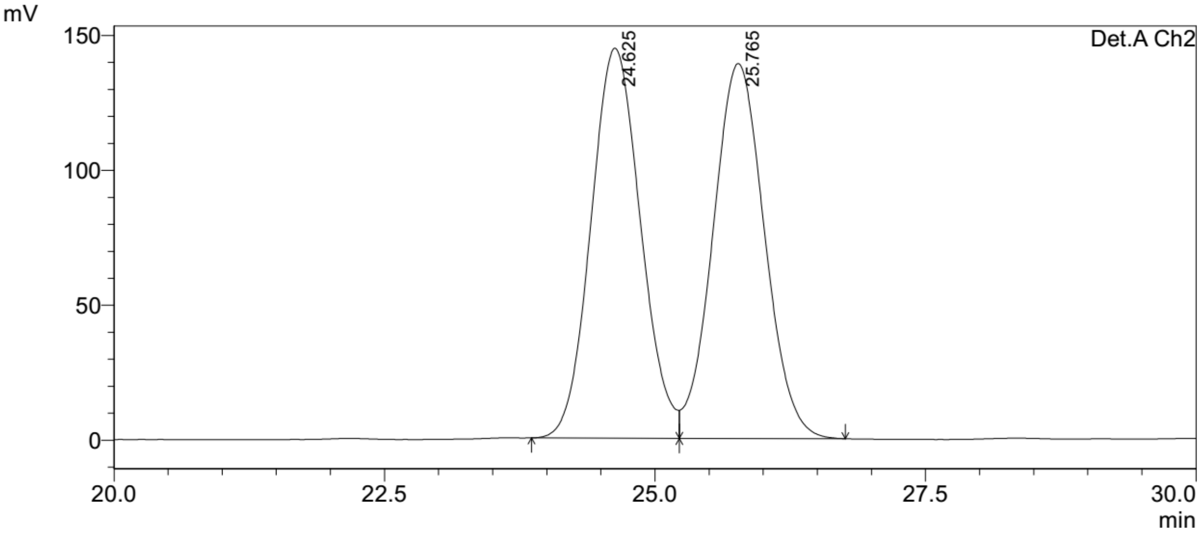

Detector A Ch2 220nm

Peak#	Ret. Time	Area	Height	Area %	Height %
1	24.625	4612479	144467	50.029	50.972
2	25.765	4607176	138955	49.971	49.028
Total		9219655	283422	100.000	100.000

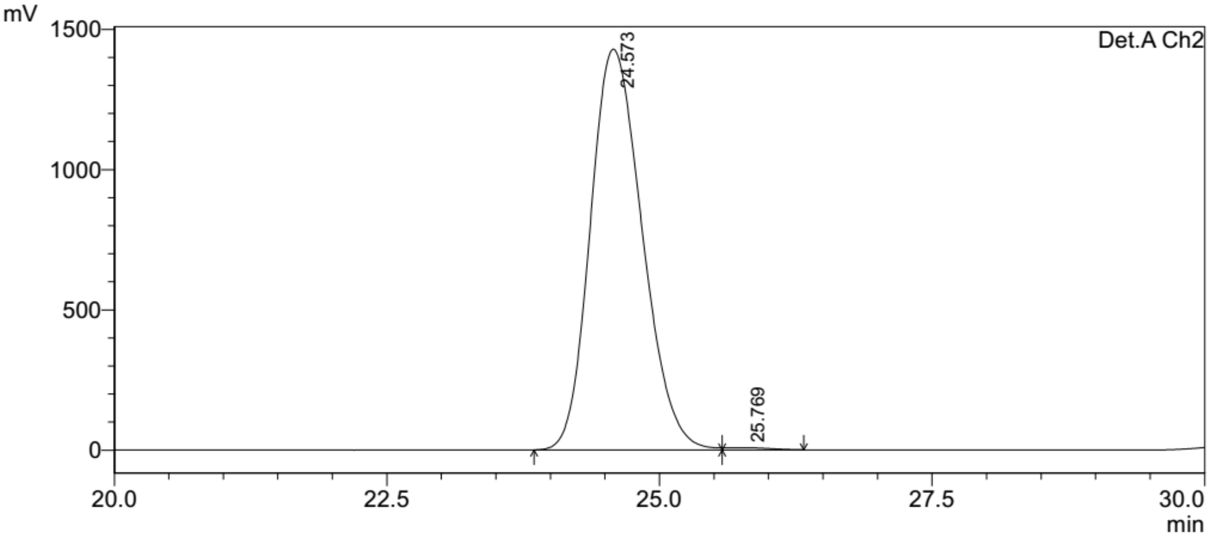

Detector A Ch2 220nm

Peak#	Ret. Time	Area	Height	Area %	Height %
1	24.573	47287430	1428401	99.547	99.452
2	25.769	214956	7865	0.453	0.548
Total		47502386	1436266	100.000	100.000

Supplementary Figure 50. HPLC spectra of **8**

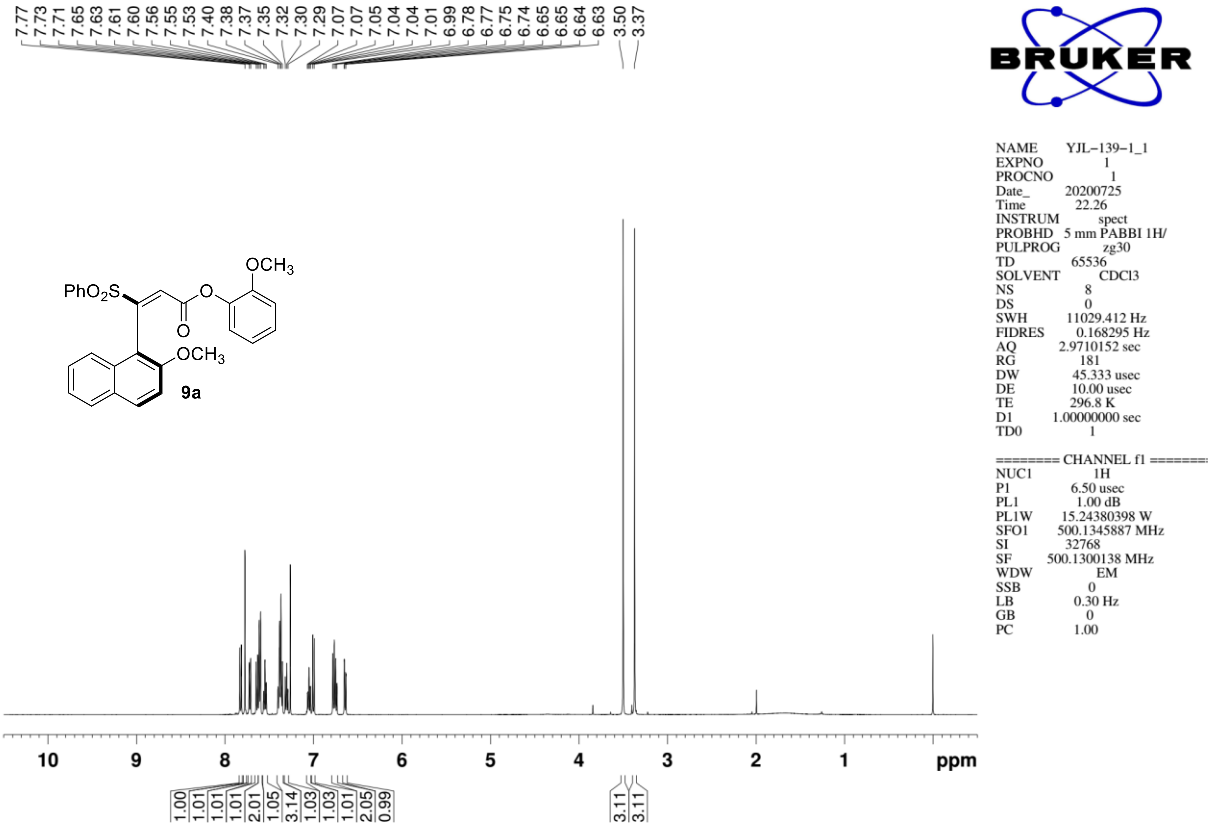

Supplementary Figure 51. ^{1}H NMR ($CDCl_3$, 500MHz) spectra of **9a**

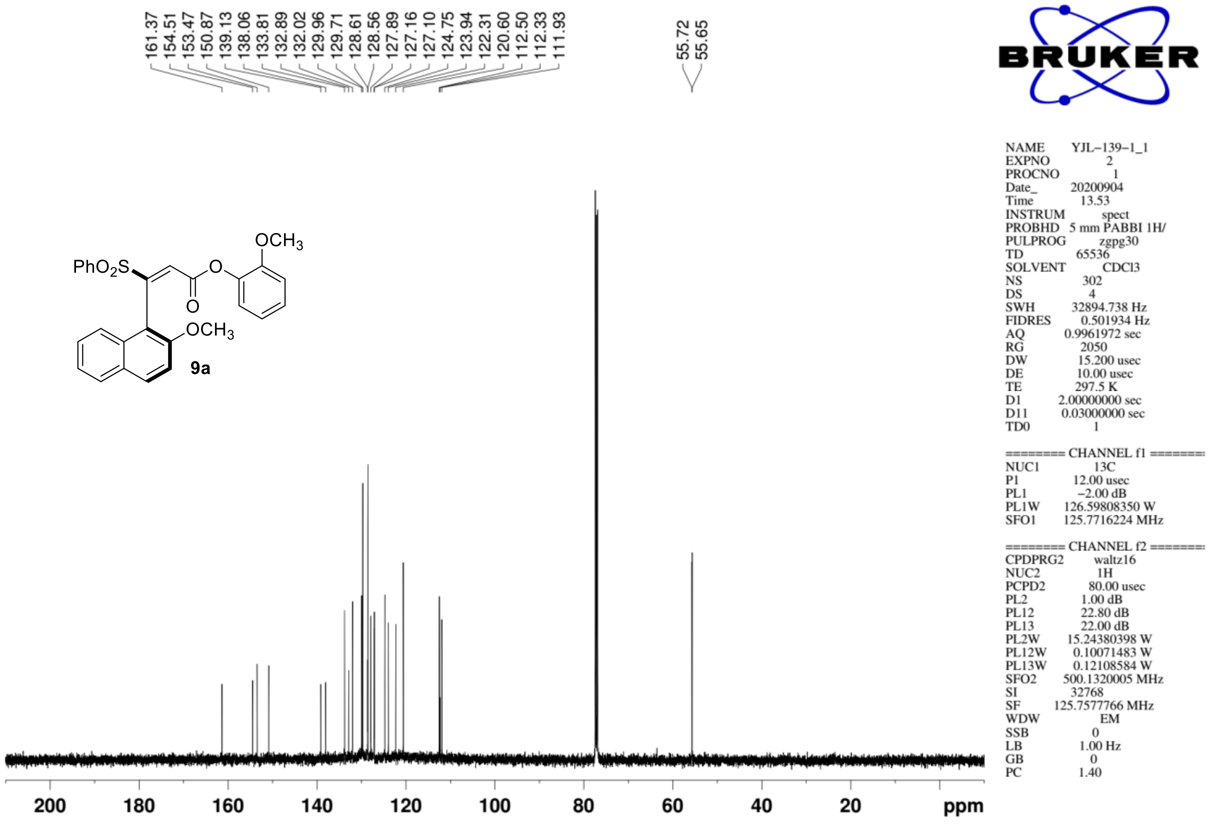

Supplementary Figure 52. ^{13}C NMR ($CDCl_3$, 126MHz) spectra of spectra of **9a**

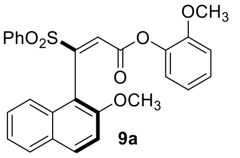

9a

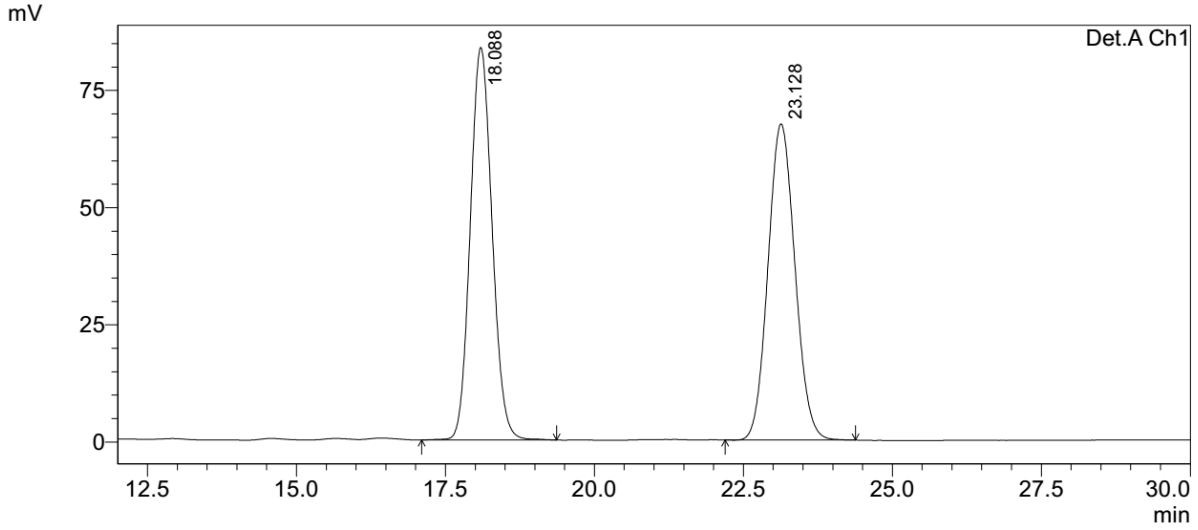

Detector A Ch1 254nm

Peak#	Ret. Time	Area	Height	Area %	Height %
1	18.088	2133280	83768	50.134	55.390
2	23.128	2121907	67466	49.866	44.610
Total		4255186	151235	100.000	100.000

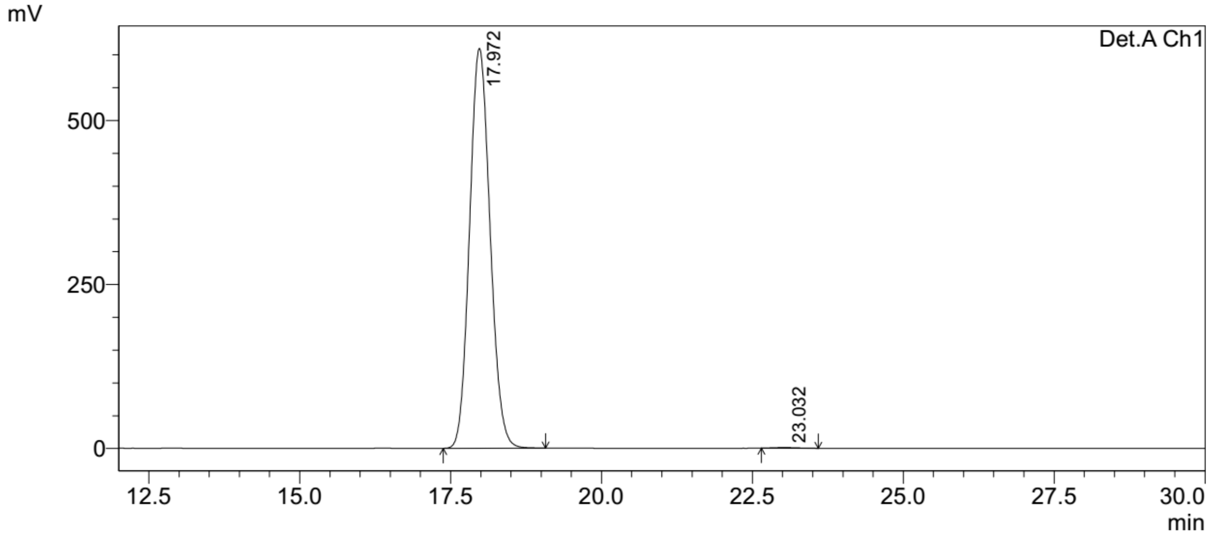

Detector A Ch1 254nm

Peak#	Ret. Time	Area	Height	Area %	Height %
1	17.972	14439648	609672	99.744	99.808
2	23.032	37112	1175	0.256	0.192
Total		14476760	610848	100.000	100.000

Supplementary Figure 53. HPLC spectra of **9a**

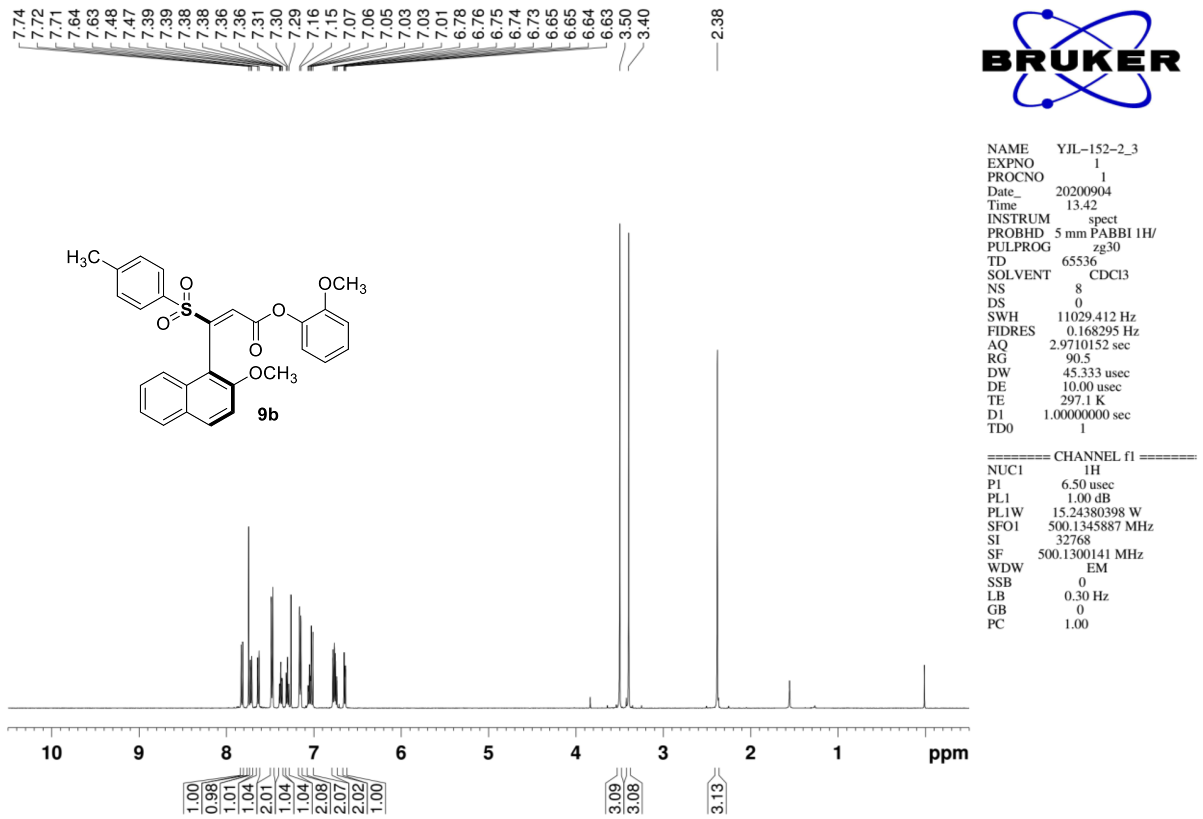

Supplementary Figure 54. ^{1}H NMR ($CDCl_3$, 500MHz) spectra of **9b**

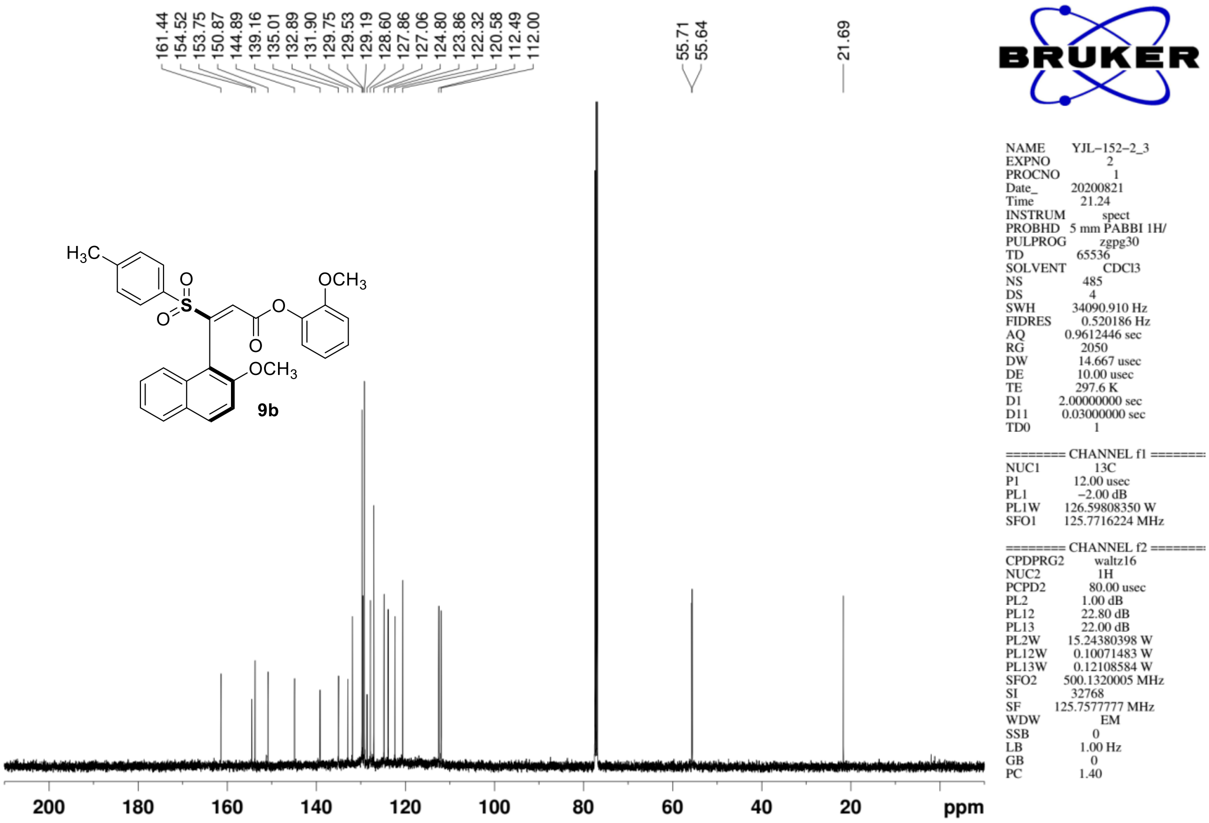

Supplementary Figure 55. ^{13}C NMR ($CDCl_3$, 126MHz) spectra of spectra of **9b**

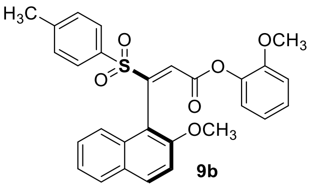

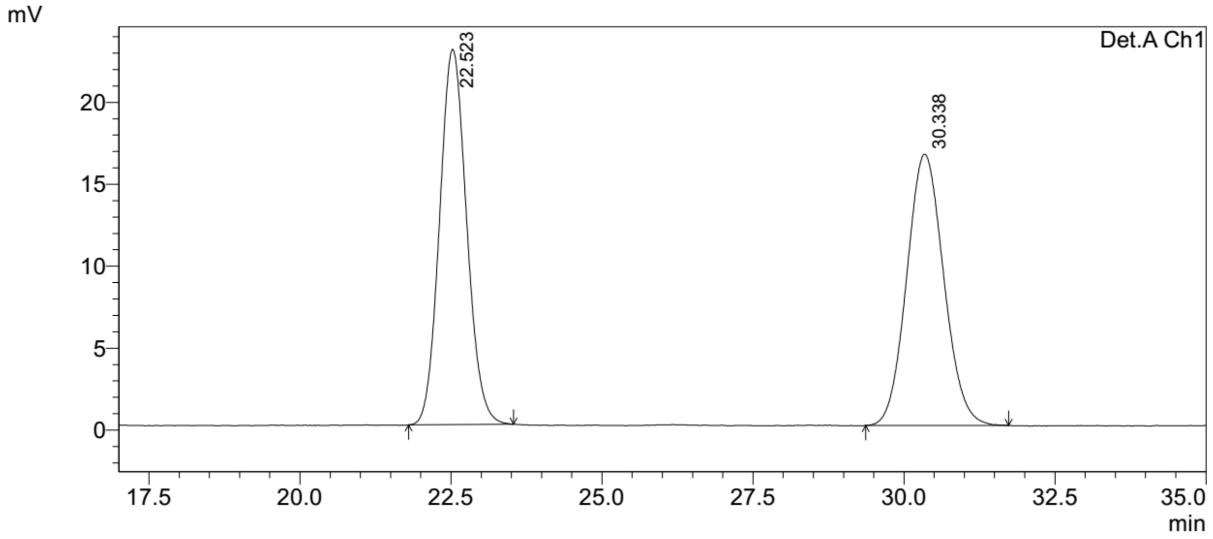

Detector A Ch1 254nm

Peak#	Ret. Time	Area	Height	Area %	Height %
1	22.523	714394	22913	50.281	58.045
2	30.338	706405	16561	49.719	41.955
Total		1420799	39474	100.000	100.000

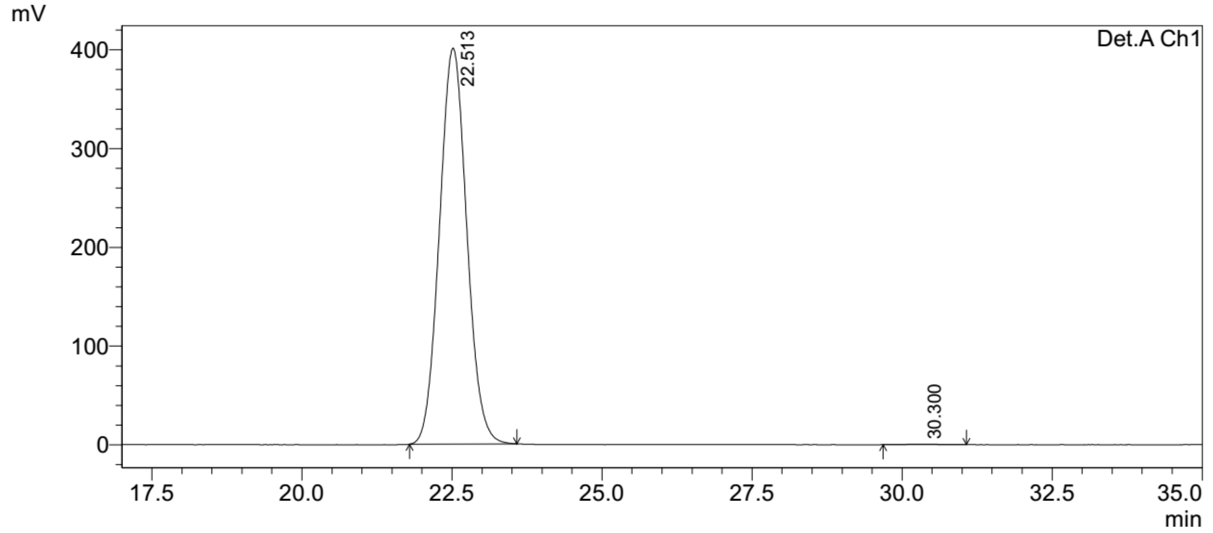

Detector A Ch1 254nm

Peak#	Ret. Time	Area	Height	Area %	Height %
1	22.513	12688316	401068	99.864	99.888
2	30.300	17279	449	0.136	0.112
Total		12705595	401517	100.000	100.000

Supplementary Figure 56. HPLC spectra of **9b**

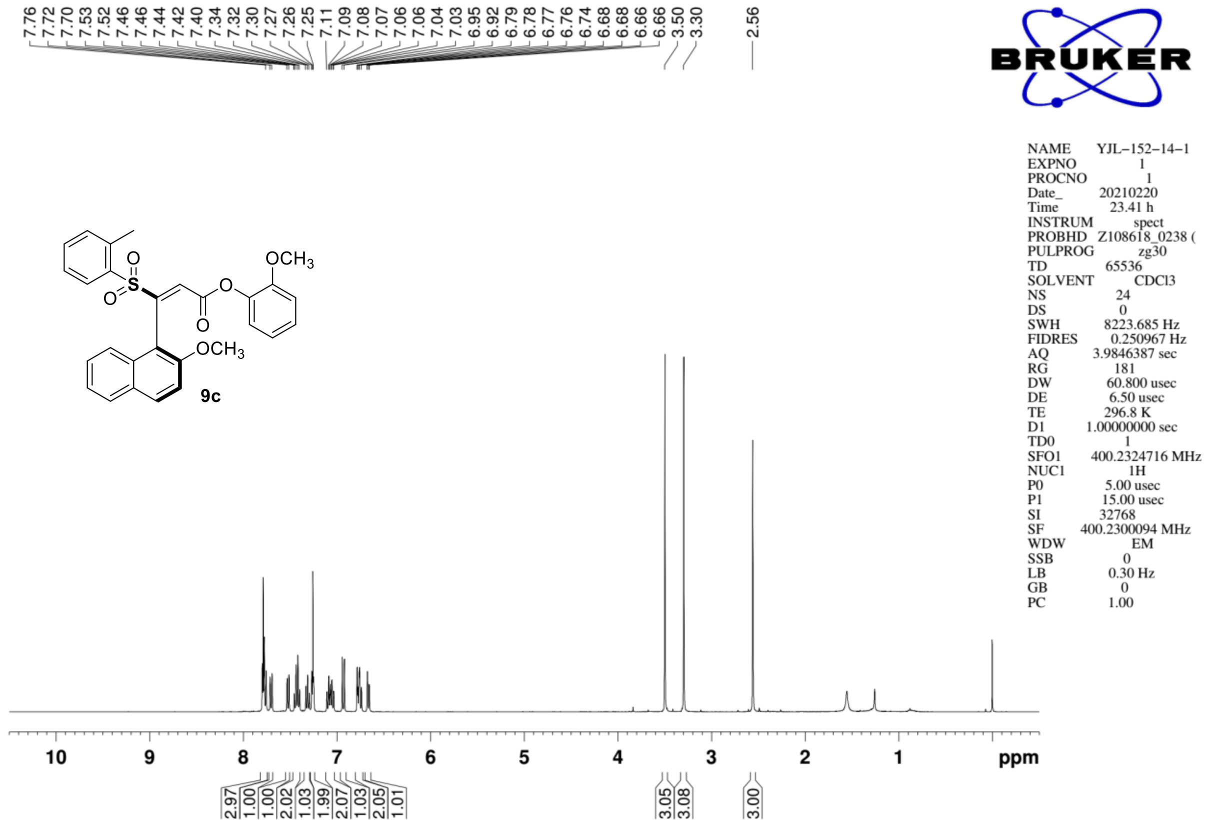

Supplementary Figure 57. ^{1}H NMR ($CDCl_3$, 400MHz) spectra of **9c**

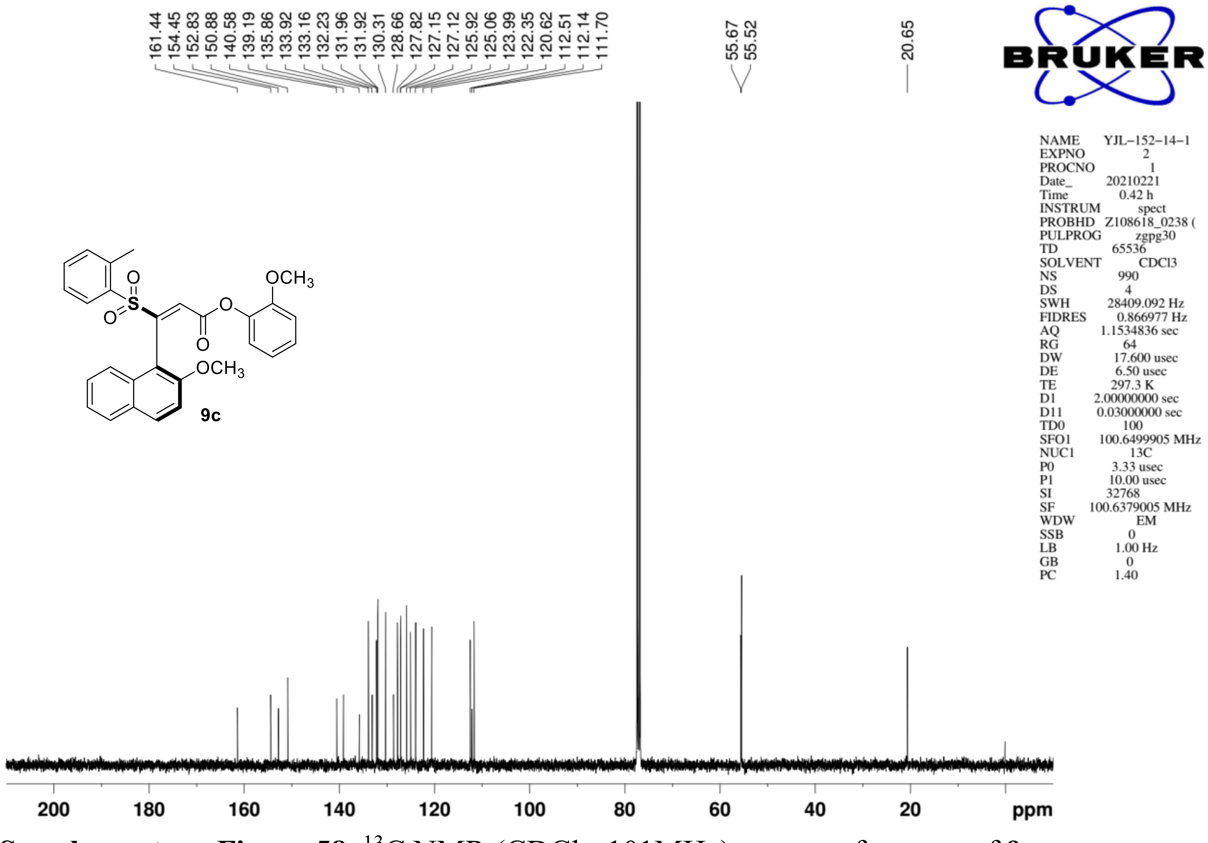

Supplementary Figure 58. ^{13}C NMR ($CDCl_3$, 101MHz) spectra of spectra of **9c**

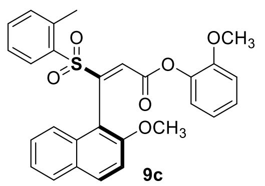

9c

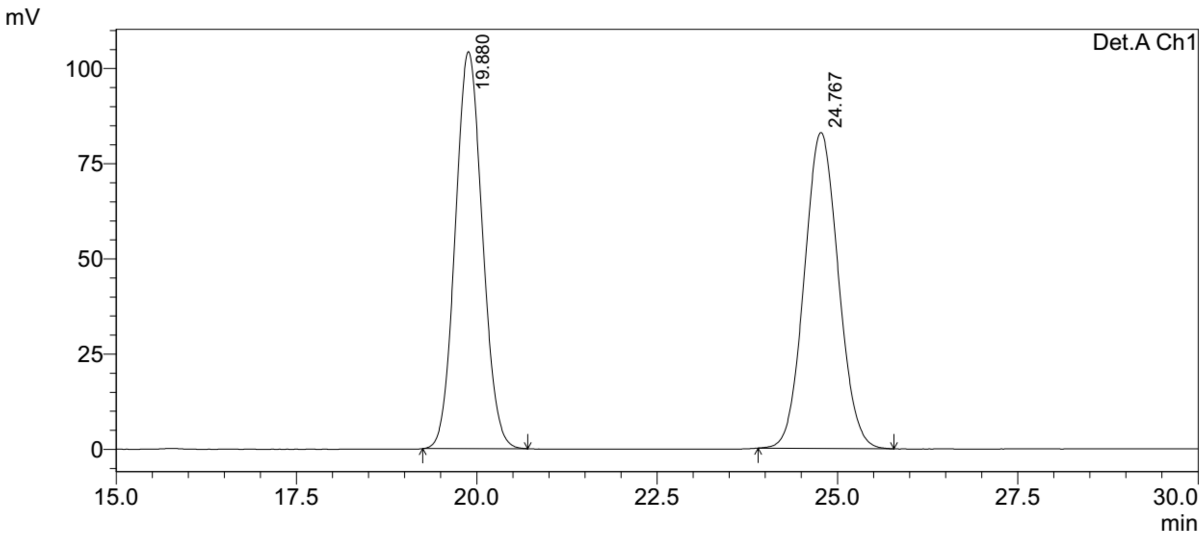

Detector A Ch1 254nm

Peak#	Ret. Time	Area	Height	Area %	Height %
1	19.880	2726956	104273	50.050	55.696
2	24.767	2721502	82946	49.950	44.304
Total		5448458	187219	100.000	100.000

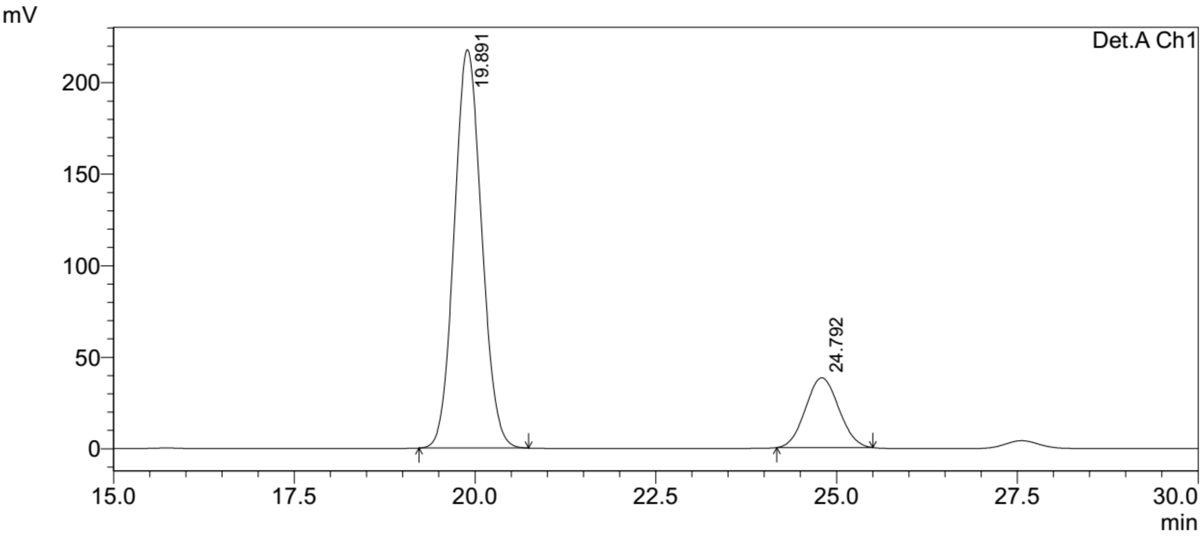

Detector A Ch1 254nm

Peak#	Ret. Time	Area	Height	Area %	Height %
1	19.891	5742650	217717	82.460	85.099
2	24.792	1221531	38124	17.540	14.901
Total		6964181	255841	100.000	100.000

Supplementary Figure 59. HPLC spectra of **9c**

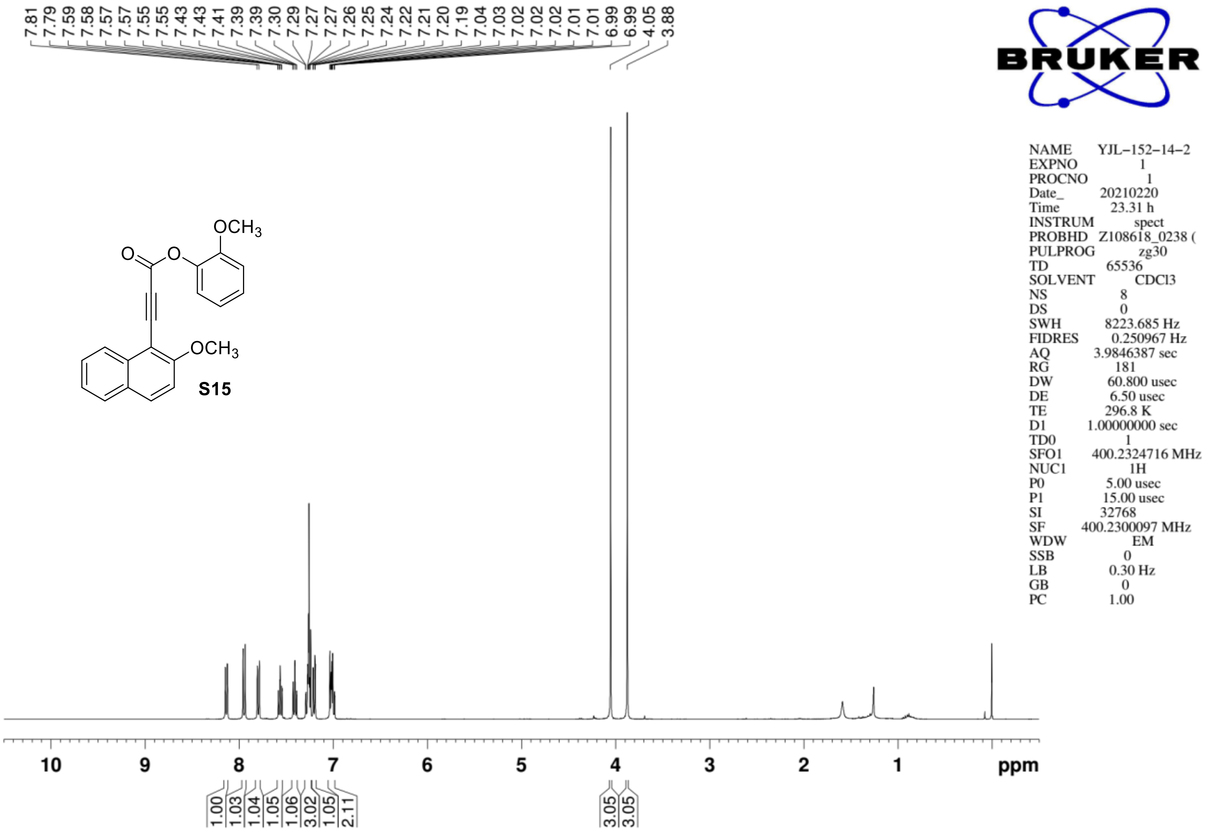

Supplementary Figure 60. ^{1}H NMR ($CDCl_3$, 400MHz) spectra of **S15**

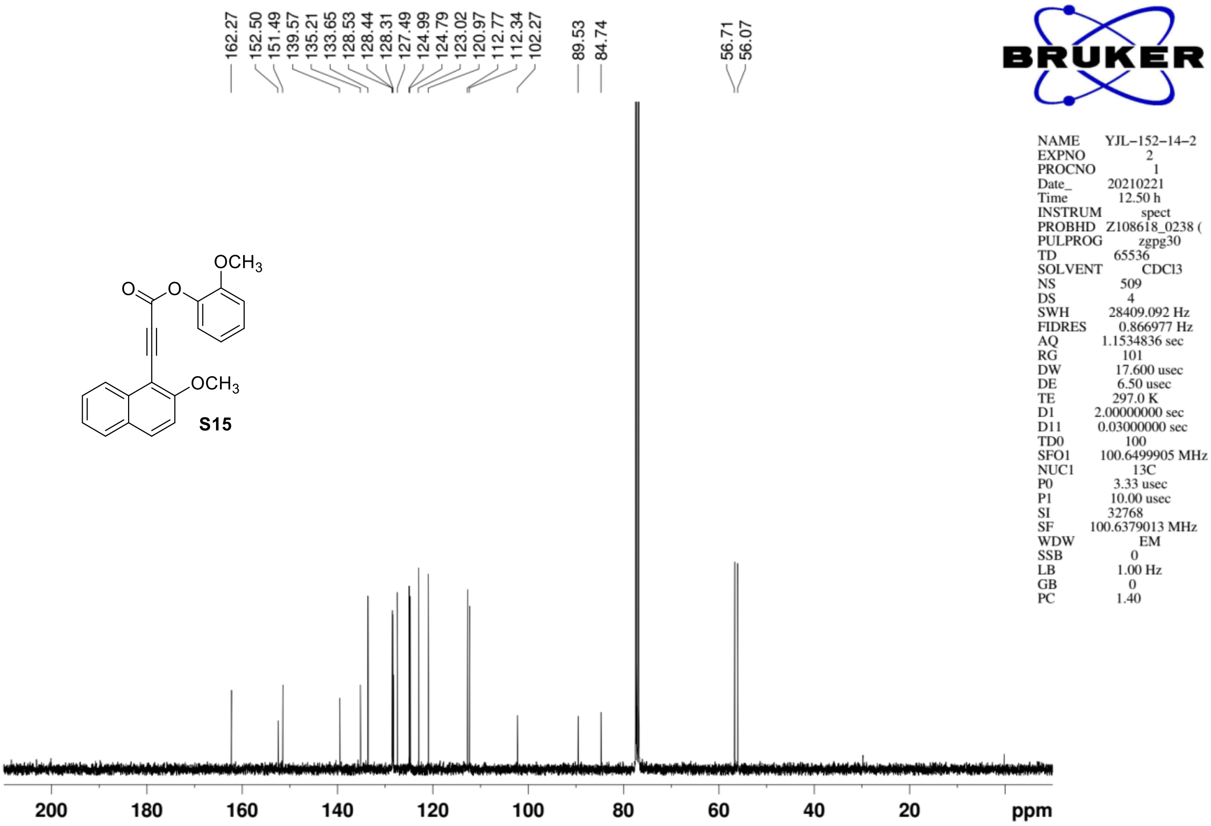

Supplementary Figure 61. ^{13}C NMR ($CDCl_3$, 101MHz) spectra of spectra of **S15**

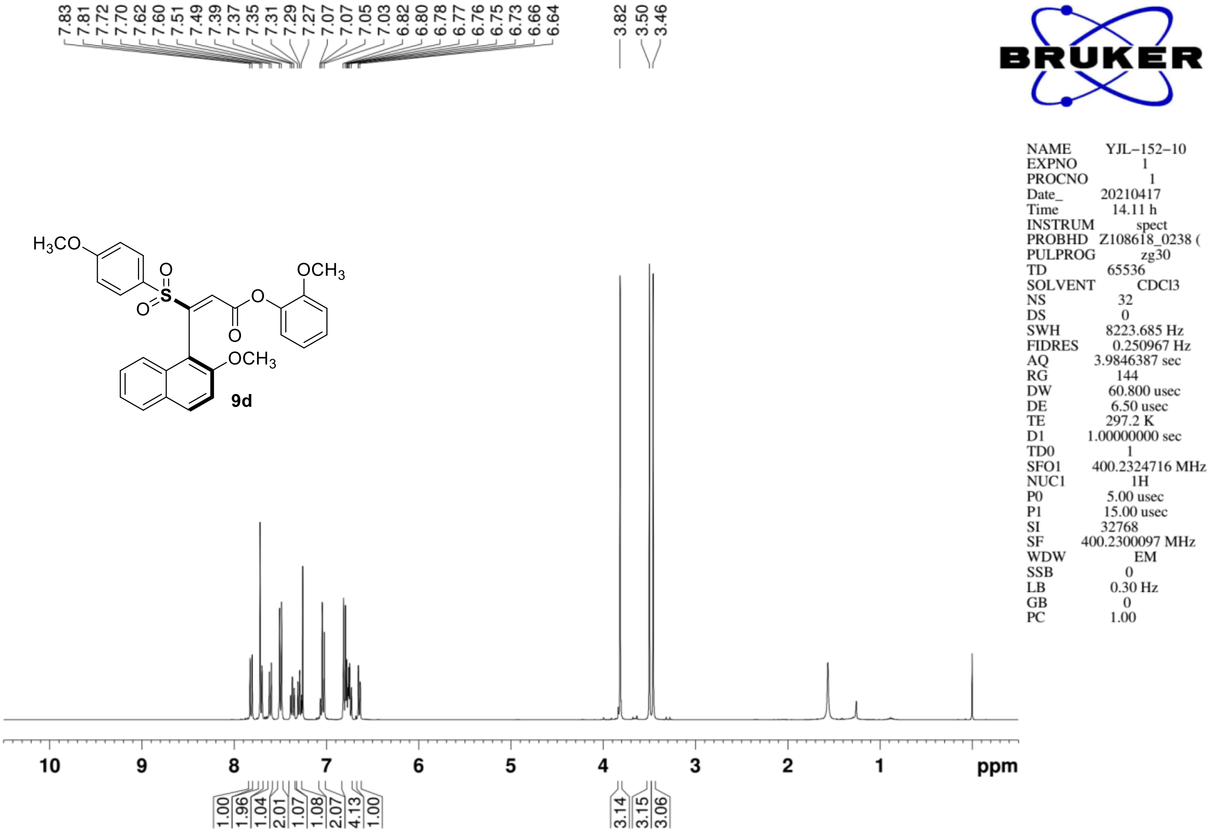

Supplementary Figure 62. ^{1}H NMR ($CDCl_3$, 400MHz) spectra of **9d**

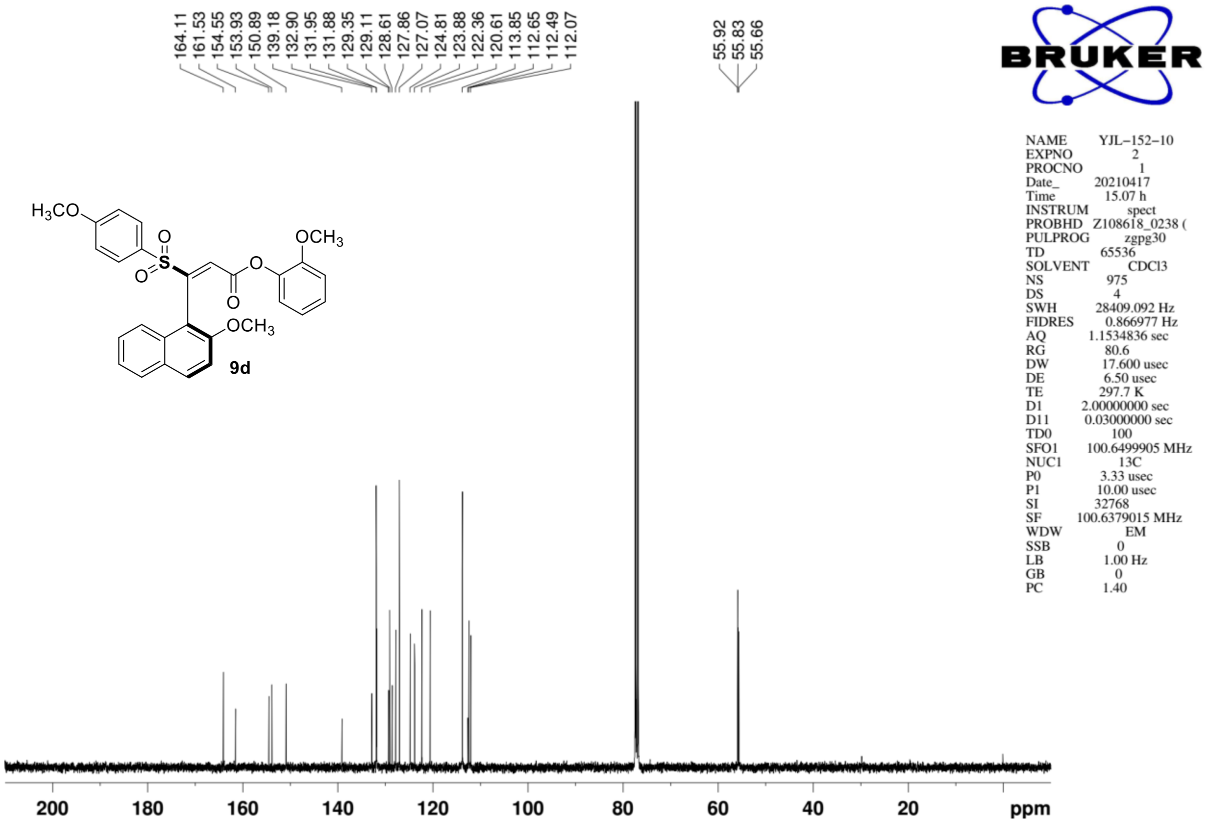

Supplementary Figure 63. ^{13}C NMR ($CDCl_3$, 101MHz) spectra of spectra of **9d**

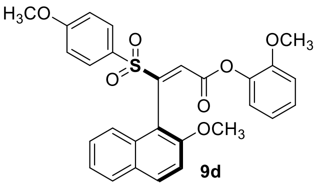

9d

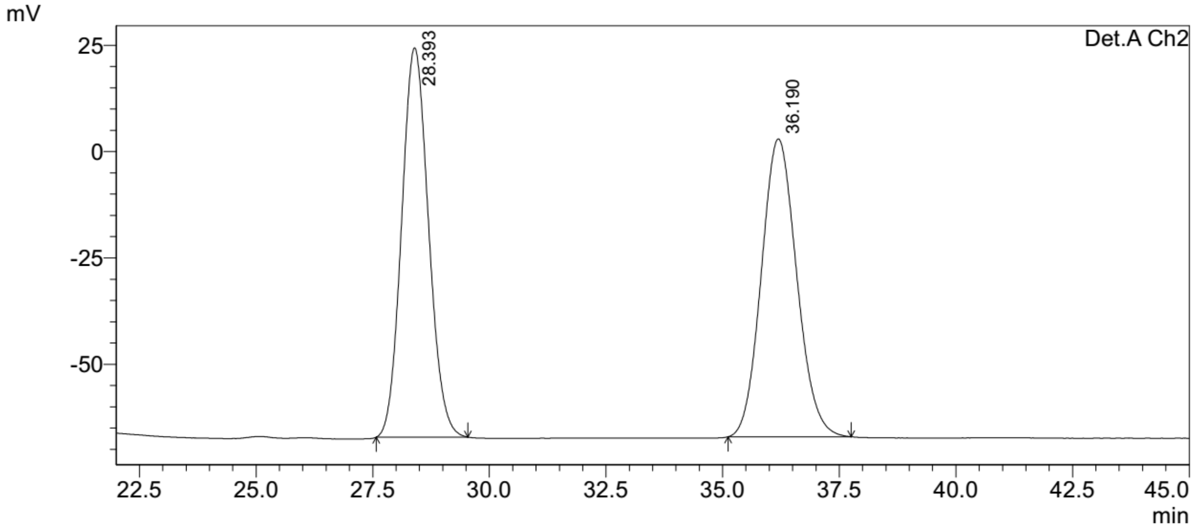

Detector A Ch2 220nm

Peak#	Ret. Time	Area	Height	Area %	Height %
1	28.393	3672542	91540	49.853	56.643
2	36.190	3694138	70068	50.147	43.357
Total		7366680	161608	100.000	100.000

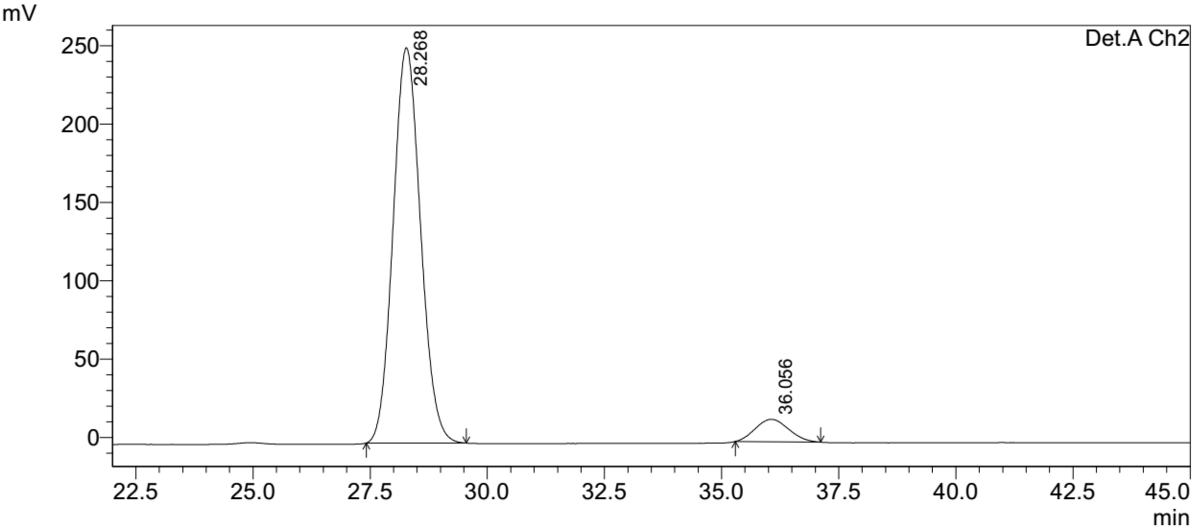

Detector A Ch2 220nm

Peak#	Ret. Time	Area	Height	Area %	Height %
1	28.268	10286226	252299	93.539	94.621
2	36.056	710450	14341	6.461	5.379
Total		10996676	266641	100.000	100.000

Supplementary Figure 64. HPLC spectra of **9d**

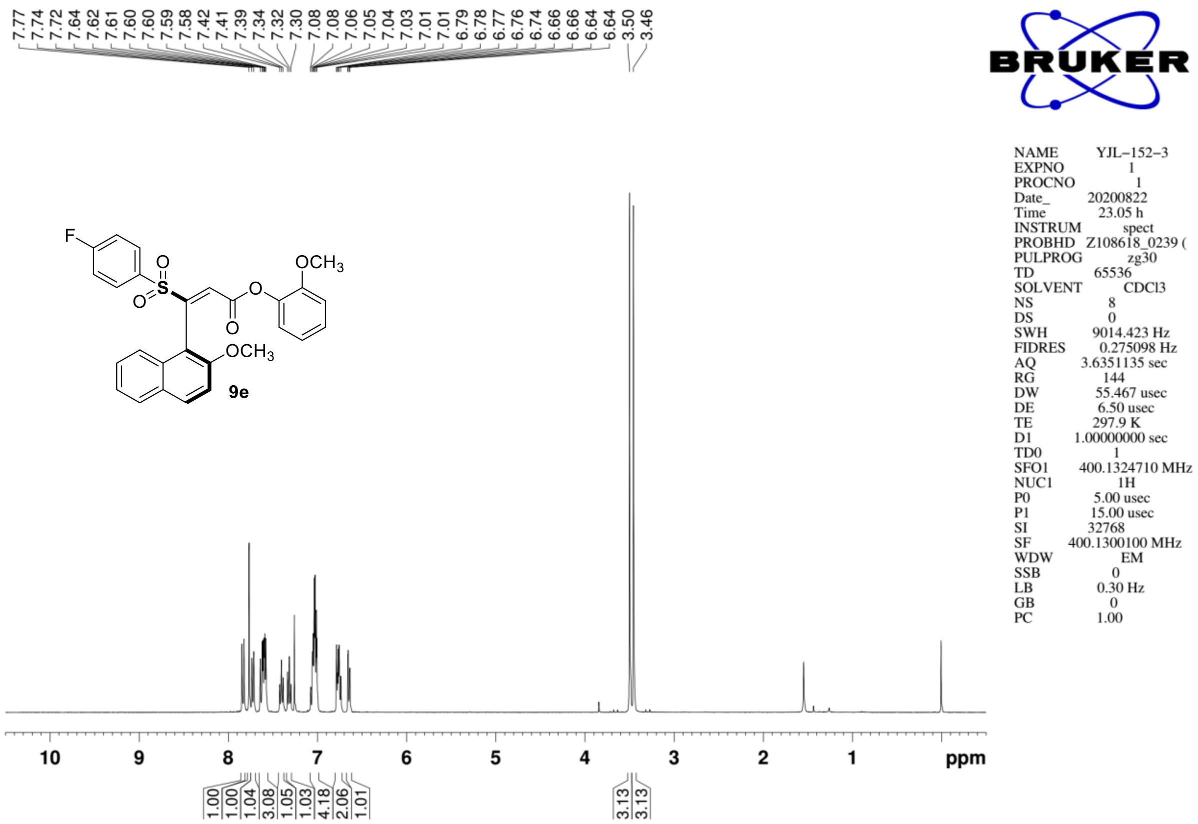

Supplementary Figure 65. ^{1}H NMR ($CDCl_3$, 400MHz) spectra of **9e**

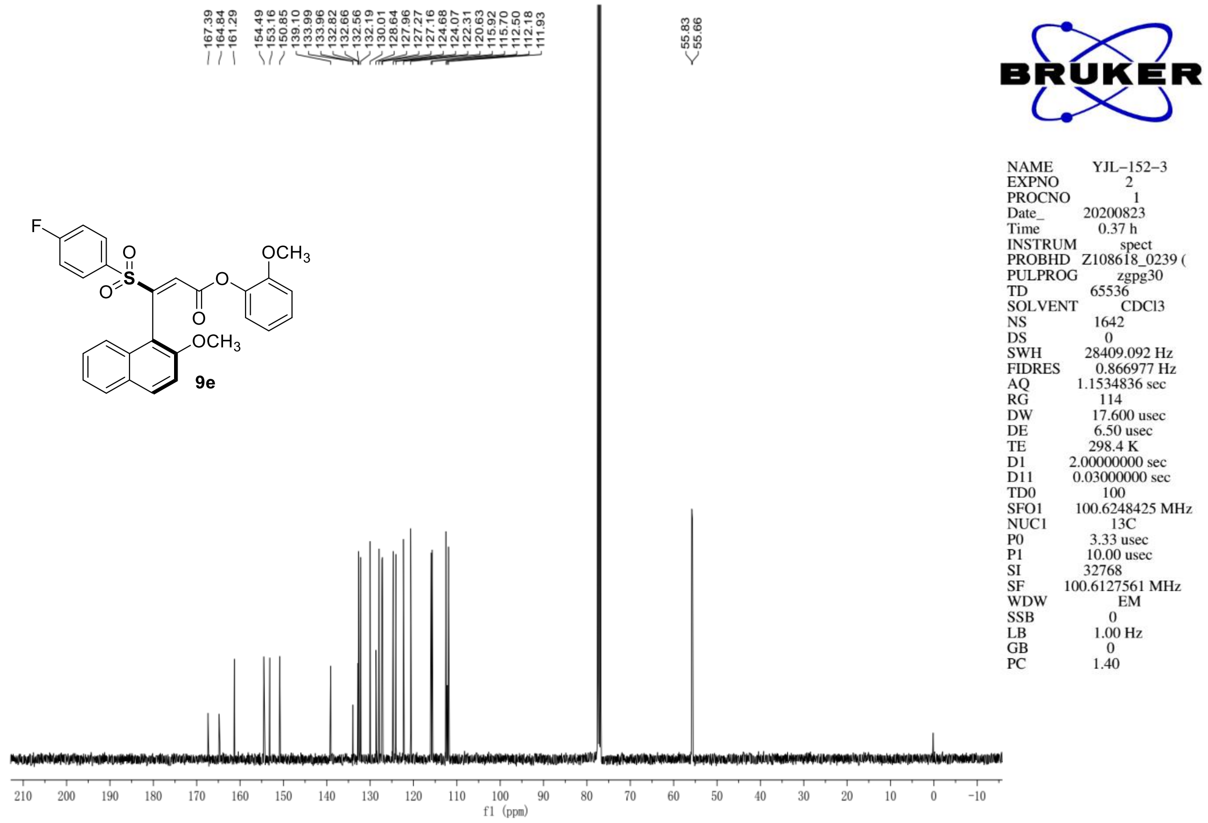

Supplementary Figure 66. ^{13}C NMR ($CDCl_3$, 101MHz) spectra of spectra of **9e**

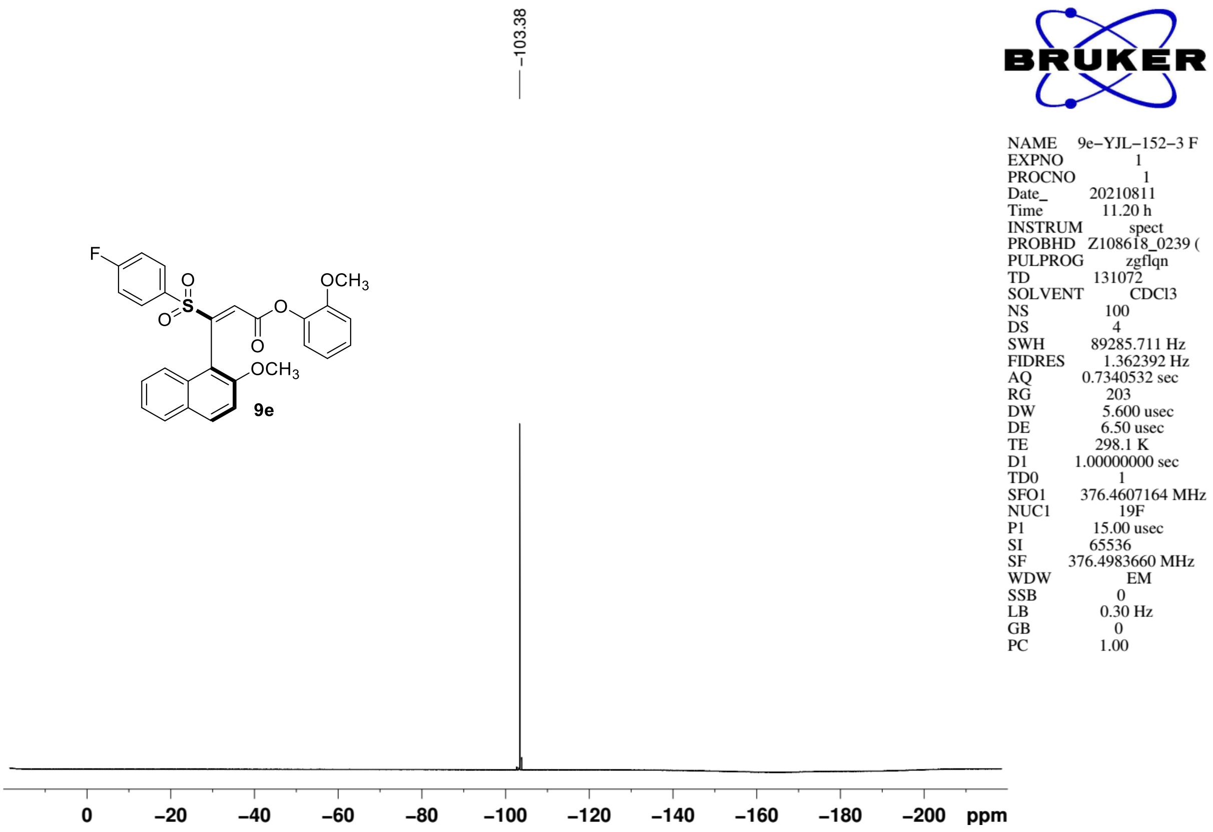

Supplementary Figure 67. ^{19}F NMR ($CDCl_3$, 376 MHz) spectra of spectra of **9e**

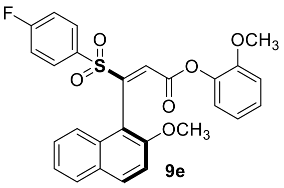

9e

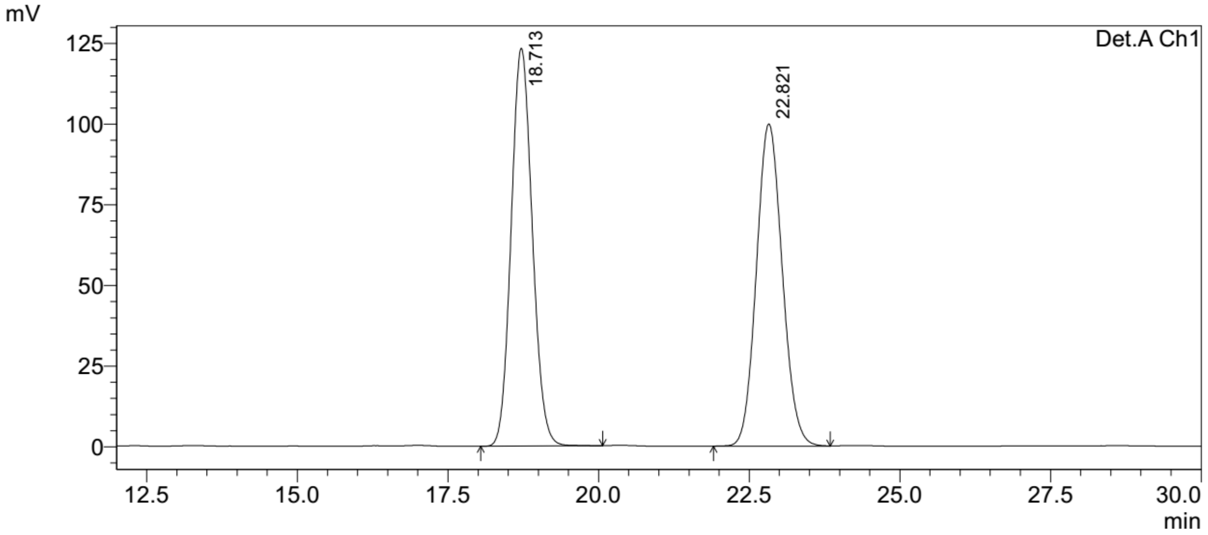

Detector A Ch1 254nm

Peak#	Ret. Time	Area	Height	Area %	Height %
1	18.713	3005909	123322	50.101	55.228
2	22.821	2993747	99973	49.899	44.772
Total		5999656	223296	100.000	100.000

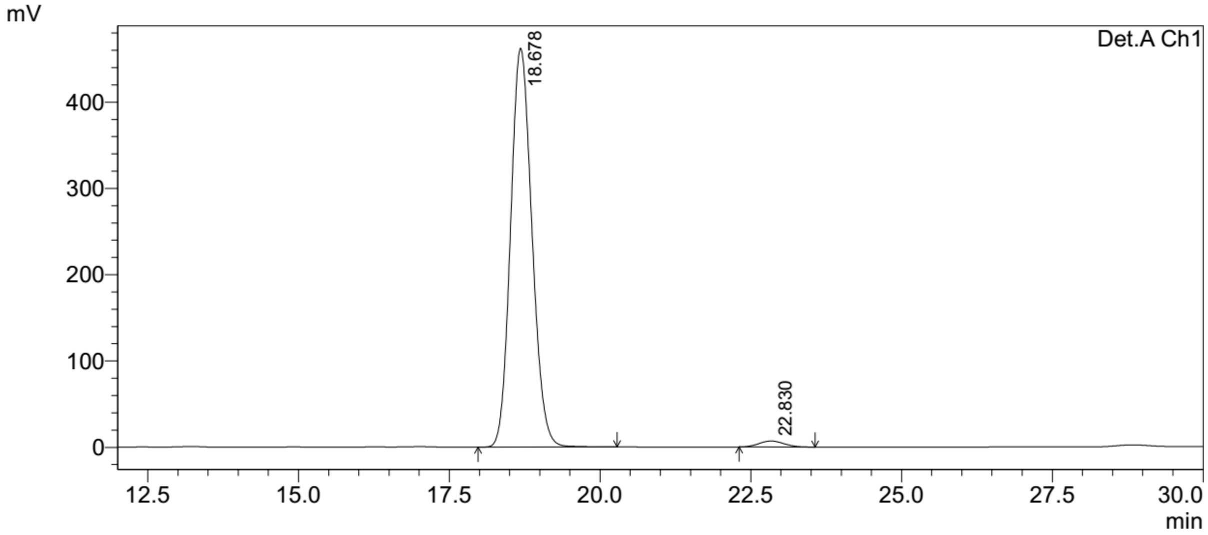

Detector A Ch1 254nm

Peak#	Ret. Time	Area	Height	Area %	Height %
1	18.678	11380558	462227	98.236	98.503
2	22.830	204353	7025	1.764	1.497
Total		11584911	469252	100.000	100.000

Supplementary Figure 68. HPLC spectra of **9e**

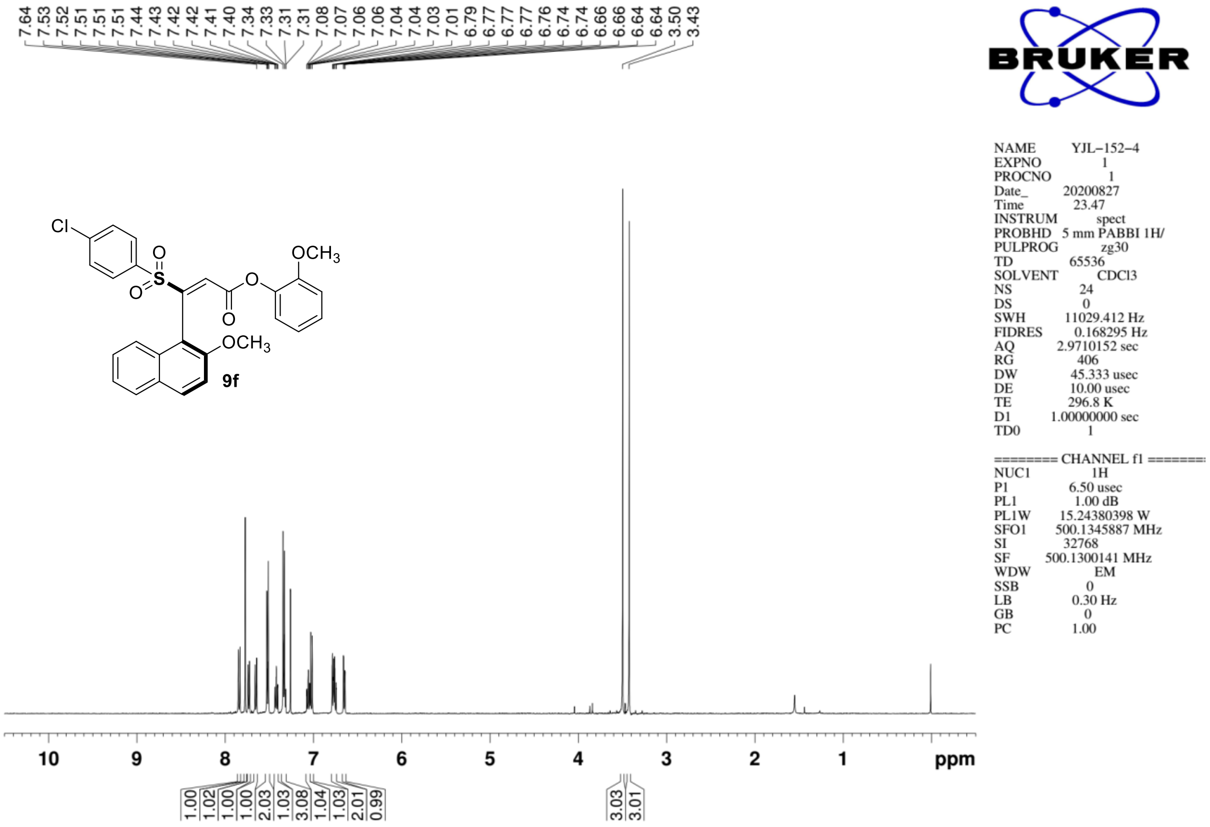

Supplementary Figure 69. ^{1}H NMR ($CDCl_3$, 500MHz) spectra of **9f**

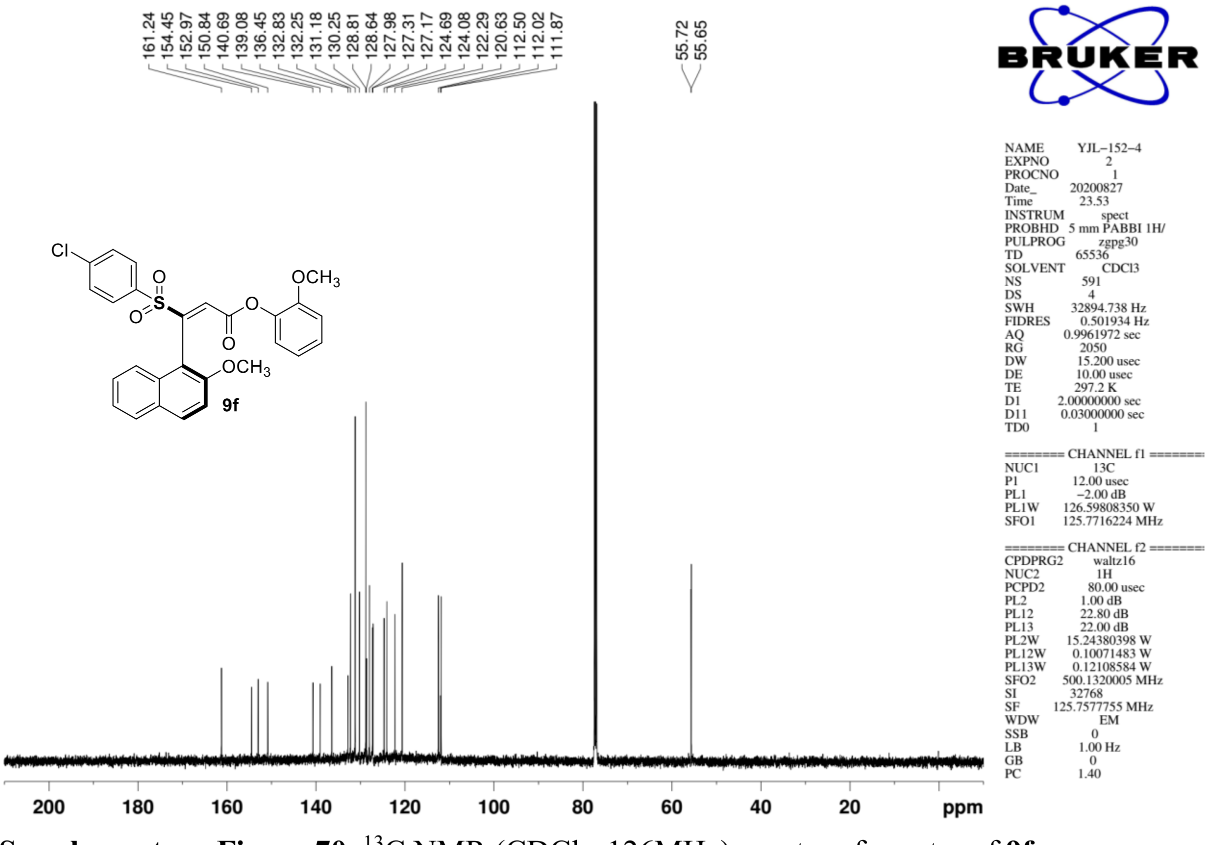

Supplementary Figure 70. ^{13}C NMR ($CDCl_3$, 126MHz) spectra of spectra of **9f**

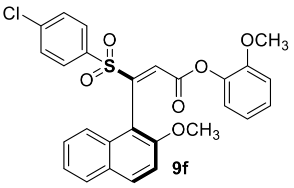

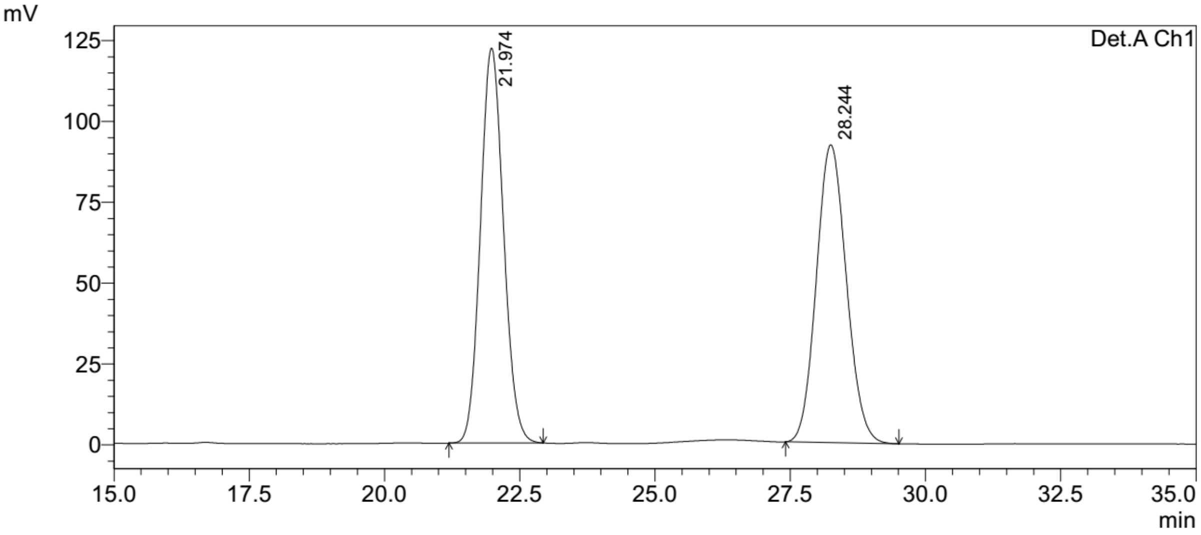

Detector A Ch1 254nm

Peak#	Ret. Time	Area	Height	Area %	Height %
1	21.974	3625156	122142	50.481	57.015
2	28.244	3556083	92085	49.519	42.985
Total		7181239	214227	100.000	100.000

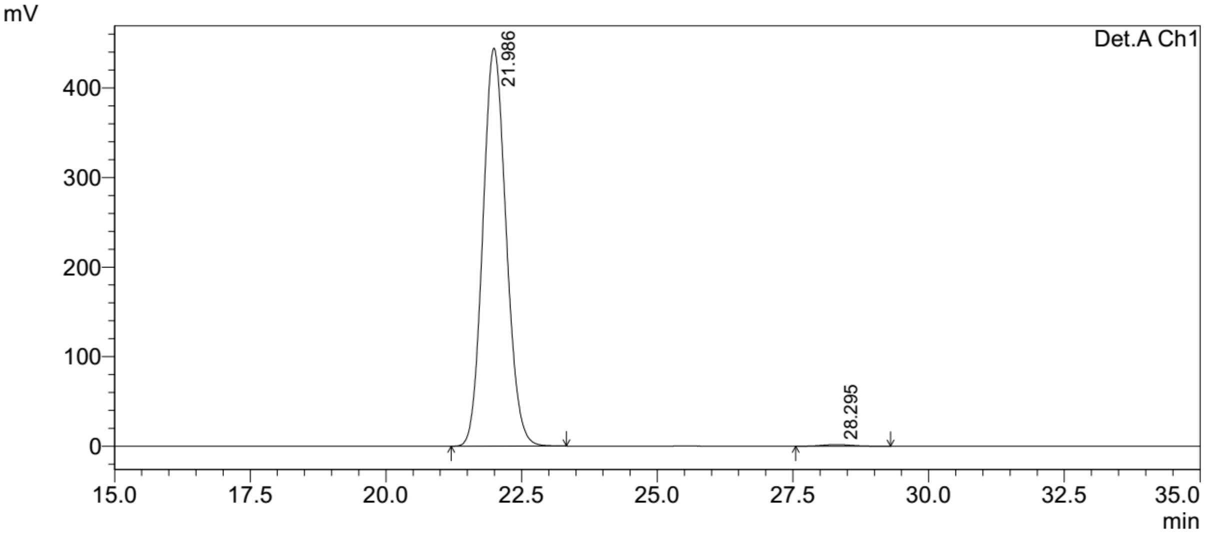

Detector A Ch1 254nm

Peak#	Ret. Time	Area	Height	Area %	Height %
1	21.986	13233456	444046	99.479	99.591
2	28.295	69256	1822	0.521	0.409
Total		13302712	445868	100.000	100.000

Supplementary Figure 71. HPLC spectra of **9f**

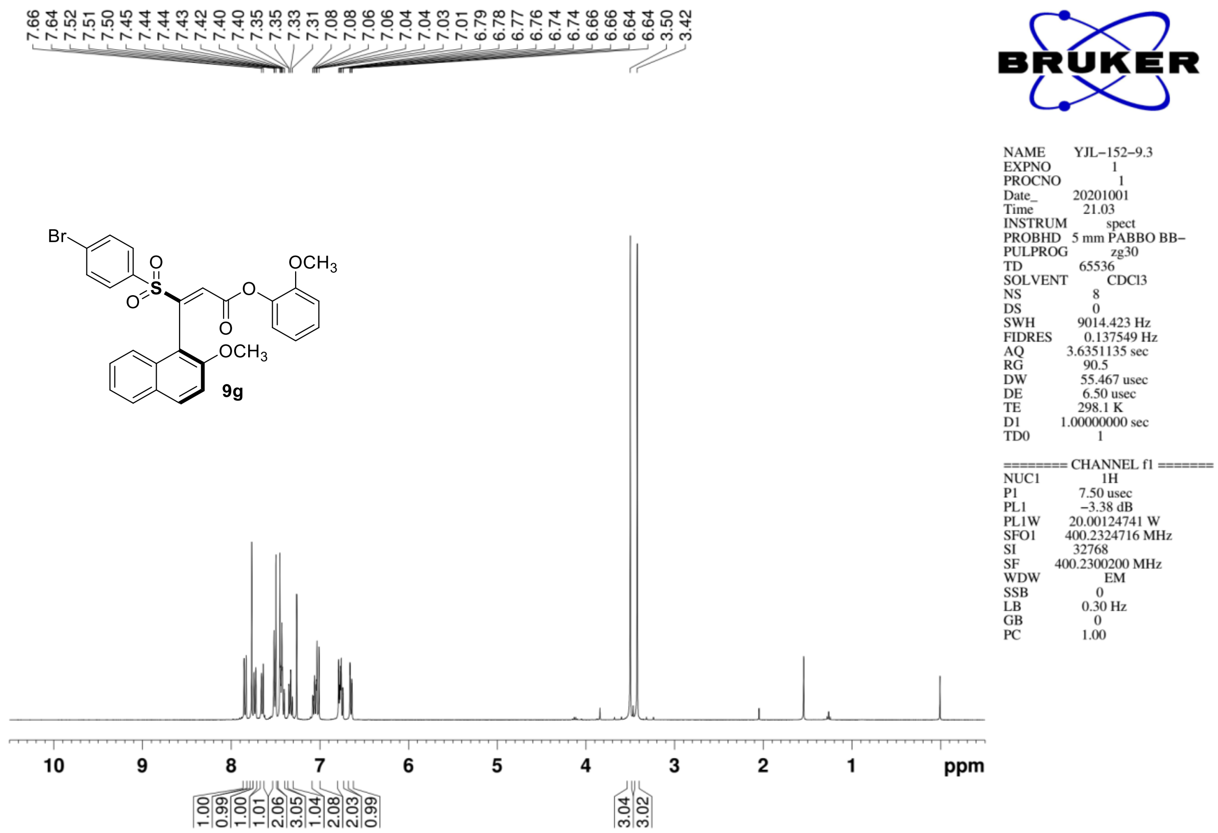

Supplementary Figure 72. ^{1}H NMR ($CDCl_3$, 400MHz) spectra of **9g**

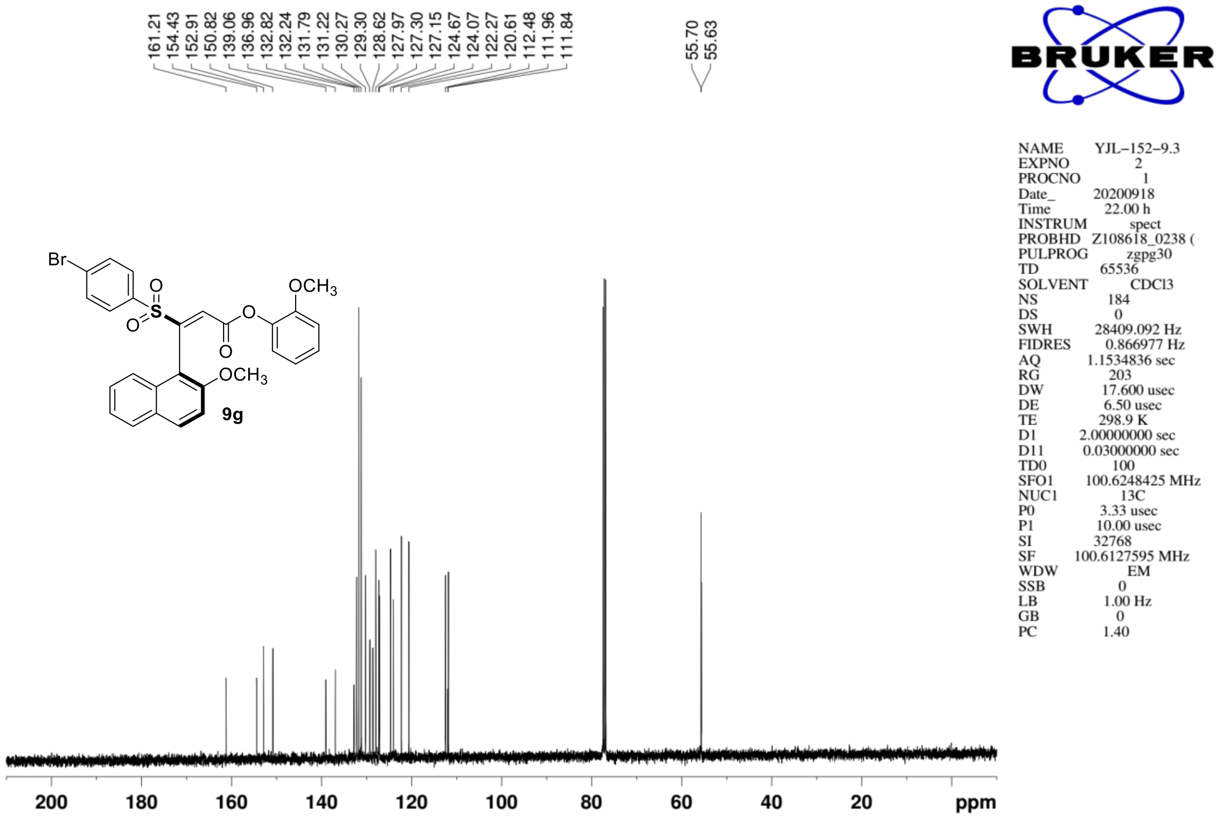

Supplementary Figure 73. ^{13}C NMR ($CDCl_3$, 101MHz) spectra of spectra of **9g**

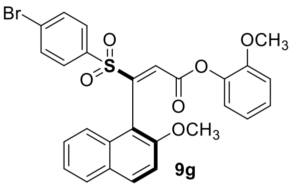

9g

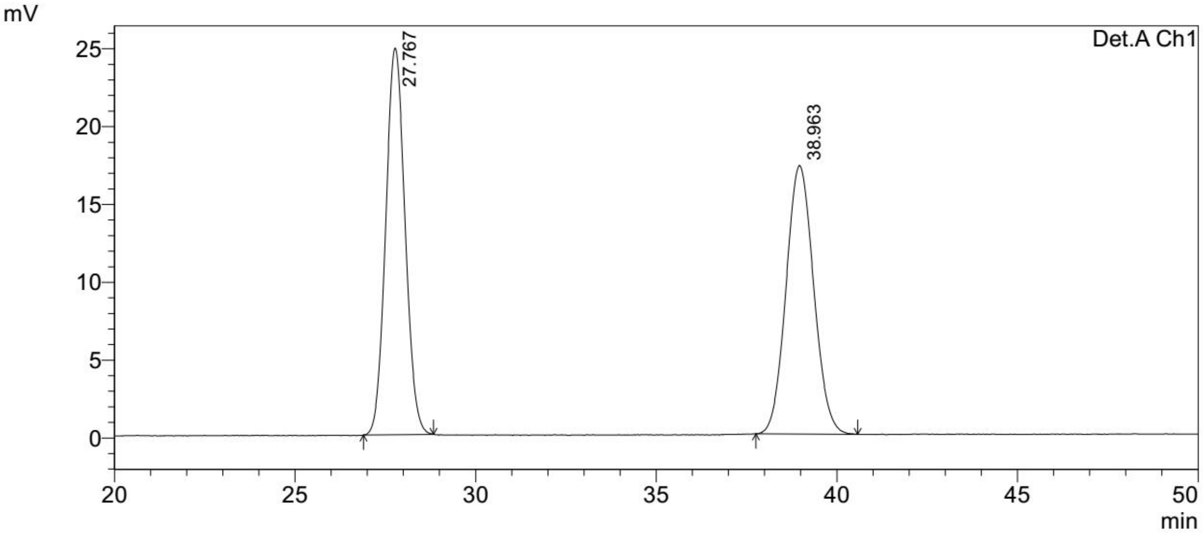

Detector A Ch1 254nm

Peak#	Ret. Time	Area	Height	Area %	Height %
1	27.767	924368	24834	50.184	58.993
2	38.963	917573	17262	49.816	41.007
Total		1841941	42096	100.000	100.000

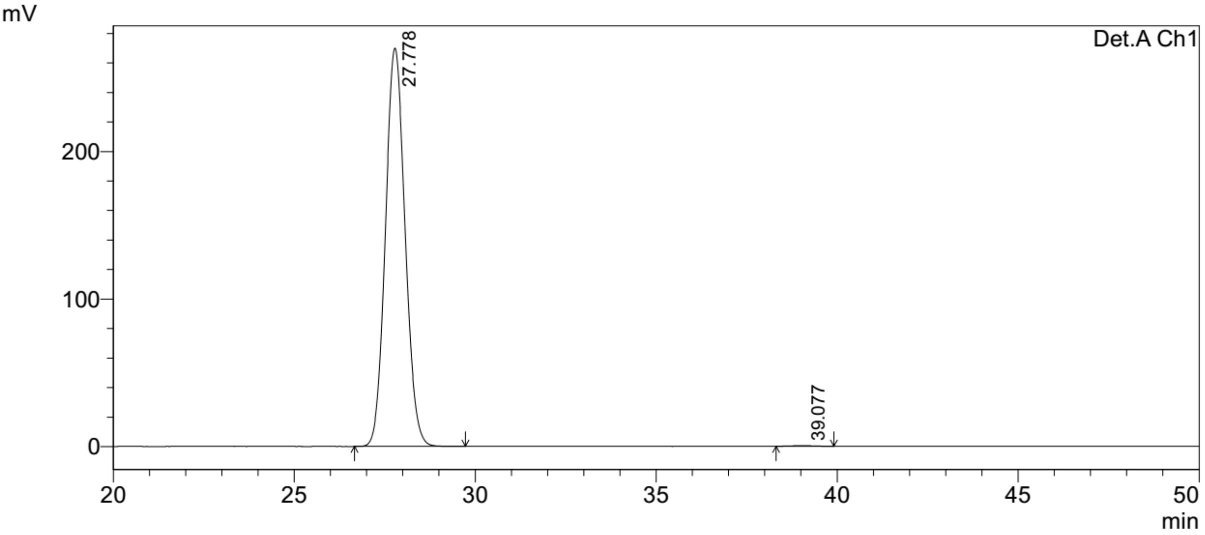

Detector A Ch1 254nm

Peak#	Ret. Time	Area	Height	Area %	Height %
1	27.778	10169197	269875	99.808	99.845
2	39.077	19551	418	0.192	0.155
Total		10188748	270293	100.000	100.000

Supplementary Figure 74. HPLC spectra of **9g**

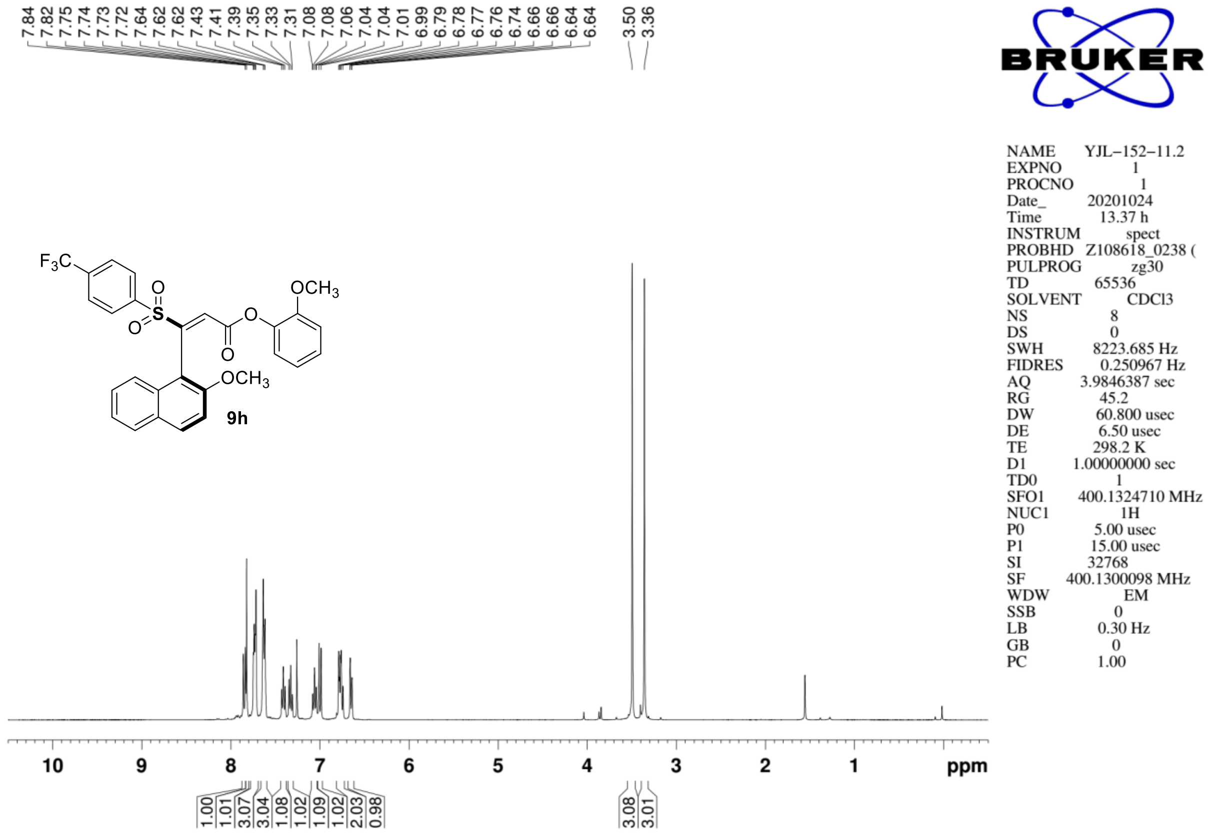

Supplementary Figure 75. ^{1}H NMR ($CDCl_3$, 400MHz) spectra of **9h**

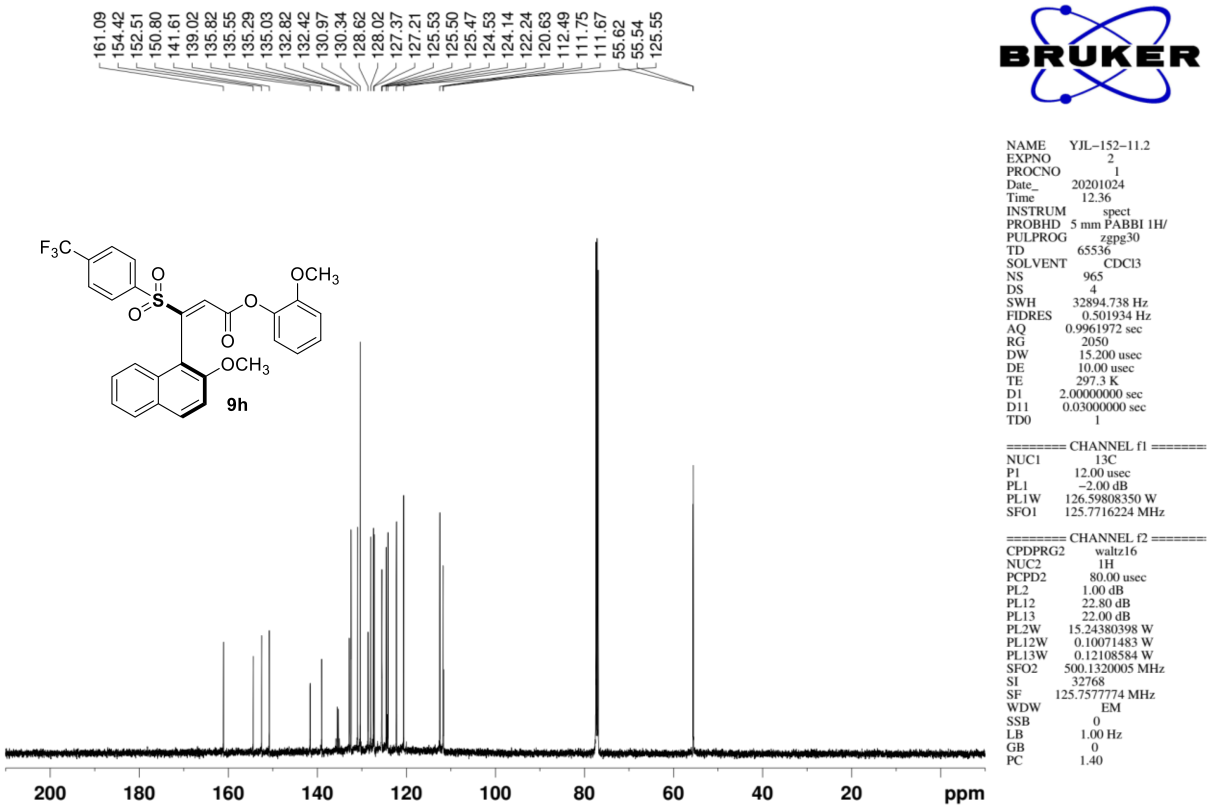

Supplementary Figure 76. ^{13}C NMR ($CDCl_3$, 126MHz) spectra of spectra of **9h**

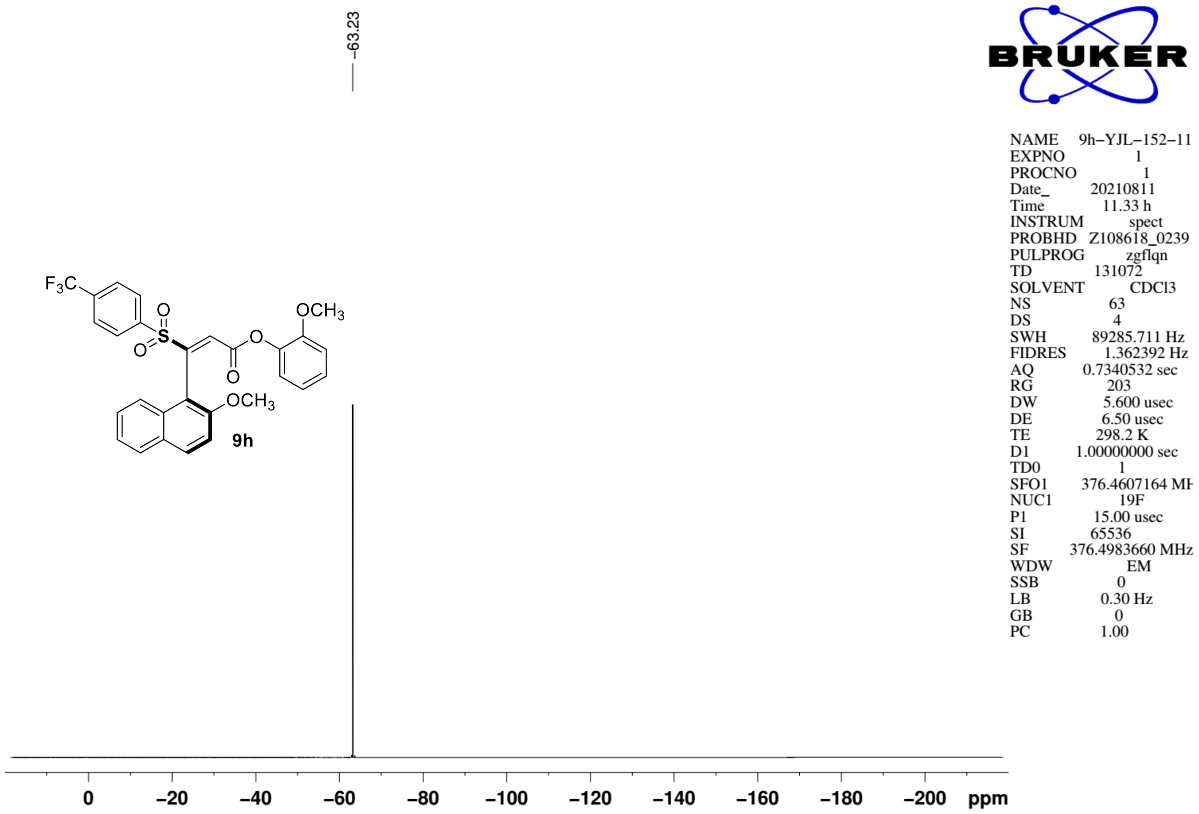

Supplementary Figure 77. ^{19}F NMR ($CDCl_3$, 376 MHz) spectra of spectra of **9h**

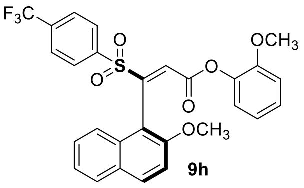

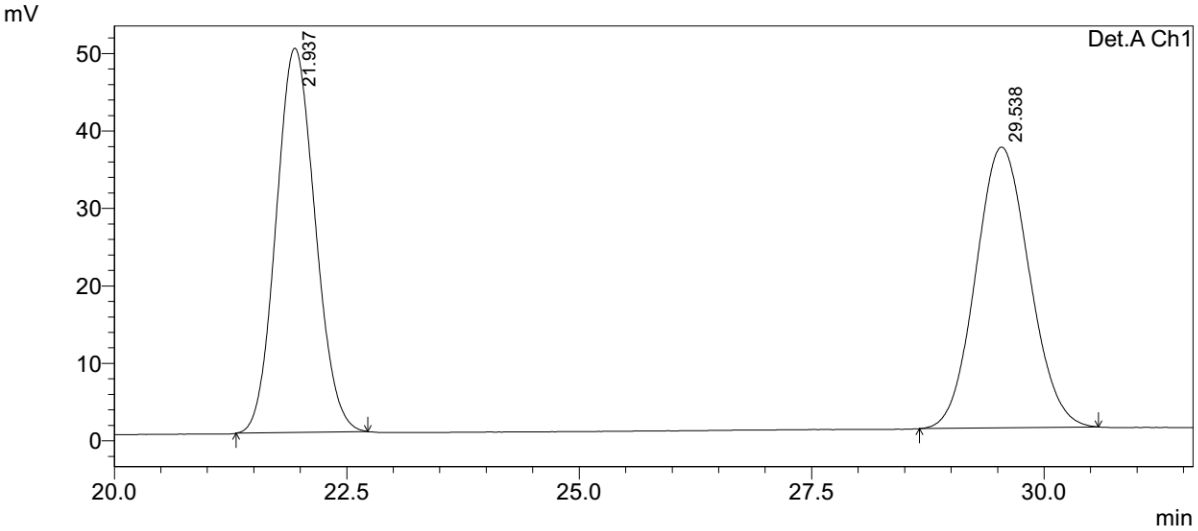

Detector A Ch1 254nm

Peak#	Ret. Time	Area	Height	Area %	Height %
1	21.937	1449082	49613	49.955	57.774
2	29.538	1451669	36262	50.045	42.226
Total		2900752	85875	100.000	100.000

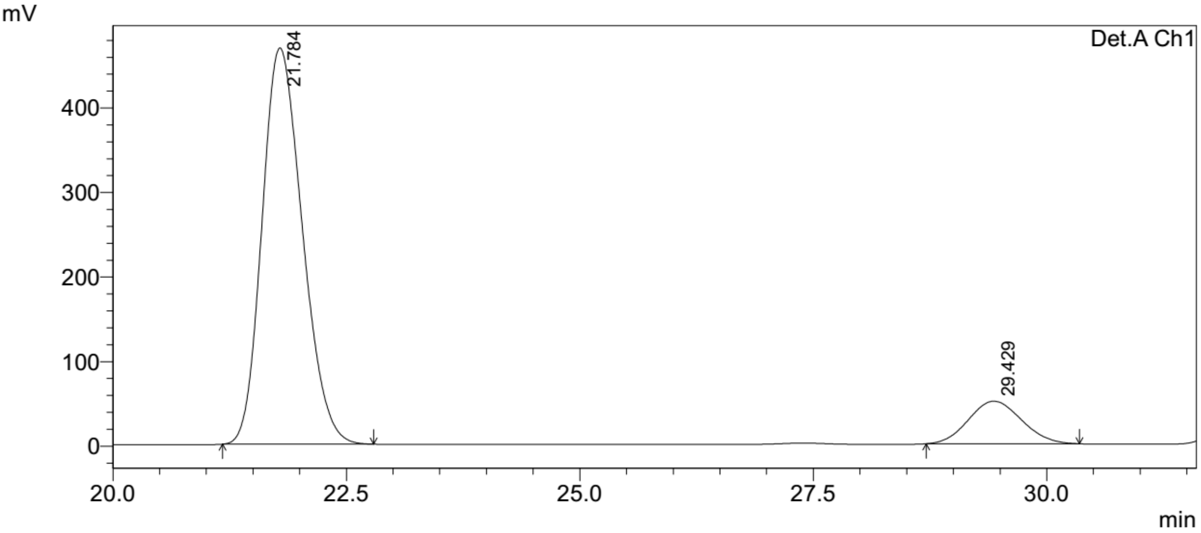

Detector A Ch1 254nm

Peak#	Ret. Time	Area	Height	Area %	Height %
1	21.784	14073939	468763	87.590	90.299
2	29.429	1994053	50362	12.410	9.701
Total		16067993	519126	100.000	100.000

Supplementary Figure 78. HPLC spectra of **9h**

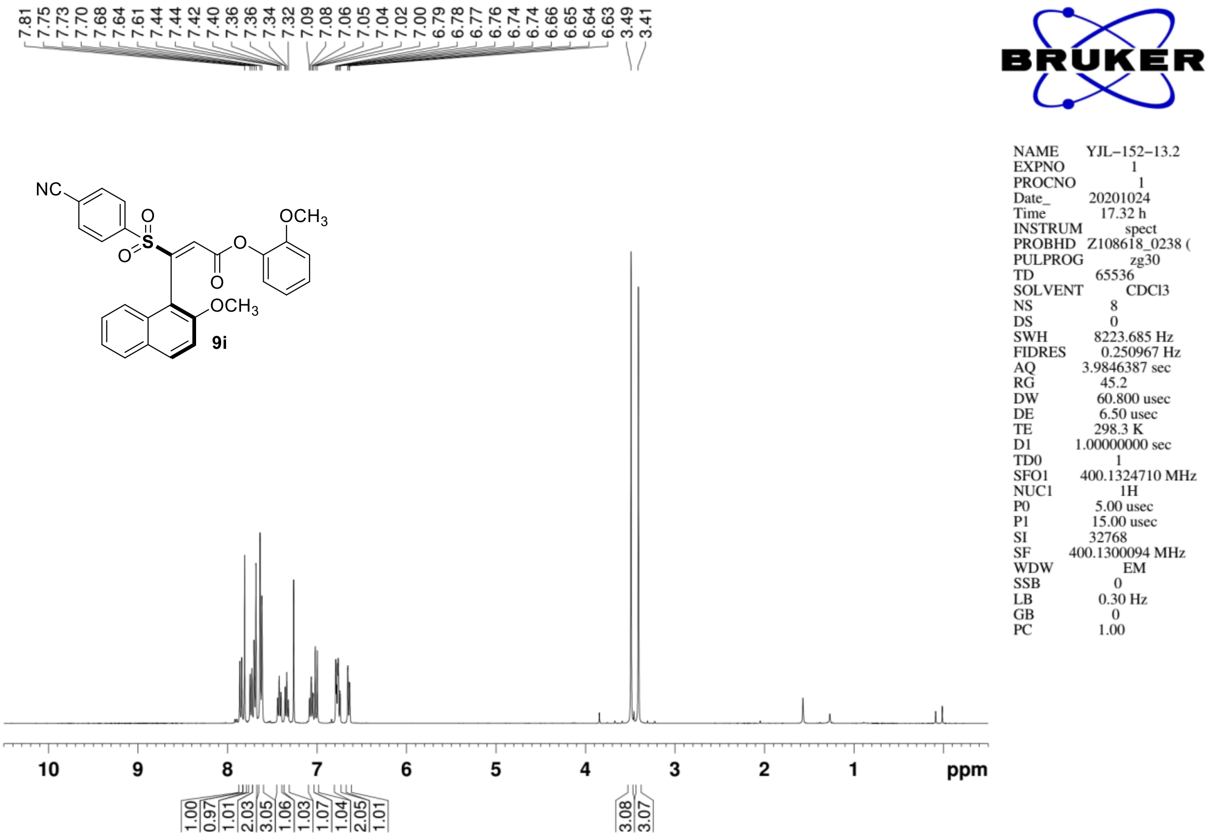

Supplementary Figure 79. ^{1}H NMR ($CDCl_3$, 400MHz) spectra of **9i**

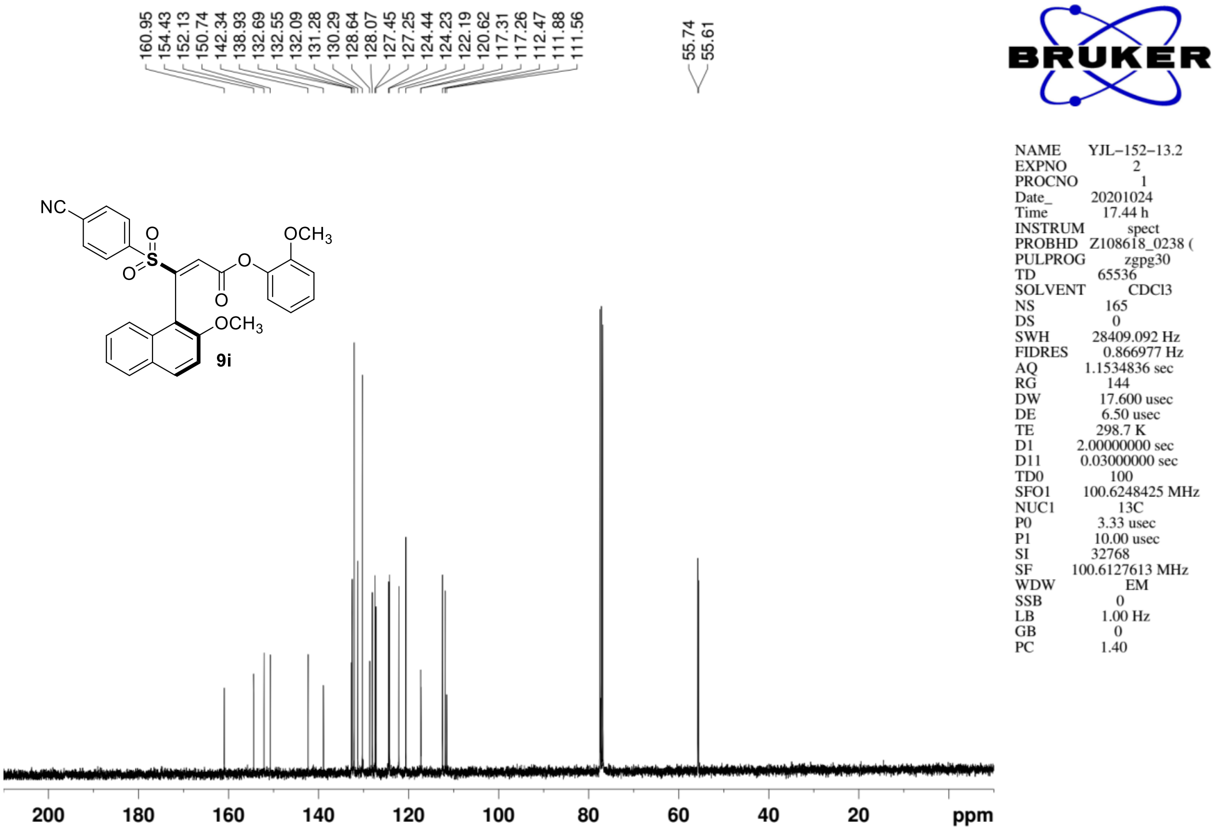

Supplementary Figure 80. ^{13}C NMR ($CDCl_3$, 101MHz) spectra of spectra of **9i**

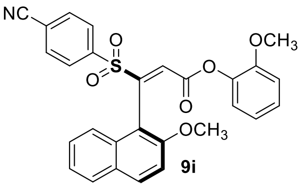

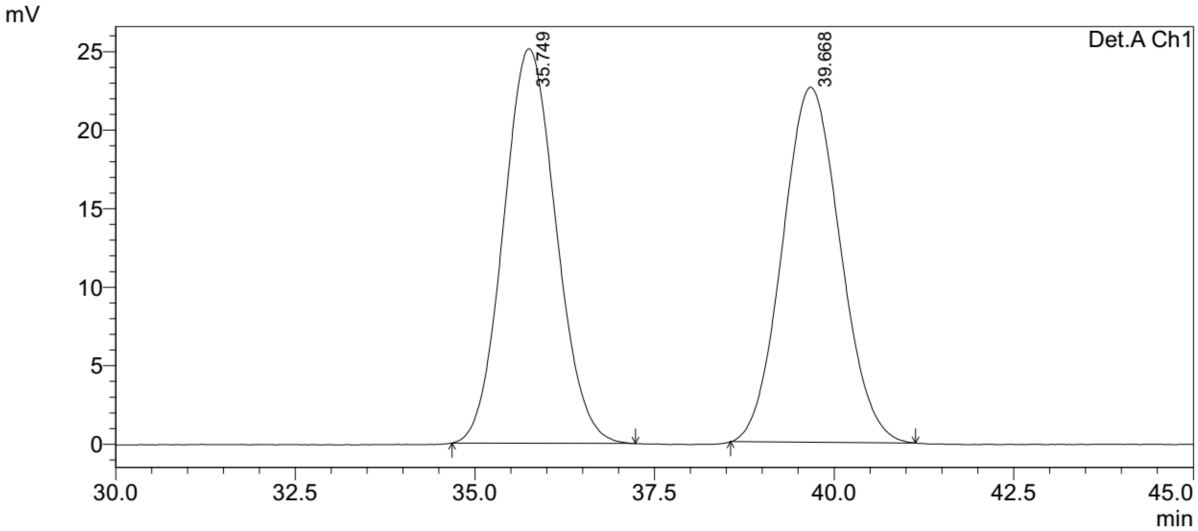

Detector A Ch1 254nm

Peak#	Ret. Time	Area	Height	Area %	Height %
1	35.749	1281043	25112	50.260	52.629
2	39.668	1267765	22602	49.740	47.371
Total		2548808	47714	100.000	100.000

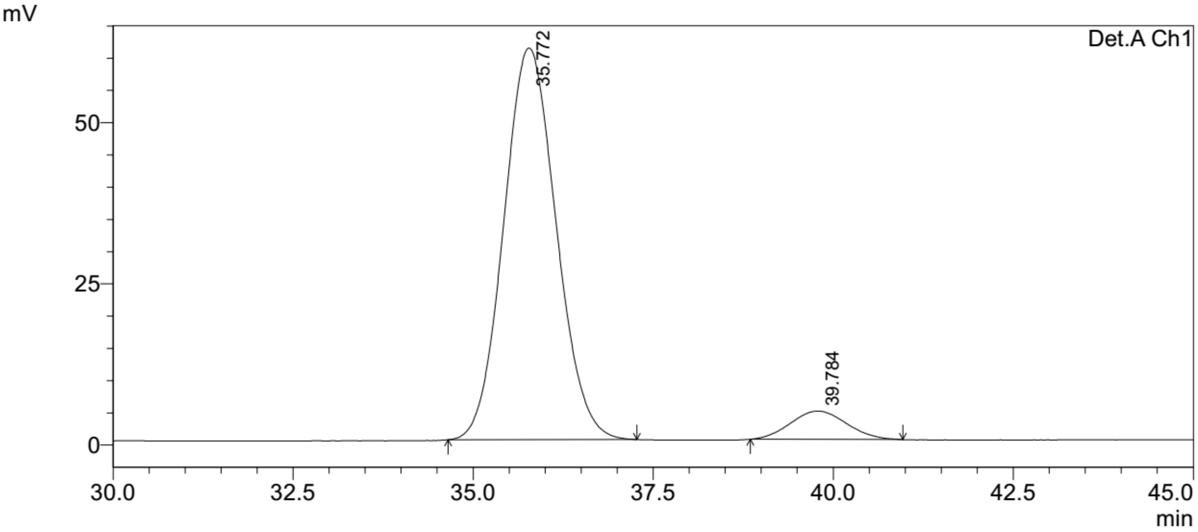

Detector A Ch1 254nm

Peak#	Ret. Time	Area	Height	Area %	Height %
1	35.772	3075045	60744	92.811	93.246
2	39.784	238194	4400	7.189	6.754
Total		3313239	65144	100.000	100.000

Supplementary Figure 81. HPLC spectra of **9i**

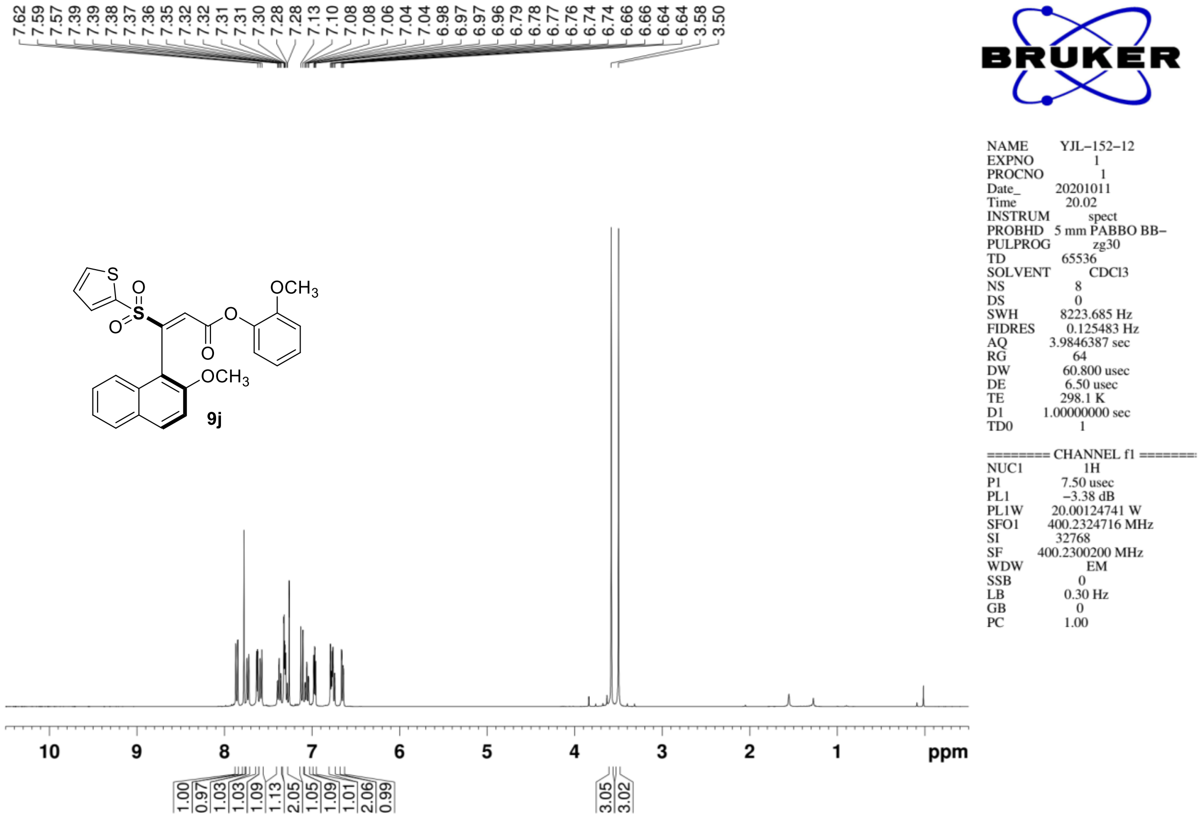

Supplementary Figure 82. ^{1}H NMR ($CDCl_3$, 400MHz) spectra of **9j**

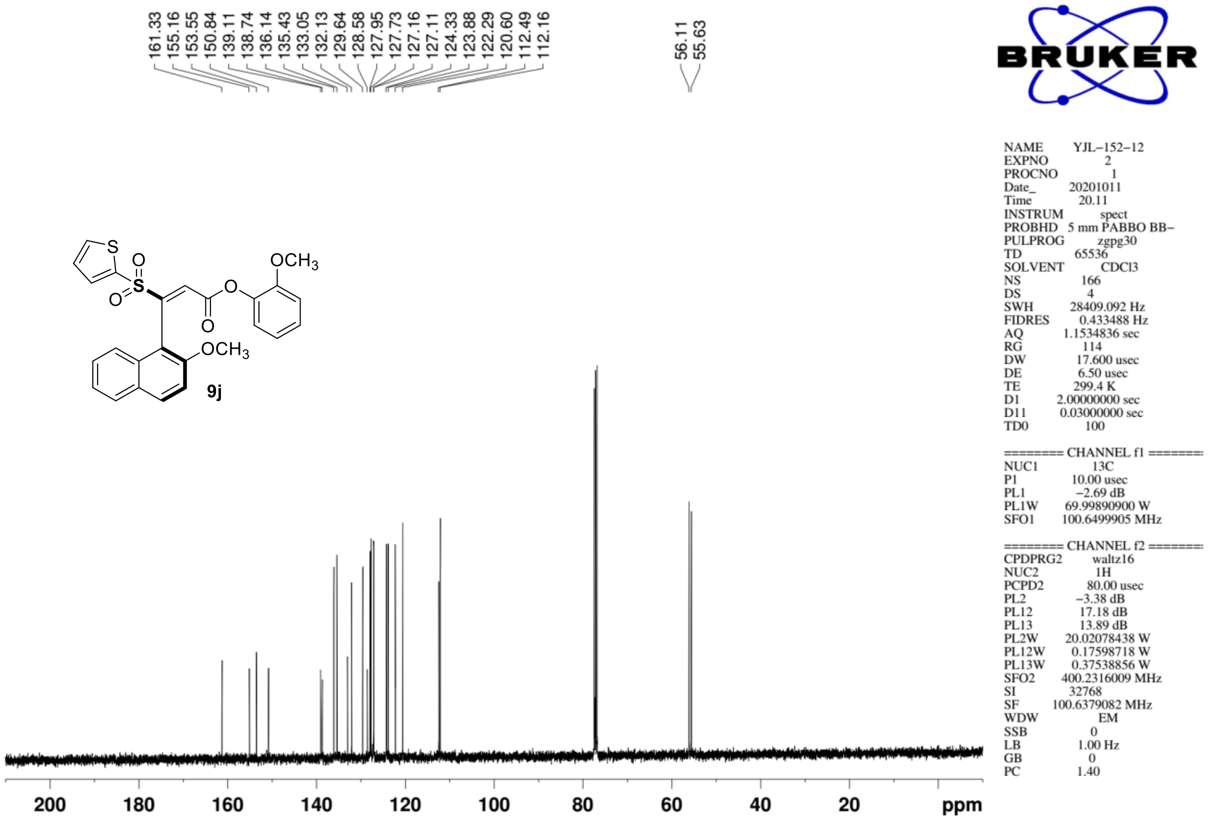

Supplementary Figure 83. ^{13}C NMR ($CDCl_3$, 101MHz) spectra of spectra of **9j**

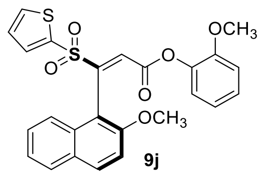

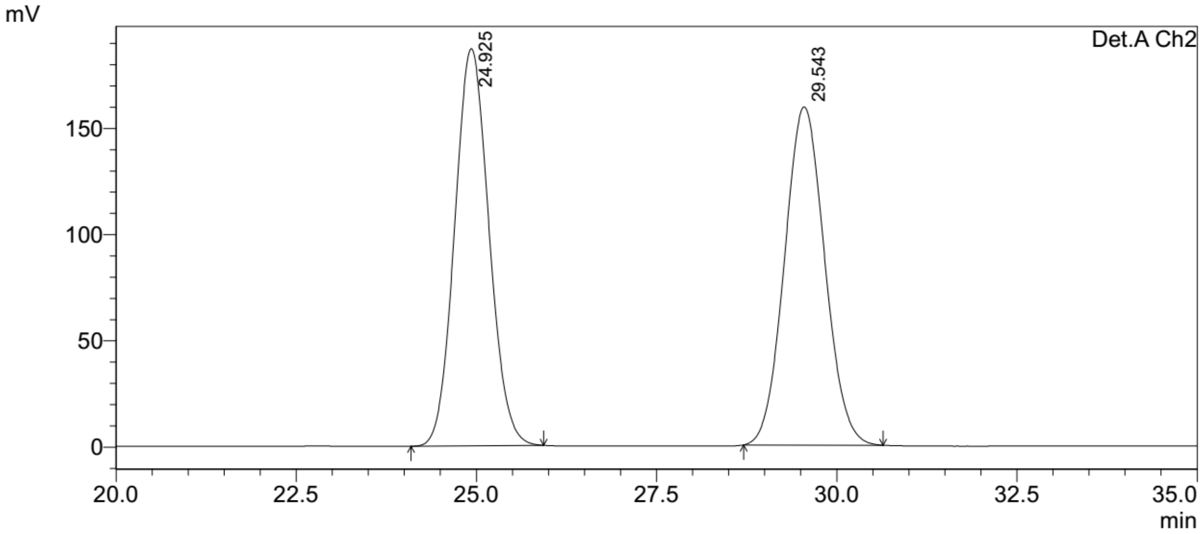

Detector A Ch2 220nm

Peak#	Ret. Time	Area	Height	Area %	Height %
1	24.925	6196940	186983	50.104	54.007
2	29.543	6171285	159238	49.896	45.993
Total		12368224	346221	100.000	100.000

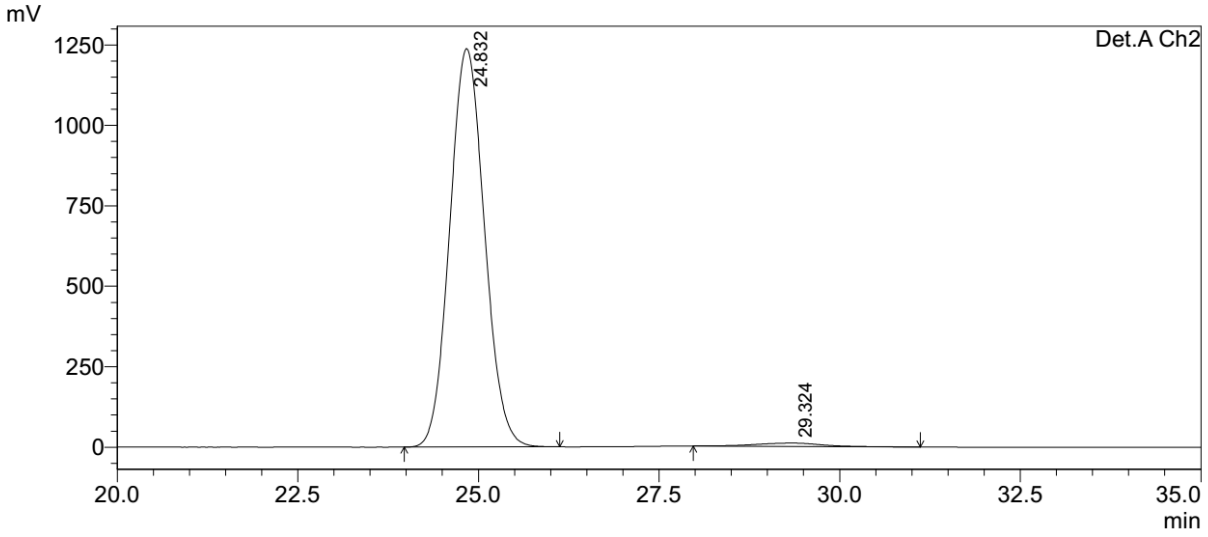

Detector A Ch2 220nm

Peak#	Ret. Time	Area	Height	Area %	Height %
1	24.832	41454910	1238175	98.429	99.172
2	29.324	661531	10342	1.571	0.828
Total		42116442	1248517	100.000	100.000

Supplementary Figure 84. HPLC spectra of **9j**

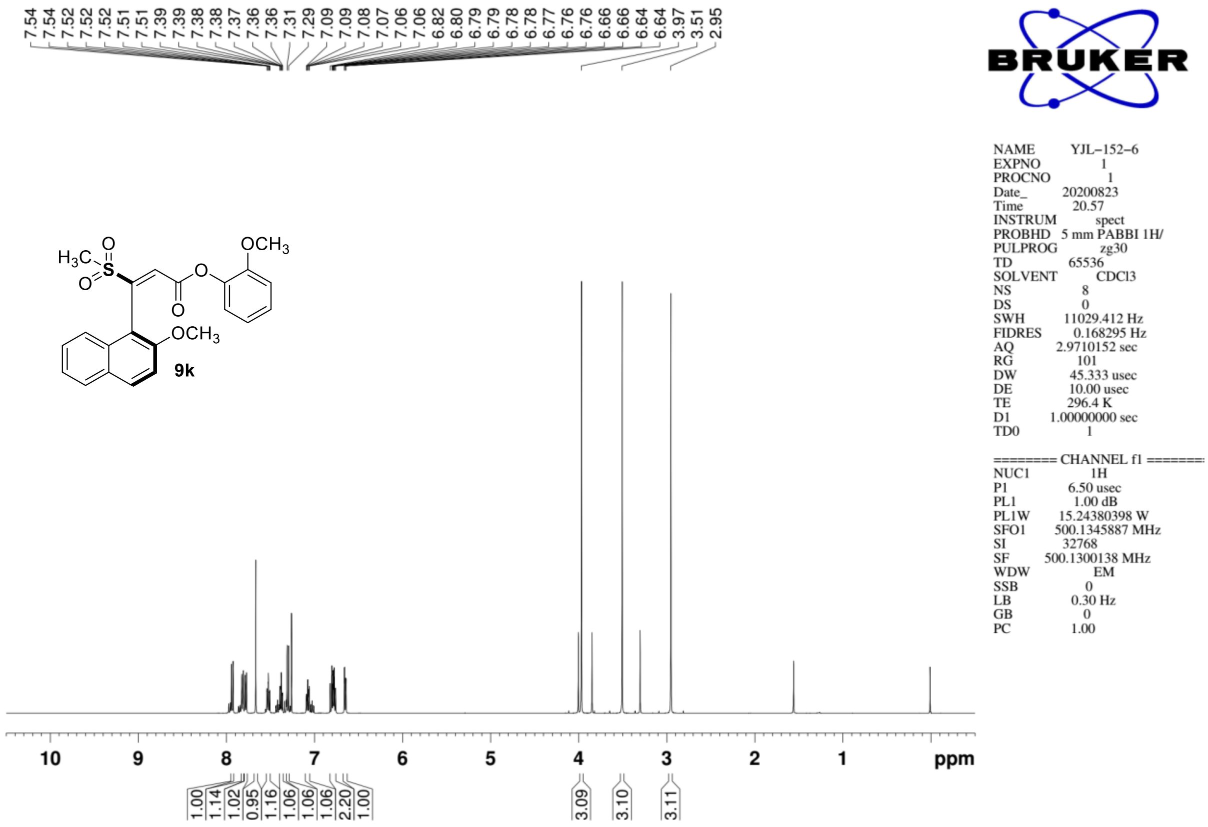

Supplementary Figure 85. ^{1}H NMR ($CDCl_3$, 500MHz) spectra of **9k**

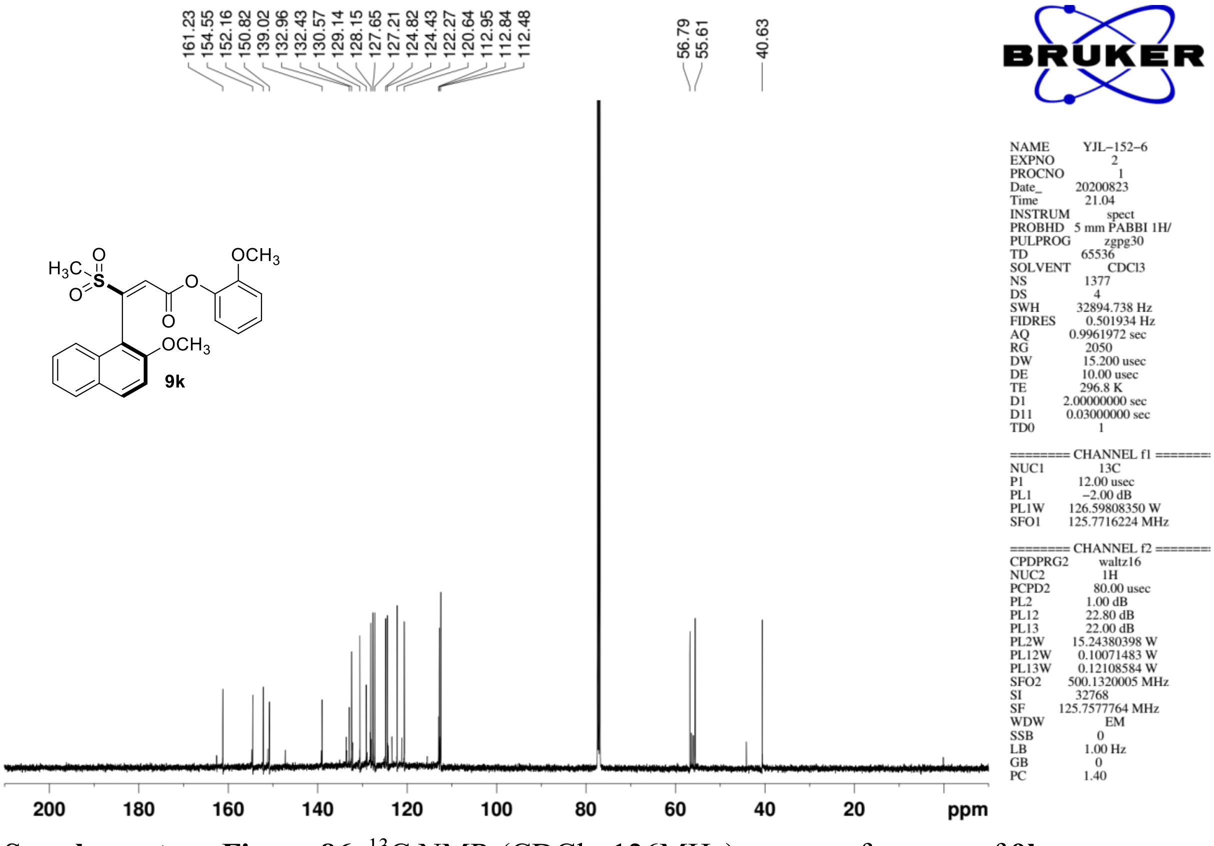

Supplementary Figure 86. ^{13}C NMR ($CDCl_3$, 126MHz) spectra of spectra of **9k**

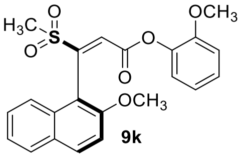

9k

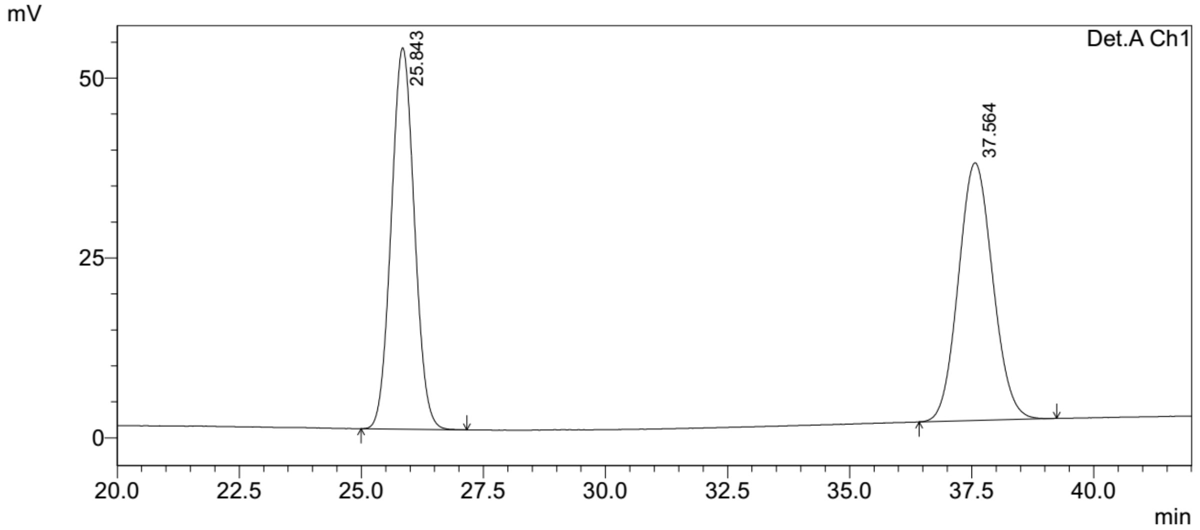

Detector A Ch1 254nm

Peak#	Ret. Time	Area	Height	Area %	Height %
1	25.843	1744272	52996	49.872	59.674
2	37.564	1753253	35813	50.128	40.326
Total		3497525	88809	100.000	100.000

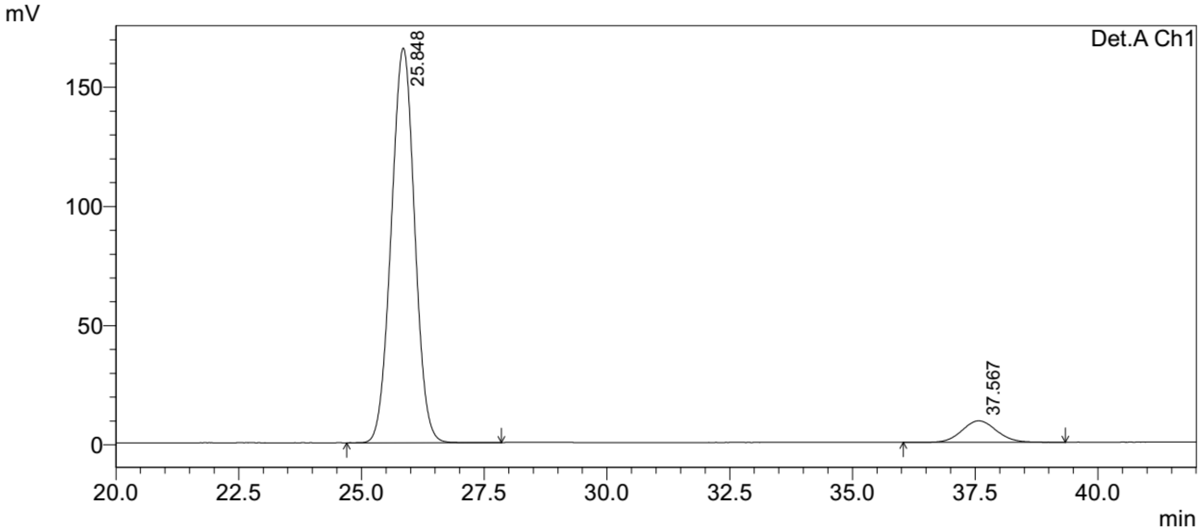

Detector A Ch1 254nm

Peak#	Ret. Time	Area	Height	Area %	Height %
1	25.848	5480392	165669	92.624	94.854
2	37.567	436440	8988	7.376	5.146
Total		5916832	174657	100.000	100.000

Supplementary Figure 87. HPLC spectra of **9k**

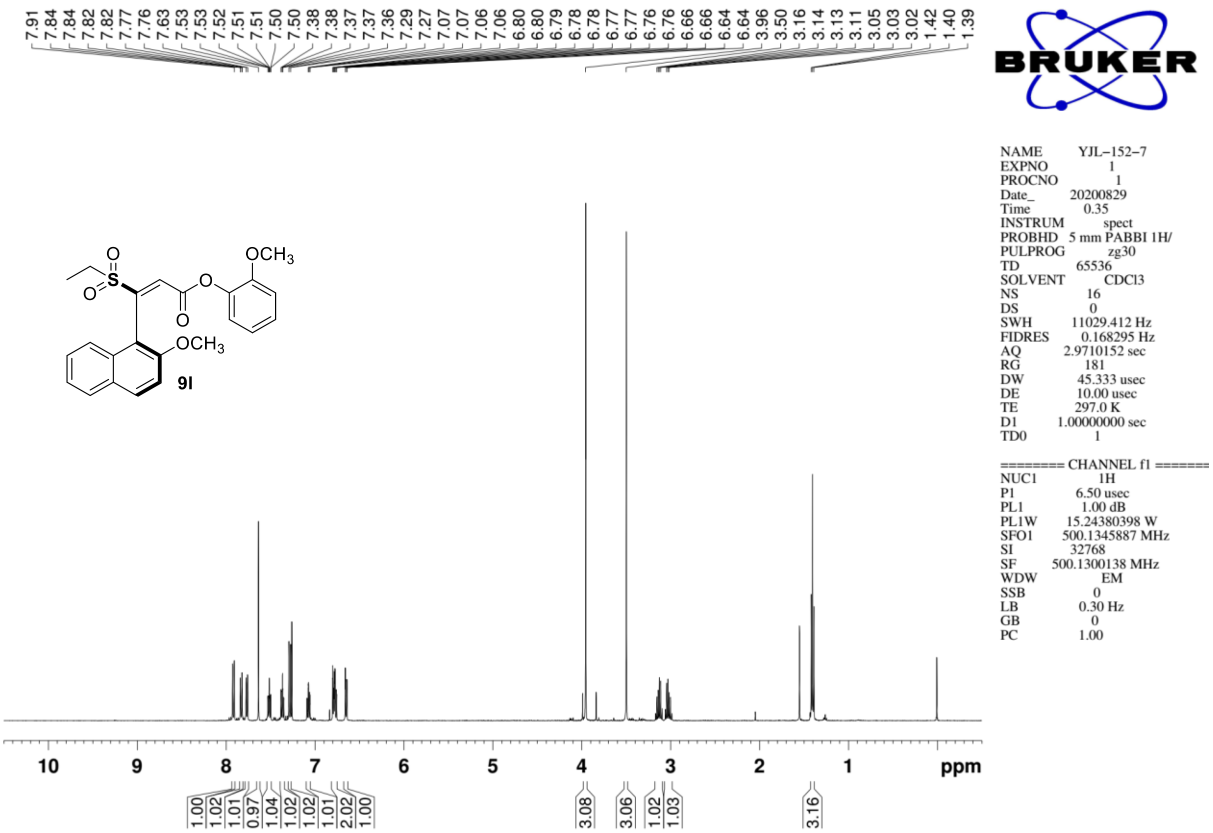

Supplementary Figure 88. ^{1}H NMR ($CDCl_3$, 500MHz) spectra of **9l**

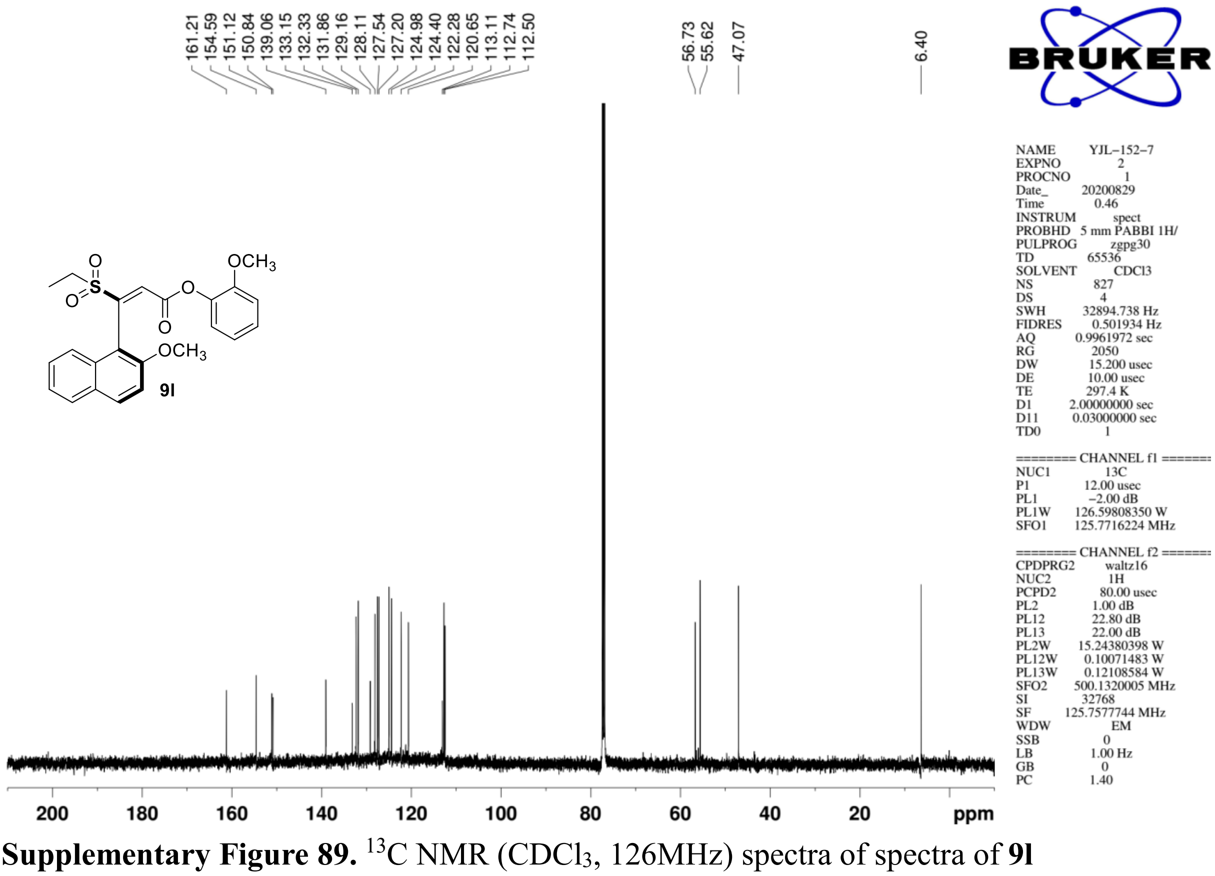

Supplementary Figure 89. ^{13}C NMR ($CDCl_3$, 126MHz) spectra of spectra of **9l**

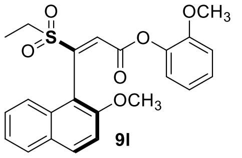

9l

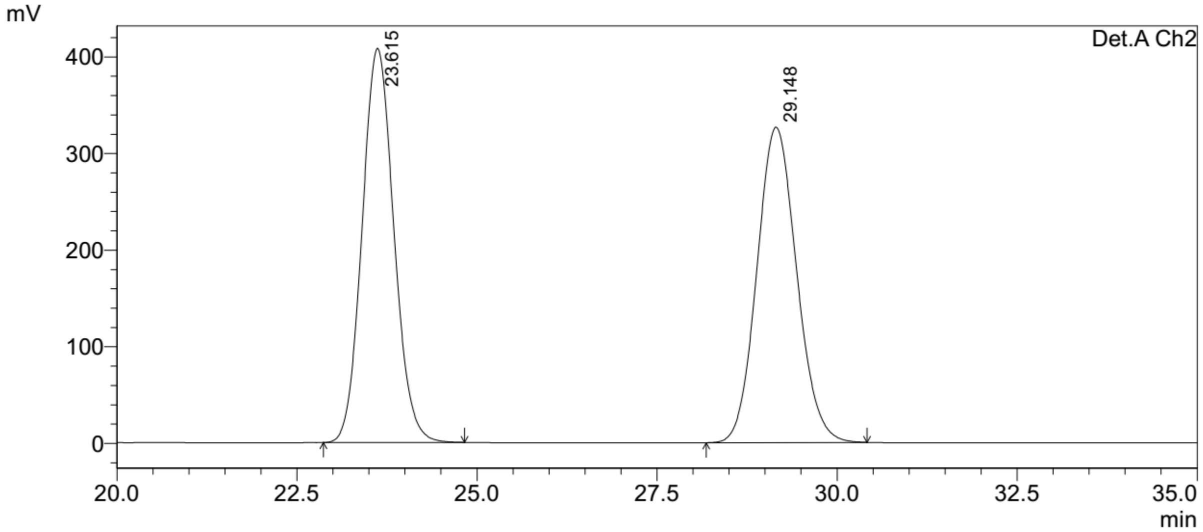

Detector A Ch2 220nm

Peak#	Ret. Time	Area	Height	Area %	Height %
1	23.615	12506338	408124	49.986	55.556
2	29.148	12513358	326488	50.014	44.444
Total		25019696	734611	100.000	100.000

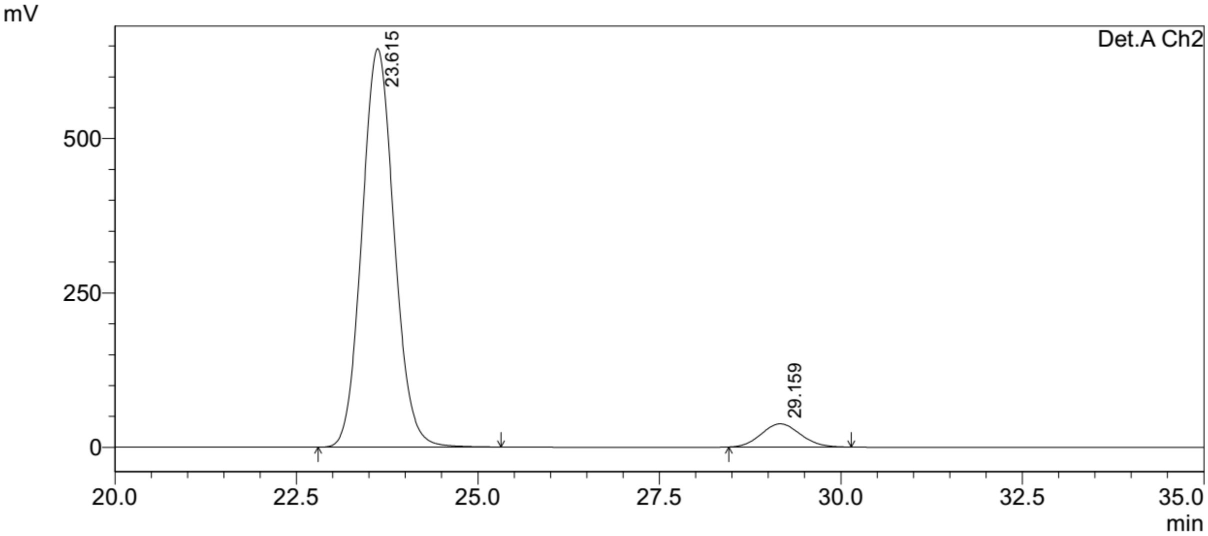

Detector A Ch2 220nm

Peak#	Ret. Time	Area	Height	Area %	Height %
1	23.615	19924807	645557	93.387	94.499
2	29.159	1410930	37582	6.613	5.501
Total		21335737	683139	100.000	100.000

Supplementary Figure 90. HPLC spectra of **9l**

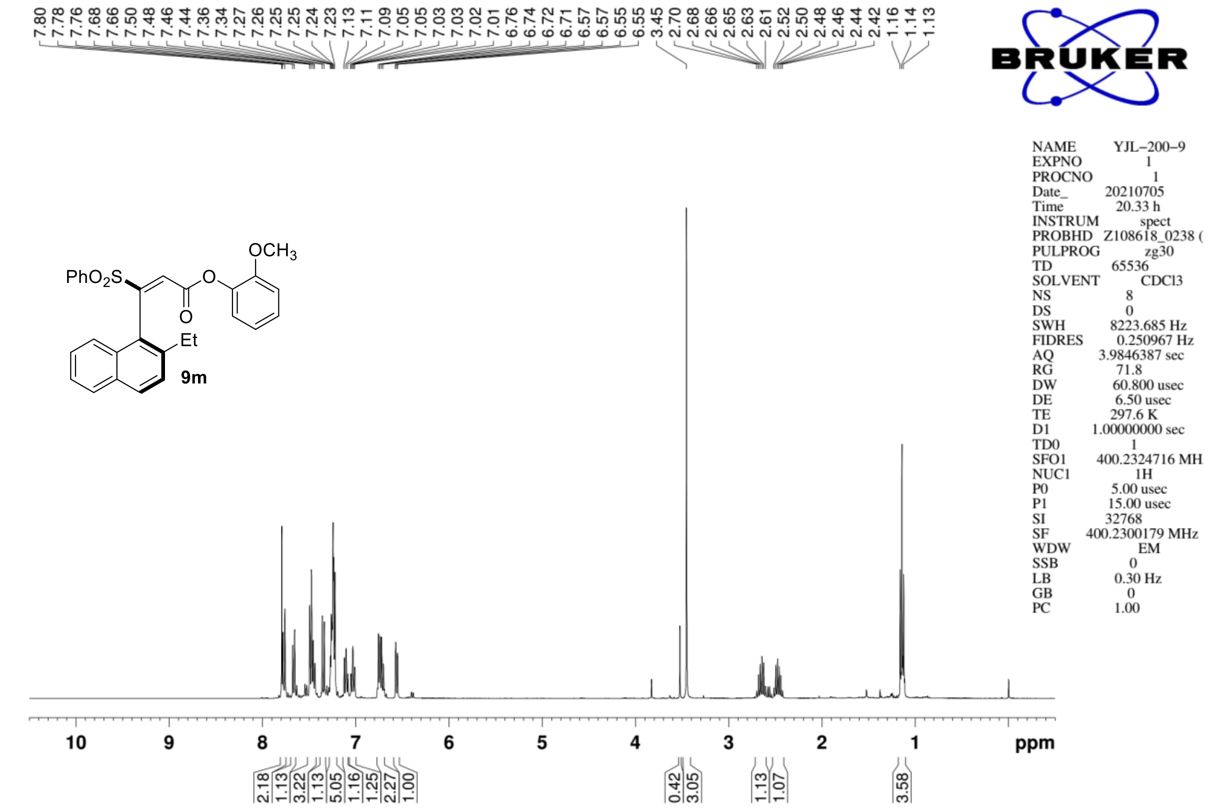

Supplementary Figure 91. ^{1}H NMR ($CDCl_3$, 400MHz) spectra of **9m**

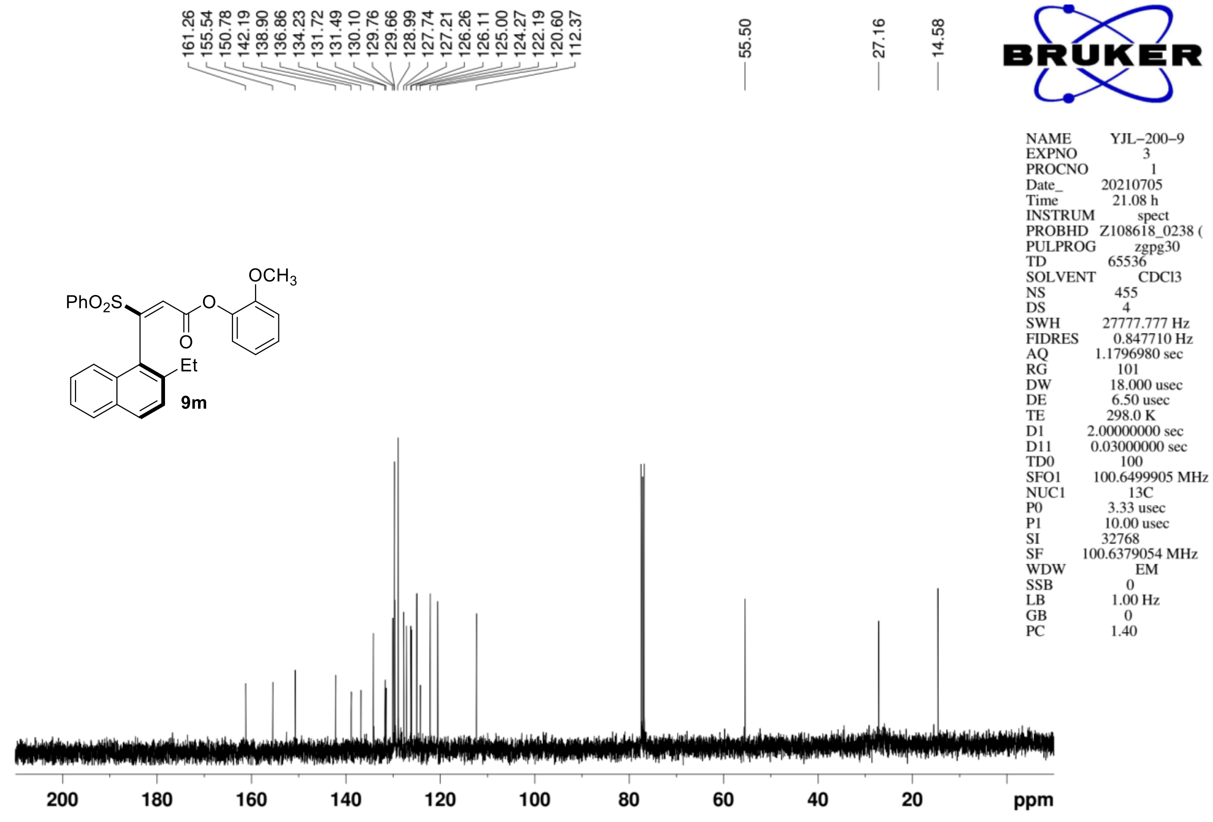

Supplementary Figure 92. ^{13}C NMR ($CDCl_3$, 101MHz) spectra of spectra of **9m**

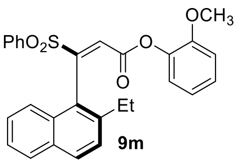

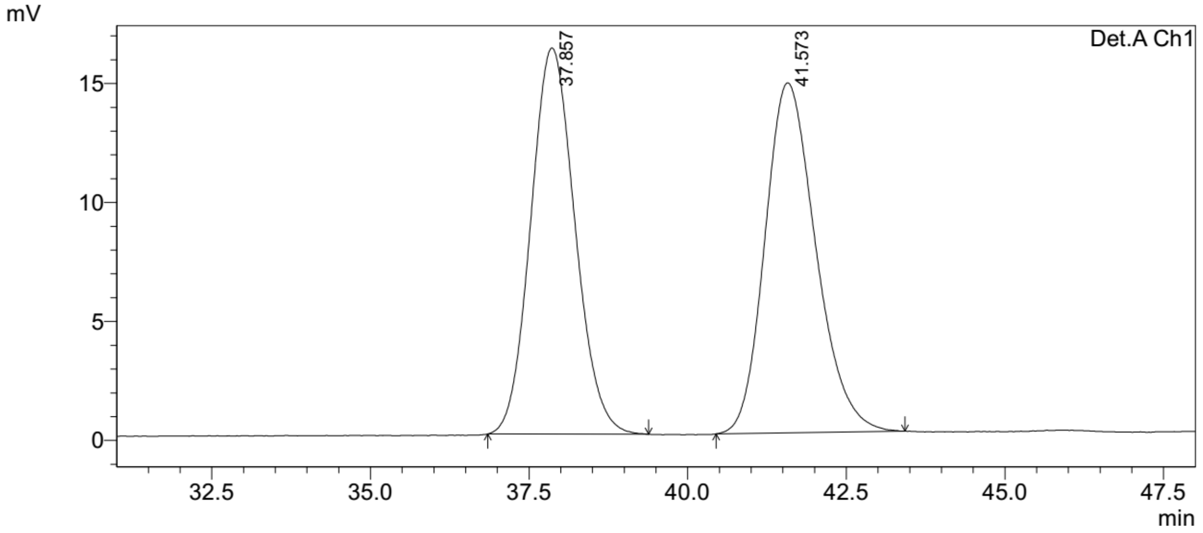

Detector A Ch1 254nm

Peak#	Ret. Time	Area	Height	Area %	Height %
1	37.857	786294	16225	48.980	52.456
2	41.573	819042	14706	51.020	47.544
Total		1605336	30930	100.000	100.000

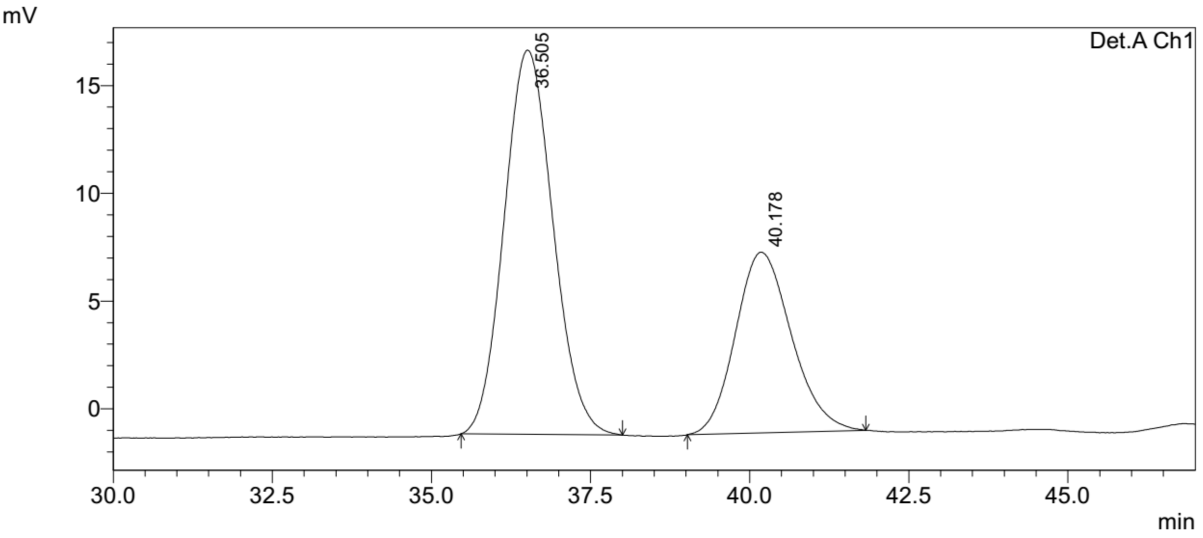

Detector A Ch1 254nm

Peak#	Ret. Time	Area	Height	Area %	Height %
1	36.505	952894	17830	65.025	68.010
2	40.178	512542	8387	34.975	31.990
Total		1465436	26216	100.000	100.000

Supplementary Figure 93. HPLC spectra of **9m**

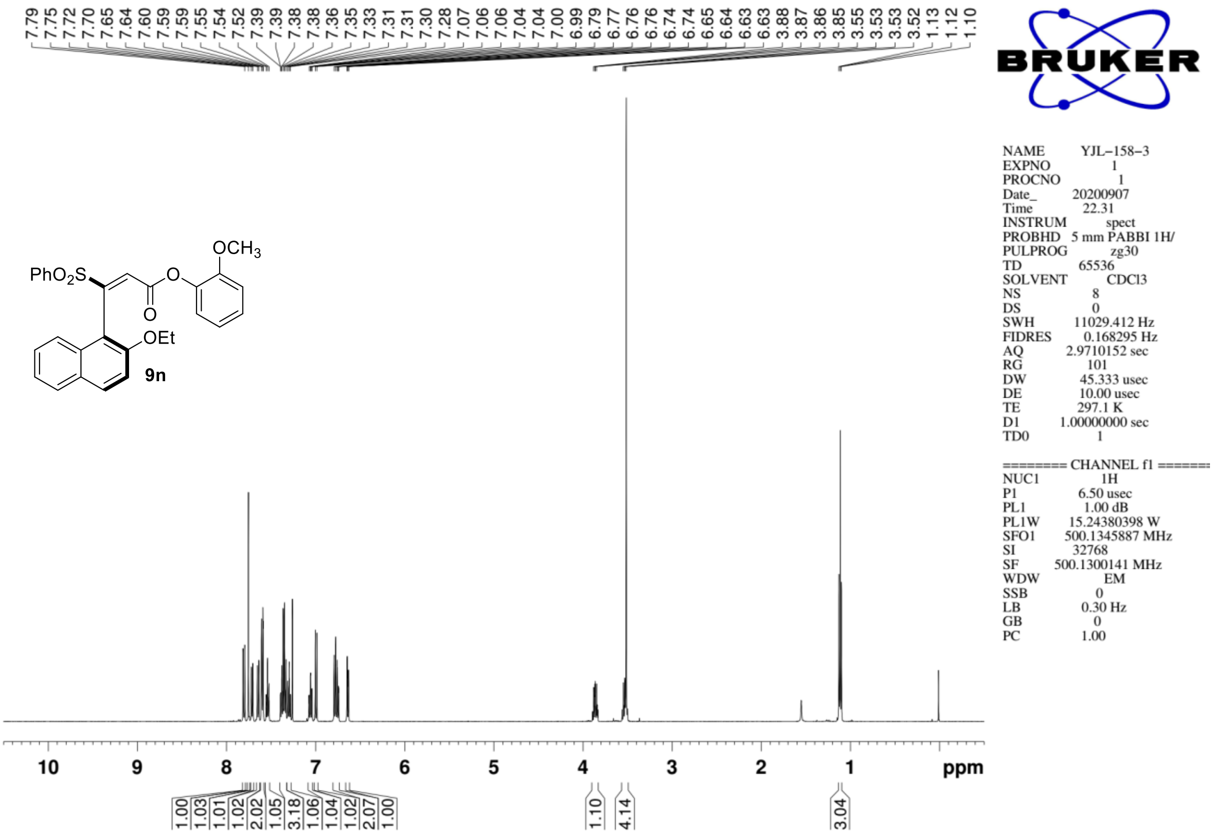

Supplementary Figure 94. ^{1}H NMR ($CDCl_3$, 500MHz) spectra of **9n**

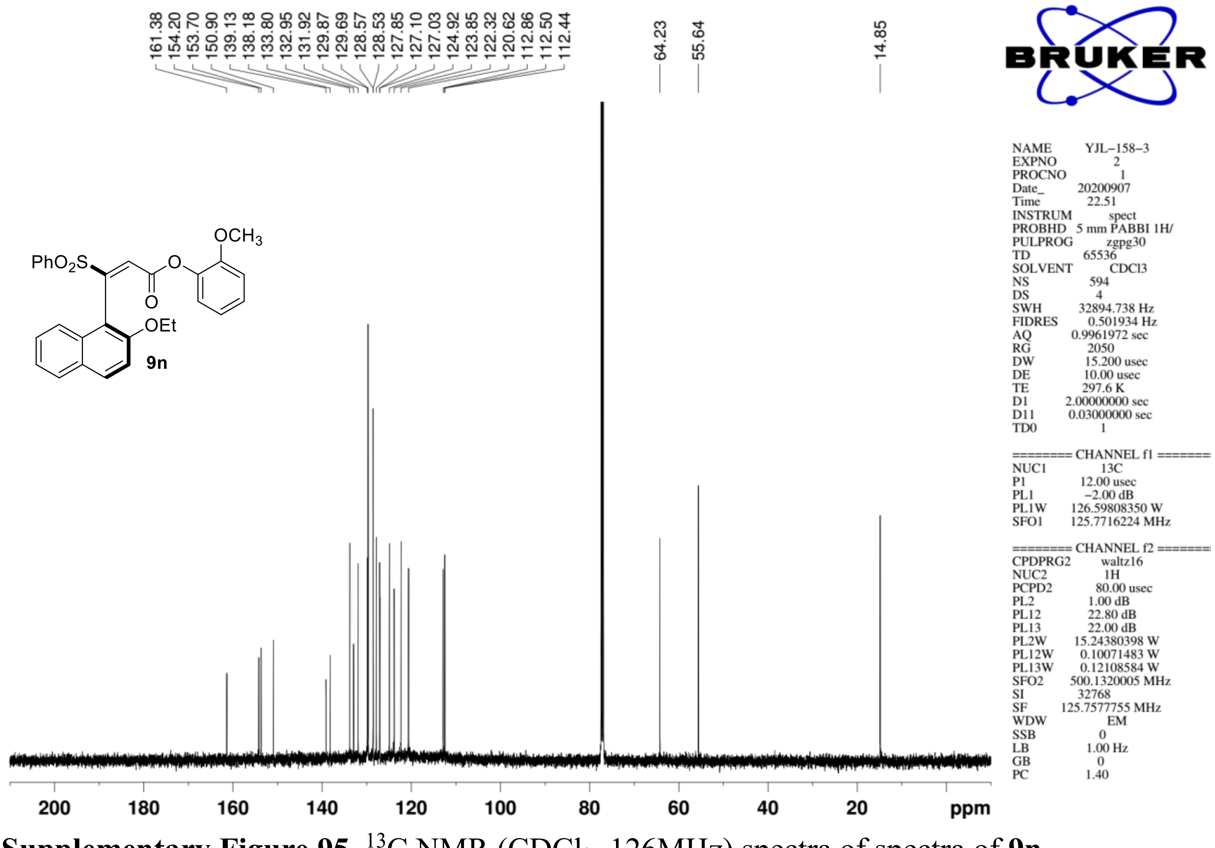

Supplementary Figure 95. ^{13}C NMR ($CDCl_3$, 126MHz) spectra of spectra of **9n**

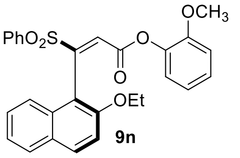

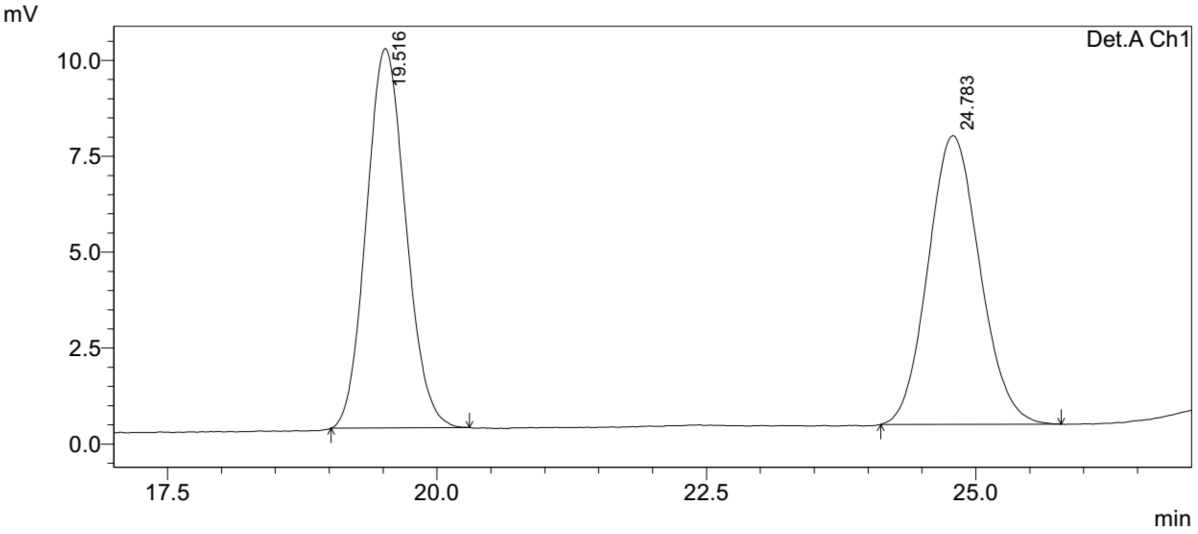

Detector A Ch1 254nm

Peak#	Ret. Time	Area	Height	Area %	Height %
1	19.516	251669	9892	50.026	56.812
2	24.783	251410	7520	49.974	43.188
Total		503079	17412	100.000	100.000

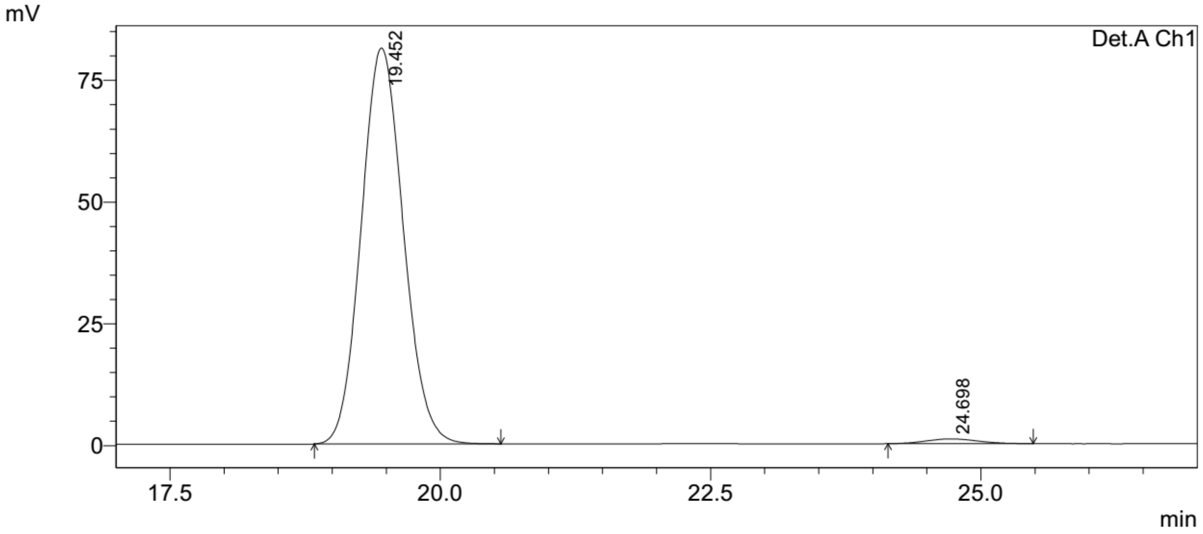

Detector A Ch1 254nm

Peak#	Ret. Time	Area	Height	Area %	Height %
1	19.452	2203563	81299	98.528	98.803
2	24.698	32915	985	1.472	1.197
Total		2236478	82284	100.000	100.000

Supplementary Figure 96. HPLC spectra of **9n**

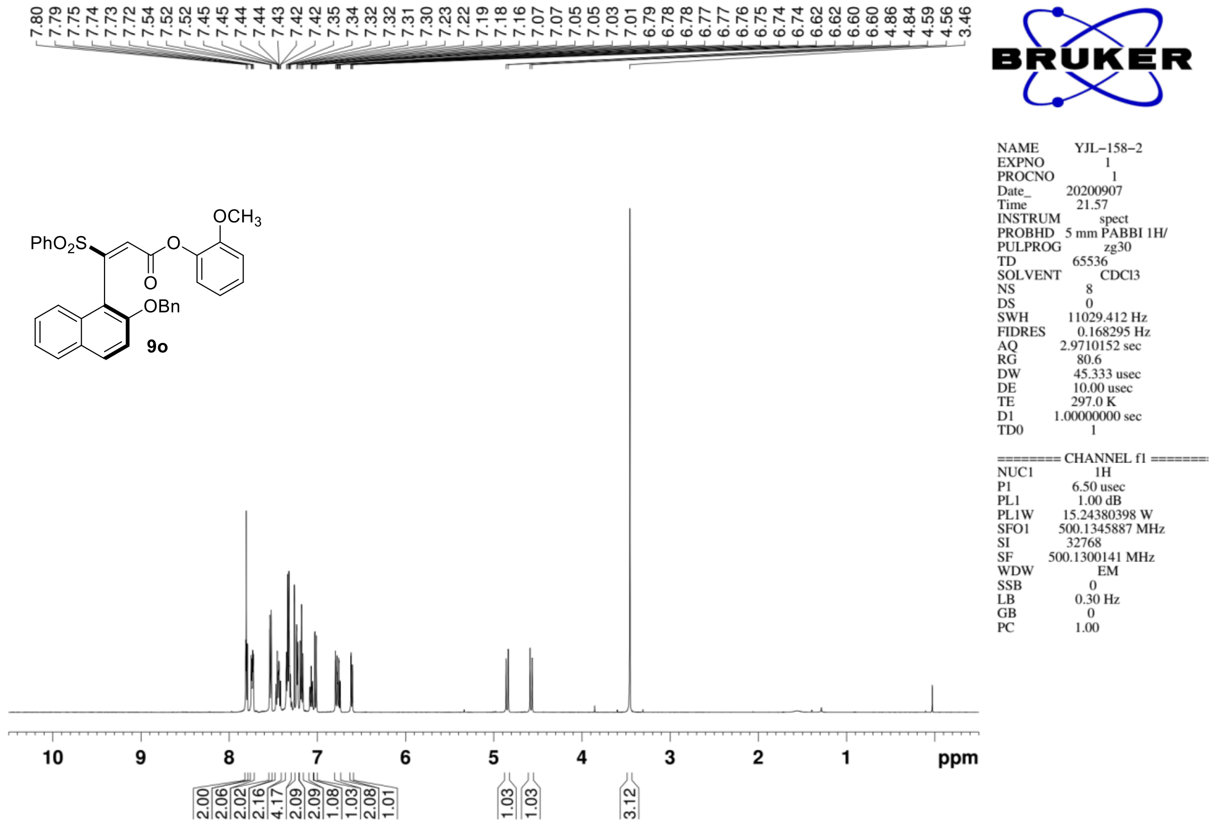

Supplementary Figure 97. ^{1}H NMR ($CDCl_3$, 500MHz) spectra of **9o**

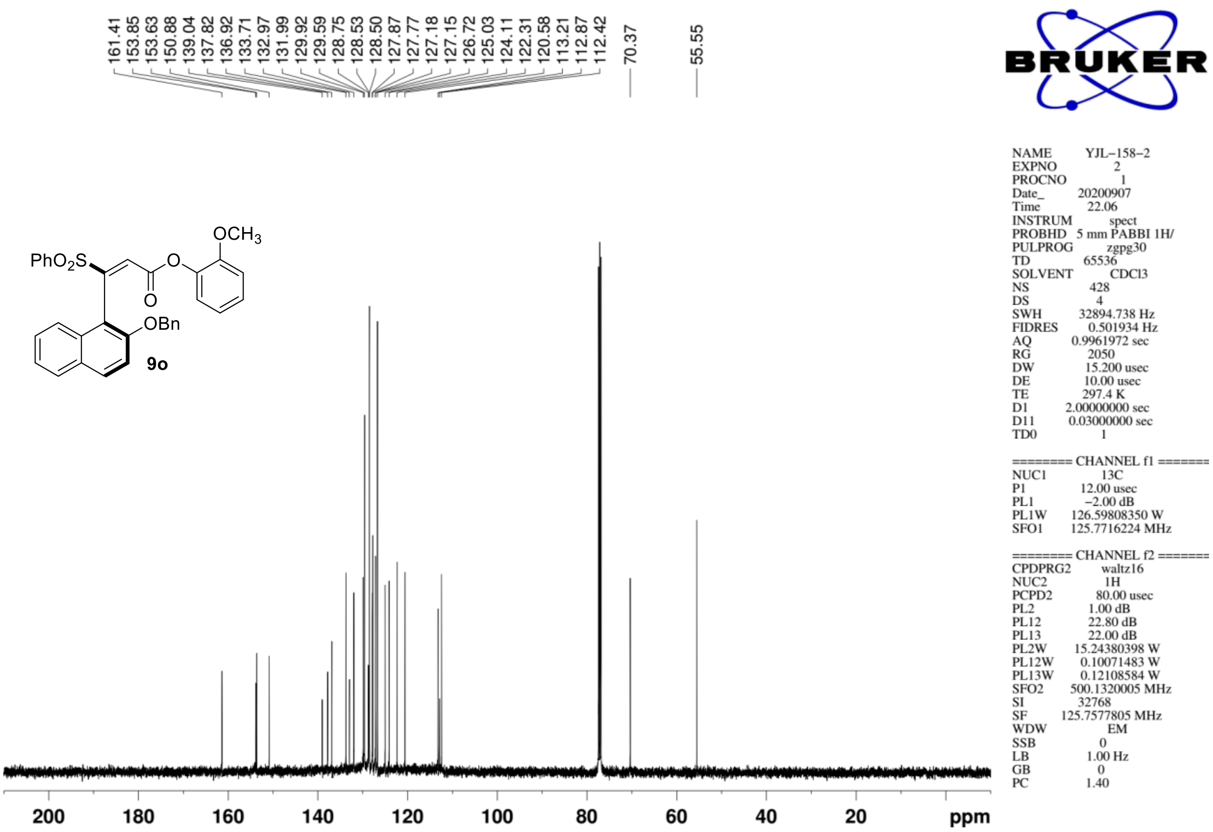

Supplementary Figure 98. ^{13}C NMR ($CDCl_3$, 126MHz) spectra of spectra of **9o**

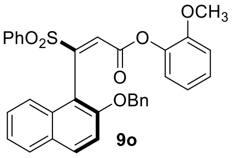

9o

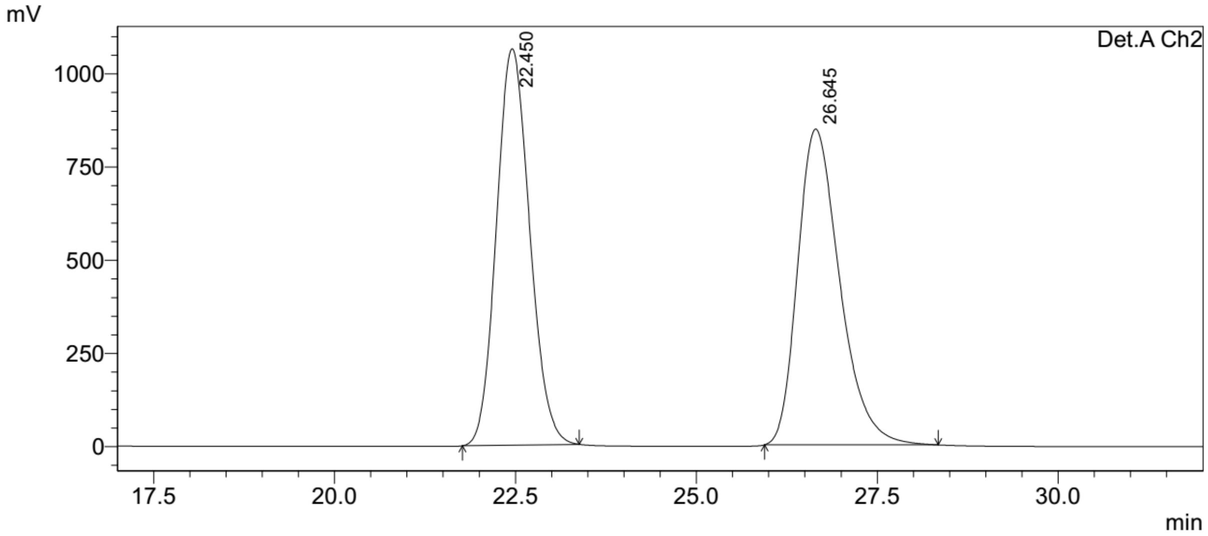

Detector A Ch2 220nm

Peak#	Ret. Time	Area	Height	Area %	Height %
1	22.450	33998937	1063024	49.760	55.638
2	26.645	34327244	847581	50.240	44.362
Total		68326181	1910604	100.000	100.000

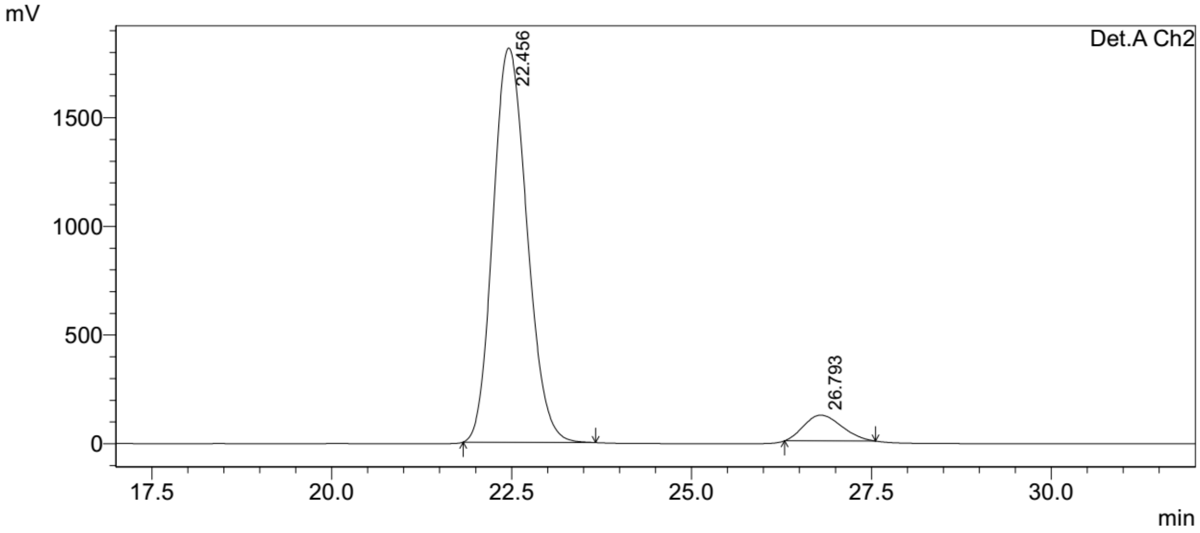

Detector A Ch2 220nm

Peak#	Ret. Time	Area	Height	Area %	Height %
1	22.456	59541165	1814047	93.192	93.859
2	26.793	4349762	118695	6.808	6.141
Total		63890927	1932742	100.000	100.000

Supplementary Figure 99. HPLC spectra of **9o**

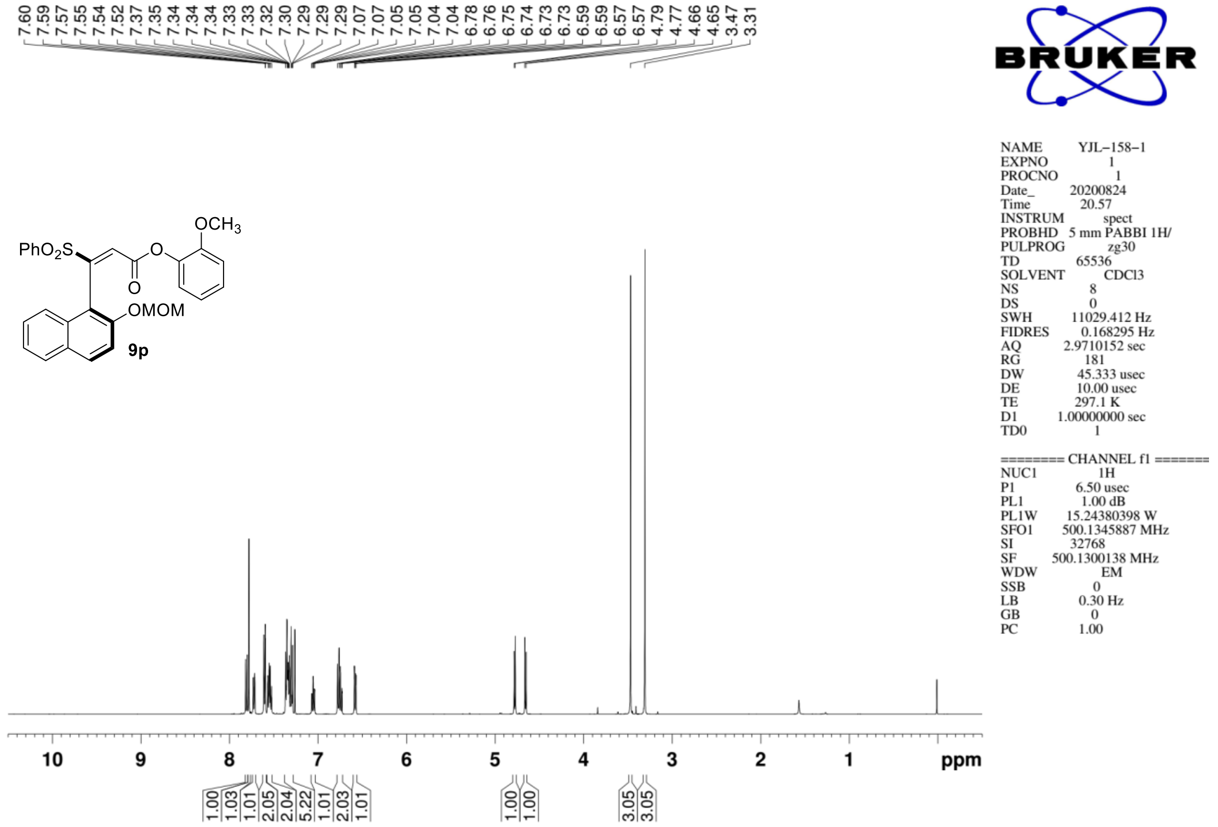

Supplementary Figure 100. ^{1}H NMR ($CDCl_3$, 500MHz) spectra of **9p**

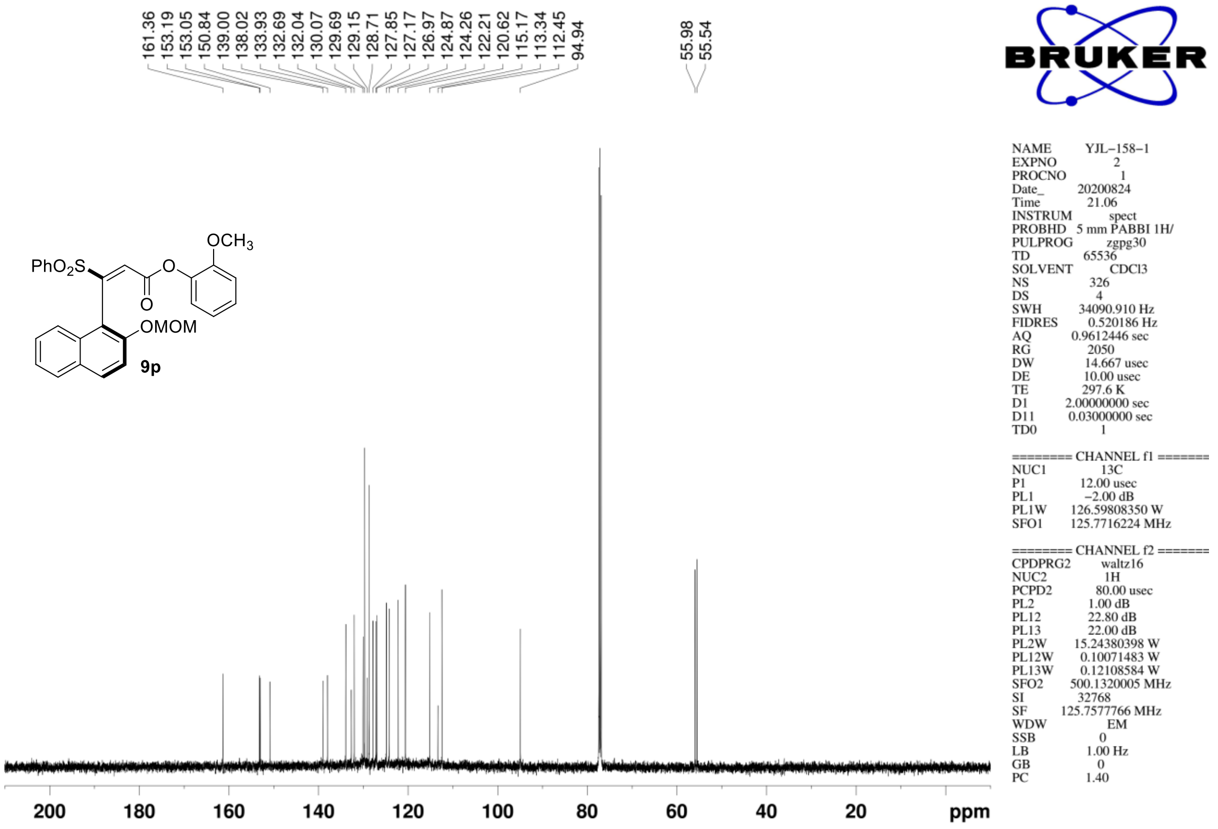

Supplementary Figure 101. ^{13}C NMR ($CDCl_3$, 126MHz) spectra of spectra of **9p**

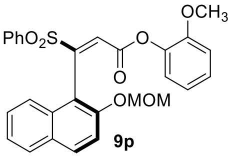

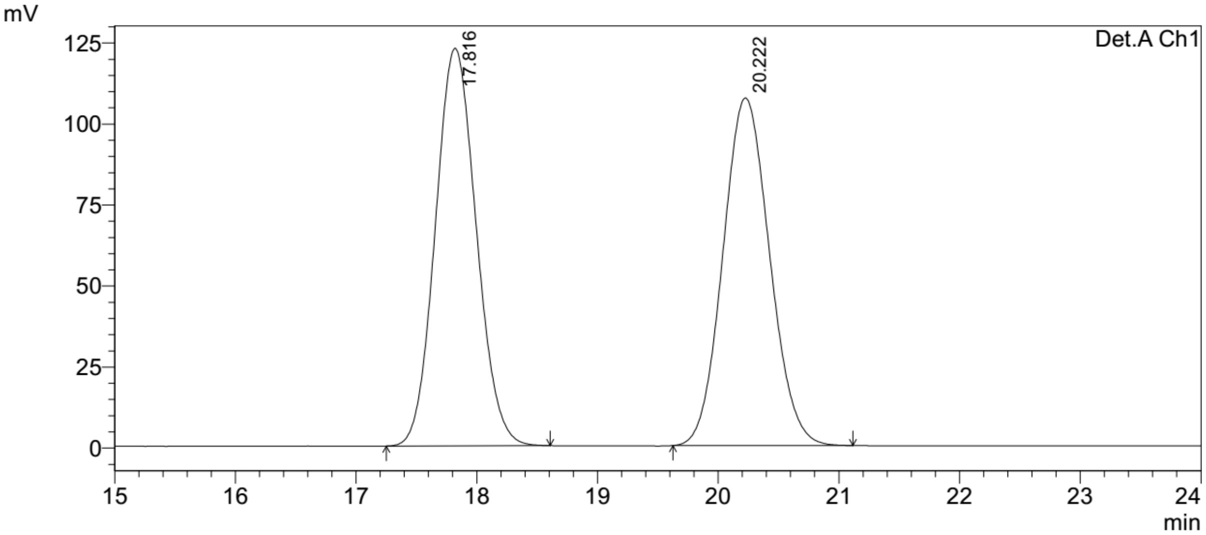

Detector A Ch1 254nm

Peak#	Ret. Time	Area	Height	Area %	Height %
1	17.816	2891714	122692	50.063	53.351
2	20.222	2884426	107279	49.937	46.649
Total		5776141	229972	100.000	100.000

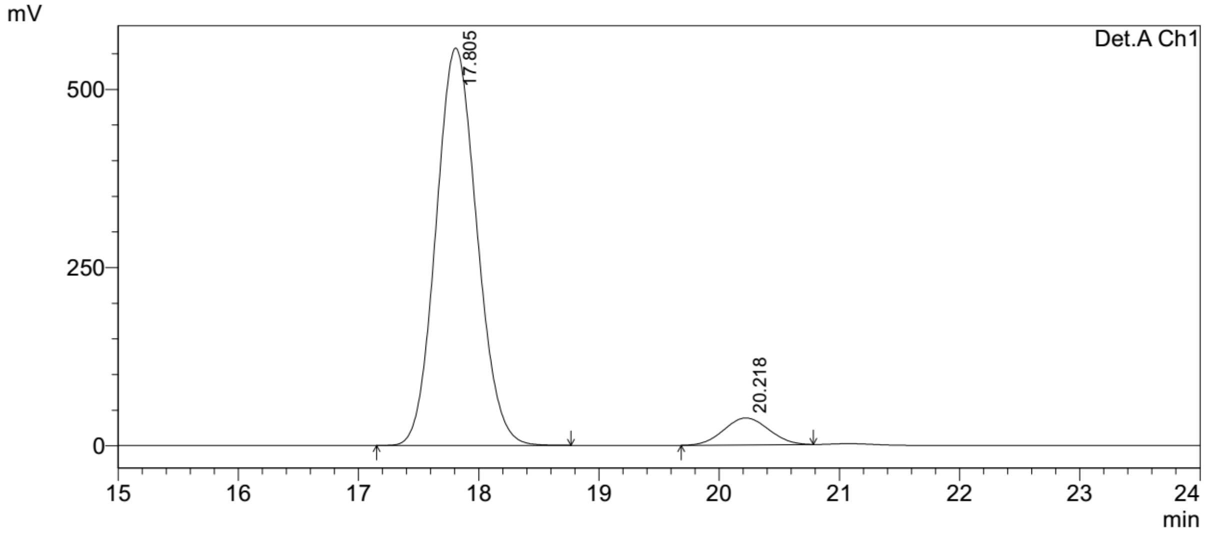

Detector A Ch1 254nm

Peak#	Ret. Time	Area	Height	Area %	Height %
1	17.805	13167744	557539	93.060	93.648
2	20.218	981976	37820	6.940	6.352
Total		14149720	595360	100.000	100.000

Supplementary Figure 102. HPLC spectra of **9p**

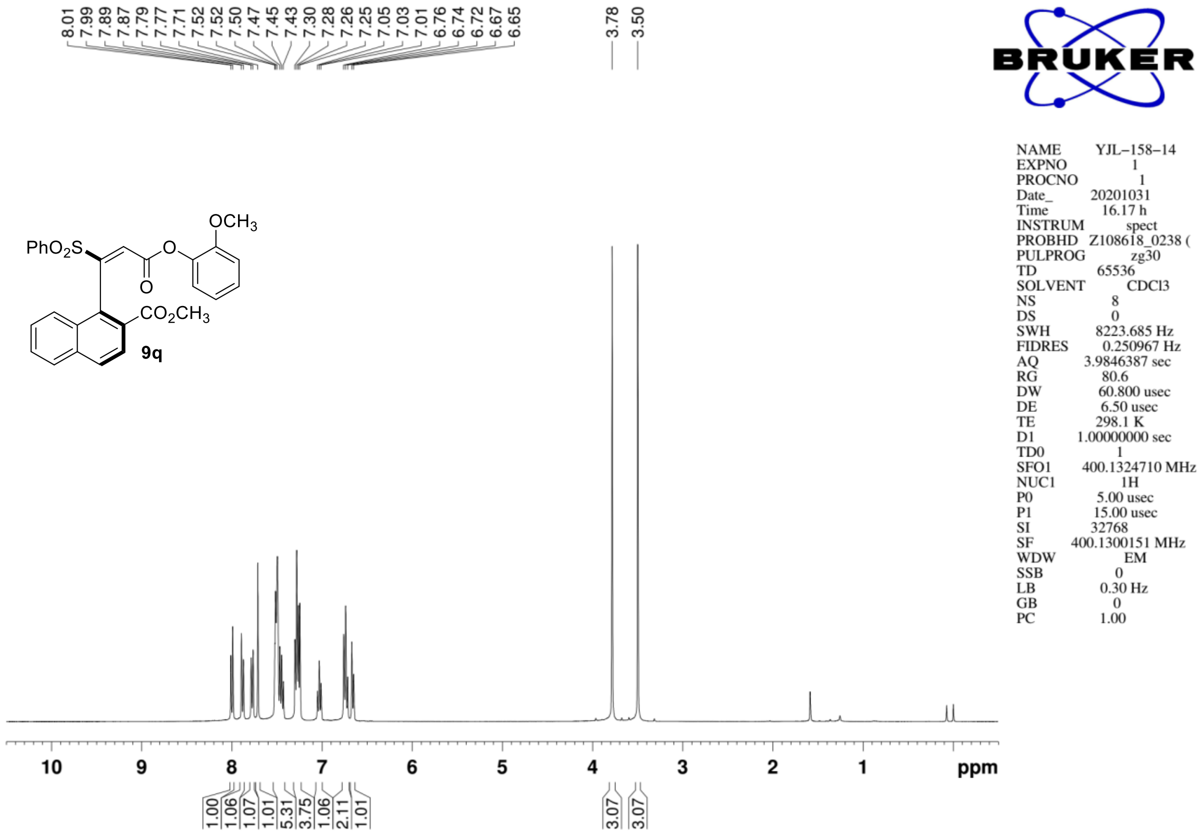

Supplementary Figure 103. ^{1}H NMR ($CDCl_3$, 400MHz) spectra of **9q**

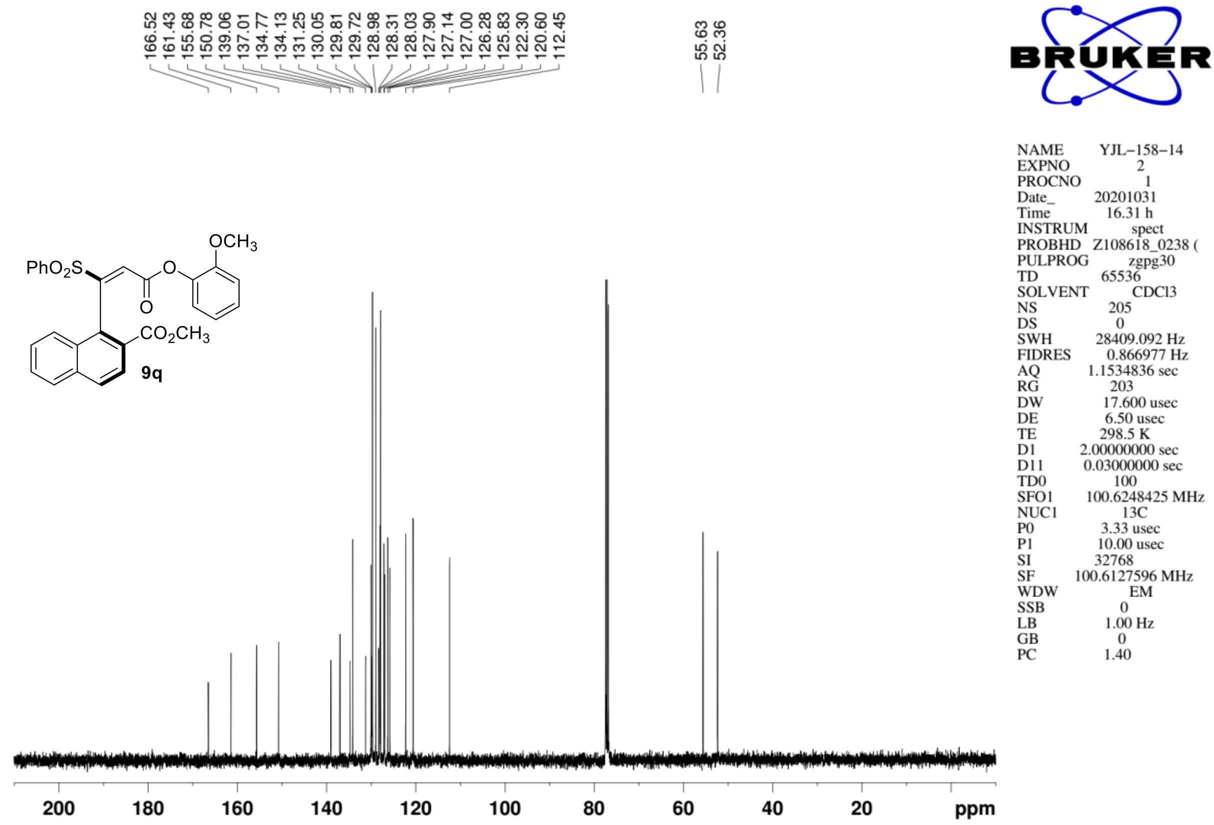

Supplementary Figure 104. ^{13}C NMR ($CDCl_3$, 101MHz) spectra of spectra of **9q**

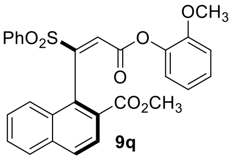

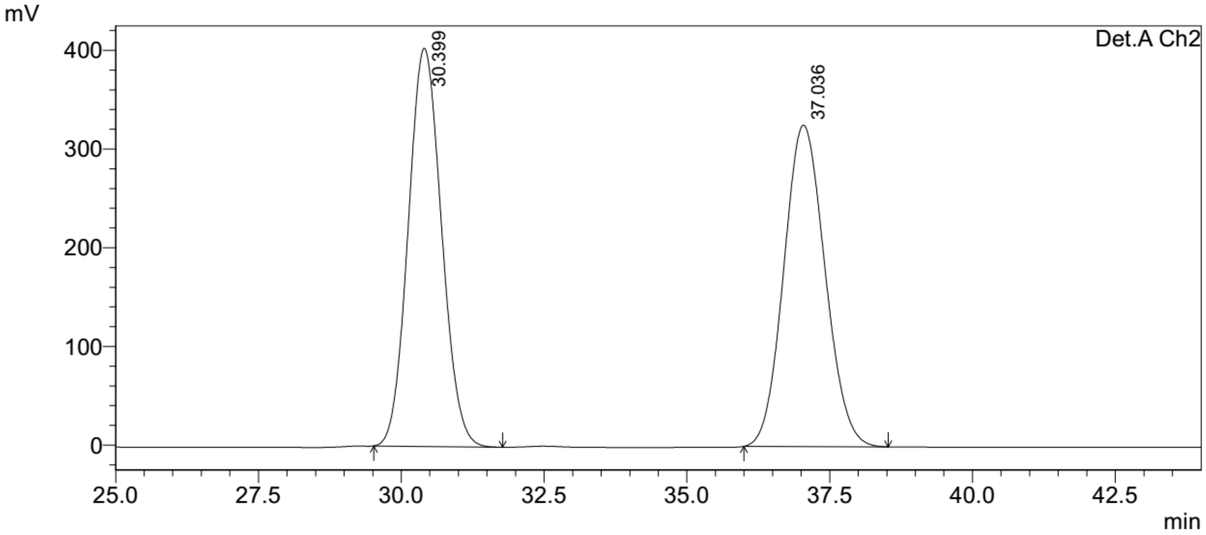

Detector A Ch2 220nm

Peak#	Ret. Time	Area	Height	Area %	Height %
1	30.399	16364476	403454	50.041	55.333
2	37.036	16337574	325687	49.959	44.667
Total		32702050	729141	100.000	100.000

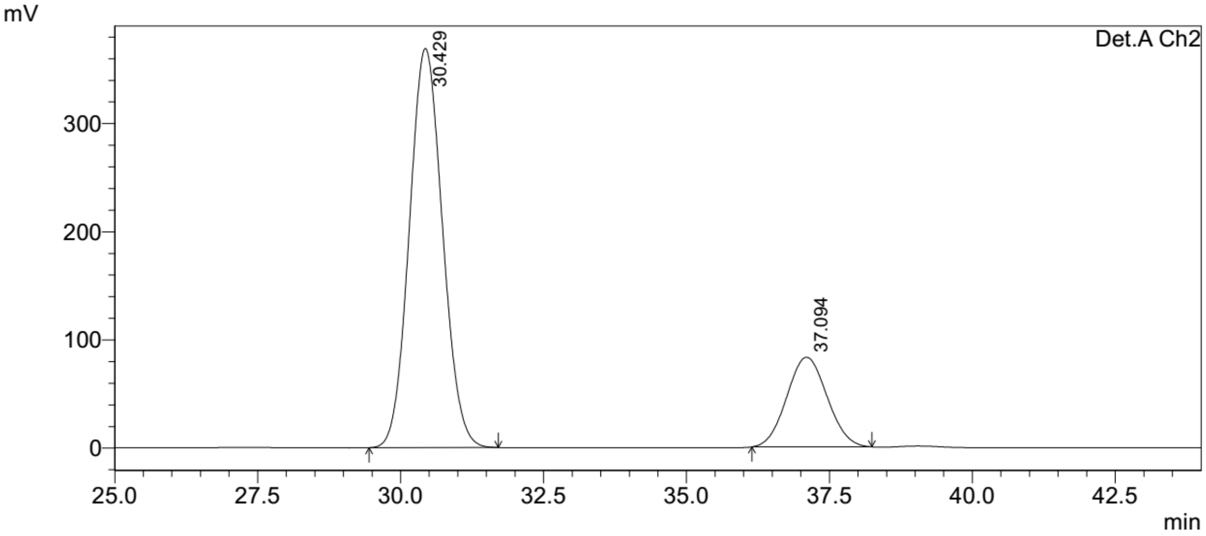

Detector A Ch2 220nm

Peak#	Ret. Time	Area	Height	Area %	Height %
1	30.429	14892122	369052	78.537	81.656
2	37.094	4069828	82906	21.463	18.344
Total		18961951	451957	100.000	100.000

Supplementary Figure 105. HPLC spectra of **9q**

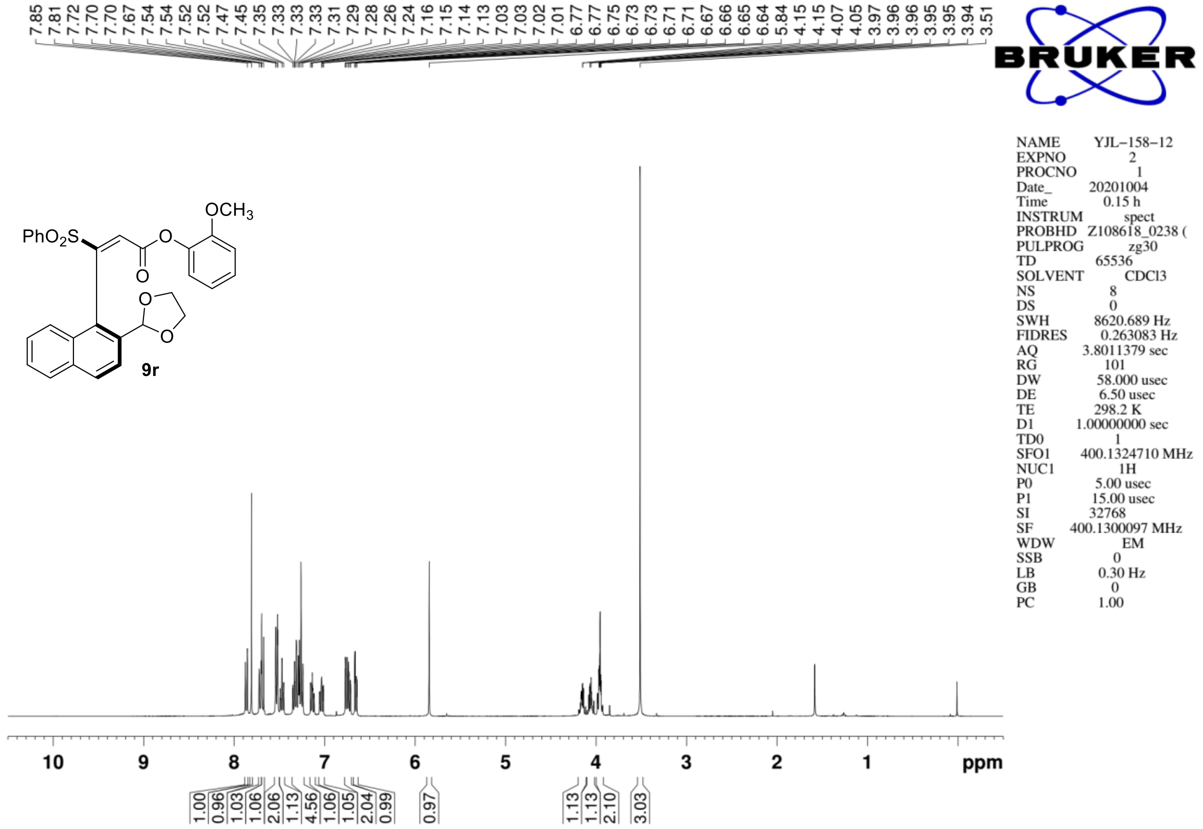

Supplementary Figure 106. ^{1}H NMR ($CDCl_3$, 400MHz) spectra of **9r**

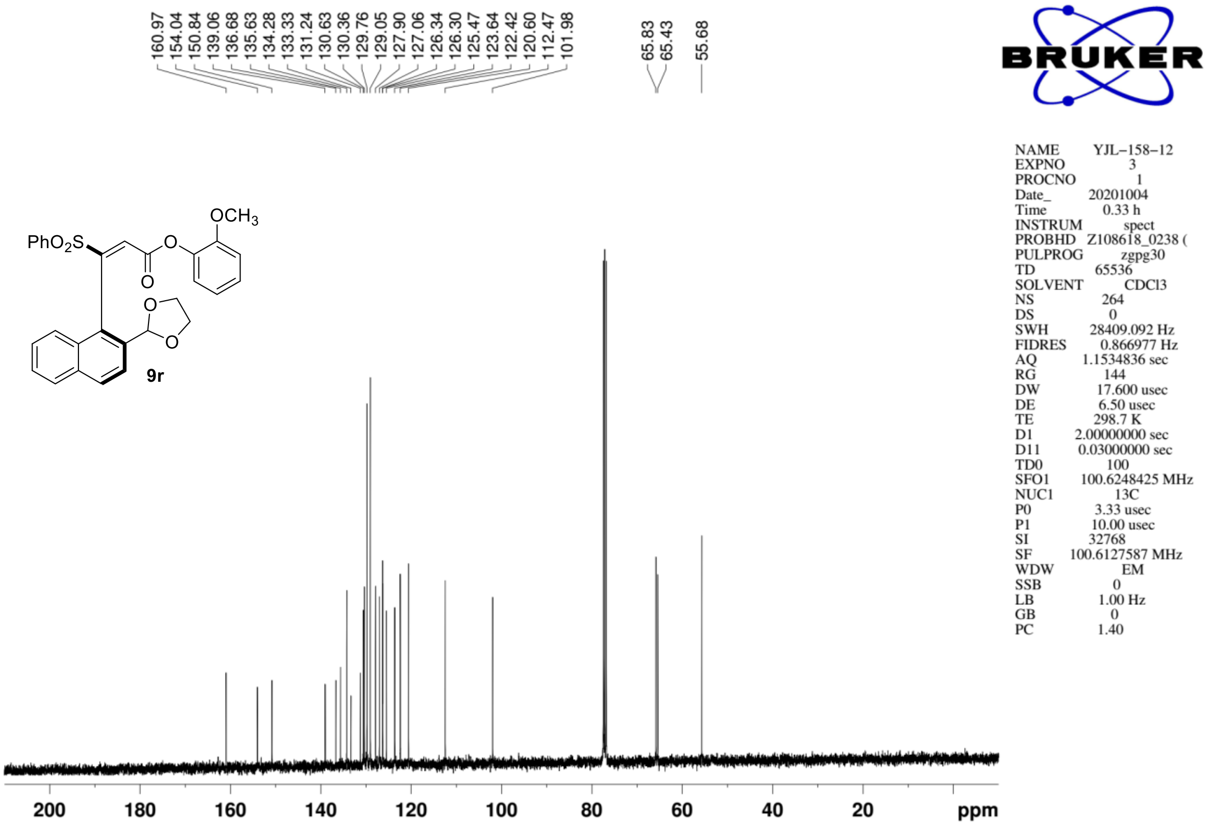

Supplementary Figure 107. ^{13}C NMR ($CDCl_3$, 101MHz) spectra of spectra of **9r**

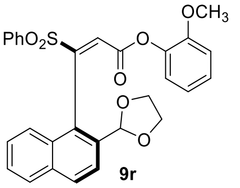

9r

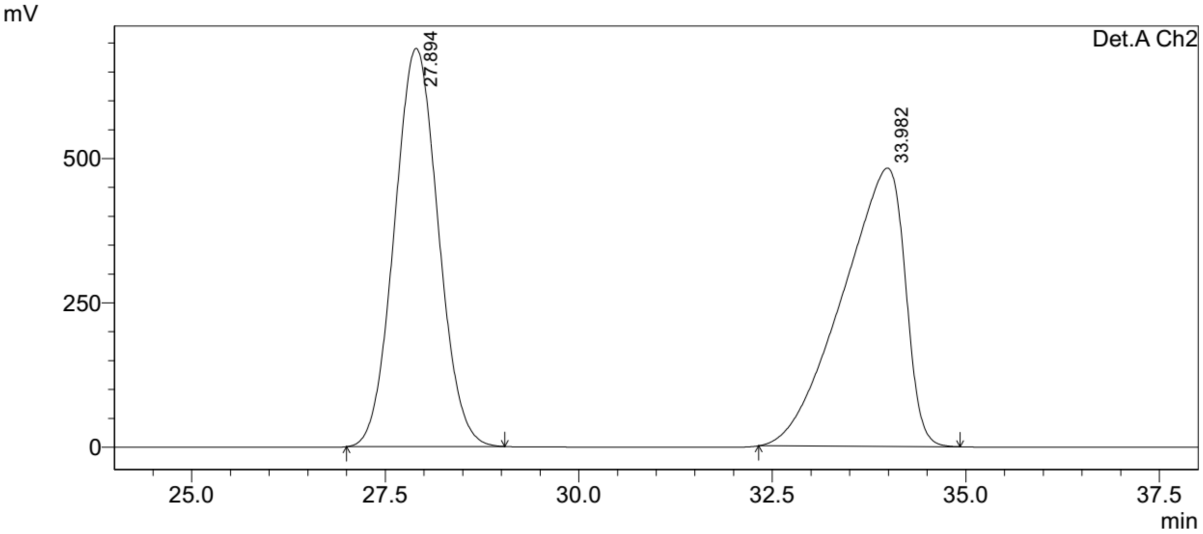

Detector A Ch2 220nm

Peak#	Ret. Time	Area	Height	Area %	Height %
1	27.894	27233101	689899	50.021	58.855
2	33.982	27210334	482293	49.979	41.145
Total		54443435	1172192	100.000	100.000

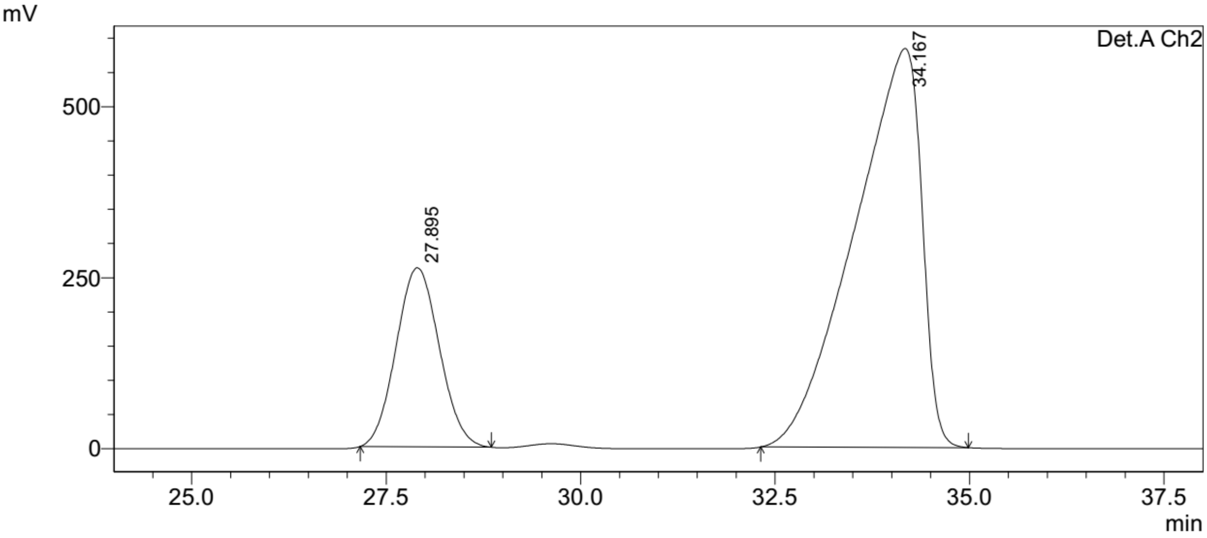

Detector A Ch2 220nm

Peak#	Ret. Time	Area	Height	Area %	Height %
1	27.895	10126896	261706	22.383	30.978
2	34.167	35115788	583105	77.617	69.022
Total		45242684	844811	100.000	100.000

Supplementary Figure 108. HPLC spectra of **9r**

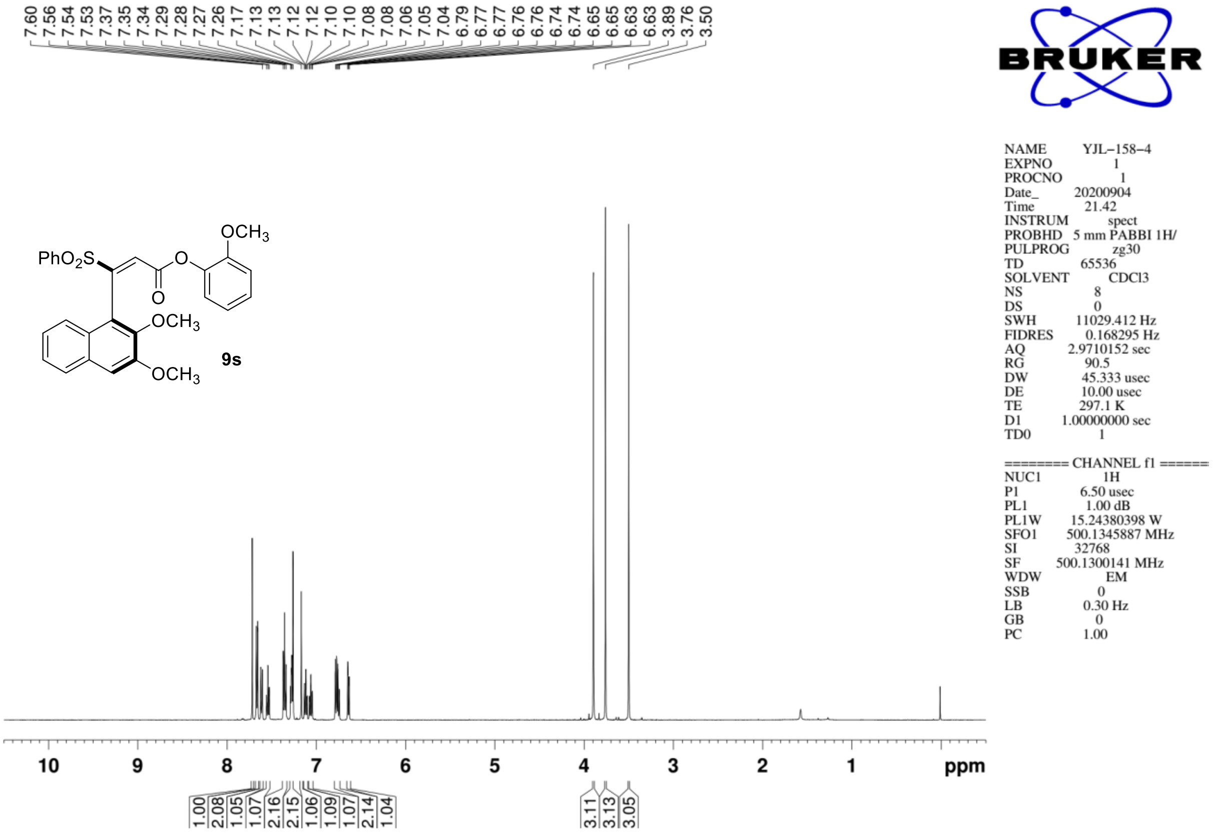

Supplementary Figure 109. ^{1}H NMR ($CDCl_3$, 500MHz) spectra of **9s**

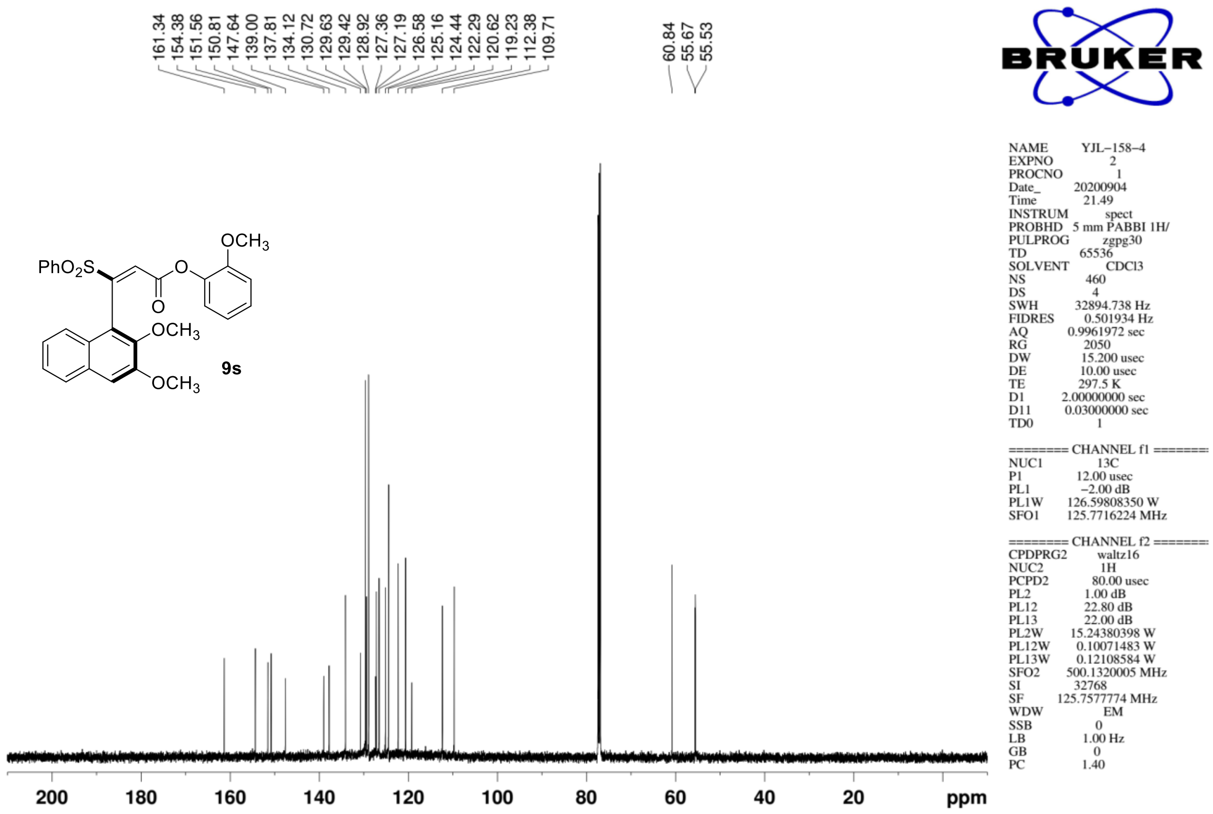

Supplementary Figure 110. ^{13}C NMR ($CDCl_3$, 126MHz) spectra of spectra of **9s**

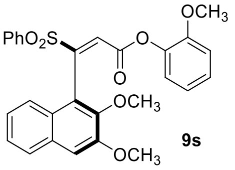

9s

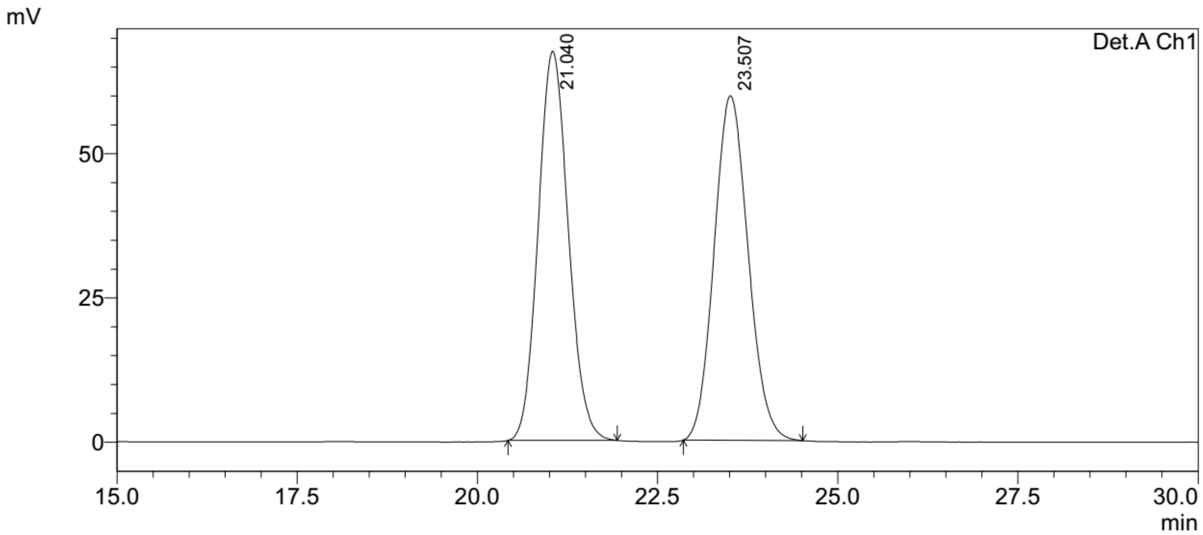

Detector A Ch1 254nm

Peak#	Ret. Time	Area	Height	Area %	Height %
1	21.040	1962116	67467	50.024	53.063
2	23.507	1960221	59678	49.976	46.937
Total		3922337	127145	100.000	100.000

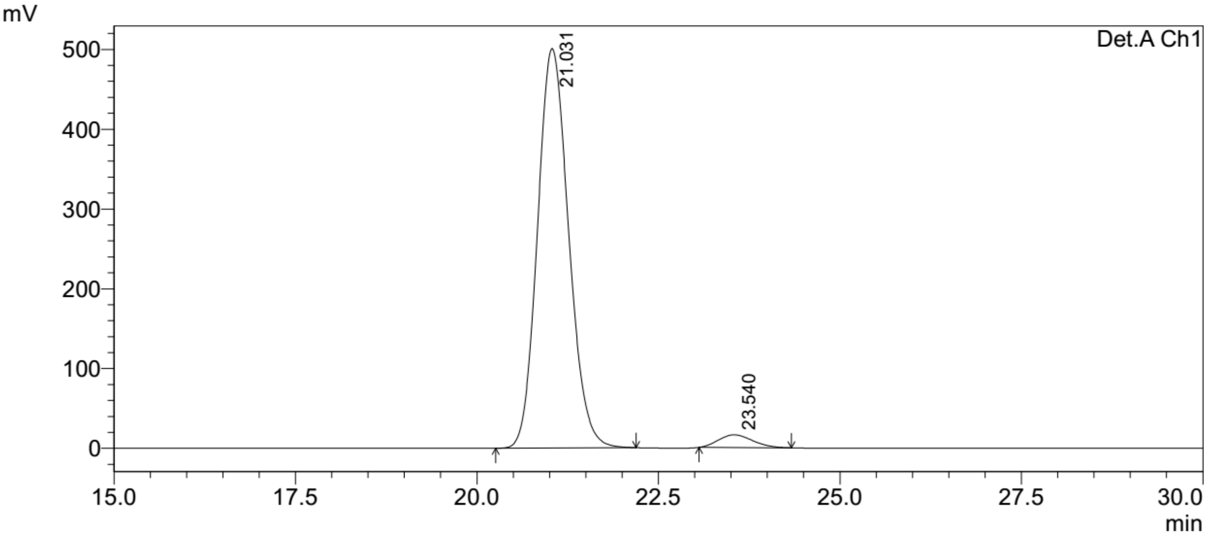

Detector A Ch1 254nm

Peak#	Ret. Time	Area	Height	Area %	Height %
1	21.031	14924203	500842	96.640	96.923
2	23.540	518837	15902	3.360	3.077
Total		15443040	516743	100.000	100.000

Supplementary Figure 111. HPLC spectra of **9s**

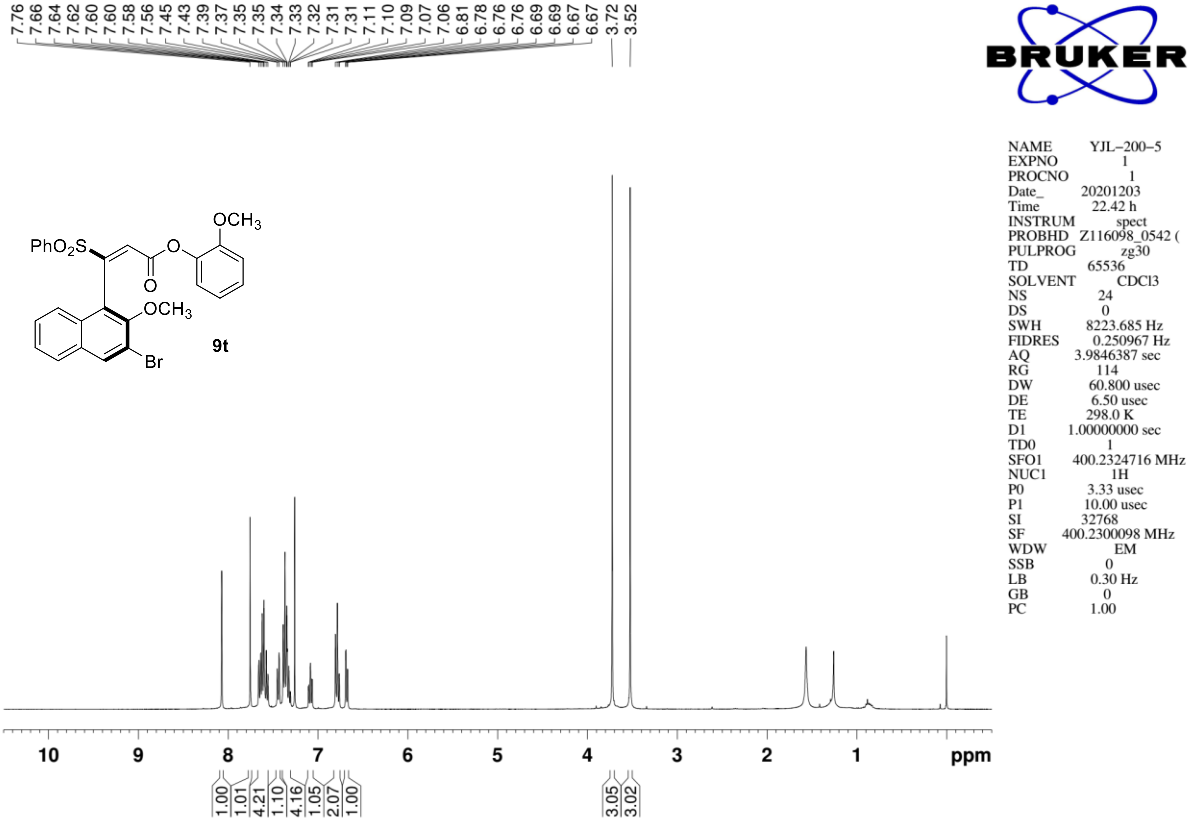

Supplementary Figure 112. ^{1}H NMR ($CDCl_3$, 400MHz) spectra of **9t**

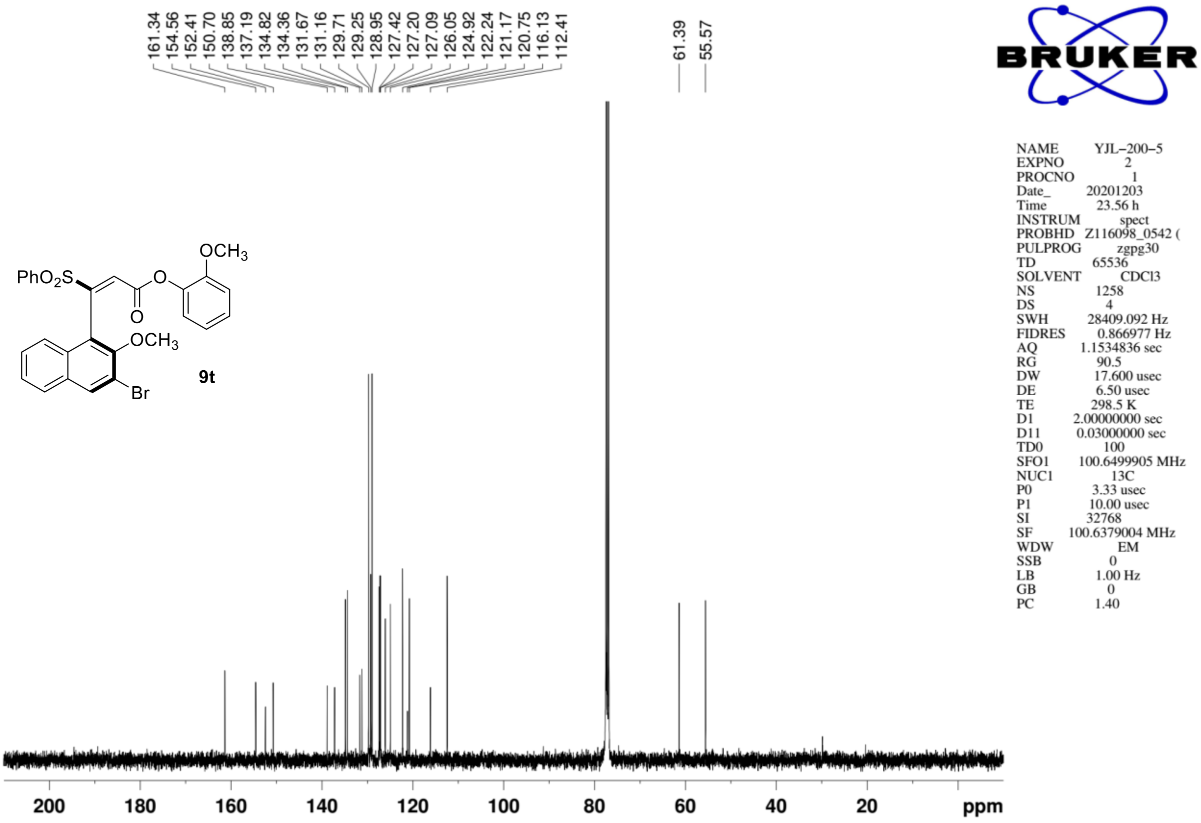

Supplementary Figure 113. ^{13}C NMR ($CDCl_3$, 101MHz) spectra of spectra of **9t**

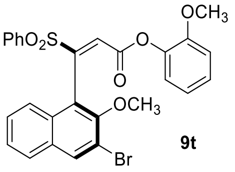

9t

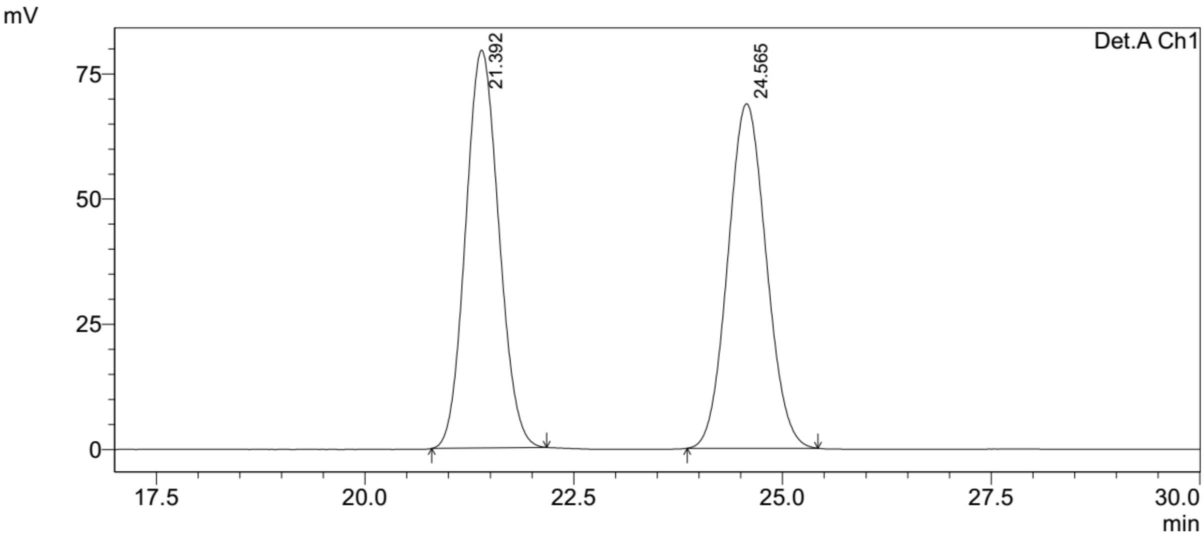

Detector A Ch1 254nm

Peak#	Ret. Time	Area	Height	Area %	Height %
1	21.392	2199006	79368	49.986	53.583
2	24.565	2200279	68753	50.014	46.417
Total		4399285	148121	100.000	100.000

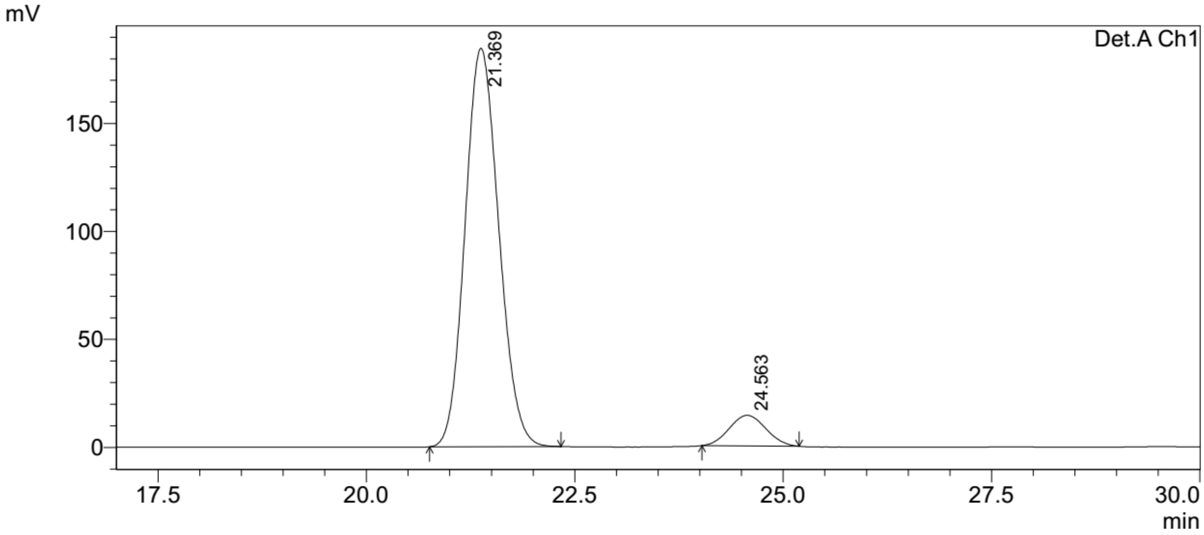

Detector A Ch1 254nm

Peak#	Ret. Time	Area	Height	Area %	Height %
1	21.369	5188589	184565	92.313	92.885
2	24.563	432070	14138	7.687	7.115
Total		5620659	198703	100.000	100.000

Supplementary Figure 114. HPLC spectra of **9t**

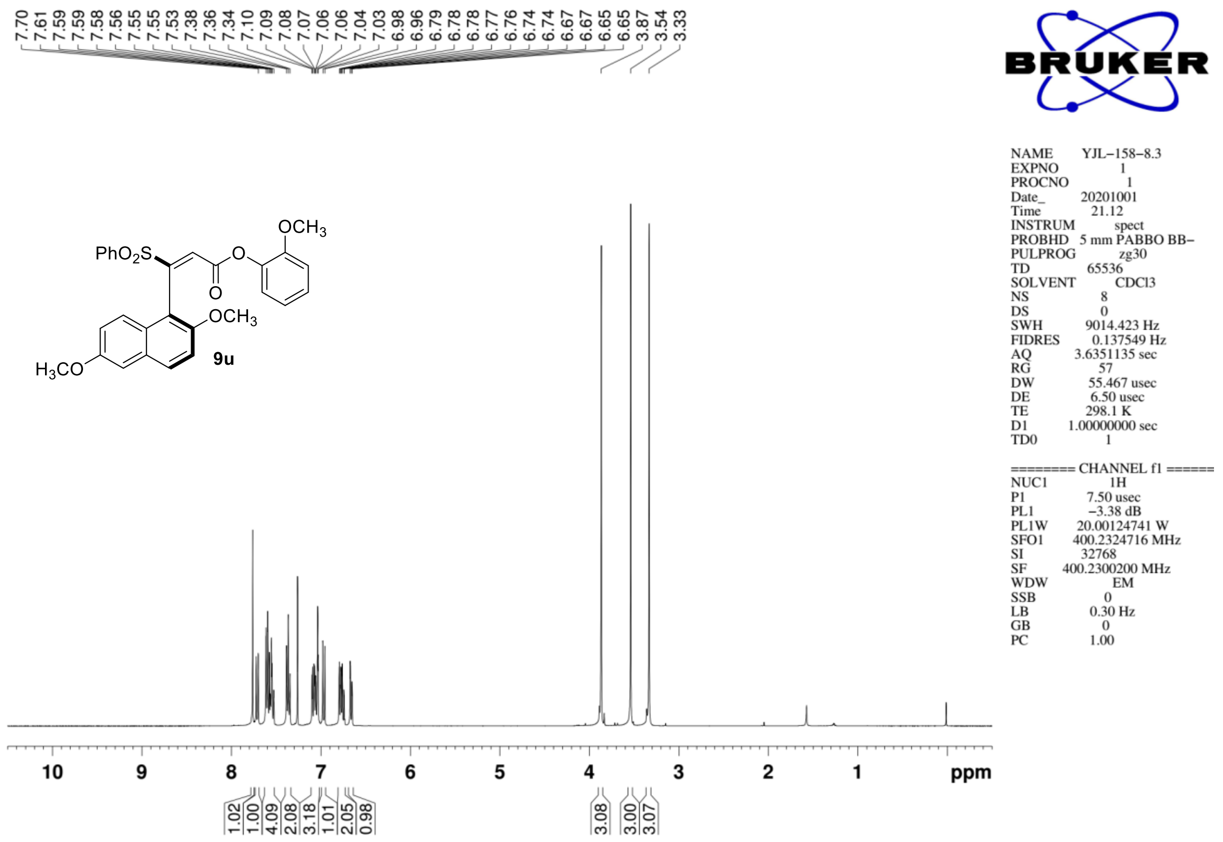

Supplementary Figure 115. ^{1}H NMR ($CDCl_3$, 400MHz) spectra of **9u**

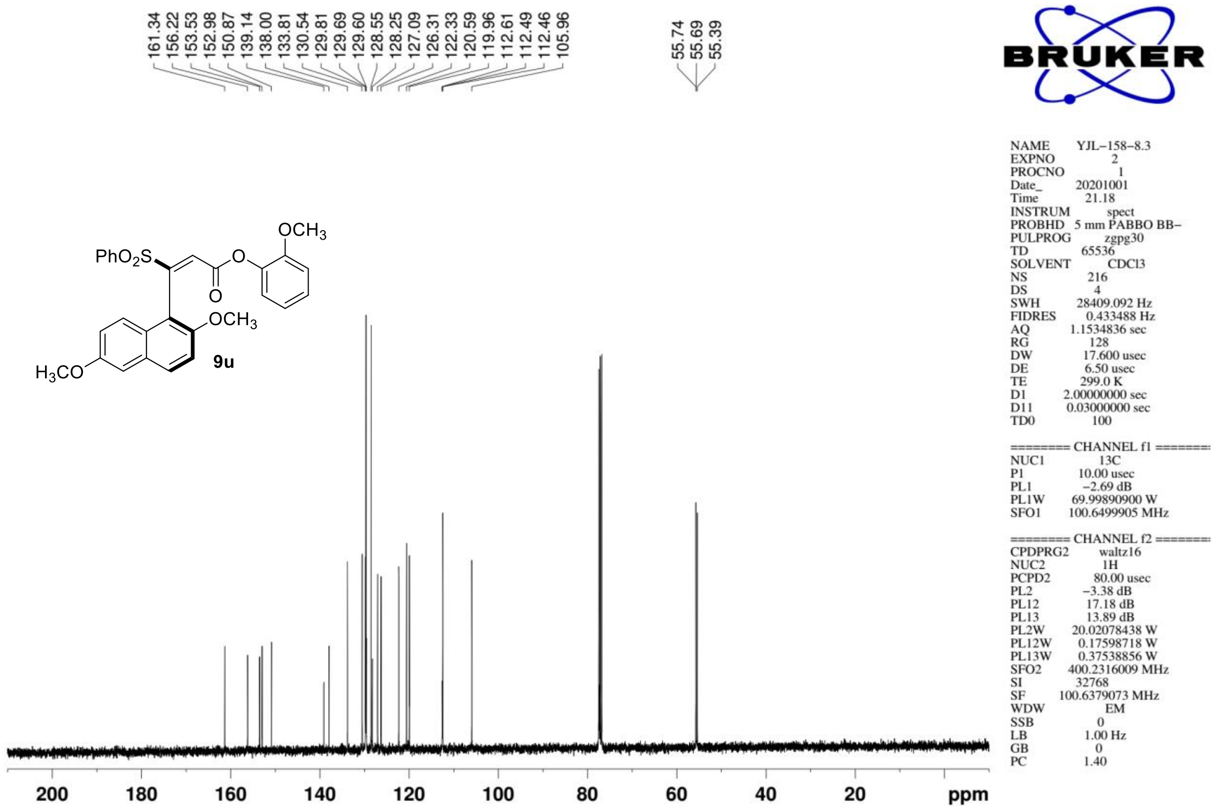

Supplementary Figure 116. ^{13}C NMR ($CDCl_3$, 101MHz) spectra of spectra of **9u**

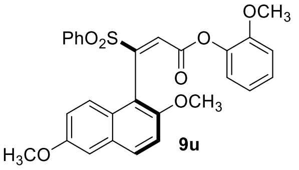

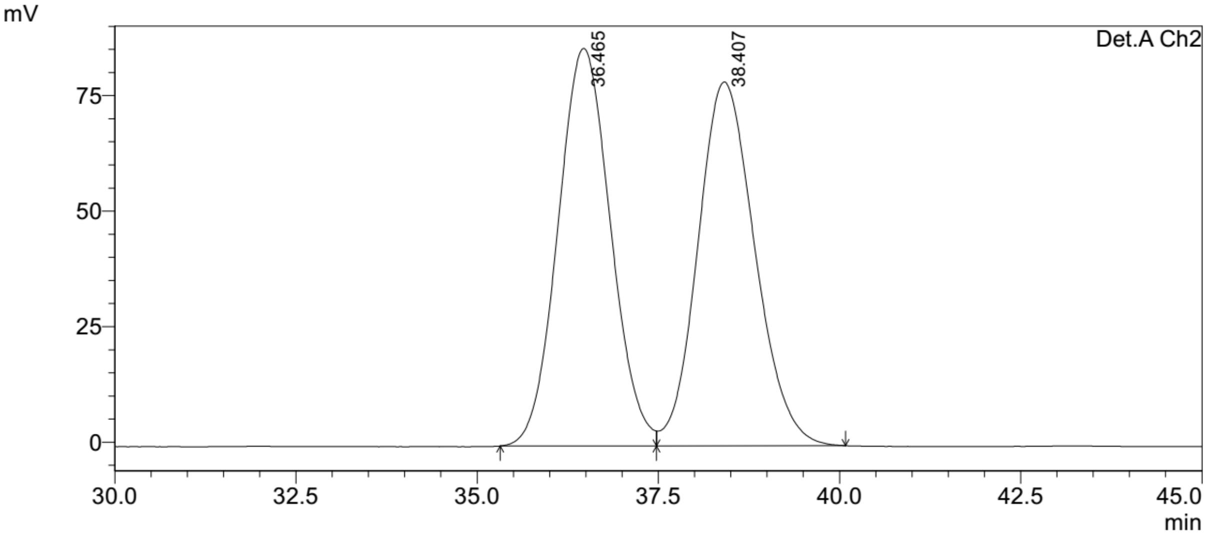

Detector A Ch2 220nm

Peak#	Ret. Time	Area	Height	Area %	Height %
1	36.465	4342040	85994	50.186	52.207
2	38.407	4309872	78723	49.814	47.793
Total		8651912	164717	100.000	100.000

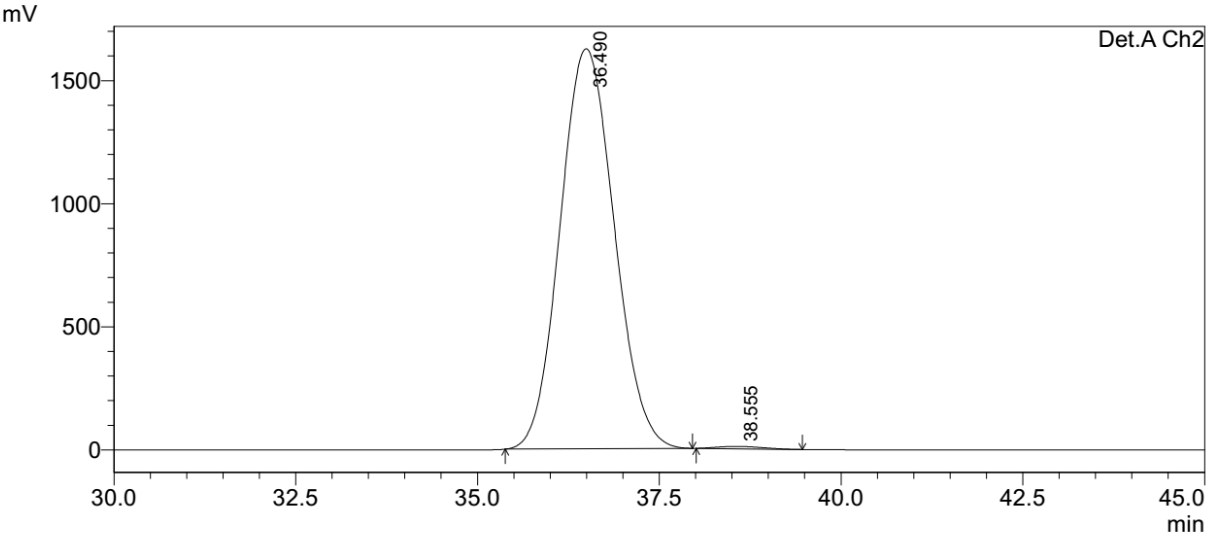

Detector A Ch2 220nm

Peak#	Ret. Time	Area	Height	Area %	Height %
1	36.490	84183714	1624447	99.509	99.417
2	38.555	415731	9527	0.491	0.583
Total		84599445	1633974	100.000	100.000

Supplementary Figure 117. HPLC spectra of **9u**

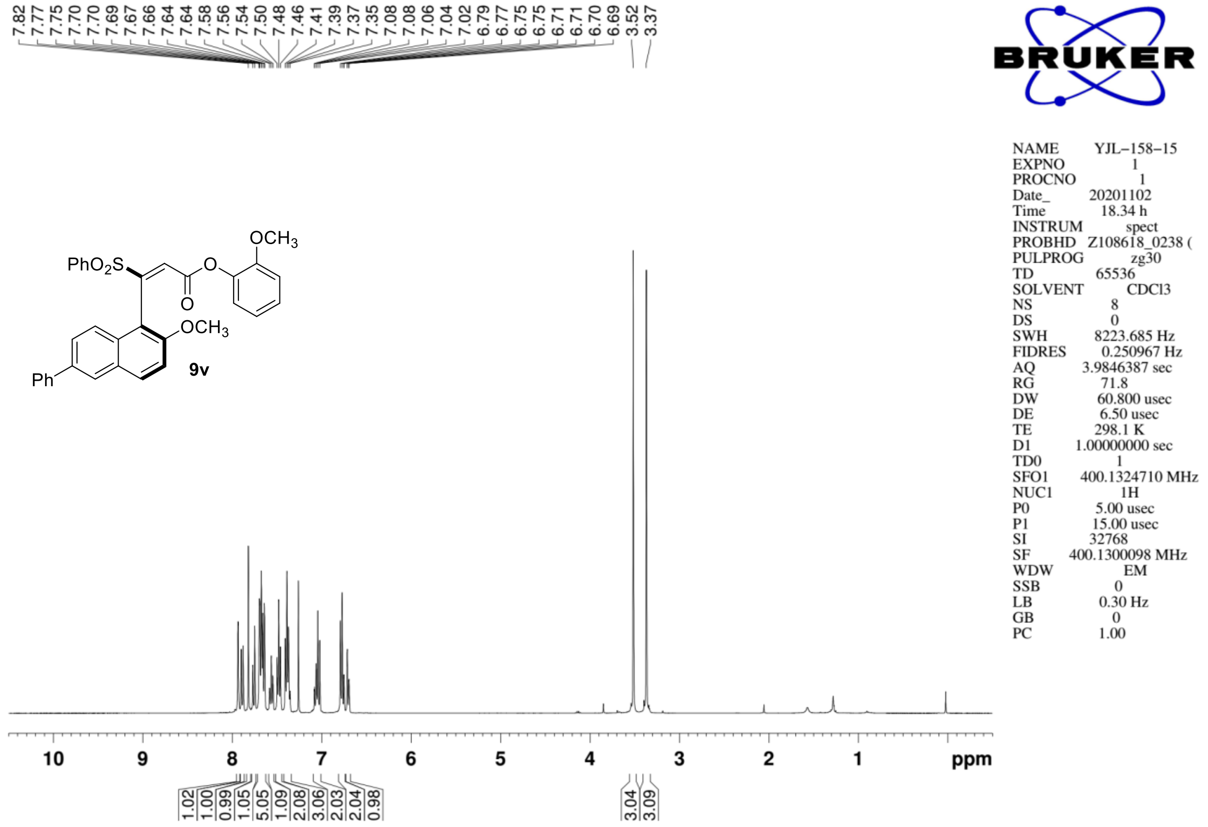

Supplementary Figure 118. ^{1}H NMR ($CDCl_3$, 400MHz) spectra of **9v**

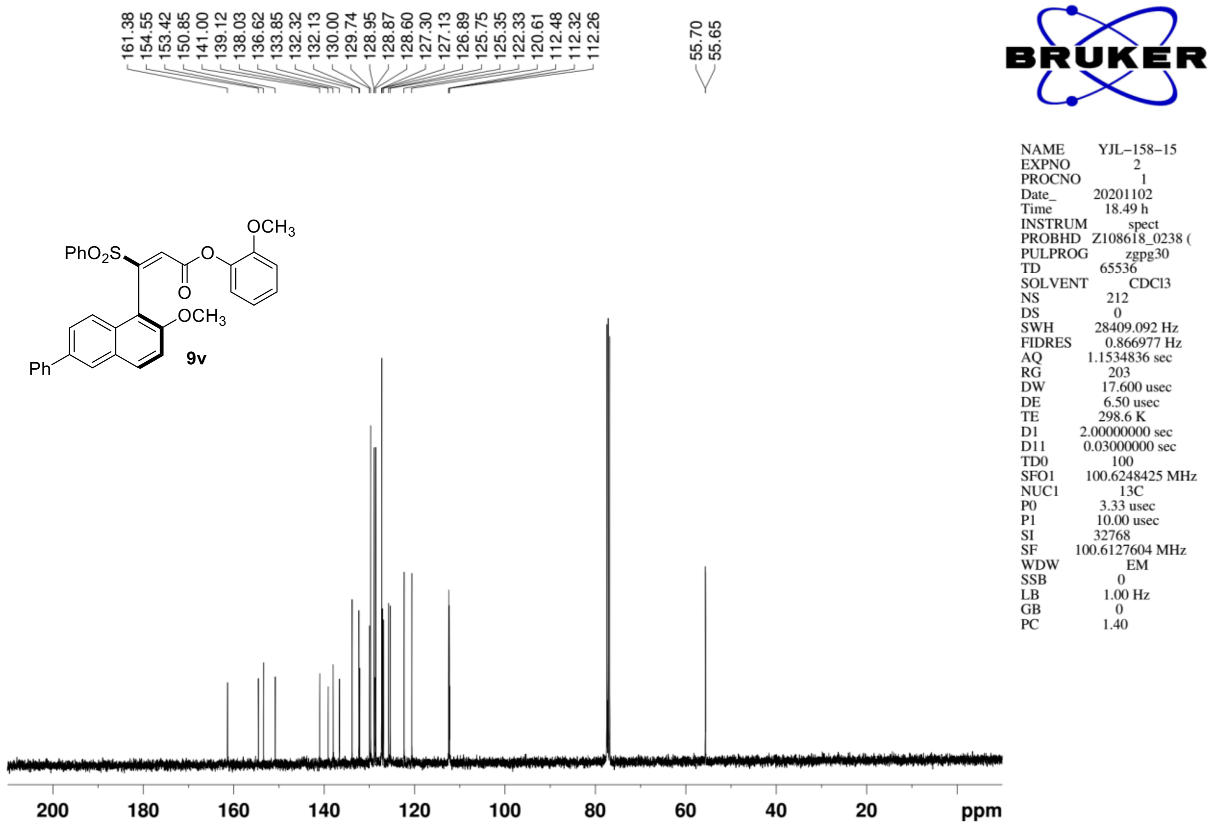

Supplementary Figure 119. ^{13}C NMR ($CDCl_3$, 101MHz) spectra of spectra of **9v**

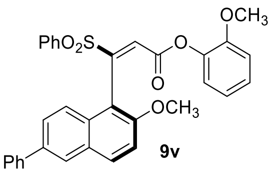

9v

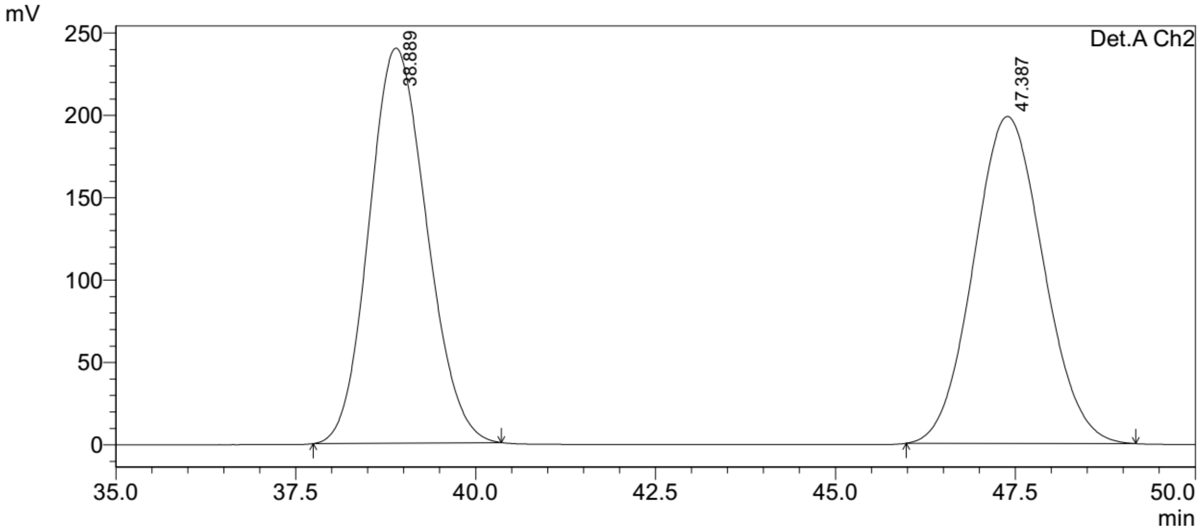

Detector A Ch2 220nm

Peak#	Ret. Time	Area	Height	Area %	Height %
1	38.889	13367755	239768	49.976	54.709
2	47.387	13380534	198497	50.024	45.291
Total		26748290	438265	100.000	100.000

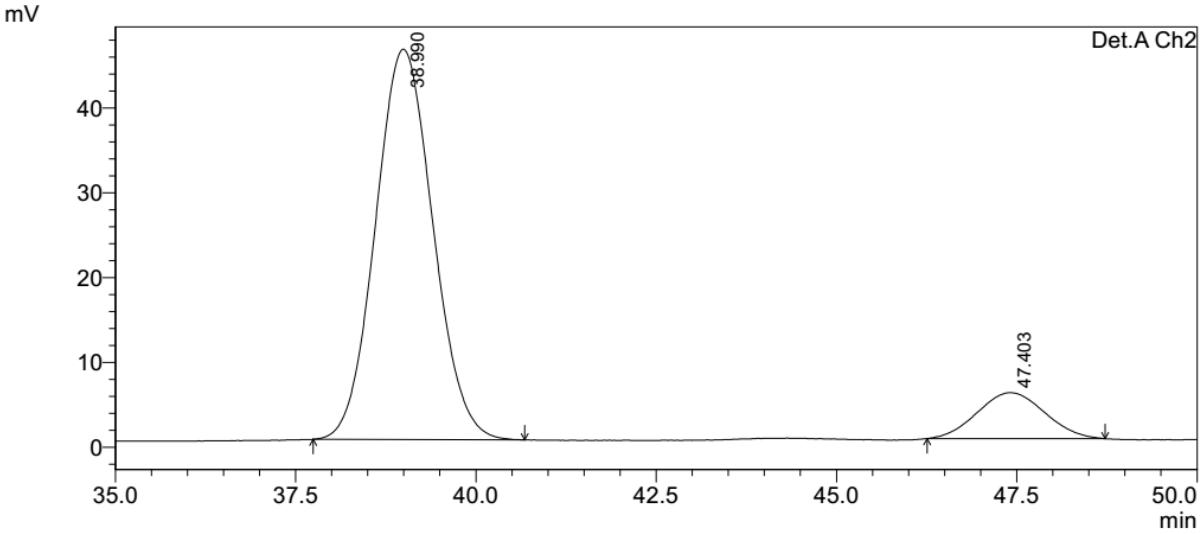

Detector A Ch2 220nm

Peak#	Ret. Time	Area	Height	Area %	Height %
1	38.990	2549295	46025	87.856	89.457
2	47.403	352376	5424	12.144	10.543
Total		2901671	51450	100.000	100.000

Supplementary Figure 120. HPLC spectra of **9v**

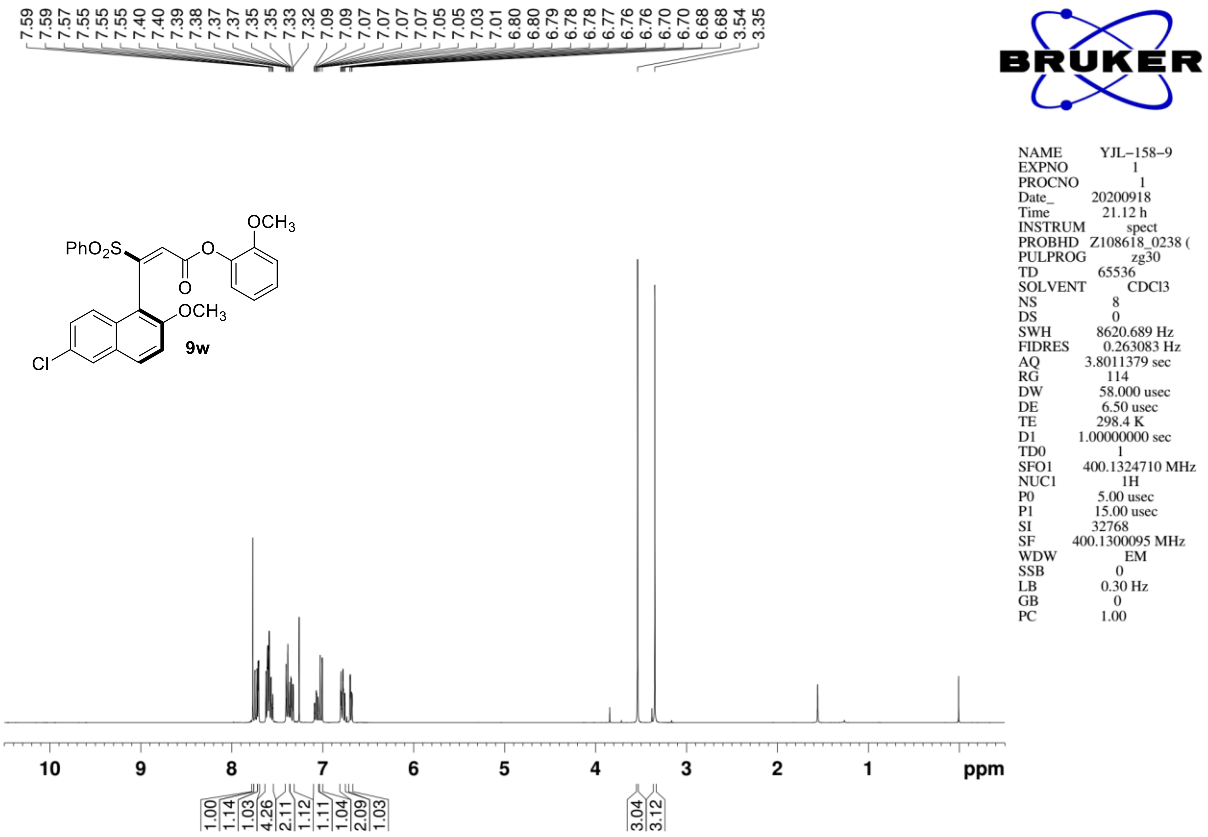

Supplementary Figure 121. ^{1}H NMR ($CDCl_3$, 400MHz) spectra of **9w**

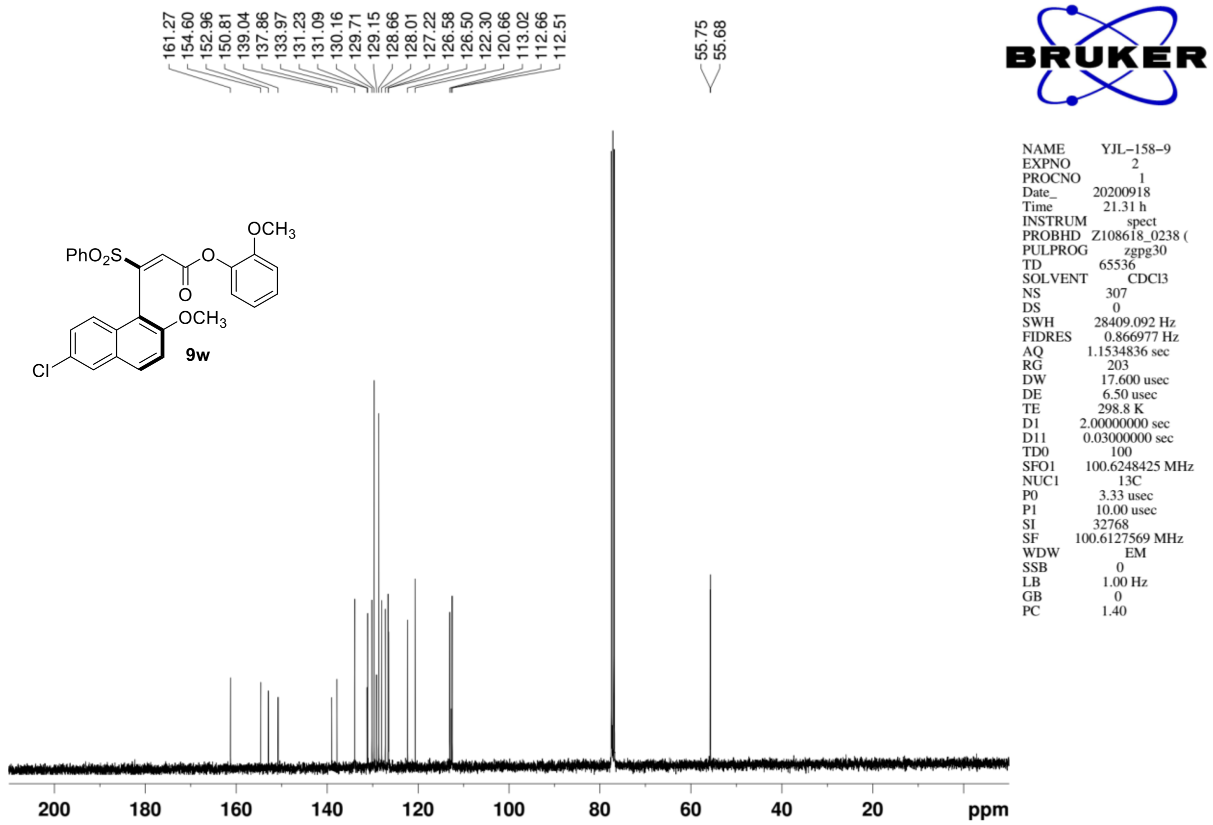

Supplementary Figure 122. ^{13}C NMR ($CDCl_3$, 101MHz) spectra of spectra of **9w**

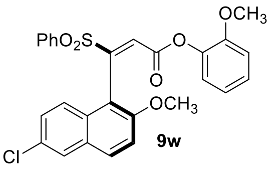

9w

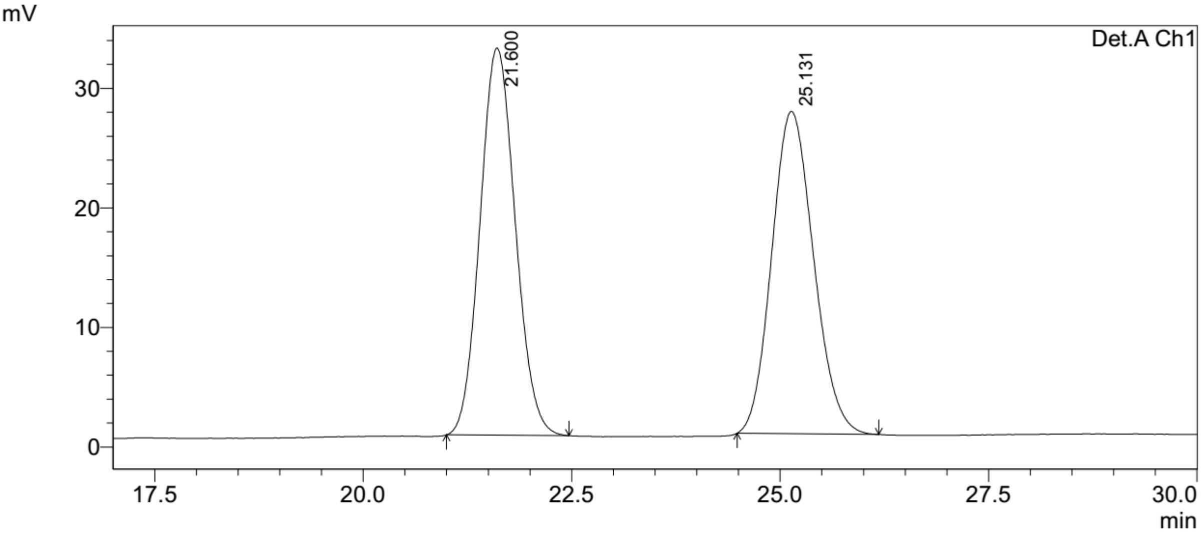

Detector A Ch1 254nm

Peak#	Ret. Time	Area	Height	Area %	Height %
1	21.600	952175	32388	50.256	54.560
2	25.131	942484	26974	49.744	45.440
Total		1894659	59362	100.000	100.000

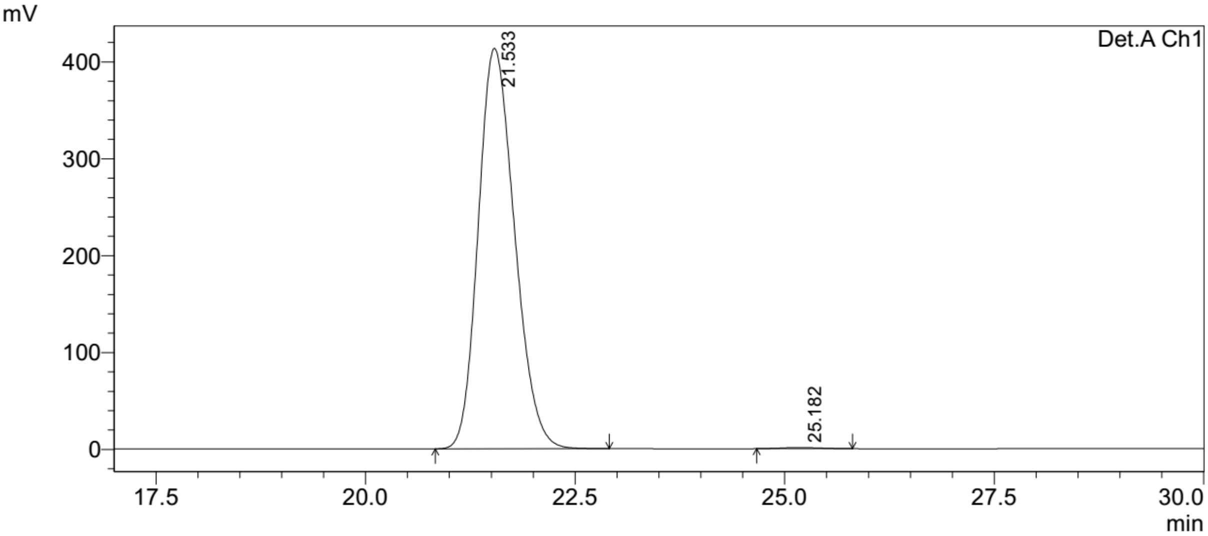

Detector A Ch1 254nm

Peak#	Ret. Time	Area	Height	Area %	Height %
1	21.533	12455346	413234	99.685	99.708
2	25.182	39390	1209	0.315	0.292
Total		12494737	414443	100.000	100.000

Supplementary Figure 123. HPLC spectra of **9w**

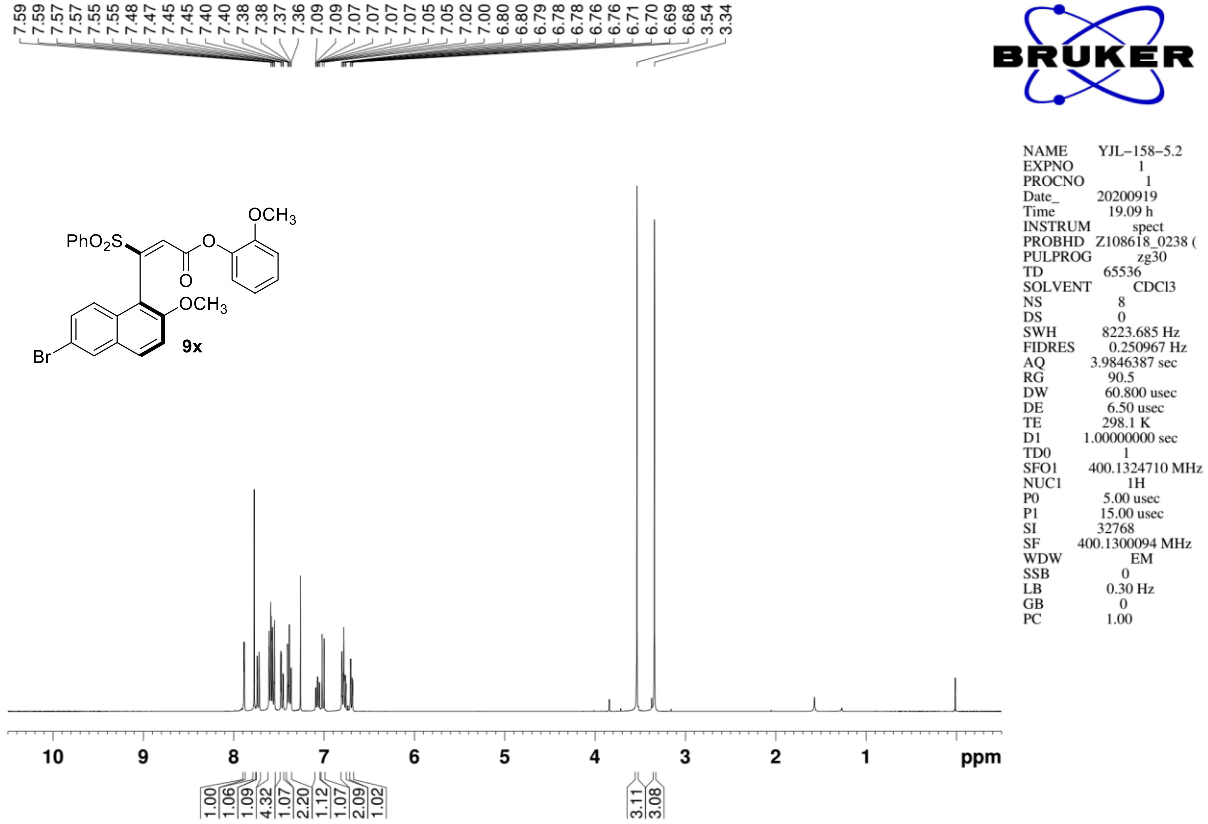

Supplementary Figure 124. ^{1}H NMR ($CDCl_3$, 400MHz) spectra of **9x**

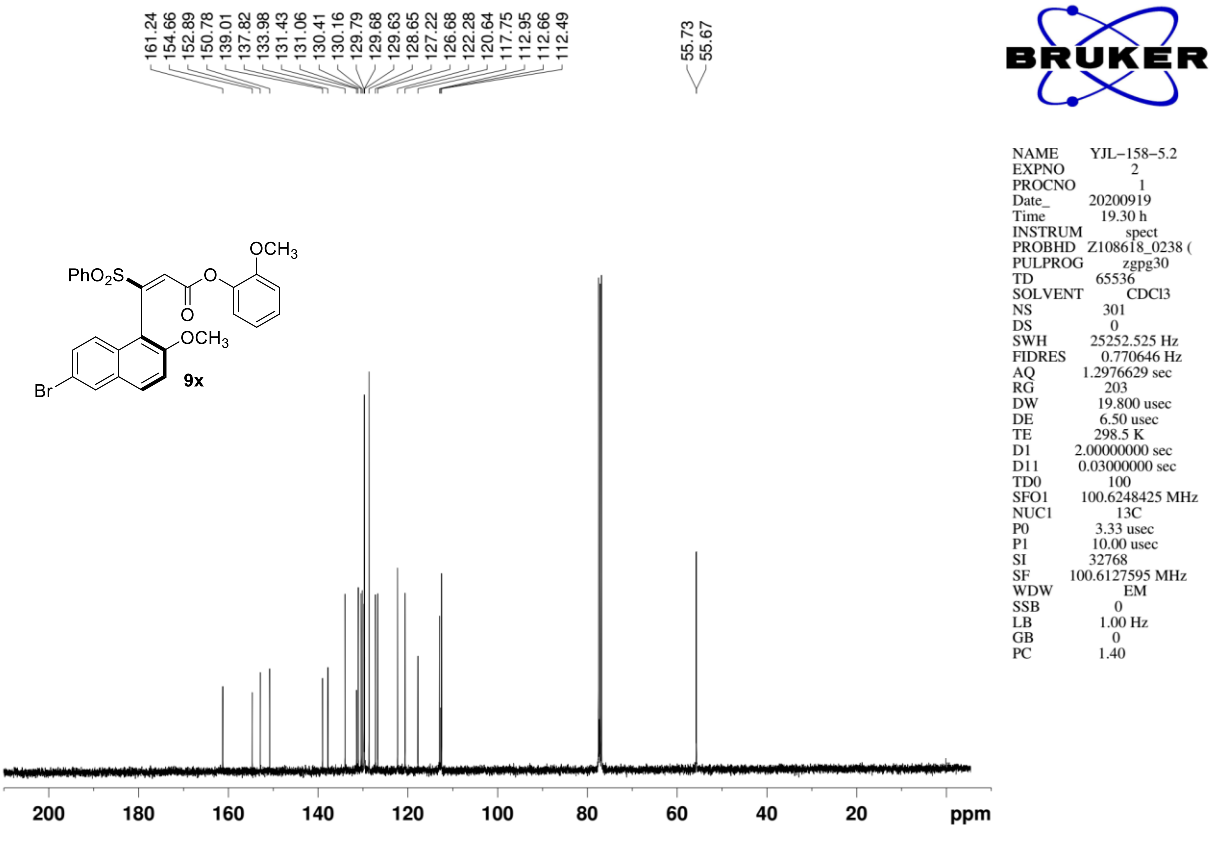

Supplementary Figure 125. ^{13}C NMR ($CDCl_3$, 101MHz) spectra of spectra of **9x**

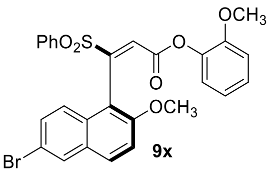

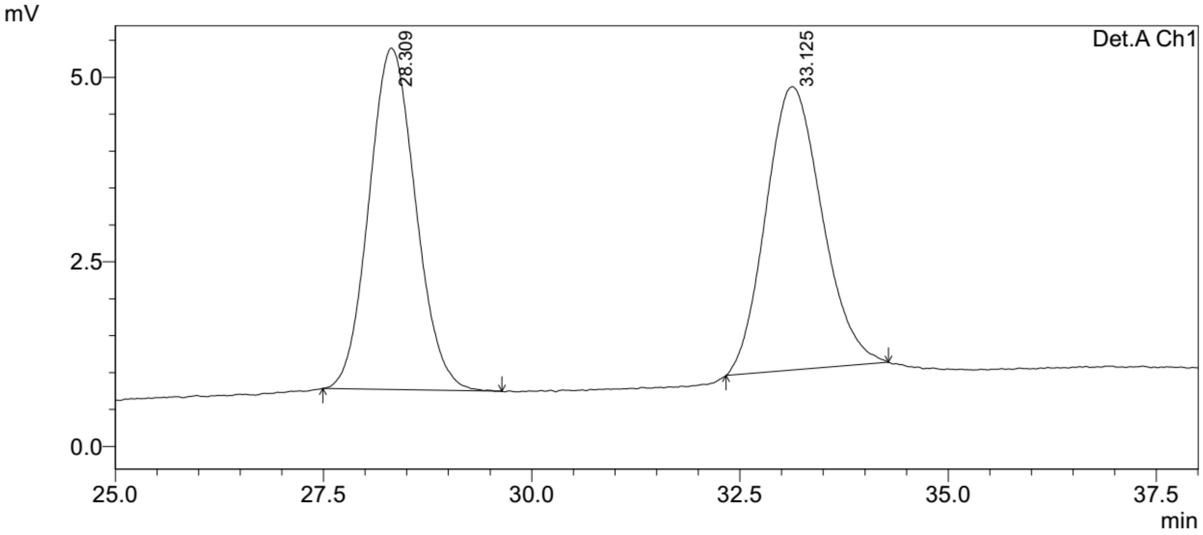

Detector A Ch1 254nm

Peak#	Ret. Time	Area	Height	Area %	Height %
1	28.309	180173	4623	49.979	54.649
2	33.125	180321	3836	50.021	45.351
Total		360494	8459	100.000	100.000

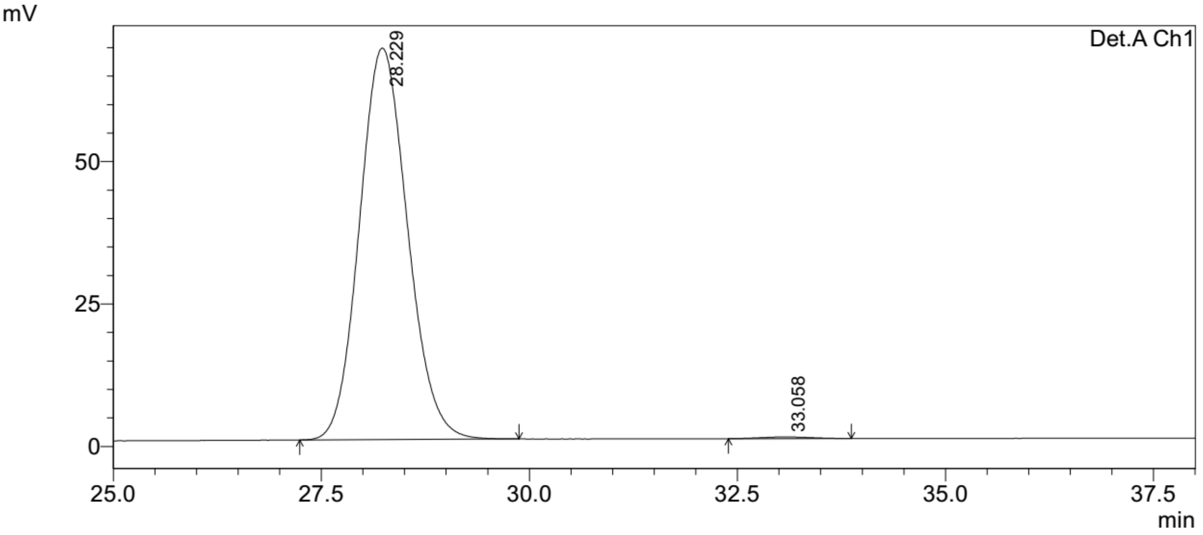

Detector A Ch1 254nm

Peak#	Ret. Time	Area	Height	Area %	Height %
1	28.229	2764718	68734	99.554	99.579
2	33.058	12392	291	0.446	0.421
Total		2777109	69025	100.000	100.000

Supplementary Figure 126. HPLC spectra of **9x**

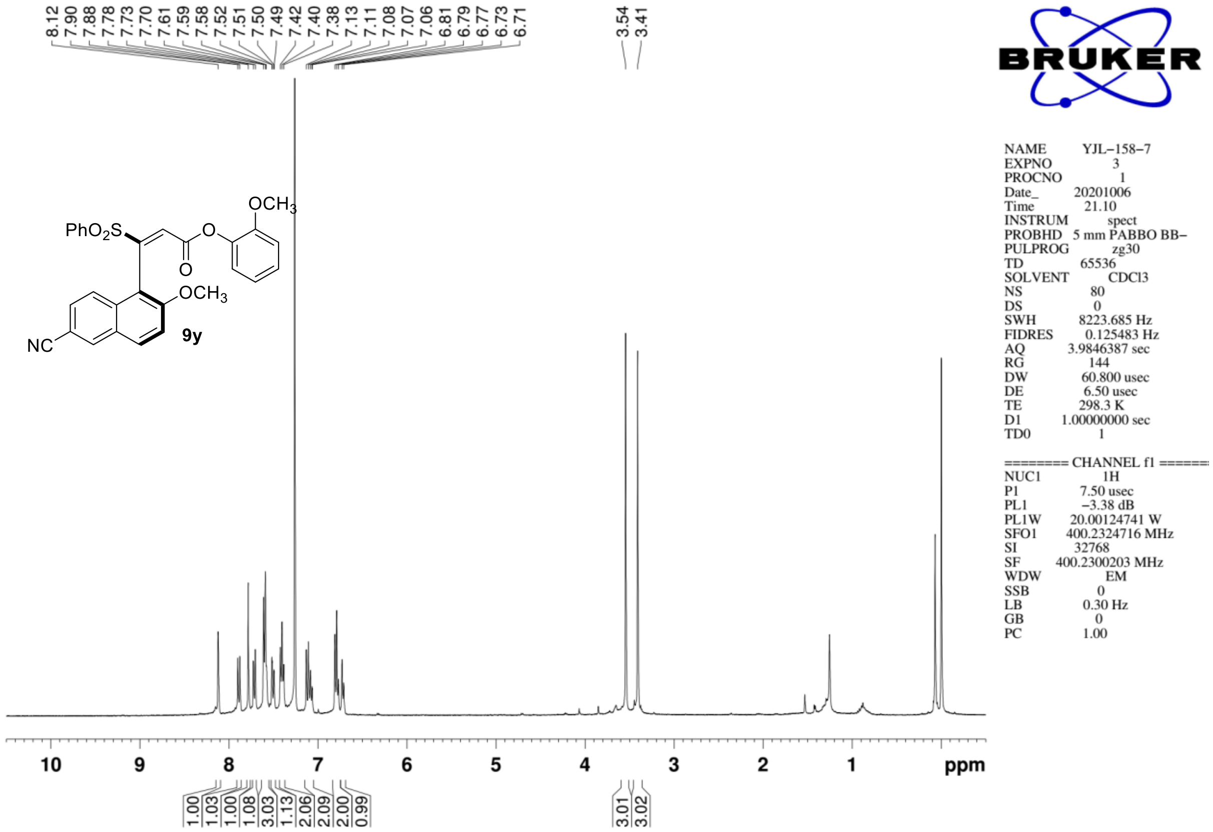

Supplementary Figure 127. ^{1}H NMR ($CDCl_3$, 400MHz) spectra of **9y**

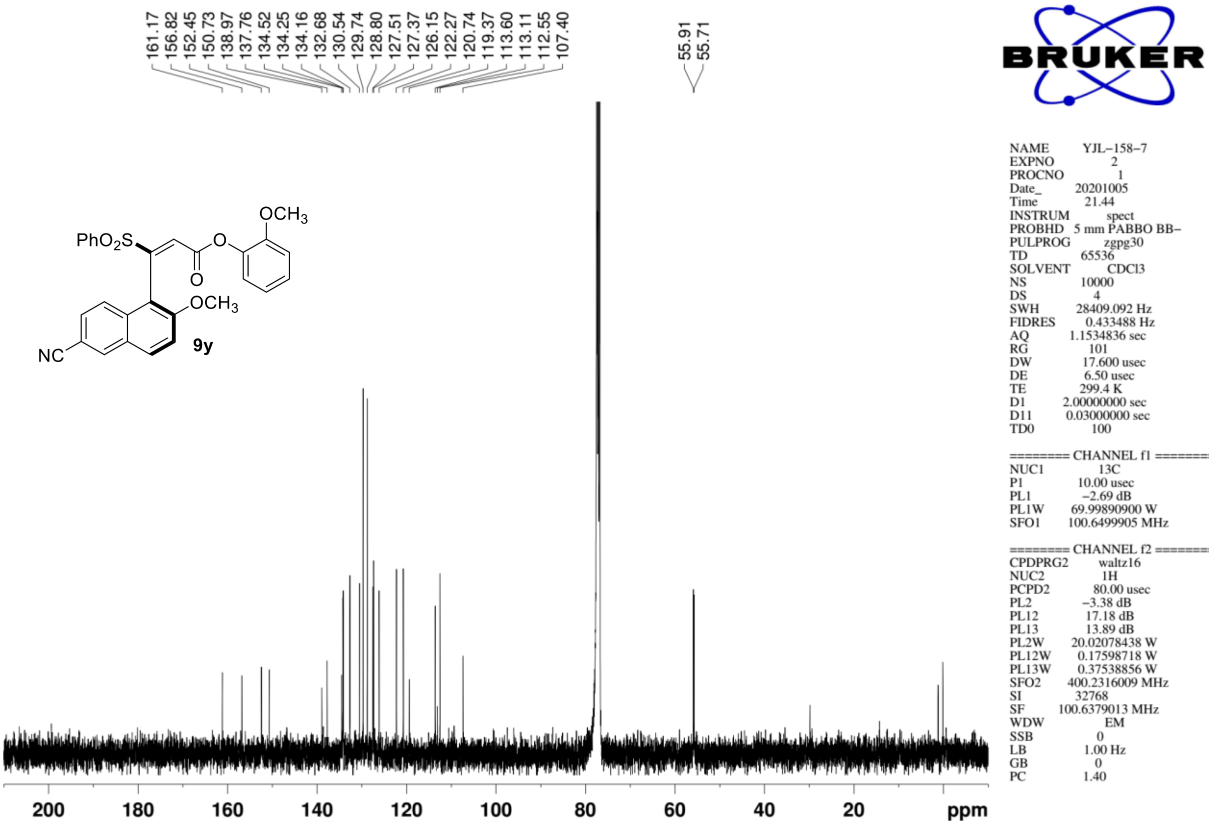

Supplementary Figure 128. ^{13}C NMR ($CDCl_3$, 101MHz) spectra of spectra of **9y**

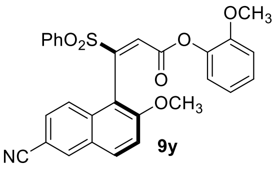

9y

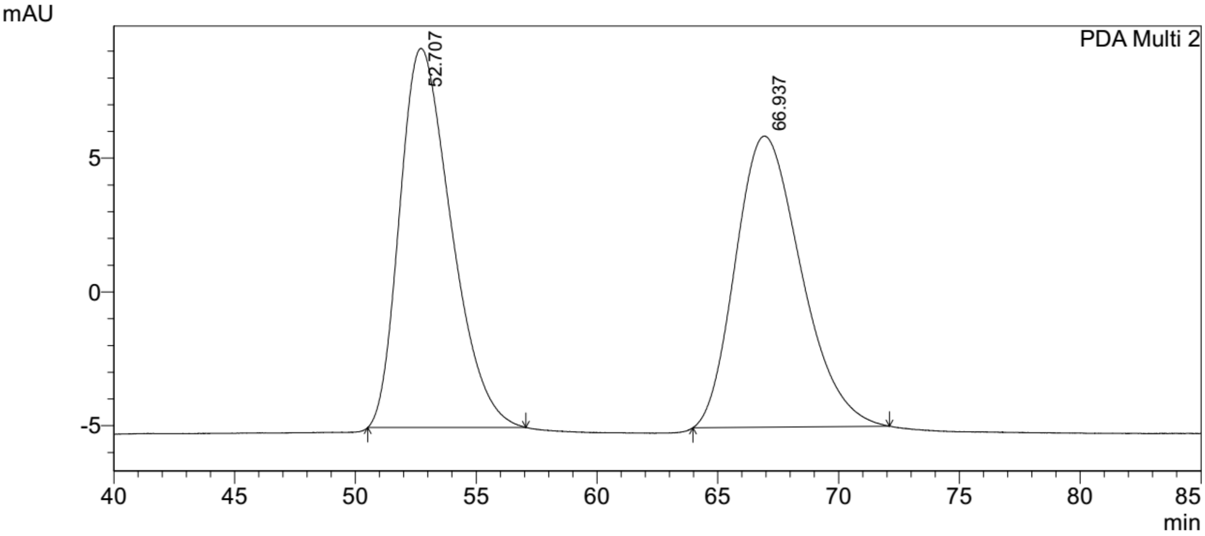

PDA Ch2 220nm 4nm

Peak#	Ret. Time	Area	Height	Area %	Height %
1	52.707	2099298	14169	50.504	56.565
2	66.937	2057366	10880	49.496	43.435
Total		4156664	25050	100.000	100.000

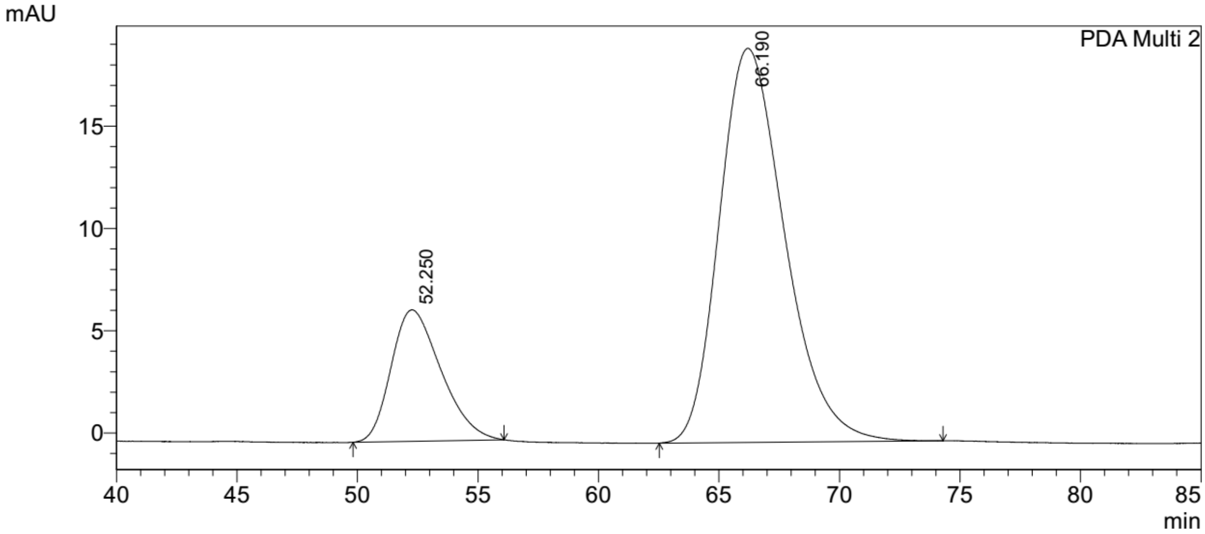

PDA Ch2 220nm 4nm

Peak#	Ret. Time	Area	Height	Area %	Height %
1	52.250	924217	6425	20.232	25.014
2	66.190	3643979	19261	79.768	74.986
Total		4568195	25686	100.000	100.000

Supplementary Figure 129. HPLC spectra of **9y**

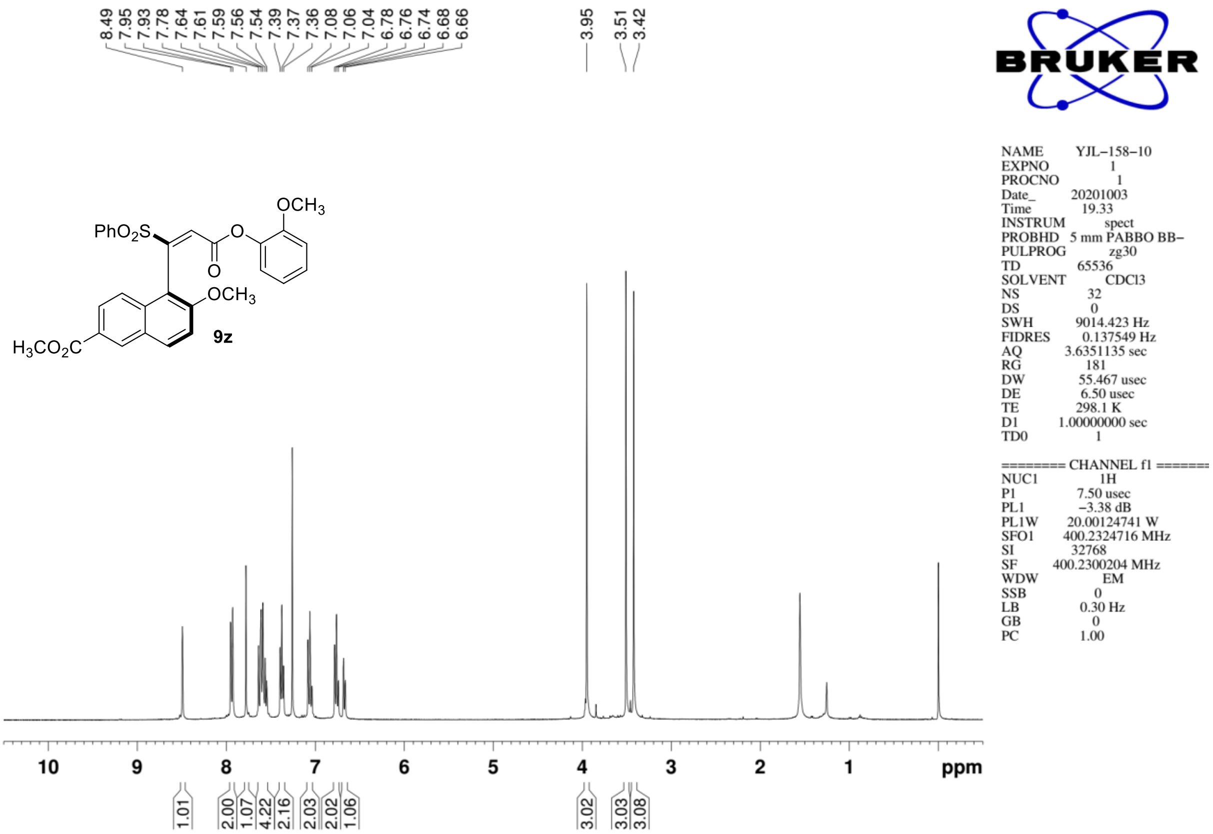

Supplementary Figure 130. ^{1}H NMR ($CDCl_3$, 400MHz) spectra of **9z**

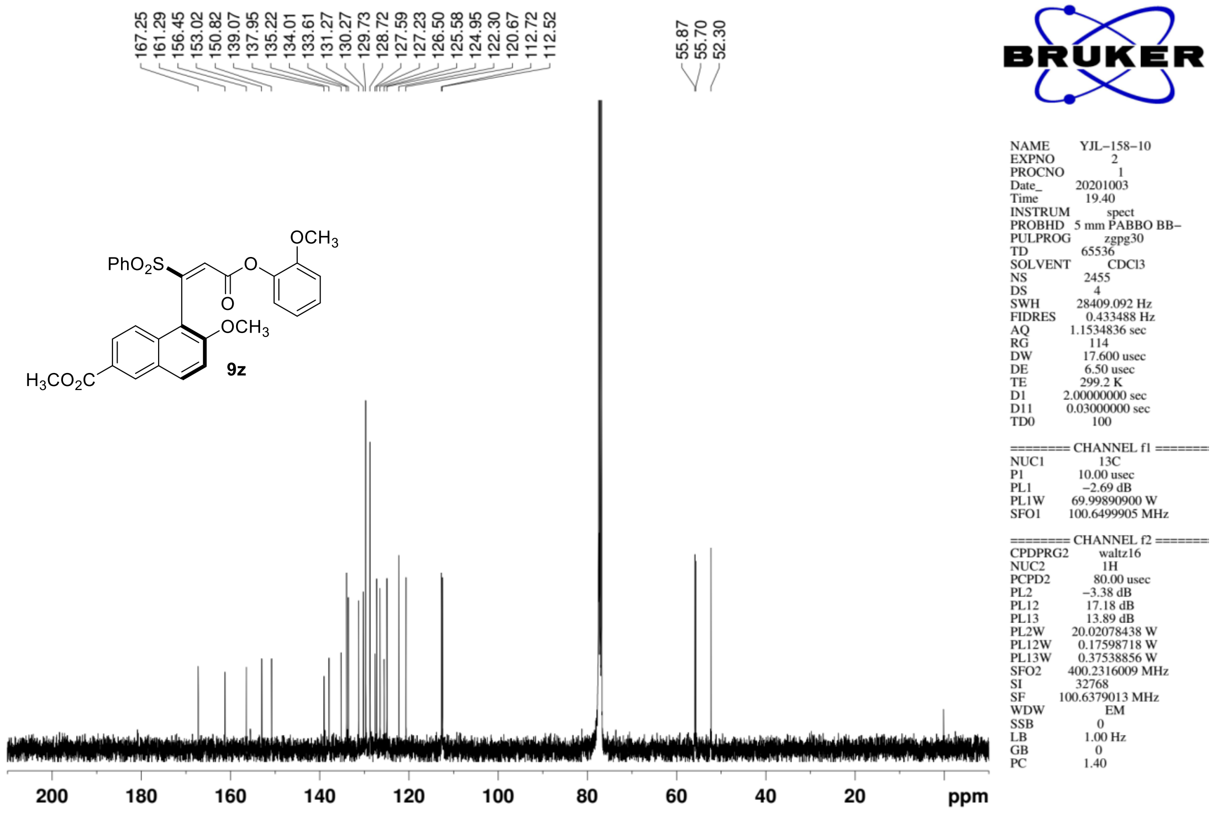

Supplementary Figure 131. ^{13}C NMR ($CDCl_3$, 101MHz) spectra of spectra of **9z**

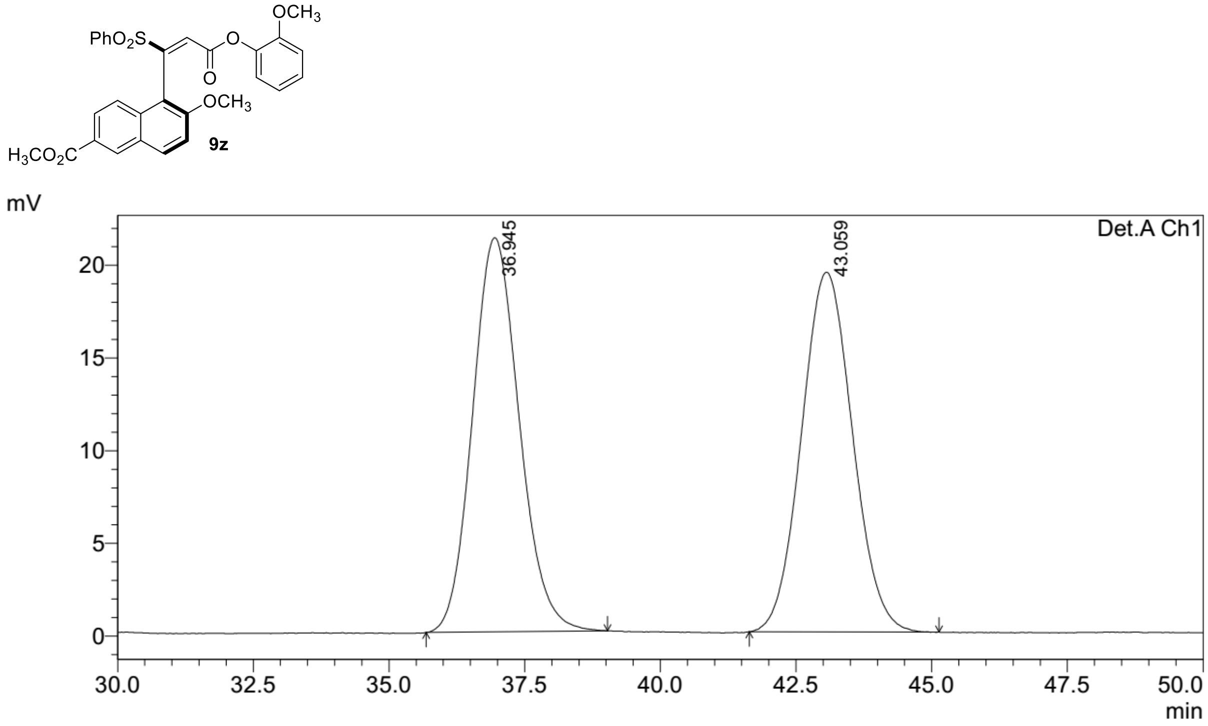

Detector A Ch1 254nm

Peak#	Ret. Time	Area	Height	Area %	Height %
1	36.945	1273672	21254	50.056	52.286
2	43.059	1270804	19396	49.944	47.714
Total		2544476	40649	100.000	100.000

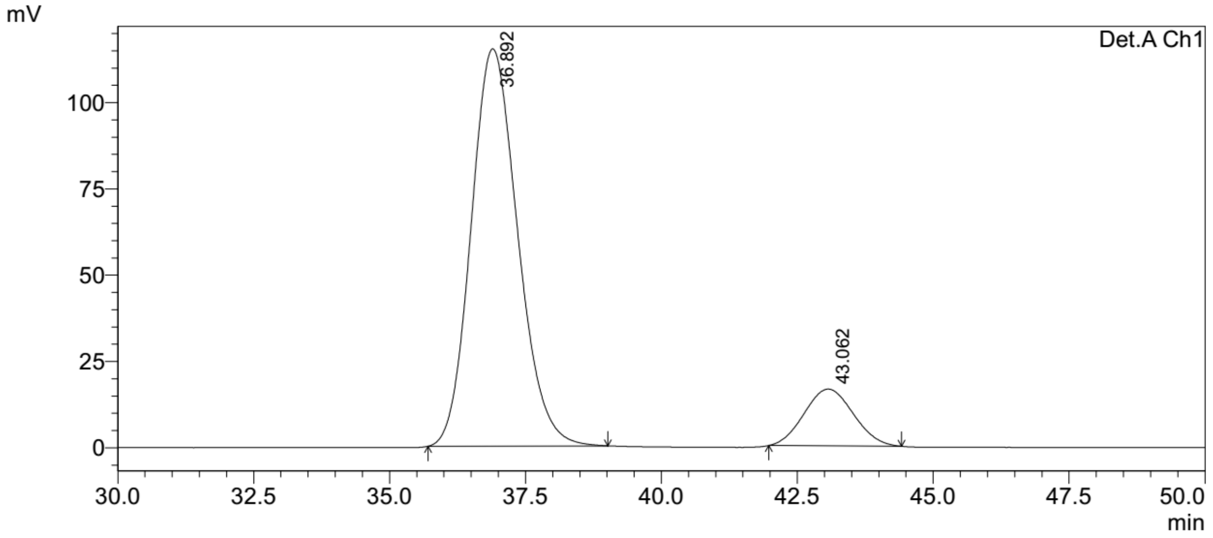

Detector A Ch1 254nm

Peak#	Ret. Time	Area	Height	Area %	Height %
1	36.892	6857515	115103	86.867	87.507
2	43.062	1036715	16433	13.133	12.493
Total		7894231	131536	100.000	100.000

Supplementary Figure 132. HPLC spectra of **9z**

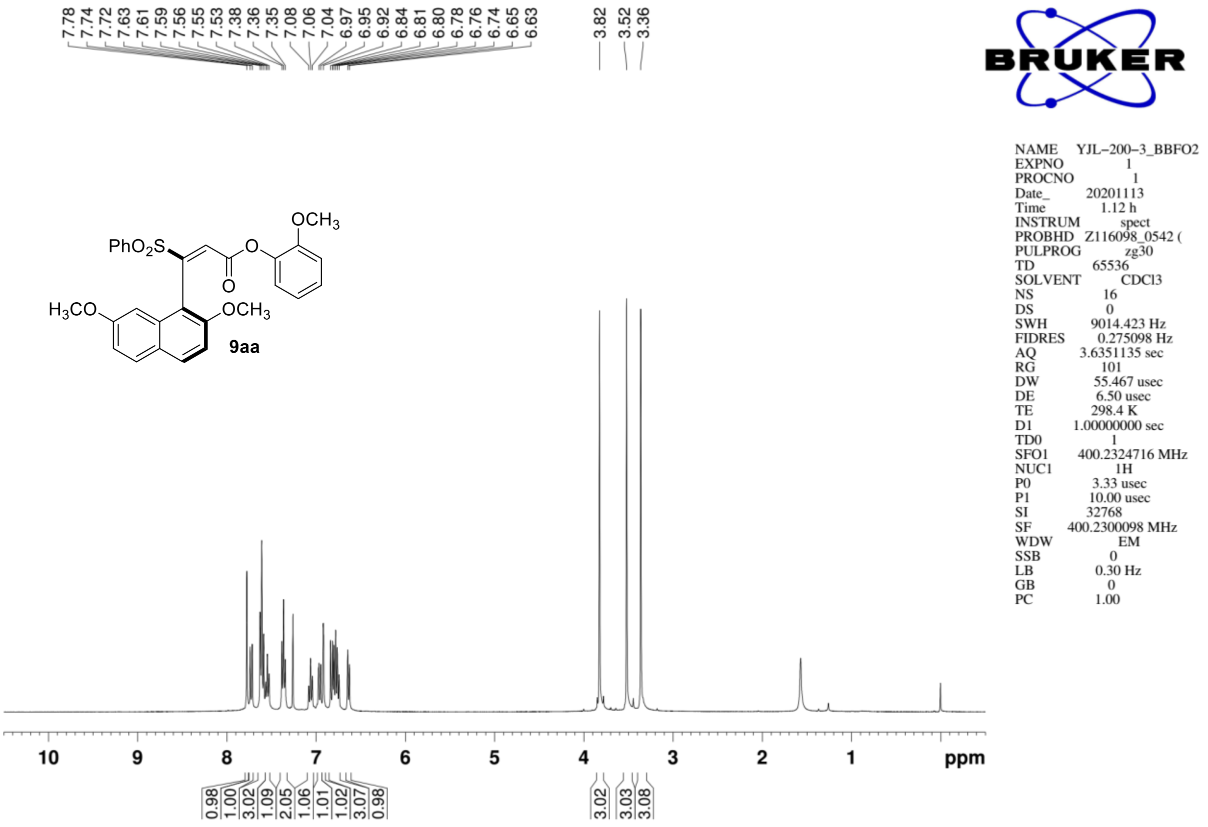

Supplementary Figure 133. ^{1}H NMR ($CDCl_3$, 400MHz) spectra of **9aa**

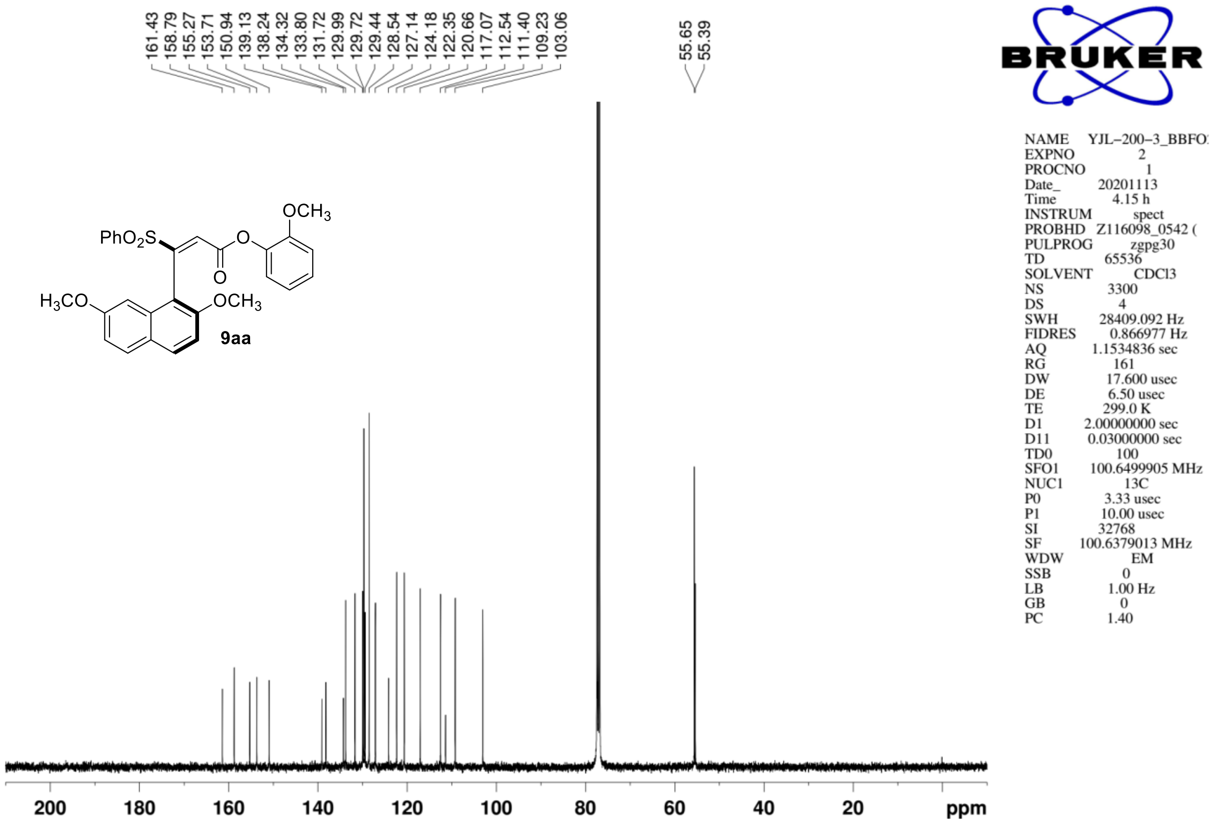

Supplementary Figure 134. ^{13}C NMR ($CDCl_3$, 101MHz) spectra of spectra of **9aa**

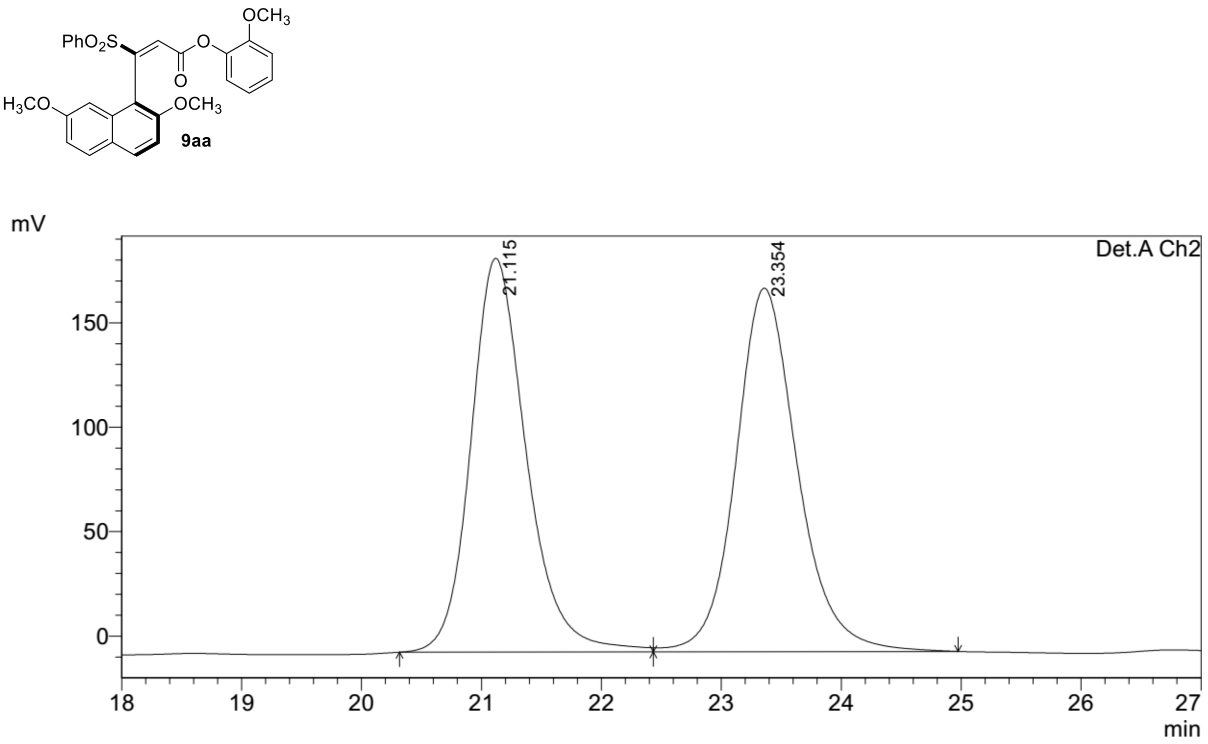

Detector A Ch2 220nm

Peak#	Ret. Time	Area	Height	Area %	Height %
1	21.115	6066886	188517	49.341	51.991
2	23.354	6229031	174078	50.659	48.009
Total		12295917	362594	100.000	100.000

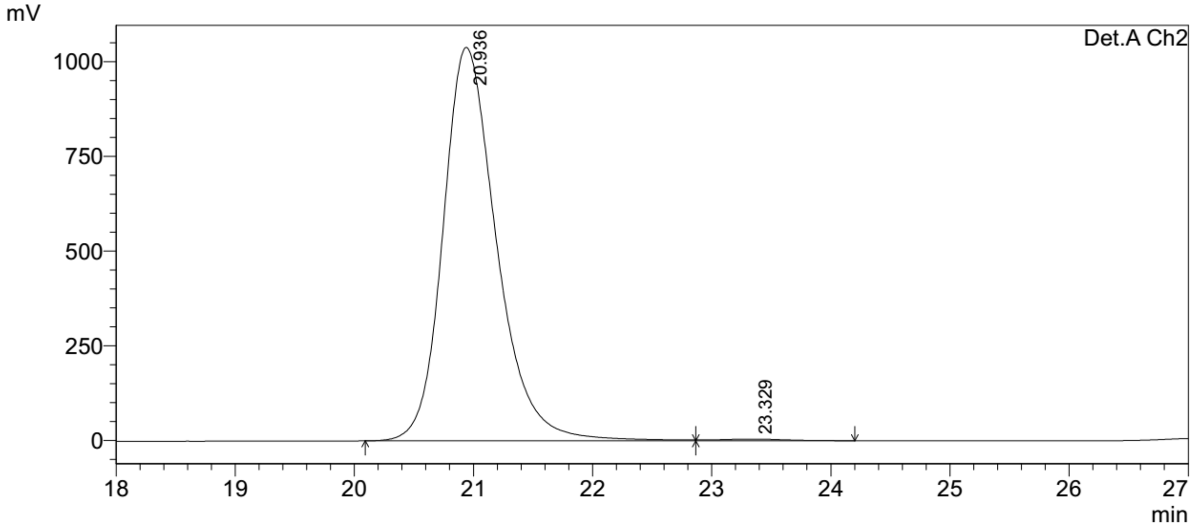

Detector A Ch2 220nm

Peak#	Ret. Time	Area	Height	Area %	Height %
1	20.936	32458416	1038717	99.479	99.586
2	23.329	170103	4316	0.521	0.414
Total		32628518	1043033	100.000	100.000

Supplementary Figure 135. HPLC spectra of **9aa**

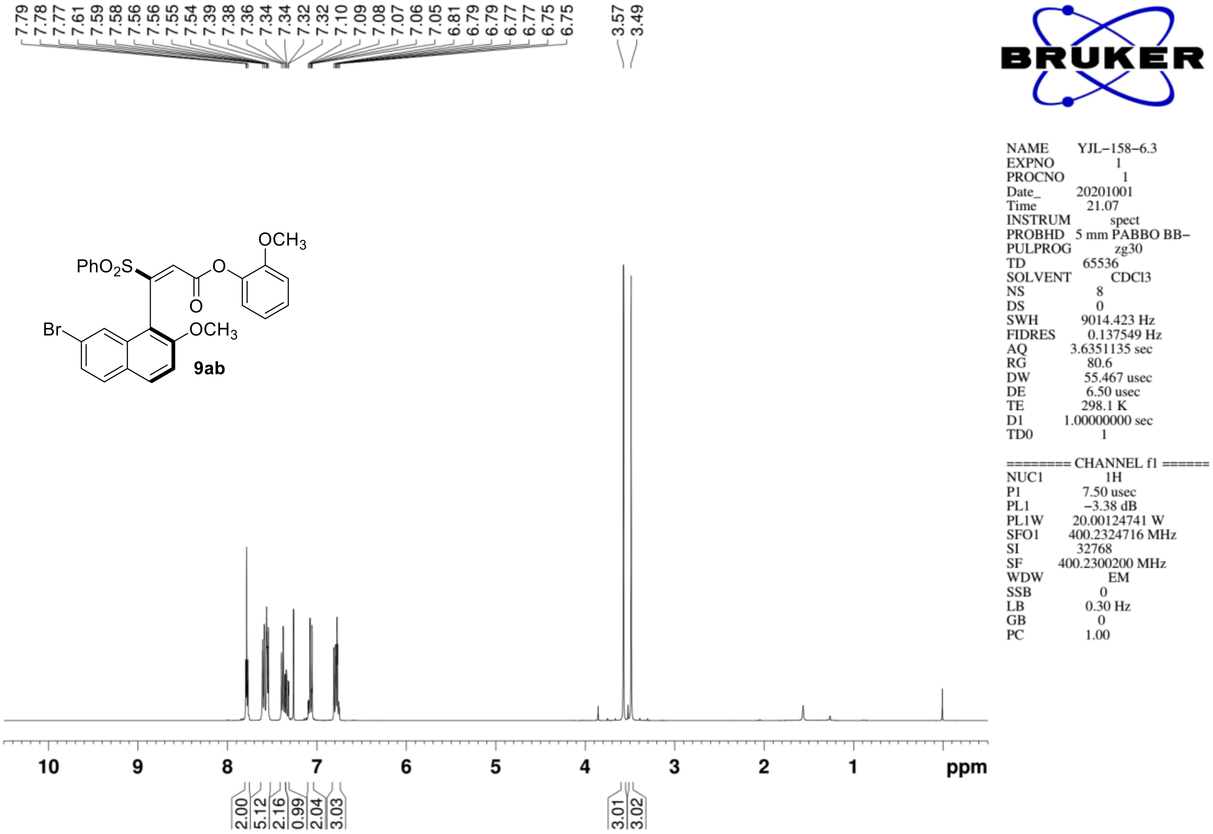

Supplementary Figure 136. ^{1}H NMR ($CDCl_3$, 400MHz) spectra of **9ab**

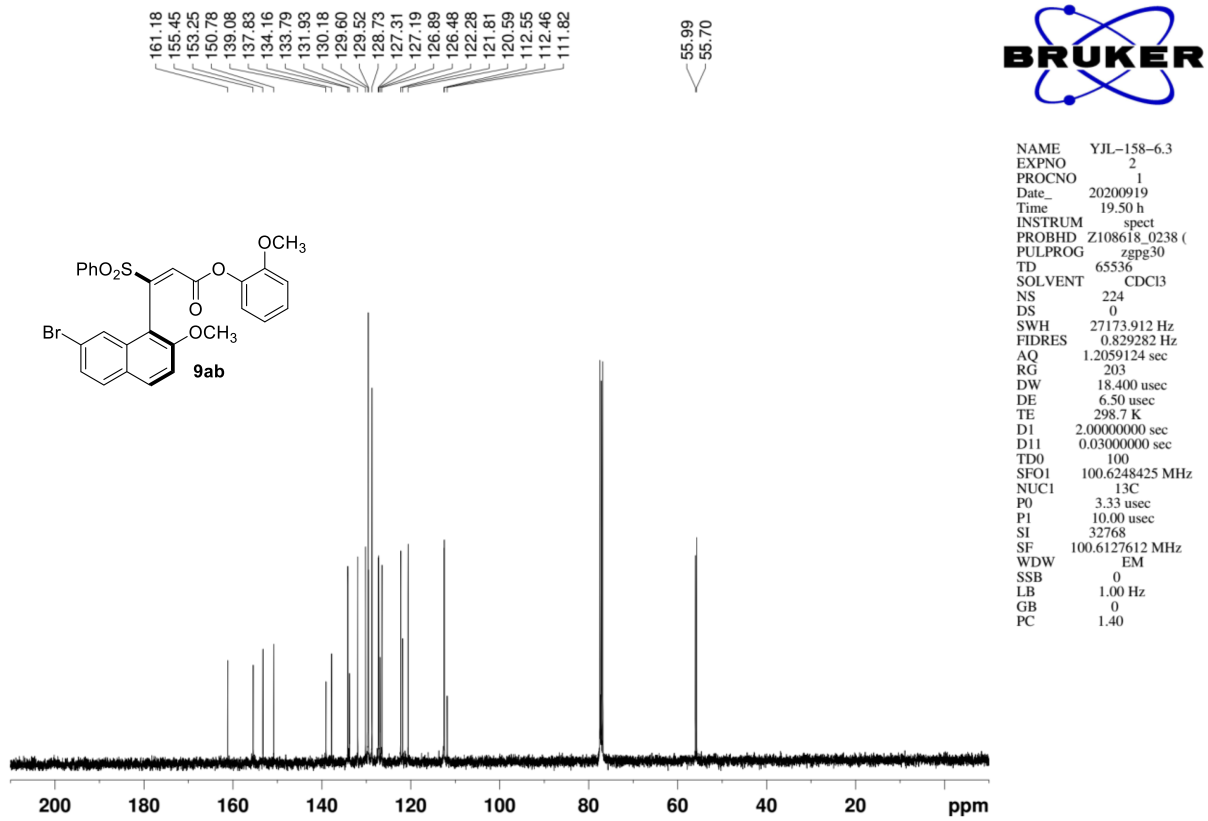

Supplementary Figure 137. ^{13}C NMR ($CDCl_3$, 101MHz) spectra of spectra of **9ab**

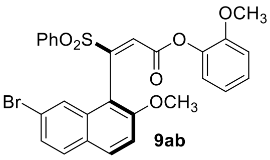

9ab

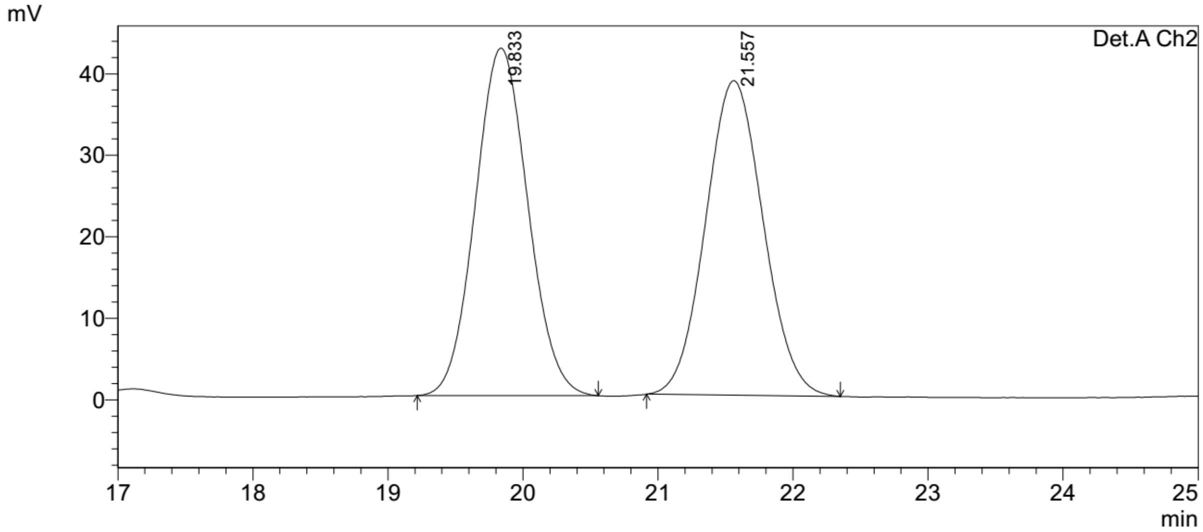

Detector A Ch2 220nm

Peak#	Ret. Time	Area	Height	Area %	Height %
1	19.833	1156244	42623	50.232	52.488
2	21.557	1145580	38582	49.768	47.512
Total		2301824	81205	100.000	100.000

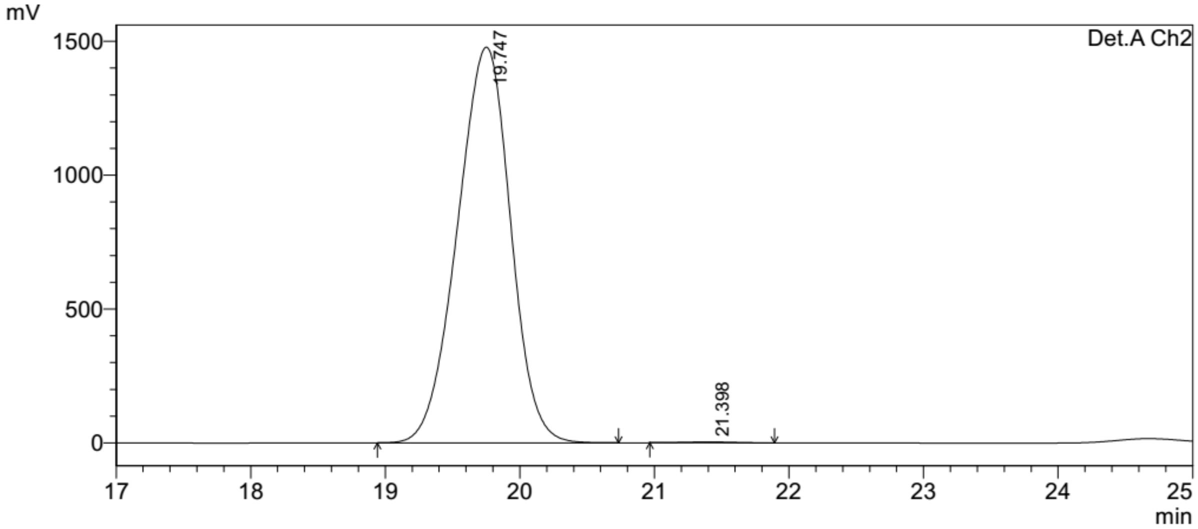

Detector A Ch2 220nm

Peak#	Ret. Time	Area	Height	Area %	Height %
1	19.747	40612247	1477747	99.787	99.779
2	21.398	86775	3278	0.213	0.221
Total		40699022	1481025	100.000	100.000

Supplementary Figure 138. HPLC spectra of **9ab**

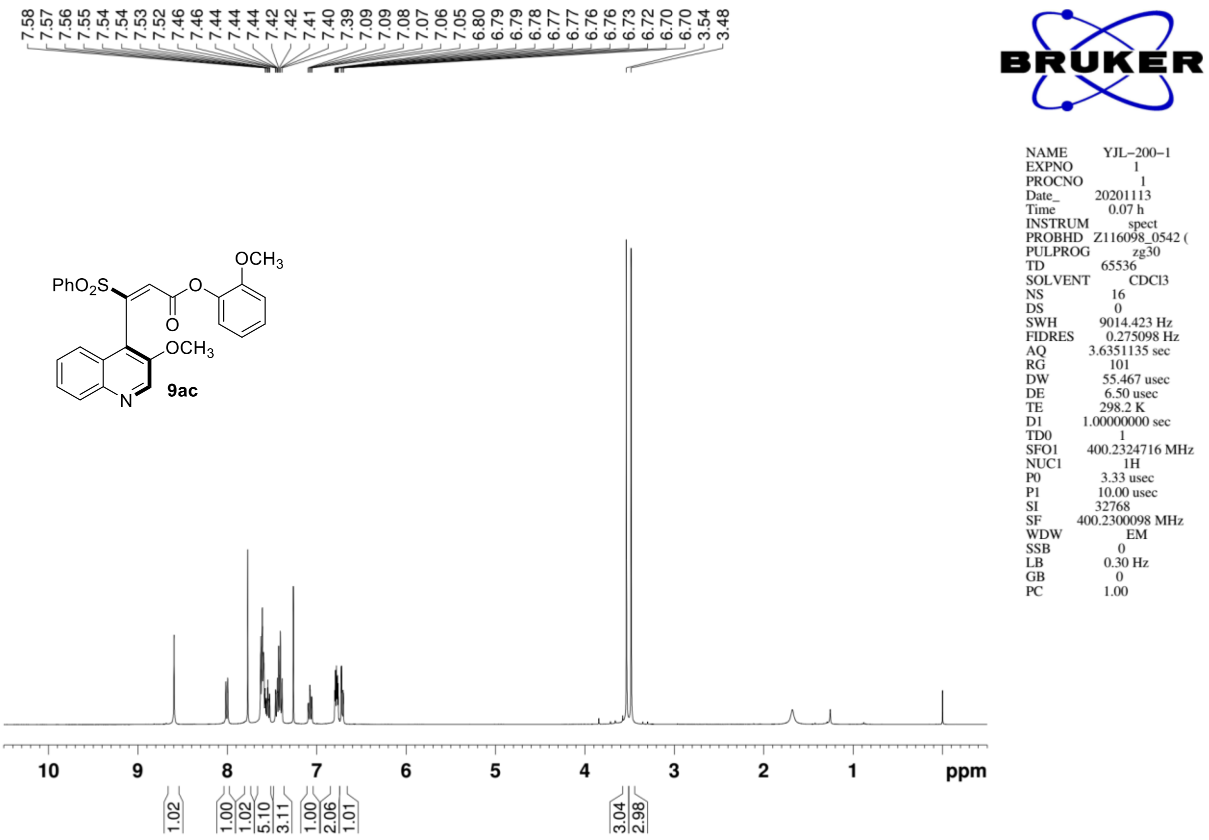

Supplementary Figure 139. ^{1}H NMR ($CDCl_3$, 400MHz) spectra of **9ac**

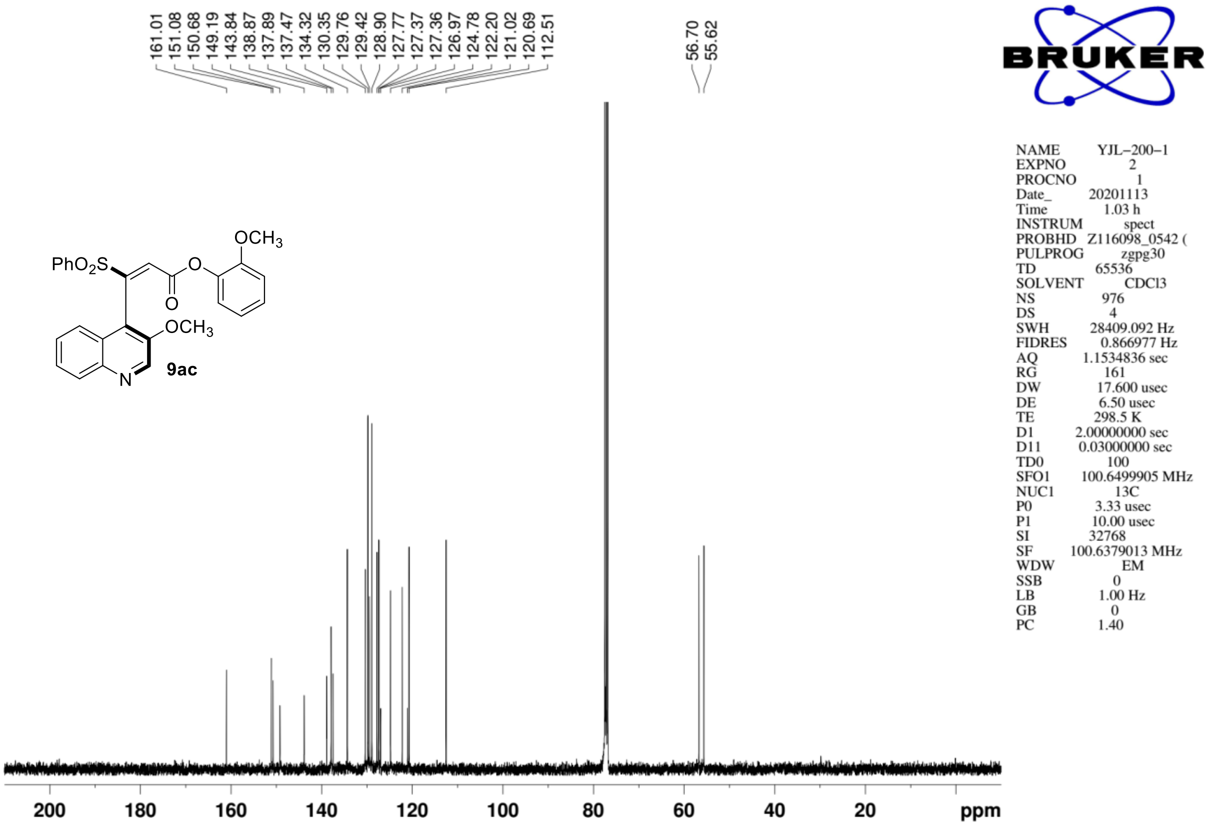

Supplementary Figure 140. ^{13}C NMR ($CDCl_3$, 101MHz) spectra of spectra of **9ac**

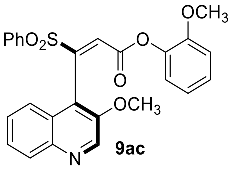

9ac

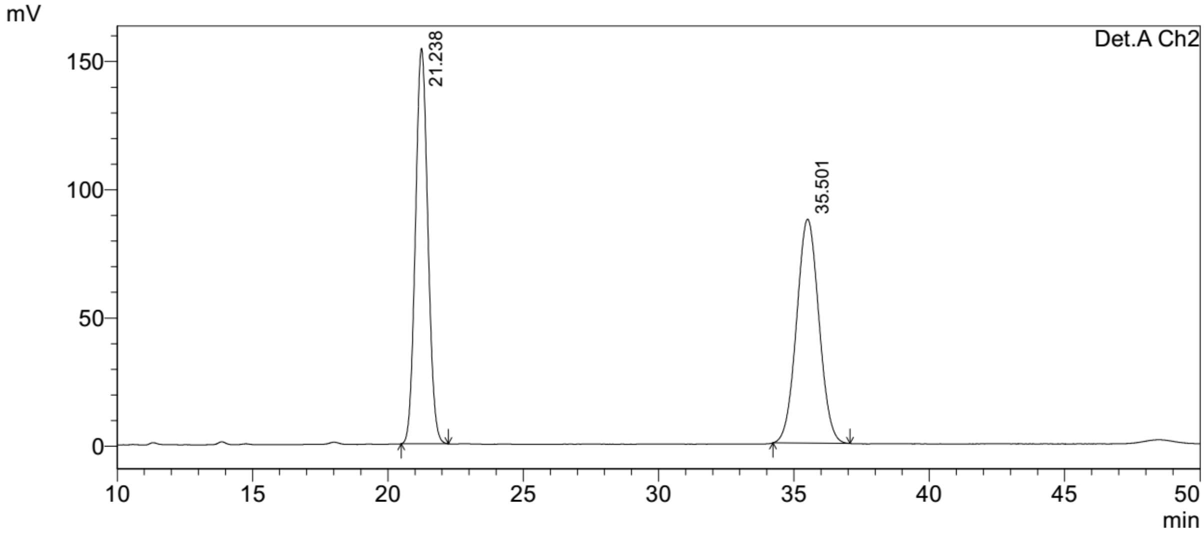

Detector A Ch2 220nm

Peak#	Ret. Time	Area	Height	Area %	Height %
1	21.238	4919969	154216	49.522	63.839
2	35.501	5014933	87356	50.478	36.161
Total		9934901	241572	100.000	100.000

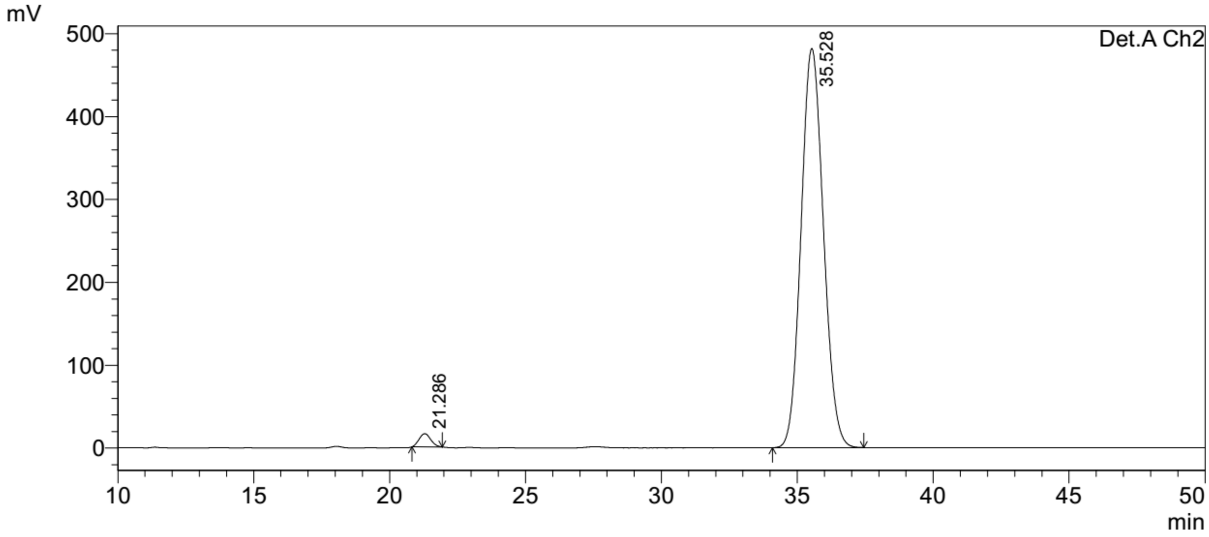

Detector A Ch2 220nm

Peak#	Ret. Time	Area	Height	Area %	Height %
1	21.286	476551	15997	1.700	3.214
2	35.528	27548852	481706	98.300	96.786
Total		28025404	497703	100.000	100.000

Supplementary Figure 141. HPLC spectra of **9ac**

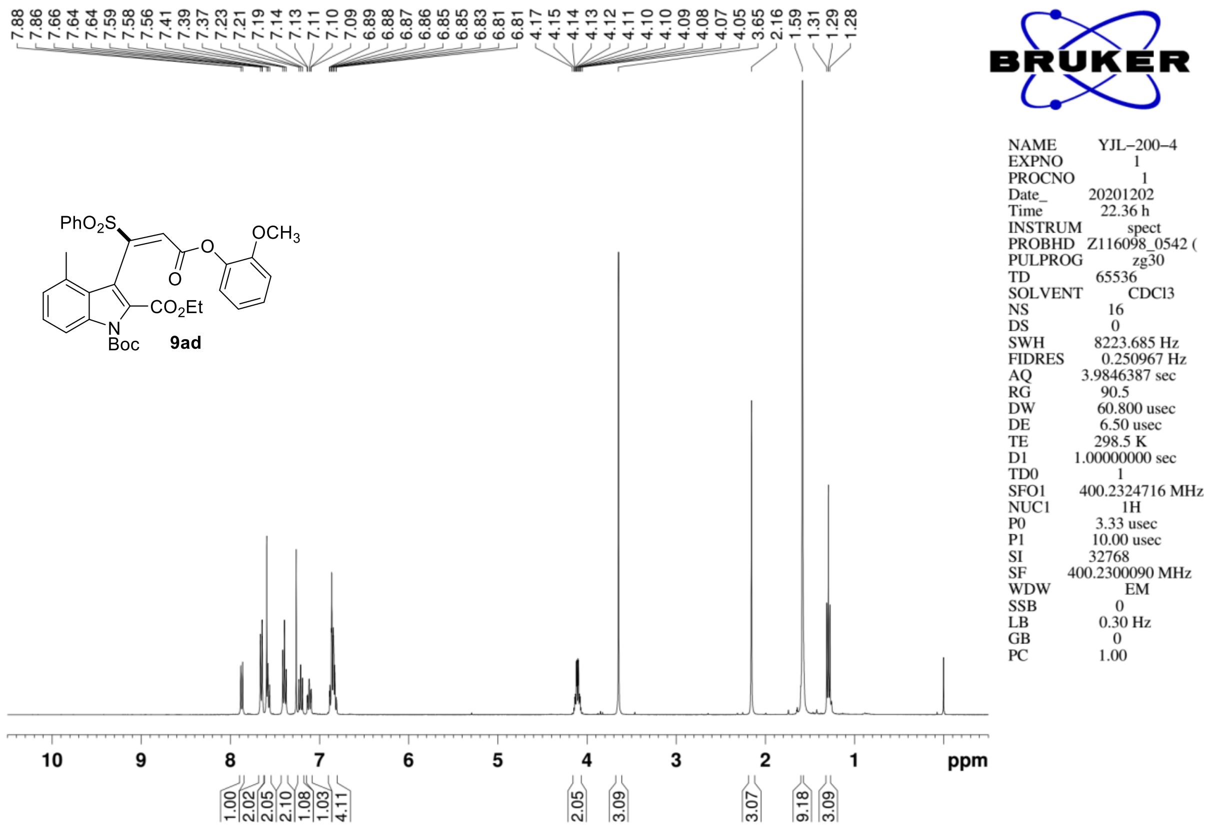

Supplementary Figure 142. ^{1}H NMR ($CDCl_3$, 400MHz) spectra of **9ad**

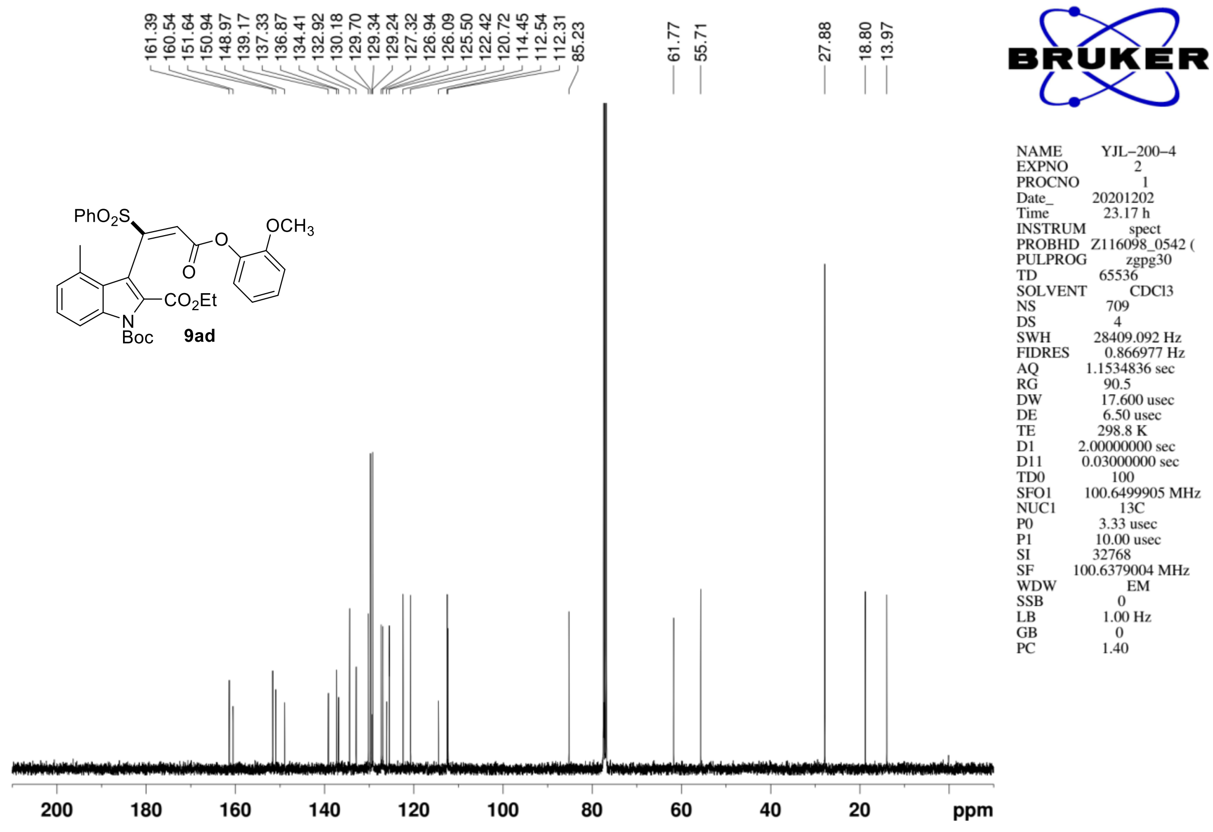

Supplementary Figure 143. ^{13}C NMR ($CDCl_3$, 101MHz) spectra of spectra of **9ad**

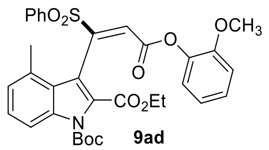

9ad

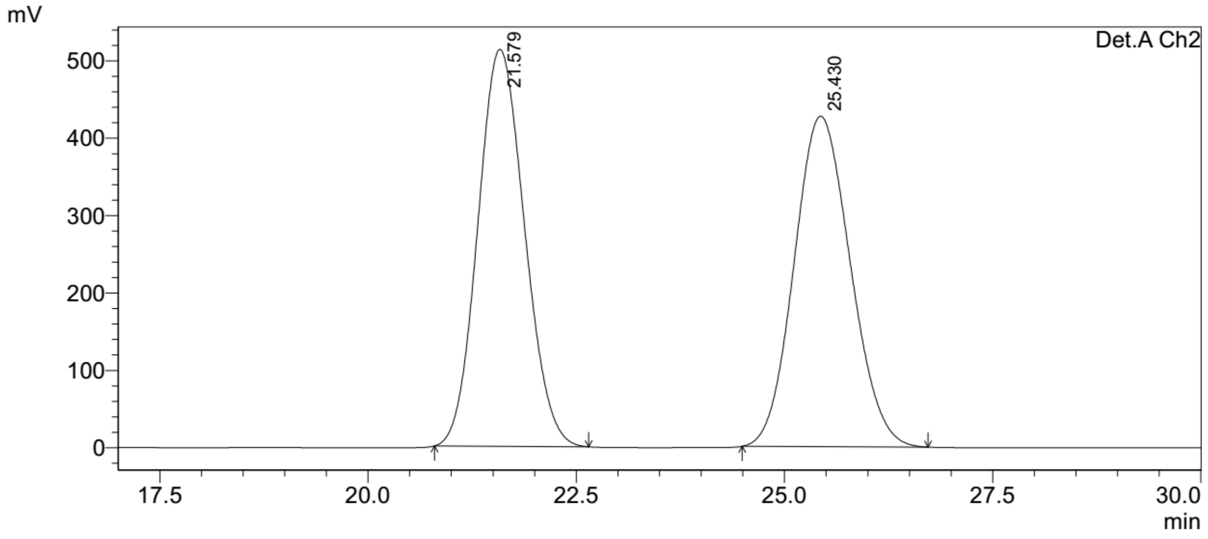

Detector A Ch2 220nm

Peak#	Ret. Time	Area	Height	Area %	Height %
1	21.579	19997232	512904	49.983	54.573
2	25.430	20011117	426940	50.017	45.427
Total		40008349	939844	100.000	100.000

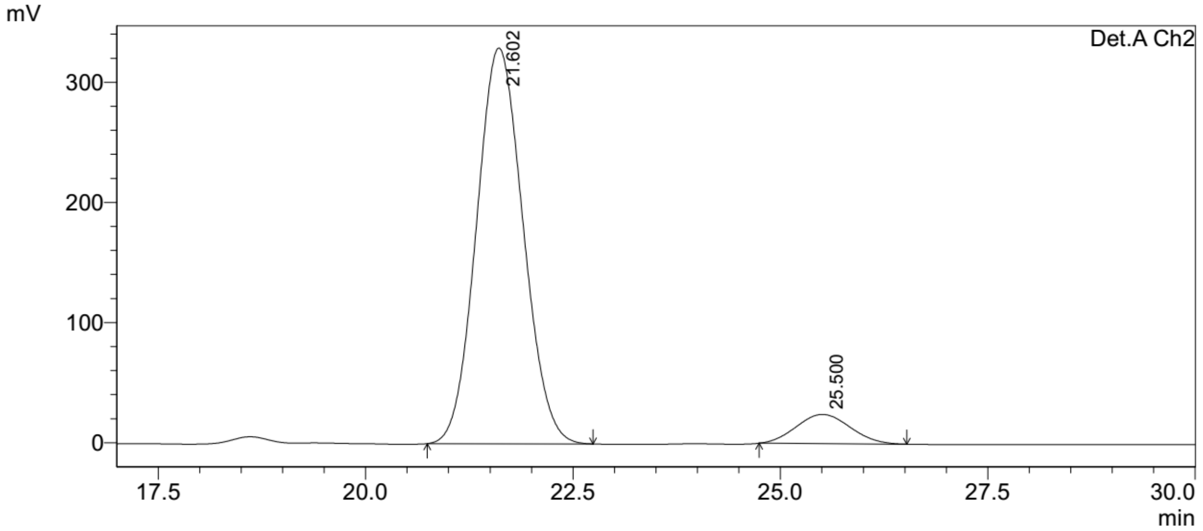

Detector A Ch2 220nm

Peak#	Ret. Time	Area	Height	Area %	Height %
1	21.602	12867957	329345	92.122	93.118
2	25.500	1100464	24341	7.878	6.882
Total		13968421	353686	100.000	100.000

Supplementary Figure 144. HPLC spectra of **9ad**

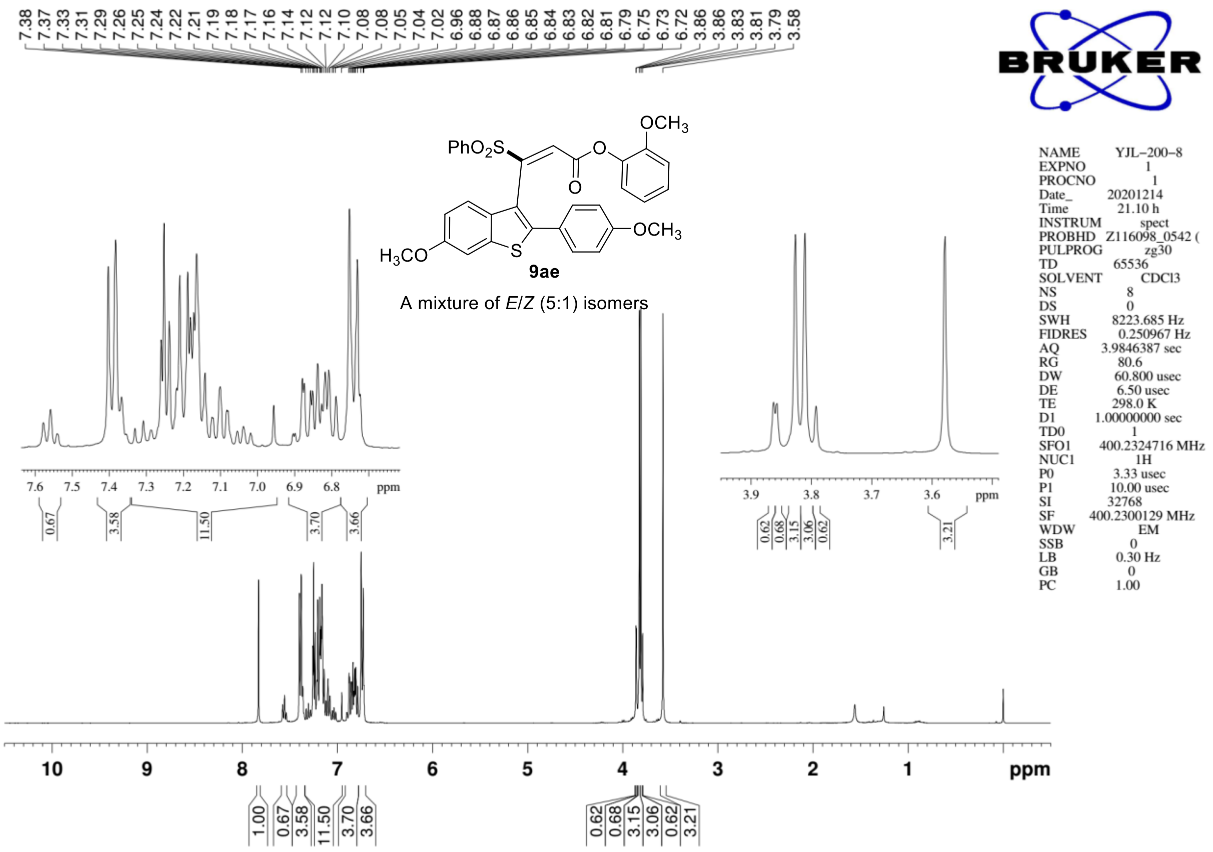

Supplementary Figure 145. ^{1}H NMR ($CDCl_3$, 400MHz) spectra of **9ae**

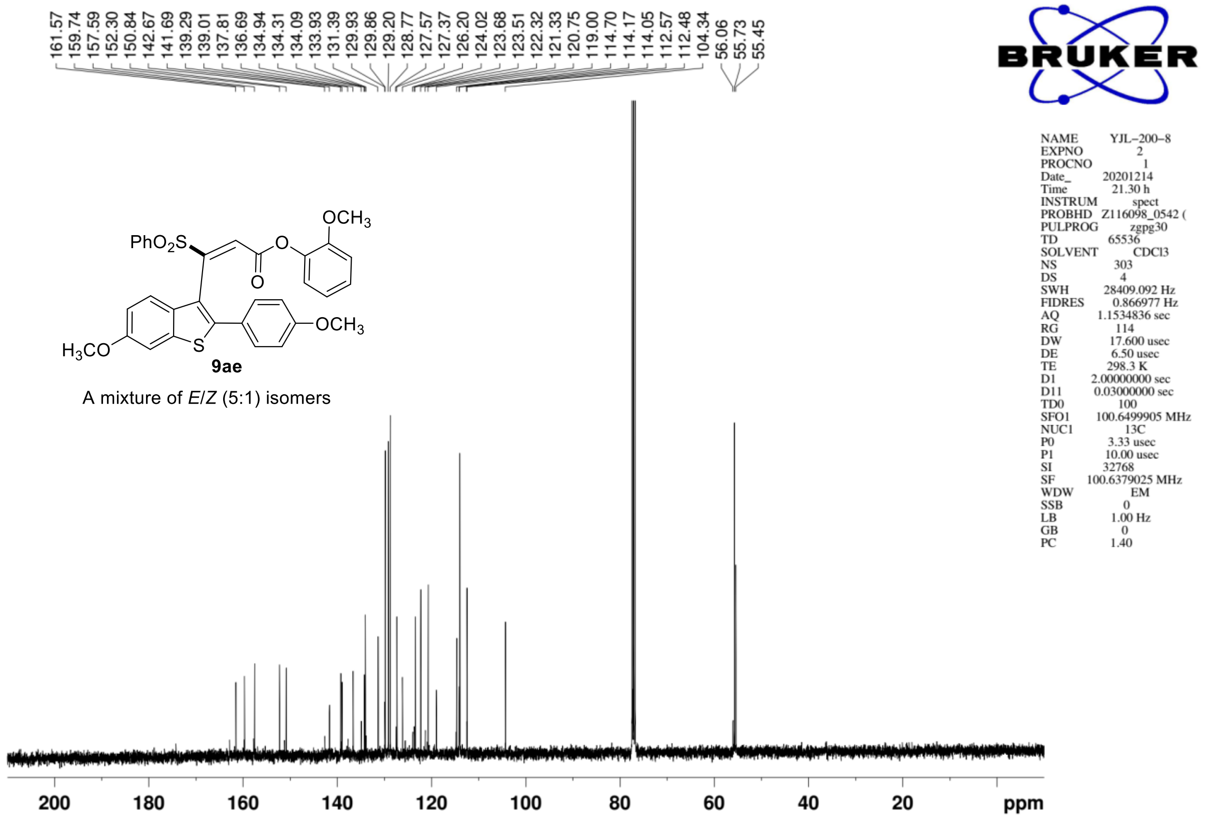

Supplementary Figure 146. ^{13}C NMR ($CDCl_3$, 101MHz) spectra of spectra of **9ae**

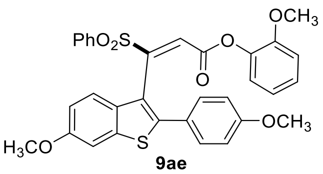

9ae
the *E* isomer

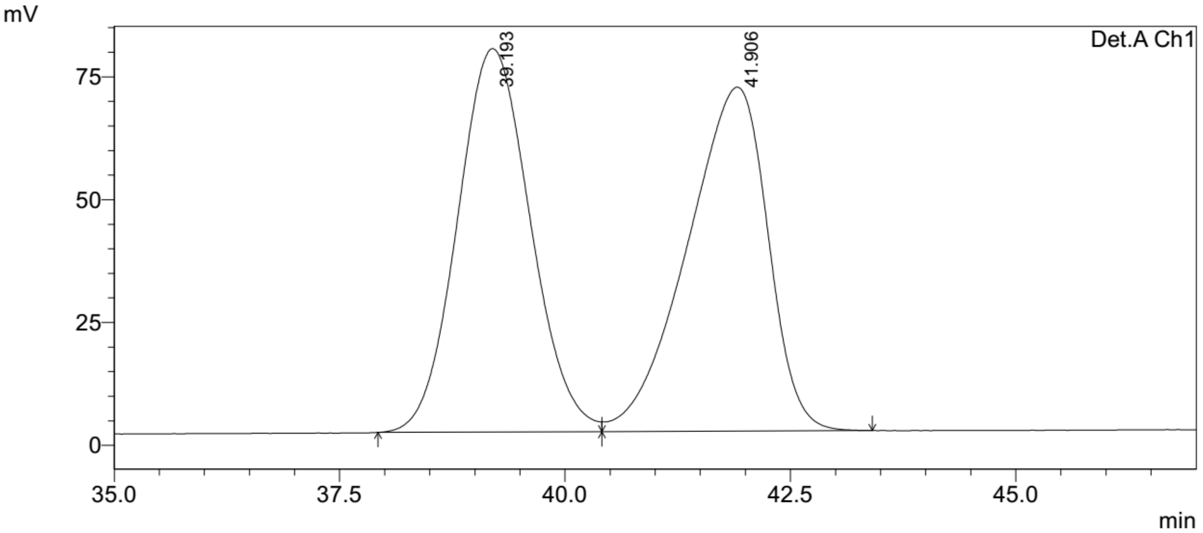

Detector A Ch1 254nm

Peak#	Ret. Time	Area	Height	Area %	Height %
1	39.193	4384570	78006	50.175	52.708
2	41.906	4353925	69990	49.825	47.292
Total		8738495	147996	100.000	100.000

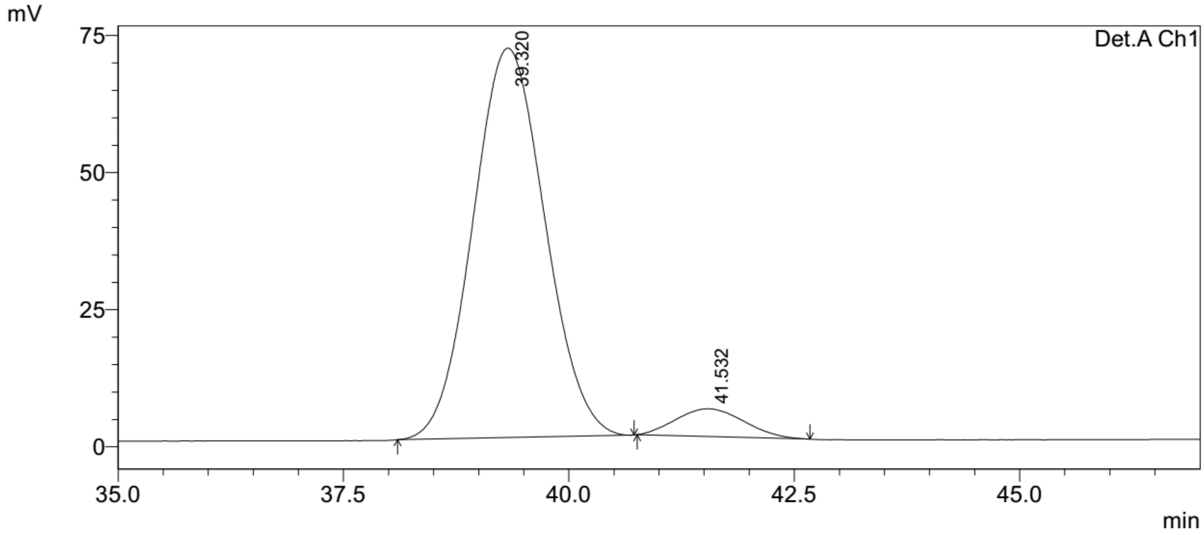

Detector A Ch1 254nm

Peak#	Ret. Time	Area	Height	Area %	Height %
1	39.320	3946373	70991	93.716	93.383
2	41.532	264596	5030	6.284	6.617
Total		4210969	76021	100.000	100.000

Supplementary Figure 147. HPLC spectra of **9ae**

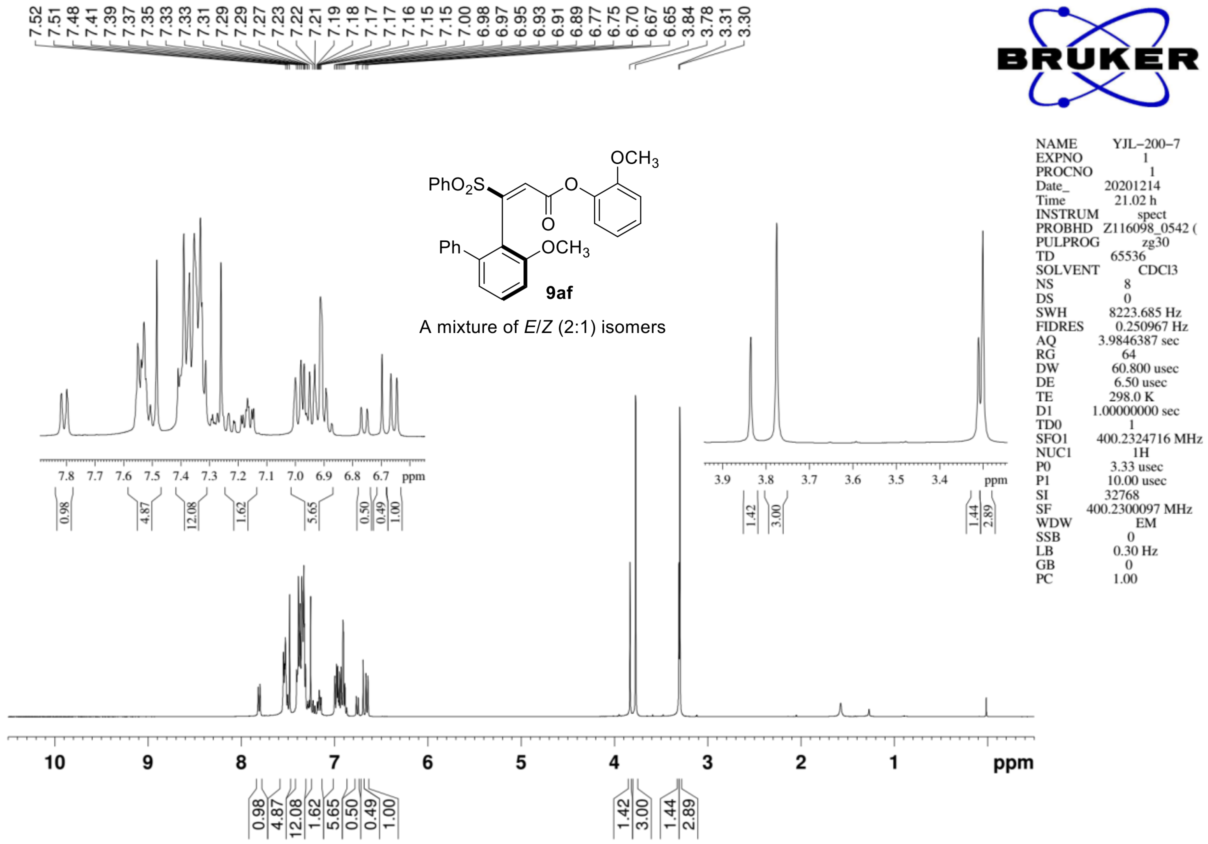

Supplementary Figure 148. ^{1}H NMR ($CDCl_3$, 400MHz) spectra of **9af**

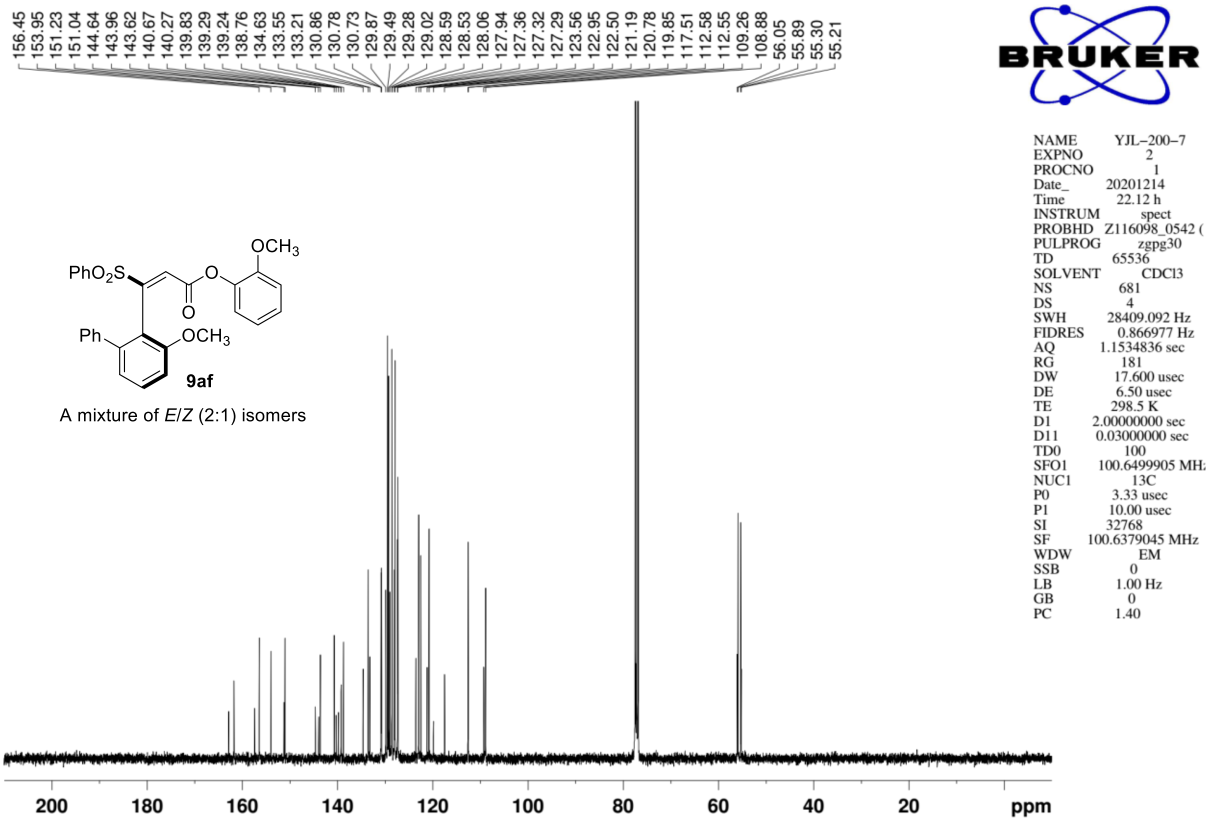

Supplementary Figure 149. ^{13}C NMR ($CDCl_3$, 101MHz) spectra of spectra of **9af**

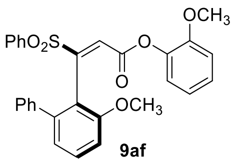

9af

the *E* isomer

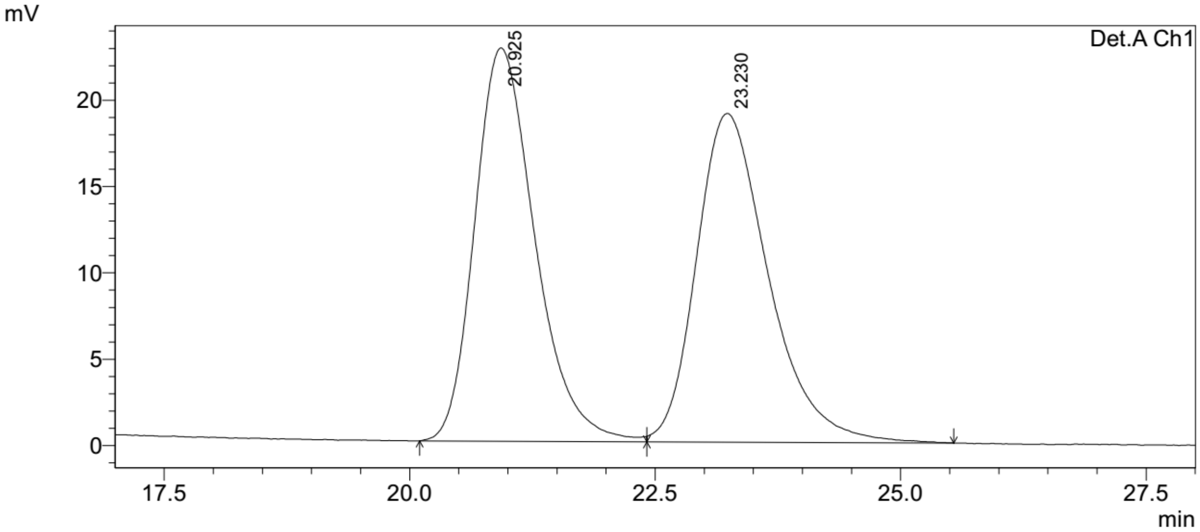

Detector A Ch1 254nm

Peak#	Ret. Time	Area	Height	Area %	Height %
1	20.925	970834	22764	50.024	54.478
2	23.230	969919	19022	49.976	45.522
Total		1940753	41786	100.000	100.000

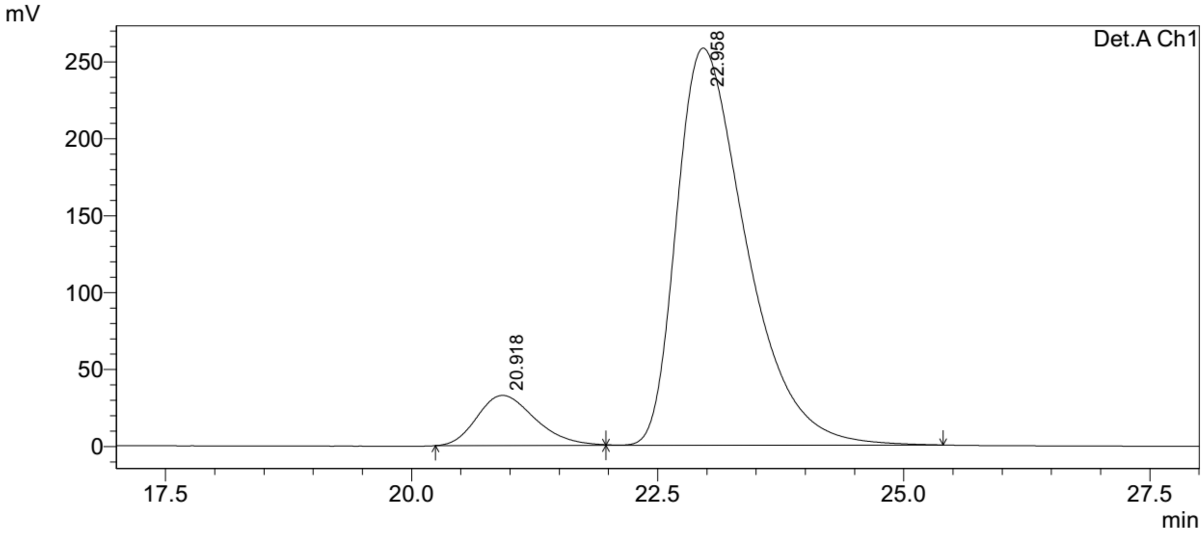

Detector A Ch1 254nm

Peak#	Ret. Time	Area	Height	Area %	Height %
1	20.918	1342419	32609	9.537	11.215
2	22.958	12733903	258158	90.463	88.785
Total		14076322	290767	100.000	100.000

Supplementary Figure 150. HPLC spectra of **9af**

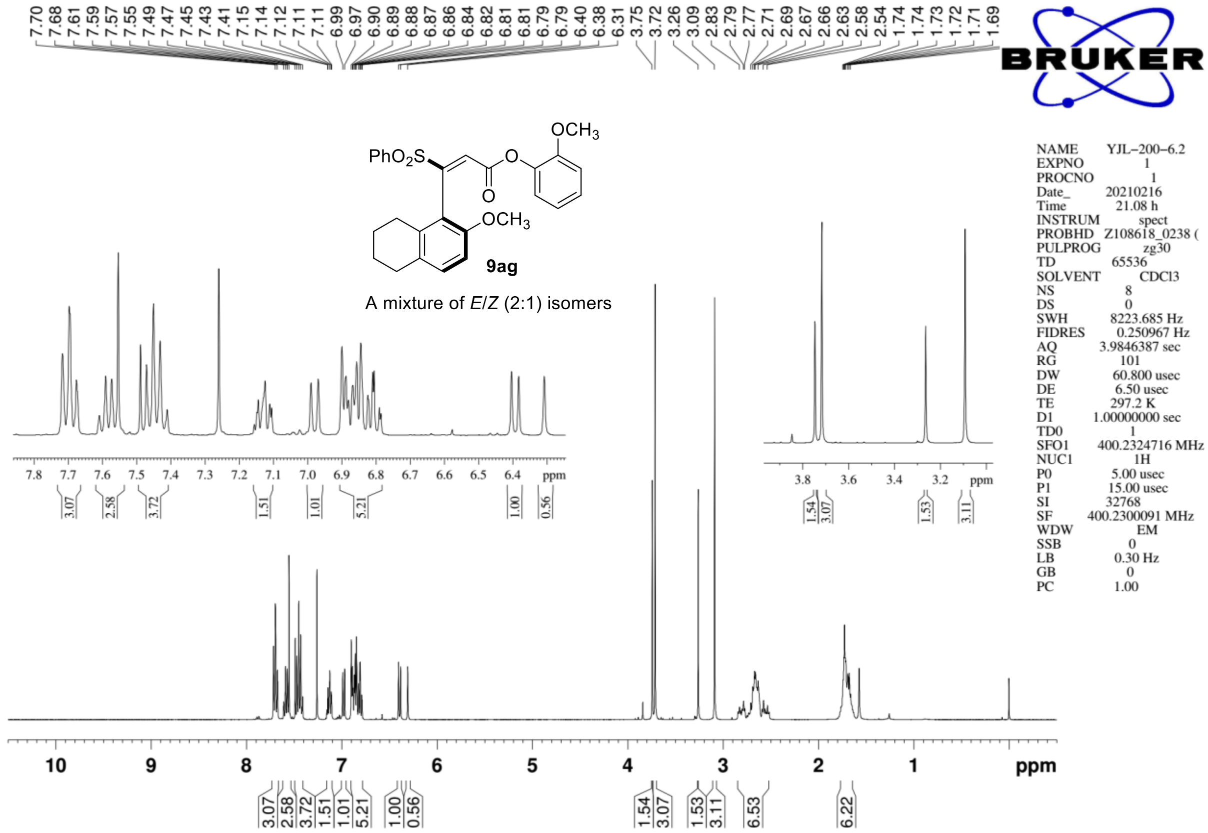

Supplementary Figure 151. ^{1}H NMR ($CDCl_3$, 400MHz) spectra of **9ag**

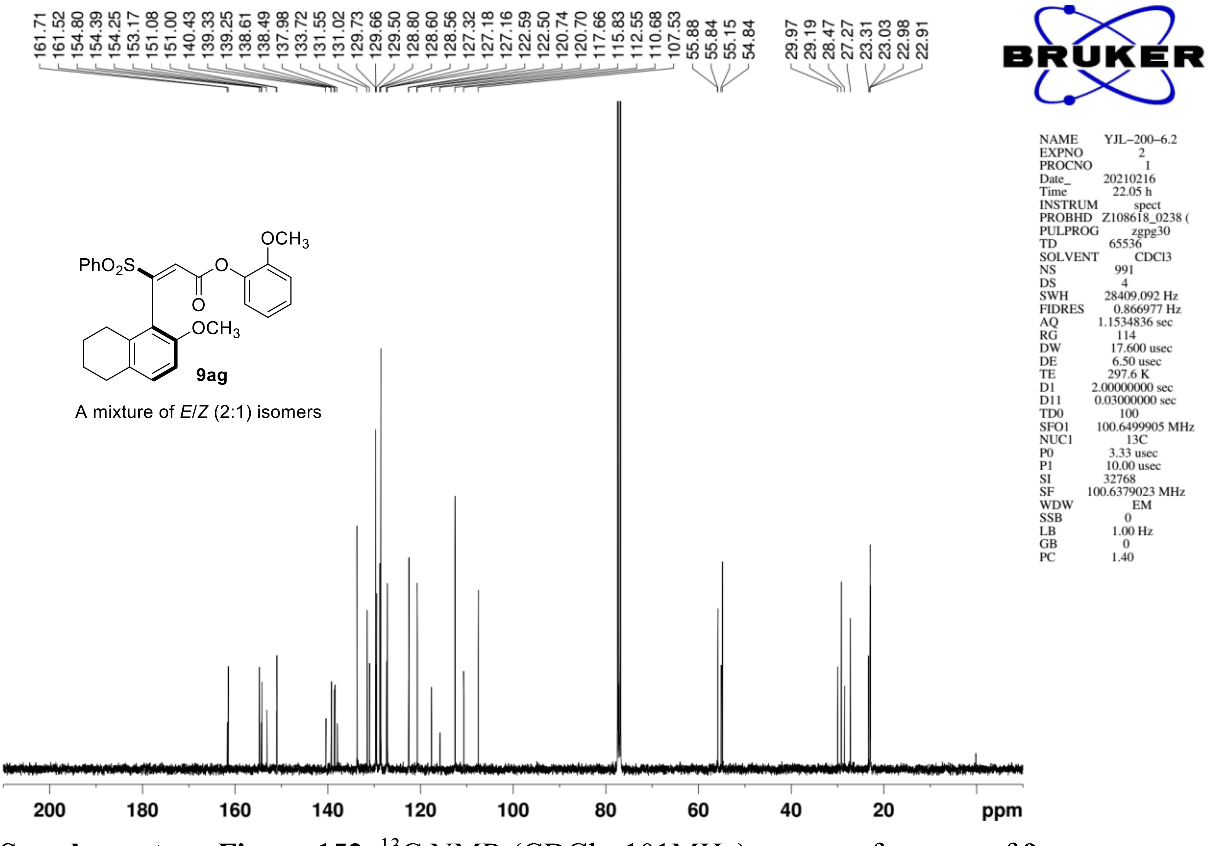

Supplementary Figure 152. ^{13}C NMR ($CDCl_3$, 101MHz) spectra of spectra of **9ag**

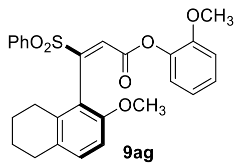

9ag

the *E* isomer

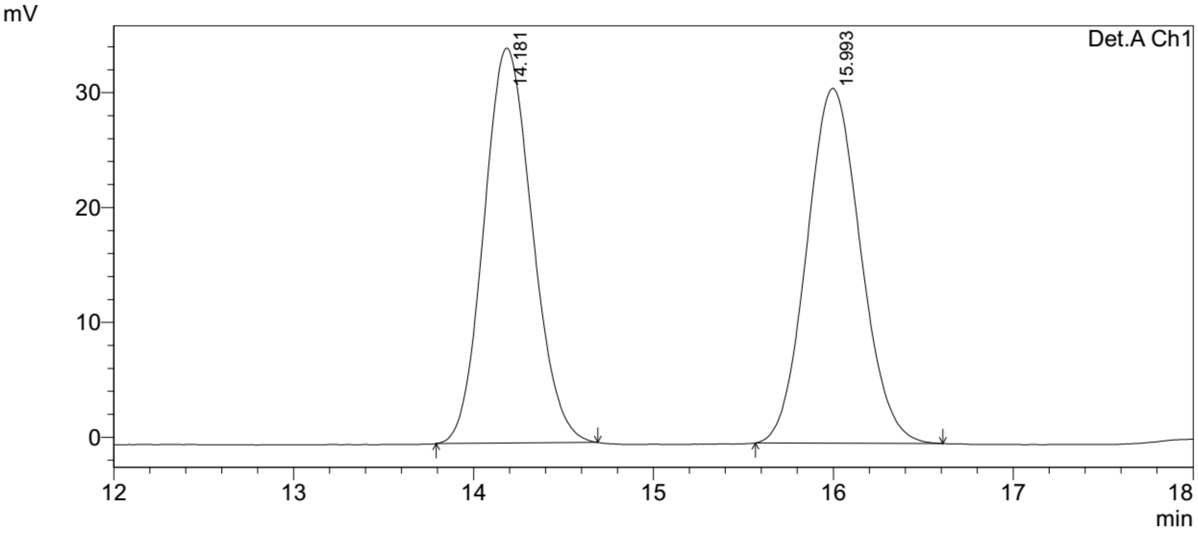

Detector A Ch1 254nm

Peak#	Ret. Time	Area	Height	Area %	Height %
1	14.181	642763	34390	50.051	52.681
2	15.993	641446	30890	49.949	47.319
Total		1284209	65281	100.000	100.000

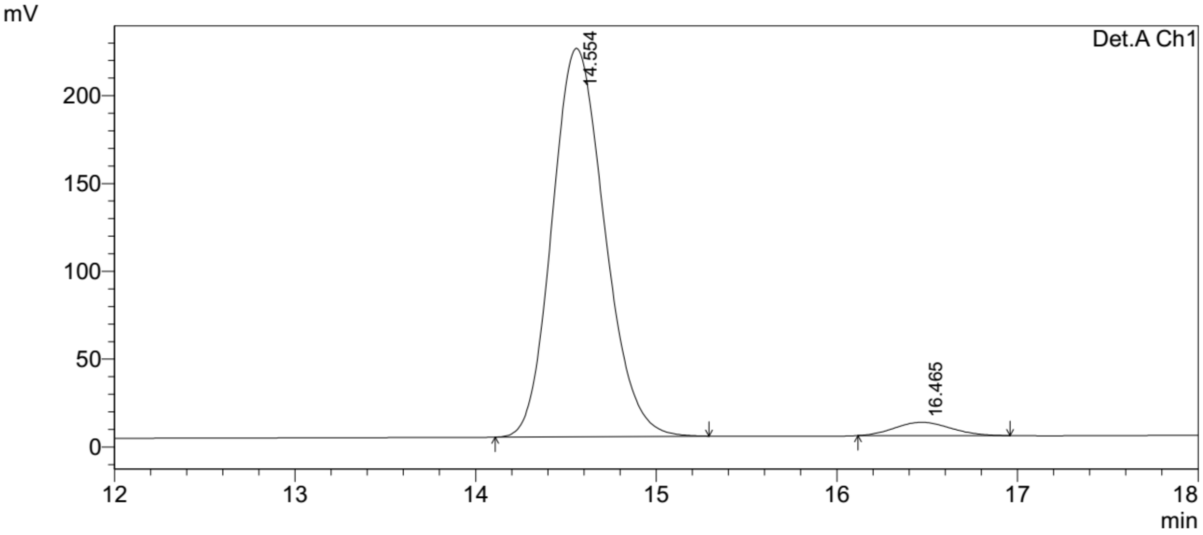

Detector A Ch1 254nm

Peak#	Ret. Time	Area	Height	Area %	Height %
1	14.554	4466088	221246	96.480	96.687
2	16.465	162949	7581	3.520	3.313
Total		4629037	228827	100.000	100.000

Supplementary Figure 153. HPLC spectra of **9ag**

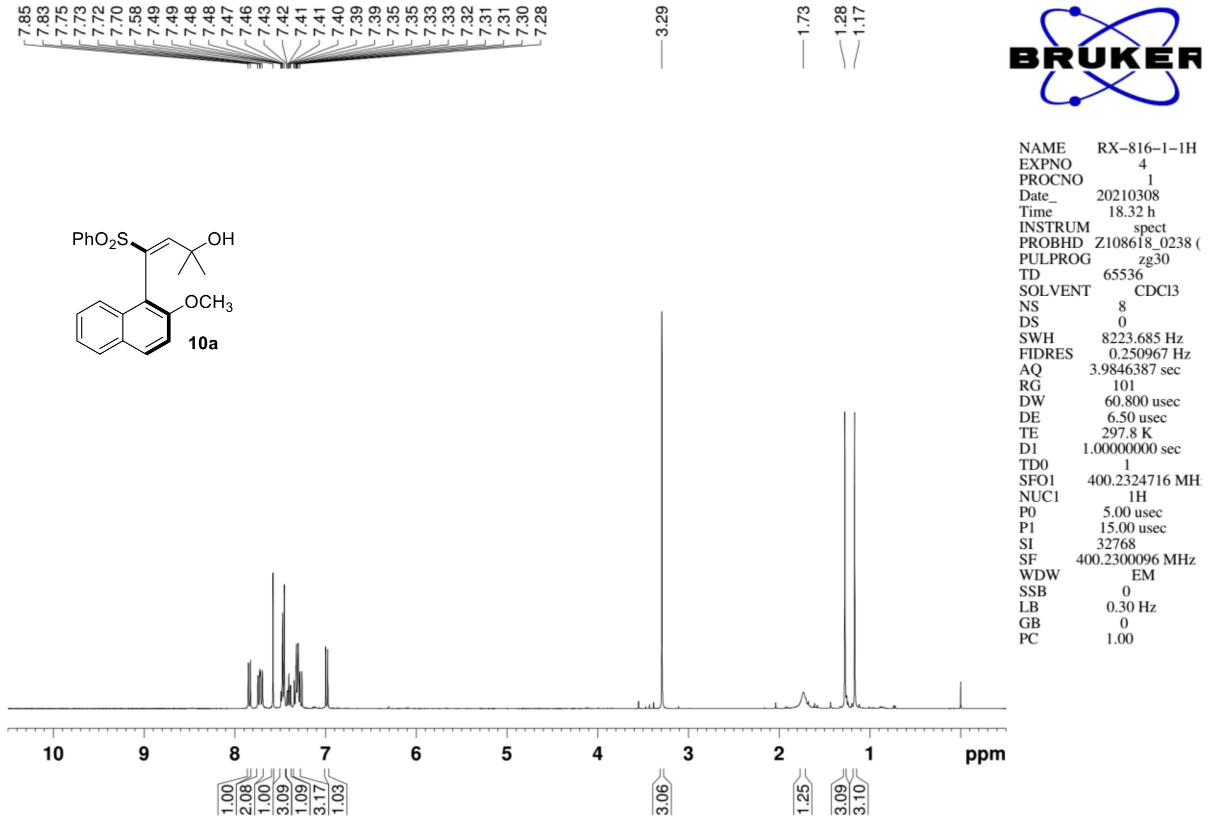

Supplementary Figure 154. ^{1}H NMR ($CDCl_3$, 400MHz) spectra of **10a**

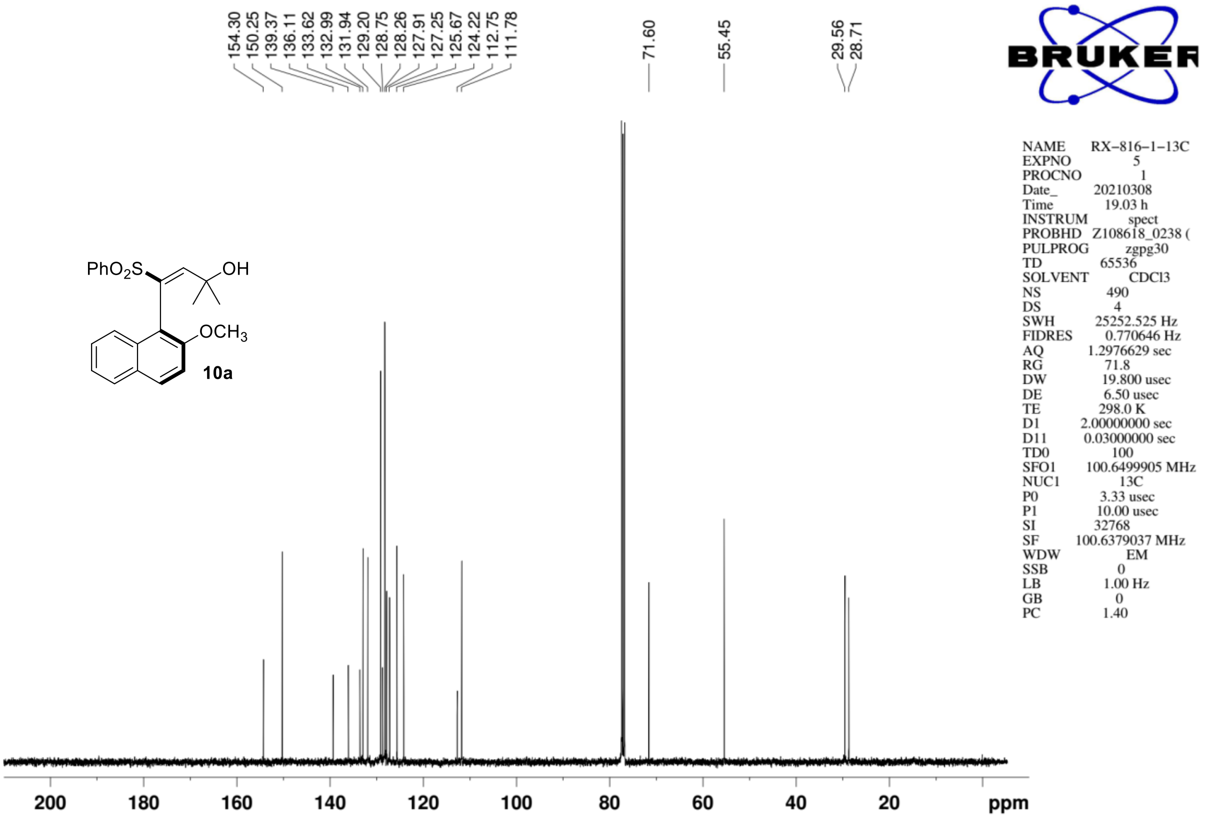

Supplementary Figure 155. ^{13}C NMR ($CDCl_3$, 101MHz) spectra of spectra of **10a**

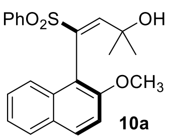

10a

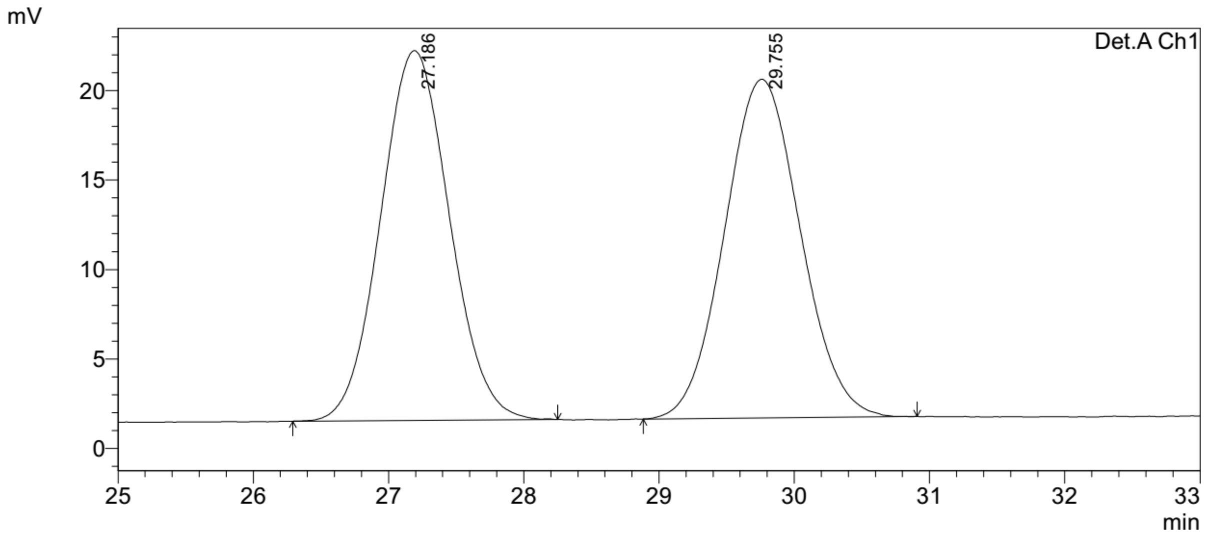

Detector A Ch1 254nm

Peak#	Ret. Time	Area	Height	Area %	Height %
1	27.186	730925	20667	49.771	52.190
2	29.755	737637	18932	50.229	47.810
Total		1468562	39599	100.000	100.000

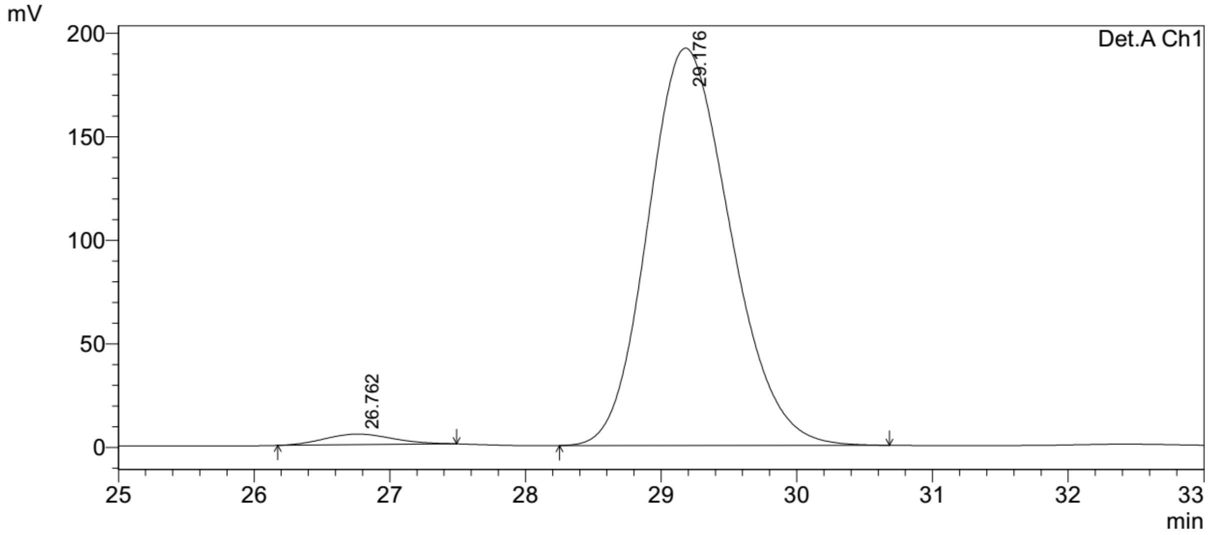

Detector A Ch1 254nm

Peak#	Ret. Time	Area	Height	Area %	Height %
1	26.762	179480	5111	2.162	2.596
2	29.176	8121393	191818	97.838	97.404
Total		8300873	196930	100.000	100.000

Supplementary Figure 156. HPLC spectra of **10a**

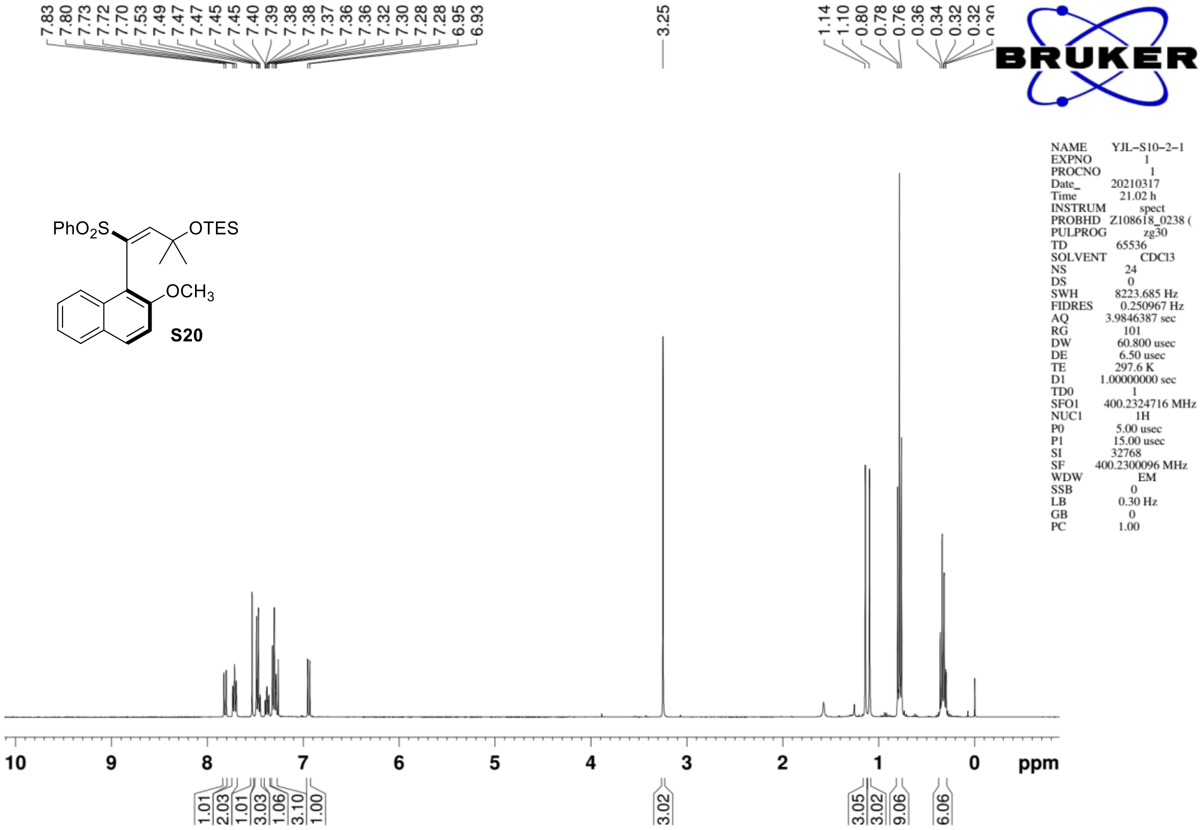

Supplementary Figure 157. ^{1}H NMR ($CDCl_3$, 400MHz) spectra of **S20**

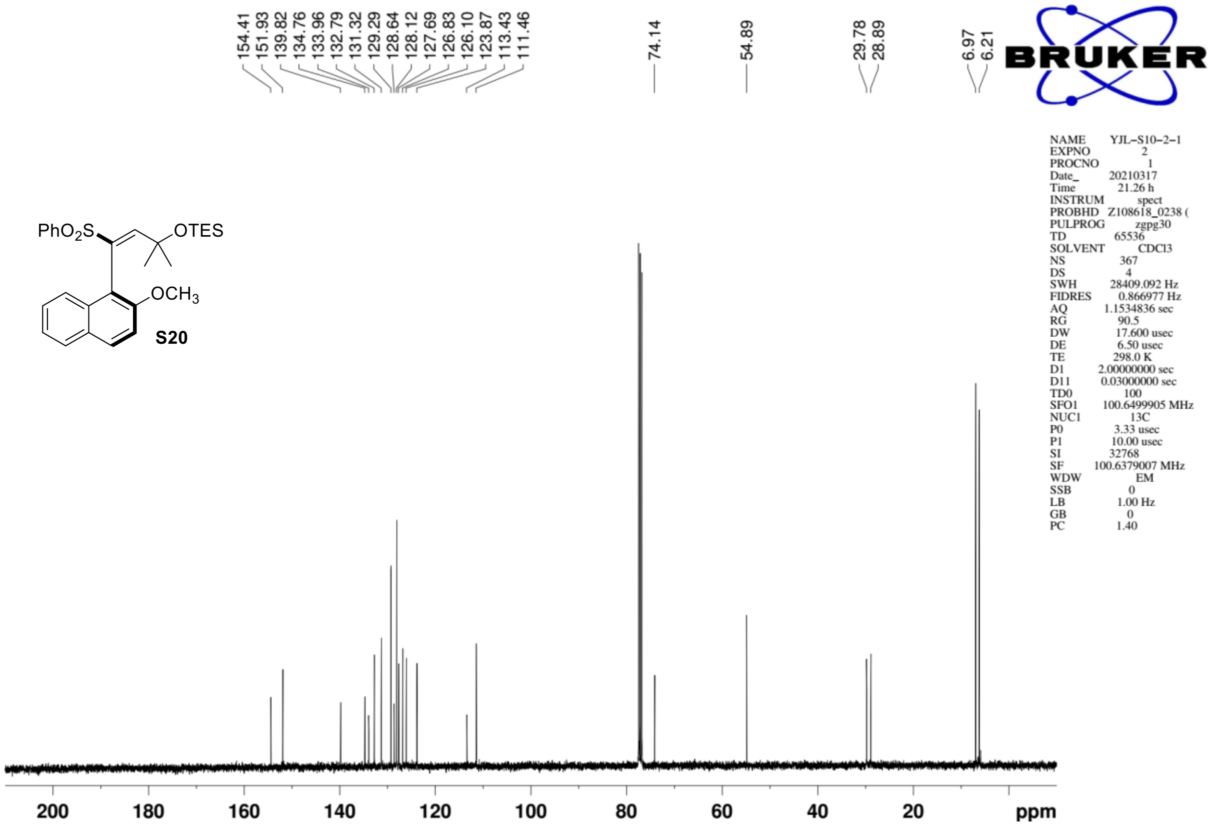

Supplementary Figure 158. ^{13}C NMR ($CDCl_3$, 101MHz) spectra of spectra of **S20**

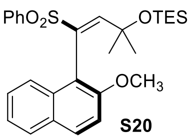

S20

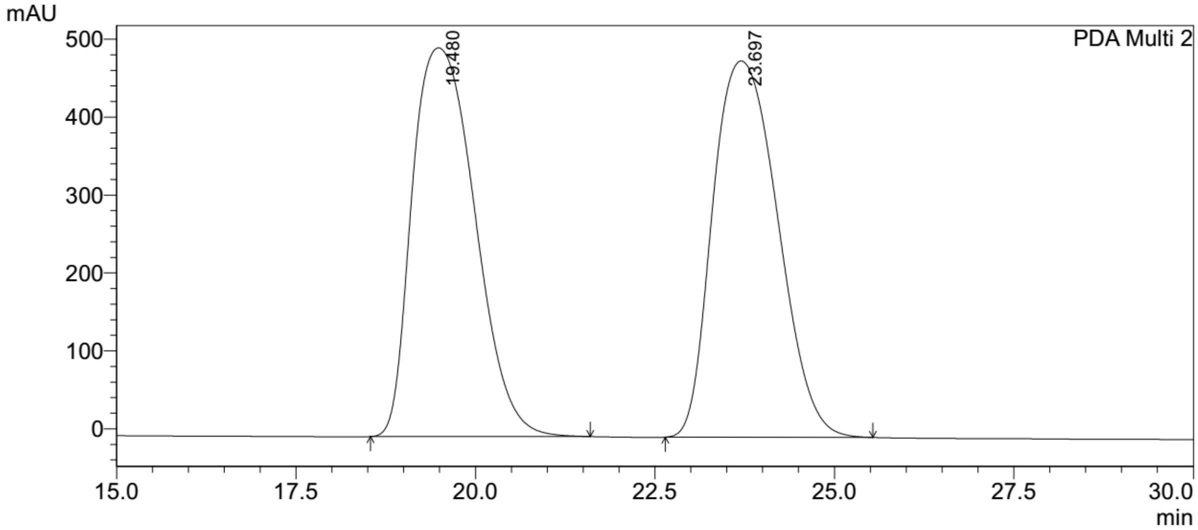

PDA Ch2 220nm 4nm

Peak#	Ret. Time	Area	Height	Area %	Height %
1	19.480	30427776	498834	49.976	50.810
2	23.697	30456486	482924	50.024	49.190
Total		60884262	981758	100.000	100.000

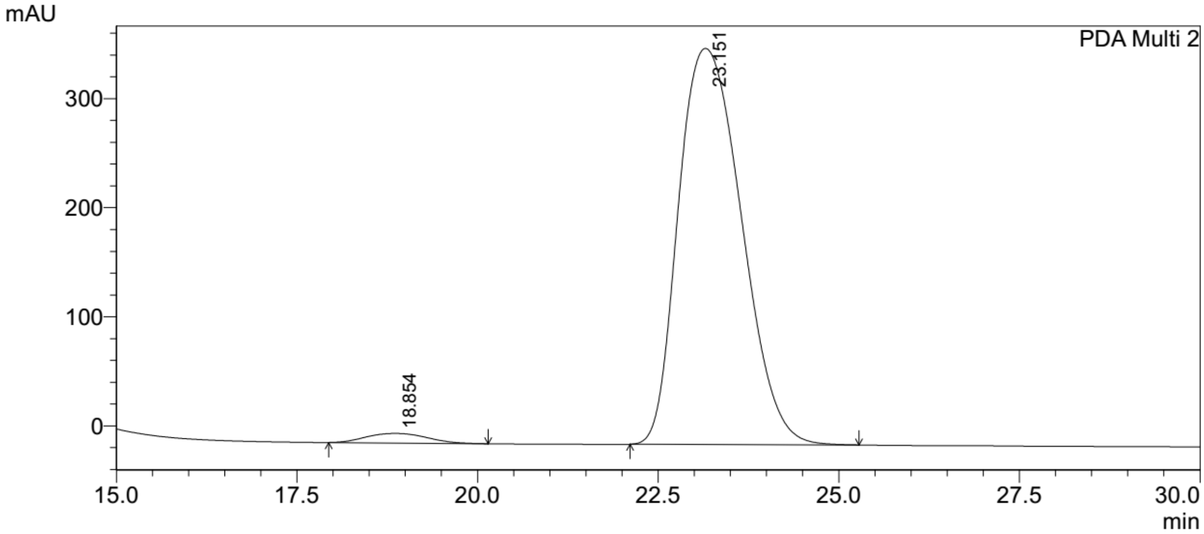

PDA Ch2 220nm 4nm

Peak#	Ret. Time	Area	Height	Area %	Height %
1	18.854	515361	8788	2.282	2.362
2	23.151	22067631	363178	97.718	97.638
Total		22582992	371965	100.000	100.000

Supplementary Figure 159. HPLC spectra of **S20**

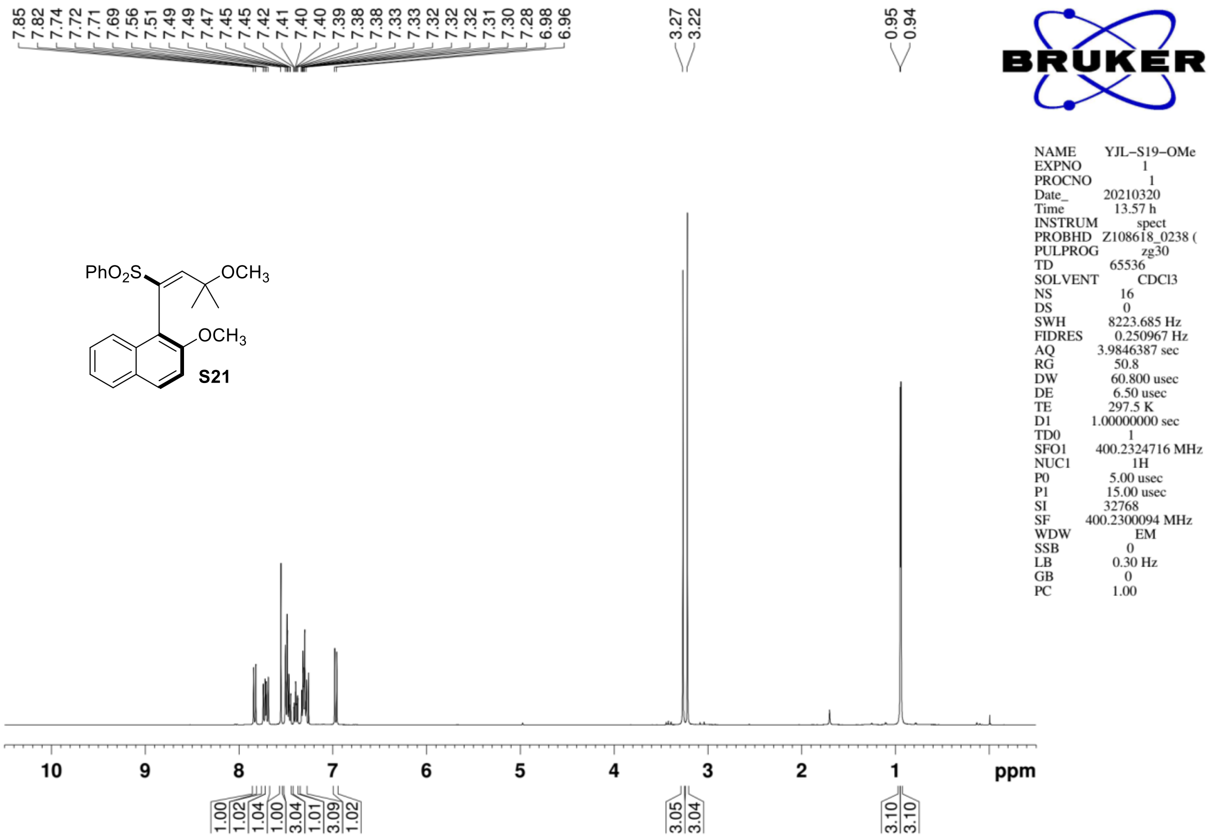

Supplementary Figure 160. ^{1}H NMR ($CDCl_3$, 400MHz) spectra of **S21**

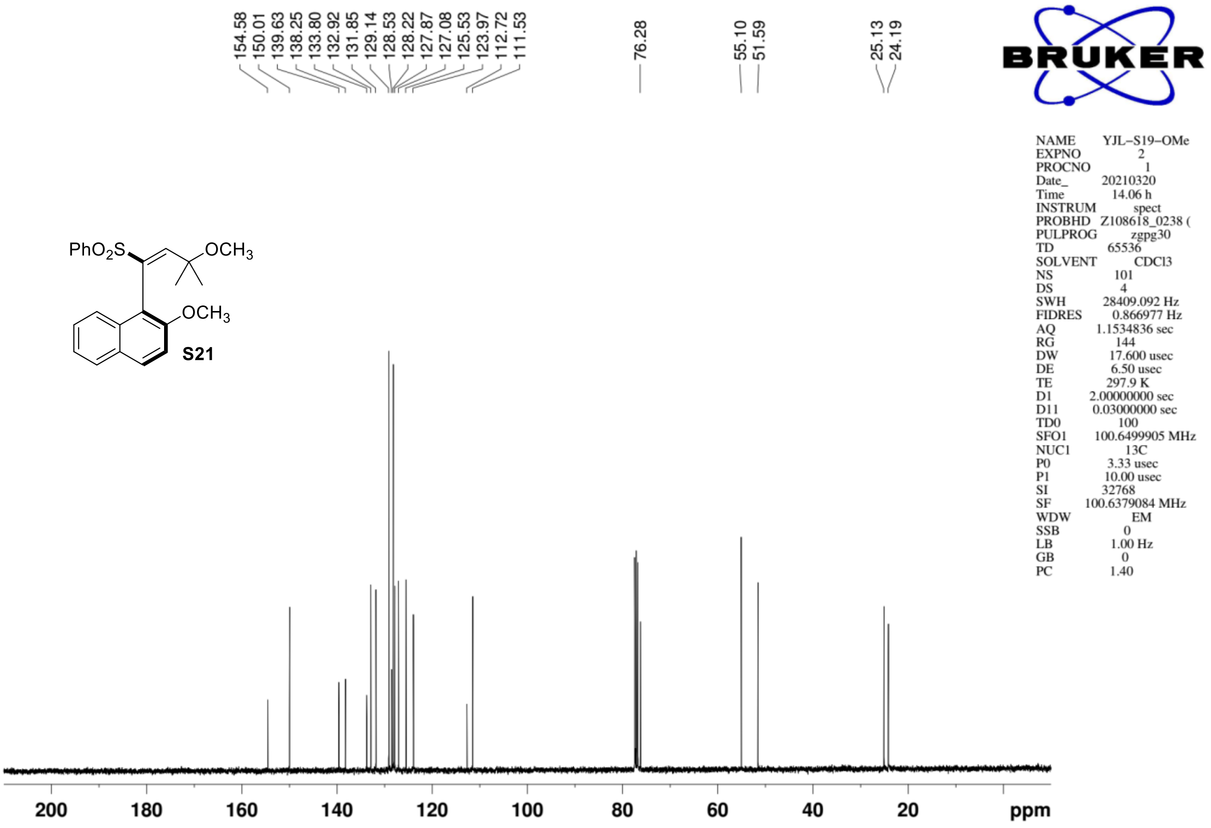

Supplementary Figure 161. ^{13}C NMR ($CDCl_3$, 101MHz) spectra of spectra of **S21**

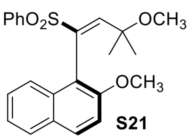

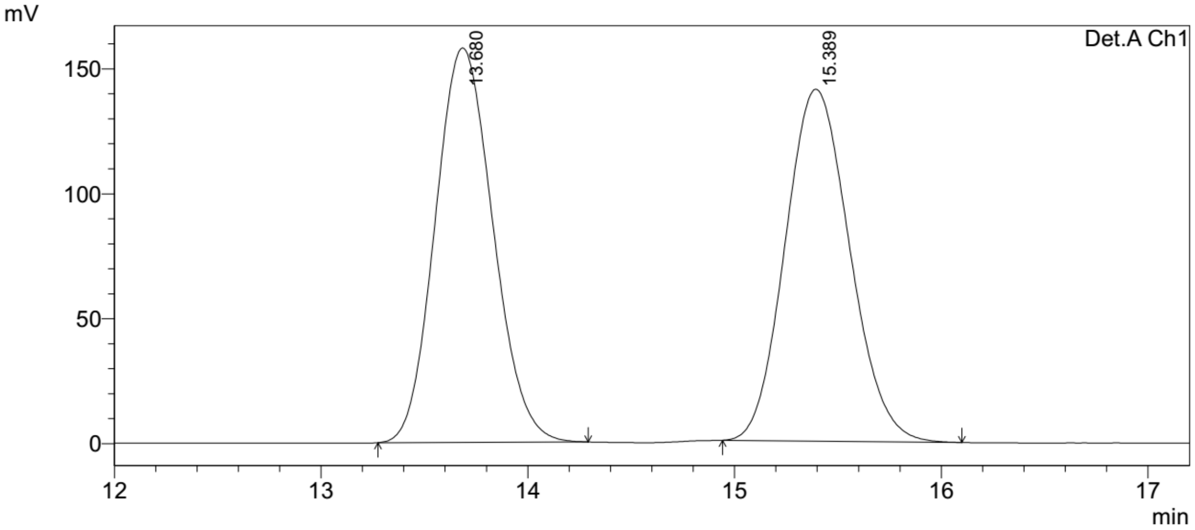

Detector A Ch1 254nm

Peak#	Ret. Time	Area	Height	Area %	Height %
1	13.680	3067873	157939	50.094	52.850
2	15.389	3056333	140907	49.906	47.150
Total		6124207	298846	100.000	100.000

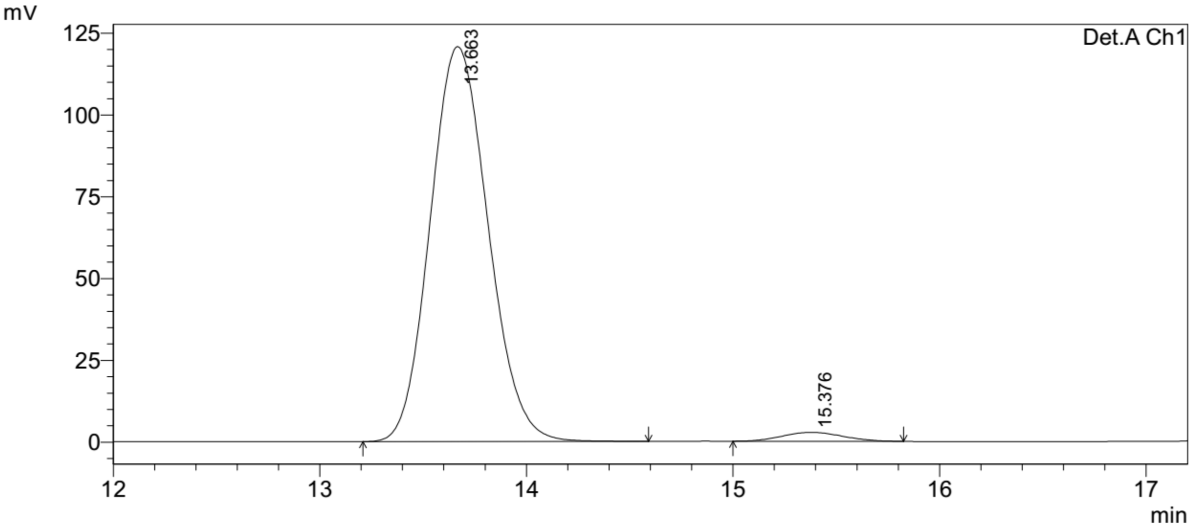

Detector A Ch1 254nm

Peak#	Ret. Time	Area	Height	Area %	Height %
1	13.663	2354180	120773	97.655	97.772
2	15.376	56526	2752	2.345	2.228
Total		2410706	123525	100.000	100.000

Supplementary Figure 162. HPLC spectra of **S21**

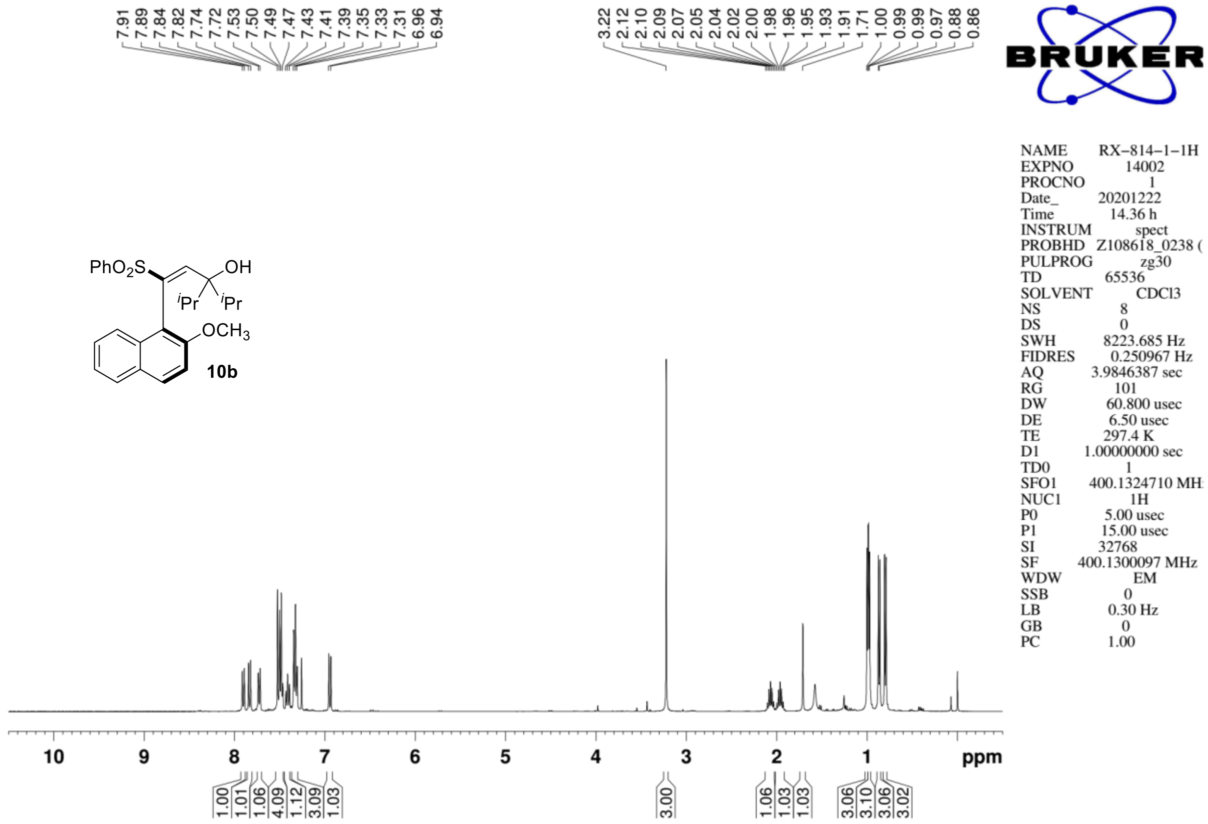

Supplementary Figure 163. ^{1}H NMR ($CDCl_3$, 400MHz) spectra of **10b**

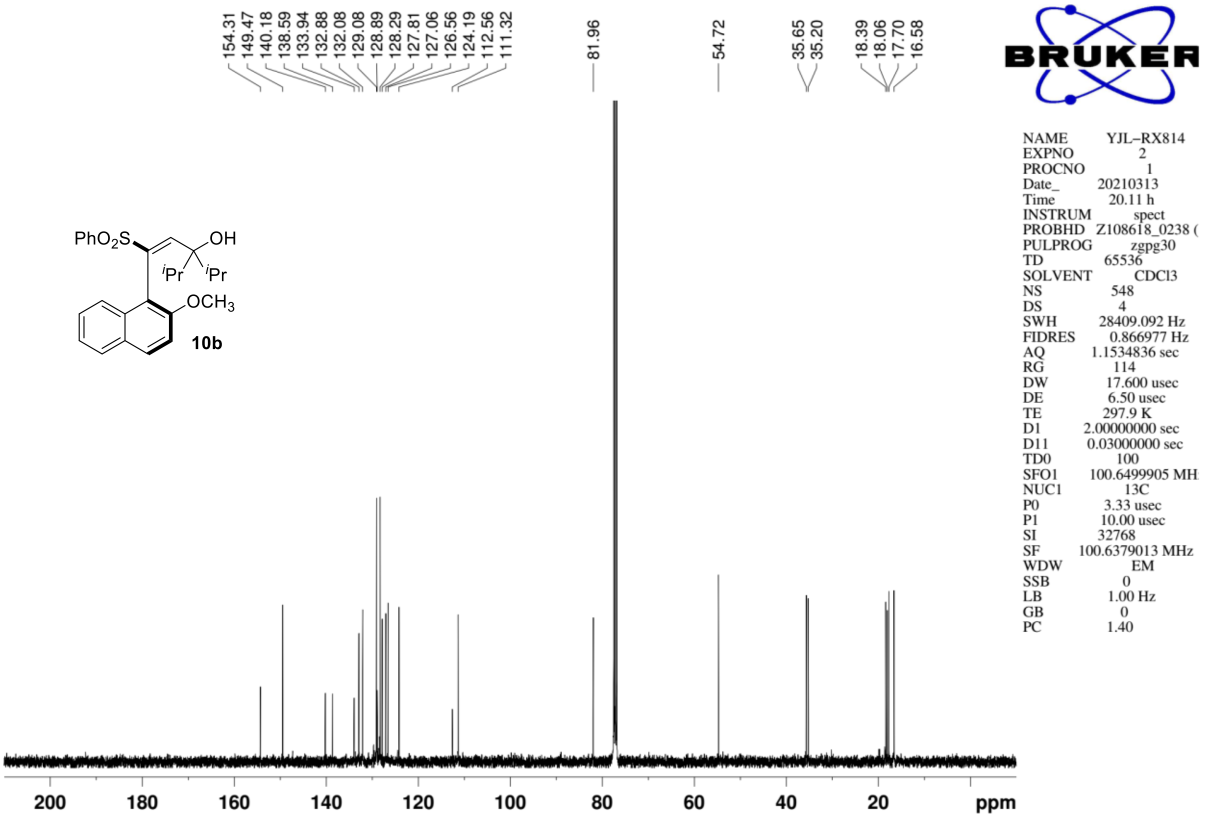

Supplementary Figure 164. ^{13}C NMR ($CDCl_3$, 101MHz) spectra of spectra of **10b**

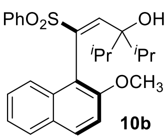

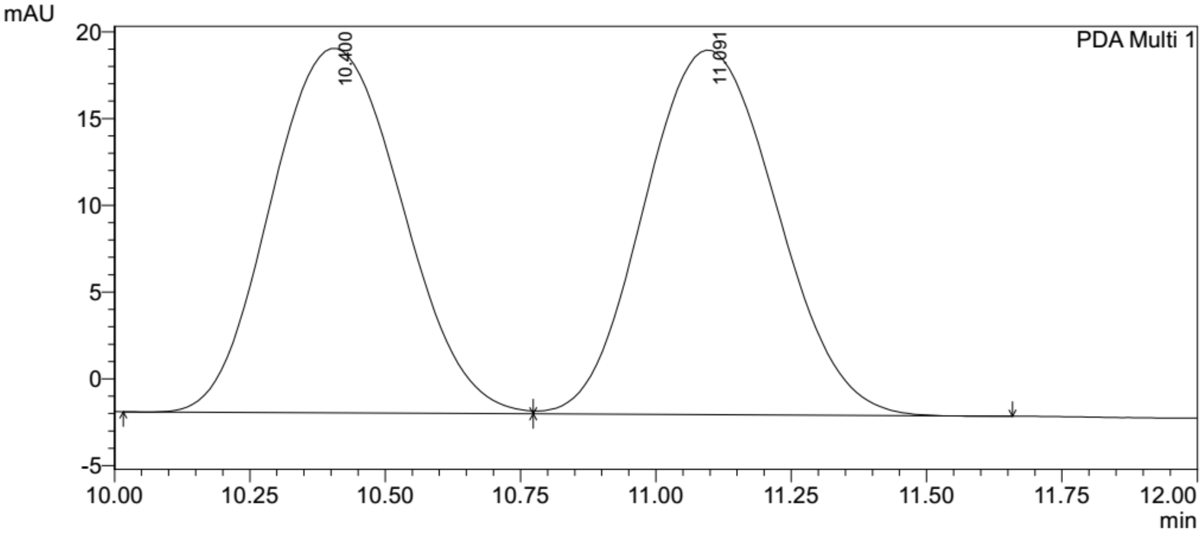

PDA Ch1 254nm 4nm

Peak#	Ret. Time	Area	Height	Area %	Height %
1	10.400	351859	20981	49.692	49.979
2	11.091	356224	20999	50.308	50.021
Total		708083	41980	100.000	100.000

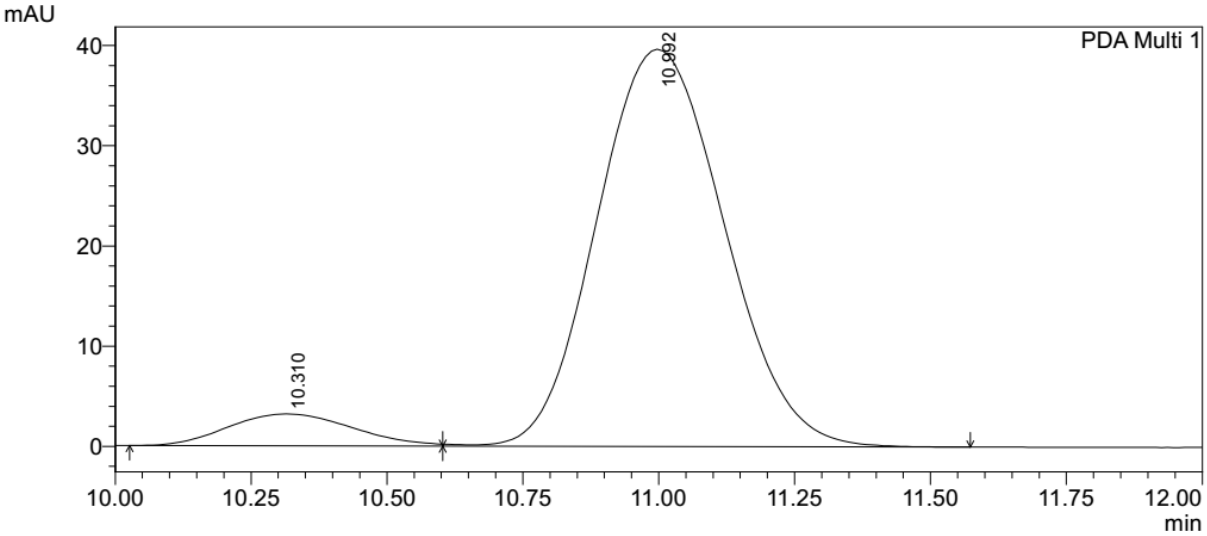

PDA Ch1 254nm 4nm

Peak#	Ret. Time	Area	Height	Area %	Height %
1	10.310	50661	3175	7.282	7.415
2	10.992	645087	39648	92.718	92.585
Total		695748	42823	100.000	100.000

Supplementary Figure 165. HPLC spectra of **10b**

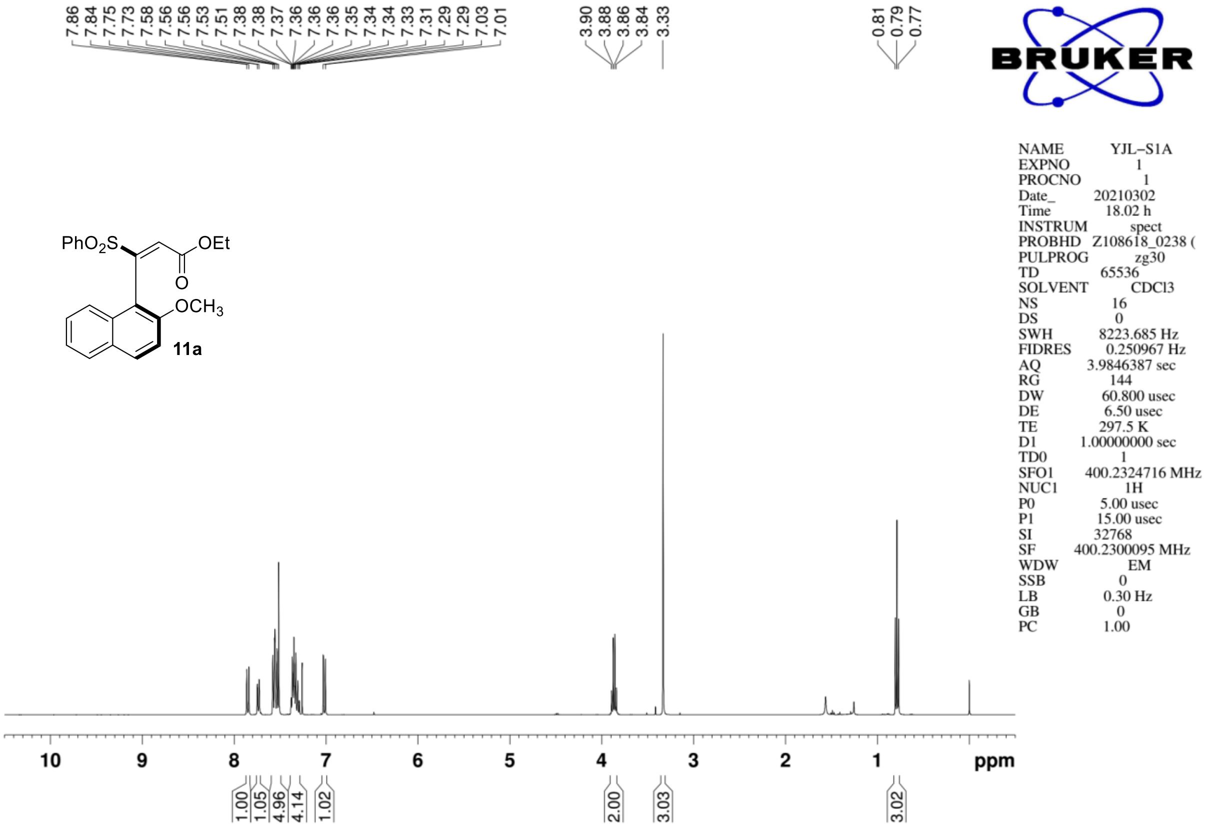

Supplementary Figure 166. ^{1}H NMR ($CDCl_3$, 400MHz) spectra of **11a**

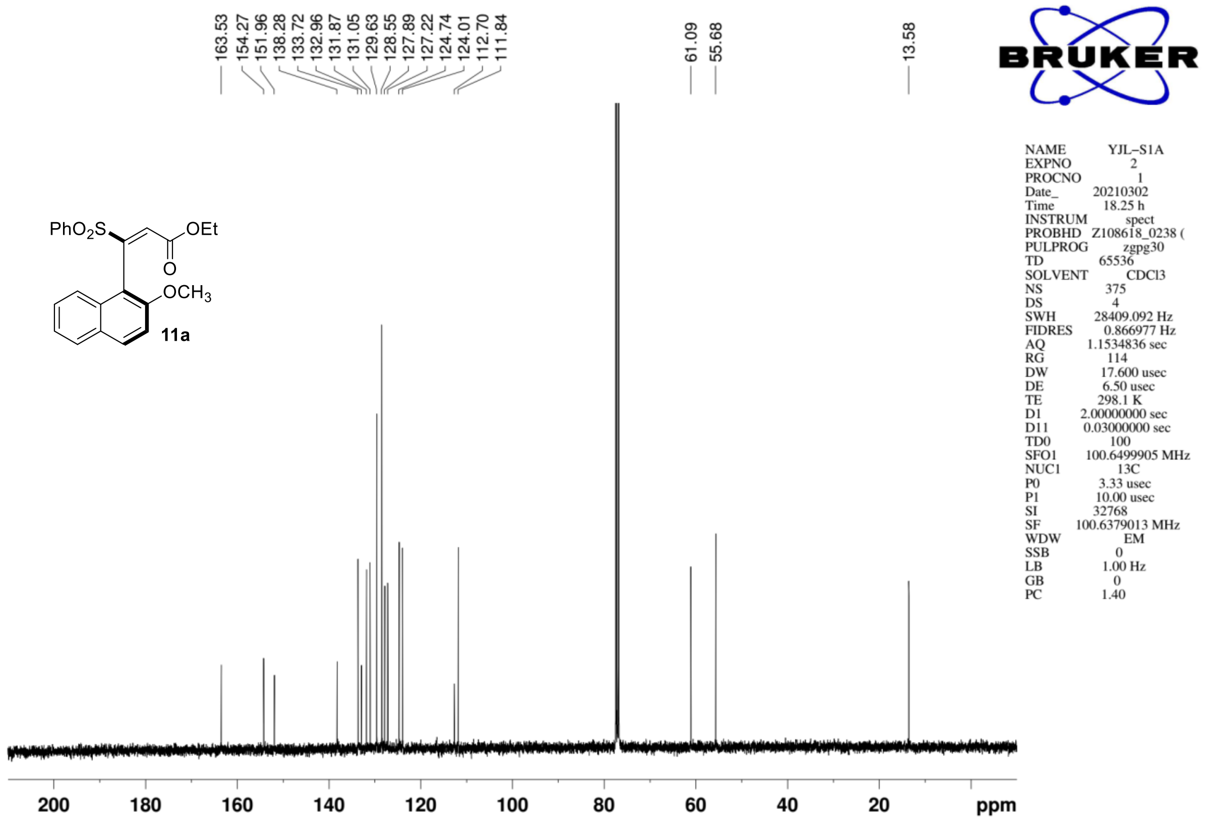

Supplementary Figure 167. ^{13}C NMR ($CDCl_3$, 101MHz) spectra of spectra of **11a**

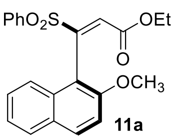

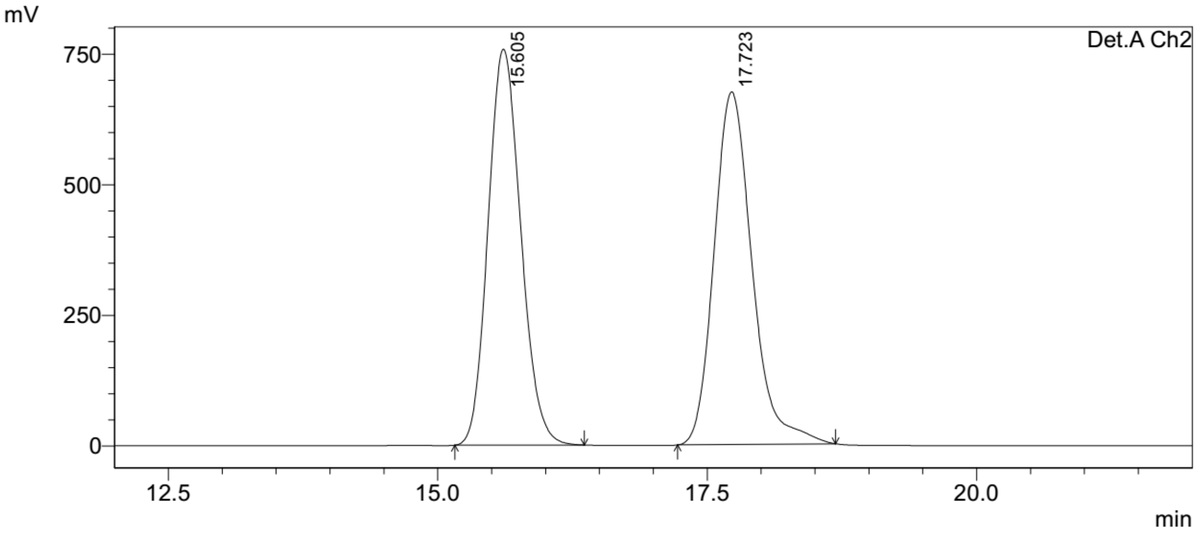

Detector A Ch2 220nm

Peak#	Ret. Time	Area	Height	Area %	Height %
1	15.605	16003505	758183	49.165	52.889
2	17.723	16547229	675355	50.835	47.111
Total		32550734	1433538	100.000	100.000

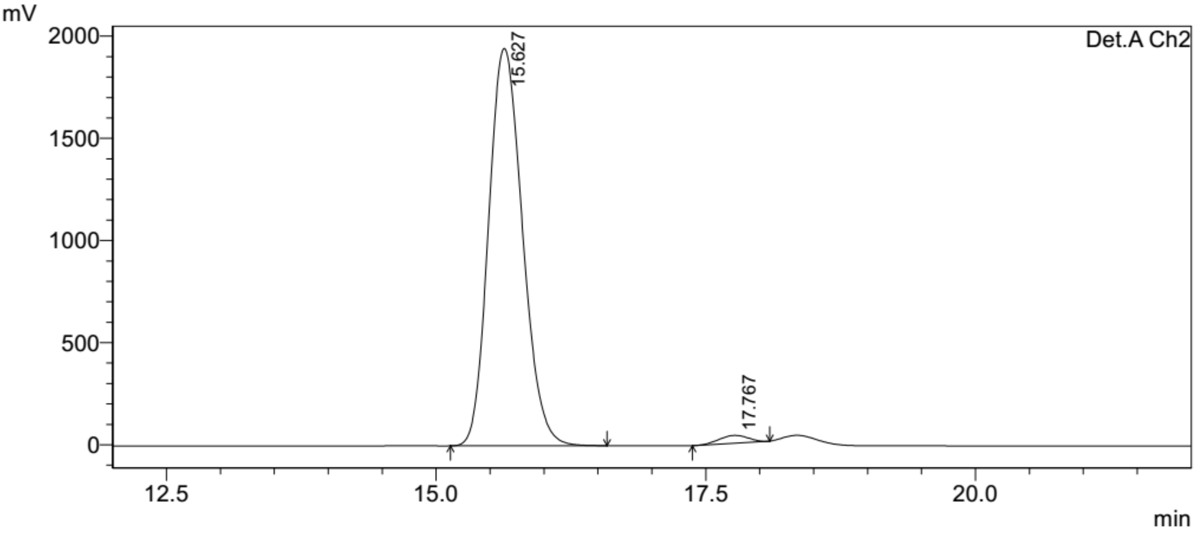

Detector A Ch2 220nm

Peak#	Ret. Time	Area	Height	Area %	Height %
1	15.627	42455706	1944941	98.354	98.084
2	17.767	710340	37997	1.646	1.916
Total		43166047	1982937	100.000	100.000

Supplementary Figure 168. HPLC spectra of **11a**

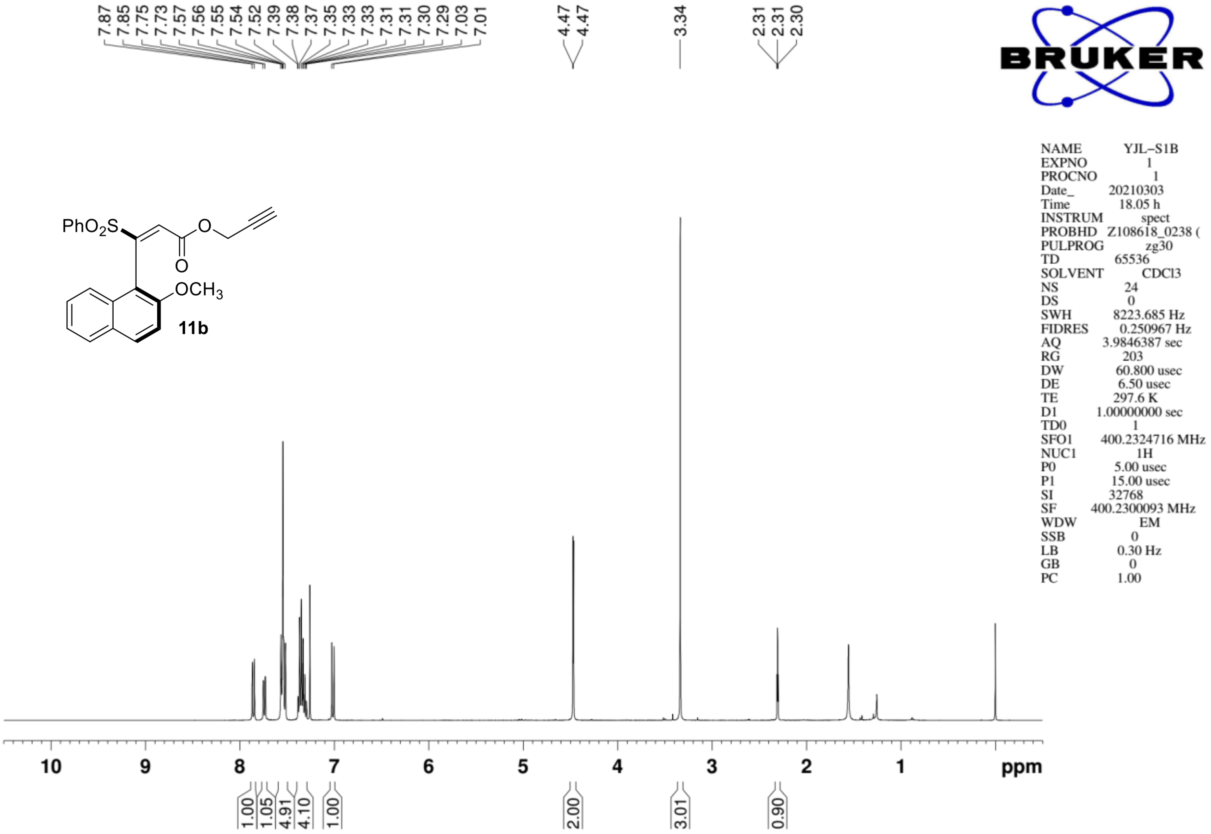

Supplementary Figure 169. ^{1}H NMR ($CDCl_3$, 400MHz) spectra of **11b**

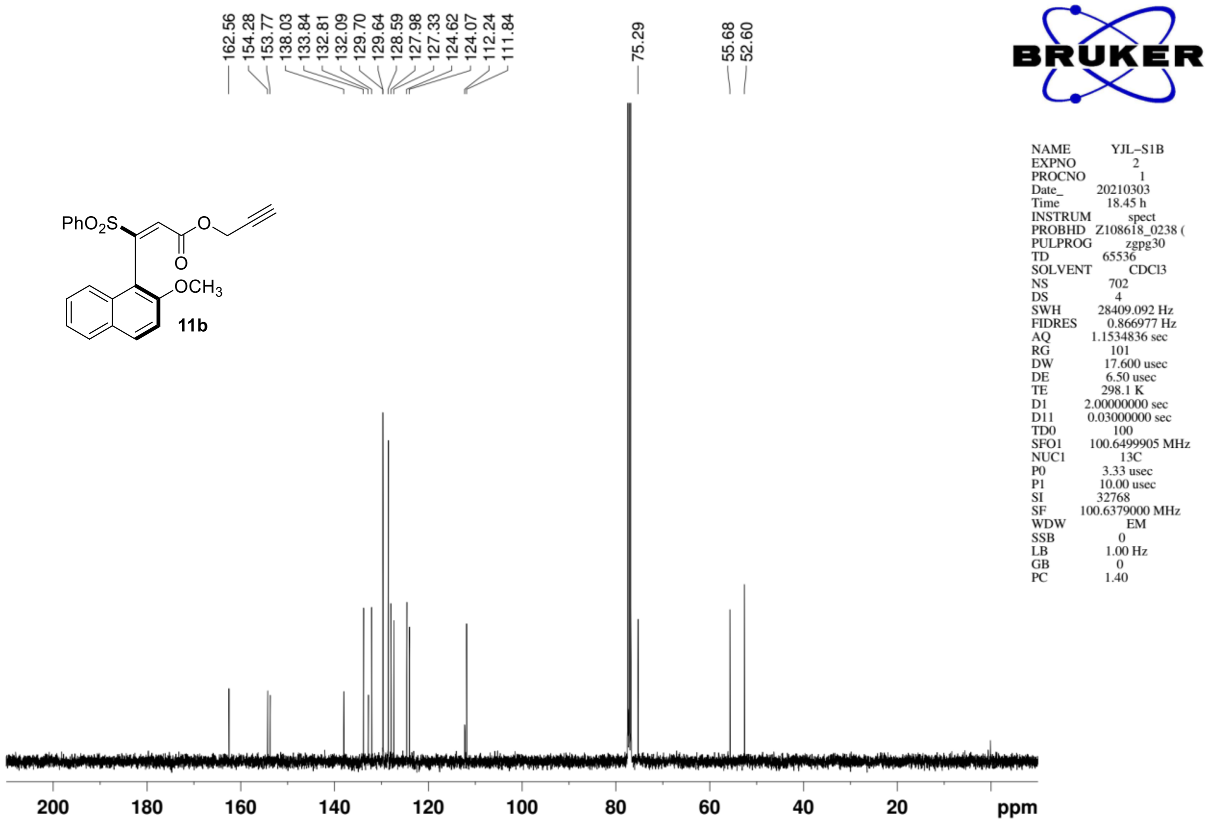

Supplementary Figure 170. ^{13}C NMR ($CDCl_3$, 101MHz) spectra of spectra of **11b**

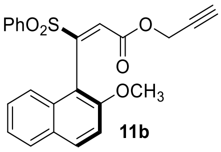

11b

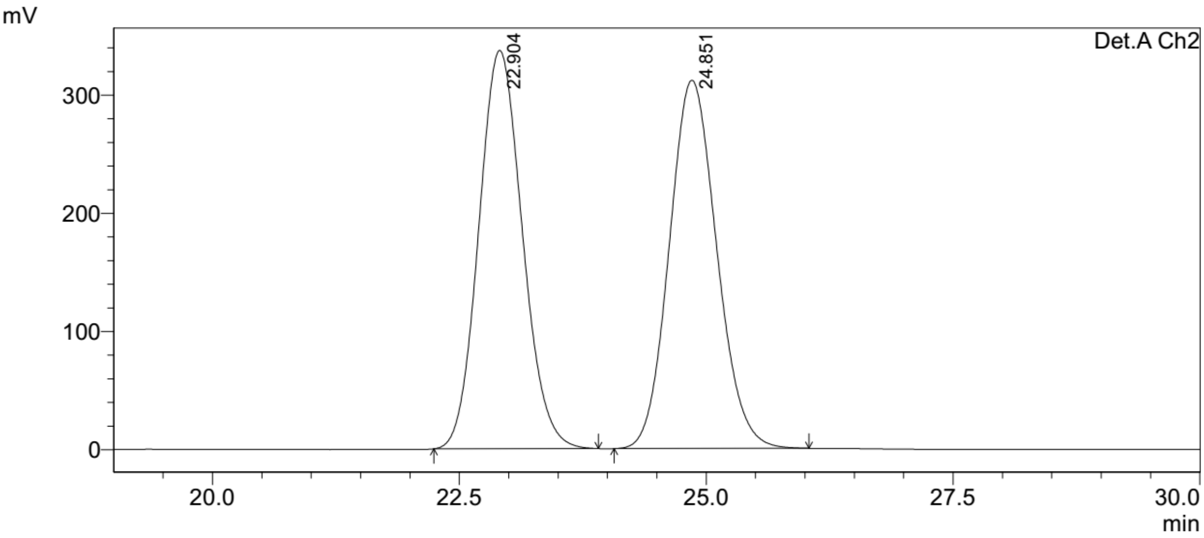

Detector A Ch2 220nm

Peak#	Ret. Time	Area	Height	Area %	Height %
1	22.904	10344436	337044	49.843	51.959
2	24.851	10409641	311634	50.157	48.041
Total		20754078	648678	100.000	100.000

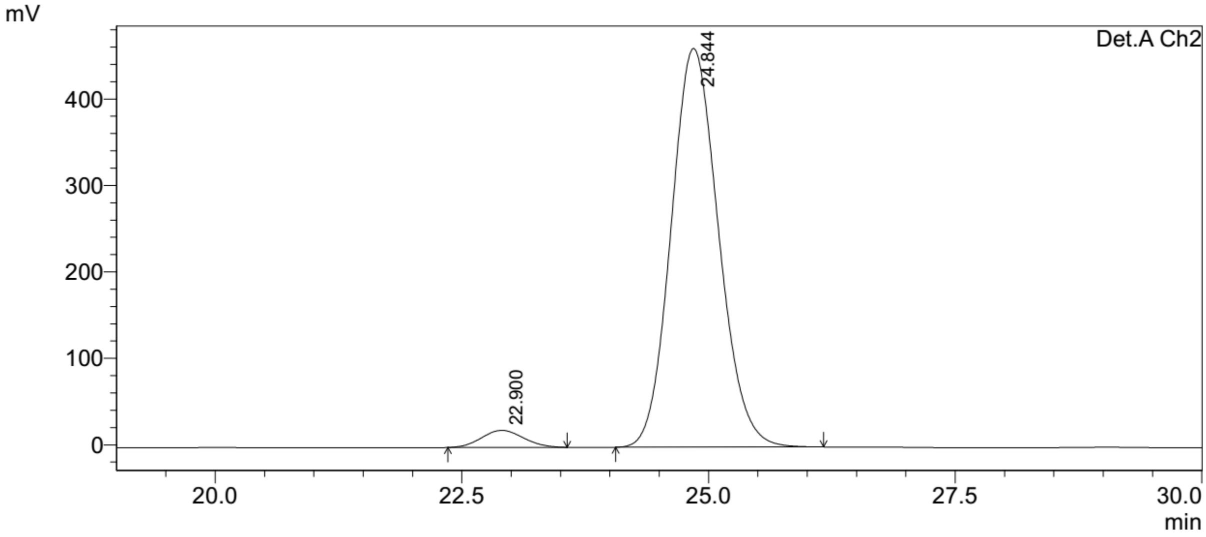

Detector A Ch2 220nm

Peak#	Ret. Time	Area	Height	Area %	Height %
1	22.900	584419	19737	3.653	4.104
2	24.844	15414294	461188	96.347	95.896
Total		15998713	480925	100.000	100.000

Supplementary Figure 171. HPLC spectra of **11b**

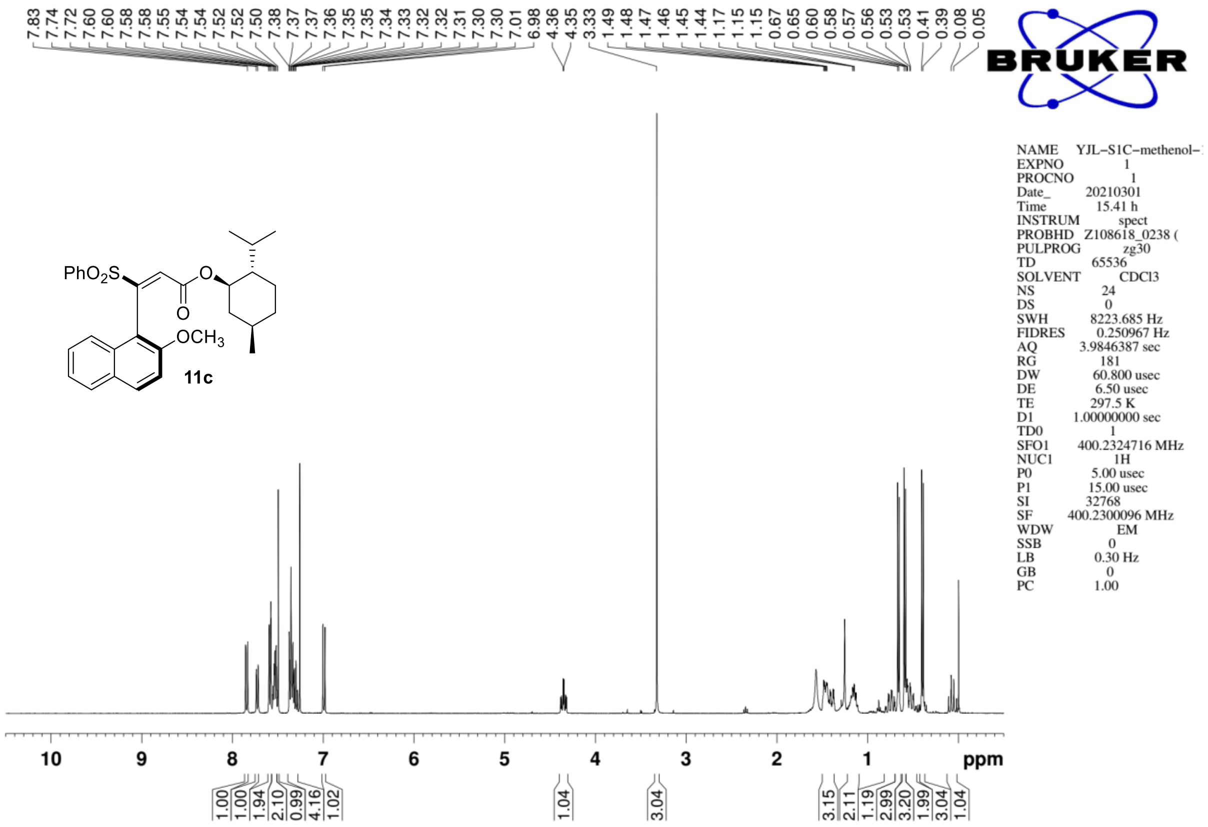

Supplementary Figure 172. ^{1}H NMR ($CDCl_3$, 400MHz) spectra of **11c**

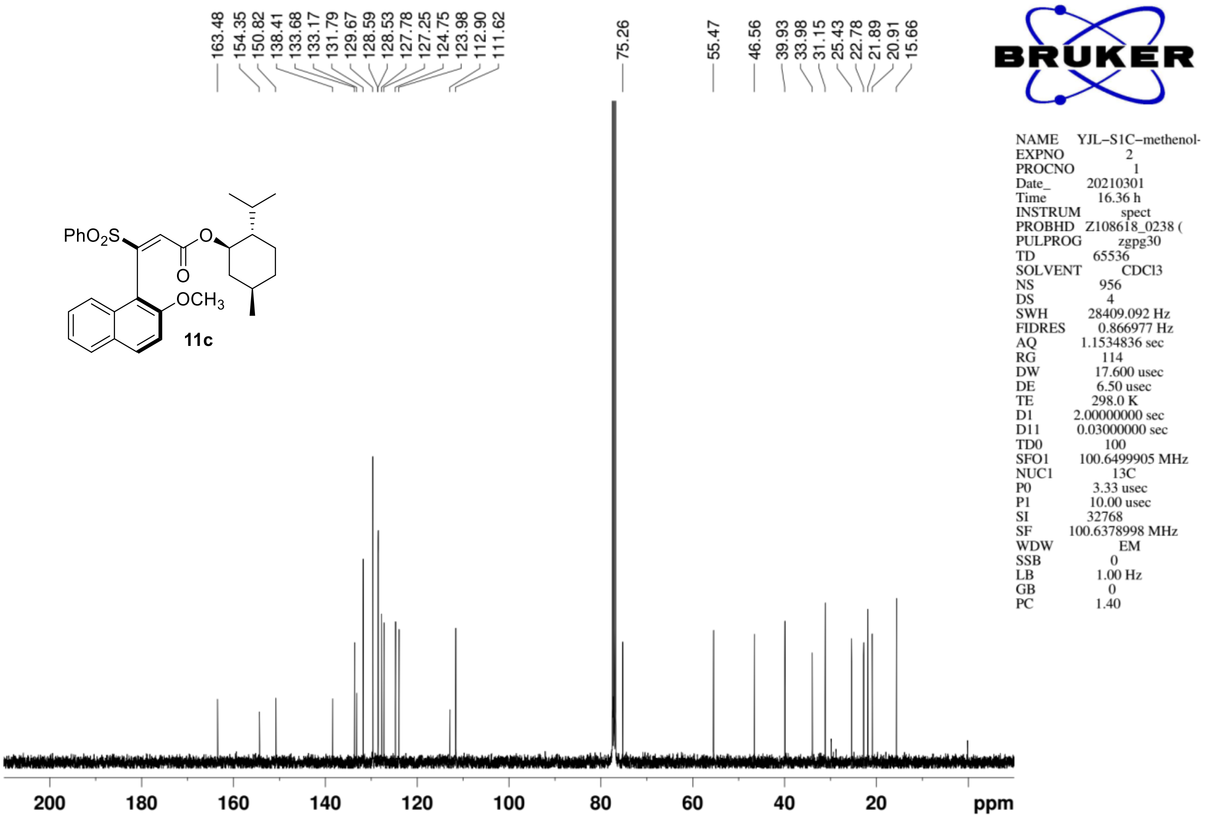

Supplementary Figure 173. ^{13}C NMR ($CDCl_3$, 101MHz) spectra of spectra of **11c**

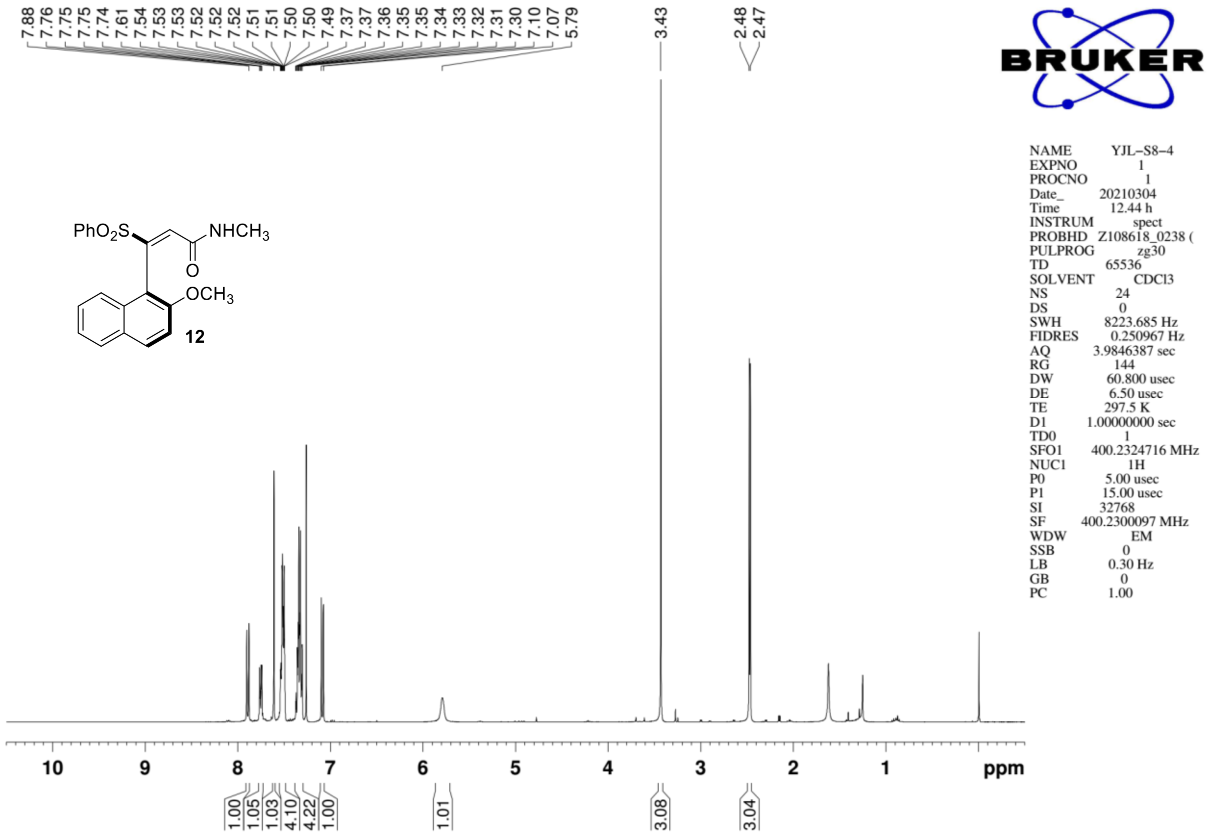

Supplementary Figure 174. ^{1}H NMR ($CDCl_3$, 400MHz) spectra of **12**

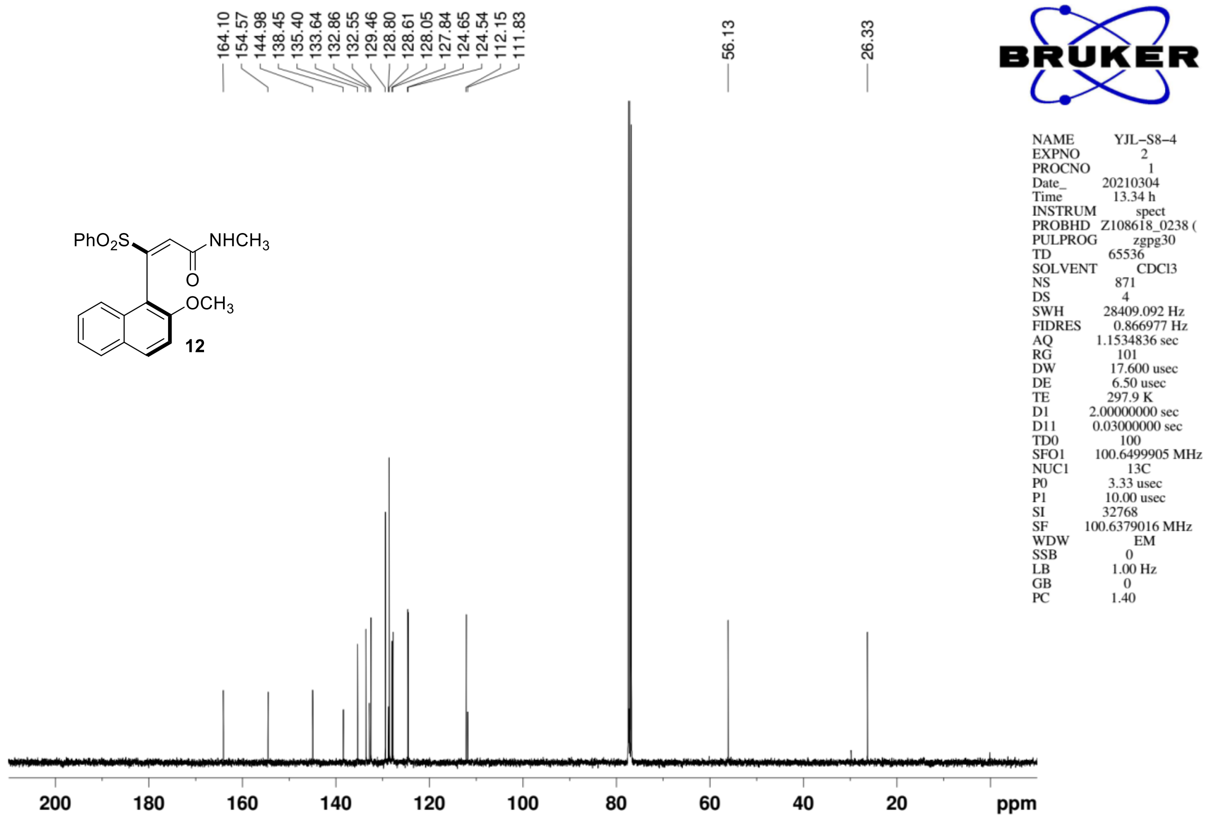

Supplementary Figure 175. ^{13}C NMR ($CDCl_3$, 101MHz) spectra of spectra of **12**

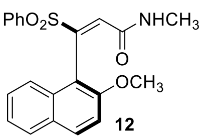

12

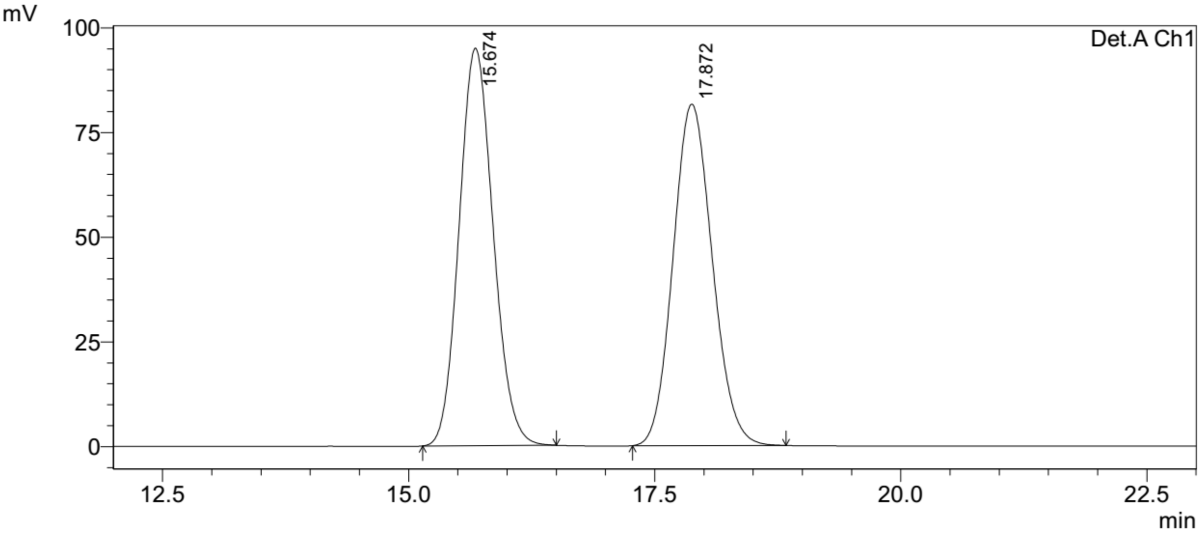

Detector A Ch1 254nm

Peak#	Ret. Time	Area	Height	Area %	Height %
1	15.674	2285335	94955	50.255	53.795
2	17.872	2262120	81560	49.745	46.205
Total		4547455	176515	100.000	100.000

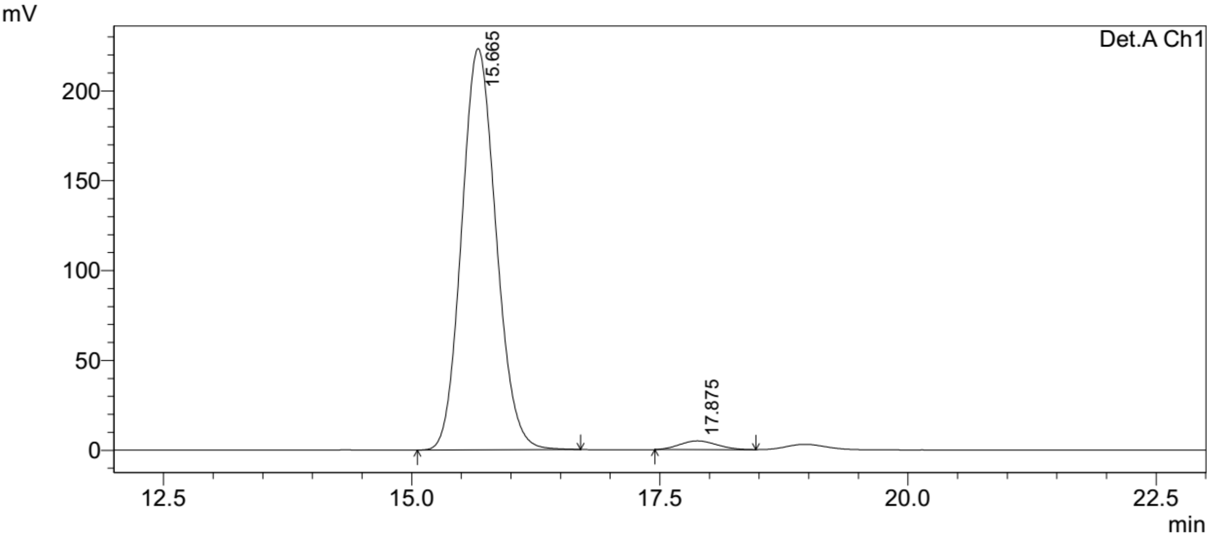

Detector A Ch1 254nm

Peak#	Ret. Time	Area	Height	Area %	Height %
1	15.665	5393075	223355	97.709	97.878
2	17.875	126432	4842	2.291	2.122
Total		5519507	228198	100.000	100.000

Supplementary Figure 176. HPLC spectra of **12**

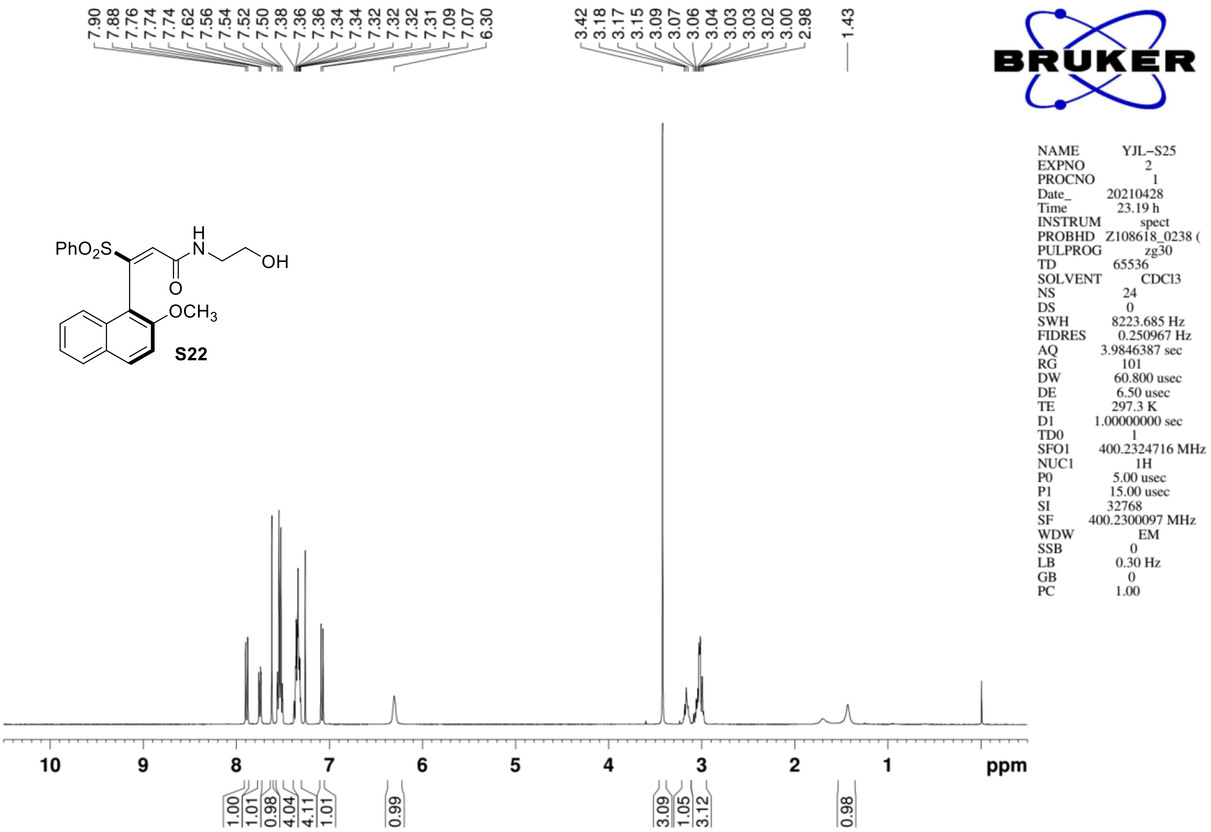

Supplementary Figure 177. ^{1}H NMR ($CDCl_3$, 400MHz) spectra of **S22**

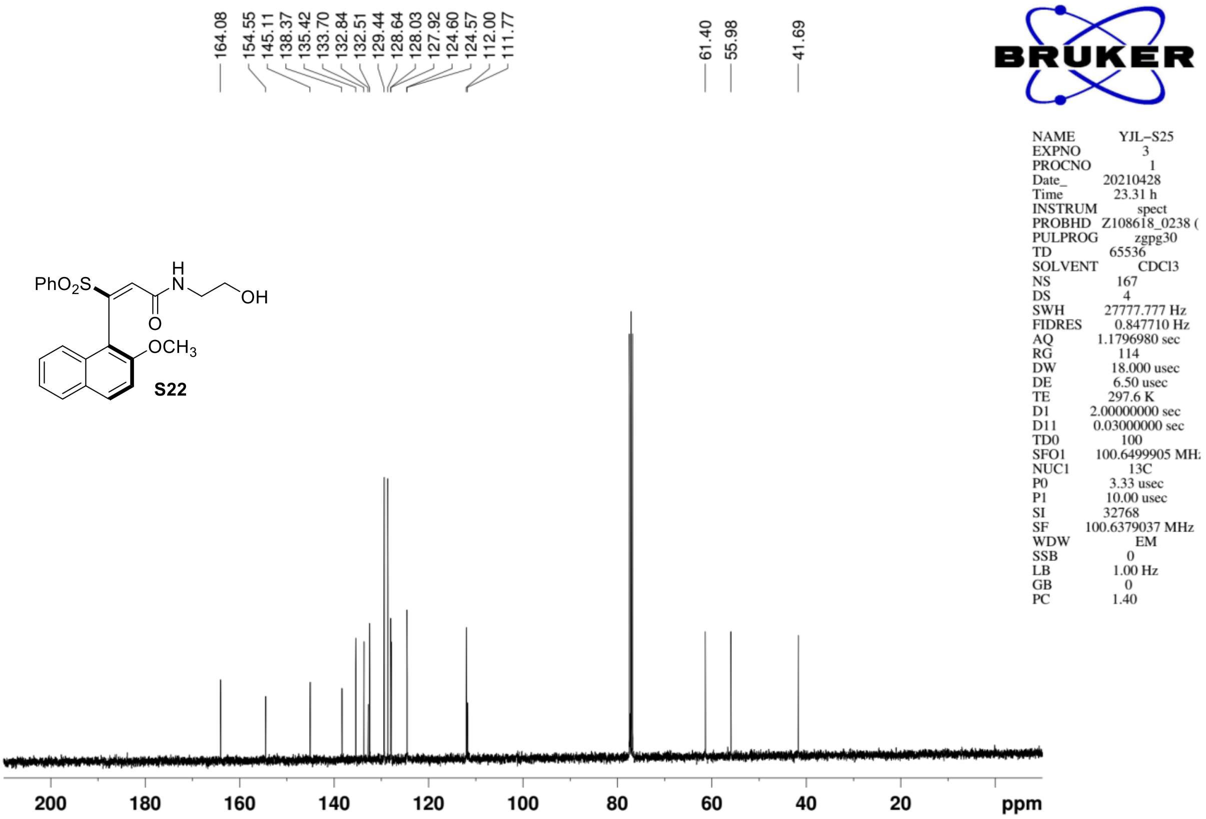

Supplementary Figure 178. ^{13}C NMR ($CDCl_3$, 101MHz) spectra of spectra of **S22**

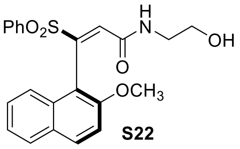

S22

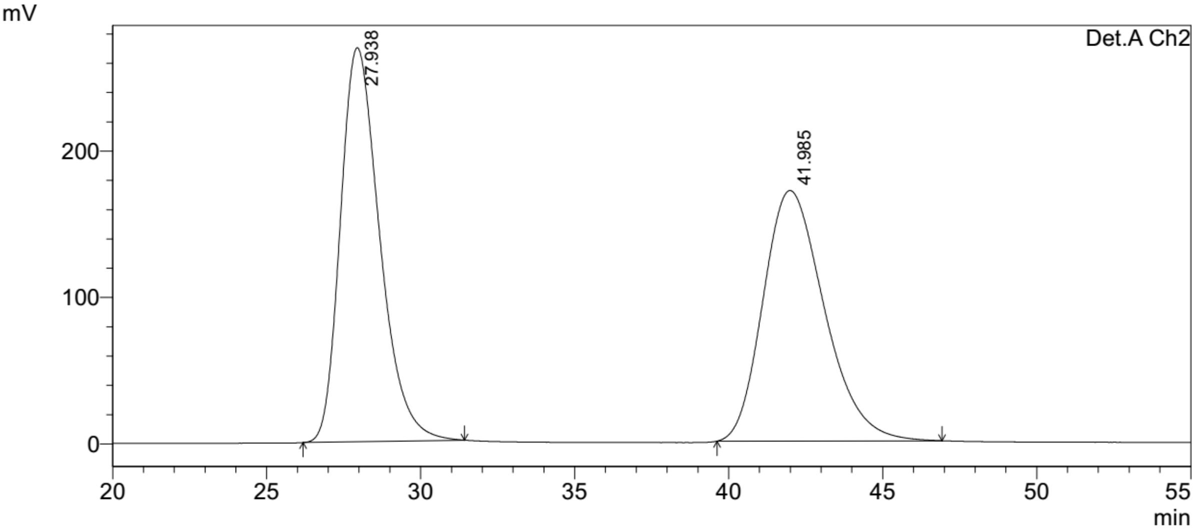

Detector A Ch2 220nm

Peak#	Ret. Time	Area	Height	Area %	Height %
1	27.938	23903089	269190	50.264	61.140
2	41.985	23652058	171092	49.736	38.860
Total		47555148	440282	100.000	100.000

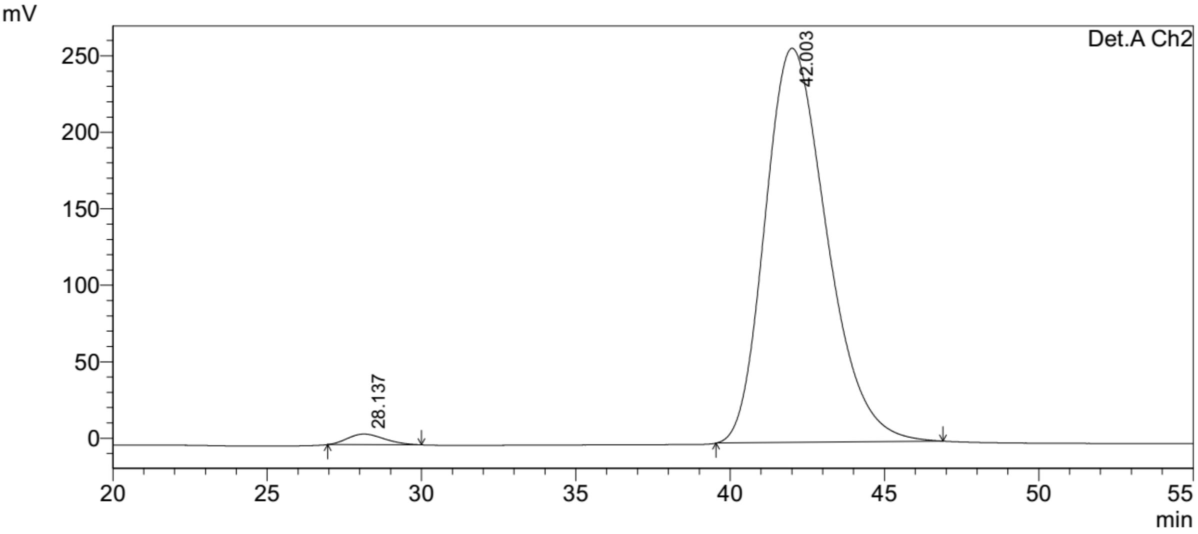

Detector A Ch2 220nm

Peak#	Ret. Time	Area	Height	Area %	Height %
1	28.137	576344	6929	1.581	2.618
2	42.003	35874053	257729	98.419	97.382
Total		36450397	264658	100.000	100.000

Supplementary Figure 179. HPLC spectra of **S22**

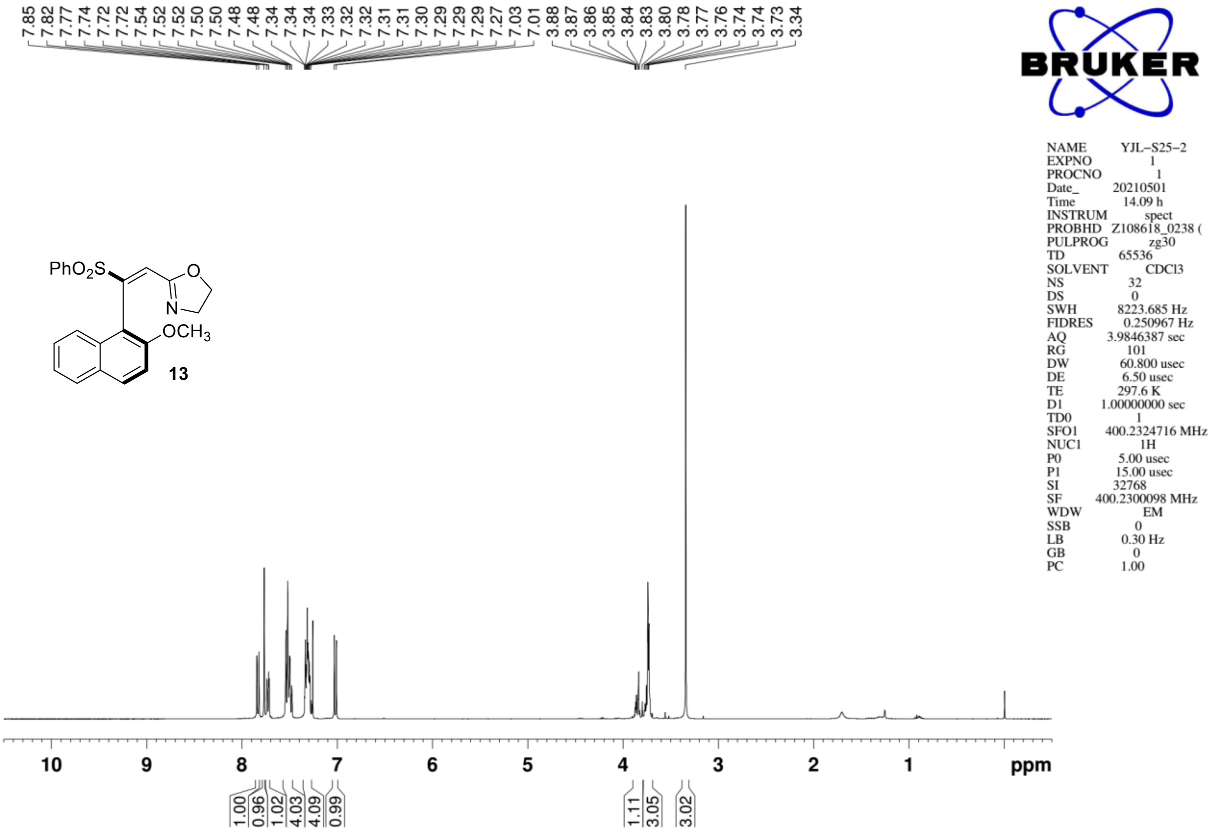

Supplementary Figure 180. ^{1}H NMR ($CDCl_3$, 400MHz) spectra of **13**

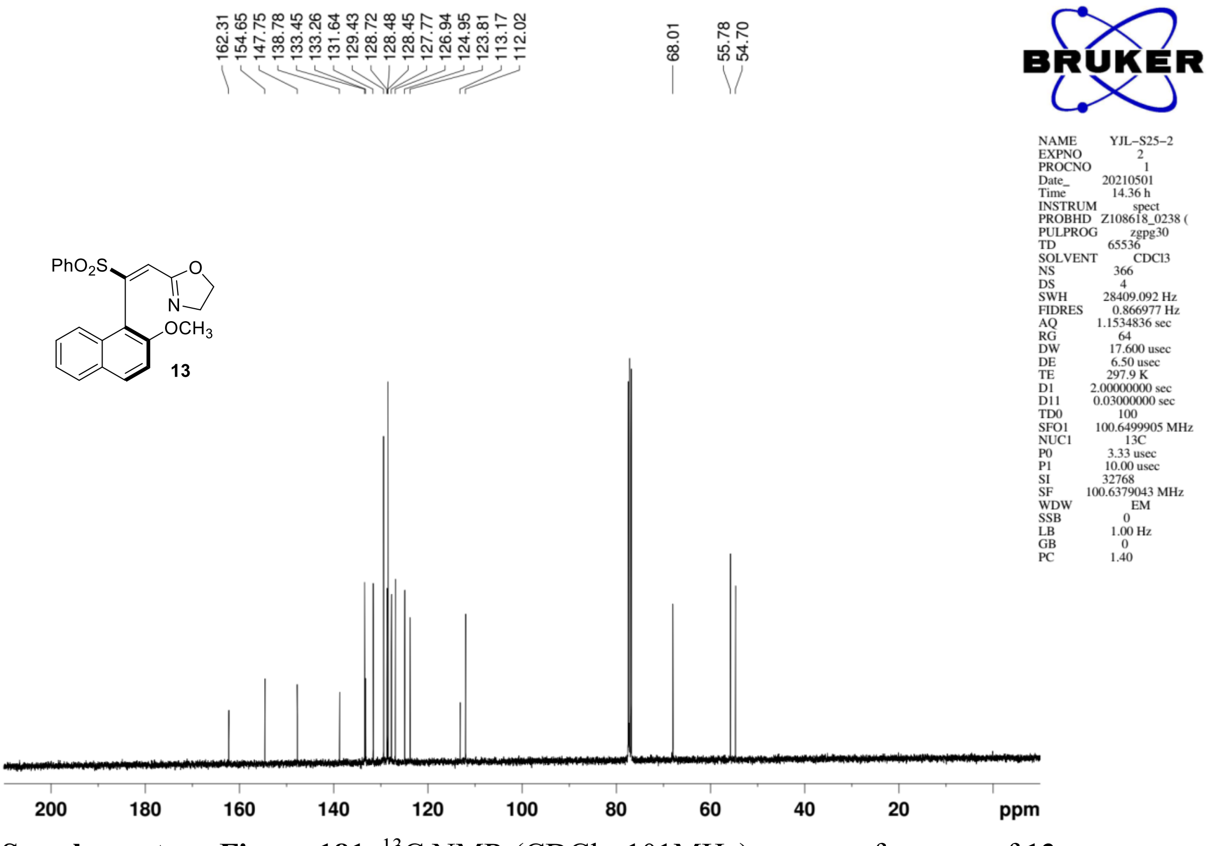

Supplementary Figure 181. ^{13}C NMR ($CDCl_3$, 101MHz) spectra of spectra of **13**

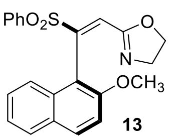

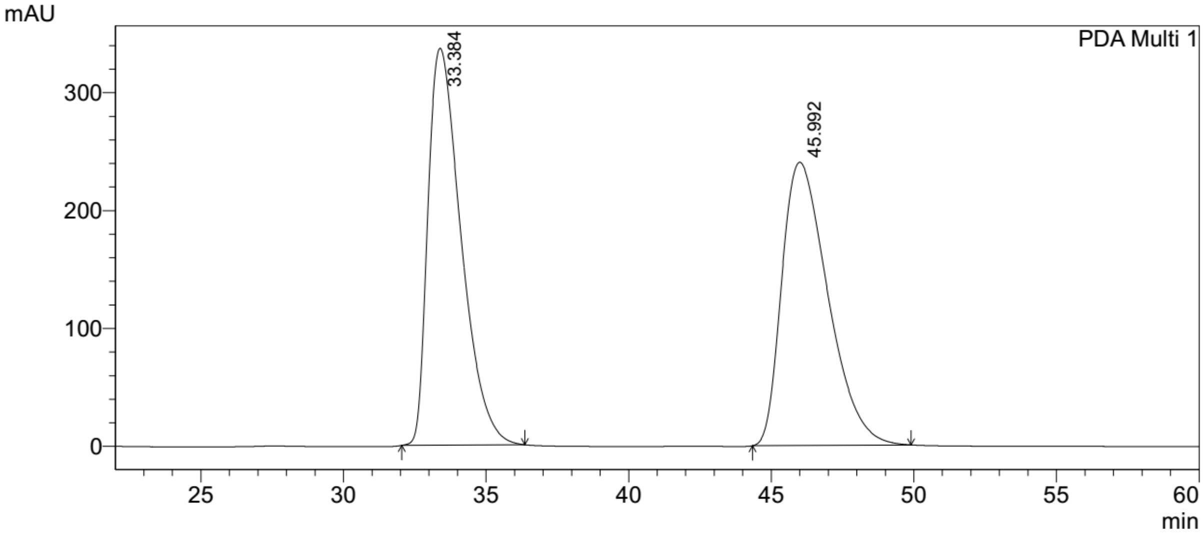

PDA Ch1 254nm 4nm

Peak#	Ret. Time	Area	Height	Area %	Height %
1	33.384	27246110	336779	50.249	58.361
2	45.992	26975878	240278	49.751	41.639
Total		54221989	577057	100.000	100.000

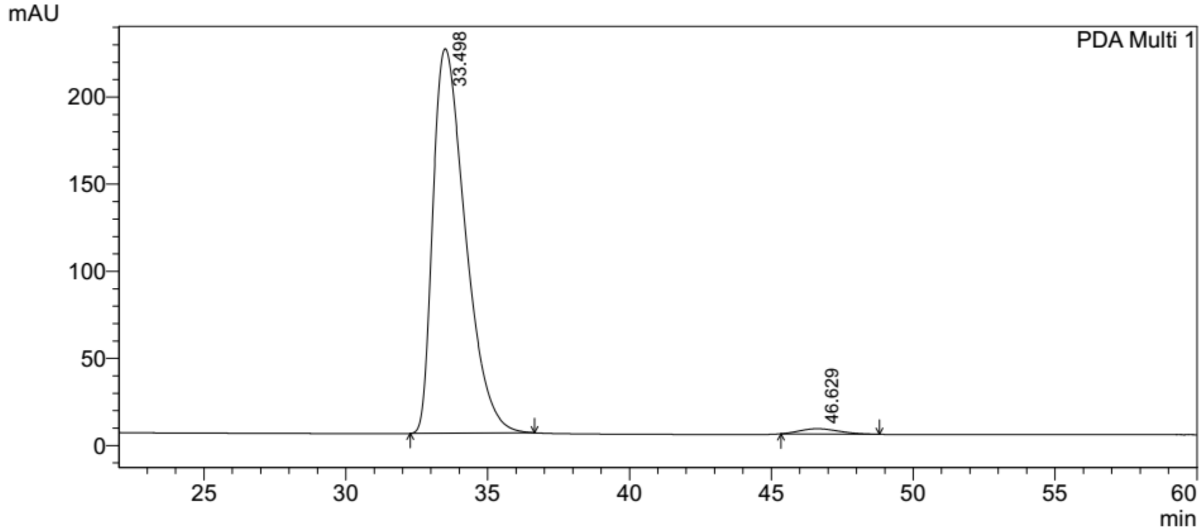

PDA Ch1 254nm 4nm

Peak#	Ret. Time	Area	Height	Area %	Height %
1	33.498	17562820	220798	98.389	98.646
2	46.629	287606	3032	1.611	1.354
Total		17850426	223830	100.000	100.000

Supplementary Figure 182. HPLC spectra of **13**

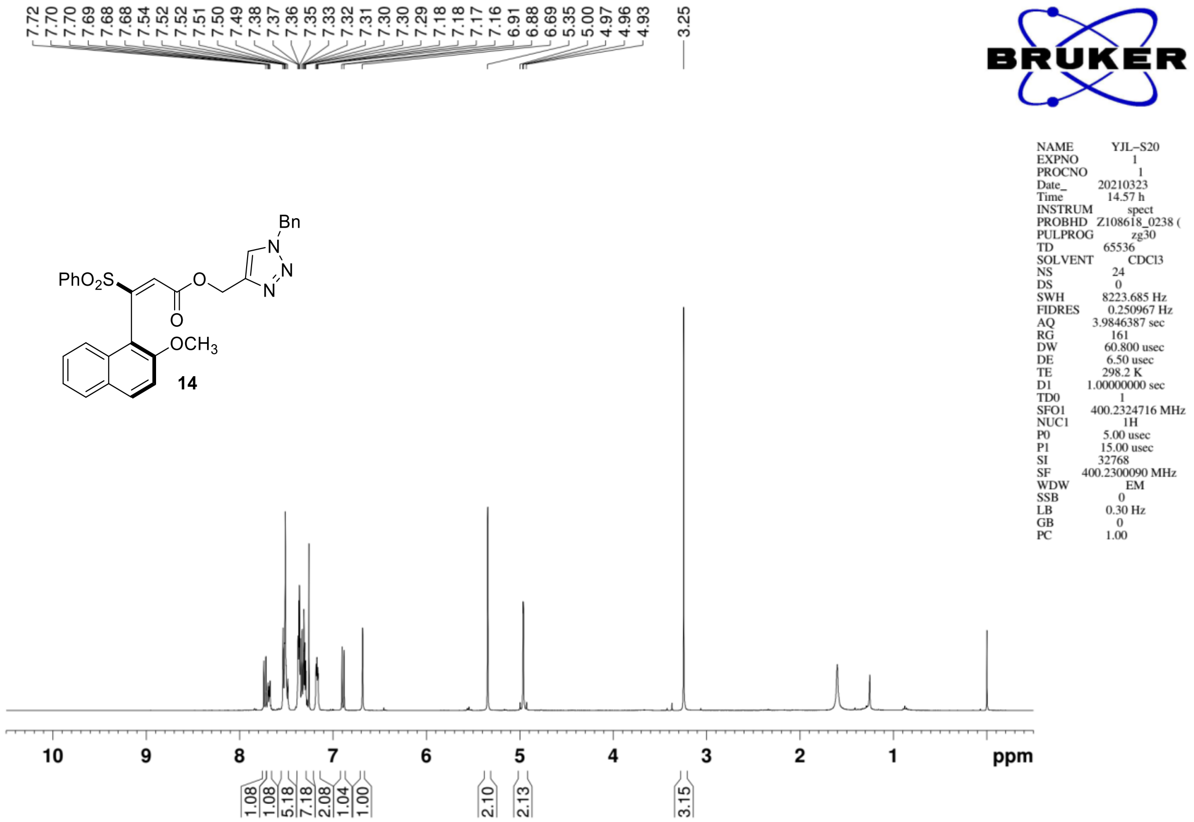

Supplementary Figure 183. ^{1}H NMR ($CDCl_3$, 400MHz) spectra of **14**

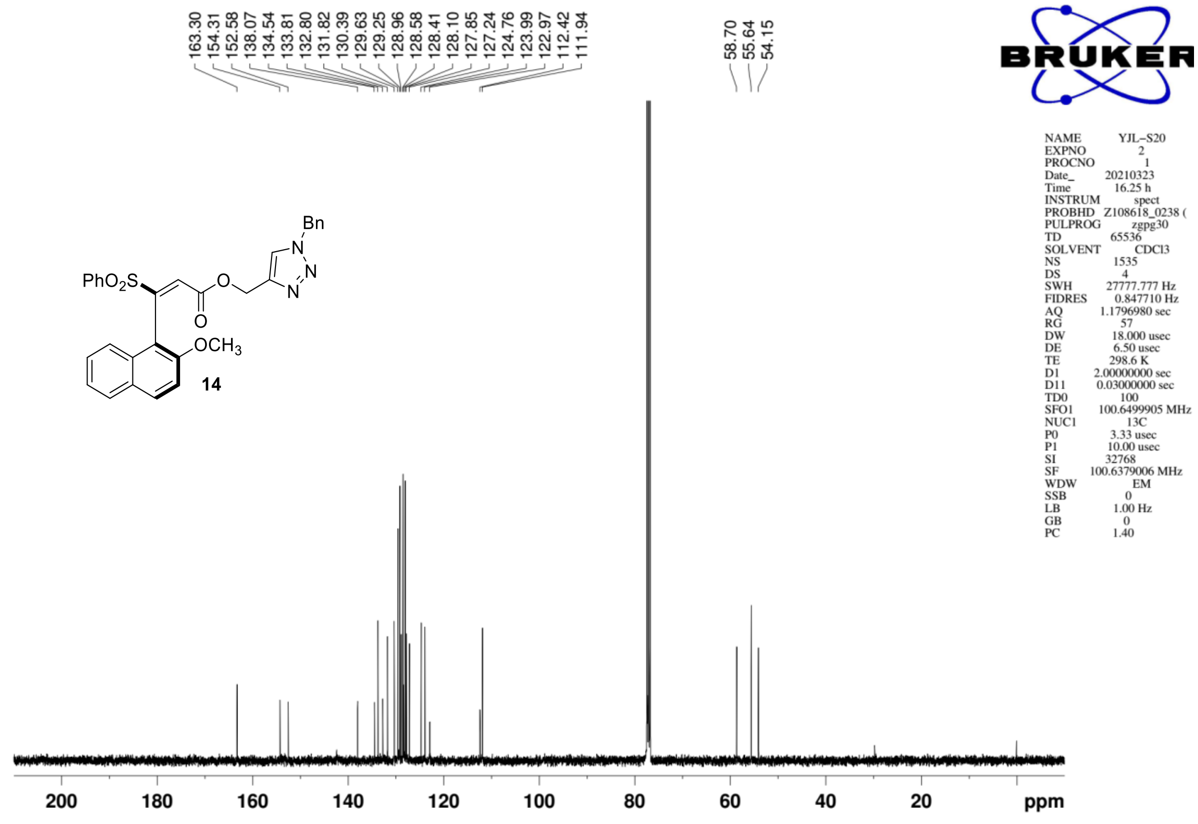

Supplementary Figure 184. ^{13}C NMR ($CDCl_3$, 101MHz) spectra of spectra of **14**

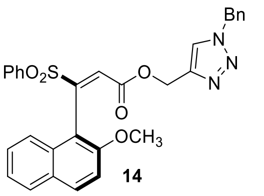

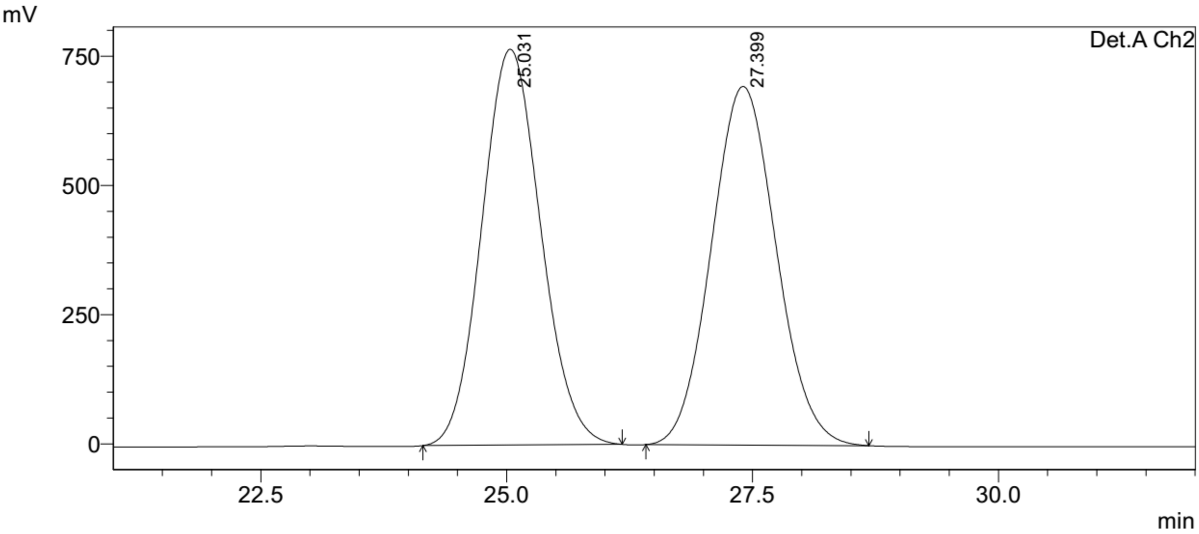

Detector A Ch2 220nm

Peak#	Ret. Time	Area	Height	Area %	Height %
1	25.031	31935132	765886	50.086	52.465
2	27.399	31825231	693912	49.914	47.535
Total		63760363	1459798	100.000	100.000

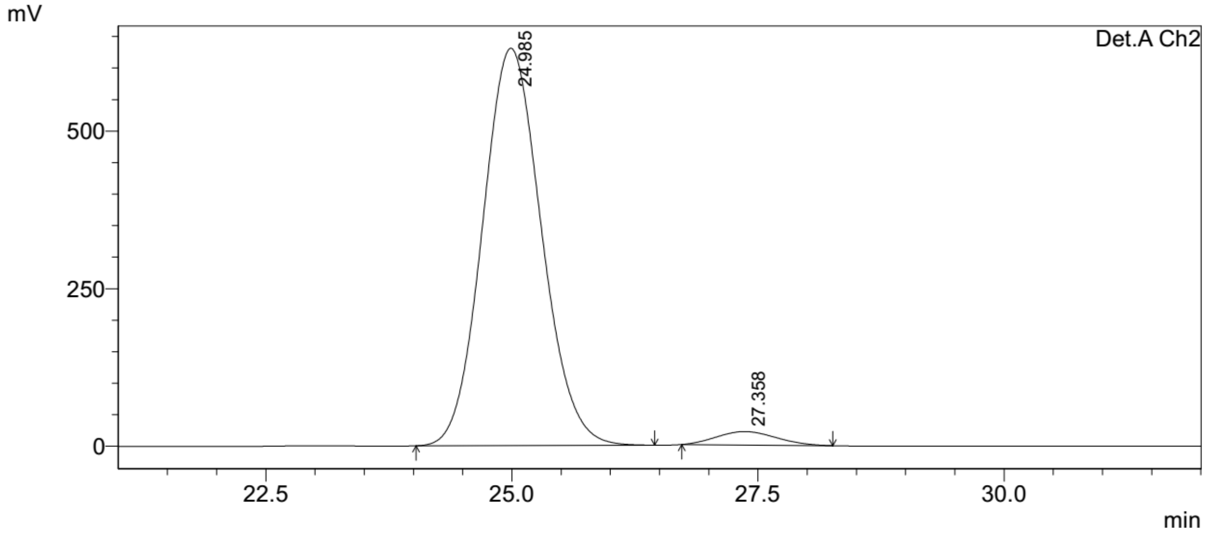

Detector A Ch2 220nm

Peak#	Ret. Time	Area	Height	Area %	Height %
1	24.985	26221146	630361	96.695	96.733
2	27.358	896192	21288	3.305	3.267
Total		27117338	651649	100.000	100.000

Supplementary Figure 185. HPLC spectra of **14**

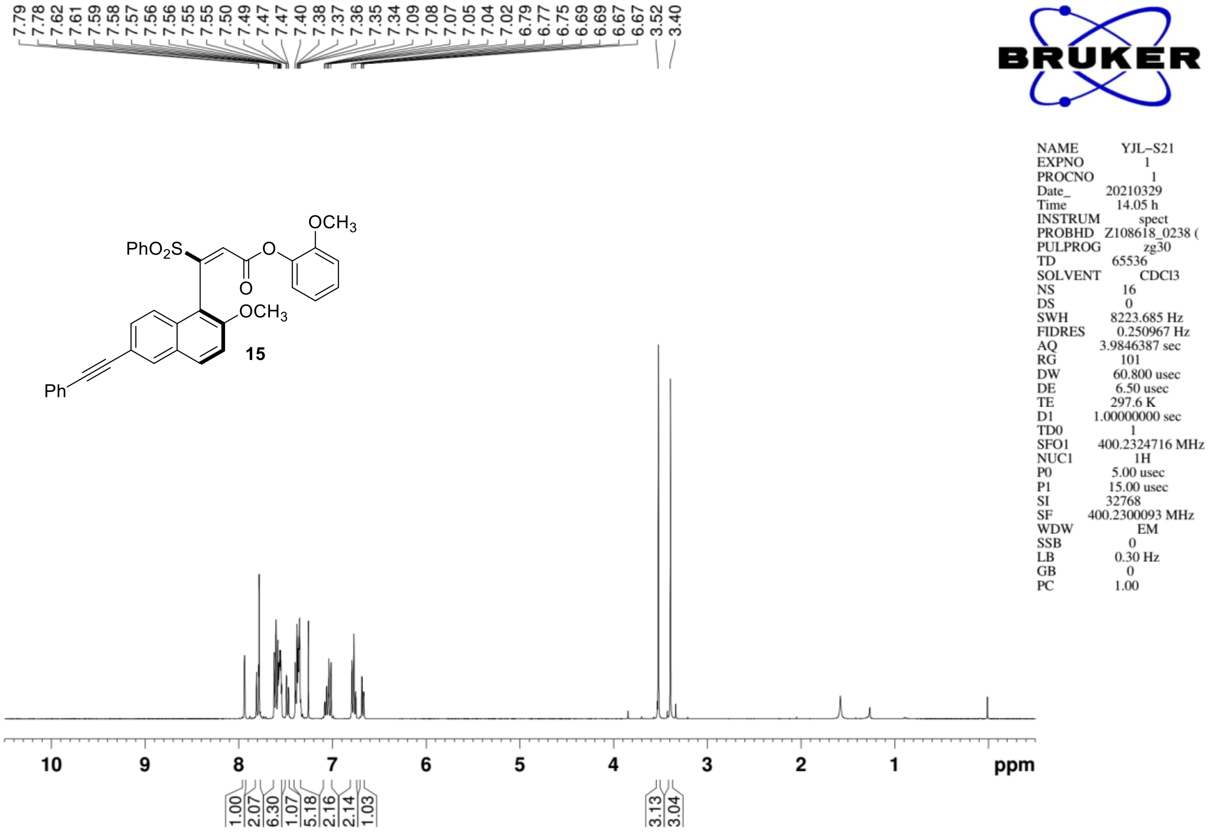

Supplementary Figure 186. ^{1}H NMR ($CDCl_3$, 400MHz) spectra of **15**

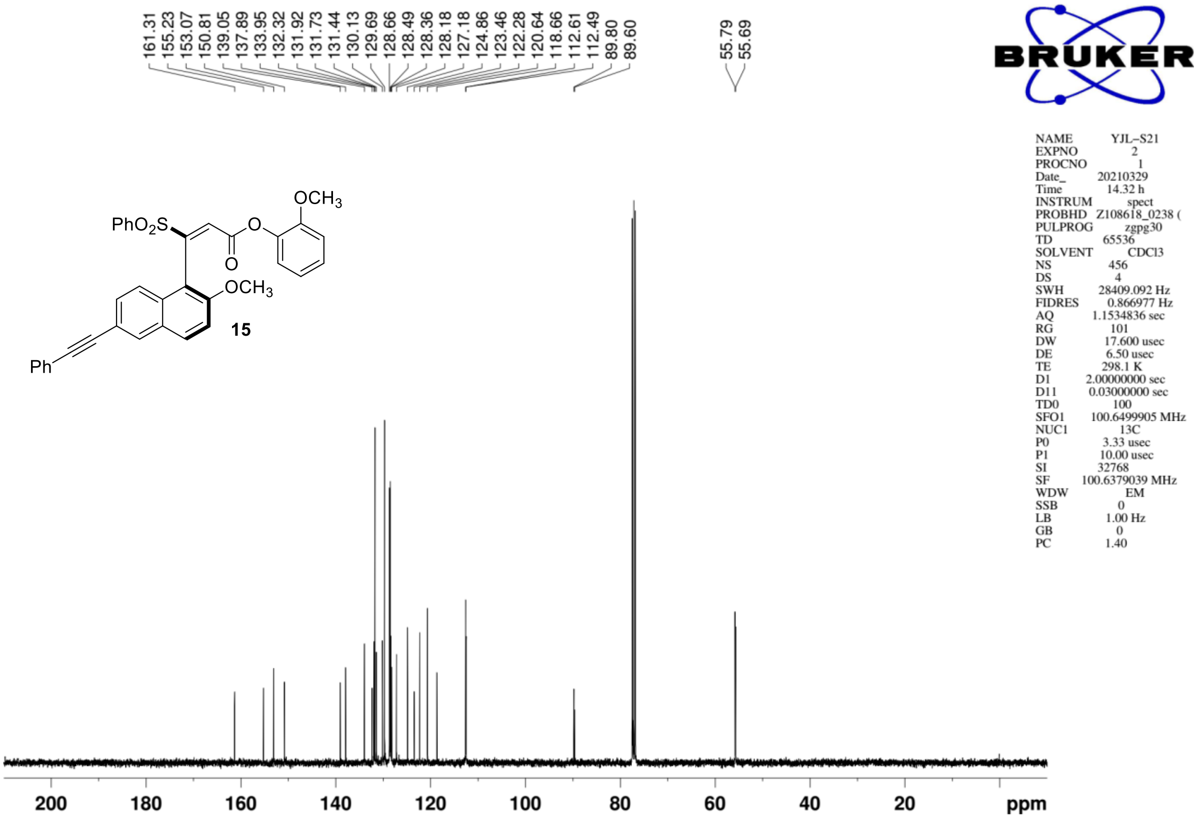

Supplementary Figure 187. ^{13}C NMR ($CDCl_3$, 101MHz) spectra of spectra of **15**

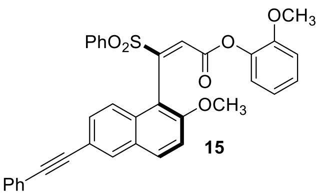

15

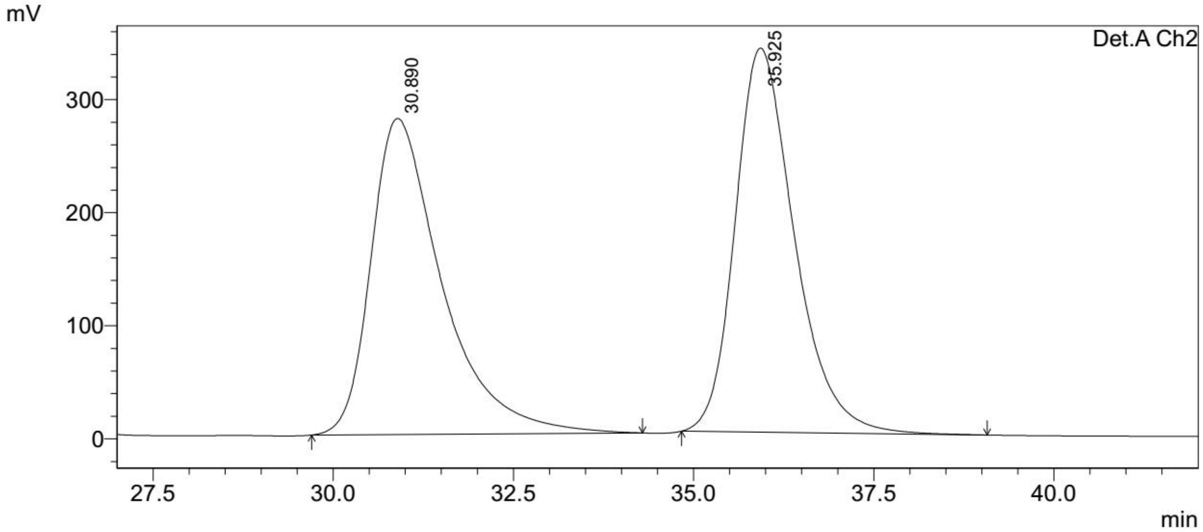

Detector A Ch2 220nm

Peak#	Ret. Time	Area	Height	Area %	Height %
1	30.890	19204038	279507	49.705	45.147
2	35.925	19431660	339603	50.295	54.853
Total		38635698	619110	100.000	100.000

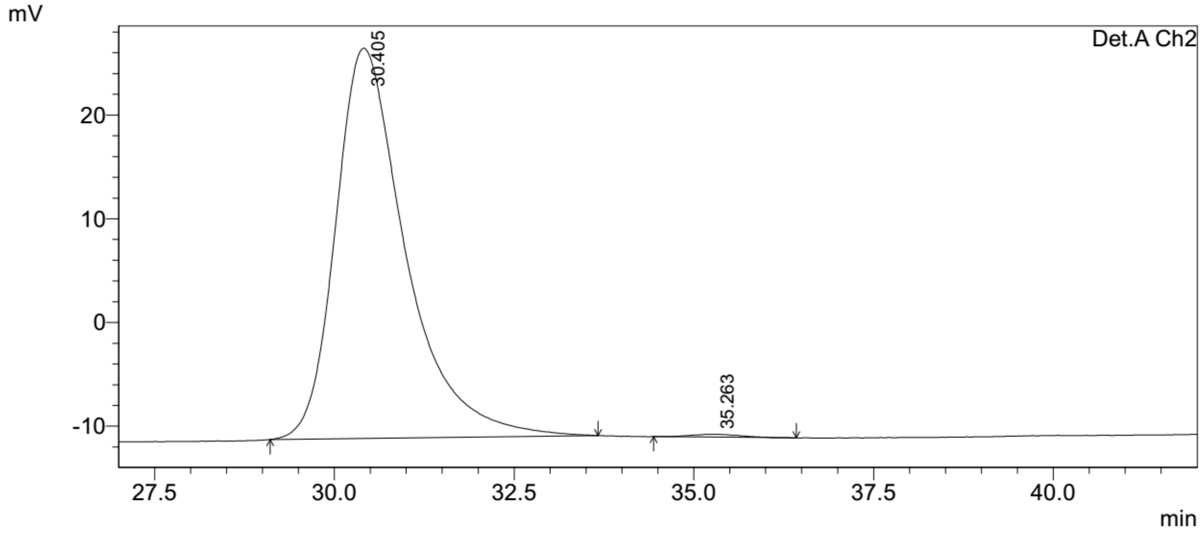

Detector A Ch2 220nm

Peak#	Ret. Time	Area	Height	Area %	Height %
1	30.405	2549149	37617	99.494	99.265
2	35.263	12958	279	0.506	0.735
Total		2562108	37895	100.000	100.000

Supplementary Figure 188. HPLC spectra of **15**

2. Supplementary Methods

2.1 General Information

All reactions were conducted in flame-dried or oven-dried glassware under an atmosphere of dry nitrogen or argon. Oxygen and/or moisture sensitive solids and liquids were transferred appropriately. Concentration of solutions in vacuo was accomplished using a rotary evaporator fitted with a water aspirator. Residual solvents were removed under high vacuum (0.1-0.2 mm Hg). All commercially available materials purchased form TCI or Sigma Aldrich was used as received. All reaction solvents were purified before use: Tetrahydrofuran were distilled from Na/benzophenone. Toluene was distilled over molten sodium metal. Dichloromethane, dimethylformamide, diethylamine, triethylamine and diisoproylethylamine were distilled from CaH_2. Methanol was distilled from Mg/I_2. Flash column chromatography was performed using silica gel (230 – 400 mesh). TLC was carried out using pre-coated sheets (Yantai silica gel 60-F250, 0.2 mm). Compounds were visualized with UV light, iodine, *p*-anisaldehyde stain, ceric ammonium molybdate stain, or phosphomolybdic acid in EtOH.

Proton nuclear magnetic resonance (^{1}H NMR) spectra were recorded with Bruker BBFO 400 MHz, Bruker Avance 400 MHz or Bruker Avance 500 MHz spectrometers. Chemical shifts are reported in delta (δ) units, parts per million (ppm) downfield from tetramethylsilane or ppm relative to the center of the singlet at 7.26 ppm for deuteriochloroform. The following abbreviations are used to describe spin multiplicity: s = singlet, d = doublet, t = triplet, q = quartet, qn = quintet, m = multiplet, br = broad, dd = doublet of doublets, dt = doublet of triplets, dq = doublet of quartets, ddd = doublet of doublet of doublets; other combinations are derived from those listed above. Integration and coupling constants were reported in Hertz (Hz). Coupling constants (*J*) are reported in Hertz (Hz) for corresponding solutions. Carbon-13 nuclear magnetic resonance (^{13}C NMR) spectra were recorded with a 101 MHz or a 126 MHz spectrometer and were routinely run with broadband decoupling. Chemical shifts are reported in delta (δ) units, ppm relative to the center of the triplet at 77.16 ppm for deuteriochloroform. High-resolution mass spectra (HRMS) were obtained on Finnigan MAT 95 XP mass spectrometer (Thermo Electron Corporation) and are reported as m/z (relative intensity). Accurate masses are reported for the molecular ion ($M+H^+$, $M+Na^+$), or a suitable fragment ion. Optical rotations were measured using a 1 mL cell with a 1 dm path length on a Jasco P-1030 polarimeter and data were reported as follow: optical rotation (c in g per 100 mL solvent). The determination of *e.r.* was performed via chiral HPLC analysis using Shimadzu LC-20AD HPLC workstation.

2.2 Preparation of the NHC Pre-catalysts

NHC pre-catalysts **A-D** are known compounds in literature reports.[1-4] NHC pre-catalyst **E** are prepared according to the following procedures:

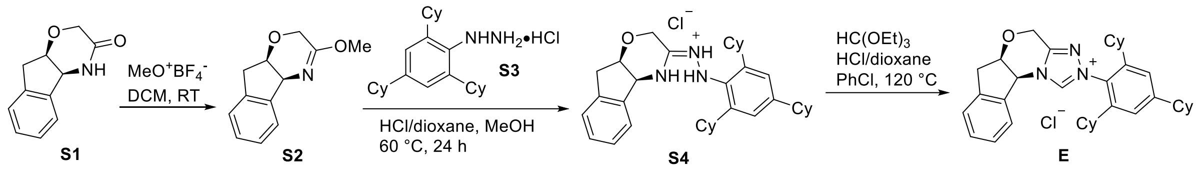

To a solution of compound **S1** (5.0 g, 26.4 mmol) in DCM (100 mL) was added trimethyloxonium tetrafluoroborate (4.7 g, 31.7 mmol) portionwise at room temperature. The mixture was stirred for 24 h at room temperature. The reaction was quenched with a saturated aquous solution of $NaHCO_3$ (100 mL) at 0 °C, and the mixture was extracted with DCM (100 mL x 3). The combined organic phases were washed sequentially with a saturated aquous solution of $NaHCO_3$ (50 mL), water (50 mL) and brine (50 mL), dried over anhydrous Na_2SO_4 and concentrated in *vacuo* to provide the crude **S2.** To a solution of the above **S2** and hydrazine chloride **S3**[5] (10.3 g, 26.4 mmol) in anhydrous MeOH (100 mL) was added HCl (4.0 M in dioxane, 0.7 mL, 2.8 mmol) at room temperature. The resulting mixture was heated to 60 °C for 36 h. After cooled to room temperature, the reaction mixture was directly concentrated in *vacuo* to provide the crude **S4.** To a sealed tube equipped with a magnetic stir bar was added the crude hydrazine **S4**, chlorobenzene (27 mL, 264 mmol), triethyl orthoformate (39 mL, 264 mmol) and HCl (4.0 M in dioxane, 7 mL, 28 mmol). The tube was sealed and heated to 120 °C for 2 h. After cooled to room temperature, the reaction mixture was directly concentrated in *vacuo,* and the residue was purified by flash chromatography on silica gel (EtOAc/hexanes =1:5) to provide NHC pre-catalyst **E** (7.3 g, 48% yield for 3 steps) as a light yellow solid.

^{1}H NMR (400 MHz, $CDCl_3$) δ 12.04 (s, 1H), 7.55 (d, *J* = 7.4 Hz, 1H), 7.39 – 7.26 (m, 3H), 7.14 (s, 1H), 7.07 (s, 1H), 6.88 (d, *J* = 4.0 Hz, 1H), 5.14 – 4.97 (m, 3H), 3.30 (dd, *J* = 17.0, 4.6 Hz, 1H), 3.19 (d, *J* = 17.0 Hz, 1H), 2.62 – 2.48 (m, 1H), 2.31 – 2.08 (m, 2H), 2.03 – 0.83 (m, 30H) ppm.

^{13}C NMR (101 MHz, $CDCl_3$) δ 152.3, 149.1, 145.6, 145.5, 144.0, 139.9, 136.1, 129.7, 128.7, 128.1, 125.6, 124.2, 123.4, 77.8, 62.4, 60.3, 45.1, 39.9, 39.1, 37.6, 35.3, 35.3, 34.4, 34.4, 33.7, 26.9, 26.8, 26.6, 26.5, 26.2, 26.1, 26.0, 25.8 ppm.

HRMS (ESI, m/z): calculated for $C_{36}H_{46}N_3O^+$: 536.3641 $(M-Cl^-)^+$, found: 536.3639.

$[\alpha]^{21}_D$ = +57.3 (c = 0.5 in MeOH).

M.P. 191 °C - 193 °C

2.3 Preparation of the Sulfinic Acids

Benzenesulfinic acid and *p*-toluenesulfinic acid were obtained by acidification of the commercially available sodium benzenesulfinate and sodium *p*-toluenesulfinate, and then the mixture was extracted by Et_2O. After being dried by Na_2SO_4, the solvent was removed under vacuum at room temperature to provide the corresponding sulfinic acids which were directly used in the catalytic reaction without further purification.[6,7]

Other arylsulfinic acids, heteroaromatic and aliphatic sulfinic acids were prepared according to the following procedures:[8,9] arylsulfonyl chloride (10 mmol) and anhydrous sodium sulfite (30 mmol) were dissolved in 20 mL water. The reaction mixture was heated to 75 °C, and stirred for additional 5 h at 75 °C. After the reaction was complete, the mixture was transferred to separation funnel and washed with chloroform. The aqueous phase was acidified with 12 *N* HCl aq. (1.5 mL) at 0 °C and then extracted by Et_2O. After being dried by Na_2SO_4, the organic solvent was removed under vacuum at room temperature to provide the corresponding sulfinic acids which were directly used in the catalytic reaction without further purification.

2.4 Preparation of the Ynals

Ynals **1a**, **1n-1o**, **1v-1y**, **1af-1ag** were prepared according to literature.[10] The other ynals were prepared according to the following procedures.

General Procedure **A**:

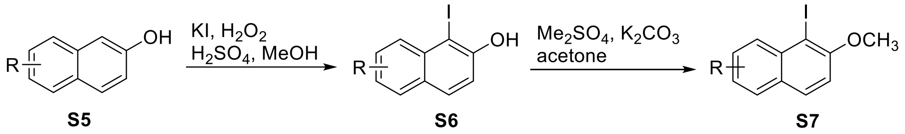

To a stirred solution of sulfuric acid (98%, 1.5 eq.) in methanol (0.2 M) was added **S5** (1.0 eq.), KI (1.0 eq.), and H_2O_2 (35%, 2.0 eq.) at 0 °C. The mixture was stirred at 0 °C for an additional 12 h. The reaction was quenched by adding excess saturated aqueous solution of $Na_2S_2O_3$. The methanol was evaporated, and the residue was extracted with EtOAc for three times. The combined organic phases were washed sequentially with water and brine, dried over anhydrous Na_2SO_4 and concentrated in *vacuo* to afford the product **S6** which was directly used for the next step without further purification.

To a solution of the crude **S6** (1.0 eq.) in anhydrous acetone (0.2 M) was added K_2CO_3 (3.0 eq.) and dimethyl sulfate (2.0 eq.) at room temperature. The resulting mixture was stirred at 60 °C for an additional 10 h, and then filtrated through a pad of silica gel, washed with EtOAc/hexanes =1:3. The combined filtrate was concentrated in *vacuo* to afford the product **S7**

which was directly used for the next step without further purification.

General Procedure **B**:

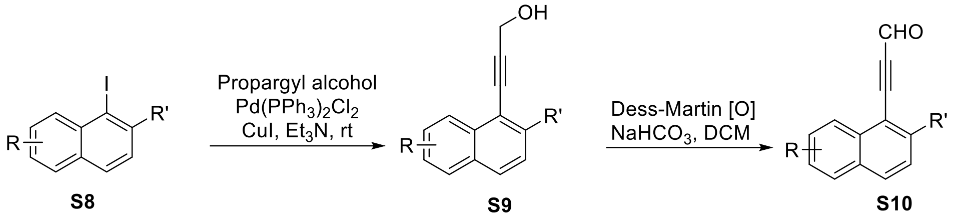

To a solution of **S8** (1.0 eq.) and propargyl alcohol (5.0 eq.) in triethylamine (0.2 M) was added $PdCl_2(PPh_3)_2$ (5 mol%) and CuI (10 mol%) at room temperature. The flask was flushed with nitrogen gas. The resulting mixture was stirred at 40 °C for 20 h. The reaction mixture was filtered and washed with EtOAc/hexanes =1:1. The combined filtrate was concentrated in *vacuo* to provide the crude alcohol **S9** which was directly used for the next step without further purification.

To a solution of **S9** (1.0 eq.) in DCM (0.5 M) was added $NaHCO_3$ (3.0 eq.), and Dess-Martin periodinane (1.3 eq.) at 0 °C. The resulting mixture was stirred at room temperature for 15 min. The reaction was quenched by adding excess saturated aqueous solution of Na_2SO_3, and the mixture was extracted with EtOAc for three times. The combined organic phases were washed sequentially with water and brine, dried over anhydrous Na_2SO_4 and concentrated in *vacuo*. The residue was purified by flash chromatography on silica gel to provide aldehyde **S10**.

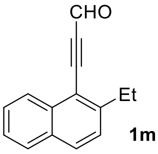

1m

Aldehyde **1m** was prepared from the corresponding aryl iodide according to General Procedure **B**.

^{1}H NMR (400 MHz, $CDCl_3$) δ 9.61 (s, 1H), 8.32 (d, *J* = 8.4 Hz, 1H), 7.88 (d, *J* = 8.5 Hz, 1H), 7.82 (d, *J* = 8.2 Hz, 1H), 7.60 (dd, *J* = 8.4, 6.9 Hz, 1H), 7.53 – 7.47 (m, 1H), 7.38 (d, *J* = 8.5 Hz, 1H), 3.05 (q, *J* = 7.6 Hz, 2H), 1.34 (t, *J* = 7.6 Hz, 3H) ppm.

^{13}C NMR (101 MHz, $CDCl_3$) δ 176.56, 149.70, 134.12, 131.94, 131.49, 128.40, 127.86, 126.58, 126.20, 125.51, 114.62, 97.28, 92.32, 28.52, 15.33 ppm.

HRMS (ESI, m/z): calculated for $C_{15}H_{12}OH^+$: 209.0966 $(M+H)^+$, found: 209.0970.

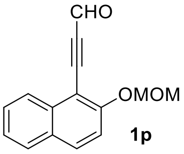

1p

Aldehyde **1p** was prepared from the corresponding aryl iodide[11] according to General Procedure

B.

^{1}H NMR (400 MHz, $CDCl_3$) δ 9.61 (s, 1H), 8.24 (dd, *J* = 8.4, 1.1 Hz, 1H), 7.92 (d, *J* = 9.1 Hz, 1H), 7.82 – 7.78 (m, 1H), 7.63 – 7.57 (m, 1H), 7.47 – 7.40 (m, 2H), 5.40 (s, 2H), 3.57 (s, 3H) ppm.

^{13}C NMR (101 MHz, $CDCl_3$) δ 176.8, 160.1, 134. 8, 133.9, 129.0, 128.5, 128.5, 125.3, 125.0, 115.5, 104.0, 98.2, 95.2, 90.9, 56.7 ppm.

M.P. 52 °C - 53 °C

HRMS (ESI, m/z): calculated for $C_{15}H_{12}O_3Na^+$: 263.0684 $(M+Na)^+$, found: 263.0685.

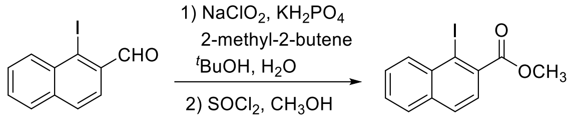

S11 **S12**

To a solution of aldehyde **S11**[12] (1.95 g, 6.9 mmol) in ^{t}BuOH (30 mL) was added 2-methyl-2-butene (12 mL, 142.8 mmol), followed by addition of a solution of KH_2PO_4 (4.6 g, 33.8 mmol) and $NaClO_2$ (80%, 1.9 g, 16.8 mmol) in H_2O (10 mL) at 0 °C. The reaction mixture was slowly warmed to room temperature and stirred for an additional 1 h. The mixture was diluted with brine (10 mL) and extracted with EtOAc (100 mL x 3). The combined organic phases were washed with brine (10 mL x 3), dried over anhydrous Na_2SO_4 and concentrated in *vacuo* to afford the corresponding acid. The acid was dissloved in methanol (20 mL) and cooled to 0 °C. Then thionyl chloride (1.5 mL, 20.7 mmol) was added dropwise. The resulting mixture was warmed to room temperature and stirred for an additional 10 h. The mixture was concentrated in *vacuo* and the residue was purified by flash chromatography on silica gel (EtOAc/hexanes =1:5) to provide **S12** (1.68 g, 78%) as a yellow solid.

^{1}H NMR (400 MHz, $CDCl_3$) δ 8.33 (d, *J* = 8.9 Hz, 1H), 7.80 (d, *J* = 8.4 Hz, 1H), 7.74 (dd, *J* = 7.8, 1.5 Hz, 1H), 7.63 – 7.49 (m, 3H), 4.01 (s, 3H) ppm.

^{13}C NMR (101 MHz, $CDCl_3$) δ 169.0, 136.8, 134.8, 134.4, 134.1, 129.0, 128.5, 128.4, 128.0, 125.5, 100.5, 52.8 ppm.

HRMS (ESI, m/z): calculated for $C_{12}H_9IO_2Na^+$: 334.9545 $(M+Na)^+$, found: 334.9543.

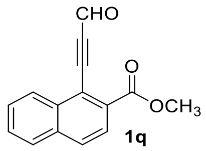

1q

Aldehyde **1q** was prepared from iodide **S12** according to General Procedure **B**.

^{1}H NMR (400 MHz, $CDCl_3$) δ 9.62 (s, 1H), 8.43 – 8.37 (m, 1H), 7.96 (d, *J* = 8.7 Hz, 1H), 7.91 (d, *J* = 8.7 Hz, 1H), 7.86 – 7.80 (m, 1H), 7.65 – 7.55 (m, 2H), 3.98 (s, 3H) ppm.

^{13}C NMR (101 MHz, $CDCl_3$) δ 176.8, 166.1, 134.3, 133.5, 132.7, 131.1, 128.8, 128.5, 128.4, 126.9, 125.8, 118.4, 97.8, 90.9, 52.6 ppm.
M.P. 87 °C - 88 °C
HRMS (ESI, m/z): calculated for $C_{15}H_{10}O_3H^+$: 239.0708 $(M+H)^+$, found: 239.0708.

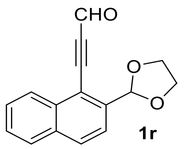

1r

Aldehyde **1r** was prepared from the corresponding aryl iodide[12] according to General Procedure **B**.
^{1}H NMR (400 MHz, $CDCl_3$) δ 9.61 (s, 1H), 8.38 (dd, *J* = 8.4, 1.2 Hz, 1H), 8.00 (d, *J* = 8.6 Hz, 1H), 7.88 (d, *J* = 7.5 Hz, 1H), 7.72 (d, *J* = 8.6 Hz, 1H), 7.65 (ddd, *J* = 8.4, 6.8, 1.4 Hz, 1H), 7.58 (ddd, *J* = 8.2, 6.9, 1.3 Hz, 1H), 6.41 (s, 1H), 4.32 – 4.19 (m, 2H), 4.21 – 4.08 (m, 2H) ppm.
^{13}C NMR (101 MHz, $CDCl_3$) δ 176.5, 141.5, 133.7, 133.4, 132.1, 128.6, 128.2, 127.6, 126.0, 123.4, 116.4, 102.0, 97.8, 90.0, 66.0 ppm.
HRMS (ESI, m/z): calculated for $C_{16}H_{12}O_3H^+$: 253.0865 $(M+Na)^+$, found: 253.0866.

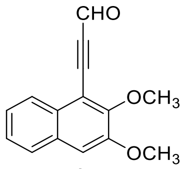

1s

Aldehyde **1s** was prepared from 2,3-dihydroxynaphthalene according to General Procedures **A** and **B** sequentially.
^{1}H NMR (400 MHz, $CDCl_3$) δ 9.60 (s, 1H), 8.19 – 8.13 (m, 1H), 7.76 – 7.70 (m, 1H), 7.52 – 7.43 (m, 2H), 7.31 (s, 1H), 4.11 (s, 3H), 4.00 (s, 3H) ppm.
^{13}C NMR (101 MHz, $CDCl_3$) δ 176.7, 155.0, 151.6, 130.6, 129.4, 127.1, 126.5, 125.9, 125.1, 111.6, 109.3, 97.3, 90.1, 62.1, 56.1 ppm.
M.P. 84 °C - 85 °C
HRMS (ESI, m/z): calculated for $C_{15}H_{12}O_3H^+$: 241.0865 $(M+H)^+$, found: 241.0863.

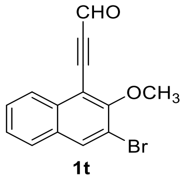

1t

Aldehyde **1t** was prepared from 3-bromo-2-naphthol according to General Procedures **A** and **B**

sequentially.

^{1}H NMR (400 MHz, $CDCl_3$) δ 9.60 (s, 1H), 8.22 – 8.14 (m, 2H), 7.75 – 7.68 (m, 1H), 7.65 – 7.56 (m, 1H), 7.54 – 7.45 (m, 1H), 4.13 (s, 2H) ppm.

^{13}C NMR (101 MHz, $CDCl_3$) δ 176.4, 159.6, 136.3, 133.8, 130.7, 128.5, 127.5, 126.9, 125.3, 116.5, 110.0, 97.8, 88.8, 62.5 ppm.

M.P. 79 °C - 80 °C

HRMS (ESI, m/z): calculated for $C_{14}H_9BrO_2H^+$: 288.9864 $(M+H)^+$, found: 288.9861.

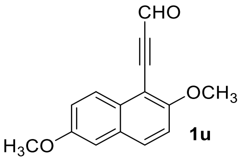

1u

Aldehyde **1u** was prepared from 2,6-dihydroxynaphthalene according to General Procedures **A** and **B** sequentially.

^{1}H NMR (400 MHz, $CDCl_3$) δ 9.57 (s, 1H), 8.10 (d, J = 9.1 Hz, 1H), 7.83 (d, J = 9.1 Hz, 1H), 7.25 (dd, J = 9.1, 2.7 Hz, 1H), 7.19 (d, J = 9.1 Hz, 1H), 7.08 (d, J = 2.6 Hz, 1H), 4.01 (s, 3H), 3.89 (s, 3H) ppm.

^{13}C NMR (101 MHz, $CDCl_3$) δ 176.8, 160.7, 156.9, 132.8, 130.2, 129.4, 126.3, 121.3, 112.7, 106.6, 102.4, 98.3, 91.5, 56.7, 55.5 ppm.

M.P. 120 °C - 121 °C.

HRMS (ESI, m/z): calculated for $C_{15}H_{12}O_3H^+$: 241.0865 $(M+H)^+$, found: 241.0862.

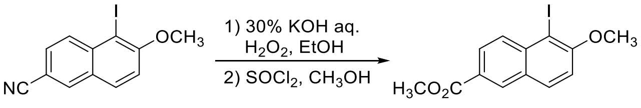

S13 S14

To a solution of aldehyde **S13** (1.0 g, 3.2 mmol) in ethanol (10 mL) was added 30% KOH aq. (10 mL) and H_2O_2 (35%, 10 mL) at room temperature. The reaction mixture was heated to 80 °C for 12 h. After cooled to 0 °C, the mixture was acidified to pH=3 with 2.0 N HCl aq. and then extracted with EtOAc (100 mL x 3). The combined organic phases were washed with brine (10 mL), dried over anhydrous Na_2SO_4 and concentrated in *vacuo* to afford the corresponding acid. The acid was dissloved in methanol (10 mL) and cooled to 0 °C. Then thionyl chloride (0.7 mL, 9.6 mmol) was added dropwise. The resulting mixture was warmed to room temperature and stirred for an additional 10 h. The mixture was concentrated in *vacuo* and the residue was purified by flash chromatography on silica gel (EtOAc/hexanes =1:3) to provide **S14** (645.7 mg, 59%) as a yellow solid.

^{1}H NMR (500 MHz, $CDCl_3$) δ 8.42 (d, *J* = 1.7 Hz, 1H), 8.11 (d, *J* = 8.9 Hz, 1H), 8.03 (dd, *J* = 8.9, 1.7 Hz, 1H), 7.84 (d, *J* = 8.9 Hz, 1H), 7.17 (d, *J* = 8.9 Hz, 1H), 4.00 (s, 3H), 3.96 (s, 3H) ppm.
^{13}C NMR (126 MHz, $CDCl_3$) δ 166.9, 158.4, 137.9, 131.9, 131.4, 131.4, 128.7, 127.4, 125.8, 113.3, 87.3, 57.2, 52.3 ppm.
HRMS (ESI, m/z): calculated for $C_{13}H_{11}IO_3H^+$: 342.9831 $(M+H)^+$, found: 342.9828.

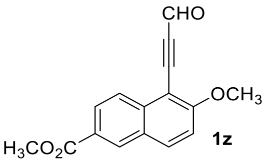

1z

Aldehyde **1z** was prepared from iodide **S10** according to General Procedure **B**.
^{1}H NMR (400 MHz, $CDCl_3$) δ 9.60 (s, 1H), 8.56 (d, *J* = 1.7 Hz, 1H), 8.24 (d, *J* = 8.8 Hz, 1H), 8.15 (dd, *J* = 8.8, 1.7 Hz, 1H), 8.07 (d, *J* = 9.2 Hz, 1H), 7.32 (d, *J* = 9.2 Hz, 1H), 4.09 (s, 3H), 3.98 (s, 3H) ppm.
^{13}C NMR (101 MHz, $CDCl_3$) δ 176.7, 166.9, 163.7, 137.5, 135.4, 131.6, 128.0, 127.4, 126.6, 125.2, 113.0, 102.6, 98.5, 90.2, 56.8, 52.5 ppm.
HRMS (ESI, m/z): calculated for $C_{16}H_{12}O_4H^+$: 269.0814 $(M+H)^+$, found: 269.0813.

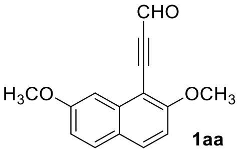

1aa

Aldehyde **1aa** was prepared from 2,7-dihydroxynaphthalene according to General Procedures **A** and **B** sequentially.
^{1}H NMR (400 MHz, $CDCl_3$) δ 9.58 (s, 1H), 7.81 (d, *J* = 9.1 Hz, 1H), 7.63 (d, *J* = 8.9 Hz, 1H), 7.43 (d, *J* = 2.5 Hz, 1H), 7.05 – 6.98 (m, 2H), 4.00 (s, 3H), 3.94 (s, 3H) ppm.
^{13}C NMR (101 MHz, $CDCl_3$) δ 176.6, 162.8, 160.1, 137.0, 133.9, 130.1, 123.7, 117.5, 109.2, 103.2, 101.0, 99.0, 91.8, 56.4, 55.5 ppm.
M.P. 99 °C - 100 °C.
HRMS (ESI, m/z): calculated for $C_{15}H_{12}O_3H^+$: 241.0865 $(M+H)^+$, found: 241.0864.

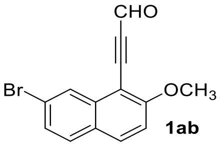

1ab

Aldehyde **1ab** was prepared from 2-bromo-7-hydroxynaphthalene according to General Procedures **A** and **B** sequentially.

^{1}**H NMR** (500 MHz, $CDCl_3$) δ 9.60 (s, 1H), 8.33 (d, *J* = 1.9 Hz, 1H), 7.91 (d, *J* = 9.1 Hz, 1H), 7.65 (d, *J* = 8.6 Hz, 1H), 7.48 (dd, *J* = 8.7, 1.9 Hz, 1H), 7.25 (d, *J* = 9.3 Hz, 1H), 4.06 (s, 3H) ppm.

^{13}**C NMR** (126 MHz, $CDCl_3$) δ 176.7, 162.8, 136.1, 134.0, 130.1, 128.5, 127.0, 126.8, 123.6, 112.5, 101.6, 98.6, 90.2, 56.7 ppm.

M.P. 136 °C - 137 °C.

HRMS (ESI, m/z): calculated for $C_{14}H_9BrO_2H^+$: 288.9864 $(M+H)^+$, found: 288.9859.

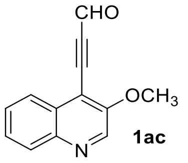

1ac

Aldehyde **1ac** was prepared from 3-hydroxyquinoline according to General Procedures **A** and **B** sequentially.

^{1}**H NMR** (400 MHz, $CDCl_3$) δ 9.56 (s, 1H), 8.80 (s, 1H), 8.11 – 7.98 (m, 2H), 7.64 – 7.52 (m, 2H), 4.14 (s, 3H) ppm.

^{13}**C NMR** (101 MHz, $CDCl_3$) δ 176.3, 154.6, 143.4, 138.4, 129.9, 128.9, 127.9, 124.5, 109.7, 99.2, 86.5, 57.5 ppm.

M.P. 112 °C - 113 °C.

HRMS (ESI, m/z): calculated for $C_{13}H_9NO_2H^+$: 212.0712 $(M+H)^+$, found: 212.0717.

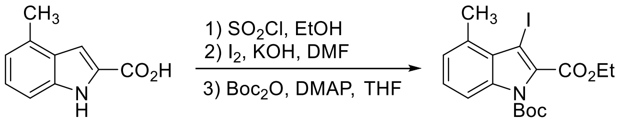

S15 **S16**

To a solution of **S15** (1.0 g, 5.7 mmol) in ethanol (10 ml) was added thionyl chloride (2.1 mL, 28.5 mmol) dropwise at 0 °C. The resulting mixture was warmed to room temperature and stirred for an additional 10 h. The mixture was concentrated in *vacuo* to afford the corresponding ester which was redissolved in DMF (4.5 ml). To this solution was added KOH (1.2 g, 21.1 mmol) at room temperature. After 15 min, a solution of iodine (1.4 g, 5.7 mmol) in DMF (4.5 mL) was added dropwise at room temperature. The resulting mixture was stirred for an additional 45 min and then poured into a cold solution of $Na_2S_2O_3$. The precipitate was filterated and dried in *vacuo* to give a yellow solid. The solid was redissolved in THF (30 mL). Then Boc2O (1.4 mL, 6.1 mmol) and DMAP (6.7 mg, 0.06 mmol) were added at room temperature. The mixture was stirred for an additional 2 h before being concentated in *vacuo*. The residue was purified by flash chromatography on silica gel (EtOAc/hexanes =1:10) to provide **S16** (1.6 g, 67%) as a yellow solid.

^{1}H NMR (400 MHz, $CDCl_3$) δ 8.06 (d, *J* = 8.5 Hz, 1H), 7.26 (dd, *J* = 8.5, 7.3 Hz, 1H), 7.03 (d, *J* = 7.3 Hz, 0H), 4.45 (q, *J* = 7.2 Hz, 2H), 2.88 (s, 3H), 1.62 (s, 9H), 1.44 (t, *J* = 7.2 Hz, 3H) ppm.
^{13}C NMR (101 MHz, $CDCl_3$) δ 162.9, 148.2, 136.0, 133.5, 132.7, 126.7, 126.5, 126.2, 113.4, 85.5, 66.1, 62.2, 28.0, 20.1, 14.3 ppm.
HRMS (ESI, m/z): calculated for $C_{17}H_{20}INO_4Na^+$: 452.0335 $(M+Na)^+$, found: 452.0334.

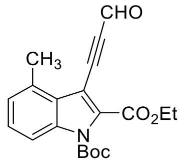

1ad

Aldehyde **1ad** was prepared from iodide **S16** according to General Procedure **B.**
^{1}H NMR (400 MHz, $CDCl_3$) δ 9.43 (s, 1H), 7.94 (d, *J* = 8.5 Hz, 1H), 7.36 – 7.27 (m, 1H), 7.06 (d, *J* = 7.3 Hz, 1H), 4.48 (q, *J* = 7.2 Hz, 2H), 2.76 (s, 3H), 1.63 (s, 9H), 1.46 (t, *J* = 7.1 Hz, 3H) ppm.
^{13}C NMR (101 MHz, $CDCl_3$) δ 176.0, 160.9, 148.0, 137.2, 135.8, 132.9, 127.4, 125.9, 125.9, 112.9, 102.7, 94.6, 89.0, 86.3, 62.5, 27.8, 19.0, 14.3 ppm.
M.P. 106 °C - 107 °C.
HRMS (ESI, m/z): calculated for $C_{20}H_{21}NO_5Na^+$: 378.1317 $(M+Na)^+$, found: 378.1317.

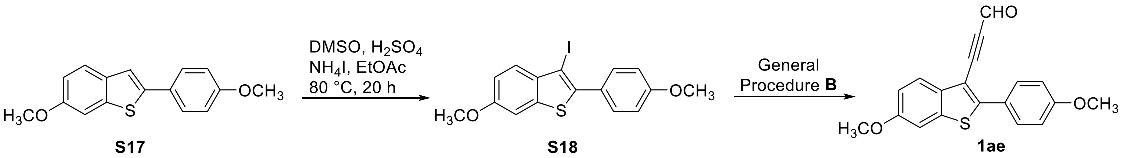

To a solution of **S17** (1.0 g, 3.7 mmol) in EtOAc (15 mL) was added H_2SO_4 (98%, 0.6 mL, 11.1 mmol), DMSO (1.6 mL, 22.2 mmol) and NH_4I (1.1g, 7.4 mmol) at room temperature. The mixture was heated to 80 °C and stirred for 20 h. The reaction was quenched by adding excess saturated aqueous solution of $Na_2S_2O_3$ at 0 °C. The mixture was extracted with EtOAc (50 mL x 3). The combined organic phases were washed sequentially with water (20 mL) and brine (20 mL), dried over anhydrous Na_2SO_4 and concentrated in *vacuo* to afford crude iodide **S18** (1.3 g, 88%) which was directly used for the next step without further purification. **S18** was further converted to aldehyde **1ae** according to General Procedure **B**.
^{1}H NMR (400 MHz, $CDCl_3$) δ 9.49 (s, 1H), 7.88 – 7.78 (m, 3H), 7.26 (d, *J* = 2.2 Hz, 1H), 7.08 (dd, *J* = 8.8, 2.3 Hz, 1H), 7.04 – 6.98 (m, 2H), 3.89 (s, 3H), 3.88 (s, 3H) ppm.
^{13}C NMR (101 MHz, $CDCl_3$) δ 176.4, 160.9, 158.5, 152.3, 138.5, 134.6, 129.9, 125.7, 123.7, 115.4, 114.6, 108.6, 105.0, 93.9, 91.1, 55.8, 55.6 ppm.
M.P. 131 °C - 132 °C
HRMS (ESI, m/z): calculated for $C_{19}H_{14}O_3SH^+$: 323.0742 $(M+H)^+$, found: 323.0738.

3. Supplementary Results of Condition Optimization

Supplementary Table 1. Preliminary Screening of Nucleophiles[a]

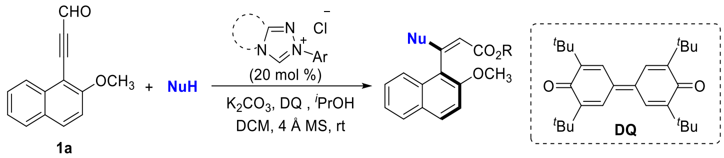

Entry	NuH	Yield[b]
1	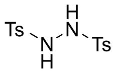	trace
2	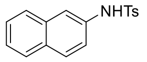	NP
3	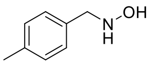	NP
4	$PhSO_2H$	20%
5	$PhSO_2Na$	trace
6	$Ph_2P(O)H$	NP
8	Ph_2PH	NP

[a] Reaction conditions: **1a** (0.05 mmol), **NuH** (0.1 mmol), NHC precatalyst (20 mol%), K_2CO_3 (0.15 mmol), DQ (0.1 mmol), ^{i}PrOH (0.055 mmol), CH_2Cl_2 (1 mL), and 4 Å molecular sieve (100 mg), room temperature, 12 h. [b] Estimated via ^{1}H NMR analysis of the crude reaction mixture with 1,1,2,2-tetrachloroethane as an internal standard. NP = no desired product.

Supplementary Table 2. Screening of Solvents[a]

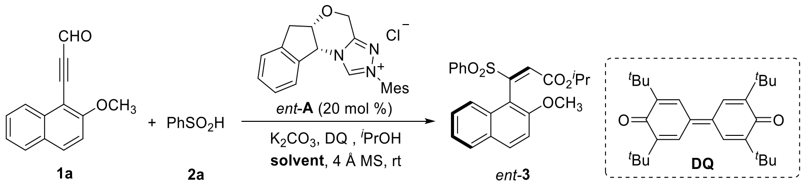

Entry	Solvent	Yield[b]	*e.r.*[c]	*E*/*Z*[d]
1	CH_2Cl_2	37%	65:35	>20:1
2	DCE	41%	66:34	>20:1
3	$CHCl_3$	NP	-	-
4	THF	NP	-	-
5	DME	NP	-	-
6	MeCN	NP	-	-
7	toluene	27%	64:36	>20:1
8	Et_2O	42%	70:30	>20:1

[a] Reaction conditions: **1a** (0.05 mmol), **2a** (0.1 mmol), NHC precatalyst **A** (20 mol%), K_2CO_3 (0.15 mmol), DQ (0.1 mmol), ^{i}PrOH (0.055 mmol), solvent (1 mL), and 4 Å molecular sieve (100 mg), room temperature, 12 h. [b] Estimated via ^{1}H NMR analysis of the crude reaction mixture with 1,1,2,2-tetrachloroethane as an internal standard. [c] Determined via HPLC on a chiral stationary phase. [d] Determined via ^{1}H NMR analysis of the crude reaction mixture. Mes = 2,4,6-trimethylphenyl. DCE = 1,2-dichloroethane.

Supplementary Table 3. Screening of Bases[a]

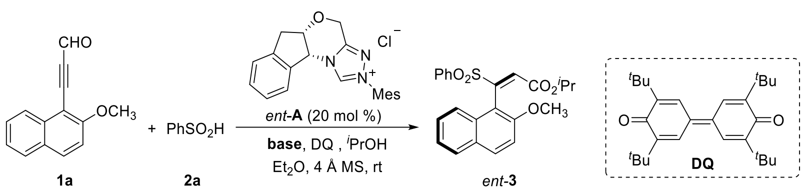

Entry	Base	Yield[b]	*e.r.* [c]	*E*/*Z*[d]
1	Cs_2CO_3	trace	-	-
2	Na_2CO_3	24%	72:28	>20:1
3	K_3PO_4	trace	-	-
4	KOAc	40%	75:25	>20:1
5	NaOAc	54%	78:22	>20:1
6	DIPEA	trace	-	-
7	DBU	trace	-	-
8	DMAP	NP	-	-

[a] Reaction conditions: **1a** (0.05 mmol), **2a** (0.1 mmol), NHC precatalyst **A** (20 mol%), base (0.15 mmol), DQ (0.1 mmol), ^{i}PrOH (0.055 mmol), Et_2O(1 mL), and 4 Å molecular sieve (100 mg), room temperature, 12 h. [b] Estimated via ^{1}H NMR analysis of the crude reaction mixture with 1,1,2,2-tetrachloroethane as an internal standard. [c] Determined via HPLC on a chiral stationary phase. [d] Determined via ^{1}H NMR analysis of the crude reaction mixture. Mes = 2,4,6-trimethylphenyl. DIPEA = *N*, *N*-Diisopropylethylamine. DBU = 1,8-diazabicyclo[5.4.0]undec-7-ene. DMAP = 4-dimethylaminopyridine

Supplementary Table 4. Screening of NHC Pre-catalysts[a]

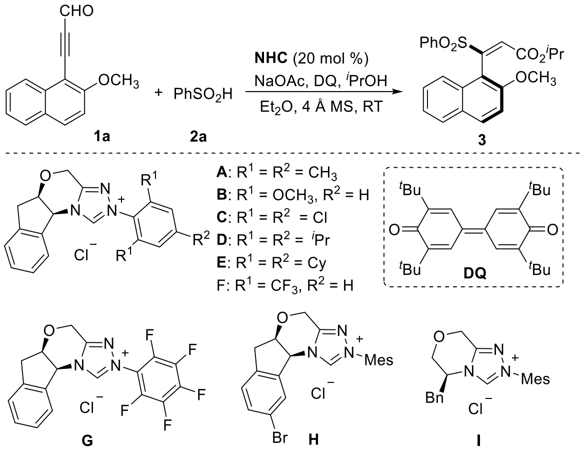

Entry	NHC	Yield[b]	*e.r.* [c]	*E*/*Z*[d]
1	*ent*-**A**	54	22:78	>20:1
2	B	40	78:22	>20:1
3	C	NP	-	-
4	D	37%	85:15	>20:1
5	E	40%	87:13	>20:1
6	F	22%	83:17	>20:1
7	G	29%	60:40	>20:1

8	H	10%	76:24	>20:1
9	I	63%	52:48	>20:1

[a] Reaction conditions: **1a** (0.05 mmol), **2a** (0.1 mmol), NHC precatalyst (20 mol%), NaOAc (0.15 mmol), DQ (0.1 mmol), ^{i}PrOH (0.055 mmol), Et_2O(1 mL), and 4 Å molecular sieve (100 mg), room temperature, 12 h. [b] Estimated via ^{1}H NMR analysis of the crude reaction mixture with 1,1,2,2-tetrachloroethane as an internal standard. [c] Determined via HPLC on a chiral stationary phase. [d] Determined via ^{1}H NMR analysis of the crude reaction mixture. Mes = 2,4,6-trimethylphenyl.

4. Proposed Reaction Mechanism

The proposed reaction mechanism is described in Supplementary Fig. 189. The free NHC catalyst was generated in situ from the pre-catalyst **E** after deprotonation, and then reacted with the ynal substrate (**1a**) to afford the Breslow intermediate **I**. The Breslow intermediate **I** was further oxidized by DQ to give acetylenic acylazolium intermediate **II**, which then reacted with in situ generated sulfinic anion to provide the allenolate **III**. *E*-Selective protonation of the allenolate **III** gave rise to the acylazolium intermediate **IV**, which was then trapped by a hard nucleophile (phenol) to afford the corresponding axially chiral styrene (**9a**) and release the NHC catalyst for additional catalytic cycles.

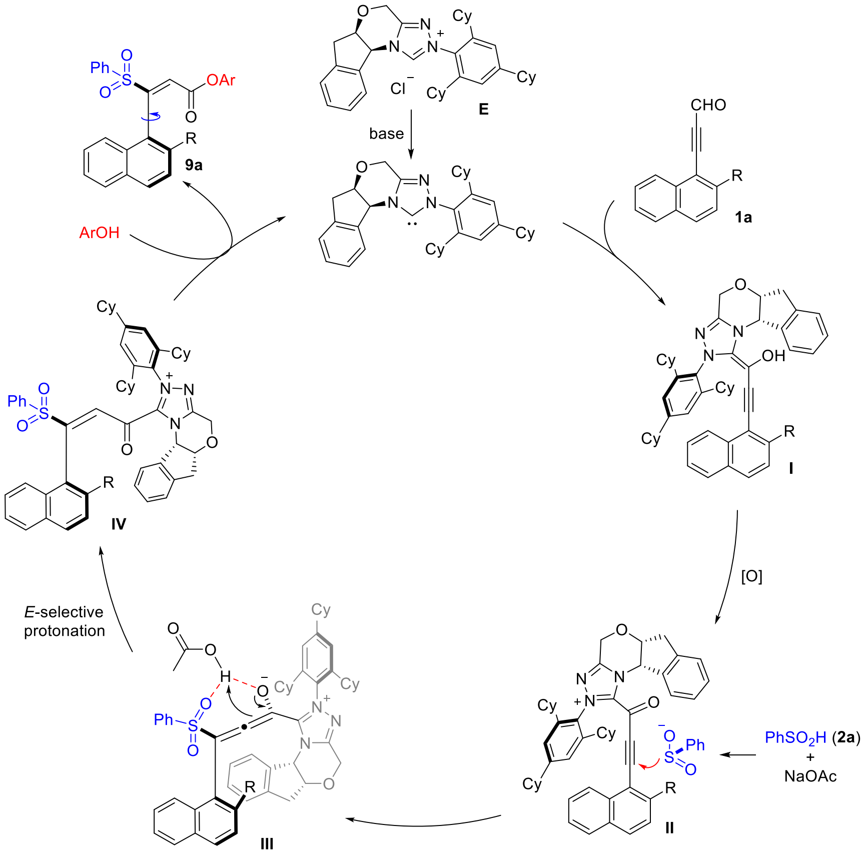

Supplementary Figure 189. Proposed Reaction Mechanism

For the substrates with 2-alkoxyl substitution (e.g., OMe) on the naphthalene ring, the reaction generally provided better results than those without ether groups. We hypothesized that the 2-

alkoxyl substituent may have effect on the reaction outcomes. Inspired by the coordination effect of Lewis acid with nucleophiles and 2-methoxyl substituted acetylenic acylazolium intermediates,[10] we proposed that the hydrogen bond interaction between nucleophiles and 2-alkoxyl substituents played an important role in controlling the enantio- and *E*/*Z* selectivity (Supplementary Figure 190).

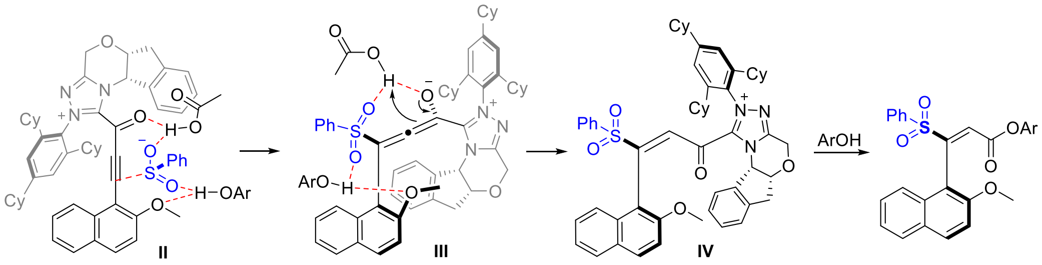

Supplementary Figure 190. Plausible Hydrogen Bond Interaction

Besides, the presence of 2-alkoxyl substitutions (e.g., OMe) might have other functions which benefit the reaction. For example, the high active acetylenic acylazolium intermediate (**II**) may resonate to intermediate **II'** which may react with sulfinic anion to provide allenolate **III** (Supplementary Fig. 191). The allenolate **III** then undergoes protonation and esterification to afford the product.

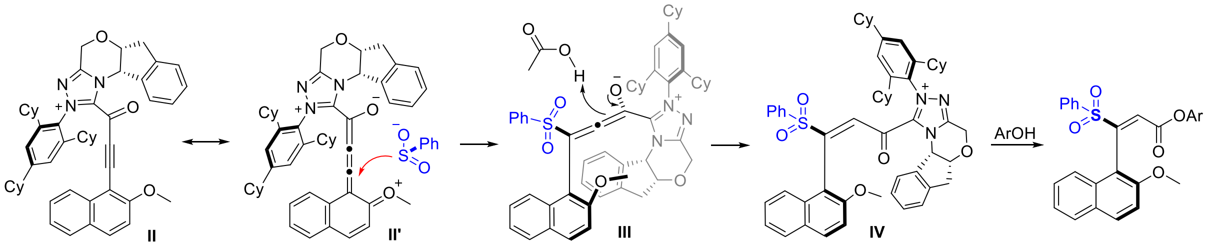

Supplementary Figure 191.Plausible Resonance Intermediates Involved Pathway

For most of the substrates, the naphthalene rings on the ynal substrates provided enough steric hindrance to prevent the rotation of allenolate intermediate (**III**). However, for **9ae-9ag**, the less steric hindrance of aromatic rings on the ynals might lead to more flexibility of the allenolate intermediates (**III**), which may rotate from **III** to **III'** (Supplementary Fig. 192). The hydrogen bond interaction modes for intermediates **III** and **III'** were different, and intermediate **III'** would prefer to perform the *Z*-selective protonation, which then resulted in the non-axially chiral product with *Z*-alkene.

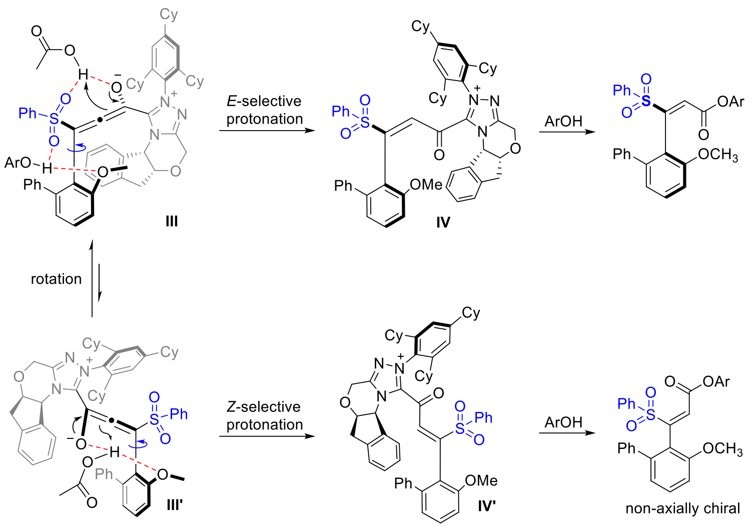

Supplementary Figure 192. Plausible Reasons for the Low *E*/*Z* Selectivity of **9ae-9ag**

5. Supplementary Data of the Products

5.1 Characterization of the Axially Chiral Styrenes

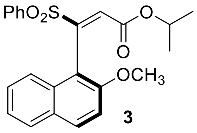

Light yellow oil, 48% yield, *E*/*Z* >20:1.

^{1}H NMR (500 MHz, $CDCl_3$) δ 7.85 (d, *J* = 9.0 Hz, 1H), 7.73 (d, *J* = 8.1 Hz, 1H), 7.61 – 7.56 (m, 2H), 7.56 – 7.50 (m, 2H), 7.48 (s, 1H), 7.38 – 7.33 (m, 3H), 7.30 (ddd, *J* = 8.0, 6.8, 1.2 Hz, 1H), 7.02 (d, *J* = 9.1 Hz, 1H), 4.73 – 4.62 (m, 1H), 3.34 (s, 3H), 0.73 (d, *J* = 6.2 Hz, 3H), 0.65 (d, *J* = 6.2 Hz, 3H) ppm.

^{13}C NMR (126 MHz, $CDCl_3$) δ 163.2, 154.3, 151.2, 138.4, 133.7, 133.1, 131.8, 131.7, 129.6, 128.5, 127.8, 127.2, 124.8, 124.0, 113.0, 111.8, 68.7, 55.7, 21.2, 21.1 ppm.

HRMS (ESI, m/z): calculated for $C_{23}H_{22}O_5SH^+$: 411.1266 $(M+H)^+$, found: 411.1263.

$[\alpha]^{21}_D$ = -27.5 (c = 0.6 in $CHCl_3$).

HPLC analysis: 91:9 *e.r.* (CHIRALPAK AD-H column, ^{i}PrOH/Hexane = 20:80, 0.5 mL/min, λ = 254 nm, t_{major} = 14.1 min, t_{minor} = 19.5 min).

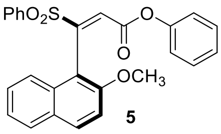

Light yellow solid, 90% yield, *E*/*Z* 20:1.

^{1}H NMR (500 MHz, $CDCl_3$) δ 7.85 (d, *J* = 9.1 Hz, 1H), 7.77 – 7.72 (m, 2H), 7.65 – 7.58 (m, 3H), 7.58 – 7.53 (m, 1H), 7.42 – 7.35 (m, 3H), 7.32 (ddd, *J* = 8.0, 6.8, 1.2 Hz, 1H), 7.21 – 7.15 (m, 2H), 7.11 – 7.05 (m, 1H), 7.02 (d, *J* = 9.1 Hz, 1H), 6.69 – 6.65 (m, 2H), 3.36 (s, 3H) ppm.

^{13}C NMR (126 MHz, $CDCl_3$) δ 161.9, 154.3, 153.8, 150.1, 138.1, 133.9, 132.8, 132.1, 130.2, 129.7, 129.3, 128.6, 128.0, 127.4, 126.1, 124.6, 124.1, 121.0, 112.3, 111.9, 55.7 ppm.

M.P. 51 °C - 52 °C

HRMS (ESI, m/z): calculated for $C_{26}H_{20}O_5SH^+$: 445.1110 $(M+H)^+$, found: 445.1110.
$[\alpha]^{21}_D$ = -19.4 (c = 2.0 in $CHCl_3$).
HPLC analysis: 86:14 *e.r.* (CHIRALPAK AD-H column, ^{i}PrOH/Hexane = 20:80, 0.5 mL/min, λ = 220 nm, t_{major} = 19.1 min, t_{minor}= 20.8 min).

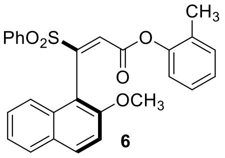

6

Light yellow oil, 91% yield, *E*/*Z* >20:1.
^{1}H NMR (400 MHz, $CDCl_3$) δ 7.82 (d, *J* = 9.1 Hz, 1H), 7.77 (s, 1H), 7.72 (dd, *J* = 7.9, 1.2 Hz, 1H), 7.67 – 7.63 (m, 1H), 7.63 – 7.58 (m, 2H), 7.58 – 7.53 (m, 1H), 7.42 – 7.28 (m, 4H), 7.06 – 6.96 (m, 4H), 6.67 – 6.60 (m, 1H), 3.35 (s, 3H), 1.74 (s, 3H) ppm.
^{13}C NMR (101 MHz, $CDCl_3$) δ 162.0, 154.3, 153.4, 148.7, 138.0, 133.9, 132.9, 132.2, 131.1, 130.1, 129.9, 129.7, 128.6, 128.6, 128.0, 127.4, 126.8, 126.3, 124.7, 124.1, 121.4, 112.3, 111.75, 55.6, 15.8 ppm.
HRMS (ESI, m/z): calculated for $C_{27}H_{22}O_5SH^+$: 459.1266 $(M+H)^+$, found: 459.1267.
$[\alpha]^{21}_D$ = -15.5 (c = 2.1 in $CHCl_3$).
HPLC analysis: 83:17 *e.r.* (CHIRALPAK AD-H column, ^{i}PrOH/Hexane = 20:80, 0.5 mL/min, λ = 220 nm, t_{major} = 18.2 min, t_{minor}= 22.5 min).

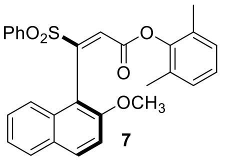

7

Light yellow oil, 65% yield, *E*/*Z* >20:1.
^{1}H NMR (400 MHz, $CDCl_3$) δ 7.81 (d, *J* = 9.1 Hz, 1H), 7.78 (s, 1H), 7.73 – 7.65 (m, 2H), 7.64 – 7.58 (m, 2H), 7.58 – 7.53 (m, 1H), 7.42 – 7.34 (m, 3H), 7.30 (ddd, *J* = 8.1, 6.9, 1.2 Hz, 1H), 6.97 (d, *J* = 9.0 Hz, 1H), 6.93 – 6.82 (m, 3H), 3.34 (s, 3H), 1.74 (s, 6H) ppm.
^{13}C NMR (101 MHz, $CDCl_3$) δ 161.6, 154.2, 153.2, 147.6, 138.0, 133.9, 133.0, 132.2, 130.1, 129.9, 129.8, 128.6, 128.6, 128.5, 127.9, 127.4, 126.1, 124.8, 124.1, 112.3, 111.6, 55.4, 16.0 ppm.
HRMS (ESI, m/z): calculated for $C_{28}H_{24}O_5SH^+$: 473.1423 $(M+H)^+$, found: 473.1423.
$[\alpha]^{21}_D$ = -1.8 (c = 4.3 in $CHCl_3$).
HPLC analysis: 84:16 *e.r.* (CHIRALCEL AD-H column, ^{i}PrOH/Hexane = 20:80, 0.5 mL/min, λ = 220 nm, t_{major} = 15.8 min, t_{minor}= 18.5 min).

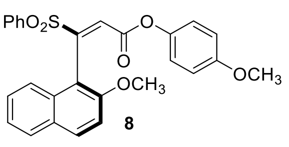

8

Light yellow solid, 97% yield, *E*/*Z* 15:1.

¹H NMR (500 MHz, $CDCl_3$) δ 7.84 (d, *J* = 9.0 Hz, 1H), 7.75 – 7.72 (m, 1H), 7.71 (s, 1H), 7.63 – 7.59 (m, 3H), 7.57 – 7.53 (m, 1H), 7.41 – 7.35 (m, 3H), 7.32 (ddd, *J* = 8.0, 6.8, 1.2 Hz, 1H), 7.01 (d, *J* = 9.1 Hz, 1H), 6.70 – 6.66 (m, 2H), 6.60 – 6.55 (m, 2H), 3.68 (s, 3H), 3.36 (s, 3H) ppm.

¹³C NMR (126 MHz, $CDCl_3$) δ 162.3, 157.4, 154.4, 153.5, 143.6, 138.1, 133.9, 132.8, 132.1, 130.3, 129.7, 128.6, 128.0, 127.4, 124.6, 124.1, 121.8, 114.3, 112.4, 111.9, 55.7, 55.6 ppm.

M.P. 49 ℃ - 50 ℃.

HRMS (ESI, m/z): calculated for $C_{27}H_{22}O_6SH^+$: 475.1215 $(M+H)^+$, found: 475.1215.

[α]^{21}D = -19.3 (c = 2.3 in $CHCl_3$).

HPLC analysis: >99:1 *e.r.* (CHIRALPAK AD-H column, ^{i}PrOH/Hexane = 24:76, 0.5 mL/min, λ = 220 nm, t_{major} = 24.6 min, t_{minor}= 25.8 min).

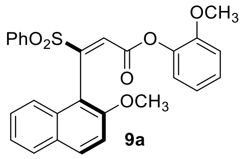

9a

Light yellow oil, 94% yield, *E*/*Z* >20:1.

¹H NMR (500 MHz, $CDCl_3$) δ 7.82 (d, *J* = 9.1 Hz, 1H), 7.77 (s, 1H), 7.72 (d, *J* = 8.2 Hz, 1H), 7.64 (d, *J* = 8.5 Hz, 1H), 7.60 (dd, *J* = 7.7, 1.2 Hz, 2H), 7.57 – 7.52 (m, 1H), 7.41 – 7.34 (m, 3H), 7.33 – 7.28 (m, 1H), 7.08 – 7.03 (m, 1H), 7.00 (d, *J* = 9.0 Hz, 1H), 6.76 (ddd, *J* = 15.2, 7.9, 1.4 Hz, 2H), 6.64 (dd, *J* = 8.0, 1.6 Hz, 1H), 3.50 (s, 3H), 3.37 (s, 3H) ppm.

¹³C NMR (126 MHz, $CDCl_3$) δ 161.4, 154.5, 153.5, 150.9, 139.1, 138.1, 133.8, 132.9, 132.1, 130.0, 129.7, 128.6, 128.6, 127.9, 127.2, 127.1, 124.7, 123.9, 122.3, 120.6, 112.5, 112.3, 111.9, 55.7, 55.7 ppm.

HRMS (ESI, m/z): calculated for $C_{27}H_{22}O_6SH^+$: 475.1215 $(M+H)^+$, found: 475.1215.

[α]^{21}D = -13.4 (c = 4.9 in CHCl3).

HPLC analysis: >99:1 *e.r.* (CHIRALPAK AD-H column, ^{i}PrOH/Hexane = 24:76, flow rate = 0.5 mL/min, λ = 254 nm, t_{major} = 18.0 min, t_{minor}= 23.0 min).

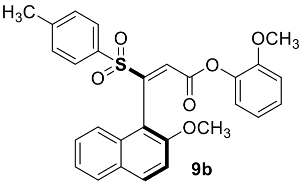

9b

Light yellow solid, 87% yield, *E*/*Z* >20:1

¹H NMR (500 MHz, $CDCl_3$) δ 7.82 (d, *J* = 9.0 Hz, 1H), 7.74 (s, 1H), 7.72 (dd, *J* = 8.1, 1.3 Hz, 1H), 7.65 – 7.62 (m, 1H), 7.50 – 7.46 (m, 2H), 7.38 (ddd, *J* = 8.4, 6.8, 1.3 Hz, 1H), 7.30 (ddd, *J* = 8.0, 6.8, 1.1 Hz, 1H), 7.16 (d, *J* = 8.1 Hz, 2H), 7.08 – 7.00 (m, 2H), 6.79 – 6.73 (m, 2H), 6.64 (dd, *J* = 7.9, 1.7 Hz, 1H), 3.50 (s, 3H), 3.40 (s, 3H), 2.38 (s, 3H) ppm.

¹³C NMR (126 MHz, $CDCl_3$) δ 161.4, 154.5, 153.7, 150.9, 144.9, 139.2, 135.0, 132.9, 131.9, 129.8, 129.5, 129.2, 128.6, 127.9, 127.1, 124.8, 123.9, 122.3, 120.9, 112.5, 112.0, 55.7, 55.6, 21.7 ppm.

M.P. 58 ℃ - 59 ℃.

HRMS (ESI, m/z): calculated for $C_{28}H_{24}O_6SH^+$: 489.1372 (M+H)$^+$, found: 489.1372.
[α]^{21}$_D$ = -8.5 (c = 3.7 in $CHCl_3$).
HPLC analysis: >99:1 *e.r.* (CHIRALPAK AD-H column, ^{i}PrOH/Hexane = 24:76, flow rate = 0.5 mL/min, λ = 254 nm, t_{major} = 22.5 min, t_{minor}= 30.3 min).

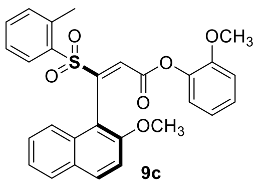

9c

Light yellow oil, 46% yield, *E*/*Z* >20:1.
^{1}H NMR (400 MHz, $CDCl_3$) δ 7.82 – 7.74 (m, 3H), 7.71 (d, *J* = 8.2 Hz, 1H), 7.52 (d, *J* = 7.9 Hz, 1H), 7.48 – 7.38 (m, 2H), 7.36 – 7.28 (m, 1H), 7.29 – 7.23 (m, 2H), 7.13 – 7.01 (m, 2H), 6.93 (d, *J* = 9.1 Hz, 1H), 6.81 – 6.72 (m, 2H), 6.67 (dd, *J* = 7.9, 1.7 Hz, 1H), 3.50 (s, 3H), 3.30 (s, 3H), 2.56 (s, 3H) ppm.
^{13}C NMR (101 MHz, $CDCl_3$) δ 161.4, 154.5, 152.8, 150.9, 140.6, 139.2, 135.9, 133.9, 133.2, 132.2, 132.0, 131.9, 130.3, 128.7, 127.8, 127.2, 127.1, 125.9, 125.1, 124.0, 122.4, 120.6, 112.5, 112.1, 111.7, 55.7, 55.5, 20.7 ppm.
HRMS (ESI, m/z): calculated for $C_{28}H_{24}O_6SNa^+$: 511.1191 (M+Na)$^+$, found: 511.1195.
[α]^{21}$_D$ = +15.5 (c = 1.0 in $CHCl_3$).
HPLC analysis: 82.5:17.5 *e.r.* (CHIRALCEL AD-H column, ^{i}PrOH/Hexane = 24:76, flow rate = 0.5 mL/min, λ = 254 nm, t_{major} = 19.9 min, t_{minor}= 24.8 min).

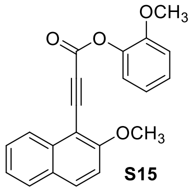

S15

Side product, light yellow oil, 29% yield.
^{1}H NMR (400 MHz, $CDCl_3$) δ 8.14 (d, *J* = 8.3 Hz, 1H), 7.95 (d, *J* = 9.1 Hz, 1H), 7.80 (d, *J* = 8.7 Hz, 1H), 7.60 – 7.53 (m, 1H), 7.44 – 7.38 (m, 1H), 7.30 – 7.23 (m, 2H), 7.21 (dd, *J* = 7.9, 1.6 Hz, 1H), 7.05 – 6.98 (m, 2H), 4.05 (s, 3H), 3.88 (s, 3H) ppm.
^{13}C NMR (101 MHz, $CDCl_3$) δ 162.3, 152.5, 151.5, 139.6, 135.2, 133.7, 128.5, 128.4, 128.3, 127.5, 125.0, 124.8, 123.0, 121.0, 112.8, 112.4, 102.3, 89.5, 84.8, 56.7, 56.1 ppm.
HRMS (ESI, m/z): calculated for $C_{21}H_{16}O_4H^+$: 333.1127 (M+H)$^+$, found: 333.1128.

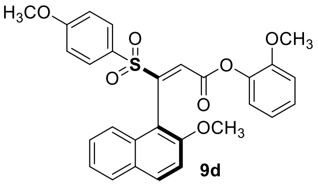

9d

Light yellow oil, 69% yield, *E*/*Z* >20:1.
^{1}H NMR (400 MHz, $CDCl_3$) δ 7.82 (d, *J* = 9.0 Hz, 1H), 7.74 – 7.69 (m, 2H), 7.61 (d, *J* = 8.5 Hz, 1H), 7.50 (d, *J* = 8.8 Hz, 2H), 7.37 (ddd, *J* = 8.4, 6.8, 1.3 Hz, 1H), 7.29 (ddd, *J* = 8.0, 6.9,

1.2 Hz, 1H), 7.05 (t, *J* = 8.8 Hz, 2H), 6.83 – 6.72 (m, 4H), 6.65 (d, *J* = 7.8 Hz, 1H), 3.82 (s, 3H), 3.50 (s, 3H), 3.46 (s, 3H) ppm.
^{13}C NMR (101 MHz, $CDCl_3$) δ 164.1, 161.5, 154.6, 153.9, 150.9, 139.2, 132.9, 132.0, 131.9, 129.4, 129.1, 128.6, 127.9, 127.1, 124.8, 123.9, 122.4, 120.6, 113.9, 112.7, 112.5, 112.1, 55.9, 55.8, 55.7 ppm.
HRMS (ESI, m/z): calculated for $C_{28}H_{24}O_7SNa^+$: 527.1140 $(M+Na)^+$, found: 527.1136.
$[\alpha]^{21}_D$ = -7.2 (c = 1.0 in $CHCl_3$).
HPLC analysis: 95:5 *e.r.* (CHIRALPAK AD-H column, ^{i}PrOH/Hexane = 30:70, flow rate = 0.5 mL/min), λ = 220 nm, t_{major} = 28.3 min, t_{minor}= 36.1 min).

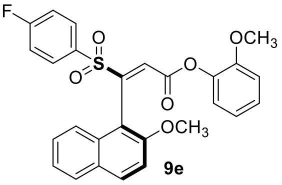

Light yellow oil, 75% yield, *E*/*Z* >20:1.
^{1}H NMR (400 MHz, $CDCl_3$) δ 7.84 (d, *J* = 9.1 Hz, 1H), 7.77 (s, 1H), 7.73 (d, *J* = 8.1 Hz, 1H), 7.66 – 7.55 (m, 3H), 7.41 (ddd, *J* = 8.4, 6.7, 1.4 Hz, 1H), 7.32 (ddd, *J* = 8.1, 6.8, 1.2 Hz, 1H), 7.04 (ddt, *J* = 12.2, 8.5, 4.7 Hz, 4H), 6.80 – 6.72 (m, 2H), 6.65 (dd, *J* = 7.9, 1.7 Hz, 1H), 3.50 (s, 3H), 3.46 (s, 3H) ppm.
^{13}C NMR (101 MHz, $CDCl_3$) δ 166.1 (d, *J* = 256.6 Hz), 161.3, 154.5, 153.2, 150.9, 139.1, 134.0 (d, *J* = 2.9 Hz), 132.8, 132.7, 132.6, 132.2, 130.0, 128.6, 128.0, 127.2 (d, *J* = 11.5 Hz), 124.7, 124.1, 122.3, 120.6, 115.8 (d, J = 22.6 Hz), 112.5, 112.2, 111.9, 55.8, 55.7 ppm.
^{19}F NMR (376 MHz, $CDCl_3$) δ -103.4 ppm.
HRMS (ESI, m/z): calculated for $C_{27}H_{21}FO_6SH^+$: 493.1121 $(M+H)^+$, found: 493.1121.
$[\alpha]^{21}_D$ = -18.7 (c = 3.4 in $CHCl_3$).
HPLC analysis: 98:2 *e.r.* (CHIRALPAK AD-H column, ^{i}PrOH/Hexane = 24:76, flow rate = 0.5 mL/min), λ = 254 nm, t_{major} = 18.7 min, t_{minor}= 22.8 min).

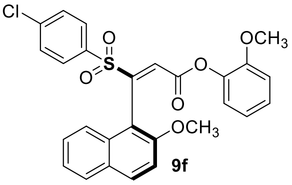

Light yellow oil, 77% yield, *E*/*Z* >20:1.
^{1}H NMR (500 MHz, $CDCl_3$) δ 7.84 (d, *J* = 9.1 Hz, 1H), 7.77 (s, 1H), 7.73 (dd, *J* = 8.2, 1.2 Hz, 1H), 7.65 (dd, *J* = 8.4, 1.1 Hz, 1H), 7.55 – 7.49 (m, 2H), 7.42 (ddd, *J* = 8.3, 6.8, 1.3 Hz, 1H), 7.36 – 7.30 (m, 3H), 7.06 (ddd, *J* = 8.3, 7.5, 1.7 Hz, 1H), 7.02 (d, *J* = 9.1 Hz, 1H), 6.80 – 6.73 (m, 2H), 6.65 (dd, *J* = 7.9, 1.7 Hz, 1H), 3.50 (s, 3H), 3.43 (s, 3H) ppm.
^{13}C NMR (126 MHz, $CDCl_3$) δ 161.2, 154.4, 153.0, 150.8, 140.7, 139.1, 136.5, 132.8, 132.2, 131.2, 130.2, 128.8, 128.6, 128.0, 127.3, 127.2, 124.7, 124.1, 122.3, 120.6, 112.5, 112.0, 111.9, 55.7, 55.6 ppm.
HRMS (ESI, m/z): calculated for $C_{27}H_{21}ClO_6SH+$: 509.0826 (M+H)+, found: 509.0826.

[α]^{21}D = +1.2 (c = 3.9 in $CHCl_3$).
HPLC analysis: >99:1 *e.r.* (CHIRALPAK AD-H column, ^{i}PrOH/Hexane = 24:76, flow rate = 0.5 mL/min, λ = 254 nm, t_{major} = 22.0 min, t_{minor}= 28.3 min).

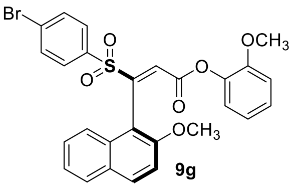

9g

Light yellow solid, 85% yield, *E*/*Z* >20:1.
^{1}H NMR (400 MHz, $CDCl_3$) δ 7.84 (d, *J* = 9.0 Hz, 1H), 7.77 (s, 1H), 7.75 – 7.71 (m, 1H), 7.68 – 7.63 (m, 1H), 7.54 – 7.48 (m, 2H), 7.47 – 7.39 (m, 3H), 7.33 (ddd, *J* = 8.0, 6.9, 1.2 Hz, 1H), 7.09 – 6.99 (m, 2H), 6.81 – 6.73 (m, 2H), 6.65 (dd, *J* = 7.9, 1.7 Hz, 1H), 3.50 (s, 3H), 3.42 (s, 3H) ppm.
^{13}C NMR (101 MHz, $CDCl_3$) δ 161.2, 154.4, 152.9, 150.8, 139.1, 137.0, 132.8, 132.2, 131.8, 131.2, 130.3, 129.3, 128.6, 128.0, 127.3, 127.2, 124.7, 124.1, 122.3, 120.6, 112.5, 112.0, 111.8, 55.7, 55.6 ppm.
M.P. 166 °C - 167 °C
HRMS (ESI, m/z): calculated for $C_{27}H_{21}BrO_6SH^+$: 553.0320 $(M+H)^+$, found: 553.0318.
[α]^{21}D = +9.6 (c = 4.2 in $CHCl_3$).
HPLC analysis: >99:1 *e.r.* (CHIRALPAK AD-H column, ^{i}PrOH/Hexane = 20:80, flow rate = 0.5 mL/min), λ = 254 nm, t_{major} = 27.8 min, t_{minor}= 39.1 min).

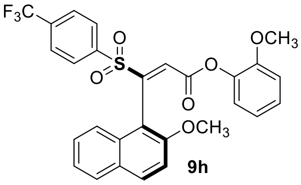

9h

Light yellow oil, 98% yield, *E*/*Z* >20:1.
^{1}H NMR (400 MHz, $CDCl_3$) δ 7.85 (d, *J* = 9.1 Hz, 1H), 7.82 (s, 1H), 7.78 – 7.70 (m, 3H), 7.68 – 7.59 (m, 3H), 7.41 (ddd, *J* = 8.4, 6.8, 1.4 Hz, 1H), 7.33 (ddd, *J* = 8.1, 6.8, 1.2 Hz, 1H), 7.06 (td, *J* = 7.9, 1.7 Hz, 1H), 7.00 (d, *J* = 9.1 Hz, 1H), 6.82 – 6.72 (m, 2H), 6.65 (dd, *J* = 7.9, 1.7 Hz, 1H), 3.50 (s, 3H), 3.36 (s, 3H) ppm.
^{13}C NMR (126 MHz, $CDCl_3$) δ 161.1, 154.4, 152.5, 150.8, 141.6, 139.0, 135.4 (q, *J* = 33.1 Hz), 132.8, 132.4, 131.0, 130.3, 128.6, 128.0, 127.4, 127.2, 125.5 (q, *J* = 3.7 Hz), 124.5, 124.1, 122.2, 120.6, 112.5, 111.8, 111.7, 55.6, 55.5 ppm.
^{19}F NMR (376 MHz, $CDCl_3$) δ -63.2 ppm.
HRMS (ESI, m/z): calculated for $C_{28}H_{21}F_3O_6SH^+$: 543.1089 $(M+H)^+$, found: 543.1089.
[α]^{21}D = -15.2 (c = 3.5 in $CHCl_3$).
HPLC analysis: 87.5:12.5 *e.r.* (CHIRALPAK AD-H column, ^{i}PrOH/Hexane = 20:80, flow rate = 0.5 mL/min, λ = 254 nm, t_{major} = 21.8 min, t_{minor}= 29.4 min).

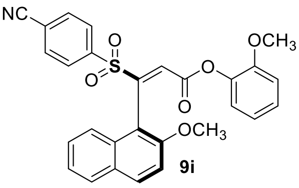

9i

Light yellow oil, 96% yield, *E*/*Z* >20:1.

^{1}H NMR (400 MHz, $CDCl_3$) δ 7.85 (d, *J* = 9.1 Hz, 1H), 7.81 (s, 1H), 7.74 (d, *J* = 8.1 Hz, 1H), 7.72 – 7.67 (m, 2H), 7.62 (dd, *J* = 8.5, 1.7 Hz, 3H), 7.42 (ddd, *J* = 8.4, 6.9, 1.4 Hz, 1H), 7.34 (ddd, *J* = 8.1, 6.8, 1.2 Hz, 1H), 7.10 – 7.03 (m, 1H), 7.01 (d, *J* = 9.1 Hz, 1H), 6.81 – 6.73 (m, 2H), 6.64 (dd, *J* = 7.9, 1.7 Hz, 1H), 3.49 (s, 3H), 3.41 (s, 3H) ppm.

^{13}C NMR (101 MHz, $CDCl_3$) δ 160.9, 154.4, 152.1, 150.7, 142.3, 138.9, 132.7, 132.6, 132.1, 131.3, 130.3, 128.6, 128.1, 127.4, 127.2, 124.4, 124.2, 122.2, 120.6, 117.3, 117.3, 112.5, 111.9, 111.6, 55.7, 55.6 ppm.

HRMS (ESI, m/z): calculated for $C_{28}H_{21}NO_6SH^+$: 500.1168 (M+H)+, found: 500.1169.

$[\alpha]^{21}_D$ = +6.6 (c = 4.3 in $CHCl_3$).

HPLC analysis: 93:7 *e.r.* (CHIRALPAK AD-H column, ^{i}PrOH/Hexane = 30:70, flow rate = 0.5 mL/min, λ = 254 nm, t_{major} = 35.8 min, t_{minor}= 39.8 min).

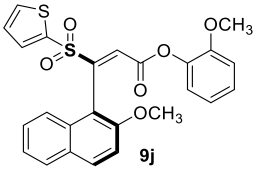

9j

Light yellow oil, 99% yield, *E*/*Z* >20:1.

^{1}H NMR (400 MHz, $CDCl_3$) δ 7.86 (d, *J* = 9.0 Hz, 1H), 7.78 (s, 1H), 7.75 – 7.71 (m, 1H), 7.63 (dd, *J* = 5.0, 1.4 Hz, 1H), 7.60 – 7.56 (m, 1H), 7.37 (ddd, *J* = 8.4, 6.8, 1.4 Hz, 1H), 7.33 – 7.27 (m, 2H), 7.12 (d, *J* = 9.0 Hz, 1H), 7.06 (ddd, *J* = 8.3, 7.5, 1.7 Hz, 1H), 6.97 (dd, *J* = 5.0, 3.8 Hz, 1H), 6.80 – 6.73 (m, 2H), 6.65 (dd, *J* = 7.8, 1.7 Hz, 1H), 3.58 (s, 3H), 3.50 (s, 3H) ppm.

^{13}C NMR (101 MHz, $CDCl_3$) δ 161.3, 155.2, 153.5, 150.8, 139.1, 138.7, 136.1, 135.4, 133.1, 132.1, 129.6, 128.6, 128.0, 127.7, 127.2, 127.1, 124.3, 123.9, 122.3, 120.6, 112.5, 112.2, 56.1, 55.6 ppm.

HRMS (ESI, m/z): calculated for $C_{25}H_{20}O_6S_2H+$: 481.0780 (M+H)+, found: 481.0779.

$[\alpha]^{21}_D$ = -34.1 (c = 4.9 in $CHCl_3$).

HPLC analysis: 98.5:1.5 *e.r.* (CHIRALPAK AD-H column, ^{i}PrOH/Hexane = 24:76, flow rate = 0.5 mL/min, λ = 220 nm, t_{major} = 24.8 min, t_{minor}= 29.3 min).

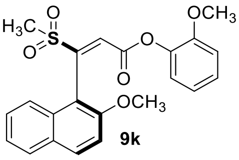

9k

Light yellow oil, 90% yield, *E*/*Z* 5:1.

^{1}H NMR (500 MHz, $CDCl_3$) δ 7.93 (d, *J* = 9.0 Hz, 1H), 7.83 – 7.80 (m, 1H), 7.80 – 7.76 (m, 1H), 7.67 (s, 1H), 7.52 (ddd, *J* = 8.4, 6.8, 1.3 Hz, 1H), 7.38 (ddd, *J* = 8.1, 6.8, 1.2 Hz, 1H), 7.30

(d, J = 9.1 Hz, 1H), 7.08 (ddd, J = 8.2, 7.5, 1.7 Hz, 1H), 6.83 – 6.74 (m, 2H), 6.65 (dd, J = 7.9, 1.6 Hz, 1H), 3.97 (s, 3H), 3.51 (s, 3H), 2.95 (s, 3H) ppm.
^{13}C NMR (126 MHz, $CDCl_3$) δ 161.2, 154.5, 152.2, 150.8, 139.0, 133.0, 132.4, 130.6, 129.1, 128.1, 127.6, 127.2, 124.8, 124.4, 122.3, 120.6, 113.0, 112.8, 112.5, 56.8, 55.6, 40.6 ppm.
HRMS (ESI, m/z): calculated for $C_{22}H_{20}O_6SH^+$: 413.1059 $(M+H)^+$, found: 413.1058.
$[\alpha]^{21}_D$ = -54.3 (c = 3.7 in $CHCl_3$).
HPLC analysis for the *E* isomer: 93:7 *e.r.* (CHIRALPAK AD-H column, ^{i}PrOH/Hexane = 25:75, flow rate = 0.5 mL/min, λ = 254 nm, t_{major} = 25.8 min, t_{minor}= 37.6 min).

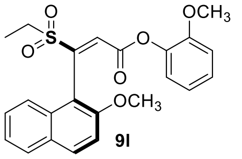

9l

Light yellow oil, 83% yield, *E*/*Z* 20:1.
^{1}H NMR (500 MHz, $CDCl_3$) δ 7.92 (d, J = 9.0 Hz, 1H), 7.83 (dd, J = 8.6, 1.0 Hz, 1H), 7.78 – 7.75 (m, 1H), 7.63 (s, 1H), 7.51 (ddd, J = 8.4, 6.8, 1.3 Hz, 1H), 7.37 (ddd, J = 8.1, 6.8, 1.1 Hz, 1H), 7.28 (d, J = 9.1 Hz, 1H), 7.07 (ddd, J = 8.3, 7.5, 1.7 Hz, 1H), 6.81 – 6.75 (m, 2H), 6.65 (dd, J = 7.9, 1.7 Hz, 1H), 3.96 (s, 3H), 3.50 (s, 3H), 3.18 – 3.09 (m, 1H), 3.07 – 2.98 (m, 1H), 1.40 (t, J = 7.4 Hz, 3H) ppm.
^{13}C NMR (126 MHz, $CDCl_3$) δ 161.2, 154.6, 151.1, 150.8, 139.1, 133.2, 132.3, 131.9, 129.2, 128.1, 127.5, 127.2, 125.0, 124.4, 122.3, 120.7, 113.1, 112.7, 112.5, 56.7, 55.6, 47.1, 6.4 ppm.
HRMS (ESI, m/z): calculated for $C_{23}H_{22}O_6SH^+$: 427.1215 (M+H)+, found: 427.1211.
$[\alpha]^{21}_D$ = -38.2 (c = 2.0 in $CHCl_3$).
HPLC analysis: 93:7 *e.r.* (CHIRALPAK AD-H column, ^{i}PrOH/Hexane = 24:76, flow rate = 0.5 mL/min, λ = 220 nm, t_{major} = 23.6 min, t_{minor}= 29.2 min).

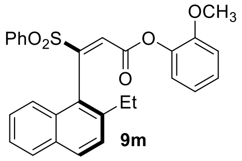

9m

Light yellow oil, 91% yield, *E*/*Z* 7:1.
^{1}H NMR (400 MHz, $CDCl_3$) δ 7.81 – 7.74 (m, 2H), 7.67 (d, J = 8.2 Hz, 1H), 7.51 – 7.43 (m, 3H), 7.35 (d, J = 8.5 Hz, 1H), 7.25 (m, 4H), 7.11 (m, 1H), 7.06 – 7.00 (m, 1H), 6.78 – 6.68 (m, 2H), 6.56 (dd, J = 7.9, 1.6 Hz, 1H), 3.45 (s, 3H), 2.65 (dq, J = 15.0, 7.6 Hz, 1H), 2.47 (dq, J = 15.0, 7.6 Hz, 1H), 1.14 (t, J = 7.5 Hz, 3H) ppm.
^{13}C NMR (101 MHz, $CDCl_3$) δ 161.3, 155.5, 150.8, 142.2, 138.9, 136.7, 134.2, 131.7, 131.5, 130.1, 129.8, 129.7, 129.0, 127.7, 127.2, 126.3, 126.1, 125.0, 125.0, 124.3, 122.2, 120.6, 112.4, 55.5, 27.2, 14.6 ppm.
HRMS (ESI, m/z): calculated for $C_{28}H_{24}O_5SH^+$: 473.1423 $(M+H)^+$, found: 473.1414.
$[\alpha]^{21}_D$ = -9.3 (c = 2.0 in $CHCl_3$).
HPLC analysis: 65:35 *e.r.* (CHIRALPAK AD-H column, ^{i}PrOH/Hexane = 5:95, flow rate = 0.5 mL/min, λ = 254 nm, t_{major} = 36.5 min, t_{minor}= 40.2 min).

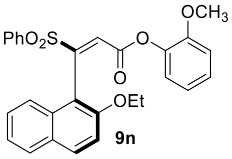

9n

Light yellow oil, 78% yield, *E*/*Z* >20:1.

^{1}H NMR (500 MHz, $CDCl_3$) δ 7.80 (d, *J* = 9.1 Hz, 1H), 7.75 (s, 1H), 7.73 – 7.69 (m, 1H), 7.65 (dd, *J* = 8.5, 1.1 Hz, 1H), 7.62 – 7.58 (m, 2H), 7.56 – 7.51 (m, 1H), 7.40 – 7.32 (m, 3H), 7.30 (ddd, *J* = 8.1, 6.8, 1.2 Hz, 1H), 7.06 (ddd, *J* = 8.2, 7.5, 1.7 Hz, 1H), 6.99 (d, *J* = 9.0 Hz, 1H), 6.80 – 6.73 (m, 2H), 6.64 (dd, *J* = 7.9, 1.7 Hz, 1H), 3.90 – 3.83 (m, 1H), 3.57 – 3.49 (m, 1H), 3.52 (s, 3H), 1.12 (t, *J* = 7.0 Hz, 3H) ppm.

^{13}C NMR (126 MHz, $CDCl_3$) δ 161.4, 154.2, 153.7, 150.9, 139.1, 138.2, 133.79, 132.9, 131.9, 129.9, 129.7, 128.6, 128.5, 127.8, 127.1, 127.0, 124.9, 123.9, 122.3, 120.6, 112.9, 112.5, 112.4, 64.2, 55.6, 14.9 ppm.

HRMS (ESI, m/z): calculated for $C_{28}H_{24}O_6SH^+$: 489.1372 $(M+H)^+$, found: 489.1373.

$[\alpha]^{21}_{D}$ = -24.1 (c = 2.8 in $CHCl_3$).

HPLC analysis: 98.5:1.5 *e.r.* (CHIRALPAK AD-H column, ^{i}PrOH/Hexane = 24:76, flow rate = 0.5 mL/min, λ = 254 nm, t_{major} = 19.5 min, t_{minor}= 24.7 min).

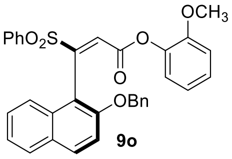

9o

Light yellow oil, 82% yield, *E*/*Z* >20:1.

^{1}H NMR (500 MHz, $CDCl_3$) δ 7.83 – 7.78 (m, 2H), 7.74 (ddd, *J* = 7.6, 3.9, 1.1 Hz, 2H), 7.55 – 7.51 (m, 2H), 7.48 – 7.41 (m, 2H), 7.36 – 7.30 (m, 4H), 7.25 – 7.21 (m, 2H), 7.21 – 7.15 (m, 2H), 7.07 (ddd, *J* = 8.4, 7.5, 1.7 Hz, 1H), 7.02 (d, *J* = 9.1 Hz, 1H), 6.81 – 6.73 (m, 2H), 6.61 (dd, *J* = 8.0, 1.6 Hz, 1H), 4.85 (d, *J* = 12.2 Hz, 1H), 4.58 (d, *J* = 12.1 Hz, 1H), 3.46 (s, 3H) ppm.

^{13}C NMR (126 MHz, $CDCl_3$) δ 161.4, 153.9, 153.6, 150.9, 139.0, 137.8, 136.9, 133.7, 133.0, 132.0, 129.9, 129.6, 128.7, 128.5, 128.5, 127.9, 127.8, 127.2, 127.1, 126.7, 125.0, 124.1, 122.3, 120.6, 113.2, 112.9, 112.4, 70.4, 55.6 ppm.

HRMS (ESI, m/z): calculated for $C_{33}H_{26}O_6SH^+$: 551.1528 $(M+H)^+$, found: 551.1528.

$[\alpha]^{21}_{D}$ = -13.4 (c = 4.5 in $CHCl_3$).

HPLC analysis: 93:7 *e.r.* (CHIRALPAK AD-H column, ^{i}PrOH/Hexane = 24:76, flow rate = 0.5 mL/min, λ = 220 nm, t_{major} = 22.5 min, t_{minor}= 26.8 min).

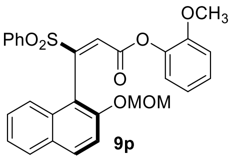

9p

Light yellow oil, 91% yield, *E*/*Z* >20:1.

^{1}H NMR (500 MHz, $CDCl_3$) δ 7.81 (d, *J* = 9.1 Hz, 1H), 7.78 (s, 1H), 7.72 (dd, *J* = 8.1, 1.4 Hz, 1H), 7.63 – 7.57 (m, 2H), 7.59 – 7.51 (m, 2H), 7.39 – 7.27 (m, 5H), 7.05 (dd, *J* = 1.6, 0.7 Hz, 1H), 6.76 (ddd, *J* = 9.1, 8.0, 1.4 Hz, 2H), 6.58 (dd, *J* = 8.0, 1.6 Hz, 1H), 4.78 (d, *J* = 7.0 Hz, 1H), 4.66 (d, *J* = 7.1 Hz, 1H), 3.47 (s, 3H), 3.31 (s, 3H) ppm.

^{13}C NMR (126 MHz, $CDCl_3$) δ 161.4, 153.2, 153.0, 150.8, 139.0, 138.0, 133.9, 132.7, 132.0, 130.1, 129.7, 129.1, 128.7, 127.9, 127.2, 127.0, 124.9, 124.3, 122.2, 120.6, 115.2, 113.3, 112.5, 94.9, 56.0, 55.5 ppm.
HRMS (ESI, m/z): calculated for $C_{28}H_{24}O_7SH^+$: 505.1321 $(M+H)^+$, found: 505.1320.
$[\alpha]^{21}_D$ = -7.3 (c = 4.6 in $CHCl_3$).
HPLC analysis: 93:7 *e.r.* (CHIRALPAK AD-H column, ^{i}PrOH/Hexane = 24:76, flow rate = 0.5 mL/min, λ = 254 nm, t_{major} = 17.8 min, t_{minor}= 20.2 min).

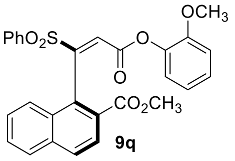

9q

Light yellow oil, 84% yield, *E*/*Z* >20:1.
^{1}H NMR (400 MHz, $CDCl_3$) δ 8.00 (dd, *J* = 8.7, 1.3 Hz, 1H), 7.88 (d, *J* = 8.7 Hz, 1H), 7.78 (d, *J* = 8.2 Hz, 1H), 7.71 (s, 1H), 7.55 – 7.41 (m, 5H), 7.31 – 7.24 (m, 3H), 7.07 – 6.99 (m, 1H), 6.78 – 6.71 (m, 2H), 6.69 – 6.63 (m, 1H), 3.78 (s, 3H), 3.50 (s, 3H) ppm.
^{13}C NMR (101 MHz, $CDCl_3$) δ 166.4, 161.3, 155.6, 150.7, 139.0, 136.9, 134.7, 134.0, 131.2, 129.9, 129.7, 129.6, 128.9, 128.2, 127.9, 127.8, 127.0, 126.9, 126.2, 125.7, 122.2, 120.5, 112.4, 55.5, 52.3 ppm.
HRMS (ESI, m/z): calculated for $C_{28}H_{22}O_7SH^+$: 503.1164 $(M+H)^+$, found: 503.1166.
$[\alpha]^{21}_D$ = -52.1 (c = 2.0 in $CHCl_3$).
HPLC analysis: 78.5:21.5 *e.r.* (CHIRALPAK AD-H column, ^{i}PrOH/Hexane = 24:76, flow rate = 0.5 mL/min, λ = 220 nm, t_{major} = 30.4 min, t_{minor}= 37.1 min).

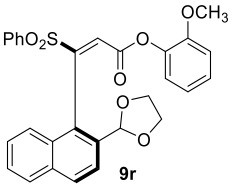

9r

Light yellow oil, 75% yield, *E*/*Z* >20:1.
^{1}H NMR (400 MHz, $CDCl_3$) δ 7.86 (d, *J* = 8.6 Hz, 1H), 7.81 (s, 1H), 7.73 – 7.70 (m, 1H), 7.68 (d, *J* = 8.6 Hz, 1H), 7.56 – 7.50 (m, 2H), 7.50 – 7.44 (m, 1H), 7.35 – 7.23 (m, 4H), 7.13 (ddd, *J* = 8.4, 6.8, 1.3 Hz, 1H), 7.03 (ddd, *J* = 8.3, 7.4, 1.7 Hz, 1H), 6.74 (ddd, *J* = 14.6, 7.9, 1.4 Hz, 2H), 6.65 (dd, *J* = 7.9, 1.7 Hz, 1H), 5.84 (s, 1H), 4.19 – 4.11 (m, 1H), 4.10 – 4.01 (m, 1H), 3.99 – 3.91 (m, 2H), 3.51 (s, 3H) ppm.
^{13}C NMR (101 MHz, $CDCl_3$) δ 161.0, 154.0, 150.8, 139.1, 136.7, 135.6, 134.3, 133.3, 131.2, 130.6, 130.4, 129.8, 129.1, 127.9, 127.1, 126.3, 126.3, 125.5, 123.6, 122.4, 120.6, 112.5, 102.0, 65.8, 65.4, 55.7 ppm.
HRMS (ESI, m/z): calculated for $C_{29}H_{24}O_7SH^+$: 517.1321 $(M+H)^+$, found: 517.1318.
$[\alpha]^{21}_D$ = -18.0 (c = 3.9 in $CHCl_3$).
HPLC analysis: 78:22 *e.r.* (CHIRALPAK AD-H column, ^{i}PrOH/Hexane = 28:72, flow rate = 0.5 mL/min, λ = 220 nm, t_{minor}= 27.9 min, t_{major} = 34.2 min).

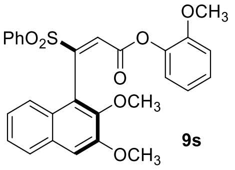

9s

Light yellow oil, 88% yield, *E*/*Z* >20:1.

^{1}H NMR (500 MHz, $CDCl_3$) δ 7.72 (s, 1H), 7.69 – 7.64 (m, 2H), 7.61 (dd, *J* = 8.5, 1.2 Hz, 1H), 7.57 – 7.52 (m, 1H), 7.38 – 7.32 (m, 2H), 7.30 – 7.26 (m, 2H), 7.17 (s, 1H), 7.12 (ddd, *J* = 8.1, 6.9, 1.3 Hz, 1H), 7.06 (ddd, *J* = 8.3, 7.5, 1.7 Hz, 1H), 6.80 – 6.73 (m, 2H), 6.64 (dd, *J* = 7.9, 1.6 Hz, 1H), 3.90 (s, 3H), 3.76 (s, 3H), 3.50 (s, 3H) ppm.

^{13}C NMR (126 MHz, $CDCl_3$) δ 161.3, 154.4, 151.6, 150.8, 147.6, 139.0, 137.8, 134.1, 130.7, 129.6, 129.4, 128.9, 127.4, 127.2, 126.6, 125.2, 124.4, 122.3, 120.6, 119.2, 112.4, 109.7, 60.8, 55.7, 55.5 ppm.

HRMS (ESI, m/z): calculated for $C_{28}H_{24}O_7SH^+$: 505.1321 $(M+H)^+$, found: 505.1315.

$[\alpha]^{21}_D$ = -41.0 (c = 1.3 in $CHCl_3$).

HPLC analysis: 97:3 *e.r.* (CHIRALPAK AD-H column, ^{i}PrOH/Hexane = 24:76, flow rate = 0.5 mL/min, λ = 254 nm, t_{major}= 21.0 min, t_{minor} = 23.5 min).

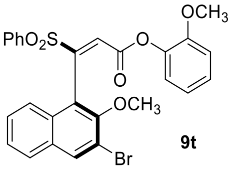

9t

Light yellow oil, 87% yield, *E*/*Z* >20:1.

^{1}H NMR (400 MHz, $CDCl_3$) δ 8.07 (s, 1H), 7.76 (s, 1H), 7.66 – 7.56 (m, 4H), 7.47 – 7.42 (m, 1H), 7.39 – 7.30 (m, 4H), 7.09 (ddd, *J* = 8.7, 7.5, 1.7 Hz, 1H), 6.82 – 6.75 (m, 2H), 6.68 (dd, *J* = 7.8, 1.7 Hz, 1H), 3.72 (s, 3H), 3.52 (s, 3H) ppm.

^{13}C NMR (101 MHz, $CDCl_3$) δ 161.3, 154.6, 152.4, 150.7, 138.8, 137.2, 134.8, 134.4, 131.7, 131.2, 129.7, 129.2, 129.0, 127.4, 127.2, 127.1, 126.0, 124.9, 122.2, 121.2, 120.7, 116.1, 112.4, 61.4, 55.6 ppm.

HRMS (ESI, m/z): calculated for $C_{27}H_{21}BrO_6SH^+$: 553.0320 $(M+H)^+$, found: 553.0324.

$[\alpha]^{21}_D$ = -23.1 (c = 1.0 in $CHCl_3$).

HPLC analysis: 92:8 *e.r.* (CHIRALPAK AD-H column, ^{i}PrOH/Hexane = 24:76, flow rate = 0.5 mL/min, λ = 254 nm, t_{major} = 21.4 min, t_{minor} = 24.6 min).

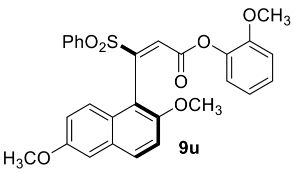

9u

Light yellow oil, 94% yield, *E*/*Z* >20:1.

^{1}H NMR (400 MHz, $CDCl_3$) δ 7.76 (s, 1H), 7.71 (d, *J* = 9.0 Hz, 1H), 7.63 – 7.52 (m, 4H), 7.39 – 7.34 (m, 2H), 7.11 – 7.02 (m, 3H), 6.97 (d, *J* = 9.1 Hz, 1H), 6.81 – 6.73 (m, 2H), 6.66 (dd, *J* = 7.8, 1.7 Hz, 1H), 3.87 (s, 3H), 3.54 (s, 3H), 3.33 (s, 3H) ppm.

^{13}**C NMR** (101 MHz, $CDCl_3$) δ 161.3, 156.2, 153.5, 153.0, 150.9, 139.1, 138.0, 133.8, 130.5, 129.8, 129.7, 129.6, 128.5, 128.2, 127.1, 126.3, 122.3, 120.6, 120.0, 112.6, 112.5, 112.5, 106.0, 55.7, 55.7, 55.4 ppm.
HRMS (ESI, m/z): calculated for $C_{28}H_{24}O_7SH^+$: 505.1321 $(M+H)^+$, found: 505.1321.
$[\alpha]^{21}_D$ = -22.5 (c = 4.7 in $CHCl_3$).
HPLC analysis: >99:1 *e.r.* (CHIRALPAK AD-H column, ^{i}PrOH/Hexane = 20:80, flow rate = 0.5 mL/min, λ = 220 nm, t_{major} = 36.5 min, t_{minor}= 38.6 min).

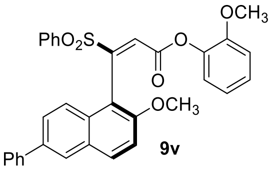

9v

Light yellow oil, 95% yield, *E*/*Z* >20:1.
^{1}**H NMR** (400 MHz, $CDCl_3$) δ 7.93 (d, *J* = 1.8 Hz, 1H), 7.89 (d, *J* = 9.1 Hz, 1H), 7.82 (s, 1H), 7.76 (d, *J* = 8.8 Hz, 1H), 7.72 – 7.62 (m, 5H), 7.59 – 7.53 (m, 1H), 7.48 (dd, *J* = 8.5, 6.9 Hz, 2H), 7.43 – 7.34 (m, 3H), 7.09 – 7.01 (m, 2H), 6.77 (dd, *J* = 8.5, 7.0 Hz, 2H), 6.70 (dd, *J* = 7.8, 1.7 Hz, 1H), 3.52 (s, 3H), 3.37 (s, 3H) ppm.
^{13}**C NMR** (101 MHz, $CDCl_3$) δ 161.4, 154.5, 153.4, 150.8, 141.0, 139.1, 138.0, 136.6, 133.8, 132.3, 132.1, 130.0, 129.7, 128.9, 128.9, 128.6, 127.3, 127.1, 126.9, 125.7, 125.6, 122.3, 120.6, 112.5, 112.3, 112.3, 55.7, 55.7 ppm.
HRMS (ESI, m/z): calculated for $C_{33}H_{26}O_6SNa^+$: 573.1348 $(M+Na)^+$, found: 573.1351.
$[\alpha]^{21}_D$ = -33.2 (c = 2.0 in $CHCl_3$).
HPLC analysis: 88:12 *e.r.* (CHIRALPAK AD-H column, ^{i}PrOH/Hexane = 24:76, flow rate = 0.5 mL/min, λ = 220 nm, t_{major} = 39.0 min, t_{minor}= 47.4 min).

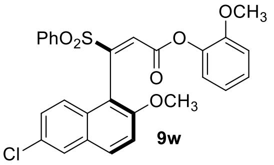

9w

Light yellow solid, 74% yield, *E*/*Z* >20:1.
^{1}**H NMR** (400 MHz, $CDCl_3$) δ 7.77 (s, 1H), 7.73 (d, *J* = 9.1 Hz, 1H), 7.71 (d, *J* = 2.2 Hz, 1H), 7.63 – 7.55 (m, 4H), 7.41 – 7.36 (m, 2H), 7.34 (dd, *J* = 9.1, 2.2 Hz, 1H), 7.07 (ddd, *J* = 8.3, 7.5, 1.7 Hz, 1H), 7.02 (d, *J* = 9.1 Hz, 1H), 6.82 – 6.75 (m, 2H), 6.69 (dd, *J* = 7.8, 1.7 Hz, 1H), 3.54 (s, 3H), 3.35 (s, 3H) ppm.
^{13}**C NMR** (101 MHz, $CDCl_3$) δ 161.3, 154.6, 153.0, 150.8, 139.0, 137.9, 134.0, 131.2, 131.1, 130.2, 129.7, 129.1, 128.7, 128.0, 127.2, 126.6, 126.5, 122.3, 120.7, 113.0, 112.7, 112.5, 55.8, 55.7 ppm.
M.P. 165 °C - 166 °C
HRMS (ESI, m/z): calculated for $C_{27}H_{21}ClO_6SH^+$: 509.0826 $(M+H)^+$, found: 509.0827.
$[\alpha]^{21}_D$ = -5.2 (c = 0.1 in $CHCl_3$).
HPLC analysis: >99:1 *e.r.* (CHIRALPAK AD-H column, ^{i}PrOH/Hexane = 24:76, flow rate = 0.5 mL/min, λ = 254 nm, t_{major} = 21.5 min, t_{minor}= 25.2 min).

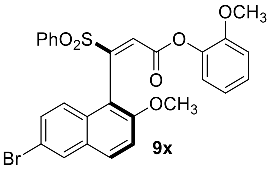

Light yellow solid, 78% yield, *E*/*Z* >20:1.

^{1}H NMR (400 MHz, $CDCl_3$) δ 7.88 (d, *J* = 2.0 Hz, 1H), 7.77 (s, 1H), 7.74 – 7.71 (m, 1H), 7.61 – 7.54 (m, 4H), 7.46 (dd, *J* = 9.1, 2.1 Hz, 1H), 7.41 – 7.36 (m, 2H), 7.07 (ddd, *J* = 8.3, 7.4, 1.7 Hz, 1H), 7.01 (d, *J* = 9.1 Hz, 1H), 6.81 – 6.75 (m, 2H), 6.69 (dd, *J* = 7.8, 1.7 Hz, 1H), 3.54 (s, 3H), 3.34 (s, 3H) ppm.

^{13}C NMR (101 MHz, $CDCl_3$) δ 161.2, 154.6, 152.9, 150.8, 139.0, 137.8, 134.0, 131.4, 131.1, 130.4, 130.2, 129.8, 129.7, 129.6, 128.6, 127.2, 126.7, 122.3, 120.6, 117.7, 112.9, 112.7, 112.5, 55.7, 55.7 ppm.

M.P. 170 °C - 171 °C

HRMS (ESI, m/z): calculated for $C_{27}H_{21}BrO_6SH^+$: 553.0320 $(M+H)^+$, found: 553.0314.

[α]^{21}$_D$ = -15.2 (c = 0.7 in $CHCl_3$).

HPLC analysis: >99:1 *e.r.* (CHIRALPAK AD-H column, ^{i}PrOH/Hexane = 24:76, flow rate = 0.5 mL/min, λ = 254 nm, t_{major} = 28.2 min, t_{minor}= 33.1 min).

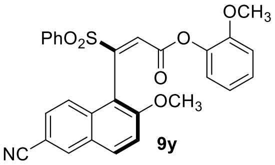

Light yellow solid, 58% yield, *E*/*Z* >20:1

^{1}H NMR (400 MHz, $CDCl_3$) δ 8.12 (d, *J* = 1.8 Hz, 1H), 7.89 (d, *J* = 9.1 Hz, 1H), 7.78 (s, 1H), 7.71 (d, *J* = 8.8 Hz, 1H), 7.63 – 7.57 (m, 3H), 7.50 (dd, *J* = 8.8, 1.8 Hz, 1H), 7.40 (dd, *J* = 8.8, 6.9 Hz, 2H), 7.16 – 7.05 (m, 2H), 6.84 – 6.76 (m, 2H), 6.72 (dd, *J* = 7.8, 1.8 Hz, 1H), 3.54 (s, 3H), 3.41 (s, 3H) ppm.

^{13}C NMR (101 MHz, $CDCl_3$) δ 161.2, 156.8, 152.5, 150.7, 139.0, 137.8, 134.5, 134.3, 134.2, 132.7, 130.5, 129.7, 128.8, 127.5, 127.4, 126.2, 122.3, 120.7, 119.4, 113.6, 113.1, 112.6, 107.4, 55.9, 55.7 ppm.

M.P. 195 °C - 197 °C

HRMS (ESI, m/z): calculated for $C_{28}H_{22}NO_6SH^+$: 500.1168 $(M+H)^+$, found: 500.1166.

[α]^{21}$_D$ = -5.3 (c = 1.4 in $CHCl_3$).

HPLC analysis: 80:20 *e.r.* (CHIRALCEL AS-H column, ^{i}PrOH/Hexane = 25:75, flow rate = 0.5 mL/min, λ = 220 nm, t_{minor} = 52.3 min, t_{major}= 66.2 min).

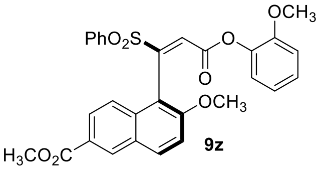

Light yellow oil, 68% yield, *E*/*Z* >20:1.

^{1}H NMR (400 MHz, $CDCl_3$) δ 8.49 (s, 1H), 7.94 (dd, *J* = 9.0, 2.1 Hz, 2H), 7.78 (s, 1H), 7.64 – 7.54 (m, 4H), 7.38 (t, *J* = 7.7 Hz, 2H), 7.11 – 7.03 (m, 2H), 6.81 – 6.72 (m, 2H), 6.68 (dd, *J* = 7.9, 1.6 Hz, 1H), 3.95 (s, 3H), 3.51 (s, 3H), 3.42 (s, 3H) ppm.
^{13}C NMR (101 MHz, $CDCl_3$) δ 167.2, 161.3, 156.4, 153.0, 150.8, 139.1, 137.9, 135.2, 134.0, 133.6, 131.3, 130.3, 129.7, 128.7, 127.6, 127.2, 126.5, 125.6, 124.9, 122.3, 120.7, 112.7, 112.5, 55.9, 55.7, 52.3 ppm.
HRMS (ESI, m/z): calculated for $C_{29}H_{24}NO_8SH^+$: 533.1270 $(M+H)^+$, found: 533.1271.
$[\alpha]^{21}_D$ = -40.0 (c = 0.3 in $CHCl_3$).
HPLC analysis: 87:13 *e.r.* (CHIRALPAK AD-H column, ^{i}PrOH/Hexane = 28:72, flow rate = 0.5 mL/min, λ = 254 nm, t_{major} = 36.9 min, t_{minor}= 43.1 min).

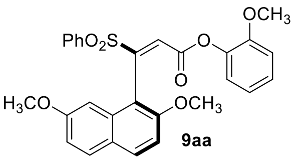

9aa

Light yellow oil, 91% yield, *E*/*Z* >20:1
^{1}H NMR (400 MHz, $CDCl_3$) δ 7.78 (s, 1H), 7.73 (d, *J* = 9.0 Hz, 1H), 7.64 – 7.57 (m, 3H), 7.57 – 7.51 (m, 1H), 7.40 – 7.33 (m, 2H), 7.10 – 7.03 (m, 1H), 6.96 (dd, *J* = 8.8, 2.3 Hz, 1H), 6.92 (d, *J* = 2.4 Hz, 1H), 6.85 – 6.73 (m, 3H), 6.63 (dd, *J* = 8.0, 1.5 Hz, 1H), 3.82 (s, 3H), 3.52 (s, 3H), 3.36 (s, 3H) ppm.
^{13}C NMR (101 MHz, $CDCl_3$) δ 161.4, 158.8, 155.3, 153.7, 150.9, 139.1, 138.2, 134.3, 133.8, 131.7, 130.0, 129.7, 129.4, 128.5, 127.1, 124.2, 122.4, 120.7, 117.1, 112.5, 111.4, 109.2, 103.1, 55.7, 55.4 ppm.
HRMS (ESI, m/z): calculated for $C_{28}H_{24}O_7SH^+$: 505.1321 $(M+H)^+$, found: 505.1320.
$[\alpha]^{21}_D$ = -45.3 (c = 1.0 in $CHCl_3$).
HPLC analysis: 99:1 *e.r.* (CHIRALPAK IC column, ^{i}PrOH/Hexane = 24:76, flow rate = 0.5 mL/min, λ = 220 nm, t_{major} = 20.9 min, t_{minor}= 23.3 min).

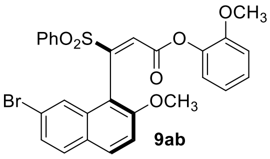

9ab

Light yellow oil, 85% yield, *E*/*Z* >20:1
^{1}H NMR (400 MHz, $CDCl_3$) δ 7.80 – 7.76 (m, 2H), 7.62 – 7.53 (m, 5H), 7.41 – 7.35 (m, 2H), 7.33 (dd, *J* = 8.7, 1.9 Hz, 1H), 7.11 – 7.04 (m, 2H), 6.83 – 6.74 (m, 3H), 3.57 (s, 3H), 3.49 (s, 3H) ppm.
^{13}C NMR (101 MHz, $CDCl_3$) δ 161.2, 155.4, 153.2, 150.8, 139.1, 137.8, 134.2, 133.8, 131.9, 130.2, 129.6, 129.5, 128.7, 127.3, 127.2, 126.9, 126.5, 122.3, 121.8, 120.6, 112.5, 112.5, 111.8, 56.0, 55.7 ppm.
HRMS (ESI, m/z): calculated for $C_{27}H_{21}BrO_6SH^+$: 553.0320 $(M+H)^+$, found: 553.0320.
$[\alpha]^{21}_D$ = +19.5 (c = 4.9 in $CHCl_3$).
HPLC analysis: >99:1 *e.r.* (CHIRALCEL AD-H column, ^{i}PrOH/Hexane = 24:76, flow rate = 0.5 mL/min, λ = 220 nm, t_{major} = 19.7 min, t_{minor}= 21.4 min).

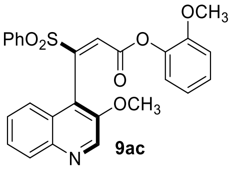

Light yellow oil, 89% yield, *E*/*Z* >20:1.

^{1}H NMR (400 MHz, $CDCl_3$) δ 8.59 (s, 1H), 8.03 – 7.98 (m, 1H), 7.77 (s, 1H), 7.66 – 7.50 (m, 5H), 7.49 – 7.35 (m, 3H), 7.12 – 7.03 (m, 1H), 6.83 – 6.74 (m, 2H), 6.71 (dd, *J* = 8.2, 1.7 Hz, 1H), 3.54 (s, 3H), 3.48 (s, 3H) ppm.

^{13}C NMR (101 MHz, $CDCl_3$) δ 161.0, 151.1, 150.7, 149.2, 143.8, 138.9, 137.9, 137.5, 134.3, 130.4, 129.8, 129.4, 128.9, 127.8, 127.4, 127.4, 127.0, 124.8, 122.2, 121.0, 120.7, 112.5, 56.7, 55.6 ppm.

HRMS (ESI, m/z): calculated for $C_{26}H_{21}NO_6SH^+$: 476.1168 $(M+H)^+$, found: 476.1168.

$[\alpha]^{21}_D$ = -19.7 (c = 1.0 in $CHCl_3$).

HPLC analysis: 98:2 *e.r.* (CHIRALPAK AD-H column, ^{i}PrOH/Hexane = 50:50, flow rate = 0.5 mL/min, λ = 220 nm, t_{minor} = 21.3 min, t_{major} = 35.5 min).

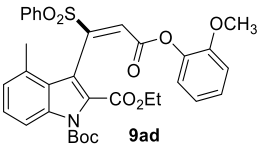

Light yellow oil, 58% yield, *E*/*Z* >20:1.

^{1}H NMR (400 MHz, $CDCl_3$) δ 7.87 (d, *J* = 8.5 Hz, 1H), 7.69 – 7.62 (m, 2H), 7.62 – 7.54 (m, 2H), 7.44 – 7.35 (m, 2H), 7.21 (dd, *J* = 8.5, 7.3 Hz, 1H), 7.12 (ddd, *J* = 8.2, 7.2, 1.9 Hz, 1H), 6.90 – 6.80 (m, 4H), 4.17 – 4.04 (m, 2H), 3.65 (s, 3H), 2.16 (s, 3H), 1.58 (s, 9H), 1.29 (t, *J* = 7.2 Hz, 3H) ppm.

^{13}C NMR (101 MHz, $CDCl_3$) δ 161.4, 160.5, 151.6, 150.9, 149.0, 139.2, 137.3, 136.9, 134.4, 132.9, 130.2, 129.7, 129.3, 129.2, 127.3, 126.9, 126.1, 125.5, 122.4, 120.7, 114.5, 112.5, 112.3, 85.2, 61.8, 55.7, 27.9, 18.8, 14.0 ppm.

HRMS (ESI, m/z): calculated for $C_{33}H_{33}NO_9SNa^+$: 642.1774 $(M+Na)^+$, found: 642.1772.

$[\alpha]^{21}_D$ = -58.1 (c = 1.0 in $CHCl_3$).

HPLC analysis: 92:8 *e.r.* (CHIRALPAK AD-H column, ^{i}PrOH/Hexane = 20:80, flow rate = 0.5 mL/min, λ = 220 nm, t_{major} = 21.6 min, t_{minor}= 25.5 min).

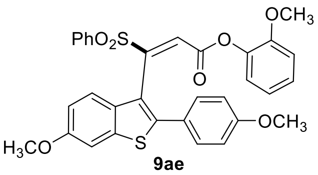

Yellow oil, 60% yield, *E*/*Z* 5:1.

^{1}H NMR (a mixture of *E*/*Z* 5:1 isomers, 400 MHz, $CDCl_3$) δ 7.83 (s, 1H), 7.59 – 7.53 (m, 0.6H), 7.43 – 7.34 (m, 3.6H), 7.33 – 6.94 (m, 11.5H), 6.92 – 6.77 (m, 3.7H), 6.77 – 6.70 (m, 3.7H), 3.86 (s, 0.6H), 3.86 (s, 0.6H), 3.83 (s, 3H), 3.81 (s, 3H), 3.79 (s, 0.6H), 3.58 (s, 3H) ppm.

^{13}C NMR (a mixture of *E*/*Z* 5:1 isomers, 101 MHz, $CDCl_3$) δ 161.6, 159.7, 157.6, 152.3, 150.8, 142.7, 141.7, 139.3, 139.0, 137.8, 136.7, 134.9, 134.3, 134.1, 133.9, 131.4, 129.9, 129.9, 129.2, 128.7, 127.6, 127.4, 126.2, 124.0, 123.7, 123.5, 122.3, 121.3, 120.7, 119.0, 114.7, 114.2, 114.1, 112.6, 112.5, 104.3, 56.1, 55.7, 55.4 ppm.
HRMS (ESI, m/z): calculated for $C_{32}H_{26}O_7S_2H^+$: 587.1198 $(M+H)^+$, found: 587.1194.
$[\alpha]^{21}_D$ = -34.0 (c = 3.0 in $CHCl_3$).
HPLC analysis for the *E* isomer: 94:6 *e.r.* (CHIRALPAK AD-H column, ^{i}PrOH/Hexane = 24:76, flow rate = 0.5 mL/min, λ = 254 nm, t_{major} = 39.3 min, t_{minor}= 41.5 min).

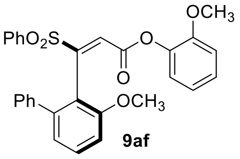

9af

Light yellow oil, 65% yield, *E*/*Z* 2:1.
^{1}H NMR (a mixture of *E*/*Z* 2:1 isomers, 400 MHz, $CDCl_3$) δ 7.81 (d, *J* = 7.0 Hz, 1H), 7.57 – 7.46 (m, 5H), 7.43 – 7.30 (m, 12H), 7.24 – 7.14 (m, 1.5H), 7.01 – 6.86 (m, 5.5H), 6.76 (d, *J* = 8.7 Hz, 0.5H), 6.70 (s, 0.5H), 6.66 (d, *J* = 8.3 Hz, 1H), 3.84 (s, 1.5H), 3.78 (s, 3H), 3.31 (s, 1.5H), 3.30 (s, 3H) ppm.
^{13}C NMR (a mixture of *E*/*Z* 2:1 isomers, 101 MHz, $CDCl_3$) δ 162.8, 161.7, 157.4, 156.5, 154.0, 151.2, 151.0, 144.6, 144.0, 143.6, 140.7, 140.3, 139.8, 139.3, 139.2, 138.8, 134.6, 133.5, 133.2, 130.9, 130.8, 130.7, 129.9, 129.5, 129.3, 129.0, 128.6, 128.5, 128.1, 127.9, 127.4, 127.3, 127.3, 123.6, 123.0, 122.5, 121.2, 120.8, 119.8, 117.5, 112.6, 112.6, 109.3, 108.9, 56.0, 55.9, 55.3, 55.2 ppm.
HRMS (ESI, m/z): calculated for $C_{29}H_{24}O_6SNa^+$: 523.1191 $(M+Na)^+$, found: 523.1193.
$[\alpha]^{21}_D$ = -82.0 (c = 3.0 in $CHCl_3$).
HPLC analysis for the *E* isomer: 90.5:9.5 *e.r.* (CHIRALCEL OD-H column, ^{i}PrOH/Hexane = 20:80, flow rate = 0.5 mL/min, λ = 254 nm, t_{minor} = 20.9 min, t_{major} = 23.0 min).

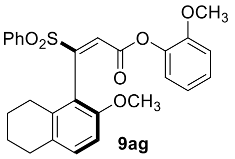

9ag

Light yellow oil, 90% yield, *E*/*Z* 2:1.
^{1}H NMR (a mixture of *E*/*Z* 2:1 isomers, 400 MHz, $CDCl_3$) δ 7.74 – 7.66 (m, 3H), 7.62 – 7.54 (m, 2.5H), 7.49 – 7.41 (m, 3.5H), 7.16 – 7.09 (m, 1.5H), 6.98 (d, *J* = 8.5 Hz, 1H), 6.90 – 6.78 (m, 5H), 6.39 (d, *J* = 8.5 Hz, 1H), 6.31 (s, 0.5H), 3.75 (s, 1.5H), 3.72 (s, 3H), 3.26 (s, 1.5H), 3.09 (s, 3H), 2.83 – 2.51 (m, 6H), 1.76 – 1.63 (m, 6H) ppm.
^{13}C NMR ((a mixture of *E*/*Z* 2:1 isomers, 101 MHz, $CDCl_3$) δ 161.7, 161.5, 154.8, 154.4, 154.3, 153.2, 151.1, 151.0, 140.4, 139.3, 139.3, 138.6, 138.5, 138.0, 133.7, 131.6, 131.0, 129.7, 129.7, 129.5, 128.8, 128.6, 128.6, 127.3, 127.2, 127.2, 122.6, 122.5, 120.8, 120.7, 117.7, 115.8, 112.6, 110.7, 107.5, 55.9, 55.9, 55.2, 54.8, 30.0, 29.2, 28.5, 27.3, 23.3, 23.0, 23.0, 22.9 ppm.
HRMS (ESI, m/z): calculated for $C_{27}H_{26}O_6SNa^+$: 501.1348 $(M+Na)^+$, found: 501.1348.
$[\alpha]^{21}_D$ = +3.2 (c = 3.0 in $CHCl_3$).
HPLC analysis for the *E* isomer: 96.5:3.5 *e.r.* (CHIRALPAK AD-H column, ^{i}PrOH/Hexane =

24:76, flow rate = 0.5 mL/min, λ = 254 nm, t_{major} = 14.6 min, t_{minor}= 16.5 min)

5.2 Further Transformations of the Products

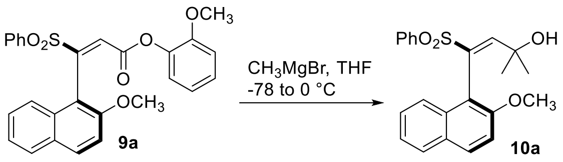

To a solution of compound **9a** (150.0 mg, 0.32 mmol) in THF (3 mL) was added CH_3MgBr (3.0M in Et_2O, 0.32 mL, 0.96 mmol) dropwise at -78 °C. The mixture was allowed to warm to 0 °C gradually and then stirred for an additional 30 min. The reaction was quenched with phosphate buffer (pH = 7.0, 5 mL) at 0 °C, and the mixture was extracted with EtOAc (50 mL x 3). The combined organic phases were washed sequentially with water (8 mL) and brine (8 mL), dried over anhydrous Na_2SO_4 and concentrated in *vacuo*. The residue was purified by flash chromatography on silica gel (EtOAc/hexanes =1:5) to provide **10a** (113.8 mg, 93% yield, 98:2 *e.r.*, *E*/*Z* >20:1) as a colorless oil.

^{1}H NMR (400 MHz, $CDCl_3$) δ 7.84 (d, *J* = 9.0 Hz, 1H), 7.77 – 7.67 (m, 2H), 7.58 (s, 1H), 7.52 – 7.43 (m, 3H), 7.41 (ddd, *J* = 8.4, 6.8, 1.4 Hz, 1H), 7.32 (tdd, *J* = 8.9, 7.2, 3.8 Hz, 3H), 6.99 (d, *J* = 9.1 Hz, 1H), 3.29 (s, 3H), 1.73 (s, 1H), 1.28 (br s, 3H), 1.17 (s, 3H) ppm.

^{13}C NMR (101 MHz, $CDCl_3$) δ 154.3, 150.3, 139.4, 136.1, 133.6, 133.0, 131.9, 129.2, 128.8, 128.3, 127.9, 127.3, 125.7, 124.2, 112.8, 111.8, 71.6, 55.5, 29.6, 28.7 ppm.

HRMS (ESI, m/z): calculated for $C_{22}H_{22}O_4SNa^+$: 405.1136 $(M+Na)^+$, found: 405.1134.

$[\alpha]^{21}_D$ = +17.5 (c = 2.0 in $CHCl_3$).

HPLC analysis: 98:2 *e.r.* (CHIRALPAK AD-H column, ^{i}PrOH/Hexane = 20:80, flow rate = 0.5 mL/min, λ = 254 nm, t_{minor} = 26.8 min, t_{major} = 29.2 min)

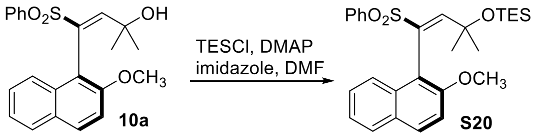

To a solution of compound **10a** (107.8 mg, 0.28 mmol) in DMF (2 mL) was added imidazole (114.4 mg, 1.68 mmol), TESCl (0.15 mL, 0.84 mmol) and DMAP (1.7 mg, 0.014 mmol) sequentially at 0 °C. The mixture was allowed to warm to room temperature and then stirred for an additional 1 h. The reaction was quenched with phosphate buffer (pH = 7.0, 10 mL) at 0 °C, and the mixture was extracted with EtOAc (50 mL x 3). The combined organic phases were washed sequentially with water (10 mL x 4) and brine (10 mL), dried over anhydrous Na_2SO_4 and concentrated in *vacuo*. The residue was purified by flash chromatography on silica gel (EtOAc/hexanes =1:20) to provide **S20** (123.8 mg, 89% yield, 98:2 *e.r.*, *E*/*Z* >20:1) as a

colorless oil.

^{1}H NMR (400 MHz, $CDCl_3$) δ 7.81 (d, *J* = 9.0 Hz, 1H), 7.74 – 7.69 (m, 2H), 7.53 (s, 1H), 7.50 – 7.44 (m, 3H), 7.38 (ddd, *J* = 8.5, 6.8, 1.4 Hz, 1H), 7.33 – 7.27 (m, 3H), 6.94 (d, *J* = 9.0 Hz, 1H), 3.25 (s, 3H), 1.14 (s, 3H), 1.10 (s, 3H), 0.78 (t, *J* = 7.9 Hz, 9H), 0.37 – 0.28 (m, 6H) ppm.
^{13}C NMR (101 MHz, $CDCl_3$) δ 154.4, 151.9, 139.8, 134.8, 134.0, 132.8, 131.3, 129.3, 128.6, 128.1, 127.7, 126.8, 126.1, 123.9, 113.4, 111.5, 74.2, 54.9, 29.8, 28.9, 7.0, 6.2 ppm.
HRMS (ESI, m/z): calculated for $C_{28}H_{36}O_4SSiNa^+$: 519.2001 $(M+Na)^+$, found: 519.2001.
$[\alpha]^{21}_D$ = -7.6 (c = 1.0 in $CHCl_3$).
HPLC analysis: 98:2 *e.r.* (CHIRALCEL OD-H column, ^{i}PrOH/Hexane = 2:98, flow rate = 0.5 mL/min, λ = 220 nm, t_{minor} = 18.9 min, t_{major} = 23.2 min)

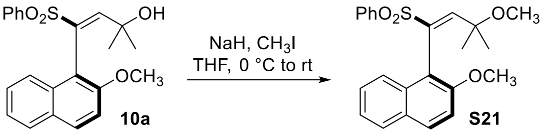

To a solution of compound **10a** (83.3 mg, 0.22 mmol) in THF (2 mL) was added NaH (60 % dispersion in mineral oil, 26.1 mg, 0.65 mmol) and methyl iodide (41 μL, 0.65 mmol) sequentially at 0 °C. The mixture was allowed to warm to room temperature gradually and then stirred for an additional 1 h. The reaction was quenched with phosphate buffer (pH = 7.0, 10 mL) at 0 °C, and the mixture was extracted with EtOAc (50 mL x 3). The combined organic phases were washed sequentially with water (10 mL) and brine (10 mL), dried over anhydrous Na_2SO_4 and concentrated in *vacuo*. The residue was purified by flash chromatography on silica gel (EtOAc/hexanes =1:10) to provide **S21** (76.0 mg, 87% yield, 98:2 *e.r.*, *E*/*Z* >20:1) as a white solid.
^{1}H NMR (400 MHz, $CDCl_3$) δ 7.84 (d, *J* = 9.0 Hz, 1H), 7.73 (d, *J* = 7.5 Hz, 1H), 7.70 (d, *J* = 8.5 Hz, 1H), 7.56 (s, 1H), 7.52 – 7.44 (m, 3H), 7.40 (ddd, *J* = 8.5, 6.8, 1.4 Hz, 1H), 7.35 – 7.27 (m, 3H), 6.97 (d, *J* = 9.0 Hz, 1H), 3.27 (s, 3H), 3.22 (s, 3H), 0.95 (s, 3H), 0.94 (s, 3H) ppm.
^{13}C NMR (101 MHz, $CDCl_3$) δ 154.6, 150.0, 139.6, 138.3, 133.8, 132.9, 131.9, 129.1, 128.5, 128.2, 127.9, 127.1, 125.5, 124.0, 112.7, 111.5, 76.3, 55.1, 51.6, 25.1, 24.2 ppm.
HRMS (ESI, m/z): calculated for $C_{23}H_{24}O_4SNa^+$: 419.1293 $(M+Na)^+$, found: 419.1296.
$[\alpha]^{21}_D$ = +5.82 (c = 1.0 in $CHCl_3$).
HPLC analysis: 98:2 *e.r.* (CHIRALPAK AD-H column, ^{i}PrOH/Hexane = 20:80, flow rate = 0.5 mL/min, λ = 254 nm, t_{major} = 13.7 min, t_{minor}= 15.4 min)

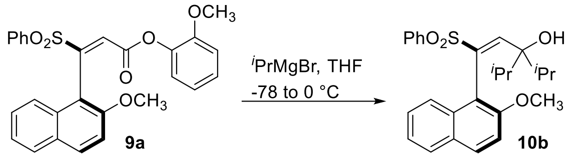

To a solution of compound **9a** (30.0 mg, 0.063 mmol) in THF (1 mL) was added ^{i}PrMgBr (2.0M in Et_2O, 0.10 mL, 0.20 mmol) dropwise at -78 °C. The mixture was allowed to warm to 0 °C gradually and then stirred for an additional 30 min. The reaction was quenched with phosphate buffer (pH = 7.0, 5 mL) at 0 °C, and the mixture was extracted with EtOAc (30 mL x 3). The combined organic phases were washed sequentially with water (8 mL) and brine (8 mL), dried over anhydrous Na_2SO_4 and concentrated in *vacuo*. The residue was purified by flash chromatography on silica gel (EtOAc/hexanes =1:10) to provide **10b** (23.5 mg, 85% yield, 93:7 *e.r.*, *E*/*Z* >20:1) as a colorless oil.

^{1}H NMR (400 MHz, $CDCl_3$) δ 7.90 (d, *J* = 8.5 Hz, 1H), 7.83 (d, *J* = 9.0 Hz, 1H), 7.73 (d, *J* = 8.1 Hz, 1H), 7.55 – 7.45 (m, 4H), 7.44 – 7.38 (m, 1H), 7.37 – 7.29 (m, 3H), 6.95 (d, *J* = 9.1 Hz, 1H), 3.22 (s, 3H), 2.13 – 2.02 (m, 1H), 2.02 – 1.90 (m, 1H), 1.71 (s, 1H), 1.00 (d, *J* = 4.8 Hz, 3H), 0.98 (d, *J* = 4.7 Hz, 3H), 0.87 (d, *J* = 6.8 Hz, 3H), 0.80 (d, *J* = 6.8 Hz, 3H) ppm.

^{13}C NMR (101 MHz, $CDCl_3$) δ 154.3, 149.5, 140.2, 138.6, 134.0, 132.9, 132.1, 129.1, 128.9, 128.3, 127.8, 127.1, 126.6, 124.2, 112.6, 111.3, 82.0, 54.7, 35.7, 35.2, 18.4, 18.1, 17.7, 16.6 ppm.

HRMS (ESI, m/z): calculated for $C_{26}H_{30}O_4SNa^+$: 461.1762 $(M+Na)^+$, found: 461.1763.

$[\alpha]^{21}_D$ = +11.3 (c = 1.0 in $CHCl_3$).

HPLC analysis: 93:7 *e.r.* (CHIRALPAK AD-H column, ^{i}PrOH/Hexane = 20:80, flow rate = 0.5 mL/min, λ = 254 nm, t_{minor} = 10.3 min, t_{major} = 11.0 min)

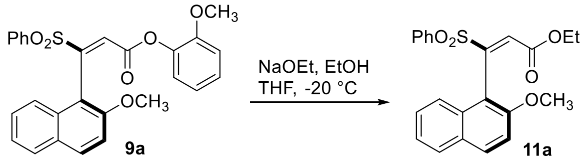

NaH (60 % dispersion in mineral oil, 3.8 mg, 0.095 mmol) was added to isopropanol (1 mL) at 0 °C. The mixture was warmed to room temperature and stirred for 2 min. Then the mixture was added to a solution of compound **9a** (29.7 mg, 0.063 mmol) in THF (0.5 mL) dropwise at -20 °C. After being stirred for 30 min at -20 °C, the reaction was quenched with phosphate buffer (pH = 7.0, 5 mL). The mixture was extracted with EtOAc (50 mL x 3). The combined organic phases were washed sequentially with water (8 mL) and brine (8 mL), dried over anhydrous Na_2SO_4 and concentrated in *vacuo*. The residue was purified by flash chromatography on silica gel (EtOAc/hexanes =1:10) to provide **11a** (22.8 mg, 92% yield, 98:2 *e.r.*, *E*/*Z* >20:1) as a colorless oil.

^{1}H NMR (400 MHz, $CDCl_3$) δ 7.85 (d, *J* = 9.1 Hz, 1H), 7.74 (dd, *J* = 8.3, 1.3 Hz, 1H), 7.60 – 7.49 (m, 5H), 7.39 – 7.28 (m, 4H), 7.02 (d, *J* = 9.1 Hz, 1H), 3.87 (q, *J* = 7.1 Hz, 2H), 3.33 (s, 3H), 0.79 (t, *J* = 7.1 Hz, 3H) ppm.
^{13}C NMR (101 MHz, $CDCl_3$) δ 163.5, 154.3, 152.0, 138.3, 133.7, 133.0, 131.9, 131.1, 129.6, 128.6, 127.9, 127.2, 124.7, 124.0, 112.7, 111.8, 61.1, 55.7, 13.6 ppm.
HRMS (ESI, m/z): calculated for $C_{22}H_{20}O_5SH^+$: 397.1110 $(M+H)^+$, found: 397.1110.
$[\alpha]^{21}_D$ = -0.5 (c = 1.0 in $CHCl_3$).
HPLC analysis: 98:2 *e.r.* (CHIRALPAK AD-H column, ^{i}PrOH/Hexane = 24:76, flow rate = 0.5 mL/min, λ = 220 nm, t_{major} = 15.6 min, t_{minor} = 17.8 min)

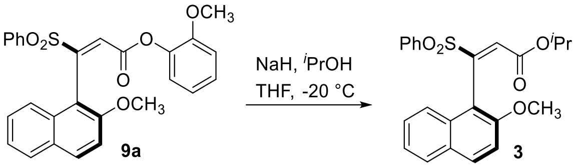

NaH (60 % dispersion in mineral oil, 3.8 mg, 0.095 mmol) was added to isopropanol (1 mL) at 0 °C. The mixture was warmed to room temperature and stirred for 2 min. Then the mixture was added to a solution of compound **9a** (29.1 mg, 0.061 mmol) in THF (0.5 mL) dropwise at -20 °C. After being stirred for 30 min at -20 °C, the reaction was quenched with phosphate buffer (pH = 7.0, 5 mL). The mixture was extracted with EtOAc (50 mL x 3). The combined organic phases were washed sequentially with water (8 mL) and brine (8 mL), dried over anhydrous Na_2SO_4 and concentrated in *vacuo*. The residue was purified by flash chromatography on silica gel (EtOAc/hexanes =1:10) to provide **3** (21.8 mg, 87% yield, 97:3 *e.r.*, *E*/*Z* >20:1) as a colorless oil.
^{1}H, **^{13}C NMR** and **HRMS** data are the same as the previously reported analysis data of **3**.
$[\alpha]^{21}_D$ = -28.3 (c = 0.6 in $CHCl_3$).
HPLC analysis: 97:3 *e.r.* (CHIRALPAK AD-H column, ^{i}PrOH/Hexane = 24:76, flow rate = 0.5 mL/min, λ = 254 nm, t_{major} = 14.1 min, t_{minor} = 19.5 min)

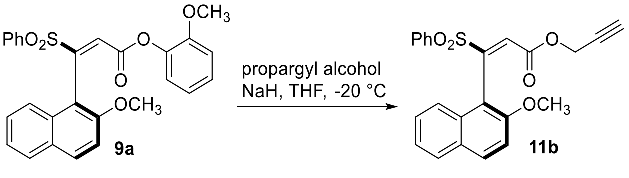

To a solution of propargyl alcohol (36.7 μL, 0.64 mmol) in THF (1 mL) was added NaH (60 % dispersion in mineral oil, 3.8 mg, 0.095 mmol) at 0 °C. The mixture was warmed to room temperature and stirred for 5 min. Then the mixture was added to a stirred solution of compound **9a** (30.2 mg, 0.064 mmol) in THF (0.6 mL) dropwise at -20 °C. After being stirred for 60 min at -20 °C, the reaction was quenched with phosphate buffer (pH = 7.0, 5 mL). The mixture was

extracted with EtOAc (50 mL x 3). The combined organic phases were washed sequentially with water (8 mL) and brine (8 mL), dried over anhydrous Na_2SO_4 and concentrated in *vacuo*. The residue was purified by flash chromatography on silica gel (EtOAc/hexanes =1:10) to provide **11b** (20.6 mg, 79% yield, 96.5:3.5 *e.r.*, *E*/*Z* >20:1) as a colorless oil.

^{1}H NMR (400 MHz, $CDCl_3$) δ 7.86 (d, *J* = 9.0 Hz, 1H), 7.74 (dd, *J* = 8.4, 1.3 Hz, 1H), 7.59 – 7.50 (m, 5H), 7.40 – 7.29 (m, 4H), 7.02 (d, *J* = 9.0 Hz, 1H), 4.47 (d, *J* = 2.5 Hz, 2H), 3.34 (s, 3H), 2.31 (t, *J* = 2.5 Hz, 1H) ppm.

^{13}C NMR (101 MHz, $CDCl_3$) δ 162.6, 154.3, 153.8, 138.0, 133.8, 132.8, 132.1, 129.7, 129.6, 128.6, 128.6, 128.0, 127.3, 124.6, 124.1, 112.2, 111.8, 75.3, 55.7, 52.6 ppm.

HRMS (ESI, m/z): calculated for $C_{23}H_{18}O_5SNa^+$: 429.0773 $(M+Na)^+$, found: 429.0774.

$[\alpha]^{21}_D$ = -11.9 (c = 1.0 in $CHCl_3$).

HPLC analysis: 96.5:3.5 *e.r.* (CHIRALPAK AD-H column, ^{i}PrOH/Hexane = 24:76, flow rate = 0.5 mL/min, λ = 220 nm, t_{minor}= 22.9 min, t_{major} = 24.8 min)

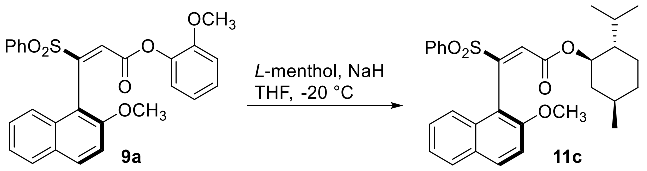

To a solution of *L*-menthol (51.6 mg, 0.33 mmol) in THF (1 mL) was added NaH (60 % dispersion in mineral oil, 5.3 mg, 0.13 mmol) at 0 °C. The mixture was warmed to room temperature and stirred for 10 min. Then the mixture was added to a solution of compound **9a** (31.4 mg, 0.066 mmol) in THF (0.6 mL) dropwise at -20 °C. After being stirred for 60 min at -20 °C, the reaction was quenched with phosphate buffer (pH = 7.0, 5 mL). The mixture was extracted with EtOAc (50 mL x 3). The combined organic phases were washed sequentially with water (8 mL) and brine (8 mL), dried over anhydrous Na_2SO_4 and concentrated in *vacuo*. The residue was purified by flash chromatography on silica gel (EtOAc/hexanes =1:10) to provide **11c** (21.7 mg, 65% yield, > 20:1 *d.r.*, *E*/*Z* >20:1) as a colorless oil.

^{1}H NMR (400 MHz, $CDCl_3$) δ 7.85 (d, *J* = 9.0 Hz, 1H), 7.73 (dd, *J* = 8.3, 1.2 Hz, 1H), 7.62 – 7.57 (m, 2H), 7.57 – 7.51 (m, 2H), 7.50 (s, 1H), 7.39 – 7.28 (m, 4H), 6.99 (d, *J* = 9.1 Hz, 1H), 4.35 (td, *J* = 10.8, 4.3 Hz, 1H), 3.33 (s, 3H), 1.51 – 1.36 (m, 3H), 1.23 – 1.10 (m, 2H), 0.81 – 0.69 (m, 1H), 0.66 (d, *J* = 6.5 Hz, 3H), 0.59 (d, *J* = 7.0 Hz, 3H), 0.58 – 0.49 (m, 2H), 0.40 (d, *J* = 6.9 Hz, 3H), 0.12 – 0.01 (m, 1H) ppm.

^{13}C NMR (101 MHz, $CDCl_3$) δ 163.5, 154.4, 150.8, 138.4, 133.7, 133.2, 131.8, 129.7, 128.6, 128.5, 127.8, 127.3, 124.8, 124.0, 112.9, 111.6, 75.3, 55.5, 46.6, 39.9, 34.0, 31.2, 25.4, 22.8, 21.9, 20.9, 15.7 ppm.

HRMS (ESI, m/z): calculated for $C_{30}H_{34}O_5SNa^+$: 529.2025 $(M+Na)^+$, found: 529.2029.

[α]$^{21}_{D}$ = -33.8 (c = 1.0 in $CHCl_3$).

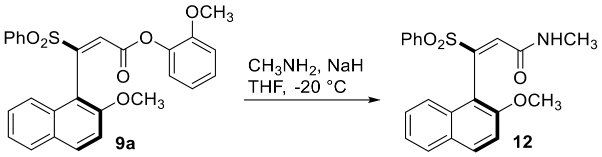

To a solution of methylamine (2.0M in THF, 0.4 mL, 0.8 mmol) in THF (0.5 mL) was added NaH (60 % dispersion in mineral oil, 6.6 mg, 0.17 mmol) at 0 °C. The mixture was warmed to room temperature and stirred for 10 min. Then the mixture was added to a solution of compound **9a** (39.2 mg, 0.083 mmol) in THF (1 mL) dropwise at -20 °C. After being stirred for 60 min at -20 °C, the reaction was quenched with phosphate buffer (pH = 7.0, 5 mL). The mixture was extracted with EtOAc (50 mL x 3). The combined organic phases were washed sequentially with water (8 mL) and brine (8 mL), dried over anhydrous Na_2SO_4 and concentrated in *vacuo*. The residue was purified by flash chromatography on silica gel (EtOAc/hexanes =1:10) to provide **12** (26.6 mg, 84% yield, 98:2 *e.r.*, *E*/*Z* >20:1) as a colorless oil.

^{1}H NMR (400 MHz, $CDCl_3$) δ 7.89 (d, *J* = 9.0 Hz, 1H), 7.78 – 7.72 (m, 1H), 7.61 (s, 1H), 7.55 – 7.48 (m, 4H), 7.38 – 7.29 (m, 4H), 7.09 (d, *J* = 9.1 Hz, 1H), 5.79 (s, 1H), 3.43 (s, 3H), 2.47 (d, *J* = 5.0 Hz, 3H) ppm.

^{13}C NMR (101 MHz, $CDCl_3$) δ 164.1, 154.6, 145.0, 138.5, 135.4, 133.6, 132.9, 132.6, 129.5, 128.8, 128.6, 128.1, 127.8, 124.7, 124.5, 112.2, 111.8, 56.1, 26.3 ppm.

HRMS (ESI, m/z): calculated for $C_{21}H_{19}NO_4SH^+$: 382.1113 $(M+H)^+$, found: 382.1114.

[α]$^{21}_{D}$ = +24.1 (c = 1.0 in $CHCl_3$).

HPLC analysis: 98:2 *e.r.* (CHIRALPAK AD-H column, ^{i}PrOH/Hexane = 30:70, flow rate = 0.5 mL/min, λ = 254 nm, t_{major} = 15.7 min, t_{minor} = 17.9 min)

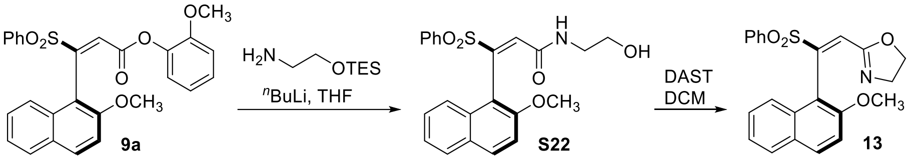

To a solution of **9a** (89.0 mg, 0.51 mmol) in THF (1 mL) was added ^{n}BuLi (2.0 M in cyclohexane, 0.25 mL, 0.5 mmol) at -20 °C. The mixture was warmed to room temperature and stirred for 5 min. Then the mixture was added to a solution of compound **9a** (80.0 mg, 0.17 mmol) in THF (1.5 mL) dropwise at -20 °C. After being stirred for 20 min at -20 °C, the reaction was quenched with phosphate buffer (pH = 7.0, 5 mL). The mixture was extracted with EtOAc (50 mL x 3). The combined organic phases were washed sequentially with water (8 mL) and brine (8 mL), dried over anhydrous Na_2SO_4 and concentrated in *vacuo*. The residue was purified by flash chromatography on silica gel (EtOAc/hexanes =2:1) to provide **S22** (58.7 mg, 84%

yield, 98:2 *e.r.*, *E*/*Z* >20:1) as a colorless oil.

¹H NMR (400 MHz, $CDCl_3$) δ 7.89 (d, *J* = 9.1 Hz, 1H), 7.75 (dd, *J* = 7.5, 1.9 Hz, 1H), 7.62 (s, 1H), 7.57 – 7.49 (m, 4H), 7.39 – 7.30 (m, 4H), 7.08 (d, *J* = 9.1 Hz, 1H), 6.30 (s, 1H), 3.42 (s, 3H), 3.23 – 3.11 (m, 1H), 3.09 – 2.94 (m, 3H), 1.43 (s, 1H) ppm.

¹³C NMR (101 MHz, $CDCl_3$) δ 164.1, 154.6, 145.1, 138.4, 135.4, 133.7, 132.8, 132.5, 129.5, 128.6, 128.0, 127.9, 124.6, 124.6, 112.0, 111.8, 61.4, 56.0, 41.7 ppm.

HRMS (ESI, m/z): calculated for $C_{22}H_{21}NO_5SH^+$: 412.1219 $(M+H)^+$, found: 412.1222.

$[\alpha]^{21}_D$ = +15.1 (c = 0.3 in $CHCl_3$).

HPLC analysis: 98:2 *e.r.* (CHIRALCEL AS-H column, ^{i}PrOH/Hexane = 30:70, flow rate = 0.5 mL/min, λ = 220 nm, t_{minor} = 28.1 min, t_{major} = 42.0 min)

To a solution of compound **S22** (30.0 mg, 0.073 mmol) in DCM (1.5 mL) was added diethylaminosulfur trifluoride (14.4 μL, 0.11 mmol) dropwise at -78 °C. The mixture was stirred for an additional 1h at -78 °C. Then K_2CO_3 (30.0 mg, 0.22 mmol) was added, and the mixture was allowed to warm to room temperature. The reaction mixture was poured into a saturated solution of $NaHCO_3$ (10 mL) and extracted with DCM. The combined organic phases were washed sequentially with water (8 mL) and brine (8 mL), dried over anhydrous Na_2SO_4 and concentrated in *vacuo*. The residue was purified by flash chromatography on silica gel (EtOAc/hexanes =1:10) to provide **13** (22.5 mg, 78% yield, 98:2 *e.r.*, *E*/*Z* >20:1) as a colorless oil.

¹H NMR (400 MHz, $CDCl_3$) δ 7.84 (d, *J* = 9.0 Hz, 1H), 7.77 (s, 1H), 7.73 (dd, *J* = 8.0, 1.6 Hz, 1H), 7.56 – 7.47 (m, 4H), 7.35 – 7.27 (m, 4H), 7.02 (d, *J* = 9.1 Hz, 1H), 3.90 – 3.79 (m, 1H), 3.78 – 3.69 (m, 3H), 3.34 (s, 3H) ppm.

¹³C NMR (101 MHz, $CDCl_3$) δ 162.3, 154.7, 147.8, 138.8, 133.5, 133.3, 131.6, 129.4, 128.7, 128.5, 128.5, 127.8, 127.0, 125.0, 123.8, 113.2, 112.0, 68.0, 55.8, 54.7 ppm.

HRMS (ESI, m/z): calculated for $C_{22}H_{19}NO_4SH^+$: 394.1113 $(M+H)^+$, found: 394.1117.

$[\alpha]^{21}_D$ = -156.5 (c = 0.3 in $CHCl_3$).

HPLC analysis: 98:2 *e.r.* (CHIRALCEL AS-H column, ^{i}PrOH/Hexane = 30:70, flow rate = 0.5 mL/min, λ = 254 nm, t_{major} = 33.5 min, t_{minor} = 46.6 min)

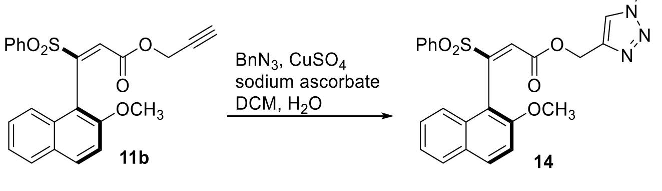

To a solution of compound **11b** (11.9 mg, 0.029 mmol) in DCM (1 mL) and H_2O (1 mL) was added BnN_3 (4.3 mg, 0.032 mmol), $CuSO_4$ (1.0 mg, 0.006 mmol) and sodium ascorbate (2.3 mg, 0.012 mmol) sequentially at room temperature. After being stirred for 2 h, the reaction mixture was cooled to 0 °C and quenched with a saturated aqueous solution of $NaHCO_3$ (5 mL). The mixture was extracted with DCM (50 mL x 3). The combined organic phases were washed sequentially with water (8 mL) and brine (8 mL), dried over anhydrous Na_2SO_4 and

concentrated in *vacuo*. The residue was purified by flash chromatography on silica gel (EtOAc/hexanes =1:10) to provide **14** (14.9 mg, 95% yield, 96.5:3.5 *e.r.*, *E*/*Z* >20:1) as a colorless oil.

^{1}H NMR (400 MHz, $CDCl_3$) δ 7.73 (d, *J* = 9.1 Hz, 1H), 7.71 – 7.66 (m, 1H), 7.55 – 7.48 (m, 5H), 7.38 – 7.29 (m, 7H), 7.20 – 7.14 (m, 2H), 6.90 (d, *J* = 9.1 Hz, 1H), 6.69 (s, 1H), 5.35 (s, 2H), 4.97 (d, *J* = 1.9 Hz, 2H), 3.25 (s, 3H) ppm.

^{13}C NMR (101 MHz, $CDCl_3$) δ 163.3, 154.3, 152.6, 138.1, 134.5, 133.8, 132.8, 131.8, 130.4, 129.6, 129.3, 129.0, 128.6, 128.4, 128.1, 127.9, 127.3, 124.8, 124.0, 123.0, 112.4, 112.0, 58.7, 55.7, 54.2 ppm.

HRMS (ESI, m/z): calculated for $C_{30}H_{25}N_3O_5SH^+$: 540.1593 $(M+H)^+$, found: 540.1600.

$[\alpha]^{21}_D$ = +2.4 (c = 1.0 in $CHCl_3$).

HPLC analysis: 96.5:3.5 *e.r.* (CHIRALPAK AD-H column, ^{i}PrOH/Hexane = 50:50, flow rate = 0.5 mL/min, λ = 220 nm, t_{major} = 25.0 min, t_{minor}= 27.4 min)

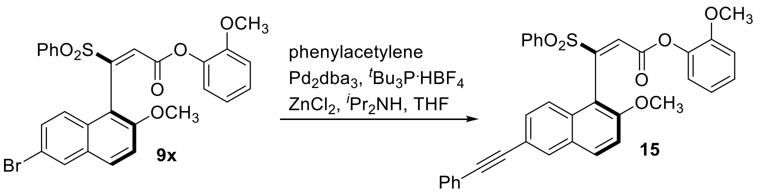

In a glove box, Pd_2dba_3 (3.9 mg, 0.0043 mmol) and $^tBu_3P{\cdot}HBF_4$ (2.5 mg, 0.0086 mmol) were dissolved in THF (0.6 mL) and diisopropylamine (0.3 mL). The mixture was stirred at room temperature for 15 min. Then compound **9x** (40.0 mg, 0.072 mmol) and zinc chloride (3.0 mg, 0.022 mmol) were added. The mixture was stirred for 15 min to dissolve the solid, and then phenylacetylene (20 µL, 0.18 mmol) was added. The resulting mixture was stirred at room temperature for 2.5 h, and then diluted with EtOAc (10 mL), filtrated through a pad of silica gel. The filtrate was concentrated in *vacuo* and the residue was purified by flash chromatography on silica gel (EtOAc/hexanes =1:10) to provide **15** (33.9 mg, 82% yield, > 99:1 *e.r.*, *E*/*Z* >20:1) as a yellow solid.

^{1}H NMR (400 MHz, $CDCl_3$) δ 7.94 (d, *J* = 1.6 Hz, 1H), 7.82 – 7.78 (m, 2H), 7.63 – 7.54 (m, 6H), 7.48 (dd, *J* = 8.8, 1.7 Hz, 1H), 7.40 – 7.34 (m, 5H), 7.09 – 7.01 (m, 2H), 6.81 – 6.74 (m, 2H), 6.68 (dd, *J* = 7.8, 1.7 Hz, 1H), 3.52 (s, 3H), 3.40 (s, 3H) ppm.

^{13}C NMR (101 MHz, $CDCl_3$) δ 161.3, 155.2, 153.1, 150.8, 139.1, 137.9, 134.0, 132.3, 131.9, 131.7, 131.4, 130.1, 129.7, 128.7, 128.5, 128.4, 128.2, 127.2, 124.9, 123.5, 122.3, 120.6, 118.7, 112.6, 112.5, 89.8, 89.6, 55.8, 55.7 ppm.

HRMS (ESI, m/z): calculated for $C_{35}H_{26}O_6SNa^+$: 597.1348 $(M+Na)^+$, found: 597.1348.

$[\alpha]^{21}_D$ = -8.2 (c = 1.0 in $CHCl_3$).

HPLC analysis: > 99:1 *e.r.* (CHIRALCEL IA column, ^{i}PrOH/Hexane = 24:76, flow rate = 0.5

mL/min, λ = 220 nm, t_{major} = 30.4 min, t_{minor} = 35.3 min)

6. DFT Calculations on the Rotation Barrier

In order to explore the possibility of racemization of the axially chiral styrenes in our study, the energetic barriers of the racemization process was investigated computationally. The ground-stage structure (**9a**), transition states (**TS9a**) and the enantiomer (*ent*-**9a**) corresponding to the rotation along two directions were located. The rotational barrier ($\Delta G^{\ddagger}$) for enantiomerization was obtained as the Gibbs free energy difference from the ground-state structure to the more stable transition state. The rate constants for enantiomerization (k_{ent}) and racemization (k_{rac}), and half-life for racemization ($t_{1/2}$) were calculated based on the following equations:[9]

$$k_{\text{ent}} = \kappa \frac{k_{\text{B}}T}{h} exp \frac{-\Delta G^{\neq}}{RT}$$

$$k_{\text{rac}} = 2k_{\text{ent}}$$

$$t_{1/2} = \frac{\ln 2}{k_{\text{rac}}}$$

where the transmission coefficient κ is set as 1, Boltzmann constant k_B = 1.3806503 × 10^{-23} J K^{-1}, Planck constant h = 6.62606876 ×10^{-34} J s, idea gas constant R = 8.314472 J mol^{-1} K^{-1}, T = 373.15 K.

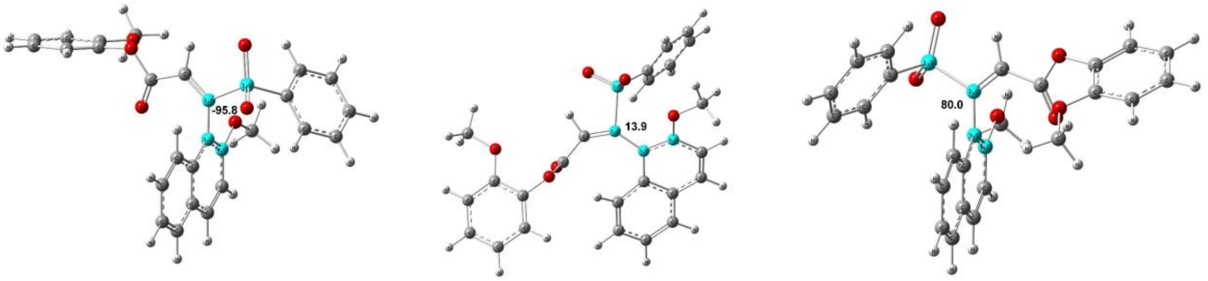

9a (0.0 kcal/mol) **TS9a** (30.1 kcal/mol) *ent*-**9a** (0.6 kcal/mol)

The DFT calculation results are shown below: $\Delta G^{\ddagger}$ =30.1 kcal/mol, and at 100 °C (373.15 K), k_{rac} = 3.56 ×10^{-5} s^{-1}, $t_{1/2}$ = 5.4 h; at 25 °C (298.15 K), k_{rac} = 1.04 ×10^{-9} s^{-1}, $t_{1/2}$ = 21.2 years. For details, please check Supplementary Data.

Supplementary Table 5. Computationally derived energy scans for **9a**. Dihedral angles were scanned from -180° to 180° in 5° increments.

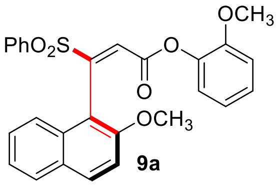

9a

Dihedral angle	ΔE /kcal mol^{-1}	Dihedral angle	ΔE /kcal mol^{-1}	Dihedral angle	ΔE /kcal mol^{-1}	Dihedral angle	ΔE /kcal mol^{-1}
-175	25.0	-85	0.6	5	24.4	95	1.9
-170	22.5	-80	1.3	10	26.5	100	2.4
-165	20.0	-75	2.3	15	28.4	105	3.4
-160	17.6	-70	3.2	20	30.1	110	4.7
-155	15.3	-65	3.5	25	11.3	115	5.9
-150	13.2	-60	3.9	30	9.7	120	6.9
-145	11.3	-55	4.5	35	8.2	125	7.9
-140	9.4	-50	5.2	40	7.0	130	9.3
-135	7.7	-45	6.2	45	6.0	135	10.8
-130	6.4	-40	7.4	50	5.1	140	12.6
-125	4.8	-35	8.8	55	4.5	145	14.5
-120	3.3	-30	10.4	60	4.1	150	16.6
-115	2.2	-25	12.1	65	3.9	155	18.9
-110	1.4	-20	13.5	70	1.4	160	21.5
-105	0.7	-15	15.5	75	1.0	165	23.9
-100	0.2	-10	17.6	80	0.8	170	25.6
-95	0.0	-5	19.8	85	0.9	175	27.3
-90	0.1	0	22.1	90	1.3	180	28.8

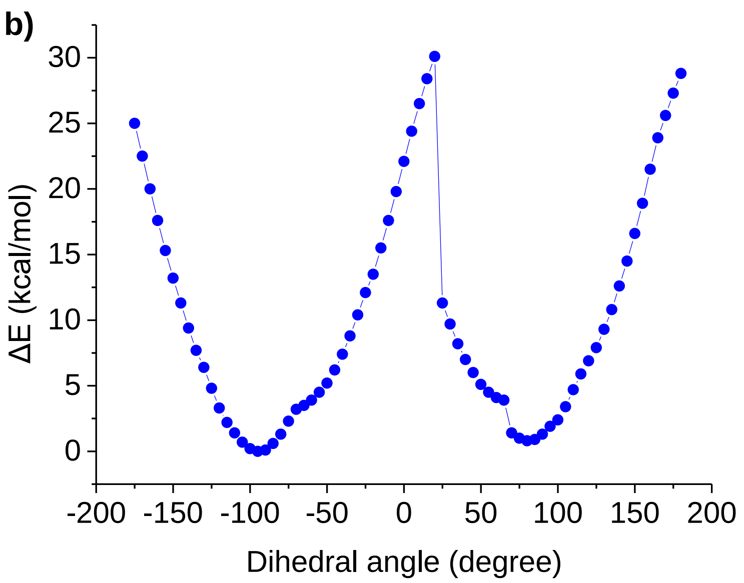

Supplementary Figure 193. Computationally derived energy scan for **9a** over dihedral angles from -180° to 180°

Computational Methods

All calculations were carried out using the Gaussian 16 C.01 program package. The geometry optimizations were performed using hybrid B3LYP exchange correlation.[13-15] The 6-31G(d, p) basis set[16-18] was used for H, C, O, and S atoms. Vibrational frequency calculations

were performed (at 303.15 K) to characterize the nature of each stationary point. A tight convergence (10^{-12} au) criterion was employed, and the solvent toluene (ε= 2.40) was considered using the SMD[19] continuum solvent model (UFF radii). Single-point calculations were carried out for each optimized structure by using M06-2X functional[20,21] and Def2-TZVP basis sets.[22,23]

The IRC calculations result for TS9a

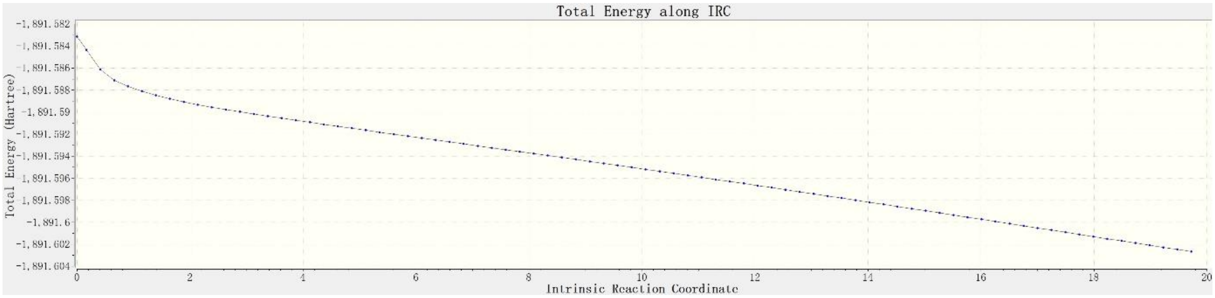

Supplementary Figure 194. Total Energy along IRC (Forward) for **TS9a**

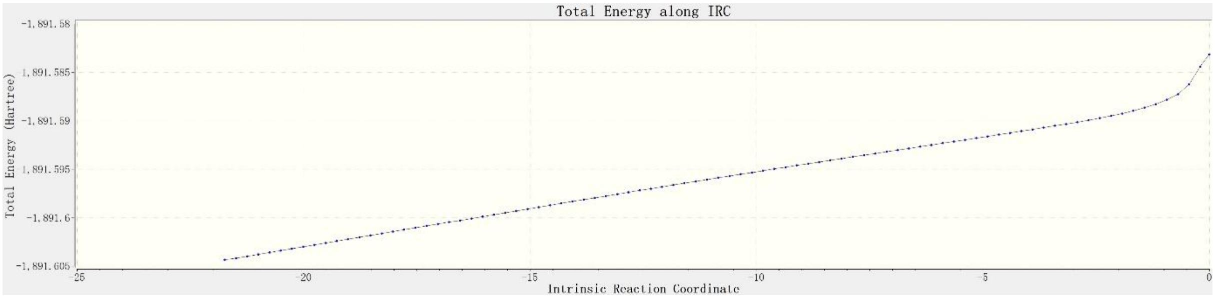

Supplementary Figure 195. Total Energy along IRC (Reverse) for **TS9a**

7. Experimental Racemization Studies

The axially chiral product **9a**, **9n** and **9o** (0.05 mmol) were dissolved in toluene (100 mL) separately and heated at 100 ºC (373.15 K) for 10 h. The *ee* value was tested every one hour *via* HPLC on chiral stationary phase. The rotation barrier was determined according to the literature method.[24-29]

Supplementary Table 6. The Detailed Racemization Result of **9a**, **9n** and **9o** over 24 h

**Time/h**	***ee* (%) (9a, 100 ºC)**	***ee* (%) (9a, 75 ºC)**	***ee* (%) (9a, 50 ºC)**	***ee* (%) (9n, 100 ºC)**	***ee* (%) (9o, 100 ºC)**
0	99.45	99.45	99.45	97.27	85.01
1	89.68	-	-	93.95	82.2
2	82.53	97.54	99.21	90.67	80.1
3	73.42	-	-	87.15	78.06
4	65.87	96.6	99.01	83.9	75.32
5	60.73	-	-	80.55	73.49
6	53.53	95.29	98.75	77.81	71.48
7	48.45	-	-	74.76	69.71
8	43.87	91.23	-	71.52	67.6
9	39.14	-	98.38	68.66	66.14
10	36.07	89.98	-	66.87	64.49
11	-	-	-	63.76	63.38
12	28.24	87.42	98.17	61.18	62.16
13	-	-	-	-	-
14	21.79	86.76	-	-	-
15	-	-	-	54.61	-
16	16.83	83.94	97.96	-	56.27
17	-	-	-	-	-
18	14.98	82.38	-	49.21	-
19	-	-	-	-	-
20	-	80.28	97.43	-	50.58
21	9.34	-	-	45.25	-
22	-	78.24	-	-	-
23	-	-	-	-	-
24	7.12	76.82	97.09	39.01	47.21

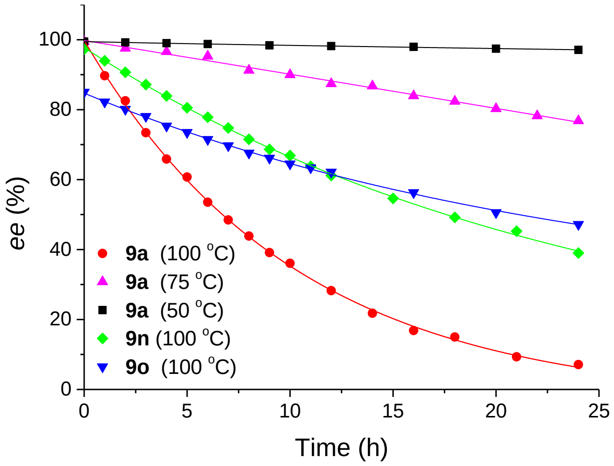

Supplementary Figure 196. The racemization results of **9a** and **9n-9o** over the time.

a) Determination of $t_{1/2rac}$ and $\Delta G^{\ddagger}$ for 9a.

The rotation barrier of **9a** was determined according to the literature method ($t_{1/2rac}$ = 6.7 h at 100 °C (373.15 K), toluene; $\Delta G^{\ddagger}$ = 30.26 kcal/mol).

Supplementary Table 7. Calculation Result for Racemization Test of **9a** at 100 °C

**Time (s)**	***ee* (%)**	***ee*$_0$/*ee*$_t$**	**ln(*ee*$_0$/*ee*$_t$)**
0	99.45	1	0
3600	89.68	1.108943	0.103407
7200	82.53	1.205016	0.186493
10800	73.42	1.354536	0.303459
14400	65.87	1.509792	0.411972
18000	60.73	1.637576	0.493217
21600	53.53	1.857837	0.619413
25200	48.45	2.052632	0.719123
28800	43.87	2.266925	0.818424
32400	39.14	2.540879	0.932510
36000	36.07	2.757139	1.014194

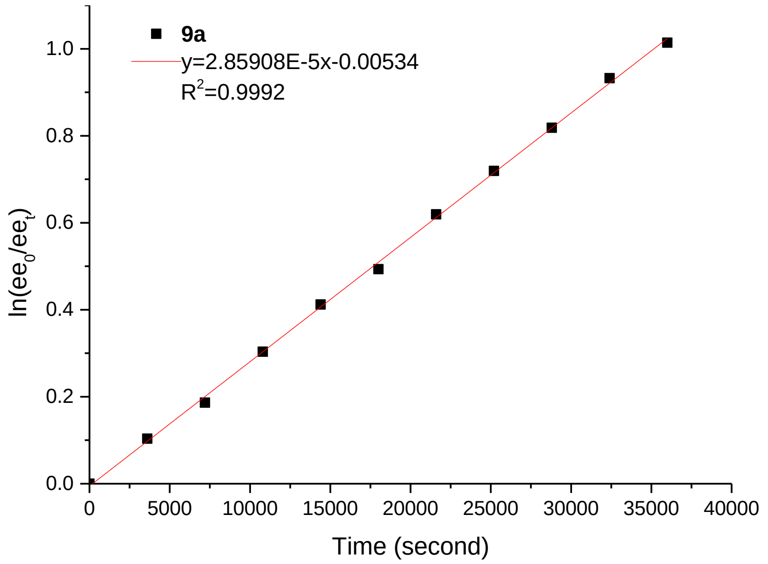

Supplementary Figure 197. Rotation Barrier Analysis of **9a**

$\ln(ee_0/ee_t)=2k_{ent}t+C$

therefore, k_{ent}=1/2 slope = 1.43 x 10^{-5}

$k_{rac}=2k_{ent}$=2.86 x 10^{-5} s^{-1}

$t_{1/2rac}=\ln2/k_{rac}$= 2.42 x 10^4 s = 6.7 h

$\Delta G^{\ddagger} = -RT\ln(k_{ent}h/k_BT)$ = 126.70 kJ/mol = 30.26 kcal/mol

b) Determination of $t_{1/2rac}$ and $\Delta G^{\ddagger}$ for 9n

The rotation barrier of **9n** was determined according to the literature method ($t_{1/2rac}$ = 18.1 h at 100 ºC (373.15 K), toluene; $\Delta G^{\ddagger}$ = 30.99 kcal/mol).

Supplementary Table 8. Calculation Result for Racemization Test of **9n** at 100 ºC

**Time (s)**	***ee* (%)**	ee_0/ee_t	$\ln(ee_0/ee_t)$
0	97.27	1	0
3600	93.95	1.035338	0.034728
7200	90.67	1.072791	0.070264
10800	87.15	1.116122	0.109860
14400	83.90	1.159356	0.147865
18000	80.55	1.207573	0.188613
21600	77.81	1.250096	0.223221
25200	74.76	1.301097	0.263208
28800	71.52	1.360039	0.307513
32400	68.66	1.416691	0.348324
36000	66.87	1.454613	0.374740

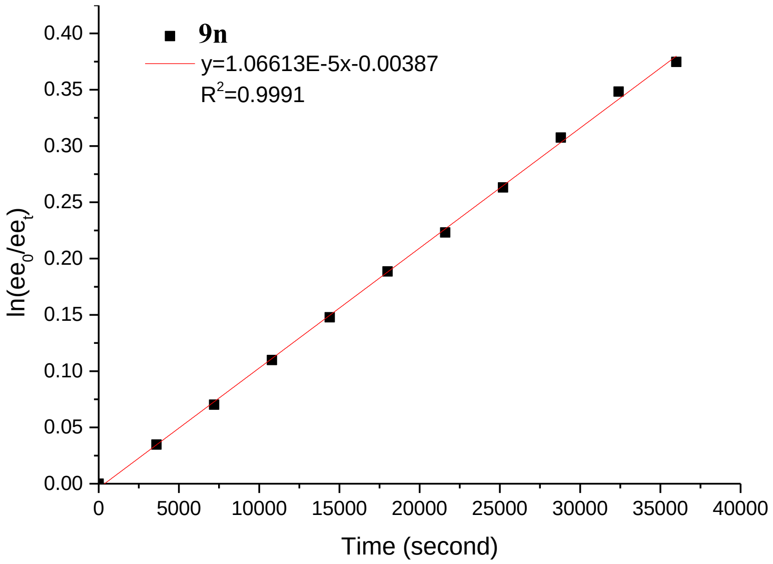

Supplementary Figure 198. Rotation Barrier Analysis of **9n**

$\ln(ee_0/ee_t)=2k_{ent}t+C$

therefore, k_{ent}=1/2 slope = 5.33 x 10^{-6}

$k_{rac}=2k_{ent}$=1.07 x 10^{-5} s^{-1}

$t_{1/2rac}=\ln2/k_{rac}$= 6.50 x 10^4 s = 18.1 h

$\Delta G^{\ddagger} = -RT\ln(k_{ent}h/k_BT)$ = 129.76 kJ/mol = 30.99 kcal/mol

b) Determination of $t_{1/2rac}$ and $\Delta G^{\ddagger}$ for 9o

The rotation barrier of **9o** was determined according to the literature method ($t_{1/2rac}$ = 25.1 h at 100 ºC (373.15 K), toluene; $\Delta G^{\ddagger}$ = 31.24 kcal/mol).

Supplementary Table 9. Calculation Result for Racemization Test of **9o** at 100 ºC

Time (s)	*ee* (%)	ee_0/ee_t	$\ln(ee_0/ee_t)$
0	85.01	1	0
3600	82.20	1.034185	0.033614
7200	80.10	1.061298	0.059493
10800	78.06	1.089034	0.085291
14400	75.32	1.128651	0.121023
18000	73.49	1.156756	0.145620
21600	71.48	1.189284	0.173351
25200	69.71	1.219481	0.198425
28800	67.60	1.257544	0.229161
32400	66.14	1.285304	0.250995
36000	64.49	1.318189	0.276259

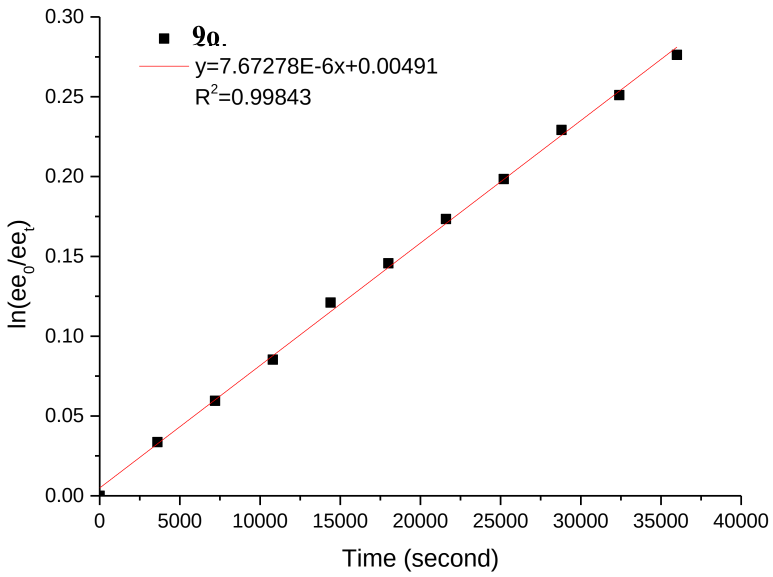

Supplementary Figure 199. Rotation Barrier Analysis of **9o**

$\ln(ee_0/ee_t)=2k_{\mathrm{ent}}t+C$

therefore, $k_{\mathrm{ent}}=1/2$ slope = 3.84×10^{-6}

$k_{\mathrm{rac}}=2k_{\mathrm{ent}}=7.67 \times 10^{-6}\ \mathrm{s}^{-1}$

$t_{1/2\mathrm{rac}}=\ln 2/k_{\mathrm{rac}}= 9.03 \times 10^{4}$ s = 25.1 h

$\Delta G^{\ddagger} = -RT\ln(k_{\mathrm{ent}}h/k_{\mathrm{B}}T)$ = 130.78 kJ/mol = 31.24 kcal/mol

8. Supplementary References

1. He, M., Struble, J.R. & Bode, J.W. Highly enantioselective azadiene Diels−Alder reactions catalyzed by chiral *N*-heterocyclic carbenes. *J. Am. Chem. Soc.* **128**, 8418-8420 (2006).

2. Wu, X. *et al.* Sulfinate and carbene co-catalyzed Rauhut-Currier reaction for enantioselective access to azepino 1,2-a indoles. *Angew. Chem. Int. Ed.* **58**, 477-481 (2019).

3. Zhao, C., Li, F. & Wang, J. *N*-Heterocyclic carbene catalyzed dynamic kinetic resolution of pyranones. *Angew. Chem. Int. Ed.* **55**, 1820-1824 (2016).

4. Zhuo, S. *et al.* Access to all-carbon spirocycles through a carbene and thiourea cocatalytic desymmetrization cascade reaction. *Angew. Chem. Int. Ed.* **58**, 1784-1788 (2019).

5. Dong, S. *et al.* Organocatalytic kinetic resolution of sulfoximines. *J. Am. Chem. Soc.* **138**, 2166-2169 (2016).

6. Li, W. & Lam, Y. A facile solid-phase synthesis of 1,2,4,5-tetrasubstituted imidazoles using sodium benzenesulfinate as a traceless linker. *J. Comb. Chem.* **7**, 644-647 (2005).

7. Sisko, J., Mellinger, M., Sheldrake, P.W. & Baine, N.H. α-Tosylbenzyl isocyanide: benzene, 1-[(isocyanophenylmethyl) sulfonyl]-4-methyl-. *Org. Synth.* **77**, 198-198 (2003).

8. Qi, Z., Jiang, Y., Wang, Y. & Yan, R. tert-Butyl nitrite promoted oxidative intermolecular sulfonamination of alkynes to synthesize substituted sulfonyl pyrroles from the alkynylamines and sulfinic Acids. *J. Org. Chem.* **83**, 8636-8644 (2018).

9. Yuan, Z. *et al.* Highly selective Pd-catalyzed intermolecular fluorosulfonylation of styrenes. *J. Am. Chem. Soc.* **137**, 2468-2471 (2015).

10. Zhao, C. *et al.* Enantioselective [3+3] atroposelective annulation catalyzed by *N*-heterocyclic carbenes. *Nat. Commun.* **9**, 611 (2018).

11. Jia, S. *et al.* Organocatalytic enantioselective construction of axially chiral sulfone-containing styrenes. *J. Am.

Chem. Soc. **140**, 7056-7060 (2018).
12. Sakai, H. *et al.* Formation of one-dimensional helical columns and excimerlike excited states by racemic quinoxaline-fused [7]crbohelicenes in the Crystal. *Chem. Eur. J.* **20**, 10099-10109 (2014).
13. Lee, C., Yang, W. & Parr, R.G. Development of the Colle-Salvetti correlation-energy formula into a functional of the electron density. *Phys. Rev. B* **37**, 785-789 (1988).
14. Becke, A.D. Density-functional thermochemistry. III. The role of exact exchange. *J. Chem. Phys.* **98**, 5648-5652 (1993).
15. Stephens, P.J., Devlin, F.J., Chabalowski, C.F. & Frisch, M.J. Ab initio calculation of vibrational absorption and circular dichroism spectra using density functional force fields. *J. Phys. Chem* **98**, 11623-11627 (1994).
16. Francl, M.M. *et al.* Self-consistent molecular orbital methods. XXIII. A polarization-type basis set for second-row elements. *J. Chem. Phys.* **77**, 3654-3665 (1982).
17. Hehre, W.J., Ditchfield, R. & Pople, J.A. Self—consistent molecular orbital methods. XII. Further extensions of Gaussian—type basis sets for use in molecular orbital studies of organic molecules. *J. Chem. Phys.* **56**, 2257-2261 (1972).
18. Hariharan, P.C. & Pople, J.A. The influence of polarization functions on molecular orbital hydrogenation energies. *Theor. Chim. Acta* **28**, 213-222 (1973).
19. Marenich, A.V., Cramer, C.J. & Truhlar, D.G. Universal solvation model based on solute electron density and on a continuum model of the solvent defined by the bulk dielectric constant and atomic surface tensions. *J. Phys. Chem. B* **113**, 6378-6396 (2009).
20. Zhao, Y. & Truhlar, D.G. The M06 suite of density functionals for main group thermochemistry, thermochemical kinetics, noncovalent interactions, excited states, and transition elements: two new functionals and systematic testing of four M06-class functionals and 12 other functionals. *Theor. Chem. Acc.* **120**, 215-241 (2008).
21. Zhao, Y. & Truhlar, D.G. Density functionals with broad applicability in chemistry. *Acc. Chem. Res.* **41**, 157-167 (2008).
22. Weigend, F. & Ahlrichs, R. Balanced basis sets of split valence, triple zeta valence and quadruple zeta valence quality for H to Rn: Design and assessment of accuracy. *Phys. Chem. Chem. Phys* **7**, 3297-3305 (2005).
23. Weigend, F. Accurate Coulomb-fitting basis sets for H to Rn. *Phys. Chem. Chem. Phys* **8**, 1057-1065 (2006).
24. Reist, M., Testa, B., Carrupt, P.-A., Jung, M. & Schurig, V. Racemization, enantiomerization, diastereomerization, and epimerization: Their meaning and pharmacological significance. *Chirality* **7**, 396-400 (1995).
25. Curran, D.P., Liu, W. & Chen, C.H.-T. Transfer of chirality in radical cyclizations. Cyclization of o-haloacrylanilides to oxindoles with transfer of axial chirality to a newly formed stereocenter. *J. Am. Chem. Soc.* **121**, 11012-11013 (1999).
26. Shirakawa, S., Liu, K. & Maruoka, K. Catalytic asymmetric synthesis of axially chiral *o*-iodoanilides by phase-transfer catalyzed alkylations. *J. Am. Chem. Soc.* **134**, 916-919 (2012).
27. Ma, C. *et al.* Atroposelective access to oxindole-based axially chiral styrenes via the strategy of catalytic kinetic resolution. *J. Am. Chem. Soc.* **142**, 15686-15696 (2020).
28. Wang, Q. *et al.* Rhodium-catalyzed atroposelective oxidative C–H/C–H cross-coupling reaction of 1-aryl isoquinoline derivatives with electron-rich heteroarenes. *J. Am. Chem. Soc.* **142**, 15678-15685 (2020).
29. Li, T. *et al.* *N*-Heterocyclic carbene-catalyzed atroposelective annulation for access to thiazine derivatives with C−N axial chirality. *Angew. Chem. Int. Ed.* **60**, 9362-9367 (2021).
