# Supplementary material for: Carbene-catalyzed atroposelective synthesis of axially chiral styrenes
Source: Nat Commun. 2022 Jan 10;13:84. doi: 10.1038/s41467-021-27771-x (PMC8748895; doi:10.1038/s41467-021-27771-x)
Supplement: Supplementary file 3 — Source Data [file 41467_2021_27771_MOESM3_ESM.zip › The magnitude of the vibrational frequency for the TS9a.docx]

Supplementary Data

## **The magnitude of the vibrational frequency for the TS9a**

Harmonic frequencies (cm**-1), IR intensities (KM/Mole), Raman scattering activities (A**4/AMU), depolarization ratios for plane and unpolarized incident light, reduced masses (AMU), force constants (mDyne/A), and normal coordinates:

1 2 3

A A A

Frequencies -- -26.1899 19.9677 24.6475

Red. masses -- 6.0048 4.9220 5.7817

Frc consts -- 0.0024 0.0012 0.0021

IR Inten -- 1.3605 1.1239 1.2173

Atom AN X Y Z X Y Z X Y Z

1 16 -0.02 0.01 0.08 0.01 -0.05 -0.00 0.03 -0.04 -0.04

2 6 0.01 0.02 0.04 0.03 -0.02 0.01 0.06 -0.03 -0.08

3 6 0.02 0.02 0.01 0.02 0.01 0.02 0.04 -0.03 -0.04

4 6 0.06 0.03 -0.06 -0.00 0.02 0.01 0.04 -0.03 -0.04

5 6 -0.06 -0.01 0.01 -0.01 -0.05 -0.02 -0.03 0.05 0.00

6 6 -0.07 -0.03 0.01 0.01 -0.04 -0.00 0.03 0.15 0.02

7 6 -0.08 -0.02 -0.02 -0.03 -0.05 -0.04 -0.15 0.01 0.02

8 6 -0.10 -0.05 -0.03 -0.01 -0.05 -0.02 -0.03 0.22 0.05

9 1 -0.06 -0.03 0.04 0.03 -0.04 0.02 0.13 0.18 0.00

10 6 -0.11 -0.04 -0.06 -0.05 -0.06 -0.05 -0.21 0.08 0.05

11 1 -0.06 -0.00 -0.02 -0.04 -0.06 -0.05 -0.19 -0.07 0.01

12 6 -0.12 -0.06 -0.07 -0.03 -0.05 -0.04 -0.15 0.18 0.06

13 1 -0.11 -0.07 -0.04 0.00 -0.04 -0.01 0.02 0.30 0.06

14 1 -0.12 -0.04 -0.09 -0.07 -0.06 -0.07 -0.31 0.05 0.07

15 1 -0.14 -0.07 -0.10 -0.04 -0.06 -0.05 -0.20 0.24 0.09

16 8 0.05 0.04 0.11 -0.00 -0.04 -0.01 0.02 -0.12 -0.00

17 8 -0.07 0.00 0.14 0.03 -0.07 0.01 0.06 -0.03 -0.07

18 6 0.02 0.02 0.05 0.05 -0.03 0.02 0.07 -0.03 -0.10

19 1 -0.01 0.01 0.10 0.05 -0.05 0.02 0.09 -0.03 -0.14

20 6 -0.00 -0.00 0.06 0.04 0.03 0.03 0.04 -0.03 -0.01

21 6 -0.04 0.01 0.15 0.06 0.03 0.02 0.03 -0.03 -0.01

22 6 0.01 -0.04 0.01 0.03 0.06 0.04 0.04 -0.03 0.03

23 6 0.07 -0.00 -0.10 -0.02 0.04 0.02 0.04 -0.03 -0.01

24 6 -0.06 -0.02 0.19 0.08 0.05 0.04 0.03 -0.03 0.03

25 1 -0.05 0.06 0.19 0.07 0.01 0.01 0.02 -0.03 -0.04

26 6 -0.01 -0.07 0.05 0.04 0.08 0.06 0.04 -0.03 0.07

27 6 0.05 -0.04 -0.07 0.00 0.06 0.04 0.04 -0.03 0.03

28 1 0.10 0.00 -0.16 -0.04 0.04 0.02 0.03 -0.03 -0.01

29 6 -0.05 -0.06 0.14 0.07 0.08 0.05 0.04 -0.03 0.07

30 1 -0.09 -0.00 0.26 0.09 0.05 0.03 0.02 -0.03 0.02

31 1 0.00 -0.11 0.01 0.04 0.10 0.07 0.04 -0.03 0.10

32 1 0.06 -0.07 -0.11 -0.01 0.08 0.05 0.04 -0.03 0.06

33 1 -0.07 -0.09 0.16 0.08 0.09 0.06 0.04 -0.03 0.10

34 8 0.08 0.07 -0.09 -0.02 -0.00 -0.01 0.03 -0.02 -0.07

35 6 0.13 0.08 -0.14 -0.05 0.01 -0.03 0.03 -0.02 -0.07

36 1 0.14 0.12 -0.13 -0.06 -0.01 -0.04 0.02 -0.02 -0.10

37 1 0.15 0.10 -0.15 -0.07 0.03 -0.02 0.01 -0.02 -0.06

38 1 0.13 0.04 -0.17 -0.05 0.00 -0.03 0.05 -0.03 -0.06

39 6 0.04 0.03 0.03 0.07 -0.02 0.02 0.06 -0.04 -0.06

40 8 0.10 0.05 0.04 0.06 -0.02 0.01 0.06 -0.08 -0.04

41 8 -0.01 -0.01 -0.01 0.09 -0.03 0.03 0.03 0.01 -0.05

42 6 -0.00 -0.01 -0.04 0.03 -0.03 -0.02 -0.01 0.01 0.00

43 6 -0.04 -0.02 -0.01 0.05 -0.11 -0.09 -0.04 0.02 0.04

44 6 0.03 0.00 -0.08 -0.05 0.05 0.01 -0.02 0.01 0.02

45 6 -0.04 -0.02 -0.03 -0.02 -0.11 -0.14 -0.09 0.03 0.10

46 1 -0.07 -0.03 0.02 0.11 -0.17 -0.11 -0.03 0.03 0.02

47 6 0.03 0.00 -0.10 -0.12 0.05 -0.04 -0.08 0.02 0.08

48 6 -0.01 -0.01 -0.08 -0.11 -0.03 -0.12 -0.11 0.03 0.12

49 1 -0.07 -0.03 -0.02 -0.01 -0.17 -0.20 -0.12 0.04 0.13

50 1 0.05 0.01 -0.13 -0.19 0.11 -0.03 -0.09 0.01 0.10

51 1 -0.01 -0.01 -0.09 -0.16 -0.03 -0.15 -0.15 0.03 0.17

52 8 0.06 0.01 -0.10 -0.06 0.12 0.07 0.01 0.00 -0.02

53 6 0.08 0.03 -0.13 -0.16 0.21 0.12 -0.01 -0.00 0.01

54 1 0.09 0.03 -0.12 -0.25 0.19 0.06 -0.03 -0.05 0.04

55 1 0.06 0.02 -0.15 -0.16 0.24 0.15 -0.03 0.05 0.03

56 1 0.09 0.03 -0.13 -0.15 0.25 0.17 0.02 -0.01 -0.03

4 5 6

A A A

Frequencies -- 33.7804 43.2614 48.9103

Red. masses -- 4.6705 4.1447 4.7953

Frc consts -- 0.0031 0.0046 0.0068

IR Inten -- 0.5029 0.3788 0.4442

Atom AN X Y Z X Y Z X Y Z

1 16 0.00 0.03 -0.01 -0.01 -0.01 0.00 -0.01 -0.03 -0.06

2 6 -0.01 0.02 -0.02 -0.03 -0.00 0.03 -0.02 -0.01 -0.02

3 6 -0.01 -0.00 0.00 -0.02 0.00 0.01 -0.02 0.00 -0.04

4 6 0.01 -0.01 -0.00 -0.01 0.01 -0.02 -0.02 0.01 -0.08

5 6 0.00 0.03 -0.01 0.02 -0.00 0.03 0.00 0.01 -0.01

6 6 0.03 0.04 0.01 0.17 0.04 0.17 -0.06 0.02 -0.08

7 6 -0.03 0.02 -0.04 -0.10 -0.03 -0.08 0.09 0.03 0.12

8 6 0.02 0.04 0.00 0.21 0.05 0.21 -0.03 0.07 -0.02

9 1 0.05 0.04 0.03 0.27 0.07 0.25 -0.13 0.01 -0.19

10 6 -0.03 0.02 -0.05 -0.07 -0.03 -0.05 0.12 0.08 0.19

11 1 -0.04 0.02 -0.06 -0.22 -0.06 -0.19 0.14 0.01 0.18

12 6 -0.01 0.03 -0.03 0.09 0.02 0.09 0.06 0.09 0.12

13 1 0.04 0.04 0.02 0.34 0.09 0.32 -0.08 0.08 -0.08

14 1 -0.05 0.01 -0.07 -0.16 -0.05 -0.14 0.20 0.10 0.29

15 1 -0.01 0.03 -0.03 0.12 0.03 0.12 0.09 0.13 0.17

16 8 0.02 0.03 -0.00 -0.03 -0.00 -0.01 -0.04 -0.05 -0.06

17 8 -0.01 0.05 -0.01 0.00 -0.00 -0.01 0.03 -0.03 -0.09

18 6 -0.02 0.02 -0.04 -0.03 -0.00 0.05 -0.01 -0.02 0.01

19 1 -0.01 0.04 -0.05 -0.03 -0.01 0.05 -0.01 -0.05 0.01

20 6 -0.02 -0.02 0.04 -0.02 0.00 -0.01 -0.03 0.01 -0.01

21 6 -0.05 -0.03 0.02 -0.02 0.01 0.03 -0.04 0.03 0.12

22 6 -0.01 -0.05 0.08 -0.02 -0.00 -0.07 -0.03 -0.00 -0.08

23 6 0.02 -0.03 0.04 -0.01 0.01 -0.08 -0.02 0.01 -0.17

24 6 -0.06 -0.04 0.05 -0.02 0.01 0.01 -0.04 0.02 0.17

25 1 -0.07 -0.01 -0.01 -0.01 0.02 0.07 -0.04 0.05 0.17

26 6 -0.02 -0.07 0.12 -0.03 -0.00 -0.10 -0.03 -0.01 -0.03

27 6 0.01 -0.05 0.08 -0.02 0.00 -0.11 -0.02 0.00 -0.18

28 1 0.04 -0.03 0.04 -0.01 0.01 -0.11 -0.01 0.02 -0.24

29 6 -0.05 -0.06 0.10 -0.03 -0.00 -0.06 -0.04 0.00 0.10

30 1 -0.09 -0.05 0.03 -0.01 0.01 0.04 -0.03 0.04 0.27

31 1 -0.01 -0.08 0.15 -0.03 -0.01 -0.15 -0.03 -0.02 -0.10

32 1 0.02 -0.07 0.12 -0.02 0.00 -0.16 -0.03 0.00 -0.26

33 1 -0.06 -0.08 0.13 -0.03 -0.00 -0.08 -0.04 -0.00 0.14

34 8 0.02 0.02 -0.05 -0.01 0.01 -0.00 -0.01 0.01 -0.03

35 6 0.04 0.02 -0.07 -0.01 0.04 -0.12 0.03 -0.01 0.04

36 1 0.04 0.05 -0.11 -0.01 0.05 -0.13 0.04 -0.01 0.14

37 1 0.01 0.03 -0.05 0.01 0.12 -0.14 0.10 -0.06 -0.00

38 1 0.08 0.00 -0.06 -0.03 -0.03 -0.18 -0.04 0.04 0.04

39 6 -0.03 0.02 -0.05 -0.02 -0.00 0.05 0.02 -0.01 0.04

40 8 -0.05 0.02 -0.07 -0.00 0.01 0.05 0.08 -0.00 0.07

41 8 -0.02 0.01 -0.05 -0.02 -0.01 0.05 -0.01 -0.02 0.02

42 6 0.03 0.01 -0.05 -0.01 -0.02 0.02 0.01 -0.02 0.01

43 6 0.12 0.04 -0.10 0.00 -0.03 0.01 0.02 -0.03 -0.01

44 6 0.00 -0.03 0.01 0.00 -0.01 0.01 0.00 -0.01 0.01

45 6 0.18 0.03 -0.10 0.02 -0.03 -0.03 0.03 -0.03 -0.03

46 1 0.13 0.06 -0.14 -0.00 -0.03 0.02 0.02 -0.04 -0.00

47 6 0.07 -0.03 0.01 0.02 -0.01 -0.03 0.01 -0.02 -0.01

48 6 0.16 -0.00 -0.05 0.03 -0.02 -0.04 0.03 -0.03 -0.04

49 1 0.25 0.06 -0.14 0.02 -0.04 -0.04 0.04 -0.04 -0.05

50 1 0.05 -0.06 0.06 0.02 -0.00 -0.04 0.01 -0.01 -0.02

51 1 0.21 -0.00 -0.05 0.04 -0.02 -0.07 0.04 -0.03 -0.05

52 8 -0.08 -0.06 0.06 -0.01 0.00 0.03 -0.01 -0.00 0.03

53 6 -0.18 -0.09 0.19 0.01 0.01 0.00 -0.06 0.01 0.08

54 1 -0.26 -0.09 0.13 0.02 0.04 -0.01 -0.11 0.00 0.04

55 1 -0.10 -0.10 0.30 0.01 -0.01 -0.02 -0.04 0.02 0.12

56 1 -0.27 -0.11 0.24 0.00 0.02 0.02 -0.09 0.01 0.10

7 8 9

A A A

Frequencies -- 62.1347 70.5829 82.4578

Red. masses -- 5.4358 6.7802 5.0194

Frc consts -- 0.0124 0.0199 0.0201

IR Inten -- 1.6248 0.0254 2.2961

Atom AN X Y Z X Y Z X Y Z

1 16 0.03 0.01 -0.05 -0.06 -0.02 -0.01 0.04 0.01 -0.03

2 6 -0.00 -0.00 0.02 -0.05 -0.01 -0.04 -0.01 0.01 0.03

3 6 0.01 -0.03 0.00 -0.06 -0.03 -0.00 -0.03 -0.01 0.08

4 6 0.01 -0.03 0.01 -0.04 -0.03 -0.00 -0.02 -0.02 0.10

5 6 0.02 0.03 -0.03 -0.05 -0.02 -0.00 0.06 0.02 -0.01

6 6 0.00 0.06 -0.07 -0.04 -0.04 0.02 0.06 0.03 -0.01

7 6 -0.01 0.02 -0.01 -0.01 -0.01 0.01 -0.01 -0.00 -0.05

8 6 -0.06 0.09 -0.09 0.02 -0.04 0.05 -0.02 0.03 -0.07

9 1 0.03 0.07 -0.10 -0.07 -0.05 0.01 0.12 0.05 0.02

10 6 -0.08 0.04 -0.03 0.05 -0.01 0.04 -0.09 -0.01 -0.11

11 1 0.00 -0.01 0.02 -0.01 0.00 -0.01 -0.01 -0.01 -0.04

12 6 -0.10 0.08 -0.07 0.07 -0.03 0.06 -0.11 0.00 -0.13

13 1 -0.08 0.11 -0.13 0.03 -0.06 0.07 -0.02 0.04 -0.08

14 1 -0.11 0.03 -0.01 0.09 -0.00 0.05 -0.16 -0.03 -0.14

15 1 -0.16 0.09 -0.09 0.12 -0.03 0.09 -0.18 -0.01 -0.18

16 8 -0.01 0.01 -0.08 -0.03 -0.02 0.01 -0.00 0.02 -0.06

17 8 0.08 -0.00 -0.08 -0.08 -0.00 -0.00 0.08 0.01 -0.07

18 6 -0.06 0.00 0.10 0.00 -0.01 -0.12 0.02 0.00 -0.01

19 1 -0.05 0.01 0.09 0.02 -0.03 -0.17 0.05 -0.01 -0.06

20 6 0.00 -0.04 0.01 -0.07 -0.05 0.01 -0.04 -0.02 0.05

21 6 -0.00 -0.04 -0.01 -0.10 -0.04 0.03 -0.06 -0.01 0.11

22 6 0.01 -0.04 0.03 -0.06 -0.07 0.01 -0.04 -0.04 -0.04

23 6 0.02 -0.04 0.03 -0.03 -0.06 0.01 -0.01 -0.03 0.07

24 6 -0.01 -0.05 -0.02 -0.11 -0.07 0.01 -0.07 -0.03 0.03

25 1 -0.00 -0.04 -0.02 -0.10 -0.01 0.06 -0.05 0.03 0.22

26 6 0.00 -0.05 0.03 -0.08 -0.09 -0.00 -0.06 -0.06 -0.15

27 6 0.01 -0.05 0.05 -0.04 -0.07 0.01 -0.03 -0.04 -0.02

28 1 0.02 -0.04 0.05 -0.01 -0.06 0.02 0.01 -0.04 0.10

29 6 -0.01 -0.05 0.00 -0.11 -0.09 -0.01 -0.08 -0.06 -0.12

30 1 -0.02 -0.05 -0.04 -0.13 -0.06 0.02 -0.07 -0.02 0.08

31 1 0.00 -0.05 0.05 -0.07 -0.11 -0.01 -0.05 -0.09 -0.24

32 1 0.02 -0.05 0.07 -0.03 -0.09 0.02 -0.03 -0.04 -0.08

33 1 -0.01 -0.06 0.00 -0.12 -0.11 -0.02 -0.09 -0.08 -0.20

34 8 0.01 -0.02 -0.01 -0.03 -0.01 -0.03 -0.01 -0.02 0.14

35 6 0.01 -0.03 -0.01 -0.00 -0.01 -0.02 -0.00 -0.03 0.16

36 1 0.01 -0.02 -0.03 0.00 0.01 -0.04 0.00 -0.02 0.19

37 1 -0.00 -0.03 0.00 -0.02 -0.02 -0.01 0.02 -0.04 0.15

38 1 0.04 -0.03 -0.00 0.02 -0.02 -0.02 -0.02 -0.02 0.16

39 6 -0.09 -0.01 0.13 0.05 0.00 -0.11 0.06 0.02 0.02

40 8 -0.15 -0.02 0.11 -0.05 -0.02 -0.16 0.15 0.03 0.06

41 8 -0.03 0.00 0.16 0.22 0.04 -0.01 0.02 0.01 -0.00

42 6 -0.02 0.00 0.11 0.16 0.05 0.02 0.03 0.01 -0.01

43 6 0.01 -0.04 0.05 0.14 0.01 0.01 -0.02 0.01 0.03

44 6 -0.01 0.03 0.06 0.09 0.10 0.05 0.06 0.01 -0.05

45 6 0.07 -0.05 -0.08 0.05 0.02 0.02 -0.03 0.02 0.04

46 1 0.00 -0.07 0.08 0.19 -0.02 -0.01 -0.05 0.01 0.07

47 6 0.06 0.02 -0.07 0.01 0.11 0.06 0.05 0.02 -0.04

48 6 0.10 -0.02 -0.14 -0.01 0.07 0.05 0.01 0.02 -0.00

49 1 0.10 -0.09 -0.13 0.03 -0.02 0.01 -0.07 0.02 0.07

50 1 0.09 0.05 -0.12 -0.05 0.15 0.08 0.08 0.02 -0.07

51 1 0.16 -0.03 -0.24 -0.08 0.07 0.07 -0.00 0.02 -0.00

52 8 -0.05 0.08 0.14 0.13 0.16 0.08 0.10 0.02 -0.08

53 6 0.16 0.11 -0.09 0.15 0.23 0.00 -0.09 0.04 0.11

54 1 0.33 0.27 -0.10 0.16 0.23 0.02 -0.26 -0.04 0.06

55 1 0.13 -0.04 -0.29 0.09 0.25 -0.06 -0.05 0.13 0.27

56 1 0.16 0.15 -0.05 0.22 0.27 0.00 -0.12 0.05 0.14

10 11 12

A A A

Frequencies -- 92.3584 98.1120 103.6291

Red. masses -- 3.9077 4.5263 6.5787

Frc consts -- 0.0196 0.0257 0.0416

IR Inten -- 0.2835 3.1214 1.1427

Atom AN X Y Z X Y Z X Y Z

1 16 0.01 0.02 0.01 -0.02 0.03 0.00 -0.03 -0.05 0.00

2 6 -0.00 -0.01 -0.02 -0.02 -0.00 -0.01 0.02 -0.02 -0.08

3 6 0.01 -0.03 -0.04 0.02 -0.03 -0.07 0.02 -0.03 -0.04

4 6 0.00 -0.03 -0.02 0.01 -0.03 -0.07 0.01 -0.03 -0.02

5 6 0.02 0.01 0.01 -0.01 0.02 -0.00 -0.07 -0.05 -0.01

6 6 0.05 0.01 0.05 0.00 0.01 0.02 -0.05 -0.06 0.02

7 6 -0.03 -0.00 -0.05 -0.02 0.01 -0.02 -0.04 -0.04 -0.00

8 6 0.03 -0.02 0.01 0.02 0.01 0.02 0.01 -0.06 0.06

9 1 0.10 0.02 0.10 0.01 0.01 0.03 -0.07 -0.07 0.02

10 6 -0.06 -0.03 -0.10 -0.01 0.01 -0.02 0.02 -0.04 0.03

11 1 -0.05 0.01 -0.07 -0.03 0.02 -0.04 -0.06 -0.04 -0.02

12 6 -0.03 -0.03 -0.07 0.01 0.00 -0.00 0.05 -0.05 0.07

13 1 0.05 -0.02 0.04 0.03 0.00 0.04 0.03 -0.07 0.08

14 1 -0.10 -0.04 -0.15 -0.02 0.01 -0.04 0.04 -0.04 0.04

15 1 -0.06 -0.05 -0.10 0.02 0.00 0.00 0.10 -0.05 0.10

16 8 0.02 0.02 0.02 -0.01 0.04 0.00 0.00 -0.10 0.05

17 8 -0.02 0.05 0.01 -0.03 0.03 0.02 -0.07 -0.05 0.03

18 6 -0.01 0.00 -0.05 -0.07 0.01 0.05 0.05 -0.02 -0.13

19 1 -0.01 0.02 -0.05 -0.09 0.02 0.09 0.06 -0.03 -0.15

20 6 0.01 -0.02 -0.04 0.02 -0.03 -0.07 0.02 -0.03 -0.03

21 6 0.02 -0.01 0.02 0.04 -0.03 -0.08 0.03 -0.03 -0.03

22 6 0.01 -0.00 -0.06 0.02 -0.01 -0.01 0.02 -0.02 -0.00

23 6 -0.01 -0.00 -0.11 -0.00 -0.01 -0.08 0.00 -0.03 0.03

24 6 0.04 0.01 0.12 0.06 -0.01 0.01 0.04 -0.02 -0.03

25 1 0.02 -0.02 -0.01 0.04 -0.06 -0.15 0.04 -0.03 -0.03

26 6 0.02 0.01 0.03 0.04 0.02 0.09 0.03 -0.01 -0.00

27 6 -0.01 0.00 -0.13 0.01 -0.00 -0.04 0.01 -0.02 0.04

28 1 -0.02 0.00 -0.15 -0.02 -0.00 -0.10 -0.01 -0.03 0.06

29 6 0.04 0.02 0.14 0.06 0.02 0.12 0.03 -0.02 -0.03

30 1 0.05 0.02 0.18 0.06 -0.01 0.01 0.04 -0.02 -0.04

31 1 0.01 0.02 0.03 0.03 0.04 0.16 0.02 -0.00 0.01

32 1 -0.01 0.01 -0.19 0.01 0.00 -0.02 0.01 -0.02 0.08

33 1 0.05 0.03 0.23 0.08 0.04 0.21 0.04 -0.01 -0.03

34 8 -0.00 -0.07 0.07 -0.00 -0.06 -0.04 -0.00 -0.04 -0.06

35 6 -0.02 -0.11 0.24 -0.03 -0.08 0.06 -0.03 -0.06 0.03

36 1 -0.02 -0.17 0.38 -0.04 -0.13 0.11 -0.04 -0.08 0.00

37 1 0.06 -0.21 0.20 -0.01 -0.14 0.05 -0.07 -0.12 0.06

38 1 -0.12 0.01 0.28 -0.07 -0.01 0.09 0.01 -0.01 0.08

39 6 -0.01 0.00 -0.05 -0.08 0.01 0.02 0.04 0.00 0.00

40 8 -0.02 -0.01 -0.04 -0.16 0.02 -0.04 0.16 -0.11 0.15

41 8 -0.01 0.02 -0.04 0.04 -0.01 0.06 -0.15 0.13 -0.01

42 6 -0.01 0.03 -0.02 0.03 -0.01 0.03 -0.11 0.14 0.05

43 6 0.04 0.04 -0.05 -0.07 -0.02 0.11 -0.10 0.18 0.07

44 6 -0.05 0.02 0.04 0.11 0.00 -0.07 -0.02 0.10 -0.01

45 6 0.05 0.04 -0.04 -0.09 -0.02 0.08 0.03 0.17 -0.01

46 1 0.08 0.04 -0.10 -0.14 -0.03 0.19 -0.17 0.22 0.11

47 6 -0.05 0.02 0.06 0.11 -0.00 -0.11 0.12 0.09 -0.11

48 6 0.00 0.03 0.02 0.02 -0.01 -0.03 0.16 0.13 -0.11

49 1 0.10 0.04 -0.07 -0.17 -0.03 0.15 0.05 0.19 -0.01

50 1 -0.08 0.01 0.10 0.19 0.01 -0.20 0.22 0.05 -0.18

51 1 0.01 0.03 0.03 0.02 -0.01 -0.06 0.29 0.11 -0.19

52 8 -0.09 0.01 0.06 0.17 0.02 -0.11 -0.06 0.09 0.01

53 6 0.06 -0.01 -0.08 -0.09 0.07 0.13 -0.06 0.07 0.02

54 1 0.19 0.04 -0.03 -0.32 -0.01 0.03 -0.05 0.16 -0.05

55 1 0.02 -0.08 -0.20 -0.02 0.17 0.33 0.02 -0.02 0.03

56 1 0.09 -0.02 -0.11 -0.14 0.09 0.20 -0.16 0.07 0.10

13 14 15

A A A

Frequencies -- 120.0566 130.8565 161.6075

Red. masses -- 3.7798 6.6180 7.1046

Frc consts -- 0.0321 0.0668 0.1093

IR Inten -- 4.6226 2.8530 4.1945

Atom AN X Y Z X Y Z X Y Z

1 16 -0.02 -0.03 -0.03 -0.07 0.08 -0.01 -0.02 -0.00 -0.04

2 6 0.01 0.02 -0.02 -0.03 0.02 -0.00 0.02 -0.01 -0.03

3 6 0.01 0.07 -0.04 0.02 -0.07 -0.05 0.00 0.01 0.02

4 6 0.03 0.07 -0.06 0.02 -0.08 -0.04 0.00 -0.00 0.10

5 6 -0.05 -0.03 -0.05 -0.08 0.04 -0.07 -0.07 -0.08 -0.20

6 6 0.00 -0.01 -0.01 -0.06 0.04 -0.05 -0.02 0.01 -0.19

7 6 -0.06 -0.03 -0.05 -0.05 0.06 -0.07 -0.09 -0.08 -0.12

8 6 0.05 0.01 0.04 0.02 0.05 0.01 0.06 0.12 -0.05

9 1 0.02 -0.01 0.00 -0.08 0.03 -0.06 0.00 0.02 -0.26

10 6 -0.03 -0.02 -0.02 0.02 0.06 -0.02 -0.02 0.02 0.02

11 1 -0.09 -0.05 -0.07 -0.07 0.06 -0.10 -0.13 -0.15 -0.13

12 6 0.03 0.00 0.03 0.07 0.06 0.03 0.08 0.12 0.08

13 1 0.09 0.02 0.08 0.05 0.04 0.04 0.12 0.19 -0.02

14 1 -0.04 -0.02 -0.02 0.05 0.07 -0.01 -0.02 0.02 0.11

15 1 0.07 0.02 0.07 0.14 0.07 0.08 0.17 0.20 0.21

16 8 0.00 -0.03 -0.02 -0.05 0.17 -0.06 0.16 0.22 -0.07

17 8 -0.03 -0.06 0.00 -0.10 0.01 0.07 -0.20 -0.09 0.20

18 6 0.01 0.00 0.04 -0.09 0.03 0.10 0.07 -0.03 -0.07

19 1 -0.01 -0.01 0.08 -0.15 -0.01 0.22 0.09 -0.03 -0.11

20 6 -0.01 0.06 -0.01 0.02 -0.08 -0.03 0.01 0.02 -0.01

21 6 -0.05 0.06 -0.03 0.06 -0.07 0.01 0.01 0.02 0.04

22 6 0.01 0.02 0.03 0.02 -0.06 -0.00 0.01 0.01 -0.05

23 6 0.04 0.03 0.02 0.00 -0.06 0.06 0.01 -0.00 0.08

24 6 -0.08 0.02 -0.06 0.07 -0.06 0.00 0.01 0.03 0.08

25 1 -0.06 0.09 -0.02 0.08 -0.09 0.03 0.00 0.03 0.05

26 6 -0.02 -0.02 0.01 0.04 -0.04 -0.02 0.00 0.01 -0.05

27 6 0.04 0.01 0.07 0.01 -0.06 0.07 0.01 0.01 -0.02

28 1 0.06 0.03 0.03 -0.01 -0.07 0.12 0.02 -0.01 0.15

29 6 -0.06 -0.02 -0.05 0.06 -0.04 -0.03 0.01 0.02 0.03

30 1 -0.11 0.02 -0.09 0.09 -0.05 0.02 0.02 0.04 0.14

31 1 -0.00 -0.04 0.04 0.03 -0.02 -0.02 0.00 0.01 -0.09

32 1 0.05 -0.01 0.13 0.01 -0.06 0.14 0.00 0.02 -0.04

33 1 -0.08 -0.05 -0.08 0.07 -0.03 -0.05 0.01 0.02 0.05

34 8 0.05 0.14 -0.17 -0.03 -0.11 -0.14 0.03 -0.01 0.20

35 6 0.12 0.02 0.26 -0.13 -0.14 -0.01 0.04 0.04 0.02

36 1 0.14 0.04 0.30 -0.17 -0.20 -0.12 0.05 0.07 -0.01

37 1 0.08 -0.30 0.33 -0.25 -0.20 0.08 0.06 0.16 -0.01

38 1 0.21 0.24 0.47 -0.04 -0.05 0.10 0.03 -0.08 -0.07

39 6 0.01 -0.00 0.03 0.04 0.06 0.08 0.03 -0.05 -0.04

40 8 -0.00 0.01 0.02 0.24 0.16 0.14 -0.05 -0.11 -0.05

41 8 0.01 -0.02 0.02 -0.02 -0.02 0.01 0.04 -0.01 -0.00

42 6 0.01 -0.03 0.01 0.04 -0.02 -0.02 -0.01 -0.02 0.02

43 6 0.00 -0.04 0.00 0.08 -0.03 -0.06 -0.05 -0.03 0.05

44 6 0.00 -0.02 0.01 -0.00 -0.01 0.02 -0.00 -0.02 0.01

45 6 -0.01 -0.04 0.00 0.04 -0.03 -0.03 -0.04 -0.03 0.03

46 1 0.01 -0.05 0.01 0.12 -0.04 -0.10 -0.07 -0.04 0.08

47 6 -0.01 -0.02 0.00 -0.08 -0.01 0.08 0.04 -0.02 -0.05

48 6 -0.01 -0.03 0.00 -0.06 -0.01 0.06 0.02 -0.03 -0.04

49 1 -0.01 -0.04 -0.00 0.07 -0.03 -0.06 -0.07 -0.04 0.05

50 1 -0.01 -0.01 0.00 -0.14 0.01 0.14 0.07 -0.02 -0.09

51 1 -0.02 -0.03 -0.00 -0.12 -0.01 0.11 0.05 -0.04 -0.08

52 8 0.00 -0.02 0.01 0.03 -0.01 -0.01 -0.05 -0.01 0.05

53 6 0.01 -0.01 0.00 0.03 0.00 -0.01 -0.00 -0.02 0.01

54 1 0.01 -0.02 0.01 0.01 -0.08 0.05 0.05 0.08 -0.05

55 1 0.00 -0.01 -0.00 -0.04 0.09 -0.01 0.06 -0.14 -0.03

56 1 0.01 -0.01 0.00 0.11 0.00 -0.08 -0.08 -0.02 0.08

16 17 18

A A A

Frequencies -- 169.6230 174.4997 188.8345

Red. masses -- 5.7494 5.3156 4.3027

Frc consts -- 0.0975 0.0954 0.0904

IR Inten -- 0.1799 3.2175 0.1586

Atom AN X Y Z X Y Z X Y Z

1 16 -0.05 -0.06 -0.05 0.01 0.02 -0.00 -0.07 0.03 -0.03

2 6 -0.02 0.04 0.06 -0.04 -0.02 -0.07 -0.02 0.02 -0.06

3 6 0.02 0.01 0.01 -0.05 0.01 -0.07 -0.00 0.01 -0.06

4 6 0.04 -0.01 0.06 -0.05 0.01 -0.03 0.01 -0.01 0.04

5 6 -0.06 -0.08 -0.03 0.04 0.01 0.01 -0.08 0.09 0.04

6 6 -0.04 -0.06 -0.02 0.05 0.02 0.00 -0.08 0.02 0.07

7 6 -0.07 -0.09 0.00 0.04 0.01 0.00 -0.03 0.11 -0.03

8 6 -0.01 -0.03 0.03 0.01 0.02 -0.02 -0.02 -0.05 0.04

9 1 -0.04 -0.06 -0.04 0.07 0.03 0.01 -0.13 0.00 0.12

10 6 -0.05 -0.06 0.05 0.01 0.01 -0.02 0.03 0.04 -0.06

11 1 -0.09 -0.12 0.00 0.05 0.02 0.01 -0.03 0.18 -0.06

12 6 -0.02 -0.03 0.07 -0.01 0.01 -0.04 0.03 -0.04 -0.03

13 1 0.01 0.00 0.04 0.01 0.02 -0.03 -0.03 -0.12 0.07

14 1 -0.06 -0.06 0.07 -0.01 0.01 -0.02 0.07 0.05 -0.12

15 1 0.01 -0.01 0.12 -0.04 0.01 -0.06 0.07 -0.09 -0.05

16 8 -0.13 0.00 -0.14 0.10 0.02 0.05 -0.12 -0.06 0.00

17 8 0.04 -0.15 -0.06 -0.08 0.09 0.02 -0.01 0.06 -0.11

18 6 -0.05 0.05 0.12 -0.02 -0.04 -0.06 -0.01 0.00 -0.03

19 1 -0.08 0.03 0.18 -0.02 -0.06 -0.06 -0.02 -0.02 -0.02

20 6 0.02 -0.01 -0.04 -0.05 0.02 -0.09 0.01 -0.00 -0.09

21 6 0.03 -0.02 -0.09 -0.07 0.01 -0.21 0.03 0.01 -0.04

22 6 0.03 -0.02 -0.00 -0.04 0.01 0.04 0.02 -0.01 -0.07

23 6 0.05 -0.02 -0.01 -0.04 0.01 -0.11 0.02 -0.04 0.11

24 6 0.03 -0.02 -0.05 -0.09 -0.02 -0.19 0.04 0.02 0.05

25 1 0.03 -0.04 -0.14 -0.07 0.01 -0.26 0.03 0.00 -0.04

26 6 0.03 -0.02 0.07 -0.04 0.00 0.19 0.02 0.01 -0.03

27 6 0.04 -0.02 -0.03 -0.04 0.01 -0.02 0.02 -0.01 0.03

28 1 0.07 -0.02 -0.02 -0.04 0.02 -0.17 0.04 -0.05 0.22

29 6 0.04 -0.02 0.07 -0.07 -0.01 0.06 0.04 0.01 0.06

30 1 0.01 -0.02 -0.08 -0.14 -0.03 -0.32 0.04 0.03 0.13

31 1 0.03 -0.01 0.14 -0.03 -0.00 0.38 0.02 0.02 -0.03

32 1 0.04 -0.03 -0.05 -0.04 0.01 -0.01 0.02 -0.01 0.08

33 1 0.04 -0.01 0.14 -0.07 -0.01 0.15 0.05 0.02 0.13

34 8 0.06 -0.00 0.22 -0.01 0.01 0.14 0.03 0.01 0.09

35 6 0.18 0.05 0.00 0.06 0.05 -0.01 0.15 0.04 -0.01

36 1 0.23 0.13 0.12 0.09 0.06 0.17 0.17 0.15 -0.07

37 1 0.33 0.16 -0.12 0.24 0.14 -0.15 0.13 0.06 -0.00

38 1 0.07 -0.10 -0.16 -0.12 -0.03 -0.16 0.22 -0.07 -0.05

39 6 -0.04 0.08 0.04 0.03 -0.04 0.03 0.04 0.00 -0.03

40 8 -0.03 0.13 0.01 0.19 -0.06 0.15 0.09 0.04 -0.02

41 8 -0.05 0.04 -0.00 -0.04 -0.03 0.02 0.06 -0.03 -0.03

42 6 -0.00 0.05 -0.04 0.01 -0.04 0.03 0.03 -0.04 0.00

43 6 0.05 0.08 -0.07 0.03 -0.05 0.01 -0.05 -0.06 0.06

44 6 -0.00 0.04 -0.03 0.01 -0.03 0.03 0.01 -0.03 0.01

45 6 0.06 0.08 -0.04 0.02 -0.06 -0.02 -0.08 -0.06 0.06

46 1 0.07 0.10 -0.11 0.05 -0.07 0.00 -0.07 -0.07 0.10

47 6 -0.04 0.05 0.05 -0.02 -0.03 0.02 0.05 -0.04 -0.06

48 6 -0.01 0.07 0.05 -0.01 -0.04 0.01 -0.00 -0.06 -0.02

49 1 0.10 0.09 -0.07 0.04 -0.07 -0.04 -0.15 -0.06 0.12

50 1 -0.08 0.03 0.11 -0.04 -0.01 0.03 0.09 -0.03 -0.11

51 1 -0.04 0.08 0.10 -0.02 -0.04 0.00 0.01 -0.06 -0.06

52 8 0.05 0.02 -0.09 0.06 -0.00 0.01 -0.08 -0.01 0.12

53 6 -0.01 0.01 -0.02 0.02 0.05 0.02 0.01 -0.01 0.02

54 1 -0.08 -0.15 0.07 -0.02 -0.01 0.04 0.13 0.25 -0.14

55 1 -0.09 0.17 0.04 -0.03 0.13 0.02 0.15 -0.29 -0.07

56 1 0.09 -0.01 -0.14 0.10 0.07 -0.01 -0.18 0.01 0.22

19 20 21

A A A

Frequencies -- 192.1480 201.1567 212.3860

Red. masses -- 3.5348 4.3940 4.1528

Frc consts -- 0.0769 0.1048 0.1104

IR Inten -- 0.9866 3.5780 6.4975

Atom AN X Y Z X Y Z X Y Z

1 16 -0.00 -0.00 -0.01 -0.05 0.04 -0.01 0.01 -0.02 -0.01

2 6 -0.01 -0.01 -0.08 -0.02 -0.03 0.04 -0.09 -0.04 0.00

3 6 -0.01 0.01 -0.07 -0.04 -0.06 0.16 -0.08 -0.05 0.01

4 6 -0.01 -0.01 0.03 -0.03 -0.03 0.03 -0.07 -0.04 -0.00

5 6 -0.02 0.02 -0.00 -0.07 0.07 -0.02 0.12 -0.09 0.02

6 6 -0.02 0.01 0.00 -0.07 0.03 0.01 0.12 -0.03 -0.00

7 6 -0.00 0.03 -0.02 -0.03 0.09 -0.06 0.07 -0.11 0.08

8 6 0.00 -0.01 -0.00 -0.01 -0.00 0.01 0.02 0.03 -0.02

9 1 -0.03 0.00 0.01 -0.10 0.02 0.03 0.19 -0.01 -0.03

10 6 0.02 0.01 -0.02 0.03 0.05 -0.06 -0.04 -0.06 0.07

11 1 -0.01 0.05 -0.03 -0.04 0.13 -0.08 0.09 -0.17 0.13

12 6 0.02 -0.00 -0.01 0.05 0.01 -0.02 -0.09 -0.00 -0.00

13 1 0.00 -0.03 0.01 0.00 -0.05 0.04 0.01 0.09 -0.06

14 1 0.03 0.02 -0.04 0.06 0.06 -0.09 -0.10 -0.08 0.11

15 1 0.03 -0.02 -0.02 0.10 -0.01 -0.01 -0.19 0.03 -0.04

16 8 0.06 -0.09 0.08 -0.07 0.03 -0.02 0.06 0.06 -0.03

17 8 -0.05 0.06 -0.03 -0.02 0.02 -0.02 -0.07 0.06 -0.00

18 6 -0.03 -0.01 -0.04 0.03 -0.03 -0.09 -0.12 -0.02 -0.01

19 1 -0.02 -0.01 -0.06 0.10 -0.01 -0.23 -0.13 -0.03 0.02

20 6 -0.00 0.01 -0.08 -0.01 -0.05 0.18 -0.05 -0.05 -0.00

21 6 0.01 0.02 0.01 0.07 -0.08 0.10 0.04 -0.06 0.00

22 6 -0.00 0.01 -0.10 -0.03 -0.01 0.12 -0.07 -0.01 -0.02

23 6 0.00 -0.03 0.15 -0.04 -0.01 -0.11 -0.08 -0.05 0.05

24 6 0.02 0.04 0.12 0.12 -0.03 -0.06 0.11 0.02 0.03

25 1 0.00 0.03 0.02 0.10 -0.16 0.07 0.07 -0.14 -0.03

26 6 0.00 0.02 -0.10 0.02 0.05 0.04 -0.02 0.08 -0.03

27 6 -0.00 0.00 0.03 -0.04 -0.01 -0.03 -0.08 -0.02 0.02

28 1 0.01 -0.05 0.30 -0.05 0.01 -0.29 -0.08 -0.05 0.07

29 6 0.01 0.03 0.05 0.09 0.04 -0.08 0.08 0.08 0.01

30 1 0.03 0.06 0.24 0.18 -0.05 -0.17 0.17 0.01 0.05

31 1 -0.00 0.02 -0.14 -0.01 0.11 0.00 -0.05 0.15 -0.05

32 1 -0.00 0.00 0.10 -0.05 -0.00 -0.15 -0.08 -0.01 0.04

33 1 0.02 0.04 0.11 0.12 0.07 -0.20 0.12 0.13 0.03

34 8 0.01 0.02 0.02 0.00 0.05 -0.03 -0.01 0.07 -0.10

35 6 0.05 0.02 -0.01 0.16 0.02 0.03 0.17 0.03 -0.01

36 1 0.04 0.10 -0.18 0.22 0.10 0.20 0.20 0.20 -0.16

37 1 -0.09 0.01 0.08 0.31 -0.08 -0.06 0.04 -0.13 0.10

38 1 0.22 -0.03 0.04 0.09 0.03 -0.01 0.42 -0.00 0.10

39 6 -0.06 -0.03 0.04 -0.00 -0.05 -0.01 -0.04 0.01 -0.03

40 8 -0.08 -0.09 0.08 -0.03 -0.14 0.03 0.05 0.07 -0.02

41 8 -0.06 0.01 0.06 -0.01 0.01 0.03 -0.03 -0.02 -0.05

42 6 -0.02 0.00 0.03 -0.02 0.00 0.02 0.01 -0.01 -0.02

43 6 0.05 -0.01 -0.04 0.01 -0.01 -0.01 0.00 0.02 0.00

44 6 -0.00 0.00 0.01 -0.00 0.00 0.01 0.01 -0.02 -0.01

45 6 0.08 -0.02 -0.08 0.02 -0.02 -0.03 -0.01 0.02 0.03

46 1 0.08 -0.02 -0.07 0.02 -0.02 -0.01 -0.01 0.04 -0.00

47 6 -0.06 0.01 0.06 -0.02 0.00 0.01 0.02 -0.02 -0.00

48 6 -0.02 -0.00 0.01 -0.00 -0.01 -0.01 0.01 0.01 0.03

49 1 0.14 -0.03 -0.14 0.05 -0.03 -0.06 -0.02 0.04 0.06

50 1 -0.11 0.02 0.10 -0.03 0.01 0.02 0.03 -0.03 -0.01

51 1 -0.04 0.00 0.03 -0.00 -0.01 -0.02 0.02 0.01 0.05

52 8 0.11 -0.01 -0.10 0.04 -0.01 -0.04 -0.01 0.01 0.03

53 6 0.00 0.01 -0.00 0.00 -0.01 0.00 -0.00 0.06 -0.01

54 1 -0.13 -0.29 0.19 -0.05 -0.14 0.09 0.01 0.17 -0.10

55 1 -0.17 0.33 0.09 -0.07 0.12 0.04 0.04 -0.04 -0.05

56 1 0.24 -0.01 -0.23 0.10 -0.03 -0.10 -0.07 0.10 0.10

22 23 24

A A A

Frequencies -- 235.2379 243.8793 251.3810

Red. masses -- 5.2936 3.8668 1.4645

Frc consts -- 0.1726 0.1355 0.0545

IR Inten -- 2.4936 1.2254 1.7111

Atom AN X Y Z X Y Z X Y Z

1 16 -0.05 0.05 0.01 0.00 0.01 0.01 -0.00 0.01 0.00

2 6 0.01 -0.01 0.04 0.03 -0.02 0.02 0.01 -0.01 0.01

3 6 0.02 0.02 -0.02 0.02 0.01 0.01 0.00 0.00 0.01

4 6 0.02 0.01 -0.01 0.02 0.01 0.01 0.00 0.00 0.00

5 6 0.06 -0.00 0.10 -0.02 0.02 0.00 -0.00 0.01 0.01

6 6 0.06 -0.01 0.12 -0.03 0.01 0.01 -0.00 0.00 0.01

7 6 0.06 -0.00 0.12 -0.01 0.03 -0.01 0.00 0.01 0.01

8 6 -0.03 -0.03 0.05 -0.01 -0.00 0.01 -0.00 -0.00 0.01

9 1 0.09 -0.00 0.15 -0.04 0.01 0.01 -0.00 0.00 0.02

10 6 -0.02 -0.02 0.06 0.01 0.02 -0.02 0.00 0.00 0.00

11 1 0.09 -0.01 0.15 -0.02 0.05 -0.03 0.00 0.01 0.00

12 6 -0.10 -0.05 -0.01 0.02 0.00 -0.00 -0.00 -0.00 -0.00

13 1 -0.05 -0.03 0.02 -0.01 -0.02 0.01 -0.01 -0.01 0.01

14 1 -0.04 -0.03 0.04 0.02 0.02 -0.03 0.00 0.00 -0.00

15 1 -0.20 -0.08 -0.10 0.04 -0.01 0.00 -0.01 -0.01 -0.01

16 8 -0.16 0.26 -0.19 -0.04 0.03 -0.02 -0.02 0.03 -0.02

17 8 0.02 -0.04 0.03 0.04 -0.03 0.01 0.01 -0.01 0.01

18 6 0.10 -0.03 -0.07 0.02 -0.02 0.01 0.02 -0.01 -0.01

19 1 0.10 -0.04 -0.07 0.01 0.02 0.03 0.03 0.00 -0.03

20 6 0.02 0.02 -0.08 0.02 0.02 0.01 0.00 0.00 -0.01

21 6 -0.02 0.03 -0.07 -0.00 0.02 0.01 -0.00 0.01 -0.01

22 6 0.03 0.00 -0.07 0.02 0.01 -0.00 0.01 0.00 -0.01

23 6 0.03 0.00 0.03 0.02 0.01 0.00 0.01 0.00 0.00

24 6 -0.04 0.01 0.01 -0.01 0.01 0.02 -0.01 0.00 -0.00

25 1 -0.03 0.07 -0.06 -0.02 0.04 -0.01 -0.01 0.01 -0.01

26 6 0.01 -0.02 -0.01 0.01 -0.00 -0.02 0.00 -0.00 -0.00

27 6 0.03 0.00 -0.01 0.02 0.01 -0.00 0.01 0.00 -0.01

28 1 0.04 -0.01 0.09 0.02 0.01 0.01 0.01 0.00 0.01

29 6 -0.02 -0.02 0.05 -0.01 -0.00 -0.00 -0.01 -0.00 0.01

30 1 -0.07 0.02 0.04 -0.02 0.01 0.02 -0.01 0.00 -0.00

31 1 0.02 -0.05 0.02 0.02 -0.02 -0.04 0.00 -0.01 0.00

32 1 0.03 0.00 0.03 0.02 0.02 -0.01 0.00 0.00 -0.02

33 1 -0.03 -0.03 0.13 -0.02 -0.02 -0.01 -0.01 -0.01 0.03

34 8 0.01 0.01 -0.05 0.01 0.00 0.00 0.00 0.00 -0.00

35 6 -0.01 0.00 -0.01 -0.02 0.01 0.00 -0.01 0.00 0.00

36 1 -0.03 0.05 -0.24 -0.03 -0.01 -0.01 -0.02 0.02 -0.08

37 1 -0.21 -0.05 0.14 -0.03 0.02 0.01 -0.07 -0.01 0.05

38 1 0.21 -0.00 0.11 -0.02 0.01 0.01 0.06 -0.00 0.04

39 6 0.07 -0.07 -0.04 -0.04 -0.05 -0.05 -0.01 -0.02 0.02

40 8 0.02 -0.18 0.01 0.00 -0.03 -0.04 -0.03 -0.07 0.05

41 8 0.04 0.01 0.01 -0.16 -0.07 -0.12 -0.04 0.00 0.02

42 6 -0.03 0.01 0.04 -0.03 -0.07 -0.03 0.01 -0.00 -0.02

43 6 -0.03 -0.01 0.03 -0.01 0.03 0.01 0.05 0.01 -0.06

44 6 -0.02 0.02 0.03 -0.01 -0.14 0.00 0.02 -0.00 -0.03

45 6 0.00 -0.02 -0.03 0.05 0.03 0.06 0.02 0.01 -0.01

46 1 -0.03 -0.03 0.04 -0.05 0.09 -0.00 0.08 0.01 -0.09

47 6 -0.01 0.02 -0.00 0.03 -0.14 0.04 -0.02 0.00 0.02

48 6 0.02 -0.01 -0.05 0.09 -0.05 0.08 -0.05 0.02 0.06

49 1 0.02 -0.03 -0.06 0.07 0.08 0.09 0.04 0.01 -0.02

50 1 -0.01 0.03 -0.01 0.04 -0.18 0.07 -0.02 -0.00 0.03

51 1 0.05 -0.01 -0.10 0.15 -0.05 0.14 -0.09 0.02 0.11

52 8 0.00 0.00 -0.01 0.04 -0.04 0.06 0.05 -0.00 -0.05

53 6 0.01 -0.05 0.01 -0.05 0.28 -0.06 -0.00 0.02 -0.01

54 1 -0.02 -0.28 0.22 -0.17 0.21 -0.09 0.03 0.41 -0.38

55 1 -0.14 0.19 0.05 -0.24 0.43 -0.18 0.32 -0.40 -0.00

56 1 0.20 -0.10 -0.22 0.21 0.47 -0.02 -0.41 0.06 0.39

25 26 27

A A A

Frequencies -- 259.3382 286.3077 306.5693

Red. masses -- 1.5276 3.5624 5.3049

Frc consts -- 0.0605 0.1721 0.2938

IR Inten -- 0.4102 2.6174 4.3485

Atom AN X Y Z X Y Z X Y Z

1 16 -0.02 0.01 -0.02 -0.03 0.02 -0.07 0.09 0.07 -0.01

2 6 -0.00 0.00 -0.04 0.01 0.02 -0.07 0.00 0.02 0.03

3 6 0.01 0.01 -0.06 0.04 0.02 -0.03 -0.02 0.00 0.10

4 6 0.02 0.00 -0.04 0.06 0.02 -0.02 -0.00 0.02 0.07

5 6 0.02 -0.02 0.02 -0.00 -0.04 0.01 -0.04 -0.03 -0.00

6 6 0.03 -0.01 0.03 -0.00 -0.06 0.02 -0.14 -0.11 -0.04

7 6 0.02 -0.02 0.04 -0.03 -0.05 0.04 -0.15 -0.07 -0.01

8 6 -0.00 -0.01 0.01 -0.02 -0.05 0.03 -0.06 -0.09 0.05

9 1 0.04 -0.01 0.03 -0.01 -0.06 0.03 -0.24 -0.13 -0.07

10 6 -0.01 -0.02 0.02 -0.04 -0.05 0.06 -0.10 -0.07 0.05

11 1 0.03 -0.03 0.06 -0.04 -0.09 0.05 -0.19 -0.13 -0.03

12 6 -0.03 -0.02 0.00 -0.04 -0.05 0.05 0.03 -0.06 0.18

13 1 -0.00 0.00 0.01 -0.02 -0.02 0.02 -0.05 -0.06 0.05

14 1 -0.02 -0.02 0.03 -0.04 -0.05 0.06 -0.10 -0.07 0.01

15 1 -0.07 -0.02 -0.02 -0.05 -0.05 0.05 0.16 -0.03 0.29

16 8 -0.01 0.03 -0.03 0.05 0.01 -0.02 0.12 0.11 -0.02

17 8 -0.05 0.04 -0.02 -0.11 0.15 -0.11 0.15 0.15 -0.14

18 6 -0.02 -0.00 -0.02 -0.03 0.03 -0.05 0.01 0.03 -0.07

19 1 -0.03 -0.00 0.01 -0.01 0.03 -0.08 0.08 0.07 -0.19

20 6 0.01 0.01 -0.00 0.04 0.03 0.09 -0.02 -0.03 -0.01

21 6 0.01 0.02 0.05 0.02 0.04 0.11 0.01 -0.05 -0.08

22 6 0.02 0.01 0.03 0.06 0.02 0.10 -0.03 -0.03 -0.08

23 6 0.02 0.00 0.02 0.06 0.04 -0.09 0.01 -0.02 0.07

24 6 0.01 0.02 0.05 -0.00 0.01 -0.01 0.03 -0.03 -0.02

25 1 0.01 0.03 0.07 0.01 0.06 0.12 0.02 -0.08 -0.12

26 6 0.01 -0.00 -0.03 0.04 -0.01 0.05 -0.01 -0.01 -0.03

27 6 0.03 0.00 0.09 0.07 0.03 -0.01 -0.02 -0.03 -0.06

28 1 0.01 0.01 0.02 0.04 0.05 -0.15 0.03 -0.02 0.07

29 6 0.00 -0.00 -0.05 0.00 -0.02 -0.09 0.03 -0.00 0.08

30 1 0.01 0.02 0.08 -0.02 -0.00 -0.07 0.03 -0.03 -0.02

31 1 0.01 -0.01 -0.07 0.05 -0.04 0.04 -0.02 0.02 -0.02

32 1 0.03 0.00 0.15 0.07 0.03 -0.05 -0.02 -0.05 -0.12

33 1 -0.00 -0.01 -0.10 -0.02 -0.05 -0.22 0.05 0.02 0.20

34 8 0.01 -0.01 -0.01 0.05 -0.05 0.10 0.01 0.08 -0.08

35 6 0.00 -0.01 -0.01 -0.04 -0.02 0.01 -0.08 0.07 0.02

36 1 0.05 -0.16 0.51 -0.11 0.04 -0.47 -0.09 -0.08 0.26

37 1 0.44 0.05 -0.32 -0.43 0.03 0.27 0.08 0.08 -0.10

38 1 -0.49 0.07 -0.22 0.34 -0.11 0.16 -0.33 0.20 -0.02

39 6 -0.01 -0.00 -0.00 -0.04 0.02 0.00 -0.02 0.01 -0.01

40 8 -0.01 -0.02 0.00 -0.02 0.03 0.01 0.03 -0.00 0.02

41 8 -0.01 -0.00 0.00 -0.02 0.00 0.00 -0.03 0.01 -0.01

42 6 -0.00 -0.00 0.00 0.01 -0.01 0.01 0.01 -0.00 0.01

43 6 0.01 -0.00 -0.00 0.00 -0.02 0.00 0.00 -0.03 0.00

44 6 0.00 -0.01 0.00 0.01 -0.02 0.01 0.01 -0.01 0.01

45 6 0.00 -0.01 -0.00 -0.01 -0.03 -0.00 -0.01 -0.03 -0.01

46 1 0.01 -0.01 -0.01 0.01 -0.04 0.01 0.01 -0.05 0.02

47 6 -0.00 -0.00 0.00 -0.00 -0.02 -0.00 -0.00 -0.01 -0.01

48 6 -0.01 -0.00 0.01 -0.01 -0.03 -0.00 -0.02 -0.03 -0.01

49 1 0.01 -0.01 -0.01 -0.01 -0.03 -0.00 -0.02 -0.04 -0.01

50 1 -0.00 -0.00 0.00 -0.01 -0.01 -0.00 -0.01 0.00 -0.01

51 1 -0.01 -0.00 0.01 -0.01 -0.03 0.00 -0.02 -0.03 -0.01

52 8 0.01 -0.00 -0.00 0.01 -0.01 0.02 0.02 -0.00 0.02

53 6 0.00 0.01 -0.00 0.01 0.01 0.01 0.02 -0.00 0.03

54 1 -0.00 0.04 -0.04 0.00 0.01 0.00 0.03 -0.01 0.03

55 1 0.03 -0.03 0.00 0.00 0.01 0.00 0.02 -0.00 0.03

56 1 -0.04 0.01 0.04 0.02 0.02 0.02 0.03 -0.01 0.02

28 29 30

A A A

Frequencies -- 320.0652 323.2060 347.3647

Red. masses -- 5.1074 4.3136 5.6897

Frc consts -- 0.3083 0.2655 0.4045

IR Inten -- 1.5372 3.6662 2.9194

Atom AN X Y Z X Y Z X Y Z

1 16 0.09 0.03 0.04 -0.01 -0.01 0.00 0.00 0.01 -0.04

2 6 0.01 -0.04 -0.05 -0.00 0.00 0.01 -0.01 0.02 -0.02

3 6 0.00 -0.04 -0.15 0.00 0.00 0.02 -0.02 0.01 -0.03

4 6 -0.04 -0.06 -0.12 0.01 0.01 0.02 -0.02 0.01 -0.02

5 6 -0.04 0.03 0.03 0.01 -0.01 -0.01 -0.01 0.02 0.01

6 6 -0.10 -0.03 0.00 0.02 0.00 -0.01 -0.02 -0.01 0.03

7 6 -0.08 0.02 -0.01 0.01 -0.01 -0.00 -0.02 0.02 0.02

8 6 -0.03 -0.05 0.04 0.01 0.01 -0.01 -0.01 -0.02 0.02

9 1 -0.18 -0.05 0.00 0.03 0.01 -0.01 -0.04 -0.01 0.04

10 6 -0.04 -0.01 -0.01 0.01 0.00 -0.00 -0.01 0.00 0.01

11 1 -0.10 0.02 -0.04 0.01 -0.01 -0.00 -0.01 0.02 0.02

12 6 0.04 -0.03 0.08 0.00 0.01 -0.01 -0.01 -0.02 0.01

13 1 -0.03 -0.07 0.05 0.01 0.01 -0.01 -0.01 -0.03 0.03

14 1 -0.03 -0.00 -0.06 0.00 -0.00 0.01 0.00 0.00 -0.01

15 1 0.12 -0.03 0.13 -0.01 0.01 -0.01 -0.01 -0.03 0.01

16 8 0.08 0.10 -0.02 -0.02 -0.02 0.01 0.08 0.02 -0.00

17 8 0.19 -0.05 0.01 -0.02 -0.00 0.00 -0.02 0.05 -0.05

18 6 -0.04 -0.03 0.01 0.00 0.01 -0.01 0.03 -0.02 0.07

19 1 -0.09 -0.02 0.12 -0.00 0.01 -0.00 0.02 -0.10 0.09

20 6 -0.01 0.02 -0.03 0.00 -0.00 0.01 -0.03 -0.02 -0.02

21 6 -0.05 0.05 0.06 0.01 -0.01 -0.01 0.01 -0.03 0.00

22 6 -0.03 0.06 0.07 0.01 -0.01 -0.00 -0.04 -0.02 -0.01

23 6 -0.09 0.02 -0.05 0.01 -0.00 0.00 -0.01 -0.01 -0.01

24 6 -0.06 0.06 0.04 0.01 -0.01 -0.00 0.02 -0.02 -0.01

25 1 -0.06 0.09 0.10 0.01 -0.01 -0.02 0.04 -0.05 0.06

26 6 -0.05 0.07 0.01 0.01 -0.01 -0.00 -0.02 -0.00 0.02

27 6 -0.05 0.05 0.11 0.01 -0.00 -0.01 -0.03 -0.03 0.00

28 1 -0.14 0.02 -0.03 0.02 0.00 -0.00 0.00 -0.01 0.01

29 6 -0.07 0.06 -0.09 0.01 -0.01 0.01 0.01 -0.01 -0.00

30 1 -0.03 0.06 0.09 0.00 -0.01 -0.01 0.03 -0.02 -0.00

31 1 -0.04 0.05 -0.00 0.01 -0.01 -0.01 -0.03 0.03 0.05

32 1 -0.05 0.07 0.24 0.01 -0.01 -0.03 -0.02 -0.04 0.02

33 1 -0.08 0.05 -0.19 0.01 -0.00 0.02 0.03 0.01 0.00

34 8 -0.04 -0.14 0.04 0.00 0.02 -0.00 -0.00 0.02 0.01

35 6 0.18 -0.15 -0.04 -0.02 0.02 0.01 -0.02 0.02 0.01

36 1 0.25 0.08 -0.13 -0.03 -0.00 0.01 -0.02 0.01 -0.00

37 1 0.16 -0.23 -0.01 -0.02 0.03 0.00 -0.02 0.03 0.01

38 1 0.36 -0.29 -0.05 -0.04 0.03 0.01 -0.02 0.02 0.01

39 6 -0.00 -0.01 -0.03 0.02 0.01 -0.07 0.12 -0.00 0.09

40 8 0.01 0.00 -0.03 0.05 0.10 -0.12 -0.01 -0.01 0.01

41 8 0.01 -0.00 -0.01 -0.00 0.01 -0.06 0.29 -0.01 0.15

42 6 -0.02 0.01 0.02 -0.13 0.01 0.15 -0.05 -0.01 0.00

43 6 -0.02 0.01 0.02 -0.15 -0.02 0.16 -0.06 0.02 0.04

44 6 -0.02 0.01 0.02 -0.10 -0.01 0.14 -0.09 -0.00 -0.03

45 6 0.01 0.00 -0.02 0.09 -0.06 -0.11 0.10 0.03 0.05

46 1 -0.02 0.01 0.03 -0.17 -0.04 0.20 -0.10 0.11 -0.00

47 6 -0.02 0.02 0.02 -0.16 -0.00 0.15 0.01 -0.01 0.10

48 6 0.01 0.01 -0.02 0.08 -0.05 -0.10 0.12 0.02 0.04

49 1 0.04 -0.01 -0.05 0.27 -0.09 -0.29 0.16 0.08 0.03

50 1 -0.02 0.02 0.02 -0.17 0.02 0.14 0.03 -0.07 0.12

51 1 0.04 0.01 -0.05 0.25 -0.08 -0.29 0.20 0.01 -0.01

52 8 0.02 0.01 -0.02 0.15 -0.01 -0.08 -0.11 -0.11 -0.14

53 6 0.01 -0.01 0.01 0.05 -0.00 0.03 -0.21 0.07 -0.22

54 1 0.01 0.01 -0.01 -0.00 0.17 -0.18 -0.30 0.14 -0.36

55 1 0.05 -0.05 0.03 0.25 -0.19 0.11 -0.25 0.07 -0.30

56 1 -0.04 -0.03 0.03 -0.20 0.01 0.26 -0.14 0.23 -0.07

31 32 33

A A A

Frequencies -- 352.1790 371.8705 406.4791

Red. masses -- 6.9963 4.7121 5.3959

Frc consts -- 0.5113 0.3839 0.5253

IR Inten -- 7.3384 2.2104 13.4911

Atom AN X Y Z X Y Z X Y Z

1 16 -0.05 0.05 0.12 0.00 0.01 0.06 -0.01 -0.05 -0.04

2 6 -0.04 -0.04 0.17 -0.04 -0.02 0.01 -0.03 -0.16 0.07

3 6 0.02 -0.02 0.06 -0.02 0.04 -0.14 -0.01 -0.04 0.03

4 6 0.02 -0.04 0.02 -0.03 0.09 -0.15 -0.07 -0.03 0.05

5 6 0.11 -0.11 -0.04 0.04 -0.01 0.01 -0.04 0.04 0.02

6 6 0.03 -0.05 -0.20 -0.02 -0.01 -0.06 -0.08 0.02 0.01

7 6 -0.01 -0.16 -0.10 -0.01 -0.03 -0.04 0.03 0.08 0.04

8 6 0.03 0.07 -0.11 0.00 0.02 -0.03 0.03 0.00 0.06

9 1 0.03 -0.05 -0.34 -0.04 -0.01 -0.11 -0.16 0.00 -0.02

10 6 -0.05 -0.06 -0.02 -0.02 -0.01 -0.03 -0.03 0.02 -0.06

11 1 -0.06 -0.23 -0.12 -0.03 -0.04 -0.06 0.08 0.13 0.07

12 6 0.09 0.09 0.06 0.05 0.03 0.02 0.00 -0.01 -0.02

13 1 0.02 0.14 -0.15 -0.01 0.02 -0.04 0.07 -0.02 0.12

14 1 -0.15 -0.09 0.03 -0.06 -0.03 -0.04 -0.05 0.01 -0.13

15 1 0.16 0.18 0.19 0.09 0.06 0.07 0.01 -0.03 -0.04

16 8 -0.25 -0.06 0.09 -0.07 0.01 0.02 0.13 0.05 -0.01

17 8 -0.00 0.25 -0.08 0.07 0.02 -0.01 -0.05 0.04 -0.08

18 6 -0.01 -0.02 0.03 -0.04 -0.04 0.05 -0.14 -0.15 0.20

19 1 -0.02 0.00 0.05 -0.13 -0.05 0.21 -0.26 -0.14 0.43

20 6 0.06 0.06 -0.02 -0.04 -0.05 -0.07 0.10 0.07 0.02

21 6 -0.02 0.09 -0.02 0.05 -0.08 -0.01 0.03 0.09 0.03

22 6 0.07 0.06 -0.02 -0.04 -0.08 0.07 0.13 0.02 -0.02

23 6 0.01 0.01 0.02 -0.00 0.01 -0.15 -0.05 -0.06 0.04

24 6 -0.07 0.04 -0.01 0.09 -0.03 0.09 -0.03 0.00 -0.02

25 1 -0.05 0.17 -0.01 0.08 -0.14 -0.01 -0.01 0.16 0.01

26 6 0.03 -0.00 -0.00 0.00 -0.04 -0.04 0.10 -0.06 0.02

27 6 0.05 0.06 0.01 -0.02 -0.09 0.16 0.03 0.05 -0.05

28 1 -0.03 0.01 0.02 0.05 0.01 -0.14 -0.10 -0.05 0.01

29 6 -0.05 0.00 0.01 0.08 -0.05 -0.06 -0.00 -0.07 0.00

30 1 -0.11 0.05 -0.00 0.12 -0.01 0.22 -0.11 0.00 -0.06

31 1 0.07 -0.07 0.02 -0.03 0.02 -0.11 0.14 -0.14 0.04

32 1 0.04 0.10 0.03 0.01 -0.14 0.38 -0.03 0.18 -0.12

33 1 -0.08 -0.04 0.04 0.10 -0.03 -0.15 -0.04 -0.13 -0.00

34 8 -0.02 -0.08 -0.03 0.02 0.12 0.13 -0.04 0.06 -0.02

35 6 0.09 -0.11 -0.03 -0.10 0.18 0.07 -0.02 0.06 0.01

36 1 0.12 -0.02 0.01 -0.17 0.11 -0.11 -0.02 0.05 0.04

37 1 0.13 -0.16 -0.06 -0.25 0.28 0.16 -0.00 0.05 -0.00

38 1 0.10 -0.14 -0.05 -0.03 0.17 0.10 -0.05 0.08 0.01

39 6 0.03 -0.04 -0.02 0.01 -0.05 -0.04 -0.01 -0.13 -0.04

40 8 0.03 -0.09 0.01 0.02 -0.09 -0.01 0.04 -0.07 -0.05

41 8 0.07 -0.01 0.01 -0.00 -0.02 -0.03 0.02 -0.11 -0.06

42 6 -0.00 -0.00 -0.00 -0.00 -0.00 -0.00 -0.00 -0.04 -0.02

43 6 -0.01 0.01 0.01 0.00 0.01 0.00 0.00 0.06 0.02

44 6 -0.02 0.01 -0.01 -0.00 0.01 -0.00 -0.03 0.05 -0.03

45 6 0.02 0.01 0.01 -0.00 0.02 -0.00 0.02 0.07 0.01

46 1 -0.01 0.03 -0.01 0.00 0.02 -0.00 -0.03 0.12 -0.00

47 6 0.00 0.01 0.01 -0.01 0.02 -0.01 -0.03 0.06 -0.01

48 6 0.02 0.02 0.00 -0.00 0.02 -0.01 0.02 0.11 -0.02

49 1 0.02 0.02 0.01 -0.01 0.01 -0.00 0.01 0.04 0.00

50 1 0.01 -0.00 0.02 -0.00 0.01 -0.01 -0.01 0.01 0.02

51 1 0.03 0.02 -0.01 0.00 0.02 -0.01 0.04 0.11 -0.03

52 8 -0.02 -0.01 -0.03 0.00 0.03 0.00 -0.01 0.10 -0.02

53 6 -0.04 0.00 -0.04 0.02 -0.01 0.02 0.03 -0.04 0.03

54 1 -0.04 0.01 -0.05 0.03 -0.02 0.04 0.09 -0.05 0.09

55 1 -0.04 0.00 -0.04 0.03 -0.02 0.04 0.09 -0.06 0.09

56 1 -0.03 0.02 -0.02 -0.00 -0.04 -0.00 -0.05 -0.13 -0.04

34 35 36

A A A

Frequencies -- 413.9902 419.5553 427.6969

Red. masses -- 3.0573 5.1330 4.2609

Frc consts -- 0.3087 0.5324 0.4592

IR Inten -- 1.4733 1.9106 2.8622

Atom AN X Y Z X Y Z X Y Z

1 16 -0.01 0.01 0.00 -0.03 -0.02 -0.02 0.02 0.03 0.04

2 6 0.00 0.03 -0.01 -0.02 0.03 0.06 0.03 0.01 -0.17

3 6 0.00 0.01 -0.01 0.00 0.03 -0.04 0.01 0.02 -0.18

4 6 0.03 0.00 -0.01 0.16 0.01 -0.03 -0.04 -0.01 0.01

5 6 0.00 -0.01 -0.01 -0.00 0.01 0.01 0.05 -0.01 0.02

6 6 -0.13 -0.04 -0.14 0.02 0.02 0.03 0.01 -0.01 -0.02

7 6 0.17 0.04 0.14 -0.03 0.00 -0.03 -0.01 -0.03 -0.02

8 6 0.16 0.05 0.13 -0.03 -0.01 -0.03 -0.01 0.00 -0.03

9 1 -0.31 -0.08 -0.30 0.05 0.02 0.06 0.01 -0.01 -0.05

10 6 -0.14 -0.04 -0.13 0.03 0.01 0.02 -0.01 -0.01 -0.00

11 1 0.34 0.08 0.31 -0.07 0.00 -0.07 -0.04 -0.05 -0.05

12 6 -0.01 -0.00 -0.01 0.01 0.01 -0.00 0.02 0.01 0.02

13 1 0.33 0.10 0.29 -0.07 -0.02 -0.05 -0.04 0.00 -0.06

14 1 -0.31 -0.09 -0.27 0.06 0.02 0.04 -0.04 -0.02 -0.00

15 1 -0.04 -0.00 -0.03 0.01 0.00 -0.01 0.04 0.03 0.05

16 8 -0.02 -0.01 0.01 0.04 0.00 0.02 -0.09 0.00 -0.00

17 8 -0.00 -0.01 0.01 -0.03 0.04 -0.07 0.06 -0.01 0.05

18 6 0.03 0.02 -0.01 0.04 -0.04 0.19 -0.04 0.00 -0.02

19 1 0.03 0.01 -0.02 -0.06 -0.10 0.37 -0.14 -0.02 0.18

20 6 -0.03 -0.02 0.01 -0.14 -0.06 0.04 0.01 -0.01 -0.00

21 6 -0.01 -0.02 0.01 -0.07 -0.08 0.05 0.02 0.02 0.24

22 6 -0.04 -0.00 -0.00 -0.18 -0.00 -0.03 0.01 -0.03 -0.08

23 6 0.03 0.01 0.01 0.15 0.05 0.07 -0.02 -0.07 0.15

24 6 0.01 0.00 -0.01 0.01 0.03 -0.06 0.02 -0.01 -0.11

25 1 0.00 -0.04 0.02 -0.02 -0.16 0.09 0.04 0.04 0.37

26 6 -0.03 0.02 0.01 -0.14 0.14 0.04 0.04 0.01 0.19

27 6 -0.00 -0.01 -0.01 0.03 -0.04 -0.05 -0.04 -0.02 -0.18

28 1 0.04 0.01 0.04 0.16 0.03 0.19 0.01 -0.09 0.31

29 6 -0.00 0.03 -0.00 -0.03 0.15 0.00 0.03 -0.02 -0.08

30 1 0.03 -0.00 -0.02 0.13 0.01 -0.12 0.03 -0.04 -0.24

31 1 -0.04 0.05 0.03 -0.19 0.25 0.11 0.04 0.04 0.45

32 1 0.02 -0.05 -0.02 0.11 -0.24 -0.07 -0.06 0.02 -0.29

33 1 0.01 0.04 0.00 0.02 0.23 0.03 0.02 -0.03 -0.12

34 8 0.02 -0.02 0.00 0.16 -0.06 -0.00 -0.02 0.05 -0.00

35 6 -0.00 -0.02 -0.01 -0.02 -0.07 -0.01 -0.02 0.07 0.01

36 1 -0.01 -0.03 -0.02 -0.07 -0.21 -0.02 -0.02 0.04 0.08

37 1 -0.02 -0.00 -0.00 -0.04 0.03 -0.01 0.02 0.07 -0.02

38 1 -0.00 -0.02 -0.01 -0.09 -0.01 -0.01 -0.07 0.08 -0.01

39 6 0.01 0.02 0.00 0.07 -0.07 -0.02 -0.01 0.01 -0.01

40 8 -0.01 -0.00 0.00 0.01 -0.11 -0.04 -0.02 -0.02 -0.00

41 8 -0.01 0.01 0.00 -0.02 -0.06 -0.07 -0.00 0.01 0.00

42 6 -0.00 0.01 0.00 -0.01 -0.02 -0.02 -0.00 0.01 0.01

43 6 -0.00 -0.01 -0.00 -0.01 0.04 0.01 0.00 -0.00 -0.00

44 6 0.00 -0.01 0.00 -0.01 0.01 -0.01 0.00 -0.00 0.01

45 6 -0.00 -0.01 -0.00 0.00 0.04 -0.00 -0.00 -0.01 -0.00

46 1 0.00 -0.01 -0.00 -0.03 0.07 -0.00 0.01 -0.01 -0.01

47 6 0.00 -0.01 -0.00 -0.03 0.02 -0.01 0.00 -0.01 -0.00

48 6 -0.00 -0.01 0.00 0.01 0.05 -0.02 -0.00 -0.01 0.00

49 1 -0.00 -0.00 -0.00 -0.00 0.03 -0.01 -0.00 -0.00 -0.00

50 1 0.00 -0.00 -0.01 -0.02 -0.01 0.01 0.01 -0.00 -0.01

51 1 -0.01 -0.01 0.00 0.03 0.05 -0.02 -0.00 -0.01 0.00

52 8 0.00 -0.01 0.01 0.02 0.06 0.01 0.00 -0.01 0.00

53 6 0.00 0.00 0.00 0.04 -0.01 0.04 -0.00 0.00 -0.00

54 1 -0.00 0.01 -0.00 0.08 -0.03 0.08 -0.01 0.00 -0.01

55 1 -0.00 0.01 -0.00 0.08 -0.03 0.08 -0.01 0.01 -0.01

56 1 0.01 0.01 0.01 -0.01 -0.07 0.00 0.01 0.01 0.01

37 38 39

A A A

Frequencies -- 450.9309 475.6141 483.9018

Red. masses -- 6.6134 3.6117 4.3222

Frc consts -- 0.7923 0.4814 0.5963

IR Inten -- 14.8947 5.5062 3.6934

Atom AN X Y Z X Y Z X Y Z

1 16 -0.01 0.06 -0.05 -0.01 -0.01 -0.00 -0.06 -0.00 0.00

2 6 0.19 0.13 -0.04 -0.00 -0.01 0.04 -0.11 0.03 0.21

3 6 0.10 0.07 -0.05 0.00 0.00 0.00 -0.05 0.01 0.05

4 6 -0.11 0.07 0.01 -0.01 0.00 -0.01 -0.03 0.00 -0.05

5 6 -0.01 -0.01 -0.05 -0.00 0.00 0.01 -0.10 -0.04 -0.09

6 6 0.03 -0.03 -0.00 -0.00 0.01 0.00 0.02 0.00 0.01

7 6 -0.01 -0.02 0.02 -0.00 0.00 -0.00 0.01 -0.01 -0.00

8 6 0.01 -0.02 0.01 -0.00 0.00 -0.00 0.04 0.02 0.03

9 1 0.04 -0.03 0.02 -0.01 0.01 0.00 0.11 0.02 0.07

10 6 -0.01 -0.01 0.04 0.00 0.00 -0.01 0.05 0.01 0.04

11 1 0.01 -0.05 0.05 -0.00 0.01 -0.01 0.08 0.01 0.06

12 6 -0.04 -0.03 0.02 0.01 0.00 -0.00 -0.06 -0.01 -0.06

13 1 0.03 0.02 0.01 -0.01 -0.01 -0.00 0.11 0.04 0.10

14 1 0.02 -0.01 0.05 0.00 0.00 -0.01 0.13 0.04 0.13

15 1 -0.06 -0.03 0.01 0.01 0.00 -0.00 -0.10 -0.02 -0.10

16 8 0.00 -0.04 0.02 0.02 0.01 0.00 0.10 0.04 0.08

17 8 -0.08 0.11 -0.02 -0.01 0.02 -0.03 0.06 -0.03 -0.10

18 6 0.29 0.06 0.23 0.03 -0.02 0.03 0.07 0.03 -0.03

19 1 0.18 -0.02 0.42 0.01 -0.04 0.07 0.19 -0.01 -0.29

20 6 0.03 -0.05 -0.02 -0.00 -0.00 -0.01 -0.01 0.01 -0.17

21 6 0.04 -0.08 -0.02 -0.00 0.00 0.01 0.01 0.07 0.12

22 6 -0.01 -0.03 0.00 -0.00 -0.00 -0.02 0.02 -0.04 -0.16

23 6 -0.14 0.06 0.00 -0.01 -0.00 -0.00 -0.01 -0.05 -0.06

24 6 0.02 -0.11 0.02 -0.00 0.00 -0.00 0.01 0.05 -0.04

25 1 0.05 -0.08 0.02 0.00 0.01 0.04 0.05 0.12 0.37

26 6 -0.01 -0.07 -0.01 0.00 -0.00 0.01 0.06 -0.00 0.12

27 6 -0.16 0.01 -0.01 -0.01 -0.01 0.01 0.00 -0.06 0.07

28 1 -0.05 0.05 -0.02 0.00 -0.00 0.01 0.03 -0.07 0.05

29 6 0.02 -0.08 0.00 0.00 -0.00 -0.00 0.04 -0.01 -0.07

30 1 0.02 -0.10 0.07 0.01 0.00 -0.00 -0.00 0.07 0.04

31 1 -0.00 -0.09 -0.02 0.00 0.00 0.03 0.06 0.03 0.42

32 1 -0.18 0.07 -0.03 -0.00 -0.01 0.04 0.01 -0.06 0.35

33 1 0.04 -0.06 0.00 -0.00 -0.00 0.01 0.01 -0.05 -0.00

34 8 -0.20 -0.01 -0.00 -0.01 0.00 0.00 -0.02 -0.01 0.01

35 6 0.07 -0.03 -0.01 0.00 -0.00 -0.00 -0.00 -0.02 -0.00

36 1 0.16 0.22 0.04 0.00 0.00 0.00 0.01 0.00 -0.00

37 1 0.15 -0.17 -0.05 0.00 -0.00 -0.00 0.00 -0.02 -0.01

38 1 0.16 -0.14 -0.04 0.00 -0.00 -0.00 0.01 -0.04 -0.01

39 6 0.17 0.00 0.08 0.04 -0.02 -0.03 0.05 0.03 0.03

40 8 -0.06 -0.05 -0.04 0.03 0.04 -0.08 -0.03 -0.00 -0.00

41 8 -0.07 -0.02 -0.05 -0.01 0.04 0.01 -0.02 0.03 -0.01

42 6 -0.02 -0.01 -0.04 -0.20 0.04 0.20 0.01 0.02 -0.03

43 6 -0.03 0.04 -0.02 0.13 -0.03 -0.12 -0.03 0.02 -0.01

44 6 0.01 -0.04 -0.02 -0.13 0.02 0.15 0.03 -0.03 -0.01

45 6 -0.01 0.04 -0.03 -0.03 -0.02 0.03 -0.01 0.01 -0.03

46 1 -0.06 0.06 -0.01 0.43 -0.09 -0.39 -0.06 0.01 0.03

47 6 -0.03 -0.04 -0.02 0.14 -0.02 -0.12 -0.02 -0.03 0.01

48 6 -0.01 0.00 -0.01 -0.08 -0.01 0.08 -0.01 -0.02 -0.01

49 1 -0.01 0.05 -0.02 0.02 -0.02 -0.02 -0.01 0.03 -0.02

50 1 -0.04 -0.05 0.01 0.45 -0.05 -0.43 -0.06 -0.01 0.03

51 1 0.02 0.01 0.03 -0.08 -0.02 0.06 0.01 -0.02 0.02

52 8 0.04 0.03 0.04 0.04 -0.02 -0.04 0.01 -0.02 0.03

53 6 0.07 0.00 0.07 -0.01 0.01 -0.01 0.02 0.01 0.02

54 1 0.08 -0.00 0.09 -0.06 -0.02 -0.03 0.01 0.01 0.01

55 1 0.08 -0.01 0.08 -0.01 0.04 0.02 0.01 0.01 0.00

56 1 0.04 -0.03 0.04 0.02 0.03 -0.00 0.03 0.02 0.03

40 41 42

A A A

Frequencies -- 500.6742 527.6407 536.8161

Red. masses -- 4.5225 5.7834 5.2306

Frc consts -- 0.6679 0.9487 0.8881

IR Inten -- 20.5554 35.0657 8.9696

Atom AN X Y Z X Y Z X Y Z

1 16 0.06 -0.09 -0.03 -0.10 -0.03 -0.00 0.05 0.08 -0.03

2 6 -0.00 -0.02 0.09 -0.11 0.09 -0.02 -0.01 0.03 -0.07

3 6 -0.01 -0.00 0.06 -0.05 0.03 -0.04 -0.06 -0.01 0.04

4 6 -0.01 0.01 -0.03 -0.01 -0.03 0.08 -0.01 -0.07 0.13

5 6 0.18 0.08 0.22 0.11 0.01 0.14 -0.00 0.02 -0.06

6 6 -0.06 0.04 0.02 0.00 0.03 0.01 0.02 -0.03 -0.02

7 6 -0.01 0.05 0.01 0.03 -0.01 -0.01 -0.01 0.00 0.01

8 6 -0.09 -0.02 -0.05 -0.05 0.01 -0.05 0.02 -0.02 0.01

9 1 -0.23 -0.01 -0.11 -0.08 0.01 -0.10 0.01 -0.04 0.02

10 6 -0.07 -0.01 -0.10 -0.03 -0.02 -0.06 -0.01 0.01 0.03

11 1 -0.15 0.05 -0.14 -0.07 -0.00 -0.11 0.00 -0.04 0.05

12 6 0.14 0.04 0.09 0.09 0.05 0.03 -0.04 -0.04 0.02

13 1 -0.26 -0.11 -0.19 -0.18 -0.05 -0.14 0.05 0.02 0.01

14 1 -0.24 -0.06 -0.29 -0.17 -0.06 -0.14 0.03 0.02 0.03

15 1 0.22 0.04 0.15 0.11 0.06 0.05 -0.04 -0.03 0.02

16 8 -0.00 -0.03 -0.13 0.11 0.06 0.07 -0.06 -0.07 -0.01

17 8 -0.16 0.04 0.05 0.04 -0.10 -0.08 0.00 0.01 0.09

18 6 0.09 -0.02 -0.03 -0.04 0.04 0.06 0.02 -0.00 -0.01

19 1 0.20 -0.03 -0.26 -0.16 -0.11 0.31 0.07 -0.02 -0.11

20 6 -0.01 -0.01 -0.09 -0.02 0.05 0.03 -0.05 0.03 -0.09

21 6 -0.00 0.02 0.05 0.01 0.05 -0.09 -0.06 0.08 -0.09

22 6 -0.01 -0.02 -0.10 0.02 -0.01 0.14 0.00 -0.08 0.11

23 6 -0.00 -0.01 -0.05 0.01 -0.05 0.04 0.02 -0.15 -0.04

24 6 0.00 0.02 -0.03 0.01 0.05 0.06 -0.01 0.19 0.09

25 1 0.02 0.05 0.21 0.02 0.00 -0.17 -0.03 0.06 -0.03

26 6 0.01 0.01 0.05 0.02 -0.03 -0.01 0.06 0.04 0.06

27 6 -0.01 -0.03 0.06 0.02 -0.01 -0.06 0.03 -0.13 -0.02

28 1 0.02 -0.02 0.01 -0.02 -0.03 -0.13 0.03 -0.12 -0.28

29 6 0.01 0.01 -0.02 0.03 -0.03 -0.02 0.02 0.05 -0.11

30 1 0.01 0.03 0.03 -0.04 0.06 0.09 -0.00 0.22 0.27

31 1 0.01 0.04 0.21 0.01 -0.03 -0.16 0.03 0.11 0.07

32 1 0.01 -0.06 0.24 -0.00 0.04 -0.31 0.01 -0.12 -0.20

33 1 0.00 0.00 0.04 0.00 -0.07 -0.13 -0.05 -0.04 -0.21

34 8 -0.01 -0.00 -0.01 -0.01 -0.01 -0.03 0.02 0.01 -0.03

35 6 0.00 -0.01 -0.00 -0.00 -0.03 -0.01 -0.02 0.00 -0.00

36 1 0.00 -0.00 0.00 0.00 -0.02 -0.00 -0.04 -0.04 -0.01

37 1 0.00 -0.02 0.00 0.01 -0.05 -0.02 -0.04 0.01 0.01

38 1 0.01 -0.01 0.00 -0.00 -0.02 -0.01 -0.03 0.03 0.01

39 6 0.04 0.01 0.06 0.09 0.07 -0.02 0.02 -0.02 0.07

40 8 -0.03 0.07 -0.01 -0.02 -0.09 0.02 -0.02 0.02 0.02

41 8 -0.03 -0.01 0.02 0.06 0.15 0.00 0.08 -0.10 0.03

42 6 -0.00 -0.01 -0.02 -0.01 0.12 -0.03 -0.08 -0.07 -0.07

43 6 -0.02 0.02 -0.00 -0.06 0.08 -0.06 -0.02 0.05 -0.09

44 6 0.01 -0.02 -0.03 0.07 -0.09 0.04 -0.13 -0.05 0.01

45 6 0.01 0.02 -0.02 -0.09 0.07 -0.12 -0.04 0.09 0.04

46 1 -0.05 0.03 0.02 -0.04 -0.00 -0.01 -0.01 0.15 -0.21

47 6 -0.02 -0.02 -0.00 -0.03 -0.09 -0.02 -0.05 -0.09 -0.03

48 6 -0.00 0.01 0.00 -0.05 -0.08 -0.03 0.06 0.02 -0.01

49 1 0.01 0.02 -0.01 -0.06 0.14 -0.10 -0.03 0.14 0.07

50 1 -0.04 -0.02 0.03 -0.09 0.00 -0.04 0.07 -0.20 -0.06

51 1 -0.01 0.01 0.03 0.02 -0.08 0.07 0.20 0.01 -0.02

52 8 0.00 0.01 0.01 0.06 -0.15 0.08 0.04 0.09 -0.01

53 6 0.02 0.00 0.02 0.04 0.03 0.04 0.08 0.01 0.07

54 1 0.03 -0.00 0.03 -0.03 0.05 -0.04 0.12 -0.02 0.13

55 1 0.02 -0.00 0.02 -0.04 0.05 -0.04 0.15 -0.01 0.15

56 1 0.01 -0.01 0.01 0.13 0.14 0.12 -0.02 -0.10 0.00

43 44 45

A A A

Frequencies -- 542.2686 546.9350 549.3660

Red. masses -- 4.3668 5.6530 6.2668

Frc consts -- 0.7566 0.9963 1.1143

IR Inten -- 5.2898 5.4506 41.4564

Atom AN X Y Z X Y Z X Y Z

1 16 0.04 0.01 -0.01 -0.01 0.04 -0.03 0.15 0.02 -0.05

2 6 -0.01 -0.02 -0.01 -0.01 0.07 -0.04 0.01 -0.02 0.11

3 6 -0.01 -0.00 0.00 -0.05 0.07 0.00 0.01 -0.03 0.06

4 6 -0.01 0.04 -0.15 -0.05 0.04 -0.07 0.00 -0.03 0.04

5 6 -0.01 0.01 -0.03 0.05 0.02 0.02 -0.06 0.04 -0.10

6 6 0.00 -0.01 -0.00 0.02 -0.01 -0.00 -0.01 -0.02 -0.00

7 6 -0.01 0.01 0.00 0.00 0.00 0.01 -0.05 0.05 0.02

8 6 0.01 -0.01 0.01 -0.01 -0.01 -0.02 0.04 -0.04 0.03

9 1 0.01 -0.01 0.02 -0.02 -0.02 -0.02 -0.01 -0.02 0.10

10 6 0.00 0.00 0.01 -0.02 -0.00 -0.00 0.01 0.03 0.04

11 1 0.01 0.00 0.03 -0.03 -0.03 -0.01 0.01 0.03 0.09

12 6 -0.02 -0.02 0.00 0.01 -0.01 0.03 -0.06 -0.07 0.01

13 1 0.03 0.01 0.02 -0.04 -0.00 -0.06 0.11 0.01 0.09

14 1 0.03 0.01 0.02 -0.05 -0.01 -0.04 0.13 0.07 0.06

15 1 -0.02 -0.02 0.00 0.01 -0.00 0.04 -0.07 -0.08 -0.01

16 8 -0.02 -0.03 -0.02 0.02 -0.02 0.03 -0.07 -0.13 -0.12

17 8 -0.00 0.01 0.04 0.01 -0.03 0.02 -0.09 0.09 0.13

18 6 -0.07 0.00 -0.02 0.05 0.02 0.07 -0.12 0.03 0.13

19 1 -0.07 0.01 -0.03 -0.01 -0.01 0.18 -0.25 0.09 0.40

20 6 0.00 0.02 0.17 -0.07 0.08 0.16 0.01 -0.02 -0.07

21 6 0.01 0.02 0.09 -0.07 0.13 0.02 -0.04 -0.00 0.00

22 6 0.02 0.00 -0.11 0.02 -0.14 0.01 -0.00 -0.01 -0.03

23 6 -0.00 0.01 -0.02 0.03 -0.15 -0.04 -0.00 -0.02 -0.04

24 6 -0.01 -0.03 -0.15 -0.01 0.21 -0.11 -0.02 0.04 0.03

25 1 -0.00 -0.02 -0.00 -0.03 -0.01 -0.10 -0.03 0.03 0.08

26 6 0.01 -0.02 -0.13 0.13 -0.01 -0.11 0.02 0.04 0.04

27 6 0.02 -0.00 0.06 0.01 -0.21 0.02 -0.01 -0.03 0.02

28 1 0.03 -0.02 0.22 0.18 -0.18 0.02 -0.01 -0.02 -0.08

29 6 0.02 0.00 0.18 0.10 0.04 0.12 -0.02 0.04 -0.05

30 1 -0.05 -0.07 -0.44 -0.08 0.18 -0.33 0.03 0.05 0.12

31 1 0.02 -0.03 -0.19 0.07 0.10 -0.23 0.02 0.05 0.15

32 1 0.03 -0.01 0.30 0.01 -0.22 0.08 -0.00 -0.04 0.07

33 1 0.03 0.01 0.36 0.00 -0.09 0.22 -0.04 0.02 -0.05

34 8 -0.02 -0.01 0.04 -0.09 -0.03 0.02 0.02 0.01 -0.00

35 6 0.00 -0.00 0.01 -0.01 -0.05 -0.01 -0.01 0.02 0.00

36 1 0.01 0.02 0.00 0.03 0.06 0.01 -0.02 -0.01 -0.00

37 1 0.01 0.01 0.01 0.03 -0.10 -0.02 -0.02 0.03 0.01

38 1 0.02 -0.03 -0.01 0.04 -0.11 -0.02 -0.02 0.04 0.01

39 6 -0.01 0.02 0.01 0.03 -0.01 -0.03 -0.01 0.02 -0.12

40 8 -0.00 -0.02 0.04 0.01 -0.03 -0.03 0.04 -0.13 -0.00

41 8 0.12 0.00 0.04 -0.10 0.00 -0.06 0.03 0.14 -0.05

42 6 -0.05 0.01 -0.07 0.05 -0.00 0.06 0.06 0.13 0.05

43 6 -0.04 0.09 -0.11 0.04 -0.08 0.09 0.01 0.04 0.03

44 6 -0.07 -0.09 0.04 0.05 0.08 -0.01 0.12 -0.01 0.06

45 6 -0.10 0.12 -0.03 0.06 -0.10 0.03 -0.06 0.01 -0.10

46 1 -0.01 0.12 -0.18 0.04 -0.11 0.13 0.06 -0.11 0.12

47 6 -0.07 -0.11 -0.03 0.05 0.10 0.03 0.00 0.02 -0.00

48 6 0.03 -0.02 -0.04 -0.01 0.01 0.02 -0.07 -0.04 -0.04

49 1 -0.08 0.19 0.01 0.05 -0.16 0.01 -0.06 -0.01 -0.11

50 1 -0.00 -0.14 -0.07 0.01 0.13 0.05 -0.08 0.18 -0.05

51 1 0.20 -0.03 -0.01 -0.14 0.02 -0.03 -0.09 -0.04 -0.00

52 8 0.07 -0.02 0.03 -0.05 0.01 -0.03 0.01 -0.19 0.05

53 6 0.07 0.02 0.07 -0.06 -0.02 -0.06 -0.04 0.01 -0.04

54 1 0.06 0.01 0.06 -0.06 -0.01 -0.06 -0.14 0.04 -0.15

55 1 0.09 0.01 0.08 -0.07 -0.01 -0.07 -0.15 0.04 -0.16

56 1 0.06 0.01 0.07 -0.04 0.00 -0.05 0.10 0.18 0.09

46 47 48

A A A

Frequencies -- 561.2283 575.2542 592.1863

Red. masses -- 4.9886 6.8484 5.9479

Frc consts -- 0.9258 1.3352 1.2289

IR Inten -- 39.7604 131.4196 26.6766

Atom AN X Y Z X Y Z X Y Z

1 16 -0.02 0.05 -0.06 -0.03 0.18 -0.12 0.09 -0.07 0.02

2 6 -0.07 0.10 0.05 -0.12 0.07 -0.02 -0.14 0.07 0.17

3 6 -0.01 0.03 0.04 -0.07 -0.04 0.08 -0.02 0.13 -0.08

4 6 0.01 0.03 0.02 0.02 -0.04 0.02 0.01 0.13 -0.06

5 6 0.08 0.03 0.03 0.14 0.07 -0.02 -0.05 0.01 -0.01

6 6 0.03 -0.02 0.00 0.08 -0.08 -0.02 -0.04 0.04 0.01

7 6 0.00 0.01 0.02 0.00 0.03 0.03 -0.03 0.02 0.01

8 6 -0.02 -0.02 -0.03 -0.01 -0.06 -0.04 0.02 0.00 0.01

9 1 -0.04 -0.04 -0.03 -0.04 -0.11 -0.03 -0.01 0.04 0.05

10 6 -0.04 0.00 -0.01 -0.08 0.02 0.01 0.03 -0.01 0.01

11 1 -0.05 -0.03 -0.02 -0.07 -0.11 0.01 0.00 0.06 0.03

12 6 0.02 -0.01 0.05 -0.01 -0.05 0.12 -0.01 -0.01 -0.03

13 1 -0.09 -0.01 -0.10 -0.10 0.02 -0.17 0.06 -0.02 0.07

14 1 -0.10 -0.02 -0.09 -0.14 0.00 -0.12 0.09 0.01 0.03

15 1 0.03 -0.01 0.06 -0.00 -0.04 0.13 -0.01 -0.01 -0.03

16 8 0.04 -0.05 0.03 0.01 -0.11 0.09 0.01 -0.03 -0.08

17 8 -0.00 -0.08 0.03 0.04 -0.14 0.10 -0.03 0.05 0.03

18 6 -0.06 0.08 0.02 -0.08 0.02 0.03 -0.09 0.06 -0.04

19 1 -0.07 0.09 0.05 -0.09 -0.03 0.05 -0.06 0.09 -0.09

20 6 0.00 -0.00 -0.05 0.02 0.00 -0.04 -0.06 0.07 0.03

21 6 0.04 -0.01 0.02 0.07 -0.01 0.05 0.06 0.03 -0.04

22 6 -0.00 0.02 -0.04 0.01 0.08 -0.09 -0.03 -0.02 0.08

23 6 0.01 0.07 -0.00 -0.00 0.05 -0.05 0.07 0.09 0.11

24 6 0.02 -0.06 0.02 0.01 -0.10 -0.02 0.06 0.00 0.05

25 1 0.04 0.00 0.04 0.05 0.05 0.09 0.08 -0.06 -0.16

26 6 -0.02 -0.03 0.03 -0.05 -0.02 -0.00 -0.03 -0.08 0.05

27 6 0.00 0.05 0.01 0.05 0.11 0.06 0.00 -0.01 -0.11

28 1 0.02 0.07 -0.00 -0.11 0.07 -0.05 0.21 0.06 0.23

29 6 0.01 -0.05 -0.02 -0.02 -0.04 0.02 0.09 -0.10 -0.04

30 1 0.01 -0.05 0.05 -0.01 -0.10 -0.02 -0.05 0.01 0.04

31 1 -0.01 -0.05 0.10 -0.02 -0.07 0.10 -0.05 -0.03 -0.06

32 1 0.01 0.04 0.05 0.05 0.12 0.18 0.03 -0.07 -0.23

33 1 0.04 -0.02 -0.03 0.04 0.03 0.09 0.09 -0.09 -0.18

34 8 -0.03 -0.01 -0.01 0.05 0.00 -0.00 -0.11 -0.04 0.00

35 6 0.01 -0.03 -0.01 -0.00 -0.00 -0.00 0.02 -0.08 -0.02

36 1 0.03 0.02 0.00 -0.02 -0.05 -0.02 0.09 0.08 0.03

37 1 0.03 -0.06 -0.02 -0.02 0.04 0.01 0.08 -0.16 -0.05

38 1 0.03 -0.05 -0.01 -0.02 0.03 0.01 0.08 -0.16 -0.05

39 6 -0.05 0.03 -0.05 0.02 -0.02 -0.04 -0.04 0.03 -0.07

40 8 -0.00 -0.09 0.06 0.03 -0.06 -0.03 0.03 -0.07 0.03

41 8 -0.03 -0.00 -0.08 0.04 -0.03 -0.01 0.06 0.04 -0.01

42 6 0.12 -0.01 -0.07 -0.12 -0.02 0.05 -0.09 0.01 -0.05

43 6 0.12 -0.07 -0.07 -0.11 -0.02 0.05 -0.14 -0.07 -0.07

44 6 -0.14 0.06 0.15 0.05 -0.01 -0.11 -0.02 0.02 -0.08

45 6 -0.14 -0.05 0.20 0.15 -0.04 -0.09 0.08 -0.08 0.01

46 1 0.20 -0.09 -0.13 -0.14 0.03 0.03 -0.16 -0.02 -0.10

47 6 -0.04 0.06 0.11 0.09 -0.02 -0.05 0.09 -0.04 0.05

48 6 0.16 -0.03 -0.13 -0.09 -0.00 0.14 0.02 -0.11 0.12

49 1 -0.27 -0.05 0.31 0.29 -0.01 -0.19 0.19 0.06 0.02

50 1 0.07 0.05 -0.01 0.07 -0.07 0.00 0.11 -0.10 0.08

51 1 0.35 -0.06 -0.42 -0.26 0.02 0.29 -0.07 -0.11 0.12

52 8 0.03 0.02 -0.08 -0.04 0.08 0.01 -0.02 0.14 -0.02

53 6 -0.02 -0.01 -0.04 0.01 0.00 0.02 0.03 0.01 0.03

54 1 -0.05 -0.00 -0.06 0.07 -0.02 0.09 0.11 -0.02 0.13

55 1 0.01 -0.01 0.00 0.04 -0.01 0.05 0.10 -0.01 0.11

56 1 -0.06 -0.01 -0.01 -0.02 -0.07 -0.05 -0.06 -0.12 -0.07

49 50 51

A A A

Frequencies -- 603.4946 622.8743 625.4362

Red. masses -- 5.4376 5.1654 6.4102

Frc consts -- 1.1668 1.1807 1.4774

IR Inten -- 8.3405 8.1002 0.2110

Atom AN X Y Z X Y Z X Y Z

1 16 0.02 0.04 -0.02 0.03 0.03 -0.03 0.01 -0.01 -0.01

2 6 -0.12 0.01 0.09 -0.13 -0.02 0.18 0.02 -0.03 0.06

3 6 -0.05 0.08 -0.07 -0.05 -0.09 -0.18 0.03 0.00 -0.02

4 6 0.01 0.08 -0.07 -0.04 -0.13 -0.16 -0.00 0.02 -0.04

5 6 0.02 0.03 -0.03 0.01 0.04 -0.04 0.08 -0.10 -0.05

6 6 0.02 -0.02 -0.01 0.02 -0.01 -0.02 0.02 -0.30 0.07

7 6 -0.01 0.02 0.01 -0.03 0.03 0.02 0.21 -0.00 -0.23

8 6 0.01 -0.02 -0.01 0.03 -0.03 -0.02 -0.24 0.01 0.25

9 1 0.00 -0.03 0.02 -0.01 -0.02 0.03 0.12 -0.27 -0.05

10 6 -0.02 0.02 0.01 -0.01 0.01 0.02 -0.04 0.34 -0.08

11 1 -0.01 -0.02 0.02 -0.03 -0.00 0.04 0.21 -0.15 -0.16

12 6 -0.01 -0.02 0.03 -0.02 -0.04 0.03 -0.08 0.11 0.06

13 1 0.01 0.01 -0.02 0.05 0.01 -0.02 -0.22 0.17 0.18

14 1 0.00 0.02 -0.02 0.04 0.03 -0.01 -0.15 0.30 0.04

15 1 -0.01 -0.02 0.03 -0.02 -0.03 0.04 0.16 -0.19 -0.09

16 8 -0.00 -0.03 0.00 -0.02 -0.04 -0.02 0.01 -0.01 -0.01

17 8 0.01 -0.01 0.04 -0.01 0.02 0.03 -0.01 -0.00 -0.00

18 6 0.01 -0.04 -0.06 0.12 -0.04 0.01 0.00 -0.00 -0.01

19 1 0.17 -0.02 -0.39 0.22 -0.16 -0.21 0.02 0.04 -0.04

20 6 -0.06 0.07 0.02 0.02 0.02 0.06 0.01 -0.01 0.01

21 6 0.07 0.04 -0.04 0.03 0.02 -0.03 -0.03 -0.02 -0.01

22 6 -0.04 0.00 0.06 0.03 0.06 0.12 0.01 -0.01 0.02

23 6 0.06 0.06 0.09 -0.10 -0.16 0.08 -0.00 0.00 0.02

24 6 0.05 -0.02 0.04 -0.01 -0.03 0.01 -0.01 0.01 0.01

25 1 0.08 -0.03 -0.13 0.00 0.02 -0.15 -0.03 -0.02 -0.03

26 6 -0.05 -0.07 0.04 -0.03 0.01 -0.02 0.02 0.01 0.01

27 6 0.02 0.01 -0.09 0.01 0.07 -0.08 -0.02 -0.01 -0.03

28 1 0.13 0.03 0.22 -0.28 -0.16 0.25 0.03 -0.01 0.10

29 6 0.07 -0.09 -0.02 -0.04 0.00 0.04 -0.01 0.02 -0.01

30 1 -0.05 -0.01 0.02 -0.05 -0.05 -0.14 0.02 0.01 0.01

31 1 -0.08 -0.03 -0.06 -0.01 -0.05 -0.26 0.02 0.01 -0.02

32 1 0.04 -0.03 -0.16 -0.05 0.20 -0.15 -0.02 -0.02 -0.03

33 1 0.09 -0.06 -0.15 -0.03 0.02 -0.10 -0.02 -0.01 -0.05

34 8 -0.06 -0.03 0.01 0.14 0.02 0.02 -0.01 0.00 0.01

35 6 0.01 -0.05 -0.01 -0.01 0.07 0.02 0.00 0.01 0.00

36 1 0.05 0.04 0.02 -0.09 -0.14 -0.03 0.01 0.02 0.01

37 1 0.05 -0.09 -0.03 -0.08 0.20 0.05 0.01 0.00 -0.00

38 1 0.04 -0.10 -0.03 -0.10 0.15 0.03 0.00 -0.00 -0.00

39 6 -0.02 -0.08 0.05 0.17 0.02 0.09 -0.03 0.00 -0.01

40 8 0.01 0.05 -0.02 -0.05 0.04 -0.04 0.01 0.01 0.01

41 8 0.00 -0.13 0.01 -0.10 0.05 -0.00 0.01 0.01 0.01

42 6 0.06 -0.09 0.10 0.01 0.03 -0.03 -0.00 0.01 -0.01

43 6 0.15 0.10 0.15 0.00 -0.03 -0.05 -0.01 -0.00 -0.02

44 6 0.01 -0.06 0.04 0.02 0.03 -0.00 0.00 0.00 -0.00

45 6 -0.01 0.12 -0.06 -0.03 -0.04 0.04 -0.00 -0.00 -0.00

46 1 0.16 0.10 0.14 -0.02 -0.05 -0.02 -0.02 -0.00 -0.01

47 6 -0.11 0.01 -0.14 0.01 0.03 0.06 0.01 -0.00 0.01

48 6 -0.09 0.21 -0.08 0.04 -0.05 -0.02 0.01 -0.02 0.00

49 1 -0.09 -0.10 -0.16 -0.07 0.01 0.11 0.00 0.02 0.01

50 1 -0.08 0.01 -0.17 -0.03 0.05 0.08 -0.00 0.00 0.02

51 1 -0.01 0.21 0.00 0.03 -0.06 -0.07 0.00 -0.02 0.00

52 8 0.02 -0.13 0.03 -0.00 0.01 -0.01 0.00 0.00 0.00

53 6 -0.03 -0.02 -0.03 -0.00 0.00 -0.00 0.00 0.00 0.00

54 1 -0.09 0.00 -0.10 0.00 0.01 -0.00 0.01 0.00 0.01

55 1 -0.10 0.01 -0.10 0.00 -0.00 -0.00 0.01 0.00 0.01

56 1 0.06 0.09 0.06 -0.01 0.00 -0.00 -0.00 -0.00 -0.00

52 53 54

A A A

Frequencies -- 634.8429 677.5087 692.8235

Red. masses -- 6.2956 5.5193 4.1993

Frc consts -- 1.4949 1.4927 1.1876

IR Inten -- 23.9368 1.4184 2.3562

Atom AN X Y Z X Y Z X Y Z

1 16 -0.07 0.06 -0.06 0.03 -0.03 -0.00 -0.01 -0.01 0.01

2 6 0.12 -0.11 0.22 -0.13 0.21 0.11 -0.01 -0.10 0.10

3 6 0.18 -0.02 -0.05 0.06 0.01 -0.17 0.06 0.06 -0.20

4 6 -0.00 0.08 -0.15 0.00 -0.07 0.12 0.02 0.01 0.22

5 6 0.04 0.06 -0.01 -0.02 -0.05 0.06 0.02 0.06 -0.08

6 6 0.06 0.03 -0.01 -0.06 0.03 0.02 0.07 -0.03 -0.02

7 6 -0.02 0.03 0.07 0.01 -0.04 -0.03 -0.01 0.08 0.03

8 6 0.03 -0.03 -0.09 -0.02 0.04 0.05 0.02 -0.05 -0.07

9 1 -0.03 0.00 0.01 -0.03 0.04 -0.04 0.06 -0.04 0.08

10 6 -0.05 -0.04 0.00 0.05 -0.03 -0.00 -0.06 0.06 -0.01

11 1 -0.07 -0.01 0.04 -0.00 0.01 -0.07 0.03 0.01 0.11

12 6 0.03 -0.04 0.06 0.01 0.04 -0.06 -0.02 -0.05 0.08

13 1 -0.04 -0.02 -0.18 -0.03 -0.04 0.09 0.05 0.05 -0.09

14 1 -0.09 -0.06 -0.13 0.00 -0.04 0.06 0.02 0.09 -0.08

15 1 -0.02 0.02 0.09 -0.00 0.02 -0.09 0.04 -0.04 0.13

16 8 0.04 -0.01 0.07 0.02 -0.02 -0.05 -0.00 0.01 0.02

17 8 -0.02 -0.08 -0.03 -0.01 0.01 0.01 -0.00 -0.01 -0.02

18 6 -0.00 0.02 -0.04 -0.01 0.17 -0.03 0.00 -0.08 -0.03

19 1 0.08 0.26 -0.18 0.03 0.11 -0.12 0.04 -0.02 -0.10

20 6 0.08 -0.12 0.04 0.04 -0.09 -0.06 -0.01 -0.01 -0.11

21 6 -0.17 -0.14 -0.05 -0.09 -0.08 0.09 0.00 0.01 0.11

22 6 0.07 -0.06 0.04 0.08 -0.01 0.07 -0.03 -0.03 0.08

23 6 -0.03 0.02 0.08 -0.07 -0.01 -0.01 0.05 0.07 -0.01

24 6 -0.07 0.06 0.06 -0.04 -0.03 -0.07 0.01 -0.01 -0.10

25 1 -0.18 -0.13 -0.10 -0.09 -0.05 0.12 0.01 0.01 0.17

26 6 0.17 0.09 0.04 0.13 0.04 -0.09 -0.03 -0.01 -0.09

27 6 -0.10 -0.06 -0.10 -0.05 0.03 0.05 -0.00 -0.03 0.07

28 1 0.12 -0.04 0.38 -0.16 0.05 -0.36 0.07 0.14 -0.57

29 6 -0.07 0.12 -0.07 -0.04 0.06 0.08 0.03 0.01 0.10

30 1 0.14 0.04 0.09 0.08 -0.06 -0.19 0.00 -0.02 -0.19

31 1 0.19 0.04 -0.01 0.16 -0.03 -0.18 -0.05 0.04 -0.16

32 1 -0.10 -0.06 -0.04 -0.11 0.14 -0.18 0.01 -0.08 -0.31

33 1 -0.17 -0.01 -0.16 -0.09 -0.01 0.10 0.04 0.03 0.14

34 8 -0.05 0.01 0.03 0.05 0.01 -0.02 -0.05 0.01 -0.03

35 6 -0.00 0.04 0.01 0.00 0.02 0.00 -0.00 0.00 -0.01

36 1 0.02 0.09 0.03 -0.02 -0.04 -0.02 0.02 0.07 -0.00

37 1 0.02 0.03 0.00 -0.03 0.05 0.02 0.02 -0.06 -0.01

38 1 0.01 0.01 -0.00 -0.02 0.06 0.02 0.04 -0.02 -0.00

39 6 -0.17 -0.01 -0.08 -0.00 0.07 -0.02 -0.03 -0.05 -0.00

40 8 0.05 0.02 0.04 -0.02 -0.07 0.06 0.02 0.04 -0.03

41 8 0.08 -0.00 0.04 0.05 -0.06 -0.08 -0.02 0.02 0.06

42 6 -0.01 0.01 -0.01 0.05 -0.09 -0.00 -0.04 0.03 0.03

43 6 -0.03 0.01 -0.01 0.02 0.01 0.13 0.00 -0.00 -0.06

44 6 -0.00 -0.01 -0.01 -0.10 -0.03 0.05 0.06 0.00 -0.05

45 6 0.00 0.02 -0.02 0.08 -0.01 -0.02 -0.05 0.01 0.02

46 1 -0.02 0.02 -0.03 0.04 0.04 0.08 0.01 -0.01 -0.06

47 6 0.00 -0.02 -0.01 -0.02 -0.04 -0.10 -0.01 0.01 0.04

48 6 -0.01 -0.01 0.01 -0.07 0.09 0.04 0.04 -0.03 -0.03

49 1 0.03 0.04 -0.03 0.12 -0.13 -0.14 -0.07 0.06 0.07

50 1 0.01 -0.02 -0.01 0.16 -0.13 -0.21 -0.13 0.05 0.14

51 1 0.01 -0.01 0.04 0.01 0.08 0.02 -0.00 -0.02 0.00

52 8 0.00 0.00 0.00 -0.00 0.02 -0.02 -0.00 -0.01 0.01

53 6 0.01 0.00 0.01 -0.01 -0.01 -0.01 0.00 0.00 0.00

54 1 0.01 -0.00 0.01 0.00 -0.02 0.01 0.00 0.01 0.00

55 1 0.01 0.00 0.01 0.01 -0.01 0.02 -0.00 -0.00 -0.01

56 1 0.00 -0.01 0.00 -0.02 -0.03 -0.03 0.01 0.01 0.01

55 56 57

A A A

Frequencies -- 702.8035 715.0931 721.5973

Red. masses -- 2.5419 4.9451 5.7424

Frc consts -- 0.7397 1.4899 1.7617

IR Inten -- 36.2748 25.5298 53.2911

Atom AN X Y Z X Y Z X Y Z

1 16 -0.01 0.02 -0.01 -0.03 -0.01 0.00 -0.01 -0.09 0.07

2 6 -0.01 -0.02 0.01 0.18 -0.07 0.07 -0.08 -0.00 -0.09

3 6 0.01 0.01 -0.01 0.05 0.00 -0.03 -0.04 -0.01 0.03

4 6 0.00 0.01 0.01 -0.00 -0.05 0.02 -0.01 0.04 -0.01

5 6 0.12 0.03 0.11 0.04 0.03 -0.00 0.08 0.11 -0.08

6 6 -0.08 -0.02 -0.08 0.02 -0.02 -0.02 0.07 -0.07 -0.09

7 6 -0.09 -0.03 -0.08 -0.02 0.02 -0.00 -0.07 0.13 0.00

8 6 0.12 0.03 0.11 0.05 -0.01 -0.00 0.11 -0.07 -0.07

9 1 -0.38 -0.10 -0.34 -0.12 -0.06 -0.10 -0.03 -0.10 0.00

10 6 0.12 0.04 0.11 0.01 0.04 0.03 -0.05 0.15 0.04

11 1 -0.37 -0.11 -0.33 -0.12 -0.04 -0.08 -0.09 -0.01 0.05

12 6 -0.09 -0.03 -0.07 -0.02 -0.03 0.02 -0.08 -0.11 0.09

13 1 -0.04 -0.01 -0.04 -0.05 0.01 -0.11 0.13 0.11 -0.14

14 1 -0.04 -0.01 -0.03 -0.07 0.02 -0.10 0.09 0.19 -0.12

15 1 -0.41 -0.12 -0.36 -0.14 -0.06 -0.09 -0.08 -0.12 0.08

16 8 0.00 -0.00 0.01 -0.00 0.02 0.02 -0.01 0.05 0.00

17 8 -0.00 -0.02 0.01 -0.02 -0.02 -0.04 -0.00 0.01 -0.03

18 6 0.00 -0.03 -0.01 0.06 0.03 0.02 -0.03 -0.04 -0.03

19 1 0.01 -0.02 -0.02 0.07 0.23 -0.00 -0.06 -0.22 0.01

20 6 -0.00 -0.01 -0.01 -0.00 0.08 -0.03 0.00 -0.05 0.03

21 6 -0.01 -0.01 0.01 0.12 0.16 0.03 -0.06 -0.10 -0.03

22 6 0.00 -0.00 0.00 -0.10 -0.02 0.01 0.06 0.02 -0.01

23 6 0.02 0.02 0.00 -0.09 -0.05 -0.01 0.08 0.01 -0.00

24 6 -0.00 -0.00 -0.01 0.03 0.04 -0.02 -0.02 -0.04 0.01

25 1 -0.01 -0.01 0.02 0.09 0.13 -0.12 -0.04 -0.07 0.11

26 6 0.01 0.00 -0.01 -0.16 -0.06 0.01 0.07 0.04 -0.00

27 6 0.00 -0.00 0.00 -0.13 -0.05 0.01 0.13 0.03 -0.01

28 1 0.03 0.02 -0.03 -0.11 -0.04 -0.07 0.11 0.00 0.03

29 6 -0.00 0.01 0.00 0.06 -0.07 0.03 -0.05 0.06 -0.03

30 1 0.02 -0.00 -0.00 -0.19 0.05 -0.14 0.13 -0.03 0.15

31 1 0.01 0.00 -0.00 -0.21 0.05 -0.09 0.11 -0.02 0.10

32 1 0.01 -0.01 -0.03 -0.13 -0.05 0.01 0.14 0.01 -0.00

33 1 -0.01 -0.00 0.01 0.11 -0.00 -0.07 -0.06 0.04 0.08

34 8 -0.02 0.00 -0.00 0.12 -0.00 -0.01 -0.11 0.00 0.01

35 6 0.00 -0.00 0.00 0.01 -0.01 -0.00 -0.01 0.01 0.00

36 1 0.01 0.03 0.01 -0.05 -0.16 -0.04 0.04 0.14 0.04

37 1 0.01 -0.02 -0.01 -0.05 0.07 0.02 0.04 -0.06 -0.02

38 1 0.01 -0.01 -0.00 -0.05 0.05 0.01 0.04 -0.05 -0.01

39 6 -0.00 -0.02 -0.00 -0.15 0.02 -0.04 0.13 0.04 0.10

40 8 0.01 0.01 -0.02 0.00 0.00 0.07 -0.06 0.00 0.01

41 8 -0.01 0.00 0.02 0.05 -0.01 -0.04 -0.04 0.00 -0.05

42 6 -0.02 0.01 0.02 0.12 0.00 -0.13 0.14 -0.01 -0.14

43 6 0.01 -0.00 -0.02 -0.08 0.01 0.04 -0.06 0.00 0.04

44 6 0.03 -0.00 -0.03 -0.12 0.03 0.14 -0.13 0.03 0.15

45 6 -0.02 0.00 0.01 0.06 -0.01 -0.08 0.06 -0.02 -0.07

46 1 0.01 -0.00 -0.03 -0.15 0.01 0.11 -0.18 0.01 0.17

47 6 -0.01 0.00 0.02 0.07 0.01 -0.03 0.06 0.02 -0.03

48 6 0.02 -0.01 -0.02 -0.08 -0.01 0.08 -0.07 -0.00 0.07

49 1 -0.03 0.02 0.03 0.11 0.02 -0.10 0.05 -0.00 -0.04

50 1 -0.06 0.02 0.07 0.30 0.01 -0.28 0.30 0.01 -0.28

51 1 -0.00 -0.00 0.00 0.01 -0.02 -0.03 -0.01 -0.01 -0.03

52 8 -0.00 -0.00 0.00 0.02 -0.01 -0.01 0.02 -0.01 -0.02

53 6 0.00 0.00 0.00 0.00 0.00 -0.00 0.00 0.00 -0.00

54 1 0.00 0.00 0.00 -0.02 -0.00 -0.02 -0.02 -0.00 -0.02

55 1 -0.00 -0.00 -0.00 -0.00 0.01 0.00 -0.00 0.01 0.00

56 1 0.00 0.00 0.00 0.02 0.01 0.00 0.02 0.02 0.01

58 59 60

A A A

Frequencies -- 729.3093 740.1968 762.1735

Red. masses -- 5.7650 4.5945 1.3638

Frc consts -- 1.8067 1.4831 0.4668

IR Inten -- 15.2607 8.4043 66.6535

Atom AN X Y Z X Y Z X Y Z

1 16 0.01 0.09 -0.06 0.01 0.07 -0.04 -0.00 -0.00 0.00

2 6 0.02 -0.09 0.07 -0.06 -0.10 -0.03 -0.00 -0.00 -0.01

3 6 0.02 -0.00 -0.08 -0.02 0.00 0.02 -0.00 -0.00 0.00

4 6 -0.05 -0.01 0.04 0.06 0.06 -0.01 0.00 0.00 -0.00

5 6 -0.05 -0.07 0.06 -0.03 -0.04 0.04 0.00 0.00 0.00

6 6 -0.06 0.05 0.06 -0.04 0.03 0.03 0.00 -0.00 -0.00

7 6 0.04 -0.10 -0.01 0.02 -0.06 -0.01 -0.00 0.00 -0.00

8 6 -0.08 0.06 0.06 -0.05 0.03 0.03 0.00 -0.00 -0.00

9 1 0.01 0.07 -0.01 0.05 0.05 0.04 0.00 -0.00 0.00

10 6 0.04 -0.11 -0.02 0.02 -0.08 -0.01 -0.00 0.00 0.00

11 1 0.05 0.00 -0.05 0.07 0.01 0.00 0.00 -0.00 0.00

12 6 0.05 0.08 -0.07 0.02 0.05 -0.05 -0.00 -0.00 0.00

13 1 -0.09 -0.08 0.11 0.01 -0.03 0.12 0.01 0.00 0.00

14 1 -0.06 -0.15 0.10 0.01 -0.08 0.11 0.00 0.00 0.00

15 1 0.05 0.09 -0.07 0.08 0.07 0.01 0.00 -0.00 0.00

16 8 0.02 -0.03 0.01 -0.01 -0.03 0.00 -0.00 0.00 0.00

17 8 0.01 -0.01 0.03 0.02 -0.00 0.04 0.00 0.00 -0.00

18 6 0.02 -0.06 0.02 -0.04 -0.13 0.01 -0.00 -0.00 0.00

19 1 0.10 0.02 -0.14 0.05 -0.19 -0.20 0.03 -0.01 -0.06

20 6 0.03 0.00 -0.03 -0.05 -0.02 -0.03 -0.00 -0.00 -0.01

21 6 0.06 -0.02 0.03 -0.08 -0.03 -0.01 -0.00 -0.00 0.00

22 6 -0.01 0.05 0.03 0.01 -0.04 0.05 0.00 -0.00 0.01

23 6 0.14 -0.15 -0.04 -0.02 0.20 0.07 0.00 0.00 0.00

24 6 0.01 -0.09 0.00 -0.02 0.06 -0.05 -0.00 -0.00 -0.00

25 1 0.01 0.03 -0.06 -0.01 -0.04 0.26 -0.00 0.00 0.01

26 6 -0.14 0.07 0.00 0.13 -0.05 -0.04 0.00 -0.00 -0.00

27 6 0.25 -0.03 -0.01 -0.13 0.03 0.01 0.00 0.00 -0.00

28 1 0.17 -0.15 -0.06 0.01 0.22 -0.09 0.00 0.00 0.00

29 6 -0.09 0.10 0.02 0.08 -0.06 0.00 0.00 0.00 0.00

30 1 0.07 -0.12 -0.12 0.00 0.11 0.20 0.01 -0.00 -0.01

31 1 -0.14 0.06 -0.10 0.13 -0.03 0.10 0.00 -0.00 -0.02

32 1 0.28 -0.11 0.00 -0.13 0.02 -0.22 0.00 0.00 -0.01

33 1 0.01 0.24 -0.11 -0.00 -0.18 0.25 -0.00 -0.00 -0.01

34 8 -0.21 0.01 0.00 0.08 -0.01 0.00 -0.00 -0.00 0.00

35 6 -0.02 0.06 0.02 0.01 -0.04 -0.01 0.00 0.00 0.00

36 1 0.06 0.29 0.07 -0.01 -0.09 -0.03 0.00 0.00 0.00

37 1 0.06 -0.07 -0.03 -0.01 -0.00 0.00 0.00 -0.00 -0.00

38 1 0.07 -0.04 -0.01 -0.01 -0.01 -0.00 0.00 -0.00 -0.00

39 6 -0.02 -0.03 0.02 0.07 -0.06 0.08 -0.00 0.00 0.02

40 8 -0.00 0.02 0.01 -0.02 0.03 -0.03 -0.01 0.00 0.02

41 8 -0.01 -0.01 -0.01 -0.04 -0.04 0.01 0.00 -0.03 -0.03

42 6 0.07 0.03 -0.09 0.06 0.04 -0.10 -0.00 0.00 -0.02

43 6 -0.07 0.01 -0.02 -0.09 0.00 -0.07 -0.05 -0.00 0.00

44 6 -0.05 0.04 0.10 -0.05 0.06 0.12 0.08 0.01 -0.06

45 6 0.02 0.00 -0.06 0.01 0.01 -0.07 -0.06 0.01 0.05

46 1 -0.15 0.00 0.06 -0.24 0.01 0.07 0.24 -0.03 -0.28

47 6 0.07 0.05 0.02 0.10 0.10 0.04 -0.02 0.05 0.06

48 6 -0.04 -0.03 0.04 -0.03 -0.03 0.03 -0.04 0.01 0.04

49 1 0.01 0.07 -0.01 -0.06 0.12 0.07 0.47 -0.04 -0.44

50 1 0.17 0.10 -0.13 0.15 0.18 -0.09 0.25 0.04 -0.23

51 1 -0.03 -0.03 -0.03 -0.10 -0.02 -0.02 0.35 -0.04 -0.40

52 8 0.02 -0.03 0.00 0.03 -0.07 0.01 -0.01 -0.03 0.02

53 6 0.01 0.00 0.00 0.01 0.01 0.01 0.01 0.00 0.01

54 1 -0.02 0.01 -0.03 -0.04 0.02 -0.04 -0.01 0.00 -0.00

55 1 -0.02 0.02 -0.01 -0.03 0.02 -0.03 -0.02 0.01 -0.01

56 1 0.04 0.04 0.03 0.07 0.08 0.06 0.04 0.03 0.01

61 62 63

A A A

Frequencies -- 767.4727 771.3748 779.5673

Red. masses -- 1.4811 1.4174 4.5431

Frc consts -- 0.5140 0.4969 1.6267

IR Inten -- 15.8229 22.0427 9.0284

Atom AN X Y Z X Y Z X Y Z

1 16 -0.01 0.00 -0.01 -0.01 0.01 -0.01 0.00 -0.02 0.01

2 6 -0.00 0.01 -0.00 0.02 -0.02 0.03 -0.05 0.09 0.03

3 6 0.00 0.00 -0.00 0.01 -0.00 -0.02 0.01 0.05 -0.17

4 6 0.00 -0.00 0.03 -0.01 -0.00 -0.05 -0.01 -0.05 0.13

5 6 0.10 0.03 0.09 0.04 0.01 0.03 -0.01 -0.00 -0.02

6 6 -0.05 -0.01 -0.05 -0.02 -0.00 -0.02 0.01 -0.00 0.00

7 6 -0.05 -0.01 -0.04 -0.02 -0.01 -0.01 0.00 0.01 0.01

8 6 -0.01 -0.01 -0.02 -0.01 -0.00 -0.00 0.01 -0.00 -0.00

9 1 0.17 0.04 0.15 0.06 0.02 0.05 -0.02 -0.01 -0.02

10 6 -0.01 0.00 -0.01 -0.00 -0.00 -0.00 -0.00 0.01 0.00

11 1 0.13 0.04 0.12 0.04 0.01 0.04 -0.02 -0.00 -0.01

12 6 -0.07 -0.02 -0.05 -0.02 -0.01 -0.02 0.01 -0.00 0.01

13 1 0.39 0.11 0.33 0.13 0.03 0.12 -0.04 -0.01 -0.06

14 1 0.37 0.12 0.32 0.13 0.04 0.12 -0.04 0.00 -0.06

15 1 0.34 0.09 0.31 0.12 0.03 0.11 -0.05 -0.02 -0.04

16 8 0.00 0.00 0.00 0.00 -0.00 0.00 0.00 0.00 -0.01

17 8 -0.01 -0.02 -0.00 -0.01 -0.01 -0.00 0.00 0.01 -0.00

18 6 0.01 -0.00 0.00 0.01 0.01 -0.01 0.05 0.03 0.01

19 1 0.02 0.00 -0.00 -0.01 0.04 0.04 0.13 0.06 -0.15

20 6 0.00 -0.00 -0.01 -0.00 0.02 0.06 -0.01 0.02 0.26

21 6 0.00 0.00 0.01 0.01 0.00 -0.03 -0.02 -0.03 -0.07

22 6 -0.00 -0.00 -0.02 -0.01 0.01 0.02 0.02 0.01 -0.19

23 6 -0.00 0.01 -0.02 -0.00 -0.04 0.04 -0.03 -0.03 -0.07

24 6 -0.00 0.00 0.02 0.01 -0.00 -0.04 0.02 0.01 0.08

25 1 -0.02 -0.02 -0.12 0.04 0.06 0.20 -0.02 -0.13 -0.30

26 6 0.01 0.00 0.03 -0.04 -0.01 -0.06 0.01 -0.00 0.02

27 6 -0.01 -0.00 -0.01 0.02 -0.00 0.02 0.01 0.02 0.06

28 1 0.01 -0.01 0.08 -0.04 -0.00 -0.20 -0.09 -0.02 -0.10

29 6 -0.00 0.00 0.02 0.00 -0.01 -0.06 -0.01 -0.02 -0.10

30 1 -0.02 -0.03 -0.17 0.05 0.07 0.43 0.03 0.00 0.07

31 1 0.00 -0.00 -0.15 -0.03 0.02 0.45 0.04 -0.01 0.45

32 1 -0.00 -0.01 0.12 0.00 0.03 -0.25 -0.01 0.08 0.15

33 1 -0.02 -0.02 -0.21 0.07 0.08 0.54 0.00 -0.00 0.17

34 8 0.00 0.00 -0.01 -0.01 -0.00 0.01 0.02 0.00 -0.02

35 6 0.00 -0.00 -0.00 -0.00 0.01 0.00 0.00 -0.00 -0.00

36 1 -0.00 -0.01 0.00 -0.00 0.01 -0.01 -0.01 -0.04 -0.01

37 1 -0.00 -0.00 -0.00 -0.00 0.01 0.00 -0.01 0.00 0.00

38 1 -0.00 0.00 -0.00 0.00 0.00 0.00 -0.01 0.02 0.00

39 6 -0.02 -0.01 -0.01 -0.02 0.01 -0.02 -0.08 -0.05 -0.01

40 8 0.01 0.00 0.00 0.00 -0.00 0.01 0.02 0.01 0.02

41 8 0.00 -0.01 0.00 0.00 0.02 0.00 0.03 -0.11 -0.01

42 6 -0.00 0.00 -0.00 0.01 -0.00 0.01 -0.08 -0.01 -0.01

43 6 -0.01 -0.00 -0.01 0.02 0.01 0.02 -0.09 -0.04 -0.10

44 6 0.00 0.01 0.01 -0.00 -0.01 -0.01 0.02 0.05 0.02

45 6 -0.00 0.00 -0.00 -0.00 0.00 0.01 0.01 -0.00 -0.00

46 1 -0.02 0.00 -0.00 0.04 -0.01 0.02 -0.14 0.05 -0.13

47 6 0.01 0.01 0.00 -0.02 -0.03 -0.01 0.07 0.14 0.04

48 6 0.00 0.00 -0.00 -0.00 -0.01 0.01 0.02 0.07 -0.03

49 1 -0.01 0.01 0.01 0.03 -0.02 -0.04 -0.07 0.06 0.11

50 1 -0.00 0.02 0.01 0.01 -0.06 -0.03 -0.07 0.22 0.13

51 1 -0.02 0.00 0.00 0.06 -0.01 -0.01 -0.22 0.09 -0.00

52 8 0.00 -0.01 0.00 -0.01 0.02 -0.01 0.03 -0.11 0.04

53 6 0.00 0.00 0.00 -0.01 -0.00 -0.01 0.04 0.01 0.04

54 1 -0.00 0.00 -0.00 0.01 -0.01 0.01 -0.03 0.04 -0.04

55 1 -0.00 0.00 -0.00 0.01 -0.01 0.01 -0.03 0.03 -0.03

56 1 0.01 0.01 0.01 -0.03 -0.03 -0.03 0.12 0.12 0.13

64 65 66

A A A

Frequencies -- 784.9328 820.1298 825.8355

Red. masses -- 4.5919 1.8316 3.8794

Frc consts -- 1.6669 0.7258 1.5588

IR Inten -- 16.4784 54.2808 28.8432

Atom AN X Y Z X Y Z X Y Z

1 16 0.01 0.04 -0.02 -0.00 0.00 -0.00 -0.00 0.02 -0.01

2 6 -0.03 -0.11 0.05 0.01 -0.03 0.02 0.03 -0.09 0.05

3 6 0.03 0.02 -0.14 0.05 -0.01 -0.04 0.08 -0.02 0.02

4 6 0.02 -0.01 0.16 0.01 -0.01 0.11 0.01 0.03 -0.05

5 6 -0.02 -0.01 -0.00 0.00 0.00 0.00 0.01 0.00 0.01

6 6 -0.01 0.01 0.02 -0.00 0.00 0.00 -0.00 0.00 0.00

7 6 0.01 -0.02 0.00 0.00 -0.00 -0.00 0.00 -0.01 -0.00

8 6 -0.02 0.01 0.01 -0.00 0.00 0.00 -0.01 0.00 0.00

9 1 -0.01 0.01 -0.00 -0.00 0.00 -0.00 -0.00 0.00 0.00

10 6 0.01 -0.03 -0.00 0.00 -0.00 -0.00 0.00 -0.01 -0.00

11 1 0.00 -0.01 -0.01 -0.00 -0.00 -0.00 0.00 -0.00 0.00

12 6 0.02 0.02 -0.01 -0.00 0.00 -0.00 -0.00 0.00 -0.01

13 1 -0.05 -0.03 0.00 0.00 -0.00 0.01 0.01 -0.00 0.02

14 1 -0.04 -0.04 -0.00 0.00 -0.00 0.01 0.01 -0.01 0.03

15 1 -0.02 0.01 -0.04 0.01 0.00 0.00 0.02 0.01 0.01

16 8 -0.00 -0.02 0.01 0.00 -0.00 0.00 0.00 -0.01 0.01

17 8 0.01 -0.00 0.02 -0.00 -0.01 -0.00 -0.00 -0.02 -0.00

18 6 -0.02 -0.08 -0.03 -0.05 0.01 -0.03 -0.12 0.01 -0.09

19 1 -0.09 -0.14 0.11 -0.08 -0.02 0.02 -0.24 -0.08 0.16

20 6 -0.01 0.01 0.18 0.02 0.01 0.01 0.07 0.01 0.03

21 6 -0.03 -0.01 -0.06 0.01 0.02 -0.02 0.06 0.05 -0.03

22 6 -0.01 -0.02 -0.17 -0.01 -0.00 0.09 -0.02 -0.03 -0.08

23 6 0.00 0.08 -0.07 -0.01 0.02 -0.12 0.02 0.02 0.08

24 6 0.01 0.04 0.08 -0.01 0.00 -0.06 -0.01 -0.01 0.05

25 1 -0.03 -0.11 -0.29 0.03 0.10 0.22 0.01 0.10 -0.15

26 6 0.02 -0.01 0.06 -0.03 -0.01 -0.02 -0.04 0.02 0.03

27 6 -0.04 -0.00 0.04 -0.03 -0.01 -0.08 -0.07 -0.09 0.06

28 1 0.04 0.06 0.03 0.09 -0.10 0.69 0.10 0.09 -0.50

29 6 0.02 -0.03 -0.05 0.01 -0.00 -0.00 -0.04 0.03 -0.00

30 1 -0.04 0.01 -0.15 0.01 0.07 0.32 -0.07 -0.04 -0.20

31 1 0.02 -0.00 0.11 -0.04 0.01 -0.04 -0.05 0.03 0.02

32 1 -0.01 -0.05 0.24 0.02 -0.08 0.43 -0.07 -0.13 -0.29

33 1 -0.02 -0.08 -0.15 0.04 0.04 0.22 -0.03 0.05 -0.17

34 8 0.01 0.00 -0.02 -0.00 0.00 -0.02 -0.01 -0.01 0.01

35 6 0.00 -0.01 -0.00 0.00 0.01 -0.00 -0.00 0.01 0.01

36 1 0.00 -0.01 0.00 0.01 0.00 0.03 -0.00 0.03 -0.02

37 1 0.00 -0.01 -0.01 0.01 -0.00 -0.01 0.00 0.00 0.00

38 1 -0.00 0.00 -0.00 -0.01 0.01 -0.01 0.01 -0.00 0.00

39 6 0.09 0.01 0.02 0.06 0.04 0.02 0.17 0.11 0.05

40 8 -0.01 0.02 -0.04 -0.02 -0.01 0.00 -0.05 -0.02 -0.00

41 8 -0.06 0.12 0.06 -0.00 0.01 -0.02 -0.02 0.07 -0.02

42 6 0.12 0.01 -0.02 -0.02 -0.01 -0.00 -0.05 -0.04 -0.03

43 6 0.08 0.05 0.10 -0.01 -0.03 -0.01 -0.04 -0.08 -0.03

44 6 -0.02 -0.04 -0.00 -0.01 -0.01 -0.01 -0.05 -0.03 -0.04

45 6 -0.02 0.02 -0.01 0.03 -0.03 0.03 0.10 -0.09 0.11

46 1 0.11 -0.05 0.16 -0.02 0.01 -0.04 -0.08 0.05 -0.10

47 6 -0.07 -0.14 -0.02 -0.01 0.01 -0.02 -0.06 0.02 -0.06

48 6 -0.03 -0.09 0.05 0.01 0.05 -0.01 0.01 0.18 -0.02

49 1 0.13 -0.04 -0.18 0.01 -0.06 0.03 0.03 -0.19 0.09

50 1 0.17 -0.22 -0.24 -0.02 -0.02 0.02 -0.02 -0.10 0.01

51 1 0.33 -0.13 -0.07 -0.04 0.06 -0.02 -0.10 0.18 -0.09

52 8 -0.03 0.12 -0.04 0.01 -0.03 0.01 0.02 -0.09 0.03

53 6 -0.04 -0.01 -0.04 0.02 0.01 0.02 0.05 0.02 0.05

54 1 0.03 -0.04 0.04 0.00 0.01 0.00 0.01 0.03 0.01

55 1 0.03 -0.03 0.04 0.00 0.01 0.00 0.01 0.03 0.01

56 1 -0.13 -0.13 -0.14 0.03 0.03 0.03 0.10 0.08 0.10

67 68 69

A A A

Frequencies -- 835.9162 857.6856 861.5635

Red. masses -- 3.0701 1.9703 1.2456

Frc consts -- 1.2639 0.8539 0.5448

IR Inten -- 22.9830 2.3984 0.4779

Atom AN X Y Z X Y Z X Y Z

1 16 0.00 -0.00 -0.00 0.00 -0.01 0.01 0.00 -0.00 0.00

2 6 -0.02 0.04 0.02 -0.04 0.05 -0.02 0.00 -0.00 -0.00

3 6 -0.06 0.01 0.02 0.03 0.02 0.01 -0.00 -0.00 -0.00

4 6 -0.01 -0.02 -0.01 0.01 0.03 0.00 -0.00 0.00 0.00

5 6 -0.01 -0.00 -0.01 -0.00 -0.00 0.00 -0.00 -0.00 0.00

6 6 0.00 0.00 0.00 -0.00 -0.00 -0.00 -0.06 -0.02 -0.05

7 6 -0.00 0.00 0.00 0.00 0.00 0.00 0.05 0.02 0.05

8 6 -0.00 0.00 0.00 0.00 -0.00 -0.00 -0.05 -0.01 -0.04

9 1 -0.00 0.00 -0.00 0.02 0.00 0.01 0.40 0.10 0.35

10 6 0.00 -0.00 -0.00 0.00 0.00 0.00 0.05 0.02 0.05

11 1 0.00 0.00 0.00 -0.01 -0.00 -0.01 -0.33 -0.09 -0.30

12 6 0.00 0.00 0.00 -0.00 -0.00 0.00 0.01 0.00 0.01

13 1 -0.02 -0.01 -0.01 0.01 0.00 0.00 0.32 0.09 0.29

14 1 -0.00 -0.00 -0.01 -0.01 0.00 -0.02 -0.38 -0.11 -0.34

15 1 -0.01 -0.01 -0.01 -0.01 -0.00 -0.00 -0.04 -0.01 -0.04

16 8 0.01 -0.01 -0.01 -0.00 0.00 -0.00 0.00 0.00 -0.00

17 8 0.00 0.01 0.00 0.00 -0.00 0.00 0.00 0.00 -0.00

18 6 0.12 -0.01 -0.04 -0.05 0.02 0.01 -0.00 -0.00 0.00

19 1 -0.16 0.02 0.53 0.04 -0.02 -0.16 0.00 0.00 -0.01

20 6 -0.05 -0.01 -0.03 0.06 0.02 0.01 -0.00 -0.00 -0.00

21 6 -0.04 -0.03 0.02 0.07 0.04 -0.00 -0.00 -0.00 0.00

22 6 0.01 0.02 0.02 0.01 -0.02 0.01 -0.00 0.00 -0.00

23 6 -0.01 -0.01 -0.01 -0.01 -0.01 -0.00 0.00 -0.00 0.00

24 6 0.01 0.01 -0.02 -0.01 -0.07 0.01 0.00 0.00 -0.00

25 1 -0.02 -0.10 0.02 0.02 0.13 -0.07 -0.00 -0.00 0.00

26 6 0.02 -0.01 -0.00 0.00 0.03 -0.01 -0.00 -0.00 0.00

27 6 0.06 0.06 -0.01 -0.08 -0.07 -0.00 0.00 0.00 -0.00

28 1 -0.09 -0.01 0.07 0.11 -0.03 0.00 -0.00 0.00 -0.00

29 6 0.04 -0.02 0.01 -0.07 0.05 -0.01 0.00 -0.00 0.00

30 1 0.02 0.02 0.02 -0.01 -0.06 0.03 0.00 0.00 -0.00

31 1 0.02 -0.01 -0.03 0.02 0.00 0.06 -0.00 0.00 -0.00

32 1 0.04 0.10 0.04 -0.07 -0.09 0.00 0.00 0.00 -0.00

33 1 0.02 -0.05 0.00 -0.03 0.11 0.05 0.00 -0.00 -0.00

34 8 0.00 0.00 0.00 0.02 0.00 0.00 -0.00 -0.00 -0.00

35 6 0.00 -0.01 -0.00 -0.00 -0.02 -0.00 0.00 0.00 0.00

36 1 -0.00 -0.01 -0.00 -0.00 -0.03 -0.01 0.00 0.00 -0.00

37 1 -0.00 0.00 -0.00 -0.00 -0.01 -0.00 0.00 -0.00 -0.00

38 1 -0.00 -0.00 -0.00 -0.00 -0.01 -0.00 -0.00 0.00 -0.00

39 6 -0.10 -0.06 -0.09 0.02 -0.03 0.01 -0.00 0.00 -0.00

40 8 0.07 0.02 -0.07 0.01 -0.01 -0.02 0.00 -0.00 0.00

41 8 -0.04 0.09 0.17 -0.01 -0.04 0.01 0.00 0.00 -0.00

42 6 0.02 -0.03 -0.08 -0.03 0.01 0.04 0.00 0.00 -0.00

43 6 -0.01 -0.06 -0.06 0.06 0.01 -0.05 -0.00 -0.00 0.00

44 6 -0.03 -0.02 -0.04 0.06 0.01 -0.03 -0.00 -0.00 -0.00

45 6 0.08 -0.05 0.05 0.01 0.02 -0.07 0.00 -0.00 0.00

46 1 -0.34 0.09 0.15 -0.31 0.04 0.31 0.00 -0.00 -0.00

47 6 -0.09 0.01 0.00 -0.06 0.02 0.10 -0.00 -0.00 -0.00

48 6 -0.02 0.12 0.02 -0.02 -0.04 0.02 0.00 0.00 -0.00

49 1 -0.03 -0.08 0.12 -0.29 0.09 0.24 0.01 -0.00 -0.00

50 1 0.32 -0.13 -0.32 0.48 -0.01 -0.46 -0.01 -0.00 0.01

51 1 0.15 0.09 -0.24 0.13 -0.05 -0.10 -0.00 0.00 0.00

52 8 0.01 -0.06 0.02 -0.01 0.01 0.01 0.00 -0.00 0.00

53 6 0.03 0.01 0.03 -0.01 -0.00 -0.01 0.00 0.00 0.00

54 1 0.01 0.01 0.01 -0.01 -0.01 -0.00 0.00 0.00 0.00

55 1 0.01 0.02 0.01 -0.01 0.00 -0.00 0.00 0.00 0.00

56 1 0.07 0.05 0.05 0.00 -0.01 -0.03 0.00 0.00 0.00

70 71 72

A A A

Frequencies -- 869.1543 885.2644 890.6259

Red. masses -- 2.8478 1.6491 1.8859

Frc consts -- 1.2675 0.7615 0.8814

IR Inten -- 16.0101 4.3005 8.0426

Atom AN X Y Z X Y Z X Y Z

1 16 0.00 -0.00 -0.00 0.00 -0.00 0.01 -0.00 0.00 -0.01

2 6 -0.09 0.07 -0.00 0.01 -0.00 -0.04 0.00 -0.02 0.05

3 6 0.01 0.04 0.03 -0.02 -0.01 0.01 0.00 -0.04 0.02

4 6 0.01 0.04 0.01 0.00 -0.00 0.01 0.01 -0.02 -0.00

5 6 -0.01 -0.00 -0.01 0.00 0.00 0.00 0.00 0.00 -0.00

6 6 -0.00 0.00 -0.00 0.00 -0.00 -0.00 -0.00 0.00 0.00

7 6 -0.00 0.00 0.00 0.00 -0.00 -0.00 -0.00 0.00 0.00

8 6 -0.00 -0.00 0.00 0.00 -0.00 -0.00 -0.00 0.00 0.00

9 1 0.01 0.00 0.01 -0.01 -0.00 -0.01 0.00 0.00 0.01

10 6 0.00 0.00 0.00 -0.00 0.00 -0.00 0.00 -0.00 0.00

11 1 -0.00 0.00 -0.00 0.01 0.00 0.00 -0.01 -0.00 -0.00

12 6 0.00 0.00 0.00 -0.00 -0.00 0.00 0.00 0.00 -0.00

13 1 -0.01 -0.00 -0.00 -0.00 0.00 -0.01 0.00 -0.00 0.01

14 1 -0.01 -0.00 -0.02 0.01 0.00 0.00 -0.01 -0.00 -0.01

15 1 -0.02 -0.01 -0.02 -0.00 0.00 0.00 0.00 -0.00 -0.00

16 8 0.00 -0.01 -0.01 -0.00 0.01 0.00 0.01 -0.01 -0.00

17 8 0.01 0.01 0.01 -0.00 -0.00 -0.00 0.00 0.00 0.00

18 6 0.04 -0.02 -0.08 -0.03 -0.01 0.11 0.05 0.01 -0.13

19 1 -0.19 -0.11 0.39 0.25 0.03 -0.46 -0.29 -0.07 0.55

20 6 0.07 0.03 -0.01 -0.01 -0.02 -0.05 -0.01 -0.03 -0.05

21 6 0.10 0.04 0.01 0.01 0.01 0.05 -0.02 0.01 0.04

22 6 0.03 -0.03 0.04 0.01 -0.01 0.06 -0.01 -0.00 0.05

23 6 -0.01 -0.01 -0.01 0.00 0.02 -0.02 0.01 0.03 -0.01

24 6 -0.02 -0.11 0.02 0.01 0.01 0.07 0.01 0.06 0.06

25 1 0.01 0.16 -0.15 -0.04 -0.11 -0.39 -0.04 -0.12 -0.30

26 6 0.03 0.05 -0.04 0.01 -0.00 -0.09 -0.02 -0.02 -0.07

27 6 -0.10 -0.10 -0.01 -0.00 0.00 -0.01 0.02 0.02 -0.01

28 1 0.15 -0.04 0.06 0.03 -0.01 0.18 -0.01 0.01 0.14

29 6 -0.10 0.07 -0.02 -0.02 -0.00 -0.04 0.01 -0.02 -0.03

30 1 0.01 -0.12 -0.01 -0.04 -0.06 -0.41 -0.06 -0.01 -0.40

31 1 0.07 -0.01 0.17 0.04 0.01 0.48 -0.01 0.02 0.40

32 1 -0.10 -0.11 0.03 0.00 -0.00 -0.02 0.03 -0.00 -0.01

33 1 -0.04 0.15 0.13 -0.02 0.00 0.22 -0.00 -0.04 0.16

34 8 0.03 0.01 0.00 -0.00 0.00 -0.00 -0.02 -0.01 -0.00

35 6 -0.00 -0.04 -0.01 0.00 0.00 0.00 -0.00 0.03 0.01

36 1 -0.01 -0.06 -0.01 0.00 0.00 0.01 0.01 0.04 0.01

37 1 -0.01 -0.02 -0.01 0.00 -0.00 -0.00 0.01 0.01 0.00

38 1 -0.01 -0.03 -0.01 -0.00 0.00 -0.00 0.00 0.01 0.00

39 6 -0.03 -0.08 -0.01 0.00 -0.01 -0.02 0.02 0.04 0.04

40 8 0.03 0.02 -0.06 0.02 -0.02 -0.02 -0.04 0.02 0.04

41 8 -0.05 -0.02 0.11 -0.01 0.07 0.01 0.02 -0.07 -0.05

42 6 0.06 -0.01 -0.05 0.01 0.01 -0.02 -0.03 -0.01 0.03

43 6 -0.04 0.02 0.02 -0.01 -0.02 0.00 0.01 0.02 0.01

44 6 -0.04 0.01 0.05 -0.02 -0.01 -0.01 0.03 0.01 -0.00

45 6 -0.06 0.03 0.01 0.02 -0.03 0.04 -0.01 0.02 -0.04

46 1 0.15 -0.01 -0.17 -0.00 0.00 -0.02 0.03 -0.00 0.00

47 6 0.08 0.00 -0.05 -0.02 -0.02 -0.03 0.01 0.02 0.04

48 6 -0.00 -0.02 0.01 0.00 0.03 0.00 0.00 -0.03 -0.01

49 1 0.26 0.03 -0.27 0.04 -0.06 -0.00 -0.11 0.06 0.07

50 1 -0.35 0.08 0.35 -0.04 -0.06 0.03 0.11 0.05 -0.09

51 1 0.01 -0.02 0.02 0.00 0.03 -0.02 -0.04 -0.03 0.04

52 8 0.01 0.00 -0.01 0.00 -0.01 0.00 -0.00 0.01 0.00

53 6 -0.01 -0.00 -0.01 0.01 0.00 0.01 -0.01 -0.00 -0.01

54 1 -0.00 0.00 -0.01 0.01 0.00 0.01 -0.01 -0.00 -0.01

55 1 -0.00 -0.01 -0.00 0.01 0.00 0.01 -0.01 -0.00 -0.01

56 1 -0.02 -0.00 0.01 0.01 0.01 0.01 -0.00 -0.00 -0.01

73 74 75

A A A

Frequencies -- 930.4873 942.4673 945.2923

Red. masses -- 3.3919 1.3425 1.7944

Frc consts -- 1.7303 0.7026 0.9447

IR Inten -- 21.9900 1.2812 5.3746

Atom AN X Y Z X Y Z X Y Z

1 16 -0.00 -0.01 0.01 -0.00 0.00 -0.00 -0.00 -0.01 0.00

2 6 -0.01 0.10 0.00 -0.01 0.01 -0.00 -0.03 0.05 -0.01

3 6 0.13 -0.11 0.02 0.01 -0.01 0.00 0.05 -0.06 0.01

4 6 0.03 -0.08 -0.04 0.01 -0.01 -0.00 0.02 -0.04 -0.02

5 6 0.00 -0.00 0.01 0.03 0.01 0.03 -0.01 -0.00 -0.00

6 6 0.00 0.00 0.00 -0.07 -0.01 -0.07 0.01 0.00 0.01

7 6 0.00 0.00 0.00 -0.06 -0.02 -0.06 0.01 0.00 0.01

8 6 0.00 -0.00 -0.01 0.02 0.00 0.02 0.00 -0.00 -0.01

9 1 -0.02 -0.00 -0.02 0.43 0.12 0.37 -0.06 -0.01 -0.05

10 6 -0.00 0.01 0.00 -0.01 0.00 -0.01 -0.00 0.01 0.00

11 1 -0.02 -0.01 -0.02 0.39 0.11 0.35 -0.05 -0.01 -0.05

12 6 -0.01 -0.01 -0.00 0.07 0.01 0.07 -0.01 -0.00 -0.01

13 1 0.02 0.01 0.00 -0.15 -0.05 -0.13 0.02 0.01 0.01

14 1 0.00 0.01 -0.01 0.03 0.01 0.01 -0.00 0.00 -0.01

15 1 0.02 0.00 0.02 -0.40 -0.12 -0.35 0.05 0.01 0.05

16 8 0.01 0.00 -0.01 0.00 -0.00 -0.00 0.01 -0.00 -0.01

17 8 -0.01 -0.01 -0.01 -0.00 -0.00 0.00 0.00 0.00 0.00

18 6 -0.09 0.10 -0.01 0.00 -0.00 0.00 -0.01 0.02 0.01

19 1 -0.02 0.05 -0.15 0.01 -0.01 -0.01 0.03 -0.01 -0.06

20 6 0.07 -0.07 0.01 0.01 -0.01 0.00 0.04 -0.03 0.01

21 6 -0.03 0.03 -0.00 0.00 0.01 -0.00 0.00 0.02 -0.00

22 6 -0.03 -0.00 -0.01 -0.00 -0.00 -0.00 -0.01 -0.01 -0.00

23 6 0.01 0.03 0.02 0.00 0.01 0.00 0.01 0.03 0.01

24 6 0.00 0.13 -0.01 -0.00 0.01 -0.00 -0.00 0.05 -0.01

25 1 -0.02 0.02 -0.01 -0.00 0.01 -0.00 -0.01 0.05 0.01

26 6 -0.10 -0.04 0.01 -0.01 -0.00 0.00 -0.04 -0.01 0.01

27 6 0.01 0.02 -0.00 -0.00 -0.00 -0.00 -0.01 -0.00 -0.01

28 1 -0.06 0.05 -0.03 0.00 0.01 -0.00 -0.00 0.04 -0.04

29 6 0.05 -0.04 -0.00 0.00 -0.00 -0.00 0.01 -0.01 -0.01

30 1 -0.10 0.14 -0.06 -0.01 0.01 0.01 -0.02 0.06 0.02

31 1 -0.14 0.05 -0.02 -0.01 0.00 -0.02 -0.05 0.01 -0.07

32 1 0.04 -0.06 0.01 0.00 -0.02 0.00 0.01 -0.06 0.04

33 1 0.03 -0.07 0.02 -0.00 -0.00 0.01 0.00 -0.02 0.05

34 8 -0.07 -0.05 -0.01 -0.01 -0.01 -0.00 -0.03 -0.03 -0.01

35 6 0.00 0.13 0.03 -0.00 0.01 0.00 0.00 0.06 0.02

36 1 0.01 0.16 0.04 0.00 0.02 0.01 0.01 0.08 0.02

37 1 0.02 0.06 0.02 -0.00 0.01 0.00 0.01 0.03 0.01

38 1 0.02 0.09 0.02 0.01 0.01 0.00 0.01 0.05 0.01

39 6 0.08 -0.03 -0.02 -0.01 -0.00 -0.00 0.00 -0.04 -0.01

40 8 0.04 -0.04 -0.08 0.00 0.00 -0.00 0.02 -0.01 -0.04

41 8 -0.06 -0.07 0.11 -0.00 0.00 0.00 -0.03 0.00 0.06

42 6 0.00 -0.03 0.00 0.00 0.00 -0.00 0.04 -0.01 -0.04

43 6 0.06 0.00 -0.07 -0.01 0.00 0.01 -0.09 0.01 0.08

44 6 -0.01 0.01 0.03 0.00 -0.00 -0.00 0.01 -0.00 -0.01

45 6 -0.07 0.04 0.00 0.00 -0.00 -0.00 0.02 0.01 -0.04

46 1 -0.42 0.08 0.37 0.04 -0.01 -0.03 0.51 -0.08 -0.49

47 6 0.07 0.02 -0.02 -0.00 -0.00 0.00 -0.04 0.01 0.05

48 6 -0.05 -0.02 0.04 0.00 0.00 -0.00 0.05 -0.01 -0.05

49 1 0.19 0.04 -0.23 -0.01 0.00 0.01 -0.17 0.04 0.15

50 1 -0.23 0.09 0.25 0.02 -0.00 -0.02 0.26 -0.03 -0.25

51 1 0.27 -0.05 -0.23 -0.02 0.00 0.02 -0.29 0.04 0.30

52 8 0.01 0.01 -0.00 -0.00 -0.00 0.00 -0.00 0.00 0.00

53 6 -0.01 -0.00 -0.01 0.00 0.00 0.00 -0.00 -0.00 -0.00

54 1 -0.00 -0.00 -0.01 -0.00 -0.00 0.00 -0.01 -0.00 -0.00

55 1 -0.01 -0.01 -0.01 0.00 0.00 0.00 0.00 0.00 0.01

56 1 -0.01 -0.00 0.00 0.00 -0.00 -0.00 0.00 -0.00 -0.01

76 77 78

A A A

Frequencies -- 955.7308 965.8465 974.8935

Red. masses -- 1.3084 3.4533 1.2458

Frc consts -- 0.7042 1.8980 0.6976

IR Inten -- 1.9794 46.5089 0.0511

Atom AN X Y Z X Y Z X Y Z

1 16 0.00 0.00 -0.00 -0.01 -0.00 0.00 -0.00 -0.00 0.00

2 6 -0.00 -0.01 -0.00 -0.12 0.02 -0.04 -0.00 0.00 -0.00

3 6 -0.01 0.01 -0.00 -0.04 -0.13 -0.01 0.00 -0.00 -0.00

4 6 -0.00 0.00 0.00 0.05 -0.09 -0.01 0.00 -0.00 -0.00

5 6 -0.00 0.00 -0.00 -0.01 -0.01 -0.01 -0.00 -0.00 0.00

6 6 0.00 -0.00 0.00 0.01 0.00 0.01 0.00 0.00 0.00

7 6 -0.00 -0.00 0.00 0.00 0.00 0.00 0.00 0.00 -0.00

8 6 -0.00 0.00 0.00 0.00 -0.00 -0.01 0.00 -0.00 -0.00

9 1 -0.00 -0.00 -0.00 -0.05 -0.01 -0.04 -0.00 -0.00 -0.00

10 6 0.00 -0.00 -0.00 0.00 0.01 0.00 -0.00 0.00 0.00

11 1 -0.00 -0.00 -0.00 -0.03 -0.01 -0.03 -0.00 -0.00 -0.00

12 6 0.00 0.00 -0.00 -0.01 -0.00 -0.01 -0.00 -0.00 0.00

13 1 -0.00 -0.00 0.00 0.02 0.01 0.01 0.00 0.00 -0.00

14 1 0.00 -0.00 0.00 -0.02 0.00 -0.02 -0.00 0.00 -0.00

15 1 0.00 0.00 0.00 0.04 0.01 0.04 0.00 -0.00 0.00

16 8 -0.00 -0.00 0.00 0.01 -0.01 -0.02 0.00 -0.00 -0.00

17 8 0.00 0.00 0.00 0.02 0.03 0.02 0.00 0.00 0.00

18 6 0.00 -0.01 0.00 0.15 -0.09 0.04 0.00 -0.00 0.00

19 1 0.00 -0.01 0.00 0.11 -0.16 0.10 0.00 -0.00 -0.00

20 6 0.00 -0.00 -0.01 0.04 -0.03 0.02 0.00 0.00 0.00

21 6 0.00 0.00 0.03 0.08 0.07 0.03 -0.00 -0.00 -0.01

22 6 0.00 -0.00 0.03 -0.01 -0.03 -0.02 -0.00 -0.00 0.00

23 6 0.01 -0.01 0.09 0.06 0.13 -0.01 0.00 0.00 0.00

24 6 -0.00 0.01 -0.00 -0.02 0.02 -0.04 0.00 0.00 0.00

25 1 -0.01 -0.06 -0.16 0.02 0.04 -0.29 0.00 0.03 0.06

26 6 0.01 -0.00 0.02 0.03 0.01 0.07 -0.00 -0.00 -0.01

27 6 -0.01 0.01 -0.12 -0.05 -0.04 0.05 -0.00 -0.00 -0.01

28 1 -0.03 0.07 -0.51 0.11 0.11 0.18 0.00 0.00 -0.02

29 6 -0.01 -0.00 -0.05 -0.06 0.01 -0.05 0.00 0.00 0.01

30 1 0.01 0.00 -0.00 -0.07 0.08 0.23 0.01 -0.00 -0.01

31 1 0.00 -0.02 -0.18 0.03 -0.05 -0.41 -0.00 0.01 0.07

32 1 0.05 -0.05 0.72 0.02 -0.22 -0.28 0.00 -0.01 0.04

33 1 0.01 0.02 0.33 -0.08 -0.01 0.34 -0.00 -0.01 -0.07

34 8 0.00 0.00 0.00 -0.05 -0.06 -0.02 -0.00 -0.00 -0.00

35 6 -0.00 -0.01 -0.00 -0.00 0.12 0.03 -0.00 0.00 0.00

36 1 -0.00 -0.00 -0.02 0.02 0.15 0.05 0.00 0.00 0.00

37 1 0.00 -0.01 -0.00 0.02 0.05 0.02 0.00 0.00 0.00

38 1 0.00 0.00 0.00 0.02 0.08 0.02 0.00 0.00 0.00

39 6 -0.00 0.00 0.01 -0.12 -0.01 0.00 -0.00 -0.00 -0.00

40 8 -0.00 0.00 0.01 -0.02 0.04 0.07 0.00 0.00 -0.00

41 8 0.01 0.00 -0.01 0.06 0.06 -0.10 -0.00 0.00 0.00

42 6 -0.00 0.00 0.00 -0.02 0.03 0.02 -0.01 0.00 0.01

43 6 0.01 -0.00 -0.00 0.03 -0.01 -0.02 -0.03 0.00 0.03

44 6 -0.00 -0.00 -0.00 -0.00 -0.00 -0.01 0.01 -0.00 -0.01

45 6 0.00 -0.00 0.00 0.03 -0.03 0.03 0.06 -0.01 -0.06

46 1 -0.03 0.00 0.03 -0.11 0.01 0.13 0.25 -0.04 -0.23

47 6 0.00 -0.00 -0.00 -0.01 -0.02 -0.03 0.03 -0.00 -0.03

48 6 -0.00 0.00 0.00 -0.02 0.01 0.01 -0.07 0.01 0.07

49 1 0.01 -0.01 -0.00 0.01 -0.06 0.03 -0.41 0.05 0.40

50 1 -0.02 -0.00 0.01 -0.09 -0.03 0.06 -0.20 0.02 0.20

51 1 0.01 -0.00 -0.02 0.08 -0.00 -0.12 0.47 -0.06 -0.45

52 8 0.00 -0.00 -0.00 -0.00 -0.00 -0.00 0.00 -0.00 -0.00

53 6 0.00 0.00 0.00 0.00 0.00 0.00 0.00 0.00 0.00

54 1 0.00 0.00 0.00 0.01 0.00 0.01 0.01 0.00 0.00

55 1 0.00 0.00 0.00 0.01 0.00 0.01 -0.00 -0.00 -0.00

56 1 0.00 0.00 0.00 0.00 0.00 0.00 -0.00 0.00 0.00

79 80 81

A A A

Frequencies -- 976.9212 987.7057 1009.5618

Red. masses -- 1.5479 1.3577 1.3566

Frc consts -- 0.8704 0.7804 0.8147

IR Inten -- 18.7697 0.4996 8.9394

Atom AN X Y Z X Y Z X Y Z

1 16 0.00 0.00 -0.00 0.00 -0.00 -0.00 -0.01 0.00 -0.00

2 6 0.04 -0.01 0.02 0.00 -0.00 0.00 -0.03 0.01 -0.01

3 6 0.00 0.04 0.01 -0.00 0.00 0.00 0.01 -0.00 -0.00

4 6 -0.02 0.03 0.00 0.00 0.00 -0.00 0.00 -0.00 -0.00

5 6 0.00 0.00 0.00 0.01 0.01 0.00 -0.01 -0.01 0.01

6 6 -0.00 -0.00 -0.00 0.05 0.01 0.05 -0.01 0.03 0.00

7 6 -0.00 -0.00 -0.00 -0.07 -0.02 -0.06 0.02 -0.01 -0.02

8 6 -0.00 0.00 0.00 -0.07 -0.02 -0.06 0.01 -0.00 -0.00

9 1 0.01 0.00 0.01 -0.31 -0.08 -0.25 0.00 0.03 0.02

10 6 -0.00 -0.00 -0.00 0.06 0.02 0.05 0.01 0.01 0.01

11 1 0.00 -0.00 0.00 0.40 0.10 0.37 0.05 -0.00 0.00

12 6 0.00 0.00 0.00 0.02 0.01 0.01 -0.02 -0.02 0.01

13 1 -0.01 -0.00 -0.00 0.39 0.10 0.35 -0.04 -0.02 -0.05

14 1 0.01 -0.00 0.01 -0.33 -0.10 -0.28 -0.06 -0.01 -0.05

15 1 -0.01 -0.00 -0.01 -0.09 -0.02 -0.09 0.05 -0.00 0.08

16 8 -0.00 0.00 0.01 -0.00 0.00 0.00 0.00 -0.00 -0.01

17 8 -0.01 -0.01 -0.01 -0.00 -0.00 -0.00 0.01 0.01 0.01

18 6 -0.04 0.03 -0.02 -0.00 0.00 -0.00 0.02 -0.02 0.01

19 1 -0.04 0.05 -0.02 -0.00 0.00 -0.00 0.01 -0.03 0.01

20 6 -0.02 0.00 -0.03 -0.00 0.00 -0.00 0.02 0.01 0.01

21 6 -0.01 -0.01 0.05 -0.00 -0.00 0.00 -0.03 -0.01 -0.09

22 6 0.00 0.01 -0.02 0.00 0.00 -0.00 -0.00 0.00 0.01

23 6 -0.02 -0.03 -0.04 -0.00 -0.00 -0.00 0.00 -0.00 -0.00

24 6 0.00 0.00 0.01 0.00 -0.00 0.00 0.01 -0.01 0.10

25 1 -0.03 -0.15 -0.27 -0.00 -0.00 -0.00 0.01 0.27 0.62

26 6 0.00 -0.00 0.09 0.00 0.00 0.00 -0.01 0.01 0.01

27 6 0.02 0.01 0.06 0.00 0.00 0.00 -0.01 -0.01 0.00

28 1 -0.02 -0.06 0.18 -0.00 -0.00 -0.00 0.02 -0.01 0.01

29 6 -0.01 -0.01 -0.10 0.00 -0.00 -0.00 0.03 -0.01 -0.05

30 1 0.03 -0.01 -0.07 0.00 -0.00 -0.00 -0.08 -0.12 -0.58

31 1 -0.02 -0.04 -0.55 0.00 -0.00 -0.00 -0.04 0.06 -0.14

32 1 -0.03 0.10 -0.31 -0.00 0.00 -0.00 -0.01 -0.02 -0.00

33 1 0.04 0.06 0.60 0.00 0.00 0.00 0.10 0.08 0.28

34 8 0.02 0.02 0.00 0.00 0.00 0.00 -0.00 -0.00 -0.00

35 6 0.00 -0.04 -0.01 -0.00 -0.00 -0.00 -0.00 0.00 0.00

36 1 -0.00 -0.05 -0.01 -0.00 -0.00 0.00 0.00 0.01 0.00

37 1 -0.01 -0.01 -0.01 -0.00 0.00 0.00 0.00 0.00 0.00

38 1 -0.01 -0.03 -0.01 0.00 -0.00 0.00 0.00 0.00 0.00

39 6 0.05 0.01 0.00 0.00 0.00 -0.00 -0.03 -0.01 -0.00

40 8 0.01 -0.01 -0.02 0.00 -0.00 -0.00 -0.00 0.01 0.01

41 8 -0.02 -0.02 0.03 -0.00 -0.00 0.00 0.01 0.01 -0.01

42 6 0.00 -0.01 -0.00 0.00 -0.00 -0.00 -0.00 0.01 0.00

43 6 -0.01 0.00 0.01 -0.00 0.00 0.00 0.00 -0.00 -0.00

44 6 0.00 0.00 0.00 0.00 0.00 0.00 -0.00 -0.00 -0.00

45 6 0.00 0.01 -0.02 -0.00 0.00 -0.00 0.00 -0.01 0.01

46 1 0.06 -0.00 -0.07 0.00 -0.00 -0.00 -0.03 -0.00 0.03

47 6 0.01 0.01 0.01 0.00 0.00 0.00 -0.00 -0.00 -0.00

48 6 -0.01 -0.00 0.01 0.00 -0.00 -0.00 0.00 0.00 -0.00

49 1 -0.07 0.03 0.06 -0.00 0.00 0.00 0.02 -0.01 -0.01

50 1 -0.01 0.02 0.02 0.00 0.00 -0.00 -0.00 -0.01 -0.00

51 1 0.06 -0.01 -0.05 0.00 -0.00 0.00 -0.01 0.00 0.00

52 8 0.00 0.00 0.00 0.00 0.00 0.00 -0.00 -0.00 -0.00

53 6 -0.00 -0.00 -0.00 -0.00 -0.00 -0.00 0.00 0.00 0.00

54 1 -0.00 -0.00 -0.00 -0.00 -0.00 -0.00 0.00 0.00 0.00

55 1 -0.00 -0.00 -0.00 -0.00 -0.00 -0.00 0.00 0.00 0.00

56 1 -0.00 0.00 -0.00 -0.00 -0.00 -0.00 0.00 0.00 0.00

82 83 84

A A A

Frequencies -- 1010.6205 1012.3601 1045.5339

Red. masses -- 1.7174 2.6356 3.5970

Frc consts -- 1.0335 1.5914 2.3167

IR Inten -- 0.7223 4.0899 38.9067

Atom AN X Y Z X Y Z X Y Z

1 16 -0.01 0.00 -0.00 -0.01 0.00 -0.00 -0.04 0.01 -0.01

2 6 0.01 -0.01 0.01 0.01 -0.01 0.01 -0.03 0.00 -0.01

3 6 -0.00 0.00 0.00 0.00 0.00 0.00 0.06 0.02 0.02

4 6 -0.00 0.00 0.00 -0.00 0.00 0.00 -0.01 0.03 0.00

5 6 -0.02 -0.02 0.02 -0.03 -0.04 0.03 -0.06 -0.09 0.09

6 6 -0.05 0.11 -0.01 -0.04 0.19 0.03 -0.04 -0.04 0.05

7 6 0.05 -0.05 -0.10 0.16 -0.06 -0.11 -0.02 -0.06 0.04

8 6 0.07 -0.00 0.02 -0.01 -0.03 -0.08 0.16 -0.13 -0.14

9 1 0.08 0.14 0.12 -0.22 0.15 -0.10 -0.20 -0.10 0.27

10 6 0.05 0.05 0.05 -0.07 0.03 -0.04 -0.11 0.22 0.04

11 1 0.29 0.01 0.11 -0.04 -0.11 -0.31 -0.06 -0.31 0.13

12 6 -0.11 -0.09 0.01 -0.02 -0.11 0.18 0.07 0.12 -0.11

13 1 -0.28 -0.11 -0.28 0.34 0.07 0.24 0.15 -0.44 -0.03

14 1 -0.38 -0.08 -0.31 0.28 0.13 0.29 -0.31 0.17 0.32

15 1 0.36 0.04 0.44 -0.46 -0.22 -0.20 0.06 0.14 -0.13

16 8 0.01 -0.01 -0.01 0.01 -0.01 -0.02 0.03 -0.03 -0.05

17 8 0.01 0.00 0.01 0.02 0.01 0.02 0.05 0.04 0.06

18 6 -0.01 0.00 -0.00 -0.00 -0.00 -0.00 -0.00 -0.03 -0.00

19 1 -0.01 0.00 -0.00 -0.00 -0.01 0.00 0.00 -0.10 -0.01

20 6 -0.01 -0.00 -0.00 -0.00 -0.00 -0.00 0.05 -0.02 -0.01

21 6 0.01 0.00 0.02 0.00 -0.00 0.01 -0.02 -0.02 0.01

22 6 0.00 0.00 -0.00 0.00 0.00 -0.00 0.02 0.00 -0.00

23 6 -0.00 -0.00 0.00 -0.00 -0.00 0.00 -0.01 -0.03 -0.00

24 6 -0.00 0.00 -0.02 -0.00 0.00 -0.01 0.01 0.05 -0.01

25 1 -0.00 -0.07 -0.16 -0.00 -0.03 -0.07 -0.01 -0.07 -0.05

26 6 0.00 -0.00 -0.00 -0.00 -0.00 -0.00 -0.06 -0.03 0.00

27 6 0.00 0.00 -0.00 0.00 0.00 0.00 0.00 0.00 0.00

28 1 -0.01 -0.00 -0.00 -0.00 -0.00 -0.00 0.03 -0.04 0.00

29 6 -0.01 0.00 0.01 -0.00 0.00 0.00 0.00 -0.00 0.00

30 1 0.02 0.03 0.14 0.01 0.01 0.06 0.07 0.05 0.01

31 1 0.01 -0.02 0.03 0.00 -0.01 0.01 -0.06 -0.04 0.01

32 1 -0.00 0.01 0.00 -0.00 0.01 -0.00 -0.03 0.08 -0.01

33 1 -0.02 -0.02 -0.07 -0.01 -0.01 -0.03 0.02 0.02 -0.02

34 8 0.00 0.00 0.00 0.00 0.00 0.00 0.01 0.04 0.01

35 6 -0.00 -0.01 -0.00 -0.00 -0.01 -0.00 -0.00 -0.05 -0.01

36 1 -0.00 -0.01 -0.00 -0.00 -0.01 -0.00 -0.00 -0.03 -0.01

37 1 -0.00 -0.00 -0.00 0.00 -0.00 -0.00 -0.01 -0.02 -0.01

38 1 -0.00 -0.00 -0.00 -0.00 -0.01 -0.00 -0.01 -0.04 -0.01

39 6 0.01 0.00 0.00 0.00 0.00 0.00 -0.01 -0.02 0.01

40 8 -0.00 -0.00 -0.00 -0.00 0.00 0.00 -0.00 0.01 0.01

41 8 -0.00 -0.00 0.00 -0.00 0.00 -0.00 0.01 0.02 -0.02

42 6 0.00 -0.00 -0.00 -0.00 0.00 0.00 -0.00 0.01 -0.00

43 6 -0.00 0.00 0.00 -0.00 -0.00 0.00 0.00 0.00 -0.00

44 6 0.00 0.00 0.00 0.00 -0.00 0.00 -0.00 0.00 -0.00

45 6 -0.00 0.00 -0.00 0.00 -0.00 -0.00 0.01 -0.01 0.01

46 1 0.01 0.00 -0.01 0.00 0.00 -0.00 -0.01 0.01 0.00

47 6 0.00 0.00 0.00 0.00 -0.00 -0.00 -0.01 -0.00 -0.01

48 6 -0.00 -0.00 0.00 -0.00 0.00 0.00 -0.00 -0.00 -0.00

49 1 -0.00 0.00 0.00 -0.00 0.00 0.00 0.01 -0.00 0.01

50 1 0.00 0.00 0.00 -0.00 0.00 0.00 -0.01 0.00 -0.01

51 1 0.00 -0.00 -0.00 0.00 -0.00 -0.00 -0.01 -0.01 -0.01

52 8 0.00 0.00 0.00 0.00 0.00 0.00 0.00 -0.00 0.00

53 6 -0.00 -0.00 -0.00 -0.00 -0.00 -0.00 0.00 0.00 0.00

54 1 -0.00 -0.00 -0.00 -0.00 0.00 -0.00 0.00 0.00 0.00

55 1 -0.00 -0.00 -0.00 -0.00 -0.00 -0.00 0.00 0.00 0.00

56 1 -0.00 -0.00 -0.00 -0.00 -0.00 -0.00 0.00 0.00 0.00

85 86 87

A A A

Frequencies -- 1050.7729 1058.0095 1069.5521

Red. masses -- 4.6254 5.1449 2.2986

Frc consts -- 3.0089 3.3932 1.5492

IR Inten -- 155.6555 47.8146 44.5113

Atom AN X Y Z X Y Z X Y Z

1 16 0.03 0.01 0.00 -0.01 -0.00 -0.00 -0.01 -0.00 -0.00

2 6 0.13 -0.05 0.04 -0.01 -0.00 -0.00 -0.00 0.01 -0.00

3 6 -0.17 -0.04 -0.05 0.01 0.00 0.00 0.03 0.01 0.01

4 6 0.03 -0.09 0.00 -0.00 0.01 0.00 0.01 -0.00 -0.00

5 6 -0.01 -0.02 0.02 -0.00 -0.00 0.00 -0.00 -0.01 0.01

6 6 -0.04 0.02 0.03 0.00 -0.00 -0.00 0.00 -0.01 -0.00

7 6 0.01 -0.05 0.01 -0.00 0.00 0.00 -0.00 0.01 0.00

8 6 0.07 -0.07 -0.05 -0.00 0.00 0.00 -0.00 0.00 -0.00

9 1 -0.11 -0.01 0.18 0.01 -0.00 -0.01 0.02 -0.01 -0.02

10 6 -0.06 0.10 0.03 0.00 -0.00 -0.00 0.00 -0.00 -0.00

11 1 -0.01 -0.25 0.08 -0.00 0.02 -0.01 0.00 0.04 -0.01

12 6 0.03 0.05 -0.05 -0.00 -0.00 0.00 -0.00 -0.00 0.00

13 1 0.05 -0.28 0.02 -0.00 0.02 -0.01 0.00 0.03 -0.01

14 1 -0.17 0.07 0.19 0.01 -0.00 -0.01 0.02 0.00 -0.02

15 1 0.04 0.04 -0.07 -0.00 0.00 0.00 -0.01 0.00 0.01

16 8 -0.02 0.02 0.03 0.00 -0.00 -0.01 0.01 -0.01 -0.01

17 8 -0.05 -0.04 -0.05 0.01 0.01 0.01 0.01 0.01 0.01

18 6 -0.02 0.09 0.00 0.01 -0.01 0.00 -0.01 -0.00 -0.01

19 1 -0.01 0.31 0.01 0.01 -0.04 -0.00 -0.01 -0.04 -0.00

20 6 -0.16 0.07 0.01 0.01 -0.01 -0.00 0.01 0.02 -0.01

21 6 0.05 0.06 -0.03 0.00 -0.00 0.00 -0.07 0.01 0.03

22 6 -0.06 -0.01 0.01 0.00 -0.00 -0.00 -0.02 0.01 -0.00

23 6 0.04 0.08 0.01 -0.00 -0.00 -0.00 -0.01 -0.02 -0.00

24 6 -0.06 -0.18 0.04 0.01 0.02 -0.00 -0.05 -0.08 0.00

25 1 0.00 0.19 0.05 0.01 -0.03 0.00 -0.19 0.14 -0.21

26 6 0.20 0.09 -0.02 -0.01 -0.01 0.00 0.02 0.04 -0.00

27 6 0.01 -0.01 -0.01 -0.00 0.00 0.00 0.00 -0.00 0.00

28 1 -0.11 0.10 0.00 0.01 -0.00 -0.00 -0.02 -0.02 -0.00

29 6 0.01 -0.00 -0.02 -0.01 0.00 0.00 0.11 -0.02 -0.01

30 1 -0.24 -0.17 0.02 0.01 0.02 -0.01 -0.16 -0.06 0.11

31 1 0.17 0.17 -0.01 -0.01 -0.02 -0.00 -0.08 0.26 0.01

32 1 0.10 -0.22 0.03 -0.01 0.01 -0.00 -0.00 0.01 -0.01

33 1 -0.00 -0.04 0.05 -0.01 0.00 -0.00 0.20 0.10 -0.03

34 8 -0.02 -0.12 -0.03 0.00 0.01 0.00 -0.00 -0.02 -0.00

35 6 0.01 0.14 0.03 -0.00 -0.01 -0.00 0.00 0.02 0.00

36 1 0.00 0.10 0.03 -0.00 -0.01 -0.00 0.00 0.02 0.01

37 1 0.02 0.05 0.03 -0.00 -0.00 -0.00 0.00 0.01 0.00

38 1 0.02 0.10 0.02 -0.00 -0.01 -0.00 0.00 0.02 0.00

39 6 0.05 0.07 -0.04 -0.00 0.01 -0.00 0.01 -0.01 0.01

40 8 0.01 -0.03 -0.02 -0.01 0.00 0.01 -0.00 0.00 -0.00

41 8 -0.03 -0.05 0.06 -0.00 -0.01 -0.02 0.00 -0.01 -0.00

42 6 0.00 -0.03 0.00 0.03 0.04 0.04 -0.00 0.02 -0.00

43 6 -0.01 -0.02 -0.00 -0.14 -0.17 -0.13 -0.02 0.07 -0.02

44 6 0.00 -0.01 0.01 0.11 -0.06 0.12 0.05 0.00 0.05

45 6 -0.03 0.03 -0.04 -0.05 0.04 -0.05 0.10 -0.04 0.11

46 1 0.04 -0.07 -0.01 -0.06 -0.49 0.05 -0.13 0.45 -0.24

47 6 0.02 0.01 0.03 0.00 -0.08 0.01 -0.08 0.01 -0.08

48 6 0.01 0.03 0.00 0.04 0.23 0.02 -0.03 -0.09 -0.02

49 1 -0.05 0.01 -0.04 -0.04 0.07 -0.06 0.17 0.12 0.18

50 1 0.04 -0.03 0.04 0.08 -0.28 0.12 -0.20 0.32 -0.24

51 1 0.04 0.03 0.04 0.23 0.24 0.19 -0.13 -0.10 -0.12

52 8 0.00 0.01 0.00 0.17 0.15 0.16 0.06 0.04 0.06

53 6 -0.01 -0.00 -0.01 -0.20 -0.13 -0.20 -0.06 -0.06 -0.06

54 1 -0.01 -0.01 -0.00 -0.11 -0.11 -0.13 -0.06 -0.03 -0.08

55 1 -0.00 -0.01 -0.01 -0.13 -0.11 -0.11 -0.08 -0.03 -0.06

56 1 -0.01 -0.01 -0.01 -0.16 -0.09 -0.16 -0.01 -0.00 -0.01

88 89 90

A A A

Frequencies -- 1070.5332 1078.3171 1107.4184

Red. masses -- 2.3617 3.5512 1.6462

Frc consts -- 1.5947 2.4329 1.1894

IR Inten -- 20.6516 131.9063 10.4643

Atom AN X Y Z X Y Z X Y Z

1 16 -0.04 -0.00 -0.00 0.10 -0.00 0.00 0.02 0.00 0.01

2 6 -0.01 -0.00 -0.01 -0.09 0.04 -0.03 -0.01 0.01 -0.00

3 6 0.04 0.01 0.02 0.07 0.02 0.03 0.01 0.02 0.01

4 6 0.01 0.00 -0.00 0.01 0.02 -0.00 0.02 0.01 0.00

5 6 -0.02 -0.03 0.03 0.08 0.10 -0.12 -0.02 0.05 0.00

6 6 0.02 -0.03 -0.01 -0.05 0.09 0.02 -0.03 -0.09 0.05

7 6 -0.01 0.03 0.00 0.03 -0.07 -0.01 0.07 0.03 -0.09

8 6 0.00 0.02 -0.01 -0.03 -0.03 0.05 0.06 -0.01 -0.06

9 1 0.06 -0.02 -0.07 -0.17 0.07 0.18 -0.26 -0.17 0.38

10 6 0.01 -0.01 -0.01 -0.02 -0.00 0.02 0.00 -0.08 0.03

11 1 0.00 0.17 -0.04 -0.02 -0.50 0.13 0.11 0.41 -0.23

12 6 -0.01 -0.01 0.01 0.03 0.01 -0.03 -0.04 0.07 0.03

13 1 0.01 0.13 -0.05 -0.06 -0.34 0.17 0.07 0.17 -0.14

14 1 0.06 0.01 -0.08 -0.13 -0.03 0.18 -0.18 -0.15 0.25

15 1 -0.03 0.02 0.03 0.09 -0.08 -0.09 -0.29 0.40 0.20

16 8 0.03 -0.03 -0.05 -0.09 0.08 0.13 -0.01 0.01 0.01

17 8 0.05 0.04 0.05 -0.11 -0.09 -0.11 -0.03 -0.02 -0.02

18 6 -0.00 -0.02 0.00 0.02 -0.05 -0.01 -0.00 -0.01 -0.01

19 1 0.01 -0.08 -0.03 -0.02 -0.11 0.05 -0.02 -0.01 0.02

20 6 0.03 0.02 -0.01 0.07 -0.01 -0.00 -0.01 0.01 -0.00

21 6 -0.10 0.01 0.04 -0.08 -0.01 0.03 -0.00 -0.01 0.00

22 6 -0.02 0.02 -0.00 0.01 0.01 -0.00 0.01 0.00 -0.00

23 6 -0.01 -0.03 -0.00 -0.01 -0.04 -0.01 -0.01 -0.02 -0.00

24 6 -0.08 -0.11 0.00 -0.03 -0.02 -0.01 0.00 0.00 -0.00

25 1 -0.27 0.19 -0.30 -0.18 0.10 -0.19 0.01 -0.03 -0.00

26 6 0.02 0.06 -0.01 -0.04 0.01 0.00 -0.00 -0.01 0.00

27 6 0.00 -0.00 0.00 -0.01 -0.00 0.01 -0.01 -0.00 0.00

28 1 -0.01 -0.03 -0.00 0.03 -0.05 -0.00 -0.00 -0.02 -0.00

29 6 0.15 -0.03 -0.02 0.09 -0.02 -0.01 -0.00 0.00 0.00

30 1 -0.24 -0.07 0.16 -0.08 -0.01 0.08 0.03 -0.00 -0.00

31 1 -0.12 0.36 0.01 -0.12 0.18 0.01 0.01 -0.03 0.00

32 1 -0.00 0.01 -0.02 -0.05 0.08 -0.01 -0.04 0.06 0.01

33 1 0.30 0.15 -0.05 0.20 0.12 -0.04 0.01 0.02 -0.00

34 8 -0.00 -0.01 -0.00 -0.00 0.00 0.00 -0.00 -0.03 -0.01

35 6 -0.00 0.01 0.00 -0.00 -0.00 -0.00 0.00 0.03 0.01

36 1 0.00 0.01 0.00 -0.00 -0.00 -0.00 -0.00 0.01 0.01

37 1 0.00 0.01 0.00 0.00 -0.00 -0.00 0.01 0.01 0.01

38 1 0.00 0.00 0.00 0.00 -0.00 -0.00 0.01 0.02 0.01

39 6 0.00 0.00 0.01 -0.03 -0.07 0.04 -0.00 -0.02 0.01

40 8 -0.00 0.00 0.01 -0.00 0.02 0.01 -0.00 0.00 0.00

41 8 0.00 0.02 -0.01 0.02 0.05 -0.04 0.00 0.01 -0.01

42 6 0.00 -0.01 0.00 0.00 0.00 -0.00 0.00 0.00 -0.00

43 6 0.01 -0.05 0.01 0.00 -0.02 0.00 0.00 -0.00 -0.00

44 6 -0.03 -0.00 -0.03 -0.01 0.00 -0.01 -0.00 0.00 -0.00

45 6 -0.06 0.02 -0.06 -0.01 0.00 -0.01 -0.00 -0.00 0.00

46 1 0.09 -0.30 0.15 0.03 -0.11 0.06 0.00 -0.01 0.01

47 6 0.05 -0.01 0.05 0.01 -0.01 0.01 -0.00 -0.00 -0.00

48 6 0.02 0.06 0.01 0.01 0.02 0.00 0.00 0.00 0.00

49 1 -0.11 -0.08 -0.11 -0.03 -0.03 -0.03 0.00 -0.00 0.00

50 1 0.12 -0.21 0.15 0.04 -0.09 0.05 0.00 -0.01 0.00

51 1 0.09 0.07 0.08 0.02 0.03 0.02 0.00 0.00 0.00

52 8 -0.04 -0.03 -0.04 -0.01 -0.01 -0.01 0.00 -0.00 0.00

53 6 0.04 0.04 0.04 0.01 0.01 0.01 0.00 0.00 0.00

54 1 0.04 0.02 0.05 0.01 0.00 0.02 0.00 0.00 0.00

55 1 0.05 0.02 0.04 0.02 0.01 0.02 0.00 0.00 0.00

56 1 0.01 0.00 0.01 0.00 0.00 0.00 0.00 0.00 0.00

91 92 93

A A A

Frequencies -- 1111.1163 1117.9418 1131.6500

Red. masses -- 4.1347 4.4425 2.0303

Frc consts -- 3.0075 3.2713 1.5319

IR Inten -- 32.6228 56.7177 150.6001

Atom AN X Y Z X Y Z X Y Z

1 16 0.01 -0.01 0.01 -0.13 -0.05 0.02 0.00 0.00 0.00

2 6 0.01 0.01 0.01 0.04 -0.02 0.00 0.00 0.01 0.00

3 6 -0.08 -0.17 -0.06 -0.00 0.02 0.00 -0.00 -0.01 -0.00

4 6 -0.13 -0.05 -0.01 0.01 0.00 0.00 -0.01 -0.00 -0.00

5 6 0.01 0.04 -0.03 0.16 0.24 -0.22 -0.00 -0.00 0.00

6 6 -0.01 -0.02 0.02 -0.04 0.04 0.03 -0.00 -0.00 0.00

7 6 0.03 0.00 -0.03 0.04 -0.05 -0.03 0.00 0.00 -0.00

8 6 0.01 -0.01 -0.01 -0.07 -0.01 0.08 0.00 -0.00 -0.00

9 1 -0.12 -0.05 0.17 -0.31 -0.03 0.41 0.00 -0.00 -0.00

10 6 -0.00 -0.04 0.01 -0.00 -0.11 0.03 -0.00 0.00 0.00

11 1 0.04 0.08 -0.07 0.01 -0.49 0.13 0.00 0.01 -0.01

12 6 -0.01 0.03 0.00 0.02 0.04 -0.03 -0.00 0.00 0.00

13 1 0.01 -0.00 -0.02 -0.09 -0.23 0.17 0.00 -0.00 -0.00

14 1 -0.10 -0.07 0.13 -0.15 -0.17 0.25 -0.00 -0.00 0.00

15 1 -0.10 0.14 0.06 -0.00 0.06 -0.02 -0.00 0.00 0.00

16 8 -0.01 0.01 0.00 0.07 -0.06 -0.10 -0.00 0.00 0.00

17 8 -0.02 -0.01 -0.02 0.12 0.10 0.12 -0.01 -0.00 -0.01

18 6 0.04 0.05 0.03 -0.01 0.01 0.01 0.00 0.01 -0.01

19 1 0.06 0.10 -0.01 0.03 -0.05 -0.07 -0.01 0.01 0.01

20 6 0.10 -0.10 0.03 -0.03 0.02 -0.00 0.00 -0.01 0.00

21 6 0.01 0.04 -0.01 0.01 -0.00 -0.00 0.01 0.00 -0.00

22 6 -0.03 -0.04 0.00 -0.00 0.00 0.00 -0.00 -0.00 0.00

23 6 0.06 0.12 0.02 -0.01 -0.01 -0.00 0.00 0.00 0.00

24 6 -0.04 0.01 -0.00 0.00 -0.01 0.00 -0.00 0.00 -0.00

25 1 -0.11 0.30 -0.04 0.03 -0.05 0.03 0.00 -0.00 -0.01

26 6 0.01 0.04 -0.01 0.01 -0.00 -0.00 0.00 0.01 -0.00

27 6 0.05 0.01 -0.01 0.00 0.00 -0.00 0.00 0.00 -0.00

28 1 0.04 0.14 0.02 -0.02 -0.01 -0.00 -0.01 0.01 0.00

29 6 0.04 -0.03 -0.00 -0.01 0.00 0.00 0.00 -0.00 -0.00

30 1 -0.21 0.03 0.05 0.01 -0.01 -0.01 -0.04 0.01 0.00

31 1 -0.10 0.29 -0.01 0.03 -0.04 0.00 -0.01 0.03 -0.00

32 1 0.25 -0.45 -0.05 -0.01 0.03 0.01 0.01 -0.02 -0.00

33 1 -0.02 -0.11 0.01 -0.02 -0.01 0.00 -0.01 -0.02 0.00

34 8 0.00 0.25 0.06 0.00 -0.03 -0.01 0.00 0.01 0.00

35 6 -0.03 -0.22 -0.04 0.01 0.02 0.01 -0.00 -0.01 -0.00

36 1 0.01 -0.08 -0.05 -0.00 0.00 -0.00 0.00 -0.00 -0.00

37 1 -0.02 -0.06 -0.05 -0.00 0.03 0.01 -0.00 -0.01 -0.00

38 1 -0.04 -0.18 -0.04 -0.01 0.01 -0.00 -0.00 -0.01 -0.00

39 6 0.01 0.06 -0.05 0.01 0.05 -0.03 0.00 -0.00 -0.00

40 8 0.01 -0.02 -0.00 0.00 -0.01 0.00 -0.00 0.00 0.00

41 8 -0.01 -0.03 0.04 -0.01 -0.03 0.02 -0.01 -0.07 -0.00

42 6 -0.01 -0.03 -0.00 0.00 0.01 0.00 0.05 0.12 0.04

43 6 0.00 0.01 0.01 -0.00 -0.00 -0.00 -0.04 -0.09 -0.03

44 6 0.00 -0.01 0.00 0.00 -0.00 0.01 0.03 0.06 0.02

45 6 -0.01 0.01 -0.01 0.00 0.00 0.00 0.05 0.04 0.05

46 1 -0.01 0.09 -0.05 -0.00 0.00 -0.01 0.10 -0.51 0.18

47 6 0.01 0.01 0.01 -0.00 0.00 -0.00 -0.11 -0.04 -0.11

48 6 -0.00 -0.01 -0.00 0.00 -0.00 0.00 0.03 -0.01 0.03

49 1 -0.02 -0.02 -0.01 0.01 0.01 0.01 0.27 0.51 0.21

50 1 0.01 0.04 -0.00 -0.01 0.02 -0.01 -0.18 0.14 -0.20

51 1 -0.02 -0.01 -0.02 0.01 -0.00 0.01 0.26 -0.02 0.26

52 8 0.01 0.01 0.01 -0.01 -0.00 -0.01 -0.06 -0.03 -0.06

53 6 -0.01 -0.01 -0.01 0.00 0.00 0.00 0.05 0.03 0.05

54 1 -0.01 -0.00 -0.01 -0.00 0.00 -0.00 -0.00 0.03 -0.00

55 1 -0.01 -0.00 -0.01 -0.00 0.00 -0.00 -0.00 0.03 -0.01

56 1 -0.00 -0.00 -0.00 -0.00 -0.00 -0.00 0.04 0.02 0.04

94 95 96

A A A

Frequencies -- 1161.9789 1174.7771 1178.7964

Red. masses -- 1.8768 1.2703 1.2690

Frc consts -- 1.4930 1.0330 1.0390

IR Inten -- 355.5749 13.3127 1.7895

Atom AN X Y Z X Y Z X Y Z

1 16 0.01 0.00 0.00 0.00 -0.00 -0.00 -0.00 -0.00 0.00

2 6 -0.00 -0.03 -0.00 0.00 0.00 0.00 0.00 0.00 0.00

3 6 -0.10 -0.03 -0.02 0.01 -0.00 0.00 0.00 -0.00 -0.00

4 6 -0.03 -0.01 0.00 0.00 -0.00 -0.00 -0.00 -0.00 -0.00

5 6 -0.00 -0.00 0.00 0.00 -0.00 0.00 0.00 0.00 -0.00

6 6 -0.00 0.00 0.00 -0.00 -0.00 0.00 -0.00 -0.00 0.00

7 6 0.00 0.00 -0.00 -0.00 0.00 0.00 -0.00 -0.00 0.00

8 6 0.00 -0.00 -0.00 0.00 0.00 -0.00 -0.00 0.00 0.00

9 1 0.01 0.00 -0.01 0.00 -0.00 -0.00 -0.00 -0.00 0.00

10 6 -0.00 0.00 0.00 -0.00 0.00 0.00 0.00 -0.00 -0.00

11 1 0.00 0.03 -0.01 0.00 0.00 0.00 -0.00 -0.00 0.00

12 6 -0.00 -0.00 0.00 0.00 -0.00 -0.00 0.00 0.00 -0.00

13 1 0.00 -0.01 0.00 0.00 0.01 -0.00 -0.00 0.00 -0.00

14 1 -0.01 -0.00 0.02 -0.00 0.00 -0.00 0.00 0.00 -0.00

15 1 -0.00 0.00 0.00 0.01 -0.01 -0.00 -0.00 0.00 -0.00

16 8 -0.00 0.00 0.00 -0.00 0.00 0.00 0.00 -0.00 -0.00

17 8 -0.01 -0.00 -0.01 0.00 -0.00 0.00 0.00 0.00 0.00

18 6 0.03 -0.00 0.02 -0.00 0.00 -0.00 -0.00 0.00 -0.00

19 1 0.04 0.03 -0.00 -0.00 0.00 -0.00 -0.00 0.00 0.00

20 6 0.04 0.04 -0.00 -0.01 0.01 -0.00 -0.00 0.00 -0.00

21 6 -0.10 0.00 0.01 -0.00 -0.00 0.00 -0.00 0.00 0.00

22 6 0.09 -0.00 -0.00 -0.00 0.00 -0.00 -0.00 -0.00 -0.00

23 6 0.03 0.05 0.01 -0.01 0.01 0.00 -0.00 0.00 0.00

24 6 0.03 0.00 -0.00 -0.00 -0.00 0.00 -0.00 -0.00 0.00

25 1 -0.18 0.12 -0.08 0.01 -0.02 0.00 -0.00 0.00 -0.00

26 6 -0.07 -0.07 0.01 0.00 -0.01 0.00 0.00 -0.00 0.00

27 6 -0.04 -0.03 -0.00 0.00 -0.01 -0.00 0.00 -0.00 -0.00

28 1 0.30 0.02 -0.01 -0.07 0.01 -0.01 -0.01 0.00 0.00

29 6 0.03 0.04 -0.00 0.00 0.01 -0.00 0.00 0.00 -0.00

30 1 0.29 -0.03 -0.01 -0.03 0.00 -0.00 0.00 -0.00 -0.00

31 1 0.09 -0.43 0.02 0.04 -0.09 0.01 0.00 -0.01 0.00

32 1 -0.07 0.04 0.01 0.03 -0.07 -0.00 0.00 -0.01 -0.00

33 1 0.40 0.56 -0.09 0.06 0.09 -0.01 0.00 0.01 -0.00

34 8 0.01 -0.03 -0.00 -0.01 0.01 -0.06 -0.00 0.00 0.00

35 6 0.01 0.02 -0.00 0.02 -0.03 0.13 0.00 -0.00 -0.00

36 1 -0.02 -0.04 0.00 -0.03 0.06 -0.27 0.00 -0.00 0.00

37 1 -0.01 -0.02 0.01 0.08 0.66 0.01 -0.00 -0.00 0.00

38 1 0.00 0.04 0.01 -0.13 -0.55 -0.32 -0.00 0.00 0.00

39 6 0.02 0.10 -0.07 -0.00 -0.01 0.01 -0.00 -0.00 0.00

40 8 -0.00 -0.01 0.01 0.00 0.00 -0.00 0.00 0.00 -0.00

41 8 -0.01 -0.05 0.04 0.00 0.01 -0.00 0.00 0.00 -0.00

42 6 -0.00 0.01 0.00 -0.00 -0.01 -0.00 -0.00 -0.00 0.00

43 6 -0.00 -0.00 -0.00 0.00 -0.00 0.00 -0.00 -0.00 0.00

44 6 0.01 -0.00 0.01 -0.00 0.00 -0.00 -0.00 0.00 -0.00

45 6 -0.00 0.00 -0.00 0.00 0.00 0.00 -0.00 0.00 -0.00

46 1 -0.02 0.04 -0.02 0.01 -0.02 0.01 0.00 0.00 -0.00

47 6 -0.00 0.00 -0.00 0.00 0.00 0.00 -0.00 0.00 0.00

48 6 0.00 0.00 0.00 -0.00 -0.00 -0.00 0.00 -0.00 0.00

49 1 -0.02 -0.04 -0.02 0.01 0.03 0.01 -0.00 -0.00 -0.00

50 1 -0.01 0.03 -0.02 0.00 -0.00 0.00 0.01 0.00 -0.01

51 1 0.03 0.00 0.03 -0.01 -0.00 -0.01 0.01 -0.00 0.01

52 8 -0.01 0.00 -0.01 0.00 0.00 0.00 0.04 -0.00 -0.04

53 6 0.00 0.00 0.00 -0.00 -0.00 -0.00 -0.10 0.01 0.09

54 1 -0.01 -0.00 0.00 -0.00 0.00 -0.00 0.52 0.33 0.26

55 1 0.00 -0.00 -0.00 -0.00 0.00 -0.00 -0.27 -0.35 -0.51

56 1 -0.01 -0.01 -0.01 0.01 0.01 0.01 0.21 -0.01 -0.19

97 98 99

A A A

Frequencies -- 1179.1444 1182.9989 1185.6084

Red. masses -- 1.3568 1.2401 1.3158

Frc consts -- 1.1115 1.0225 1.0897

IR Inten -- 370.6657 11.5978 117.2345

Atom AN X Y Z X Y Z X Y Z

1 16 0.01 0.00 -0.00 -0.00 -0.00 -0.00 -0.00 -0.00 -0.00

2 6 -0.01 -0.01 -0.00 0.01 -0.01 0.00 0.01 0.01 0.01

3 6 -0.03 0.02 0.00 -0.04 0.02 -0.00 0.00 0.05 0.01

4 6 -0.02 0.01 0.00 -0.03 -0.01 -0.00 -0.03 -0.02 -0.00

5 6 -0.00 -0.00 0.00 0.00 0.00 0.00 0.00 0.00 0.00

6 6 0.00 0.00 -0.00 -0.00 -0.00 0.00 -0.00 -0.00 0.00

7 6 0.00 0.00 -0.00 -0.00 -0.00 0.00 -0.00 -0.00 0.00

8 6 0.00 -0.00 -0.00 -0.00 0.00 -0.00 -0.00 -0.00 0.00

9 1 0.01 0.00 -0.01 -0.00 -0.00 0.00 -0.00 -0.00 0.01

10 6 -0.00 0.00 0.00 0.00 0.00 -0.00 0.00 0.00 -0.01

11 1 0.00 0.02 -0.01 -0.00 -0.01 0.01 -0.00 -0.04 0.02

12 6 0.00 -0.00 0.00 -0.00 0.00 0.00 -0.00 0.00 0.00

13 1 0.00 -0.01 0.00 0.00 0.00 -0.00 -0.00 -0.01 0.00

14 1 -0.01 -0.00 0.01 0.01 0.00 -0.02 0.05 0.01 -0.05

15 1 0.00 -0.00 -0.00 -0.01 0.01 0.00 -0.03 0.04 0.02

16 8 -0.00 0.00 0.01 0.00 -0.00 -0.00 0.00 -0.00 -0.00

17 8 -0.00 -0.00 -0.00 0.00 0.00 0.00 0.01 0.00 0.01

18 6 0.01 -0.02 0.01 -0.00 0.01 0.00 -0.02 0.01 -0.01

19 1 0.02 -0.03 -0.00 -0.00 0.03 0.00 -0.02 0.02 0.01

20 6 0.04 -0.01 0.00 0.03 0.01 0.00 0.02 0.03 -0.00

21 6 -0.01 0.01 -0.00 -0.03 0.01 0.00 -0.03 0.01 0.00

22 6 0.01 -0.02 0.00 0.01 -0.03 0.00 -0.02 -0.03 -0.00

23 6 0.04 -0.02 -0.01 0.02 0.01 -0.00 -0.02 0.04 0.01

24 6 0.03 -0.00 -0.00 0.03 -0.01 -0.00 0.03 -0.01 -0.00

25 1 -0.07 0.13 -0.03 -0.13 0.19 -0.05 -0.14 0.21 -0.07

26 6 -0.02 0.04 -0.00 -0.02 0.00 0.00 -0.00 -0.02 0.00

27 6 -0.02 0.02 0.01 -0.01 0.01 0.00 0.04 -0.01 -0.01

28 1 0.32 -0.05 -0.02 0.09 0.00 0.01 -0.38 0.07 0.05

29 6 -0.01 -0.04 0.00 -0.01 -0.02 0.00 -0.01 0.01 0.00

30 1 0.27 -0.03 -0.00 0.37 -0.05 -0.01 0.43 -0.06 -0.02

31 1 -0.19 0.39 -0.02 -0.10 0.17 -0.01 -0.00 -0.03 0.00

32 1 -0.14 0.30 0.03 -0.02 0.05 0.00 0.19 -0.36 -0.05

33 1 -0.26 -0.38 0.06 -0.13 -0.18 0.03 -0.05 -0.04 0.01

34 8 0.00 -0.03 -0.02 -0.00 -0.02 -0.01 -0.02 -0.02 0.00

35 6 0.01 0.02 0.03 0.03 0.01 0.00 0.06 0.01 -0.01

36 1 -0.02 -0.03 -0.06 -0.03 -0.13 -0.04 -0.04 -0.28 -0.05

37 1 0.01 0.14 0.01 -0.04 0.07 0.04 -0.10 0.09 0.08

38 1 -0.03 -0.09 -0.07 -0.05 0.06 -0.00 -0.10 0.19 0.03

39 6 0.01 0.05 -0.04 0.00 -0.00 0.01 -0.00 -0.05 0.04

40 8 -0.00 -0.00 0.01 0.00 -0.00 -0.00 0.00 0.01 -0.01

41 8 -0.01 -0.05 0.02 0.00 0.03 -0.00 0.01 0.03 -0.02

42 6 0.01 0.06 0.00 -0.01 -0.05 -0.00 0.00 -0.02 0.00

43 6 -0.01 0.01 -0.01 0.02 -0.01 0.02 -0.01 -0.01 -0.01

44 6 0.02 -0.00 0.02 -0.02 -0.01 -0.02 -0.00 0.01 -0.00

45 6 -0.01 -0.03 -0.00 0.01 0.05 0.01 -0.01 -0.01 -0.01

46 1 -0.06 0.15 -0.08 0.07 -0.21 0.13 -0.05 0.07 -0.04

47 6 -0.01 -0.01 -0.00 0.01 0.00 0.01 -0.01 0.01 -0.01

48 6 0.01 0.01 0.01 -0.03 -0.01 -0.03 0.02 -0.00 0.02

49 1 -0.13 -0.30 -0.09 0.21 0.46 0.15 -0.08 -0.17 -0.07

50 1 -0.02 0.04 -0.03 0.07 -0.16 0.09 -0.06 0.15 -0.08

51 1 0.17 0.01 0.17 -0.36 -0.01 -0.37 0.25 -0.00 0.26

52 8 -0.01 -0.00 -0.02 0.02 0.00 0.02 -0.00 0.00 -0.00

53 6 0.00 0.02 0.01 -0.01 -0.01 -0.01 0.01 -0.01 0.00

54 1 0.02 -0.00 0.03 -0.00 0.00 -0.01 -0.02 0.01 -0.03

55 1 0.02 -0.01 0.01 -0.02 -0.00 -0.01 -0.02 0.02 -0.01

56 1 -0.04 -0.04 -0.04 0.02 0.02 0.02 0.04 0.03 0.04

100 101 102

A A A

Frequencies -- 1186.0161 1190.3256 1202.3400

Red. masses -- 1.1044 1.9213 1.1651

Frc consts -- 0.9153 1.6039 0.9924

IR Inten -- 0.3462 139.9631 8.5648

Atom AN X Y Z X Y Z X Y Z

1 16 0.00 0.00 -0.00 0.00 -0.00 -0.00 0.00 0.00 -0.00

2 6 -0.00 -0.00 0.00 -0.01 0.01 -0.00 0.01 0.00 0.00

3 6 -0.00 -0.01 -0.00 0.03 0.03 0.01 -0.01 -0.01 -0.00

4 6 0.00 0.00 0.00 -0.01 0.00 -0.00 0.01 -0.01 -0.00

5 6 -0.00 -0.00 0.00 -0.00 -0.00 -0.00 -0.01 -0.01 0.02

6 6 0.00 -0.01 0.00 0.00 0.00 -0.00 -0.04 -0.02 0.04

7 6 0.00 -0.01 0.00 0.00 0.00 -0.00 -0.00 -0.04 0.02

8 6 0.00 -0.03 0.01 0.00 -0.00 0.00 0.01 0.06 -0.03

9 1 0.05 0.01 -0.07 0.02 0.01 -0.02 -0.30 -0.10 0.38

10 6 0.03 0.01 -0.04 -0.00 0.00 -0.00 0.03 0.01 -0.03

11 1 -0.01 -0.23 0.08 0.00 0.01 -0.01 -0.03 -0.40 0.15

12 6 -0.04 0.05 0.02 -0.00 0.00 0.00 0.01 -0.01 -0.01

13 1 -0.02 -0.35 0.14 -0.00 -0.04 0.02 0.05 0.56 -0.23

14 1 0.35 0.10 -0.42 0.00 0.00 -0.00 0.24 0.07 -0.30

15 1 -0.37 0.50 0.26 -0.02 0.03 0.02 0.06 -0.07 -0.04

16 8 -0.00 0.00 0.00 -0.00 0.00 0.01 -0.00 0.00 0.00

17 8 -0.00 -0.00 0.00 -0.00 -0.00 -0.00 -0.00 -0.00 -0.00

18 6 0.00 -0.00 0.00 -0.00 -0.01 -0.01 -0.00 0.01 -0.00

19 1 0.00 0.00 0.00 -0.01 -0.05 -0.00 -0.01 0.02 0.01

20 6 -0.00 -0.00 0.00 0.01 0.01 -0.00 -0.00 -0.00 0.00

21 6 0.00 -0.00 -0.00 -0.00 -0.00 0.00 -0.01 0.00 0.00

22 6 0.00 0.00 0.00 -0.02 -0.01 -0.00 0.00 -0.00 0.00

23 6 0.00 -0.00 -0.00 -0.03 0.01 0.00 0.00 0.00 -0.00

24 6 -0.00 0.00 0.00 -0.00 -0.00 0.00 0.00 -0.00 -0.00

25 1 0.01 -0.02 0.01 -0.01 0.00 -0.01 -0.02 0.04 -0.01

26 6 -0.00 0.00 -0.00 0.01 -0.01 0.00 -0.00 -0.00 0.00

27 6 -0.00 0.00 0.00 0.03 -0.01 -0.00 -0.00 0.00 0.00

28 1 0.04 -0.01 -0.01 -0.30 0.04 0.03 0.02 -0.00 -0.00

29 6 0.00 -0.00 -0.00 0.00 0.01 -0.00 0.00 -0.00 0.00

30 1 -0.04 0.00 0.00 0.03 -0.01 -0.00 0.05 -0.01 -0.00

31 1 -0.00 0.00 -0.00 0.03 -0.05 0.01 0.00 -0.01 -0.00

32 1 -0.02 0.04 0.01 0.13 -0.25 -0.03 -0.01 0.02 0.00

33 1 0.01 0.01 -0.00 0.02 0.03 -0.01 0.01 0.01 -0.00

34 8 0.00 0.00 -0.00 -0.01 -0.00 0.00 0.01 0.01 0.00

35 6 -0.01 -0.00 0.00 0.03 -0.00 -0.01 -0.02 -0.00 0.00

36 1 0.01 0.04 0.00 -0.02 -0.14 -0.03 0.02 0.09 0.02

37 1 0.01 -0.01 -0.01 -0.05 0.05 0.04 0.03 -0.04 -0.03

38 1 0.01 -0.03 -0.01 -0.05 0.09 0.01 0.03 -0.05 -0.00

39 6 -0.00 0.00 -0.00 0.00 0.00 -0.01 0.00 -0.02 0.01

40 8 0.00 -0.00 0.00 -0.00 0.00 0.00 0.00 0.00 -0.00

41 8 -0.00 -0.00 0.00 -0.01 -0.09 0.02 0.00 -0.00 -0.00

42 6 -0.00 -0.00 -0.00 0.03 0.19 0.00 0.00 0.02 0.00

43 6 0.00 -0.00 0.00 -0.01 0.06 -0.02 -0.00 0.00 -0.00

44 6 -0.00 -0.00 -0.00 0.04 -0.03 0.04 0.00 -0.00 0.00

45 6 0.00 0.00 -0.00 0.02 -0.05 0.03 0.00 -0.01 0.00

46 1 0.00 -0.00 -0.00 0.01 0.04 -0.01 -0.00 0.01 -0.00

47 6 0.00 0.00 0.00 0.00 -0.06 0.01 -0.00 -0.00 -0.00

48 6 -0.00 -0.00 -0.00 -0.03 0.03 -0.03 -0.00 0.00 -0.00

49 1 0.00 0.00 0.00 -0.02 -0.15 0.00 -0.01 -0.02 -0.00

50 1 -0.00 0.00 -0.00 0.12 -0.39 0.17 0.00 -0.02 0.01

51 1 -0.00 -0.00 -0.00 -0.35 0.03 -0.36 -0.01 0.00 -0.01

52 8 0.00 0.00 0.00 -0.02 -0.03 -0.02 -0.00 -0.00 -0.00

53 6 -0.00 -0.00 0.00 -0.01 0.08 -0.01 -0.00 0.01 -0.00

54 1 0.00 0.00 -0.00 0.11 -0.04 0.18 0.01 -0.00 0.02

55 1 -0.00 -0.00 -0.00 0.17 -0.06 0.10 0.02 -0.01 0.01

56 1 0.00 0.00 0.00 -0.22 -0.19 -0.22 -0.02 -0.02 -0.02

103 104 105

A A A

Frequencies -- 1205.0272 1216.9189 1219.9257

Red. masses -- 1.3528 1.9805 1.8940

Frc consts -- 1.1574 1.7280 1.6608

IR Inten -- 30.1752 286.8421 69.0469

Atom AN X Y Z X Y Z X Y Z

1 16 -0.00 0.00 0.01 0.01 -0.00 -0.01 0.01 -0.00 -0.01

2 6 0.02 0.01 0.01 -0.06 -0.03 -0.02 -0.05 -0.03 -0.02

3 6 -0.01 -0.01 -0.00 0.05 0.04 0.02 0.04 0.03 0.02

4 6 0.06 -0.02 -0.01 0.03 0.03 0.00 0.02 0.02 0.00

5 6 0.00 0.01 -0.00 -0.01 -0.01 0.00 -0.00 -0.01 0.00

6 6 0.01 0.00 -0.01 -0.01 -0.00 0.01 -0.00 -0.00 0.01

7 6 -0.00 0.01 -0.00 0.00 -0.00 0.00 0.00 -0.00 -0.00

8 6 -0.00 -0.01 0.01 0.00 0.01 -0.01 0.00 0.00 -0.00

9 1 0.06 0.02 -0.07 -0.04 -0.01 0.05 -0.02 -0.01 0.02

10 6 -0.01 -0.00 0.01 0.00 0.00 -0.00 0.00 0.00 -0.00

11 1 0.00 0.07 -0.03 -0.00 -0.03 0.01 -0.00 -0.01 0.00

12 6 -0.00 0.00 0.00 -0.00 -0.00 0.00 -0.00 -0.00 0.00

13 1 -0.01 -0.10 0.04 0.01 0.06 -0.03 0.00 0.03 -0.02

14 1 -0.05 -0.02 0.06 0.03 0.01 -0.04 0.02 0.01 -0.02

15 1 -0.00 0.00 0.00 -0.00 0.01 0.00 -0.00 0.01 0.00

16 8 0.01 -0.01 -0.01 -0.01 0.01 0.02 -0.01 0.01 0.02

17 8 -0.00 -0.00 -0.00 -0.00 0.00 -0.00 -0.00 0.00 0.00

18 6 -0.01 0.01 -0.01 0.04 -0.07 0.03 0.03 -0.05 0.03

19 1 -0.02 0.05 0.01 0.05 -0.20 -0.03 0.05 -0.16 -0.03

20 6 -0.01 0.03 -0.00 0.03 0.03 -0.01 0.02 0.03 -0.01

21 6 -0.03 0.01 0.00 0.00 -0.02 0.00 0.00 -0.02 0.00

22 6 -0.01 0.01 -0.00 -0.02 0.01 -0.00 -0.01 0.02 -0.00

23 6 -0.02 -0.01 -0.00 -0.04 -0.02 -0.00 -0.03 -0.01 -0.00

24 6 0.03 -0.01 -0.00 -0.01 -0.00 0.00 -0.01 -0.00 0.00

25 1 -0.15 0.23 -0.08 0.02 -0.06 0.01 0.03 -0.07 0.01

26 6 0.00 -0.02 0.00 0.00 -0.00 -0.00 0.00 -0.00 -0.00

27 6 -0.01 -0.01 -0.00 0.01 -0.02 -0.00 0.01 -0.01 -0.00

28 1 -0.21 0.01 0.02 -0.37 0.02 0.03 -0.26 0.01 0.02

29 6 0.00 -0.00 0.00 0.00 -0.00 -0.00 0.00 -0.00 -0.00

30 1 0.43 -0.06 -0.02 0.06 -0.01 -0.01 0.03 -0.01 -0.01

31 1 0.01 -0.05 0.00 -0.03 0.07 0.00 -0.03 0.06 0.00

32 1 0.01 -0.07 -0.01 0.11 -0.24 -0.03 0.08 -0.18 -0.02

33 1 -0.06 -0.09 0.01 -0.08 -0.11 0.02 -0.07 -0.10 0.01

34 8 0.05 0.02 0.00 0.01 -0.01 -0.00 0.01 -0.01 -0.00

35 6 -0.11 -0.01 0.01 -0.02 0.01 0.00 -0.01 0.01 0.00

36 1 0.11 0.58 0.14 0.03 0.11 0.03 0.02 0.06 0.02

37 1 0.18 -0.22 -0.16 0.04 -0.04 -0.03 0.02 -0.02 -0.01

38 1 0.20 -0.29 -0.02 0.04 -0.05 -0.01 0.02 -0.03 -0.01

39 6 0.00 -0.04 0.03 0.00 0.15 -0.11 -0.00 0.13 -0.09

40 8 0.00 0.00 -0.01 -0.01 -0.01 0.02 -0.01 -0.01 0.02

41 8 0.00 0.00 -0.01 -0.02 -0.06 0.04 -0.01 0.01 0.03

42 6 0.01 0.02 0.00 -0.01 0.01 -0.01 -0.01 -0.11 0.01

43 6 -0.00 0.00 -0.01 0.01 0.00 0.02 0.00 -0.03 0.01

44 6 0.00 0.00 0.00 -0.01 0.03 -0.01 0.00 -0.06 0.01

45 6 0.00 -0.01 0.00 0.00 0.01 -0.00 -0.01 0.04 -0.01

46 1 -0.01 0.01 0.00 0.01 0.01 0.01 -0.00 -0.04 0.01

47 6 -0.00 -0.01 -0.00 0.00 -0.02 0.01 0.01 0.06 0.01

48 6 0.00 0.00 -0.00 -0.01 -0.01 -0.01 0.01 0.00 0.01

49 1 -0.01 -0.03 -0.00 -0.01 -0.01 -0.01 0.07 0.21 0.05

50 1 0.01 -0.03 0.01 0.06 -0.19 0.08 -0.10 0.40 -0.16

51 1 -0.01 0.00 -0.01 -0.05 -0.01 -0.05 0.05 0.00 0.05

52 8 -0.00 -0.01 -0.00 -0.01 0.05 -0.01 0.01 -0.05 0.02

53 6 -0.00 0.01 -0.00 0.03 -0.10 0.04 -0.03 0.09 -0.04

54 1 0.02 -0.01 0.03 -0.18 0.07 -0.29 0.16 -0.07 0.26

55 1 0.03 -0.01 0.02 -0.27 0.08 -0.20 0.25 -0.09 0.17

56 1 -0.03 -0.03 -0.03 0.34 0.29 0.33 -0.33 -0.29 -0.33

106 107 108

A A A

Frequencies -- 1251.0493 1256.4830 1280.1848

Red. masses -- 2.1670 12.5135 1.9823

Frc consts -- 1.9983 11.6397 1.9141

IR Inten -- 14.6079 133.0780 62.9246

Atom AN X Y Z X Y Z X Y Z

1 16 -0.00 0.01 0.01 -0.02 0.23 0.33 0.00 0.00 0.00

2 6 -0.01 0.01 -0.00 -0.06 -0.04 -0.05 -0.02 0.00 -0.01

3 6 0.03 -0.09 -0.01 0.01 0.05 0.02 0.11 -0.05 0.01

4 6 -0.02 0.01 0.01 0.07 -0.01 -0.00 0.09 0.01 -0.00

5 6 0.00 0.00 -0.00 0.00 0.04 -0.03 -0.00 -0.00 -0.00

6 6 -0.00 0.00 -0.00 0.02 0.02 -0.02 -0.00 0.00 0.00

7 6 -0.00 -0.00 0.00 -0.00 -0.04 0.02 0.00 0.00 0.00

8 6 -0.00 0.00 0.00 -0.01 -0.00 0.01 0.00 0.00 -0.00

9 1 -0.01 -0.00 0.01 -0.03 0.01 0.03 -0.00 -0.00 0.01

10 6 0.00 0.00 -0.00 0.01 0.00 -0.01 -0.00 0.00 0.00

11 1 -0.00 -0.01 0.00 -0.02 -0.14 0.05 -0.00 -0.00 -0.00

12 6 0.00 0.00 -0.00 -0.00 0.01 -0.00 0.00 -0.00 -0.00

13 1 -0.00 0.00 -0.00 -0.01 -0.01 0.02 0.00 0.00 -0.00

14 1 0.01 0.00 -0.01 0.05 0.02 -0.06 0.00 0.00 -0.01

15 1 0.00 -0.00 -0.00 0.06 -0.08 -0.05 -0.00 0.00 0.00

16 8 0.01 -0.01 -0.02 0.23 -0.24 -0.38 -0.00 0.00 0.00

17 8 -0.01 -0.01 -0.01 -0.21 -0.20 -0.25 -0.00 -0.00 -0.00

18 6 0.01 0.01 0.00 0.03 -0.08 0.01 0.01 -0.03 0.01

19 1 0.01 0.02 0.00 -0.00 0.32 0.05 0.02 -0.15 -0.03

20 6 0.00 0.11 -0.02 0.03 0.02 -0.00 -0.14 -0.08 0.01

21 6 -0.03 -0.06 0.02 0.00 -0.01 -0.00 0.03 0.06 -0.01

22 6 -0.06 0.24 -0.00 0.03 -0.05 -0.00 -0.10 0.01 0.00

23 6 -0.01 -0.05 -0.00 -0.00 0.00 -0.00 0.01 -0.04 -0.01

24 6 -0.02 -0.00 0.00 -0.01 0.00 0.00 0.03 -0.02 -0.00

25 1 -0.01 -0.14 -0.01 0.07 -0.13 0.03 -0.23 0.58 -0.11

26 6 0.03 0.00 -0.00 -0.02 -0.01 0.00 0.05 -0.00 0.00

27 6 -0.05 -0.04 0.01 -0.00 -0.01 -0.00 0.02 0.02 0.00

28 1 0.55 -0.10 -0.08 -0.26 0.03 0.02 0.02 -0.04 -0.03

29 6 0.06 -0.03 -0.00 -0.01 0.01 0.00 0.01 0.01 -0.00

30 1 0.20 -0.04 -0.04 -0.03 0.01 0.00 0.16 -0.03 -0.01

31 1 0.16 -0.26 0.02 -0.12 0.21 -0.00 0.24 -0.40 0.00

32 1 0.11 -0.46 -0.04 -0.06 0.13 0.02 -0.15 0.39 0.05

33 1 -0.18 -0.37 0.05 0.01 0.04 -0.01 -0.02 -0.03 0.01

34 8 -0.01 0.01 0.00 -0.04 0.02 0.01 -0.06 0.06 0.02

35 6 0.01 -0.00 -0.00 0.04 -0.03 -0.01 0.03 -0.04 -0.01

36 1 -0.04 -0.14 -0.03 -0.00 -0.12 -0.03 -0.01 -0.13 -0.04

37 1 -0.03 0.02 0.02 -0.10 0.12 0.07 -0.08 0.10 0.05

38 1 -0.02 0.04 0.01 -0.11 0.11 0.01 -0.09 0.11 0.02

39 6 -0.00 -0.01 0.01 0.02 0.05 -0.02 -0.00 0.04 -0.03

40 8 0.00 0.00 -0.00 -0.01 0.00 0.01 -0.00 -0.00 0.01

41 8 0.00 -0.00 -0.00 -0.00 -0.01 0.01 -0.00 -0.01 0.01

42 6 0.00 0.01 0.00 -0.01 -0.01 -0.00 0.00 -0.01 0.01

43 6 -0.00 0.00 -0.00 0.01 0.00 0.01 -0.00 -0.01 0.00

44 6 0.00 0.00 0.00 -0.01 -0.01 -0.01 0.01 -0.00 0.01

45 6 0.00 -0.00 0.00 -0.00 0.00 -0.00 -0.00 0.01 -0.00

46 1 -0.01 0.00 -0.00 0.02 -0.03 0.02 -0.03 0.05 -0.03

47 6 -0.00 -0.00 -0.00 0.00 0.01 0.00 0.00 -0.00 0.00

48 6 -0.00 0.00 -0.00 0.00 -0.00 0.00 -0.00 0.00 -0.01

49 1 -0.00 -0.01 -0.00 0.00 0.01 0.00 0.01 0.04 0.01

50 1 0.00 -0.02 0.01 -0.01 0.04 -0.02 0.00 0.00 0.00

51 1 -0.00 0.00 -0.00 0.00 -0.00 0.01 -0.01 0.01 -0.01

52 8 -0.00 -0.00 -0.00 0.01 -0.00 0.01 -0.01 0.00 -0.01

53 6 0.00 0.00 0.00 -0.00 -0.00 -0.00 0.00 0.00 0.01

54 1 -0.00 0.00 -0.00 0.00 -0.01 0.01 -0.01 0.00 -0.01

55 1 -0.00 0.00 -0.00 0.01 -0.01 0.01 -0.01 0.00 -0.01

56 1 0.00 0.00 0.00 -0.01 -0.01 -0.01 -0.00 -0.00 -0.00

109 110 111

A A A

Frequencies -- 1292.7121 1312.5529 1318.6966

Red. masses -- 2.1225 2.4158 2.2789

Frc consts -- 2.0897 2.4522 2.3349

IR Inten -- 91.7565 158.2403 182.5124

Atom AN X Y Z X Y Z X Y Z

1 16 -0.00 0.00 0.00 0.00 -0.01 -0.01 -0.00 0.00 0.00

2 6 -0.00 -0.00 -0.00 -0.01 0.00 -0.00 0.01 0.01 0.00

3 6 -0.01 0.00 -0.00 0.06 -0.13 -0.01 -0.00 0.00 -0.00

4 6 -0.01 -0.00 0.00 0.13 -0.06 -0.01 -0.01 -0.00 -0.00

5 6 -0.00 0.00 0.00 0.00 -0.01 -0.00 -0.00 0.00 0.00

6 6 0.00 0.00 -0.00 -0.00 -0.00 0.01 0.00 -0.00 -0.00

7 6 -0.00 -0.00 0.00 0.00 0.01 -0.00 0.00 -0.00 0.00

8 6 -0.00 -0.00 0.00 0.00 0.00 -0.00 -0.00 -0.00 0.00

9 1 0.00 0.00 -0.00 -0.00 -0.00 0.00 0.00 0.00 -0.00

10 6 0.00 -0.00 -0.00 -0.00 0.00 0.00 0.00 -0.00 -0.00

11 1 0.00 -0.00 0.00 -0.00 0.00 -0.00 0.00 -0.00 0.00

12 6 -0.00 0.00 0.00 0.00 -0.00 -0.00 -0.00 0.00 0.00

13 1 -0.00 -0.00 0.00 0.00 -0.00 0.00 0.00 0.00 -0.00

14 1 -0.00 -0.00 0.00 0.00 0.00 -0.01 -0.00 -0.00 0.00

15 1 0.00 -0.00 -0.00 -0.01 0.01 0.00 0.00 -0.00 -0.00

16 8 0.00 -0.00 -0.00 -0.01 0.01 0.02 0.00 -0.00 -0.00

17 8 -0.00 -0.00 -0.00 0.01 0.01 0.01 -0.00 -0.00 -0.00

18 6 0.00 0.00 0.00 0.01 0.02 0.00 -0.01 0.01 -0.00

19 1 0.00 0.02 0.00 0.01 0.04 0.00 -0.01 -0.00 -0.00

20 6 0.01 0.01 -0.00 -0.07 0.19 -0.02 0.00 0.00 -0.00

21 6 -0.00 -0.01 0.00 -0.02 0.01 0.00 -0.00 -0.00 0.00

22 6 0.01 -0.00 -0.00 0.07 -0.02 -0.01 0.00 -0.00 -0.00

23 6 -0.00 0.00 0.00 0.01 0.04 0.01 0.00 0.00 0.00

24 6 -0.01 0.00 0.00 -0.04 -0.04 0.01 -0.00 -0.00 0.00

25 1 0.03 -0.07 0.02 0.21 -0.46 0.08 0.01 -0.01 0.00

26 6 -0.00 -0.00 -0.00 0.01 -0.05 0.00 -0.00 0.00 -0.00

27 6 -0.00 -0.00 -0.00 -0.01 -0.07 -0.01 0.00 0.00 0.00

28 1 -0.01 0.00 0.00 -0.13 0.06 0.00 0.02 0.00 0.00

29 6 -0.00 -0.00 0.00 -0.02 0.01 0.00 -0.00 -0.00 0.00

30 1 -0.03 0.01 0.00 0.38 -0.10 -0.04 -0.00 -0.00 0.00

31 1 -0.03 0.05 -0.00 -0.23 0.43 -0.01 -0.00 0.01 -0.00

32 1 0.01 -0.03 -0.00 -0.17 0.30 0.05 0.01 -0.01 -0.00

33 1 0.01 0.01 -0.00 0.05 0.12 -0.02 0.00 0.01 -0.00

34 8 0.00 -0.00 -0.00 -0.08 0.09 0.03 0.00 -0.00 -0.00

35 6 -0.00 0.00 0.00 0.02 -0.06 -0.02 -0.00 0.00 0.00

36 1 0.00 0.01 0.00 0.03 -0.03 -0.01 -0.00 0.00 0.00

37 1 0.01 -0.01 -0.00 -0.10 0.14 0.05 0.00 -0.01 -0.00

38 1 0.01 -0.01 -0.00 -0.10 0.15 0.05 0.01 -0.01 -0.00

39 6 -0.00 -0.00 0.01 -0.00 -0.01 0.00 -0.00 -0.03 0.02

40 8 -0.00 0.00 0.00 0.00 -0.00 -0.00 0.00 0.00 -0.00

41 8 -0.01 0.04 -0.02 -0.00 -0.00 0.00 0.01 0.02 -0.00

42 6 0.07 -0.09 0.09 -0.00 0.01 -0.00 -0.06 -0.01 -0.06

43 6 -0.03 -0.11 -0.02 -0.00 0.00 -0.00 -0.01 -0.02 -0.01

44 6 0.12 0.06 0.11 0.00 0.00 0.00 0.10 -0.15 0.12

45 6 -0.02 0.04 -0.03 0.00 -0.00 0.00 0.04 0.06 0.03

46 1 -0.31 0.63 -0.41 0.00 -0.01 0.01 0.05 -0.20 0.08

47 6 0.01 -0.07 0.01 -0.00 -0.00 -0.00 0.06 -0.09 0.07

48 6 -0.05 0.03 -0.05 0.00 0.00 -0.00 -0.02 0.07 -0.03

49 1 0.09 0.30 0.05 -0.00 -0.01 -0.00 -0.09 -0.23 -0.07

50 1 0.09 -0.30 0.13 -0.00 0.00 -0.00 -0.19 0.62 -0.28

51 1 0.04 0.03 0.04 -0.00 0.00 -0.00 -0.27 0.08 -0.29

52 8 -0.07 0.00 -0.08 -0.00 0.00 -0.00 -0.08 0.08 -0.09

53 6 0.03 0.03 0.03 0.00 -0.00 0.00 0.05 -0.00 0.06

54 1 -0.04 0.01 -0.01 -0.00 0.00 -0.00 -0.14 0.05 -0.16

55 1 -0.01 0.01 -0.04 -0.00 0.00 -0.00 -0.15 0.05 -0.15

56 1 -0.03 -0.04 -0.03 0.00 0.00 0.00 -0.01 -0.07 -0.00

112 113 114

A A A

Frequencies -- 1338.2725 1354.8967 1359.2766

Red. masses -- 1.6968 3.3242 3.3721

Frc consts -- 1.7904 3.5954 3.6708

IR Inten -- 3.7520 214.3767 10.3149

Atom AN X Y Z X Y Z X Y Z

1 16 0.00 -0.01 -0.00 -0.00 -0.01 -0.01 -0.00 0.01 0.01

2 6 -0.00 0.00 0.00 0.00 -0.05 -0.01 -0.00 0.01 -0.00

3 6 0.00 -0.00 0.00 -0.16 0.12 -0.01 0.05 -0.03 0.00

4 6 0.00 -0.00 0.00 0.22 -0.07 -0.02 -0.07 0.02 0.01

5 6 -0.11 0.15 0.08 0.02 -0.02 -0.01 0.06 -0.08 -0.04

6 6 0.02 -0.03 -0.02 -0.04 -0.02 0.05 -0.13 -0.06 0.16

7 6 0.02 -0.02 -0.02 0.01 0.06 -0.03 0.03 0.20 -0.09

8 6 0.01 -0.09 0.02 0.01 0.04 -0.02 0.02 0.14 -0.06

9 1 0.34 0.07 -0.41 0.11 0.03 -0.13 0.35 0.08 -0.41

10 6 0.07 -0.01 -0.07 -0.03 -0.01 0.03 -0.09 -0.04 0.11

11 1 -0.02 -0.48 0.16 -0.01 -0.15 0.05 -0.02 -0.49 0.18

12 6 -0.01 0.01 0.01 0.03 -0.05 -0.02 0.11 -0.15 -0.08

13 1 0.05 0.37 -0.17 0.00 0.03 -0.02 0.01 0.06 -0.04

14 1 -0.27 -0.11 0.34 -0.03 -0.01 0.03 -0.06 -0.03 0.08

15 1 -0.09 0.12 0.06 -0.07 0.09 0.05 -0.21 0.27 0.14

16 8 0.00 0.00 -0.00 -0.01 0.01 0.01 0.01 -0.01 -0.01

17 8 -0.00 0.00 0.00 0.01 0.01 0.01 -0.01 -0.01 -0.01

18 6 0.00 -0.00 -0.00 0.00 0.00 0.00 -0.00 -0.00 -0.00

19 1 -0.00 -0.03 0.00 -0.00 0.43 0.04 0.00 -0.10 -0.01

20 6 -0.00 0.00 -0.00 0.14 -0.05 0.01 -0.04 0.01 -0.00

21 6 -0.00 0.00 -0.00 -0.01 -0.07 0.01 0.01 0.02 -0.00

22 6 0.00 -0.00 -0.00 0.01 0.09 0.00 -0.01 -0.03 -0.00

23 6 -0.00 0.00 0.00 0.09 -0.06 -0.02 -0.03 0.02 0.01

24 6 -0.00 -0.00 0.00 0.02 0.03 -0.01 -0.00 -0.01 0.00

25 1 0.00 -0.01 0.00 -0.08 0.07 -0.02 0.02 -0.01 0.00

26 6 0.00 -0.00 0.00 -0.05 0.04 -0.00 0.01 -0.01 0.00

27 6 0.00 -0.00 -0.00 -0.12 -0.04 0.01 0.04 0.01 -0.00

28 1 0.00 0.00 -0.00 -0.38 -0.03 0.03 0.13 0.01 -0.01

29 6 -0.00 -0.00 0.00 0.04 -0.02 -0.00 -0.01 0.01 0.00

30 1 0.01 -0.00 -0.00 -0.27 0.07 0.02 0.07 -0.02 -0.01

31 1 -0.00 0.01 -0.00 0.07 -0.21 0.01 -0.02 0.06 -0.00

32 1 -0.00 0.00 0.00 -0.16 0.01 0.01 0.05 -0.01 -0.00

33 1 0.00 0.00 -0.00 -0.09 -0.20 0.03 0.03 0.06 -0.01

34 8 -0.00 0.00 -0.00 -0.10 0.05 0.02 0.04 -0.02 -0.00

35 6 0.00 -0.00 0.00 0.03 -0.06 -0.02 -0.01 0.02 0.01

36 1 0.00 -0.00 0.00 0.09 0.12 0.03 -0.03 -0.04 -0.01

37 1 -0.00 0.01 0.00 -0.13 0.23 0.07 0.04 -0.08 -0.02

38 1 -0.00 0.00 0.00 -0.13 0.22 0.08 0.04 -0.07 -0.03

39 6 -0.00 0.00 -0.00 0.02 0.00 0.01 -0.01 -0.00 -0.00

40 8 -0.00 -0.00 0.00 -0.00 -0.00 -0.00 0.00 0.00 0.00

41 8 -0.00 -0.00 0.00 -0.00 -0.00 0.00 0.00 0.00 -0.00

42 6 0.00 0.00 0.00 -0.00 0.00 -0.00 0.00 -0.00 0.00

43 6 0.00 0.00 0.00 -0.00 -0.00 0.00 -0.00 0.00 -0.00

44 6 -0.00 0.00 -0.00 0.00 0.00 0.00 -0.00 -0.00 -0.00

45 6 -0.00 -0.00 -0.00 0.00 0.00 0.00 -0.00 -0.00 -0.00

46 1 -0.00 0.00 -0.00 0.01 -0.01 -0.00 -0.00 0.00 -0.00

47 6 -0.00 0.00 -0.00 0.00 -0.01 0.00 -0.00 0.00 -0.00

48 6 0.00 -0.00 0.00 -0.00 0.00 -0.00 0.00 0.00 0.00

49 1 0.00 0.00 0.00 -0.01 -0.02 -0.00 0.00 0.01 0.00

50 1 0.00 -0.00 0.00 -0.01 0.01 -0.01 0.00 -0.00 0.00

51 1 0.00 -0.00 0.00 -0.01 0.00 -0.01 -0.00 0.00 -0.00

52 8 0.00 -0.00 0.00 -0.00 0.00 -0.00 -0.00 0.00 -0.00

53 6 -0.00 -0.00 -0.00 0.00 0.00 0.00 0.00 -0.00 0.00

54 1 0.00 -0.00 0.00 -0.00 0.00 0.00 -0.00 0.00 -0.00

55 1 0.00 -0.00 0.00 0.00 0.00 -0.00 -0.00 0.00 -0.00

56 1 0.00 0.00 0.00 -0.00 -0.00 -0.00 0.00 0.00 0.00

115 116 117

A A A

Frequencies -- 1364.8768 1388.3516 1400.0865

Red. masses -- 6.7739 6.4570 2.9605

Frc consts -- 7.4350 7.3330 3.4192

IR Inten -- 51.6137 5.1144 63.7580

Atom AN X Y Z X Y Z X Y Z

1 16 -0.00 0.00 -0.00 -0.00 0.00 0.00 -0.00 -0.01 -0.01

2 6 -0.00 -0.00 -0.00 0.02 0.01 0.01 -0.04 -0.03 -0.02

3 6 0.01 -0.00 0.00 0.00 -0.08 -0.02 -0.07 -0.14 -0.04

4 6 -0.01 0.00 0.00 0.10 0.14 0.03 0.04 0.22 0.06

5 6 0.00 -0.00 -0.00 0.00 -0.00 -0.00 0.00 0.00 0.00

6 6 -0.00 -0.00 0.00 -0.00 -0.00 0.00 0.00 0.00 -0.00

7 6 0.00 0.00 -0.00 0.00 0.00 -0.00 -0.00 -0.00 0.00

8 6 0.00 0.00 -0.00 0.00 0.00 -0.00 -0.00 -0.00 0.00

9 1 0.00 0.00 -0.00 0.00 0.00 -0.00 -0.00 0.00 -0.00

10 6 -0.00 -0.00 0.00 -0.00 -0.00 0.00 0.00 0.00 -0.00

11 1 -0.00 -0.00 0.00 0.00 -0.00 0.00 0.00 -0.01 0.00

12 6 0.00 -0.00 -0.00 0.00 -0.00 -0.00 -0.00 0.00 0.00

13 1 -0.00 -0.00 0.00 -0.00 -0.00 0.00 0.00 0.01 -0.00

14 1 -0.00 -0.00 0.00 0.00 0.00 -0.00 -0.01 -0.00 0.01

15 1 -0.00 0.00 0.00 0.00 -0.00 -0.00 -0.00 0.00 0.00

16 8 0.00 -0.00 -0.00 0.00 -0.00 -0.00 -0.00 0.00 0.00

17 8 0.00 0.00 0.00 -0.00 -0.00 -0.00 0.01 0.01 0.01

18 6 -0.00 -0.00 0.00 -0.00 -0.01 -0.00 0.03 0.02 0.01

19 1 0.00 -0.01 -0.01 -0.00 -0.09 -0.01 0.03 0.58 0.06

20 6 -0.01 0.00 -0.00 -0.24 -0.21 0.01 0.01 -0.00 0.00

21 6 0.00 0.00 -0.00 -0.07 0.16 -0.02 0.09 -0.12 0.02

22 6 -0.00 -0.00 -0.00 0.41 0.17 -0.00 0.02 0.04 -0.01

23 6 -0.00 0.00 0.00 -0.12 -0.01 0.01 -0.10 0.02 0.01

24 6 0.00 -0.00 0.00 0.17 -0.03 -0.01 -0.08 0.06 -0.00

25 1 -0.00 0.01 0.00 -0.05 0.13 0.01 -0.17 0.41 -0.10

26 6 0.00 -0.00 0.00 -0.12 0.16 0.00 -0.02 -0.11 0.01

27 6 0.00 0.00 -0.00 -0.03 -0.11 -0.01 0.08 -0.10 -0.02

28 1 0.02 0.00 -0.00 -0.11 -0.01 -0.02 -0.21 0.04 -0.01

29 6 -0.00 0.00 0.00 -0.08 -0.17 0.02 0.04 0.10 -0.01

30 1 0.01 -0.00 -0.00 -0.09 0.00 0.04 -0.07 0.06 -0.03

31 1 0.00 0.00 -0.00 -0.16 0.26 -0.08 -0.18 0.21 0.01

32 1 0.01 0.00 -0.00 0.16 -0.57 -0.03 -0.05 0.22 0.03

33 1 0.00 0.01 -0.00 0.06 0.01 -0.01 -0.10 -0.07 0.01

34 8 0.00 -0.00 -0.00 -0.02 -0.00 -0.00 0.02 -0.05 -0.01

35 6 -0.00 0.00 0.00 0.02 -0.00 -0.00 0.02 0.04 0.01

36 1 -0.00 -0.00 -0.00 -0.01 -0.07 -0.02 -0.06 -0.19 -0.05

37 1 0.01 -0.01 -0.00 -0.02 -0.00 0.03 0.03 -0.12 0.01

38 1 0.01 -0.01 -0.00 -0.03 0.01 -0.02 0.02 -0.09 -0.08

39 6 -0.01 0.00 -0.00 -0.00 -0.00 0.00 0.02 -0.01 0.02

40 8 0.01 -0.00 -0.00 0.00 0.00 -0.00 0.00 -0.00 -0.01

41 8 0.00 -0.01 0.02 0.00 0.00 -0.00 -0.00 -0.00 -0.00

42 6 -0.23 0.01 -0.25 -0.00 -0.00 -0.00 -0.00 0.00 0.00

43 6 0.10 -0.16 0.12 0.00 -0.00 0.00 -0.00 0.00 -0.00

44 6 0.07 0.35 0.03 -0.00 -0.00 -0.00 0.00 0.00 0.00

45 6 0.14 0.22 0.12 -0.00 0.00 -0.00 0.00 0.00 0.00

46 1 0.10 -0.13 0.11 0.00 0.00 -0.00 0.00 -0.01 0.00

47 6 0.04 -0.20 0.07 0.00 -0.00 0.00 0.00 -0.00 0.00

48 6 -0.19 -0.06 -0.18 -0.00 0.00 -0.00 0.00 0.00 0.00

49 1 -0.13 -0.39 -0.09 0.00 0.00 0.00 -0.00 -0.01 -0.00

50 1 -0.02 -0.07 0.00 -0.00 0.00 -0.00 -0.00 0.01 -0.01

51 1 0.25 -0.08 0.27 0.00 0.00 0.00 -0.01 0.00 -0.01

52 8 0.06 -0.07 0.07 0.00 0.00 0.00 -0.00 0.00 -0.00

53 6 -0.05 0.03 -0.05 -0.00 -0.00 -0.00 0.00 0.00 0.00

54 1 0.13 -0.05 0.17 -0.00 0.00 -0.00 -0.00 -0.00 -0.00

55 1 0.16 -0.06 0.14 -0.00 -0.00 -0.00 0.00 -0.00 -0.00

56 1 -0.09 -0.03 -0.10 -0.00 -0.00 -0.00 -0.00 -0.00 -0.00

118 119 120

A A A

Frequencies -- 1420.5893 1464.0835 1483.8226

Red. masses -- 1.5385 2.0461 1.4759

Frc consts -- 1.8293 2.5841 1.9146

IR Inten -- 33.4489 46.4153 15.1797

Atom AN X Y Z X Y Z X Y Z

1 16 -0.00 -0.01 -0.01 0.00 0.00 -0.00 0.00 -0.00 -0.00

2 6 -0.11 -0.06 -0.05 0.02 0.00 0.01 -0.00 -0.00 -0.00

3 6 0.10 0.04 0.03 -0.05 0.04 -0.01 0.02 0.02 0.01

4 6 -0.04 -0.04 -0.01 0.08 0.02 0.00 -0.02 -0.03 -0.00

5 6 0.00 0.00 -0.00 -0.00 0.00 0.00 -0.01 0.02 0.01

6 6 0.00 0.00 -0.00 0.00 -0.00 0.00 0.00 -0.01 0.00

7 6 -0.00 -0.00 0.00 0.00 -0.00 -0.00 0.01 0.00 -0.01

8 6 -0.00 -0.00 0.00 0.00 0.00 -0.00 0.01 0.01 -0.01

9 1 -0.01 0.00 0.00 0.00 -0.00 -0.00 0.02 -0.01 -0.02

10 6 0.00 0.00 -0.00 -0.00 -0.00 0.00 -0.01 -0.01 0.01

11 1 -0.00 -0.00 0.00 -0.00 0.00 -0.00 0.01 -0.03 -0.00

12 6 -0.00 0.00 0.00 -0.00 0.00 0.00 -0.01 0.01 0.01

13 1 -0.00 0.01 -0.00 0.00 -0.00 0.00 0.00 -0.06 0.01

14 1 -0.01 -0.00 0.01 0.00 0.00 -0.01 0.04 -0.00 -0.04

15 1 -0.01 0.01 0.00 0.00 -0.01 -0.00 0.05 -0.07 -0.04

16 8 -0.01 0.01 0.01 0.00 0.00 0.00 -0.00 0.00 0.00

17 8 0.01 0.01 0.01 -0.00 0.00 0.00 -0.00 0.00 -0.00

18 6 0.03 -0.03 0.01 -0.01 -0.01 -0.00 0.00 -0.00 0.00

19 1 0.02 0.94 0.09 -0.01 -0.06 -0.01 0.00 -0.02 -0.00

20 6 -0.03 -0.02 0.00 0.02 -0.10 0.01 -0.04 0.04 -0.00

21 6 -0.02 0.05 -0.01 0.07 0.01 -0.01 0.01 -0.06 0.01

22 6 -0.01 -0.02 0.00 -0.06 0.06 -0.00 0.05 -0.03 -0.00

23 6 -0.01 -0.01 -0.00 -0.06 -0.06 -0.00 -0.07 0.03 0.01

24 6 0.02 -0.02 0.00 -0.10 0.07 0.00 0.04 -0.00 -0.00

25 1 0.05 -0.08 0.01 0.06 0.06 0.00 -0.12 0.18 -0.04

26 6 0.01 0.03 -0.00 0.10 -0.10 -0.00 -0.02 -0.03 0.00

27 6 -0.00 0.04 0.00 -0.02 0.09 0.01 0.03 0.01 -0.00

28 1 0.13 -0.02 -0.01 0.28 -0.11 -0.05 0.24 -0.00 -0.02

29 6 -0.02 -0.03 0.00 -0.06 -0.06 0.01 0.01 0.06 -0.01

30 1 0.08 -0.02 0.00 0.36 0.02 -0.05 -0.23 0.03 0.02

31 1 0.04 -0.02 -0.01 -0.14 0.42 -0.01 -0.06 0.03 0.00

32 1 0.06 -0.10 -0.01 0.13 -0.27 -0.04 0.08 -0.08 -0.01

33 1 0.05 0.06 -0.01 0.30 0.44 -0.06 -0.12 -0.10 0.02

34 8 0.01 0.00 0.00 -0.02 -0.01 -0.00 0.01 -0.02 -0.01

35 6 -0.01 -0.00 0.00 0.00 -0.03 -0.01 -0.01 -0.07 -0.02

36 1 -0.00 0.02 0.01 0.06 0.12 0.04 0.16 0.33 0.15

37 1 0.02 0.01 -0.02 -0.04 0.19 -0.00 -0.07 0.45 -0.04

38 1 0.03 -0.01 0.02 -0.01 0.14 0.10 0.03 0.31 0.26

39 6 0.04 0.01 0.01 -0.00 0.00 -0.00 0.00 0.01 -0.01

40 8 -0.00 -0.00 -0.00 -0.00 -0.00 0.00 -0.00 -0.00 0.00

41 8 -0.00 -0.01 0.00 -0.00 -0.00 0.00 -0.00 -0.00 -0.00

42 6 0.00 0.00 0.00 -0.00 0.00 -0.00 0.03 -0.01 0.03

43 6 -0.00 -0.00 -0.00 0.00 -0.00 0.00 -0.02 -0.02 -0.02

44 6 -0.00 -0.00 -0.00 -0.00 -0.00 -0.00 -0.00 0.02 -0.01

45 6 0.00 0.00 -0.00 -0.00 -0.00 -0.00 0.00 0.04 -0.00

46 1 -0.00 0.00 -0.00 -0.00 -0.00 0.00 -0.03 0.00 -0.03

47 6 0.00 -0.00 0.00 0.00 0.00 0.00 -0.01 -0.03 -0.00

48 6 0.00 0.00 0.00 -0.00 0.00 -0.00 0.03 -0.00 0.03

49 1 -0.00 -0.00 -0.00 0.00 0.00 0.00 -0.06 -0.10 -0.05

50 1 -0.00 0.01 -0.01 0.00 -0.00 0.00 -0.05 0.08 -0.06

51 1 -0.01 0.00 -0.01 0.00 0.00 0.00 -0.13 -0.00 -0.13

52 8 -0.00 0.00 -0.00 0.00 0.00 0.00 -0.01 -0.01 -0.01

53 6 0.00 0.00 0.00 0.00 -0.00 -0.00 -0.03 -0.01 -0.03

54 1 -0.00 -0.00 -0.00 -0.00 -0.00 0.00 0.19 0.02 0.11

55 1 -0.00 -0.00 -0.00 -0.00 -0.00 -0.00 0.10 0.03 0.19

56 1 -0.00 -0.00 -0.00 -0.00 -0.00 0.00 0.12 0.17 0.12

121 122 123

A A A

Frequencies -- 1483.9795 1484.7766 1501.1486

Red. masses -- 1.4521 2.1457 1.7609

Frc consts -- 1.8842 2.7870 2.3379

IR Inten -- 30.2670 18.6101 21.4734

Atom AN X Y Z X Y Z X Y Z

1 16 -0.00 0.00 0.00 -0.00 0.01 0.00 -0.00 -0.00 -0.00

2 6 0.00 0.00 0.00 0.00 0.00 -0.00 0.00 0.00 0.00

3 6 -0.01 -0.01 -0.00 0.00 0.01 0.00 -0.00 -0.00 -0.00

4 6 0.01 0.02 0.00 -0.00 -0.01 -0.00 0.00 -0.00 -0.00

5 6 0.01 -0.01 -0.01 0.09 -0.11 -0.06 0.00 0.00 0.00

6 6 -0.00 0.01 -0.00 -0.00 0.10 -0.02 0.00 -0.00 -0.00

7 6 -0.01 -0.00 0.01 -0.07 -0.01 0.08 -0.00 0.00 -0.00

8 6 -0.00 -0.01 0.01 -0.06 -0.08 0.09 -0.00 0.00 0.00

9 1 -0.01 0.01 0.01 -0.13 0.08 0.13 -0.00 -0.00 0.00

10 6 0.00 0.01 -0.01 0.05 0.10 -0.09 0.00 -0.00 -0.00

11 1 -0.00 0.02 0.00 -0.06 0.19 0.01 0.00 -0.00 0.00

12 6 0.01 -0.01 -0.00 0.08 -0.10 -0.05 0.00 -0.00 0.00

13 1 -0.00 0.03 -0.01 -0.03 0.41 -0.09 -0.00 -0.00 0.00

14 1 -0.02 0.00 0.02 -0.27 0.02 0.29 -0.00 -0.00 0.00

15 1 -0.03 0.04 0.02 -0.36 0.48 0.25 -0.00 0.00 0.00

16 8 0.00 -0.00 -0.00 -0.00 -0.00 0.00 -0.00 0.00 0.00

17 8 0.00 -0.00 0.00 0.00 -0.00 -0.00 0.00 0.00 0.00

18 6 0.00 0.00 0.00 -0.00 0.00 -0.00 -0.00 -0.00 -0.00

19 1 0.00 -0.01 0.00 0.00 -0.02 -0.01 -0.00 0.02 0.00

20 6 0.02 -0.02 0.00 -0.01 0.01 -0.00 -0.00 0.00 -0.00

21 6 -0.00 0.03 -0.01 0.00 -0.02 0.00 -0.00 -0.00 0.00

22 6 -0.02 0.02 0.00 0.01 -0.01 -0.00 0.00 0.00 -0.00

23 6 0.03 -0.01 -0.00 -0.02 0.01 0.00 -0.00 0.00 0.00

24 6 -0.02 0.00 0.00 0.01 0.00 -0.00 -0.00 -0.00 0.00

25 1 0.06 -0.09 0.02 -0.03 0.05 -0.01 -0.00 0.00 -0.00

26 6 0.01 0.02 -0.00 -0.01 -0.01 0.00 -0.00 -0.00 0.00

27 6 -0.02 -0.00 0.00 0.01 0.00 -0.00 0.00 -0.00 -0.00

28 1 -0.12 -0.00 0.01 0.07 -0.00 -0.01 0.00 0.00 -0.00

29 6 -0.00 -0.03 0.00 0.00 0.02 -0.00 0.00 0.00 -0.00

30 1 0.12 -0.02 -0.01 -0.06 0.01 0.00 -0.00 0.00 0.00

31 1 0.03 -0.01 -0.00 -0.02 0.01 0.00 -0.00 -0.00 0.00

32 1 -0.04 0.04 0.01 0.02 -0.03 -0.00 -0.00 0.00 0.00

33 1 0.06 0.05 -0.01 -0.03 -0.02 0.00 -0.00 -0.00 0.00

34 8 -0.01 0.01 0.00 0.00 -0.01 -0.00 -0.00 0.00 0.00

35 6 0.01 0.04 0.01 -0.00 -0.02 -0.01 0.00 0.00 0.00

36 1 -0.08 -0.18 -0.08 0.05 0.11 0.04 -0.00 -0.00 -0.00

37 1 0.04 -0.24 0.02 -0.04 0.11 0.01 0.00 -0.00 -0.00

38 1 -0.02 -0.16 -0.14 -0.02 0.08 0.05 -0.00 -0.00 -0.00

39 6 -0.00 0.01 -0.00 -0.00 -0.00 0.00 0.00 -0.01 0.00

40 8 -0.00 -0.00 0.00 -0.00 0.00 0.00 -0.00 0.00 -0.00

41 8 -0.00 0.00 -0.01 0.00 0.00 -0.00 0.00 -0.01 0.00

42 6 0.05 -0.02 0.05 -0.00 0.00 -0.00 -0.03 0.08 -0.04

43 6 -0.03 -0.03 -0.03 0.00 0.00 0.00 0.05 -0.02 0.06

44 6 -0.01 0.04 -0.01 -0.00 -0.00 0.00 -0.02 -0.09 -0.01

45 6 0.00 0.07 -0.01 -0.00 -0.00 0.00 -0.03 -0.10 -0.02

46 1 -0.05 0.00 -0.05 0.00 -0.00 0.00 -0.01 0.19 -0.04

47 6 -0.01 -0.06 -0.01 0.00 0.00 0.00 0.06 -0.02 0.07

48 6 0.05 -0.00 0.05 -0.00 0.00 -0.00 -0.07 0.07 -0.08

49 1 -0.12 -0.18 -0.10 0.00 0.00 0.00 0.19 0.38 0.15

50 1 -0.09 0.15 -0.11 0.00 -0.00 0.00 0.00 0.17 -0.02

51 1 -0.23 -0.00 -0.24 0.00 0.00 0.00 0.23 0.08 0.23

52 8 -0.02 -0.01 -0.01 0.00 0.00 0.00 -0.01 0.02 -0.02

53 6 -0.05 -0.03 -0.05 0.00 0.00 0.00 -0.03 -0.05 -0.04

54 1 0.35 0.04 0.21 -0.00 0.00 -0.00 0.43 0.24 0.06

55 1 0.19 0.06 0.35 -0.00 -0.00 -0.00 0.03 0.29 0.38

56 1 0.23 0.32 0.22 -0.00 -0.00 -0.00 0.11 0.18 0.16

124 125 126

A A A

Frequencies -- 1505.1031 1506.7486 1509.7714

Red. masses -- 1.0470 1.1105 1.0680

Frc consts -- 1.3974 1.4854 1.4343

IR Inten -- 7.3172 71.1505 13.9057

Atom AN X Y Z X Y Z X Y Z

1 16 0.00 -0.00 -0.00 -0.00 0.00 0.00 -0.00 0.00 0.00

2 6 0.00 0.00 0.00 0.00 -0.00 0.00 0.00 0.00 0.00

3 6 -0.00 -0.00 -0.00 -0.00 0.02 0.00 -0.01 -0.01 -0.00

4 6 -0.00 0.00 0.00 0.03 -0.04 -0.01 -0.00 0.02 0.00

5 6 -0.00 0.00 0.00 0.00 0.00 -0.00 0.00 -0.00 -0.00

6 6 0.00 -0.00 0.00 -0.00 0.00 0.00 -0.00 0.00 0.00

7 6 0.00 0.00 -0.00 -0.00 -0.01 0.00 0.00 -0.00 0.00

8 6 0.00 0.00 -0.00 -0.00 -0.01 0.00 -0.00 -0.00 0.00

9 1 -0.00 -0.00 -0.00 0.01 0.01 -0.01 0.00 0.00 -0.00

10 6 -0.00 -0.00 0.00 -0.00 0.00 0.00 -0.00 0.00 0.00

11 1 0.00 -0.00 0.00 0.00 0.02 -0.01 -0.01 0.00 -0.01

12 6 -0.00 0.00 0.00 0.00 -0.00 -0.00 0.00 -0.00 -0.00

13 1 -0.00 -0.00 0.00 0.00 0.03 -0.01 0.00 0.01 -0.00

14 1 0.00 -0.00 -0.00 0.00 0.01 -0.01 0.00 0.00 -0.00

15 1 0.00 -0.00 -0.00 -0.01 0.01 0.00 -0.00 0.01 0.00

16 8 -0.00 0.00 0.00 0.00 -0.00 -0.00 0.00 -0.00 -0.00

17 8 -0.00 0.00 0.00 -0.00 0.00 -0.00 -0.00 -0.00 -0.00

18 6 -0.00 -0.00 0.00 -0.00 -0.00 -0.00 0.00 0.00 0.00

19 1 0.00 -0.00 -0.00 -0.00 -0.02 -0.00 0.00 0.00 0.00

20 6 0.00 -0.00 0.00 -0.01 0.01 -0.00 0.01 -0.01 0.00

21 6 -0.00 0.00 -0.00 0.00 -0.02 0.00 -0.00 0.02 -0.00

22 6 -0.00 0.00 0.00 0.01 -0.00 -0.00 -0.01 0.01 0.00

23 6 0.00 -0.00 0.00 -0.01 0.00 -0.00 0.01 -0.00 -0.00

24 6 -0.00 -0.00 0.00 0.01 0.00 -0.00 -0.01 -0.00 0.00

25 1 0.00 -0.00 0.00 -0.04 0.06 -0.01 0.03 -0.05 0.01

26 6 -0.00 0.00 -0.00 0.00 -0.02 0.00 -0.00 0.02 -0.00

27 6 0.00 -0.00 -0.00 -0.01 0.01 0.00 0.00 -0.01 -0.00

28 1 -0.00 0.00 -0.00 0.02 -0.00 0.01 -0.07 0.01 -0.01

29 6 0.00 -0.00 0.00 -0.01 0.01 -0.00 0.01 -0.01 0.00

30 1 0.00 -0.00 -0.00 -0.06 0.01 0.00 0.06 -0.01 -0.00

31 1 0.00 -0.00 -0.00 -0.03 0.04 -0.00 0.02 -0.03 -0.00

32 1 -0.00 0.00 0.00 0.01 -0.03 -0.00 -0.02 0.04 0.01

33 1 0.00 -0.00 -0.00 -0.01 0.01 0.00 0.02 -0.00 -0.00

34 8 0.00 -0.00 -0.00 -0.03 0.02 0.01 -0.00 -0.00 -0.01

35 6 0.00 -0.00 -0.00 -0.05 0.01 0.02 -0.02 0.01 -0.05

36 1 0.00 0.00 0.00 -0.22 -0.38 -0.27 0.02 -0.28 0.63

37 1 -0.01 0.00 0.00 0.55 0.10 -0.38 -0.21 0.34 0.07

38 1 -0.00 -0.00 -0.00 0.42 0.06 0.28 0.51 -0.26 0.06

39 6 -0.00 -0.00 0.00 0.00 0.00 -0.00 -0.00 -0.00 0.00

40 8 0.00 -0.00 -0.00 -0.00 0.00 0.00 0.00 -0.00 -0.00

41 8 -0.00 -0.00 0.00 -0.00 -0.00 0.00 0.00 0.00 -0.00

42 6 -0.00 0.00 -0.00 -0.00 -0.00 0.00 0.00 -0.00 -0.00

43 6 0.00 -0.00 0.00 -0.00 0.00 -0.00 0.00 0.00 -0.00

44 6 -0.00 -0.00 0.00 0.00 0.00 0.00 0.00 -0.00 0.00

45 6 -0.00 -0.00 -0.00 0.00 0.00 0.00 -0.00 -0.00 -0.00

46 1 -0.00 0.01 -0.00 0.00 -0.00 -0.00 -0.00 0.00 0.00

47 6 0.00 -0.00 0.00 -0.00 0.00 -0.00 0.00 -0.00 0.00

48 6 -0.00 0.00 -0.00 0.00 -0.00 0.00 -0.00 0.00 -0.00

49 1 0.01 0.01 0.01 -0.00 -0.00 -0.00 0.00 0.00 0.00

50 1 -0.02 0.01 0.01 0.00 -0.00 0.00 0.00 0.00 -0.00

51 1 0.01 0.00 0.01 0.00 -0.00 0.00 0.00 0.00 0.00

52 8 -0.01 0.00 0.01 -0.00 -0.00 0.00 -0.00 0.00 -0.00

53 6 -0.04 0.00 0.04 -0.00 -0.00 0.00 0.00 -0.00 -0.00

54 1 -0.22 0.23 -0.35 -0.00 0.00 -0.01 0.00 -0.00 0.00

55 1 0.34 -0.24 0.28 0.00 0.00 0.00 -0.00 0.00 -0.00

56 1 0.53 -0.03 -0.48 0.00 -0.00 -0.01 -0.00 0.00 0.00

127 128 129

A A A

Frequencies -- 1512.7100 1516.4517 1519.9435

Red. masses -- 1.1036 1.7732 2.0586

Frc consts -- 1.4879 2.4025 2.8021

IR Inten -- 42.8981 26.5499 8.5577

Atom AN X Y Z X Y Z X Y Z

1 16 0.00 0.00 0.00 0.00 0.00 0.00 0.00 0.00 -0.00

2 6 -0.00 -0.00 -0.00 -0.00 0.00 0.00 0.00 0.00 0.00

3 6 0.00 0.00 0.00 -0.02 -0.06 -0.01 0.00 0.00 0.00

4 6 0.00 -0.00 -0.00 -0.06 0.12 0.03 0.01 -0.01 -0.00

5 6 -0.00 -0.00 0.00 -0.00 -0.01 0.00 -0.04 -0.08 0.07

6 6 0.00 -0.00 -0.00 0.00 -0.00 -0.01 0.09 -0.02 -0.10

7 6 0.00 0.00 -0.00 -0.00 0.01 -0.00 -0.02 0.14 -0.02

8 6 -0.00 0.00 -0.00 -0.00 0.01 0.00 -0.02 0.13 -0.01

9 1 -0.00 -0.00 0.00 -0.02 -0.01 0.02 -0.30 -0.15 0.38

10 6 0.00 -0.00 -0.00 0.00 -0.00 -0.00 0.09 -0.02 -0.09

11 1 -0.00 -0.00 0.00 -0.00 -0.02 0.01 -0.07 -0.40 0.20

12 6 -0.00 -0.00 0.00 -0.00 -0.01 0.00 -0.04 -0.06 0.06

13 1 -0.00 -0.00 0.00 -0.00 -0.02 0.01 -0.07 -0.40 0.21

14 1 -0.00 -0.00 0.00 -0.02 -0.01 0.02 -0.27 -0.14 0.34

15 1 0.00 -0.00 0.00 -0.01 0.00 0.01 -0.05 -0.07 0.07

16 8 0.00 -0.00 -0.00 0.00 -0.00 -0.00 0.00 -0.00 -0.00

17 8 -0.00 -0.00 -0.00 -0.00 -0.00 -0.00 0.00 0.00 -0.00

18 6 0.00 0.00 0.00 0.00 0.01 0.00 -0.00 -0.00 -0.00

19 1 0.00 -0.00 0.00 0.01 0.02 0.01 -0.00 -0.01 -0.00

20 6 -0.00 0.00 -0.00 0.06 -0.05 0.01 -0.00 0.00 -0.00

21 6 0.00 -0.00 0.00 -0.02 0.09 -0.01 0.00 -0.01 0.00

22 6 -0.00 -0.00 0.00 -0.05 0.01 0.00 0.00 -0.00 -0.00

23 6 -0.00 -0.00 -0.00 0.03 0.01 -0.00 -0.00 -0.00 -0.00

24 6 0.00 0.00 -0.00 -0.07 -0.02 0.01 0.00 0.00 -0.00

25 1 -0.00 0.00 0.00 0.17 -0.27 0.06 -0.01 0.02 -0.00

26 6 0.00 -0.00 0.00 -0.01 0.08 -0.00 0.00 -0.01 0.00

27 6 -0.00 0.00 0.00 0.03 -0.06 -0.01 -0.00 0.00 0.00

28 1 0.00 -0.00 -0.00 -0.21 0.04 0.03 0.01 -0.00 -0.00

29 6 -0.00 -0.00 0.00 0.04 -0.05 0.00 -0.00 0.00 0.00

30 1 -0.00 0.00 -0.00 0.27 -0.07 -0.02 -0.02 0.01 0.00

31 1 -0.00 0.00 -0.00 0.12 -0.19 0.00 -0.01 0.02 0.00

32 1 0.00 -0.00 -0.00 -0.08 0.19 0.02 0.00 -0.01 -0.00

33 1 0.00 0.00 -0.00 0.05 -0.05 -0.00 -0.00 0.00 -0.00

34 8 0.00 0.00 -0.00 0.03 -0.06 -0.01 -0.00 0.01 0.00

35 6 0.00 0.00 -0.00 -0.01 -0.06 0.00 -0.00 0.00 0.00

36 1 0.00 0.00 0.00 0.08 0.27 -0.17 -0.02 -0.05 0.00

37 1 -0.00 -0.00 0.00 0.21 0.37 -0.19 0.02 -0.01 -0.01

38 1 -0.00 -0.00 -0.00 0.02 0.41 0.34 0.04 -0.03 0.00

39 6 -0.00 0.00 -0.00 -0.00 -0.00 0.01 0.00 0.00 -0.00

40 8 0.00 -0.00 0.00 0.00 -0.00 -0.00 -0.00 0.00 0.00

41 8 -0.00 -0.00 -0.00 0.00 0.00 -0.00 -0.00 -0.00 0.00

42 6 0.02 -0.01 0.02 0.00 -0.00 0.00 -0.00 -0.00 -0.00

43 6 -0.01 -0.01 -0.01 -0.00 0.00 -0.00 -0.00 0.00 -0.00

44 6 -0.01 0.03 -0.02 0.00 0.00 0.00 0.00 -0.00 0.00

45 6 0.00 0.03 -0.00 -0.00 -0.00 0.00 0.00 0.00 0.00

46 1 -0.01 -0.01 -0.01 -0.00 0.00 0.00 0.00 -0.00 0.00

47 6 -0.01 -0.01 -0.01 0.00 -0.00 0.00 -0.00 0.00 -0.00

48 6 0.02 -0.01 0.02 -0.00 0.00 -0.00 0.00 -0.00 0.00

49 1 -0.05 -0.08 -0.04 0.00 0.00 0.00 -0.00 -0.00 -0.00

50 1 -0.01 -0.03 -0.00 -0.00 0.00 -0.00 0.00 -0.00 0.00

51 1 -0.07 -0.02 -0.07 0.00 0.00 0.00 0.00 -0.00 0.00

52 8 0.01 -0.02 0.02 -0.00 -0.00 -0.00 -0.00 -0.00 -0.00

53 6 0.02 -0.04 0.02 -0.00 -0.00 -0.00 -0.00 0.00 0.00

54 1 0.20 0.48 -0.33 0.01 0.00 -0.00 -0.00 -0.00 0.00

55 1 -0.27 0.52 0.16 -0.00 0.01 0.00 0.00 -0.00 -0.00

56 1 -0.23 -0.34 -0.23 -0.00 -0.00 0.00 0.00 0.00 0.00

130 131 132

A A A

Frequencies -- 1544.8281 1559.8899 1600.9470

Red. masses -- 2.6268 3.3474 6.9719

Frc consts -- 3.6935 4.7990 10.5283

IR Inten -- 152.5790 91.8301 20.7384

Atom AN X Y Z X Y Z X Y Z

1 16 0.00 0.00 0.00 0.00 0.00 0.00 -0.00 -0.00 -0.00

2 6 -0.01 -0.01 -0.00 -0.02 -0.02 -0.01 -0.01 0.01 0.00

3 6 0.00 0.00 0.00 0.07 0.00 0.01 -0.06 -0.22 -0.04

4 6 -0.00 0.00 0.00 -0.17 -0.02 -0.00 0.15 0.24 0.05

5 6 -0.00 0.00 -0.00 0.00 0.00 -0.00 0.00 0.00 -0.00

6 6 -0.00 0.00 -0.00 -0.00 -0.00 0.00 -0.00 -0.00 0.00

7 6 -0.00 -0.00 0.00 0.00 -0.00 -0.00 0.00 -0.00 0.00

8 6 -0.00 -0.00 0.00 0.00 0.00 -0.00 0.00 0.00 -0.00

9 1 0.00 0.00 -0.00 0.00 0.00 -0.00 0.00 0.00 -0.00

10 6 -0.00 0.00 0.00 -0.00 0.00 0.00 0.00 0.00 -0.00

11 1 0.00 0.00 -0.00 0.00 0.00 0.00 0.00 -0.00 0.00

12 6 0.00 0.00 -0.00 0.00 -0.00 -0.00 -0.00 -0.00 0.00

13 1 0.00 0.00 -0.00 -0.00 0.00 -0.00 0.00 -0.00 0.00

14 1 0.00 0.00 -0.00 0.00 0.00 -0.00 -0.00 -0.00 0.00

15 1 -0.00 0.00 -0.00 -0.00 0.00 0.00 0.00 -0.00 -0.00

16 8 0.00 -0.00 -0.00 -0.00 -0.00 -0.00 -0.00 0.00 0.00

17 8 0.00 -0.00 0.00 -0.00 -0.00 -0.00 -0.00 -0.00 -0.00

18 6 0.00 0.01 0.00 0.01 0.02 0.00 0.01 0.02 0.00

19 1 0.01 0.00 0.00 0.01 0.00 0.00 0.01 0.03 0.01

20 6 -0.00 -0.00 0.00 -0.05 0.06 -0.01 0.17 0.18 -0.01

21 6 0.00 -0.00 0.00 0.02 -0.13 0.02 -0.20 0.02 0.01

22 6 -0.00 0.00 -0.00 -0.03 0.16 0.00 -0.20 -0.16 0.00

23 6 0.00 -0.00 -0.00 0.20 0.02 -0.01 -0.09 -0.10 -0.01

24 6 0.00 0.00 -0.00 0.11 0.08 -0.02 0.36 -0.03 -0.03

25 1 -0.00 0.01 -0.00 -0.21 0.31 -0.07 -0.16 -0.12 -0.01

26 6 0.00 0.00 -0.00 0.09 -0.03 -0.00 0.09 0.05 -0.01

27 6 -0.00 0.00 0.00 -0.05 -0.11 -0.00 0.01 0.15 0.01

28 1 -0.00 -0.00 0.00 -0.44 0.09 0.07 0.08 -0.13 -0.04

29 6 -0.00 -0.00 0.00 -0.14 -0.13 0.02 -0.20 -0.12 0.02

30 1 -0.00 0.00 -0.00 -0.17 0.13 0.00 -0.52 0.08 0.07

31 1 -0.00 0.00 -0.00 -0.05 0.30 -0.01 0.10 0.06 -0.00

32 1 -0.00 -0.00 0.00 -0.20 0.20 0.04 0.13 -0.13 -0.03

33 1 0.00 0.01 -0.00 0.19 0.35 -0.05 0.03 0.22 -0.02

34 8 0.00 -0.00 -0.00 0.03 -0.02 -0.01 -0.02 -0.02 -0.01

35 6 0.00 0.00 0.00 -0.01 -0.02 -0.00 0.01 -0.01 -0.00

36 1 0.00 0.00 0.00 0.05 0.12 0.02 0.00 -0.04 -0.02

37 1 -0.00 -0.00 0.00 0.02 0.12 -0.04 0.01 0.06 -0.01

38 1 -0.00 -0.00 -0.00 0.02 0.10 0.09 0.01 0.05 0.04

39 6 0.00 0.01 -0.00 0.00 -0.00 0.00 -0.00 -0.01 0.01

40 8 0.00 -0.00 -0.00 0.00 0.00 0.00 0.00 -0.00 -0.00

41 8 0.01 0.02 0.01 0.00 -0.00 0.00 0.00 0.00 -0.00

42 6 -0.05 -0.14 -0.03 0.00 0.00 -0.00 0.00 -0.00 0.00

43 6 -0.06 0.15 -0.08 0.00 -0.00 0.00 -0.00 0.00 -0.00

44 6 0.15 -0.04 0.15 -0.00 0.00 -0.00 -0.00 0.00 -0.00

45 6 0.06 -0.02 0.06 -0.00 -0.00 -0.00 -0.00 -0.00 -0.00

46 1 0.13 -0.40 0.20 -0.00 0.01 -0.00 0.00 0.00 -0.00

47 6 -0.04 0.12 -0.05 0.00 -0.00 0.00 0.00 -0.01 0.00

48 6 -0.05 -0.05 -0.05 0.00 0.00 0.00 -0.00 0.00 -0.00

49 1 -0.01 -0.20 0.02 0.00 0.00 0.00 0.00 0.01 0.00

50 1 0.16 -0.41 0.22 -0.00 0.01 -0.00 -0.00 0.01 -0.00

51 1 0.19 -0.06 0.20 -0.00 0.00 -0.00 -0.00 0.00 -0.00

52 8 -0.05 0.01 -0.06 0.00 -0.00 0.00 0.00 -0.00 0.00

53 6 -0.03 -0.02 -0.03 0.00 0.00 0.00 0.00 0.00 0.00

54 1 0.31 0.14 0.08 -0.00 -0.00 -0.00 -0.00 -0.00 -0.00

55 1 0.07 0.16 0.29 -0.00 -0.00 -0.00 -0.00 -0.00 -0.00

56 1 0.10 0.15 0.09 -0.00 -0.00 -0.00 -0.00 -0.00 -0.00

133 134 135

A A A

Frequencies -- 1637.5832 1639.3435 1640.9183

Red. masses -- 6.4293 5.4555 5.8426

Frc consts -- 10.1583 8.6383 9.2689

IR Inten -- 18.9723 1.0210 0.2039

Atom AN X Y Z X Y Z X Y Z

1 16 -0.00 -0.00 -0.00 0.00 -0.00 -0.00 -0.00 0.01 0.00

2 6 0.04 0.08 0.02 0.00 0.01 0.00 -0.00 -0.01 -0.01

3 6 -0.01 0.00 -0.00 -0.00 0.00 -0.00 0.00 0.00 0.00

4 6 0.00 -0.02 -0.00 0.00 -0.00 -0.00 0.00 0.00 0.00

5 6 -0.01 0.01 0.01 -0.11 -0.03 0.12 0.14 -0.25 -0.09

6 6 0.02 0.00 -0.02 0.19 0.10 -0.24 -0.10 0.12 0.07

7 6 0.01 0.00 -0.01 0.09 0.16 -0.15 -0.04 0.28 -0.04

8 6 -0.01 -0.01 0.01 -0.10 -0.18 0.16 0.06 -0.27 0.02

9 1 -0.03 -0.01 0.03 -0.28 -0.03 0.32 0.06 0.18 -0.12

10 6 -0.02 -0.00 0.02 -0.20 -0.09 0.25 0.09 -0.14 -0.05

11 1 0.01 -0.01 -0.01 0.07 -0.27 0.01 -0.09 -0.30 0.19

12 6 0.02 -0.01 -0.02 0.12 0.02 -0.14 -0.18 0.30 0.10

13 1 -0.01 0.02 0.01 -0.06 0.33 -0.03 0.12 0.24 -0.20

14 1 0.02 0.01 -0.03 0.26 0.05 -0.31 0.02 -0.19 0.04

15 1 -0.01 0.03 0.00 -0.01 0.22 -0.05 0.29 -0.31 -0.22

16 8 -0.00 0.00 0.00 -0.00 0.00 0.00 -0.00 -0.01 0.00

17 8 0.00 0.00 0.00 -0.00 0.00 -0.00 0.00 -0.00 -0.00

18 6 -0.03 -0.10 -0.02 -0.00 -0.01 -0.00 0.00 0.02 0.00

19 1 -0.03 0.13 -0.01 -0.00 0.02 0.00 0.01 -0.03 -0.00

20 6 0.01 0.00 -0.00 0.00 -0.00 0.00 0.00 0.00 0.00

21 6 -0.01 -0.00 0.00 -0.00 0.00 -0.00 0.00 -0.00 0.00

22 6 -0.02 0.02 0.00 -0.00 0.00 0.00 -0.00 -0.00 0.00

23 6 -0.03 0.02 0.01 -0.01 0.00 0.00 -0.00 0.00 0.00

24 6 0.02 0.00 -0.00 0.00 -0.00 -0.00 -0.00 0.00 -0.00

25 1 -0.01 -0.01 -0.00 -0.00 -0.00 0.00 -0.00 0.00 -0.00

26 6 0.01 0.00 -0.00 0.00 -0.00 0.00 -0.00 0.01 -0.00

27 6 0.03 -0.04 -0.01 0.01 -0.01 -0.00 0.00 -0.00 -0.00

28 1 0.04 0.02 -0.00 0.01 0.00 -0.00 0.00 0.00 -0.00

29 6 -0.01 -0.01 0.00 -0.00 -0.00 0.00 -0.00 -0.00 0.00

30 1 -0.02 0.01 0.00 -0.01 0.00 0.00 0.00 0.00 -0.00

31 1 0.01 0.01 0.00 -0.00 0.00 0.00 0.00 -0.00 -0.00

32 1 -0.01 0.07 0.01 -0.00 0.01 0.00 0.00 0.00 -0.00

33 1 0.01 0.02 -0.00 -0.00 0.00 -0.00 0.01 0.00 -0.00

34 8 -0.00 0.00 0.00 -0.00 0.00 -0.00 0.00 -0.00 -0.00

35 6 -0.00 0.00 0.00 0.00 -0.00 -0.00 0.00 0.00 0.00

36 1 -0.00 -0.00 0.00 0.00 -0.00 0.00 -0.00 -0.00 -0.00

37 1 -0.00 -0.01 0.00 -0.00 -0.00 0.00 0.00 -0.00 -0.00

38 1 -0.00 -0.01 -0.01 -0.00 -0.00 -0.00 0.00 -0.00 -0.00

39 6 0.02 -0.02 -0.01 0.00 0.01 -0.00 -0.00 -0.01 0.00

40 8 -0.02 0.02 0.02 -0.00 0.00 0.00 0.00 0.00 -0.00

41 8 -0.00 0.00 -0.01 -0.00 -0.00 0.00 0.00 0.00 -0.00

42 6 0.08 0.16 0.06 -0.01 -0.01 -0.01 0.00 0.01 0.00

43 6 -0.01 -0.25 0.02 0.00 0.02 -0.00 -0.00 -0.01 0.00

44 6 -0.10 -0.32 -0.06 0.01 0.03 0.01 -0.01 -0.01 -0.00

45 6 0.14 0.32 0.10 -0.01 -0.03 -0.01 0.01 0.01 0.00

46 1 -0.16 0.13 -0.18 0.01 -0.01 0.02 -0.01 0.01 -0.01

47 6 0.01 0.26 -0.03 -0.00 -0.02 0.00 0.00 0.01 -0.00

48 6 -0.12 -0.15 -0.10 0.01 0.01 0.01 -0.01 -0.01 -0.01

49 1 -0.17 -0.37 -0.13 0.01 0.03 0.01 -0.01 -0.02 -0.01

50 1 0.20 -0.23 0.22 -0.02 0.02 -0.02 0.01 -0.01 0.01

51 1 0.13 -0.18 0.15 -0.01 0.02 -0.01 0.01 -0.01 0.01

52 8 -0.00 0.04 -0.01 0.00 -0.00 0.00 0.00 0.00 -0.00

53 6 -0.00 -0.02 0.00 -0.00 0.00 -0.00 0.00 -0.00 0.00

54 1 0.03 -0.00 0.01 -0.00 0.00 -0.00 0.00 -0.00 0.00

55 1 0.01 0.00 0.03 -0.00 -0.00 -0.00 -0.00 -0.00 0.00

56 1 0.06 0.06 0.06 -0.01 -0.00 -0.00 0.00 0.00 0.00

136 137 138

A A A

Frequencies -- 1646.6172 1659.8882 1662.4133

Red. masses -- 6.2571 6.3297 6.5790

Frc consts -- 9.9957 10.2752 10.7124

IR Inten -- 80.1409 45.3344 36.1678

Atom AN X Y Z X Y Z X Y Z

1 16 0.00 0.00 0.01 0.01 0.00 0.00 -0.00 -0.00 -0.00

2 6 -0.09 -0.17 -0.05 -0.13 -0.27 -0.07 0.05 0.09 0.02

3 6 0.05 -0.10 -0.01 0.01 0.06 0.01 -0.01 -0.01 -0.00

4 6 -0.05 0.15 0.03 0.07 -0.04 -0.01 -0.02 0.00 0.00

5 6 -0.01 0.00 0.01 -0.01 0.01 0.01 0.00 -0.00 -0.00

6 6 0.01 0.00 -0.01 0.01 -0.00 -0.01 -0.00 0.00 0.00

7 6 0.01 0.00 -0.01 0.00 -0.01 -0.00 -0.00 0.00 0.00

8 6 -0.01 -0.00 0.01 -0.01 0.01 0.00 0.00 -0.00 -0.00

9 1 -0.02 -0.01 0.02 -0.02 -0.01 0.02 0.01 0.00 -0.01

10 6 -0.01 -0.00 0.01 -0.01 0.00 0.01 0.00 -0.00 -0.00

11 1 0.01 -0.01 -0.00 0.00 0.00 -0.01 -0.00 -0.00 0.00

12 6 0.01 -0.01 -0.01 0.01 -0.01 -0.01 -0.00 0.00 0.00

13 1 -0.01 0.01 0.00 -0.01 -0.00 0.01 0.00 0.00 -0.00

14 1 0.01 0.01 -0.02 0.01 0.01 -0.01 -0.00 -0.00 0.00

15 1 -0.01 0.02 0.00 -0.01 0.02 0.01 0.00 -0.01 -0.00

16 8 0.00 -0.00 -0.00 -0.00 -0.00 -0.00 0.00 0.00 0.00

17 8 -0.00 -0.00 -0.00 -0.00 -0.00 -0.00 0.00 0.00 0.00

18 6 0.06 0.22 0.03 0.08 0.33 0.05 -0.03 -0.12 -0.02

19 1 0.06 -0.24 0.02 0.08 -0.40 0.03 -0.02 0.11 -0.01

20 6 -0.10 -0.00 0.00 0.01 0.07 -0.01 0.00 -0.04 0.00

21 6 0.01 0.03 -0.01 0.09 -0.13 0.01 -0.04 0.07 -0.01

22 6 0.11 -0.07 -0.01 0.01 -0.10 0.00 -0.01 0.05 0.00

23 6 0.28 -0.18 -0.04 -0.14 0.05 0.01 0.04 -0.01 -0.00

24 6 -0.05 -0.04 0.01 -0.09 0.10 -0.01 0.05 -0.04 0.00

25 1 0.03 0.00 0.00 -0.06 0.18 -0.03 0.03 -0.09 0.02

26 6 -0.03 -0.12 0.01 -0.04 0.23 -0.01 0.02 -0.10 0.01

27 6 -0.24 0.28 0.04 0.09 -0.05 -0.01 -0.02 -0.00 0.00

28 1 -0.37 -0.14 0.02 0.20 0.02 -0.02 -0.06 0.00 0.01

29 6 0.07 0.15 -0.02 -0.03 -0.20 0.02 0.01 0.08 -0.01

30 1 -0.02 -0.05 0.01 0.21 0.07 -0.04 -0.09 -0.03 0.02

31 1 -0.12 0.04 0.00 0.19 -0.22 -0.01 -0.08 0.10 0.00

32 1 0.05 -0.45 -0.04 0.05 0.07 0.00 -0.03 0.00 0.00

33 1 -0.15 -0.14 0.03 0.23 0.11 -0.03 -0.10 -0.05 0.01

34 8 0.00 -0.03 -0.01 -0.01 0.01 0.00 0.00 -0.00 -0.00

35 6 0.00 -0.02 -0.00 0.00 0.01 0.00 -0.00 -0.00 -0.00

36 1 0.02 0.02 -0.00 -0.01 -0.02 -0.00 0.00 0.01 0.00

37 1 0.02 0.09 -0.03 -0.00 -0.05 0.01 0.00 0.01 -0.00

38 1 0.02 0.07 0.07 -0.01 -0.04 -0.03 0.00 0.01 0.01

39 6 -0.02 -0.03 0.02 -0.04 -0.03 0.03 0.01 0.04 -0.02

40 8 0.01 -0.01 -0.02 0.02 -0.02 -0.03 -0.01 0.00 0.01

41 8 0.00 -0.00 -0.00 -0.00 -0.01 -0.00 -0.01 -0.00 -0.01

42 6 0.02 0.04 0.02 0.09 0.00 0.10 0.21 -0.09 0.24

43 6 -0.00 -0.04 0.00 -0.05 0.03 -0.06 -0.15 0.19 -0.18

44 6 -0.03 -0.06 -0.02 -0.07 -0.02 -0.07 -0.14 0.07 -0.15

45 6 0.03 0.06 0.02 0.06 0.03 0.05 0.11 -0.01 0.11

46 1 -0.03 0.02 -0.03 -0.02 -0.10 0.01 0.03 -0.34 0.09

47 6 0.01 0.04 0.00 0.05 -0.03 0.06 0.14 -0.17 0.16

48 6 -0.03 -0.03 -0.02 -0.09 -0.00 -0.09 -0.21 0.04 -0.22

49 1 -0.03 -0.07 -0.02 -0.00 -0.10 0.01 0.05 -0.17 0.08

50 1 0.03 -0.03 0.04 0.01 0.10 -0.00 -0.03 0.31 -0.07

51 1 0.03 -0.03 0.03 0.11 -0.01 0.11 0.25 0.04 0.25

52 8 0.00 0.01 -0.00 0.01 0.00 0.01 0.02 -0.01 0.02

53 6 0.00 -0.00 0.00 0.01 0.00 0.01 0.02 0.01 0.02

54 1 -0.00 -0.00 -0.00 -0.05 -0.02 -0.01 -0.12 -0.03 -0.04

55 1 -0.00 -0.00 -0.00 -0.01 -0.02 -0.05 -0.03 -0.04 -0.11

56 1 0.01 0.01 0.01 -0.01 -0.02 -0.00 -0.03 -0.06 -0.03

139 140 141

A A A

Frequencies -- 1674.9609 1831.4868 3028.9875

Red. masses -- 6.5233 13.1194 1.0342

Frc consts -- 10.7828 25.9281 5.5904

IR Inten -- 119.0253 220.3005 64.3105

Atom AN X Y Z X Y Z X Y Z

1 16 0.00 0.00 0.00 -0.00 -0.00 0.00 -0.00 0.00 -0.00

2 6 -0.10 -0.21 -0.05 0.01 0.03 0.01 0.00 0.00 0.00

3 6 -0.00 0.12 0.02 0.01 0.00 0.00 -0.00 -0.00 -0.00

4 6 0.01 -0.07 -0.01 -0.00 -0.01 -0.00 -0.00 0.00 0.00

5 6 -0.00 0.00 0.00 -0.00 -0.00 0.00 0.00 -0.00 -0.00

6 6 0.00 -0.00 -0.00 0.00 0.00 -0.00 -0.00 -0.00 0.00

7 6 0.00 -0.00 -0.00 0.00 0.00 -0.00 -0.00 -0.00 0.00

8 6 -0.00 0.00 0.00 -0.00 -0.00 0.00 -0.00 0.00 0.00

9 1 -0.01 -0.00 0.01 -0.00 0.00 0.00 0.00 -0.00 -0.00

10 6 -0.00 0.00 0.00 -0.00 -0.00 0.00 0.00 -0.00 -0.00

11 1 0.00 0.00 -0.00 0.00 -0.01 0.00 -0.00 0.00 0.00

12 6 0.00 -0.00 -0.00 0.00 0.00 -0.00 -0.00 -0.00 0.00

13 1 -0.00 -0.00 0.00 0.00 0.00 -0.00 0.00 -0.00 -0.00

14 1 0.00 0.00 -0.00 0.00 -0.00 -0.00 -0.00 0.00 0.00

15 1 -0.00 0.01 0.00 0.00 0.00 -0.00 0.00 0.00 -0.00

16 8 0.00 -0.00 -0.00 -0.00 0.00 0.00 0.00 -0.00 -0.00

17 8 0.00 -0.00 0.00 -0.00 0.00 -0.00 0.00 -0.00 0.00

18 6 0.06 0.25 0.03 0.02 -0.11 -0.02 -0.00 -0.00 -0.00

19 1 0.06 -0.28 0.02 -0.03 0.01 0.03 0.00 0.00 0.00

20 6 0.08 -0.17 0.02 -0.01 0.01 -0.00 -0.00 -0.00 0.00

21 6 -0.15 0.22 -0.02 -0.00 -0.01 0.00 0.00 0.00 -0.00

22 6 -0.12 0.20 0.00 0.00 -0.00 0.00 -0.00 -0.00 -0.00

23 6 -0.10 0.07 0.02 0.00 -0.00 -0.00 0.00 -0.00 -0.00

24 6 0.17 -0.12 0.00 -0.00 0.00 -0.00 0.00 -0.00 0.00

25 1 0.10 -0.29 0.06 -0.01 0.04 0.01 -0.00 -0.00 0.00

26 6 0.09 -0.24 0.01 -0.00 0.00 0.00 -0.00 -0.00 0.00

27 6 0.10 -0.16 -0.02 -0.00 0.00 0.00 -0.00 0.00 0.00

28 1 0.10 0.06 -0.00 0.00 -0.00 -0.00 -0.00 -0.00 0.00

29 6 -0.02 0.17 -0.01 0.00 -0.00 0.00 -0.00 0.00 0.00

30 1 -0.25 -0.08 0.05 0.00 0.00 0.00 0.00 0.00 -0.00

31 1 -0.17 0.28 0.01 0.00 -0.00 0.00 0.00 0.00 -0.00

32 1 -0.07 0.25 0.02 0.00 -0.00 -0.00 0.00 0.00 -0.00

33 1 -0.21 -0.06 0.02 0.00 0.00 0.00 0.00 -0.00 -0.00

34 8 0.01 0.00 0.00 -0.00 0.00 0.00 0.00 -0.00 -0.00

35 6 -0.00 0.01 0.00 -0.00 -0.00 -0.00 -0.00 -0.00 -0.00

36 1 -0.01 -0.00 0.00 0.00 0.00 -0.00 -0.00 0.00 0.00

37 1 -0.01 -0.04 0.02 -0.00 0.00 -0.00 0.00 0.00 0.00

38 1 -0.01 -0.04 -0.03 0.00 0.00 0.00 0.00 0.00 -0.00

39 6 -0.03 -0.03 0.03 -0.37 0.46 0.57 0.00 0.00 -0.00

40 8 0.02 -0.01 -0.02 0.24 -0.27 -0.40 -0.00 -0.00 0.00

41 8 0.00 -0.00 -0.00 0.02 -0.02 -0.03 -0.00 -0.00 0.00

42 6 -0.01 0.03 -0.01 -0.01 0.04 0.02 -0.00 0.00 -0.00

43 6 0.01 -0.03 0.02 0.00 -0.03 0.00 -0.00 -0.00 -0.00

44 6 0.00 -0.02 0.01 0.00 -0.03 0.01 0.00 -0.00 0.00

45 6 -0.00 0.02 -0.00 0.00 0.01 0.00 -0.00 -0.00 -0.00

46 1 -0.01 0.04 -0.02 -0.03 0.03 -0.02 0.00 0.00 0.00

47 6 -0.01 0.02 -0.01 -0.00 0.01 -0.01 0.00 0.00 0.00

48 6 0.01 -0.01 0.01 -0.00 -0.01 0.00 0.00 0.00 0.00

49 1 -0.01 -0.00 -0.01 -0.01 -0.02 -0.01 -0.00 0.00 -0.00

50 1 0.01 -0.03 0.01 0.01 -0.02 0.01 -0.01 -0.01 -0.01

51 1 -0.01 -0.01 -0.01 -0.00 -0.01 -0.00 -0.00 -0.00 -0.00

52 8 -0.00 0.00 -0.00 -0.00 0.01 -0.01 -0.00 -0.00 -0.00

53 6 -0.00 -0.00 -0.00 0.00 -0.00 -0.00 -0.02 -0.04 -0.02

54 1 0.00 -0.00 0.00 -0.00 -0.01 0.00 -0.32 0.39 0.41

55 1 0.00 -0.00 0.00 0.00 -0.00 0.00 0.48 0.34 -0.35

56 1 0.00 0.01 0.00 0.00 0.00 0.01 0.14 -0.24 0.17

142 143 144

A A A

Frequencies -- 3038.5775 3097.7895 3101.4561

Red. masses -- 1.0336 1.1072 1.0817

Frc consts -- 5.6227 6.2601 6.1302

IR Inten -- 70.8214 35.3401 109.4124

Atom AN X Y Z X Y Z X Y Z

1 16 0.00 0.00 0.00 0.00 0.00 0.00 0.00 0.00 0.00

2 6 0.00 0.00 0.00 0.00 0.00 -0.00 -0.00 0.00 -0.00

3 6 0.00 -0.00 -0.00 0.00 0.00 0.00 0.00 0.00 0.00

4 6 -0.00 0.00 0.00 -0.00 -0.00 0.00 0.00 -0.00 -0.00

5 6 0.00 -0.00 -0.00 -0.00 -0.00 -0.00 -0.00 -0.00 -0.00

6 6 -0.00 0.00 0.00 -0.00 0.00 -0.00 -0.00 0.00 0.00

7 6 -0.00 0.00 0.00 0.00 -0.00 -0.00 0.00 -0.00 -0.00

8 6 0.00 0.00 -0.00 0.00 -0.00 -0.00 -0.00 0.00 0.00

9 1 0.00 -0.00 0.00 0.00 -0.00 0.00 0.00 -0.00 -0.00

10 6 -0.00 0.00 0.00 0.00 -0.00 -0.00 0.00 -0.00 0.00

11 1 0.00 -0.00 -0.00 -0.00 0.00 0.00 -0.00 0.00 0.00

12 6 0.00 -0.00 -0.00 -0.00 -0.00 0.00 -0.00 -0.00 0.00

13 1 -0.00 0.00 0.00 -0.00 0.00 0.00 0.00 -0.00 -0.00

14 1 0.00 -0.00 -0.00 -0.00 0.00 0.00 -0.00 0.00 0.00

15 1 0.00 0.00 -0.00 0.00 0.00 -0.00 0.00 0.00 -0.00

16 8 -0.00 -0.00 -0.00 0.00 -0.00 -0.00 -0.00 -0.00 -0.00

17 8 -0.00 0.00 -0.00 -0.00 -0.00 -0.00 -0.00 -0.00 -0.00

18 6 0.00 -0.00 0.00 -0.00 -0.00 -0.00 -0.07 -0.00 -0.04

19 1 -0.00 -0.00 -0.00 0.02 -0.00 0.01 0.89 -0.03 0.44

20 6 -0.00 -0.00 0.00 -0.00 0.00 -0.00 -0.00 0.00 -0.00

21 6 0.00 0.00 0.00 -0.00 -0.00 -0.00 0.00 -0.00 -0.00

22 6 0.00 -0.00 -0.00 0.00 0.00 0.00 0.00 0.00 0.00

23 6 0.00 0.00 0.00 0.00 -0.00 0.00 0.00 0.00 0.00

24 6 0.00 -0.00 0.00 -0.00 -0.00 0.00 -0.00 -0.00 0.00

25 1 0.00 0.00 -0.00 0.00 0.00 0.00 -0.00 -0.00 0.00

26 6 0.00 0.00 -0.00 -0.00 0.00 0.00 0.00 -0.00 -0.00

27 6 0.00 0.00 0.00 -0.00 0.00 0.00 0.00 -0.00 -0.00

28 1 -0.00 -0.02 -0.00 -0.00 -0.00 0.00 -0.00 -0.00 -0.00

29 6 -0.00 -0.00 0.00 0.00 -0.00 -0.00 0.00 -0.00 -0.00

30 1 0.00 0.00 -0.00 0.00 0.00 0.00 0.00 0.00 -0.00

31 1 -0.00 -0.00 0.00 0.00 0.00 0.00 -0.00 -0.00 0.00

32 1 0.00 -0.00 0.00 0.00 0.00 0.00 0.00 0.00 -0.00

33 1 0.00 -0.00 0.00 -0.00 0.00 0.00 -0.00 0.00 -0.00

34 8 0.00 -0.00 -0.00 0.00 -0.00 -0.00 -0.00 0.00 0.00

35 6 -0.03 -0.04 -0.01 -0.00 0.00 -0.00 -0.00 0.00 -0.00

36 1 -0.31 0.11 0.06 -0.00 0.00 -0.00 -0.00 0.00 -0.00

37 1 0.37 0.06 0.56 0.00 0.00 0.00 0.01 0.00 0.01

38 1 0.26 0.34 -0.50 -0.00 -0.00 0.00 -0.00 -0.00 0.00

39 6 -0.00 0.00 -0.00 -0.00 0.00 0.00 -0.00 0.00 -0.00

40 8 0.00 -0.00 -0.00 0.00 -0.00 -0.00 0.00 -0.00 -0.00

41 8 -0.00 -0.00 0.00 0.00 0.00 -0.00 -0.00 -0.00 0.00

42 6 0.00 0.00 0.00 -0.00 0.00 0.00 -0.00 0.00 -0.00

43 6 -0.00 -0.00 -0.00 -0.00 0.00 0.00 -0.00 -0.00 -0.00

44 6 -0.00 -0.00 -0.00 0.00 -0.00 -0.00 0.00 -0.00 0.00

45 6 0.00 -0.00 0.00 -0.00 0.00 0.00 0.00 -0.00 0.00

46 1 0.00 0.00 -0.00 -0.00 -0.00 -0.00 0.00 0.00 0.00

47 6 -0.00 -0.00 -0.00 0.00 0.00 -0.00 -0.00 -0.00 -0.00

48 6 0.00 -0.00 0.00 -0.00 -0.00 0.00 0.00 -0.00 0.00

49 1 -0.00 0.00 -0.00 0.00 -0.00 -0.00 -0.00 0.00 -0.00

50 1 0.00 0.00 0.00 0.00 -0.00 -0.00 0.00 0.00 0.00

51 1 0.00 0.00 -0.00 0.00 0.00 -0.00 0.00 0.00 -0.00

52 8 0.00 -0.00 0.00 0.00 -0.00 -0.00 -0.00 0.00 -0.00

53 6 0.00 0.00 0.00 -0.07 0.01 0.07 0.00 0.00 -0.00

54 1 0.00 -0.00 -0.00 0.35 -0.45 -0.44 -0.01 0.01 0.01

55 1 -0.00 -0.00 0.00 0.47 0.36 -0.35 -0.01 -0.01 0.01

56 1 -0.00 0.00 -0.00 -0.03 0.02 0.00 0.00 -0.00 0.00

145 146 147

A A A

Frequencies -- 3111.4128 3159.6054 3168.5006

Red. masses -- 1.1075 1.1013 1.1016

Frc consts -- 6.3167 6.4775 6.5159

IR Inten -- 34.5244 18.1181 11.2600

Atom AN X Y Z X Y Z X Y Z

1 16 0.00 0.00 -0.00 0.00 0.00 0.00 -0.00 0.00 0.00

2 6 -0.00 -0.00 0.00 -0.00 0.00 -0.00 -0.00 0.00 -0.00

3 6 -0.00 -0.00 -0.00 -0.00 0.00 0.00 0.00 0.00 0.00

4 6 0.00 -0.00 0.00 0.00 -0.00 -0.00 0.00 -0.00 -0.00

5 6 -0.00 0.00 0.00 -0.00 -0.00 -0.00 0.00 0.00 -0.00

6 6 0.00 -0.00 -0.00 -0.00 -0.00 -0.00 0.00 -0.00 0.00

7 6 0.00 -0.00 -0.00 0.00 -0.00 -0.00 0.00 -0.00 -0.00

8 6 0.00 -0.00 -0.00 -0.00 0.00 0.00 0.00 -0.00 -0.00

9 1 -0.00 0.00 -0.00 0.00 0.00 0.00 -0.00 0.00 -0.00

10 6 -0.00 -0.00 -0.00 0.00 -0.00 -0.00 -0.00 0.00 0.00

11 1 -0.00 0.00 0.00 -0.00 0.00 0.00 -0.01 0.01 0.01

12 6 -0.00 0.00 -0.00 -0.00 -0.00 0.00 0.00 0.00 -0.00

13 1 -0.00 0.00 0.00 0.00 -0.00 -0.00 -0.00 0.00 0.00

14 1 0.00 0.00 0.00 -0.00 0.00 0.00 0.00 -0.00 -0.00

15 1 -0.00 -0.00 0.00 0.00 0.00 -0.00 0.00 0.00 -0.00

16 8 -0.00 -0.00 0.00 -0.00 -0.00 -0.00 0.00 -0.00 -0.00

17 8 0.00 -0.00 0.00 -0.00 -0.00 -0.00 0.00 -0.00 -0.00

18 6 0.00 0.00 0.00 -0.00 0.00 -0.00 0.00 -0.00 0.00

19 1 -0.01 0.00 -0.01 0.00 -0.00 0.00 -0.00 0.00 -0.00

20 6 0.00 0.00 -0.00 0.00 -0.00 0.00 0.00 0.00 0.00

21 6 -0.00 -0.00 0.00 -0.00 -0.00 0.00 0.00 0.00 -0.00

22 6 -0.00 -0.00 -0.00 -0.00 0.00 -0.00 -0.00 0.00 -0.00

23 6 0.00 0.00 0.00 -0.00 0.00 0.00 0.00 0.00 0.00

24 6 0.00 0.00 0.00 0.00 0.00 -0.00 0.00 0.00 -0.00

25 1 0.00 0.00 -0.00 0.00 0.00 -0.00 -0.00 -0.00 0.00

26 6 -0.00 0.00 0.00 0.00 0.00 -0.00 0.00 0.00 -0.00

27 6 -0.00 -0.00 -0.00 0.00 0.00 -0.00 -0.00 -0.00 0.00

28 1 -0.00 -0.01 0.00 -0.00 -0.00 0.00 -0.00 -0.04 -0.00

29 6 -0.00 0.00 -0.00 0.00 -0.00 0.00 -0.00 0.00 0.00

30 1 -0.00 -0.00 -0.00 -0.00 -0.00 0.00 -0.00 -0.00 0.00

31 1 0.00 0.00 -0.00 -0.00 -0.00 0.00 -0.00 -0.00 0.00

32 1 0.00 0.00 0.00 -0.00 -0.00 0.00 0.01 0.01 -0.00

33 1 0.00 -0.00 -0.00 -0.00 0.00 0.00 0.00 -0.00 0.00

34 8 -0.00 0.00 0.00 -0.00 0.00 -0.00 0.00 0.00 0.00

35 6 -0.01 0.03 -0.09 -0.00 0.00 0.00 -0.09 0.01 0.01

36 1 0.02 -0.00 -0.02 0.00 -0.00 -0.00 0.88 -0.30 -0.17

37 1 0.40 0.06 0.57 -0.00 0.00 0.00 0.10 0.02 0.18

38 1 -0.30 -0.37 0.53 0.00 0.00 -0.00 0.08 0.12 -0.18

39 6 0.00 -0.00 -0.00 -0.00 -0.00 0.00 -0.00 -0.00 0.00

40 8 -0.00 0.00 0.00 0.00 0.00 -0.00 0.00 0.00 -0.00

41 8 -0.00 -0.00 0.00 0.00 0.00 -0.00 0.00 0.00 -0.00

42 6 0.00 -0.00 0.00 0.00 0.00 0.00 -0.00 -0.00 0.00

43 6 0.00 0.00 0.00 0.00 0.00 0.00 0.00 0.00 0.00

44 6 -0.00 0.00 -0.00 -0.00 -0.00 -0.00 -0.00 0.00 -0.00

45 6 0.00 -0.00 0.00 0.00 -0.00 0.00 -0.00 0.00 -0.00

46 1 -0.00 -0.00 -0.00 -0.00 -0.00 -0.00 -0.00 -0.00 -0.00

47 6 0.00 0.00 0.00 0.00 0.00 0.00 0.00 0.00 0.00

48 6 0.00 0.00 -0.00 -0.00 -0.00 -0.00 -0.00 0.00 -0.00

49 1 -0.00 -0.00 -0.00 -0.00 0.00 -0.00 0.00 -0.00 0.00

50 1 -0.00 -0.00 -0.00 -0.02 -0.02 -0.02 -0.00 -0.00 -0.00

51 1 0.00 -0.00 0.00 0.00 0.01 -0.00 -0.00 -0.00 0.00

52 8 0.00 -0.00 0.00 0.00 0.00 0.00 0.00 -0.00 0.00

53 6 0.00 -0.00 -0.00 0.03 -0.08 0.04 -0.00 0.00 -0.00

54 1 -0.00 0.00 0.00 -0.11 0.13 0.15 0.00 -0.00 -0.00

55 1 -0.00 -0.00 0.00 0.15 0.09 -0.11 0.00 0.00 -0.00

56 1 -0.00 0.00 -0.00 -0.41 0.70 -0.49 0.00 -0.00 0.00

148 149 150

A A A

Frequencies -- 3181.5307 3190.2603 3191.1378

Red. masses -- 1.0873 1.0862 1.0867

Frc consts -- 6.4846 6.5133 6.5201

IR Inten -- 2.9322 0.7296 2.7862

Atom AN X Y Z X Y Z X Y Z

1 16 0.00 -0.00 0.00 0.00 0.00 -0.00 0.00 0.00 0.00

2 6 0.00 0.00 0.00 -0.00 0.00 0.00 -0.00 0.00 -0.00

3 6 -0.00 -0.00 0.00 0.00 -0.00 0.00 0.00 0.00 -0.00

4 6 0.00 -0.00 -0.00 -0.00 0.00 -0.00 0.00 0.00 0.00

5 6 -0.00 0.00 -0.00 -0.00 -0.00 0.00 -0.00 0.00 -0.00

6 6 -0.00 0.00 0.00 0.00 -0.01 -0.00 0.00 -0.00 0.00

7 6 0.00 -0.00 -0.00 -0.01 0.00 0.01 -0.00 0.00 0.00

8 6 0.00 -0.00 -0.00 -0.02 0.01 0.02 0.00 -0.00 -0.00

9 1 0.00 -0.00 0.00 -0.04 0.14 0.00 -0.00 0.00 0.00

10 6 -0.00 0.00 0.00 0.01 -0.05 0.00 0.00 -0.00 0.00

11 1 -0.00 0.00 0.00 0.09 -0.04 -0.09 0.00 -0.00 -0.00

12 6 0.00 0.00 -0.00 0.02 0.04 -0.04 0.00 0.00 -0.00

13 1 -0.00 0.00 0.00 0.29 -0.13 -0.28 -0.00 0.00 0.00

14 1 0.00 -0.00 -0.00 -0.16 0.54 0.00 -0.00 0.00 0.00

15 1 -0.00 -0.00 0.00 -0.27 -0.44 0.44 -0.00 -0.00 0.00

16 8 -0.00 0.00 0.00 -0.00 -0.00 0.00 -0.00 -0.00 -0.00

17 8 -0.00 0.00 -0.00 -0.00 -0.00 0.00 -0.00 -0.00 -0.00

18 6 -0.00 -0.00 -0.00 0.00 -0.00 0.00 -0.00 0.00 -0.00

19 1 0.00 -0.00 0.00 -0.00 0.00 -0.00 0.00 0.00 0.00

20 6 0.00 0.00 -0.00 -0.00 0.00 0.00 0.00 -0.00 0.00

21 6 -0.00 -0.00 0.00 0.00 -0.00 0.00 -0.00 -0.00 0.00

22 6 0.00 -0.00 -0.00 0.00 -0.00 0.00 -0.00 0.00 0.00

23 6 -0.00 -0.00 -0.00 -0.00 0.00 -0.00 0.00 0.00 0.00

24 6 0.00 0.01 -0.00 -0.00 -0.00 0.00 0.00 0.00 -0.00

25 1 0.01 0.01 -0.00 -0.00 0.00 -0.00 0.00 0.00 -0.00

26 6 -0.07 -0.03 0.00 -0.00 -0.00 0.00 0.00 0.00 -0.00

27 6 0.01 0.00 -0.00 0.00 0.00 -0.00 -0.00 -0.00 0.00

28 1 0.00 0.02 0.00 0.00 -0.00 -0.00 -0.00 -0.01 -0.00

29 6 0.03 -0.02 -0.00 0.00 -0.00 0.00 0.00 -0.00 0.00

30 1 -0.02 -0.18 0.03 0.00 0.00 -0.00 -0.00 -0.03 0.00

31 1 0.78 0.37 -0.05 0.00 0.00 -0.00 -0.01 -0.00 0.00

32 1 -0.13 -0.05 0.00 -0.00 -0.00 0.00 0.03 0.01 -0.00

33 1 -0.35 0.25 0.00 -0.00 0.00 0.00 -0.01 0.01 -0.00

34 8 -0.00 0.00 0.00 0.00 0.00 0.00 -0.00 0.00 0.00

35 6 -0.00 -0.00 0.00 -0.00 0.00 0.00 0.00 -0.00 -0.00

36 1 0.00 -0.00 -0.00 0.01 -0.00 -0.00 -0.00 0.00 0.00

37 1 0.00 0.00 0.00 0.00 0.00 0.00 -0.00 -0.00 -0.00

38 1 0.00 0.00 -0.00 0.00 0.00 -0.00 -0.00 -0.00 0.00

39 6 -0.00 0.00 -0.00 -0.00 0.00 0.00 0.00 -0.00 0.00

40 8 0.00 -0.00 -0.00 0.00 -0.00 -0.00 0.00 -0.00 -0.00

41 8 -0.00 -0.00 0.00 -0.00 -0.00 0.00 -0.00 0.00 -0.00

42 6 -0.00 0.00 -0.00 -0.00 0.00 0.00 -0.00 0.00 -0.00

43 6 0.00 0.00 0.00 -0.00 -0.00 -0.00 0.01 0.01 0.01

44 6 -0.00 -0.00 -0.00 -0.00 -0.00 0.00 -0.00 0.00 -0.00

45 6 -0.00 0.00 -0.00 0.00 -0.00 0.00 -0.02 0.02 -0.03

46 1 -0.00 -0.00 -0.00 0.00 0.00 0.00 -0.13 -0.11 -0.12

47 6 0.00 0.00 0.00 -0.00 -0.00 -0.00 0.01 0.01 0.01

48 6 -0.00 -0.00 0.00 -0.00 -0.00 0.00 -0.00 -0.07 0.00

49 1 0.00 -0.00 0.00 -0.00 0.00 -0.00 0.29 -0.25 0.33

50 1 -0.00 -0.00 -0.00 0.00 0.00 0.00 -0.11 -0.09 -0.10

51 1 0.00 0.00 -0.00 0.00 0.00 -0.00 0.05 0.81 -0.05

52 8 0.00 -0.00 0.00 -0.00 -0.00 -0.00 0.00 -0.00 0.00

53 6 -0.00 0.00 -0.00 -0.00 0.00 0.00 -0.00 0.00 -0.00

54 1 0.00 -0.00 -0.00 0.00 -0.00 -0.00 0.00 -0.00 -0.00

55 1 -0.00 -0.00 0.00 -0.00 -0.00 0.00 -0.00 -0.00 0.00

56 1 0.00 -0.00 0.00 -0.00 0.00 -0.00 0.00 -0.01 0.01

151 152 153

A A A

Frequencies -- 3191.6988 3194.4461 3200.2112

Red. masses -- 1.0902 1.0904 1.0895

Frc consts -- 6.5435 6.5559 6.5740

IR Inten -- 2.9537 23.5086 10.4040

Atom AN X Y Z X Y Z X Y Z

1 16 0.00 0.00 0.00 0.00 -0.00 0.00 -0.00 0.00 0.00

2 6 0.00 0.00 -0.00 -0.00 0.00 -0.00 -0.00 0.00 -0.00

3 6 0.00 -0.00 -0.00 0.00 -0.00 -0.00 -0.00 -0.00 -0.00

4 6 0.00 0.00 0.00 0.00 0.00 0.00 -0.00 0.00 0.00

5 6 -0.00 0.00 -0.00 -0.00 0.00 -0.00 0.00 -0.00 -0.00

6 6 -0.00 0.00 0.00 -0.00 0.00 0.00 -0.01 0.03 0.00

7 6 -0.00 0.00 0.00 -0.00 0.00 0.00 -0.01 0.01 0.01

8 6 0.00 -0.00 -0.00 0.00 -0.00 -0.00 0.04 -0.02 -0.04

9 1 0.00 -0.00 0.00 0.00 -0.00 -0.00 0.09 -0.31 -0.01

10 6 0.00 -0.00 0.00 0.00 -0.00 0.00 0.02 -0.05 -0.00

11 1 0.00 -0.00 -0.00 0.00 -0.00 -0.00 0.13 -0.06 -0.12

12 6 0.00 0.00 -0.00 0.00 0.00 -0.00 -0.01 -0.00 0.01

13 1 -0.00 0.00 0.00 -0.00 0.00 0.00 -0.45 0.21 0.44

14 1 -0.00 0.00 0.00 -0.00 0.00 -0.00 -0.18 0.61 0.00

15 1 -0.00 -0.00 0.00 -0.00 -0.00 0.00 0.04 0.05 -0.06

16 8 -0.00 -0.00 -0.00 -0.00 0.00 -0.00 -0.00 -0.00 0.00

17 8 -0.00 -0.00 -0.00 -0.00 0.00 -0.00 0.00 -0.00 -0.00

18 6 0.00 -0.00 0.00 0.00 0.00 0.00 0.00 -0.00 -0.00

19 1 -0.00 0.00 -0.00 0.00 -0.00 0.00 -0.00 0.00 -0.00

20 6 0.00 -0.00 0.00 0.00 0.00 -0.00 0.00 -0.00 0.00

21 6 -0.00 -0.00 0.00 0.00 0.00 -0.00 -0.00 0.00 0.00

22 6 -0.00 0.00 0.00 0.00 -0.00 0.00 -0.00 -0.00 -0.00

23 6 0.00 0.01 0.00 0.00 0.00 0.00 -0.00 -0.00 -0.00

24 6 0.00 0.02 -0.00 -0.01 -0.07 0.01 0.00 0.00 -0.00

25 1 0.01 0.01 -0.00 -0.04 -0.02 0.01 0.00 0.00 -0.00

26 6 -0.00 0.00 0.00 -0.03 -0.02 0.00 0.00 0.00 -0.00

27 6 -0.08 -0.03 0.00 -0.02 -0.01 0.00 0.00 0.00 -0.00

28 1 -0.02 -0.16 -0.02 -0.01 -0.04 -0.01 0.00 0.00 0.00

29 6 0.01 -0.01 0.00 -0.02 0.02 -0.00 -0.00 0.00 0.00

30 1 -0.02 -0.21 0.04 0.08 0.78 -0.14 -0.00 -0.00 0.00

31 1 0.02 0.01 -0.00 0.35 0.17 -0.02 -0.00 -0.00 0.00

32 1 0.86 0.37 -0.03 0.23 0.10 -0.01 -0.00 -0.00 0.00

33 1 -0.15 0.11 0.00 0.30 -0.22 0.00 0.00 -0.00 -0.00

34 8 -0.00 0.00 0.00 -0.00 0.00 0.00 0.00 0.00 0.00

35 6 0.00 -0.00 -0.00 0.00 -0.00 -0.00 -0.00 0.00 0.00

36 1 -0.02 0.01 0.00 -0.00 0.00 0.00 0.01 -0.00 -0.00

37 1 -0.00 -0.00 -0.01 -0.00 -0.00 -0.00 0.00 0.00 0.00

38 1 -0.00 -0.00 0.01 -0.00 -0.00 0.00 0.00 0.00 -0.00

39 6 0.00 0.00 -0.00 0.00 -0.00 0.00 0.00 -0.00 -0.00

40 8 -0.00 0.00 -0.00 0.00 0.00 0.00 -0.00 0.00 0.00

41 8 -0.00 -0.00 0.00 0.00 0.00 -0.00 -0.00 0.00 0.00

42 6 -0.00 0.00 -0.00 0.00 -0.00 0.00 0.00 0.00 -0.00

43 6 -0.00 -0.00 -0.00 -0.00 -0.00 -0.00 -0.00 -0.00 -0.00

44 6 -0.00 -0.00 -0.00 0.00 0.00 0.00 -0.00 0.00 -0.00

45 6 0.00 -0.00 0.00 -0.00 0.00 -0.00 0.00 -0.00 0.00

46 1 0.00 0.00 0.00 0.02 0.01 0.02 0.00 0.00 0.00

47 6 -0.00 -0.00 -0.00 0.00 0.00 0.00 0.00 0.00 0.00

48 6 0.00 0.00 -0.00 -0.00 -0.00 0.00 -0.00 -0.00 -0.00

49 1 -0.01 0.01 -0.01 0.00 -0.00 0.00 -0.00 0.00 -0.00

50 1 0.00 0.00 0.00 -0.00 -0.00 -0.00 -0.00 -0.00 -0.00

51 1 -0.00 -0.03 0.00 0.00 0.03 -0.00 0.00 0.00 -0.00

52 8 -0.00 0.00 -0.00 -0.00 0.00 -0.00 -0.00 -0.00 0.00

53 6 0.00 -0.00 0.00 0.00 0.00 0.00 0.00 0.00 -0.00

54 1 -0.00 0.00 0.00 0.00 -0.00 -0.00 -0.00 0.00 0.00

55 1 0.00 0.00 -0.00 -0.00 -0.00 0.00 -0.00 -0.00 0.00

56 1 -0.00 0.00 -0.00 -0.00 -0.00 -0.00 0.00 -0.00 0.00

154 155 156

A A A

Frequencies -- 3203.5850 3208.7465 3209.4533

Red. masses -- 1.0909 1.0934 1.0972

Frc consts -- 6.5962 6.6329 6.6585

IR Inten -- 17.8069 14.4786 25.4454

Atom AN X Y Z X Y Z X Y Z

1 16 -0.00 0.00 -0.00 -0.00 -0.00 -0.00 0.00 -0.00 0.00

2 6 0.00 0.00 0.00 0.00 -0.00 0.00 0.00 0.00 0.00

3 6 -0.00 -0.00 -0.00 0.00 -0.00 0.00 -0.00 0.00 -0.00

4 6 -0.00 0.00 0.00 -0.00 -0.00 -0.00 0.00 -0.00 -0.00

5 6 0.00 -0.00 0.00 -0.00 -0.00 0.00 -0.00 -0.00 0.00

6 6 0.00 -0.00 -0.00 0.01 -0.04 -0.00 0.00 -0.00 -0.00

7 6 0.00 0.00 -0.00 -0.01 0.01 0.01 -0.00 0.00 0.00

8 6 -0.00 0.00 0.00 -0.02 0.01 0.01 -0.00 0.00 0.00

9 1 -0.00 0.00 0.00 -0.14 0.51 0.01 -0.01 0.03 0.00

10 6 0.00 -0.00 -0.00 0.01 -0.03 -0.00 0.00 -0.00 -0.00

11 1 -0.00 0.00 0.00 0.16 -0.08 -0.16 0.01 -0.00 -0.01

12 6 -0.00 -0.00 0.00 -0.02 -0.04 0.04 -0.00 -0.00 0.00

13 1 0.00 -0.00 -0.00 0.18 -0.09 -0.18 0.01 -0.00 -0.01

14 1 -0.00 0.00 0.00 -0.11 0.37 0.00 -0.01 0.02 0.00

15 1 0.00 0.00 -0.00 0.26 0.42 -0.42 0.01 0.02 -0.02

16 8 0.00 -0.00 0.00 0.00 -0.00 -0.00 -0.00 0.00 0.00

17 8 0.00 -0.00 0.00 -0.00 0.00 0.00 -0.00 0.00 -0.00

18 6 0.00 -0.00 0.00 0.00 -0.00 0.00 -0.00 -0.00 -0.00

19 1 -0.00 0.00 -0.00 0.00 0.00 -0.00 0.00 -0.00 0.00

20 6 -0.00 -0.00 0.00 -0.00 0.00 -0.00 0.00 -0.00 0.00

21 6 -0.00 -0.00 0.00 0.00 0.00 -0.00 -0.00 -0.00 0.00

22 6 -0.00 0.00 0.00 -0.00 0.00 -0.00 0.00 -0.00 0.00

23 6 -0.00 -0.00 -0.00 0.00 0.00 0.00 0.00 0.00 0.00

24 6 0.00 0.00 -0.00 -0.00 -0.00 0.00 0.01 0.05 -0.01

25 1 0.00 0.00 -0.00 -0.00 -0.00 0.00 0.04 0.02 -0.01

26 6 0.00 0.00 -0.00 0.00 0.00 -0.00 -0.02 -0.01 0.00

27 6 0.00 0.00 -0.00 0.00 0.00 -0.00 -0.00 -0.00 0.00

28 1 0.00 0.00 0.00 -0.00 -0.00 -0.00 -0.00 -0.01 -0.00

29 6 0.00 -0.00 0.00 0.00 -0.00 -0.00 -0.06 0.04 0.00

30 1 -0.00 -0.02 0.00 0.00 0.02 -0.00 -0.06 -0.51 0.09

31 1 -0.02 -0.01 0.00 -0.01 -0.01 0.00 0.26 0.13 -0.02

32 1 -0.00 -0.00 0.00 -0.00 -0.00 0.00 0.03 0.01 -0.00

33 1 -0.03 0.02 -0.00 -0.03 0.02 -0.00 0.64 -0.47 0.00

34 8 0.00 -0.00 -0.00 0.00 0.00 0.00 -0.00 0.00 0.00

35 6 -0.00 0.00 0.00 -0.00 0.00 -0.00 0.00 -0.00 -0.00

36 1 0.00 -0.00 -0.00 0.01 -0.00 -0.00 -0.00 0.00 0.00

37 1 -0.00 -0.00 0.00 0.00 0.00 0.00 -0.00 0.00 -0.00

38 1 -0.00 -0.00 0.00 -0.00 0.00 -0.00 0.00 0.00 0.00

39 6 -0.00 0.00 -0.00 -0.00 0.00 0.00 0.00 0.00 -0.00

40 8 -0.00 -0.00 0.00 0.00 -0.00 -0.00 -0.00 -0.00 -0.00

41 8 -0.00 0.00 -0.00 0.00 -0.00 0.00 -0.00 -0.00 0.00

42 6 0.00 -0.00 0.00 -0.00 0.00 -0.00 -0.00 0.00 -0.00

43 6 -0.04 -0.03 -0.04 0.00 0.00 0.00 0.00 0.00 -0.00

44 6 0.00 0.00 0.00 -0.00 0.00 -0.00 -0.00 -0.00 -0.00

45 6 0.03 -0.02 0.03 -0.00 0.00 -0.00 0.00 -0.00 0.00

46 1 0.45 0.37 0.42 -0.00 -0.00 -0.00 0.00 0.00 0.00

47 6 0.01 0.01 0.01 -0.00 -0.00 -0.00 0.00 0.00 0.00

48 6 -0.00 -0.04 0.00 0.00 0.00 -0.00 -0.00 -0.00 0.00

49 1 -0.28 0.24 -0.33 0.00 -0.00 0.00 -0.02 0.02 -0.02

50 1 -0.08 -0.07 -0.07 0.00 0.00 0.00 -0.00 -0.00 -0.00

51 1 0.03 0.46 -0.03 -0.00 -0.00 0.00 0.00 0.02 -0.00

52 8 -0.00 0.00 -0.00 -0.00 -0.00 0.00 0.00 -0.00 0.00

53 6 -0.00 0.00 -0.00 0.00 0.00 0.00 -0.00 0.00 -0.00

54 1 0.00 -0.00 -0.00 0.00 -0.00 -0.00 0.00 -0.00 -0.00

55 1 -0.00 -0.00 0.00 -0.00 -0.00 0.00 -0.00 -0.00 0.00

56 1 0.00 -0.01 0.00 0.00 -0.00 0.00 0.00 -0.00 0.00

157 158 159

A A A

Frequencies -- 3215.6944 3217.5672 3222.8098

Red. masses -- 1.0963 1.0961 1.0946

Frc consts -- 6.6795 6.6860 6.6982

IR Inten -- 12.9767 6.3748 8.0445

Atom AN X Y Z X Y Z X Y Z

1 16 0.00 -0.00 0.00 0.00 -0.00 -0.00 -0.00 0.00 0.00

2 6 -0.00 -0.00 -0.00 0.00 -0.00 0.00 0.00 0.00 -0.00

3 6 0.00 0.00 -0.00 -0.00 0.00 0.00 -0.00 0.00 -0.00

4 6 0.00 -0.00 -0.00 0.00 -0.00 -0.00 -0.00 0.00 0.00

5 6 -0.00 0.00 0.00 -0.00 0.00 0.00 0.00 -0.00 0.00

6 6 0.00 -0.00 -0.00 0.02 -0.07 -0.00 0.00 -0.01 -0.00

7 6 -0.00 0.00 0.00 0.01 -0.01 -0.01 -0.06 0.02 0.05

8 6 0.00 -0.00 -0.00 0.03 -0.01 -0.03 0.01 -0.00 -0.01

9 1 -0.00 0.00 0.00 -0.20 0.74 0.02 -0.02 0.07 0.00

10 6 -0.00 0.00 -0.00 -0.00 0.01 0.00 -0.01 0.03 -0.00

11 1 0.00 -0.00 -0.00 -0.14 0.06 0.13 0.63 -0.28 -0.61

12 6 0.00 0.00 -0.00 0.01 0.02 -0.02 0.01 0.01 -0.01

13 1 -0.00 0.00 0.00 -0.36 0.17 0.35 -0.08 0.04 0.08

14 1 0.00 -0.00 -0.00 0.02 -0.07 -0.00 0.09 -0.31 0.00

15 1 -0.00 -0.00 0.00 -0.11 -0.18 0.18 -0.06 -0.10 0.10

16 8 -0.00 0.00 0.00 0.00 0.00 -0.00 0.00 -0.00 -0.00

17 8 0.00 0.00 -0.00 0.00 0.00 0.00 0.00 0.00 -0.00

18 6 0.00 0.00 -0.00 -0.00 0.00 -0.00 -0.00 0.00 -0.00

19 1 0.00 0.00 0.00 0.00 -0.00 0.00 0.00 -0.00 0.00

20 6 -0.00 0.00 0.00 -0.00 0.00 -0.00 -0.00 -0.00 -0.00

21 6 -0.00 -0.00 0.00 -0.00 -0.00 0.00 -0.00 0.00 -0.00

22 6 0.00 -0.00 0.00 -0.00 0.00 -0.00 -0.00 0.00 -0.00

23 6 0.00 0.00 0.00 -0.00 0.00 0.00 0.00 -0.00 -0.00

24 6 0.00 0.00 -0.00 -0.00 -0.00 0.00 -0.00 -0.00 0.00

25 1 0.00 0.00 -0.00 0.00 0.00 -0.00 0.00 -0.00 0.00

26 6 -0.00 -0.00 0.00 0.00 0.00 -0.00 0.00 0.00 -0.00

27 6 0.00 0.00 -0.00 0.00 0.00 -0.00 -0.00 0.00 -0.00

28 1 -0.00 -0.00 -0.00 0.00 -0.00 -0.00 0.00 0.00 0.00

29 6 -0.00 0.00 0.00 0.00 -0.00 0.00 0.00 -0.00 0.00

30 1 -0.00 -0.02 0.00 0.00 0.00 -0.00 0.00 0.00 -0.00

31 1 0.00 0.00 -0.00 -0.00 -0.00 0.00 -0.00 -0.00 0.00

32 1 -0.00 -0.00 -0.00 -0.00 -0.00 0.00 -0.00 -0.00 -0.00

33 1 0.01 -0.01 -0.00 -0.00 0.00 -0.00 -0.00 0.00 0.00

34 8 -0.00 0.00 0.00 -0.00 0.00 -0.00 0.00 -0.00 -0.00

35 6 -0.00 -0.00 -0.00 0.00 -0.00 0.00 -0.00 0.00 -0.00

36 1 -0.00 0.00 -0.00 -0.00 0.00 0.00 0.02 -0.01 -0.00

37 1 0.00 0.00 0.00 -0.00 -0.00 -0.00 0.00 -0.00 0.00

38 1 0.00 0.00 -0.00 0.00 0.00 -0.00 -0.00 -0.00 0.00

39 6 -0.00 0.00 0.00 -0.00 -0.00 0.00 0.00 -0.00 0.00

40 8 0.00 -0.00 -0.00 0.00 0.00 -0.00 -0.00 0.00 0.00

41 8 -0.00 0.00 -0.00 0.00 0.00 -0.00 0.00 0.00 -0.00

42 6 0.00 -0.00 0.00 -0.00 -0.00 -0.00 -0.00 -0.00 -0.00

43 6 -0.04 -0.03 -0.03 0.00 0.00 0.00 0.00 0.00 0.00

44 6 0.00 0.00 0.00 0.00 -0.00 0.00 0.00 -0.00 0.00

45 6 -0.03 0.03 -0.04 0.00 -0.00 0.00 0.00 -0.00 0.00

46 1 0.40 0.34 0.38 -0.00 -0.00 -0.00 -0.00 -0.00 -0.00

47 6 -0.01 -0.01 -0.01 0.00 0.00 0.00 0.00 0.00 0.00

48 6 0.00 0.02 0.00 -0.00 -0.00 -0.00 -0.00 0.00 -0.00

49 1 0.39 -0.34 0.45 -0.00 0.00 -0.00 -0.00 0.00 -0.00

50 1 0.14 0.11 0.13 -0.00 -0.00 -0.00 -0.00 -0.00 -0.00

51 1 -0.02 -0.21 0.01 0.00 0.00 -0.00 -0.00 -0.00 0.00

52 8 -0.00 -0.00 -0.00 0.00 0.00 0.00 0.00 0.00 0.00

53 6 0.00 -0.00 0.00 0.00 -0.00 -0.00 -0.00 -0.00 -0.00

54 1 -0.00 0.00 0.00 -0.00 0.00 0.00 -0.00 0.00 0.00

55 1 0.00 0.00 -0.00 -0.00 -0.00 0.00 0.00 0.00 -0.00

56 1 -0.00 0.01 -0.00 0.00 -0.00 0.00 0.00 -0.00 0.00

160 161 162

A A A

Frequencies -- 3227.7118 3236.3352 3306.3342

Red. masses -- 1.0929 1.0927 1.0881

Frc consts -- 6.7082 6.7431 7.0083

IR Inten -- 6.4654 5.2706 0.3858

Atom AN X Y Z X Y Z X Y Z

1 16 -0.00 0.00 -0.00 0.00 -0.00 0.00 -0.00 -0.00 -0.00

2 6 0.00 0.00 0.00 0.00 -0.00 0.00 0.00 -0.00 0.00

3 6 -0.00 -0.00 -0.00 -0.00 0.00 0.00 -0.00 -0.00 -0.00

4 6 -0.00 0.00 0.00 0.00 0.00 0.00 0.00 -0.00 0.00

5 6 0.00 -0.00 0.00 -0.00 0.00 0.00 -0.00 0.00 0.00

6 6 0.00 -0.00 0.00 0.00 -0.00 -0.00 -0.00 0.00 -0.00

7 6 -0.00 0.00 0.00 0.00 -0.00 -0.00 0.00 -0.00 -0.00

8 6 0.00 -0.00 -0.00 -0.00 0.00 0.00 -0.00 0.00 0.00

9 1 -0.00 0.00 -0.00 -0.00 0.00 0.00 0.00 -0.00 -0.00

10 6 -0.00 0.00 -0.00 0.00 -0.00 0.00 0.00 -0.00 0.00

11 1 0.00 -0.00 -0.00 -0.00 0.00 0.00 -0.00 0.00 0.00

12 6 0.00 0.00 -0.00 -0.00 -0.00 0.00 0.00 -0.00 -0.00

13 1 -0.00 0.00 0.00 0.00 -0.00 -0.00 0.00 -0.00 -0.00

14 1 0.00 -0.00 -0.00 -0.00 0.00 -0.00 -0.00 0.00 -0.00

15 1 -0.00 -0.00 0.00 0.00 0.00 -0.00 -0.00 0.00 -0.00

16 8 0.00 -0.00 -0.00 -0.00 0.00 0.00 0.00 0.00 0.00

17 8 0.00 -0.00 0.00 -0.00 0.00 -0.00 0.00 0.00 0.00

18 6 0.00 -0.00 0.00 -0.00 -0.00 -0.00 -0.00 -0.00 -0.00

19 1 -0.00 -0.00 -0.00 0.00 -0.00 0.00 0.00 -0.00 0.00

20 6 -0.00 -0.00 0.00 0.00 0.00 0.00 -0.00 0.00 -0.00

21 6 0.00 0.00 -0.00 -0.00 0.00 0.00 -0.08 -0.03 0.02

22 6 -0.00 -0.00 -0.00 0.00 0.00 0.00 0.00 0.00 0.00

23 6 -0.00 -0.00 -0.00 -0.01 -0.08 -0.01 0.00 -0.00 -0.00

24 6 -0.00 -0.00 0.00 0.00 0.00 -0.00 0.00 -0.01 0.00

25 1 -0.00 -0.00 0.00 -0.00 -0.00 -0.00 0.88 0.41 -0.21

26 6 0.00 0.00 -0.00 -0.00 -0.00 0.00 0.00 0.00 -0.00

27 6 -0.00 0.00 0.00 -0.02 -0.00 0.00 -0.00 0.00 0.00

28 1 0.00 0.00 0.00 0.11 0.97 0.12 0.00 0.00 0.00

29 6 0.00 -0.00 0.00 0.00 0.00 0.00 0.00 0.00 -0.00

30 1 0.00 0.00 -0.00 -0.00 -0.00 0.00 0.00 0.06 -0.01

31 1 -0.00 -0.00 0.00 0.01 0.00 -0.00 -0.01 -0.00 0.00

32 1 0.00 0.00 -0.00 0.16 0.06 -0.01 -0.00 -0.00 0.00

33 1 -0.00 0.00 -0.00 0.00 -0.00 0.00 -0.01 0.00 0.00

34 8 0.00 -0.00 -0.00 -0.00 -0.00 -0.00 0.00 -0.00 -0.00

35 6 -0.00 0.00 -0.00 -0.00 -0.00 -0.00 -0.00 0.00 0.00

36 1 0.00 -0.00 -0.00 0.02 -0.01 -0.00 0.00 -0.00 -0.00

37 1 -0.00 -0.00 -0.00 0.02 0.00 0.02 -0.00 -0.00 -0.00

38 1 -0.00 -0.00 0.00 0.01 0.01 -0.01 -0.00 0.00 -0.00

39 6 0.00 0.00 -0.00 -0.00 0.00 -0.00 0.00 0.00 -0.00

40 8 -0.00 -0.00 0.00 0.00 -0.00 -0.00 -0.00 0.00 -0.00

41 8 -0.00 -0.00 0.00 -0.00 -0.00 0.00 -0.00 -0.00 0.00

42 6 0.00 0.00 0.00 0.00 0.00 -0.00 -0.00 -0.00 0.00

43 6 0.00 0.00 0.00 -0.00 -0.00 -0.00 0.00 0.00 0.00

44 6 -0.00 0.00 -0.00 0.00 -0.00 0.00 -0.00 0.00 -0.00

45 6 0.01 -0.01 0.01 -0.00 0.00 -0.00 0.00 -0.00 0.00

46 1 -0.05 -0.05 -0.05 0.00 0.00 0.00 -0.00 -0.00 -0.00

47 6 -0.05 -0.04 -0.05 0.00 0.00 0.00 -0.00 -0.00 -0.00

48 6 -0.00 -0.02 0.00 0.00 0.00 -0.00 -0.00 -0.00 -0.00

49 1 -0.08 0.06 -0.09 0.00 -0.00 0.00 -0.00 0.00 -0.00

50 1 0.60 0.48 0.55 -0.00 -0.00 -0.00 0.00 0.00 0.00

51 1 0.02 0.26 -0.02 -0.00 -0.00 0.00 -0.00 0.00 0.00

52 8 -0.00 -0.00 -0.00 -0.00 -0.00 -0.00 0.00 -0.00 0.00

53 6 0.00 -0.00 0.00 0.00 0.00 0.00 -0.00 -0.00 -0.00

54 1 -0.01 0.01 0.01 0.00 -0.00 -0.00 -0.00 0.00 0.00

55 1 0.01 0.01 -0.01 -0.00 -0.00 0.00 0.00 0.00 0.00

56 1 -0.01 0.02 -0.01 -0.00 0.00 -0.00 0.00 -0.00 0.00
