# Supplementary material for: Carbene-catalyzed atroposelective synthesis of axially chiral styrenes
Source: Nat Commun. 2022 Jan 10;13:84. doi: 10.1038/s41467-021-27771-x (PMC8748895; doi:10.1038/s41467-021-27771-x)
Supplement: Supplementary file 3 — Source Data [file 41467_2021_27771_MOESM3_ESM.zip › Cartesian Coordinates and Energies for the Optimized Structures of 9a, TS9a, and ent-9a.docx]

Supplementary Data

## **Cartesian Coordinates and Energies for the Optimized Structures of 9a, TS9a, and *ent*-9a**


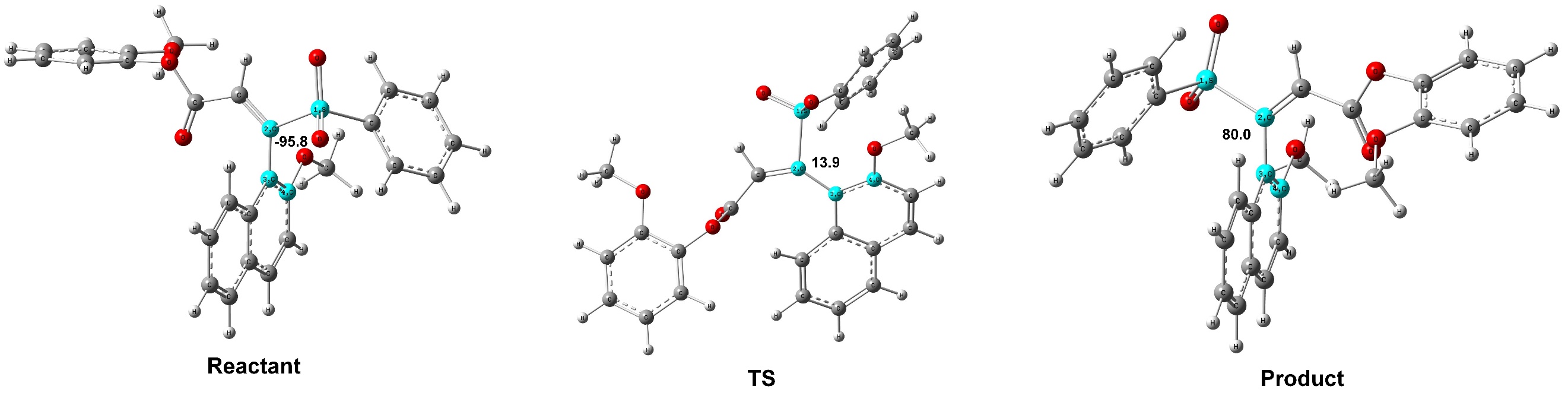
**9a**

Sum of electronic and thermal Free Energies @ B3lYP/6-31g(d,p) = -1890.94142800 a.u. Single point energy @ M06-2X/Def2-TZVP = -1891.56059590 a.u

S -3.35266200 3.68797100 -1.20061200

C -2.22046500 2.35624700 -1.79565300

C -0.79157700 2.71249000 -1.96228200

C -0.34469100 3.08687500 -3.22967300

C -3.21881800 5.01559000 -2.40595600

C -4.04776900 4.99754500 -3.53013700

C -2.31700800 6.05523500 -2.17382900

C -3.96213400 6.04576700 -4.44499200

H -4.75357200 4.18675100 -3.67326200

C -2.24071400 7.09738200 -3.09897500

H -1.70351400 6.05201700 -1.28005100

C -3.05875200 7.09092100 -4.23077900

H -4.60605600 6.05147200 -5.31928300

H -1.54767900 7.91589500 -2.93008100

H -2.99906100 7.90738600 -4.94472000

O -2.80093100 4.18252800 0.07477300

O -4.73361800 3.17053300 -1.25544300

C -2.81950000 1.17383700 -1.98247800

H -3.88893800 1.08581500 -1.82069300

C 0.12772500 2.66207600 -0.86692900

C -0.25219000 2.26084900 0.44466200

C 1.49949500 3.01640200 -1.09301900

C 1.00981100 3.44426500 -3.44078600

C 0.66959900 2.21531000 1.46579100

H -1.28526400 2.00021200 0.64106300

C 2.42370900 2.96120300 -0.01455500

C 1.90055000 3.40746300 -2.39416600

H 1.34866900 3.73760300 -4.42671400

C 2.02213400 2.56836500 1.24041100

H 0.35405200 1.90680600 2.45840900

H 3.45896500 3.23264000 -0.20497400

H 2.93949900 3.67827700 -2.56279000

H 2.73577500 2.52712700 2.05800500

O -1.27527300 3.08121800 -4.22189200

C -0.90743600 3.52823200 -5.52280600

H -1.81797000 3.47270000 -6.12167400

H -0.55151200 4.56510100 -5.50934100

H -0.14432100 2.88189000 -5.97261500

C -2.11224000 -0.06667800 -2.39693400

O -0.96198700 -0.17846500 -2.74487200

O -2.99397300 -1.11182900 -2.30774000

C -2.55647200 -2.37343800 -2.71518700

C -2.28537900 -3.33834800 -1.75825300

C -2.48154300 -2.66387500 -4.09029200

C -1.92348000 -4.62936800 -2.15477400

H -2.36263500 -3.06883600 -0.70977400

C -2.11479900 -3.95656600 -4.47917300

C -1.83819300 -4.92830200 -3.51190700

H -1.71010700 -5.38618000 -1.40698300

H -2.04524300 -4.21080800 -5.53014200

H -1.55634200 -5.92669500 -3.83342100

O -2.78724600 -1.64414500 -4.93387600

C -2.69028500 -1.87985800 -6.33458500

H -1.66912500 -2.15189200 -6.62801000

H -3.38548300 -2.66282100 -6.66194500

H -2.96211100 -0.93815300 -6.81454000


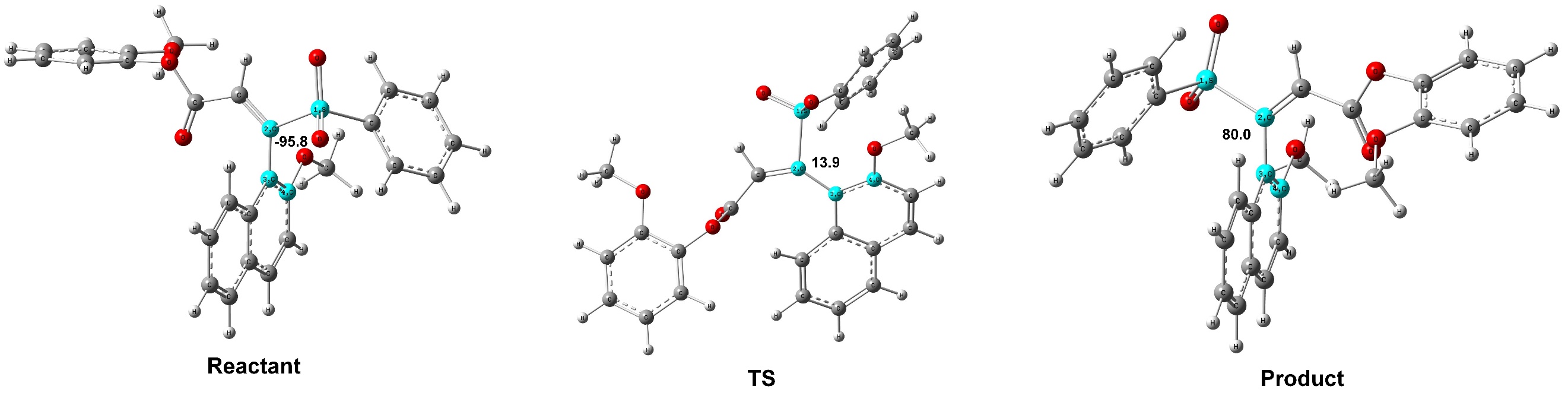
**TS9a**

Sum of electronic and thermal Free Energies @ B3lYP/6-31g(d,p) = -1890.89338400 a.u

Single point energy @ M06-2X/Def2-TZVP = -1891.56059590 a.u

S -3.33860000 3.76500000 -3.18960000

C -2.01820000 2.50790000 -2.55020000

C -0.68750000 2.96870000 -2.06110000

C -0.44460000 4.32710000 -1.77290000

C -2.46420000 4.93670000 -4.24690000

C -1.52410000 4.47300000 -5.16840000

C -2.86800000 6.27150000 -4.22340000

C -0.95690000 5.37880000 -6.06540000

H -1.23770000 3.42660000 -5.19070000

C -2.30350000 7.16390000 -5.13600000

H -3.61200000 6.59730000 -3.50500000

C -1.34610000 6.72010000 -6.05080000

H -0.21740000 5.03160000 -6.78050000

H -2.61400000 8.20450000 -5.13150000

H -0.90710000 7.41860000 -6.75710000

O -4.04490000 4.46500000 -2.10320000

O -4.18920000 2.99420000 -4.12730000

C -2.55060000 1.30110000 -2.83100000

H -3.52670000 1.33610000 -3.31970000

C 0.47650000 2.09890000 -1.96040000

C 0.40740000 0.70120000 -1.73980000

C 1.79870000 2.66380000 -1.98890000

C 0.86350000 4.87500000 -1.74400000

C 1.53190000 -0.09920000 -1.71190000

H -0.53700000 0.25720000 -1.47830000

C 2.93920000 1.81940000 -1.99680000

C 1.95470000 4.06980000 -1.92480000

H 0.99080000 5.94120000 -1.60890000

C 2.81610000 0.45300000 -1.89070000

H 1.42060000 -1.16370000 -1.52770000

H 3.91990000 2.28440000 -2.05520000

H 2.95330000 4.49630000 -1.95790000

H 3.69420000 -0.18530000 -1.89020000

O -1.52300000 5.10110000 -1.54180000

C -1.38240000 6.47560000 -1.19410000

H -2.39610000 6.82530000 -0.99800000

H -0.78070000 6.59050000 -0.28570000

H -0.94400000 7.06260000 -2.00810000

C -2.15060000 -0.13370000 -2.79540000

O -1.60370000 -0.71060000 -3.70090000

O -2.64970000 -0.73770000 -1.67420000

C -2.68060000 -2.13740000 -1.63790000

C -1.88590000 -2.81090000 -0.72480000

C -3.59230000 -2.82210000 -2.46390000

C -1.96880000 -4.20370000 -0.62630000

H -1.21550000 -2.23960000 -0.09060000

C -3.66270000 -4.21550000 -2.36520000

C -2.85180000 -4.89570000 -1.45070000

H -1.34730000 -4.73410000 0.08780000

H -4.34890000 -4.77200000 -2.99240000

H -2.92300000 -5.97750000 -1.38650000

O -4.34240000 -2.05000000 -3.28990000

C -5.24910000 -2.69460000 -4.18050000

H -4.72460000 -3.36520000 -4.87160000

H -6.01750000 -3.25630000 -3.63540000

H -5.72590000 -1.89440000 -4.74850000


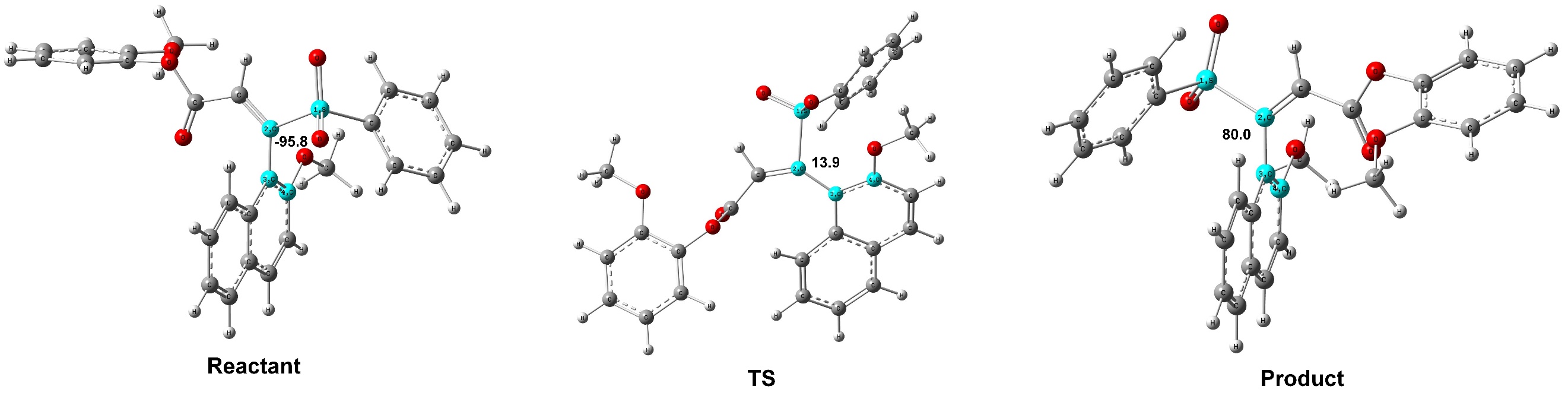
*ent-***9a**

Sum of electronic and thermal Free Energies @ B3lYP/6-31g(d,p) = -1890.94043300 a.u

Single point energy @ M06-2X/Def2-TZVP = -1891.60763237 a.u

S -3.08421200 2.95693700 -3.37016800

C -2.12115000 2.70045500 -1.80699700

C -0.66603500 2.95801400 -1.88211800

C -0.23399800 4.28247200 -1.81570000

C -2.75473900 1.48923200 -4.36075700

C -3.56159000 0.36015600 -4.19535300

C -1.71744000 1.51832000 -5.29485900

C -3.31103900 -0.76659100 -4.97725300

H -4.37862600 0.37350700 -3.48220700

C -1.47872700 0.38394500 -6.07103600

H -1.12237000 2.41616200 -5.41638400

C -2.27008300 -0.75511600 -5.90970000

H -3.93314900 -1.64921500 -4.86410100

H -0.67677600 0.39289200 -6.80279000

H -2.07969700 -1.63523700 -6.51692000

O -2.50315000 4.10778100 -4.08116900

O -4.51672200 2.93947300 -3.01534400

C -2.86103900 2.33531900 -0.75295700

H -3.92896600 2.18563300 -0.87163300

C 0.28571600 1.90143000 -2.03530400

C -0.07868800 0.52576200 -2.07008100

C 1.67621900 2.23270100 -2.16247600

C 1.14046500 4.60001000 -1.95037900

C 0.87271900 -0.45704500 -2.22837900

H -1.12262000 0.25063900 -1.96944300

C 2.63099500 1.19264100 -2.32440700

C 2.06305700 3.59569600 -2.12299800

H 1.46570200 5.63236900 -1.90793200

C 2.24326100 -0.12649200 -2.35816500

H 0.56703200 -1.49936400 -2.25523200

H 3.67964600 1.46320800 -2.41898500

H 3.11632100 3.84372400 -2.22427200

H 2.98135700 -0.91344300 -2.48212000

O -1.19355600 5.21336100 -1.58754300

C -0.88844200 6.59074000 -1.78653300

H -1.83391400 7.12211100 -1.66687300

H -0.17547100 6.96086400 -1.03985000

H -0.49895200 6.77413700 -2.79432700

C -2.31689900 2.13563000 0.61642300

O -1.22094200 2.43441300 1.02277600

O -3.29625800 1.56378900 1.38676300

C -2.97560300 1.20457500 2.69671400

C -3.56195300 1.89419100 3.74606300

C -2.13358600 0.09971100 2.92526000

C -3.31199600 1.49938300 5.06390800

H -4.20915900 2.73546500 3.51947400

C -1.88239000 -0.28419100 4.24669300

C -2.47116300 0.41603600 5.30479700

H -3.76894100 2.03891200 5.88711300

H -1.23466000 -1.12735900 4.45599200

H -2.26610700 0.10137700 6.32385800

O -1.63997700 -0.51297000 1.81867900

C -0.68296700 -1.55315300 1.99402700

H 0.19475900 -1.20168300 2.54913400

H -1.11775300 -2.42101500 2.50538700

H -0.37529800 -1.84542800 0.98892600
