# Supplementary material for: Carbene-catalyzed atroposelective synthesis of axially chiral styrenes
Source: Nat Commun. 2022 Jan 10;13:84. doi: 10.1038/s41467-021-27771-x (PMC8748895; doi:10.1038/s41467-021-27771-x)
Supplement: Supplementary file 3 — Source Data [file 41467_2021_27771_MOESM3_ESM.zip › The IRC calculation result for TS9a.docx]

Supplementary Data

## **The IRC calculation result for TS9a**

# Total Energy along IRC (Forward) for **TS9a**

# X-Axis: Intrinsic Reaction Coordinate

# Y-Axis: Total Energy (Hartree)

# X Y

0.0000000000 -1891.5831382400

0.1700900000 -1891.5843551000

0.4166400000 -1891.5861493100

0.6593000000 -1891.5871142900

0.9024100000 -1891.5876744600

1.1493800000 -1891.5881130700

1.3968000000 -1891.5884875900

1.6442500000 -1891.5888100000

1.8916700000 -1891.5890912100

2.1391100000 -1891.5893416300

2.3866100000 -1891.5895703000

2.6341900000 -1891.5897841500

2.8818500000 -1891.5899880700

3.1295600000 -1891.5901851600

3.3773000000 -1891.5903773400

3.6250700000 -1891.5905658400

3.8728400000 -1891.5907515100

4.1206300000 -1891.5909350600

4.3684200000 -1891.5911170200

4.6162200000 -1891.5912977900

4.8640200000 -1891.5914776700

5.1118300000 -1891.5916568400

5.3596300000 -1891.5918354300

5.6074400000 -1891.5920135000

5.8552400000 -1891.5921911100

6.1030500000 -1891.5923682900

6.3508600000 -1891.5925450800

6.5986700000 -1891.5927215000

6.8464800000 -1891.5928975900

7.0942800000 -1891.5930734100

7.3420900000 -1891.5932490600

7.5899000000 -1891.5934246600

7.8377100000 -1891.5936003600

8.0855200000 -1891.5937762800

8.3333300000 -1891.5939525700

8.5811300000 -1891.5941292900

8.8288800000 -1891.5943064900

9.0766000000 -1891.5944845600

9.3242200000 -1891.5946632900

9.5718300000 -1891.5948431300

9.8195600000 -1891.5950239100

10.0673000000 -1891.5952055500

10.3150800000 -1891.5953887600

10.5628200000 -1891.5955716600

10.8105900000 -1891.5957561400

11.0583400000 -1891.5959403600

11.3060900000 -1891.5961257800

11.5538700000 -1891.5963115800

11.8016300000 -1891.5964978300

12.0493900000 -1891.5966849900

12.2970700000 -1891.5968718600

12.5447000000 -1891.5970605500

12.7923400000 -1891.5972493100

13.0401000000 -1891.5974384900

13.2878600000 -1891.5976285600

13.5356500000 -1891.5978195600

13.7834000000 -1891.5980103400

14.0310700000 -1891.5982012600

14.2788200000 -1891.5983937300

14.5265800000 -1891.5985865600

14.7743600000 -1891.5987797600

15.0220800000 -1891.5989729000

15.2697400000 -1891.5991669400

15.5172100000 -1891.5993611600

15.7647100000 -1891.5995556400

16.0122500000 -1891.5997506100

16.2600100000 -1891.5999459600

16.5077500000 -1891.6001416800

16.7554400000 -1891.6003373500

17.0030100000 -1891.6005326100

17.2503100000 -1891.6007277500

17.4975800000 -1891.6009229700

17.7450200000 -1891.6011181900

17.9927000000 -1891.6013136100

18.2404500000 -1891.6015091700

18.4882400000 -1891.6017043600

18.7360100000 -1891.6018996300

18.9837700000 -1891.6020948600

19.2314500000 -1891.6022895500

19.4791500000 -1891.6024842600

19.7267900000 -1891.6026789800

# Total Energy along IRC (Reverse) for **TS9a**

# X-Axis: Intrinsic Reaction Coordinate

# Y-Axis: Total Energy (Hartree)

# X Y

-21.7383800000 -1891.6043608200

-21.4908100000 -1891.6041681100

-21.2431500000 -1891.6039744900

-20.9958100000 -1891.6037809800

-20.7481100000 -1891.6035871000

-20.5004600000 -1891.6033929000

-20.2527500000 -1891.6031982800

-20.0049500000 -1891.6030033300

-19.7572300000 -1891.6028086800

-19.5094500000 -1891.6026138200

-19.2618800000 -1891.6024190200

-19.0142400000 -1891.6022241000

-18.7667000000 -1891.6020295900

-18.5190600000 -1891.6018341300

-18.2715800000 -1891.6016394900

-18.0239700000 -1891.6014443600

-17.7764600000 -1891.6012495000

-17.5288100000 -1891.6010541100

-17.2810700000 -1891.6008585000

-17.0332700000 -1891.6006629200

-16.7856100000 -1891.6004674800

-16.5378500000 -1891.6002722200

-16.2901600000 -1891.6000765600

-16.0424000000 -1891.5998812300

-15.7946000000 -1891.5996854800

-15.5468700000 -1891.5994910800

-15.2990900000 -1891.5992962000

-15.0516000000 -1891.5991026500

-14.8040300000 -1891.5989090600

-14.5568500000 -1891.5987165300

-14.3095600000 -1891.5985241400

-14.0622600000 -1891.5983324600

-13.8148000000 -1891.5981410400

-13.5672800000 -1891.5979500900

-13.3196600000 -1891.5977593000

-13.0719400000 -1891.5975690500

-12.8242000000 -1891.5973796200

-12.5764200000 -1891.5971904600

-12.3286600000 -1891.5970021000

-12.0808700000 -1891.5968142700

-11.8330800000 -1891.5966268700

-11.5853200000 -1891.5964401600

-11.3375700000 -1891.5962537000

-11.0898700000 -1891.5960687700

-10.8421100000 -1891.5958839700

-10.5943300000 -1891.5956998500

-10.3465400000 -1891.5955158200

-10.0987600000 -1891.5953329200

-9.8510200000 -1891.5951506400

-9.6034300000 -1891.5949690100

-9.3559600000 -1891.5947888400

-9.1083900000 -1891.5946096400

-8.8607000000 -1891.5944314900

-8.6129100000 -1891.5942538900

-8.3651000000 -1891.5940769200

-8.1172900000 -1891.5939005200

-7.8694800000 -1891.5937245600

-7.6216700000 -1891.5935489400

-7.3738600000 -1891.5933735100

-7.1260600000 -1891.5931981500

-6.8782500000 -1891.5930227000

-6.6304400000 -1891.5928470600

-6.3826300000 -1891.5926711400

-6.1348200000 -1891.5924948900

-5.8870100000 -1891.5923182800

-5.6392100000 -1891.5921412700

-5.3914000000 -1891.5919638300

-5.1436000000 -1891.5917859200

-4.8957900000 -1891.5916074800

-4.6480000000 -1891.5914284200

-4.4002000000 -1891.5912486000

-4.1524200000 -1891.5910678000

-3.9046400000 -1891.5908856800

-3.6568900000 -1891.5907018100

-3.4091500000 -1891.5905156100

-3.1614500000 -1891.5903262400

-2.9138000000 -1891.5901325600

-2.6662000000 -1891.5899326200

-2.4186500000 -1891.5897230300

-2.1711600000 -1891.5894983300

-1.9236900000 -1891.5892508500

-1.6762200000 -1891.5889711100

-1.4287200000 -1891.5886486100

-1.1812600000 -1891.5882725800

-0.9343400000 -1891.5878261800

-0.6918200000 -1891.5872560900

-0.4488400000 -1891.5862632400

-0.2025900000 -1891.5844149400

0.0000000000 -1891.5831382400
